# The HD Reaction of Nitrogenase: a Detailed Mechanism

**DOI:** 10.1002/chem.202202502

**Published:** 2022-11-29

**Authors:** Ian Dance

**Affiliations:** ^1^ School of Chemistry UNSW Sydney Australia

**Keywords:** density functional calculations, enzyme catalysis, HD reaction, nitrogenases, reaction mechanisms

## Abstract

Nitrogenase is the enzyme that converts N_2_ to NH_3_ under ambient conditions. The chemical mechanism of this catalysis at the active site FeMo‐co [Fe_7_S_9_CMo(homocitrate)] is unknown. An obligatory co‐product is H_2_, while exogenous H_2_ is a competitive inhibitor. Isotopic substitution using exogenous D_2_ revealed the N_2_‐dependent reaction D_2_+2H^+^+2e^−^→2HD (the ‘HD reaction’), together with a collection of additional experimental characteristics and requirements. This paper describes a detailed mechanism for the HD reaction, developed and elaborated using density functional simulations with a 486‐atom model of the active site and surrounding protein. First D_2_ binds at one Fe atom (*endo*‐Fe6 coordination position), where it is flanked by H−Fe6 (*exo* position) and H−Fe2 (*endo* position). Then there is synchronous transfer of these two H atoms to bound D_2_, forming one HD bound to Fe2 and a second HD bound to Fe6. These two HD dissociate sequentially. The final phase is recovery of the two flanking H atoms. These H atoms are generated, sequentially, by translocation of a proton from the protein surface to S3B of FeMo‐co and combination with introduced electrons. The first H atom migrates from S3B to *exo*‐Fe6 and the second from S3B to *endo*‐Fe2. Reaction energies and kinetic barriers are reported for all steps. This mechanism accounts for the experimental data: (a) stoichiometry; (b) the N_2_‐dependence results from promotional N_2_ bound at *exo*‐Fe2; (c) different N_2_ binding K_m_ for the HD reaction and the NH_3_ formation reaction results from involvement of two different sites; (d) inhibition by CO; (e) the non‐occurrence of 2HD→H_2_+D_2_ results from the synchronicity of the two transfers of H to D_2_; (f) inhibition of HD production at high pN_2_ is by competitive binding of N_2_ at *endo*‐Fe6; (g) the non‐leakage of D to solvent follows from the hydrophobic environment and irreversibility of proton introduction.

## Graphical abstract

**A detailed mechanism** is presented for the reaction D +2H^+^+2e^−^→2HD of the enzyme nitrogenase, developed with density functional simulations of a 486‐atom model of the active site FeMo‐co and surrounding protein. It explains the accumulated experimental data and connects with the enigmatic N_2_→NH_3_ reaction of the enzyme.

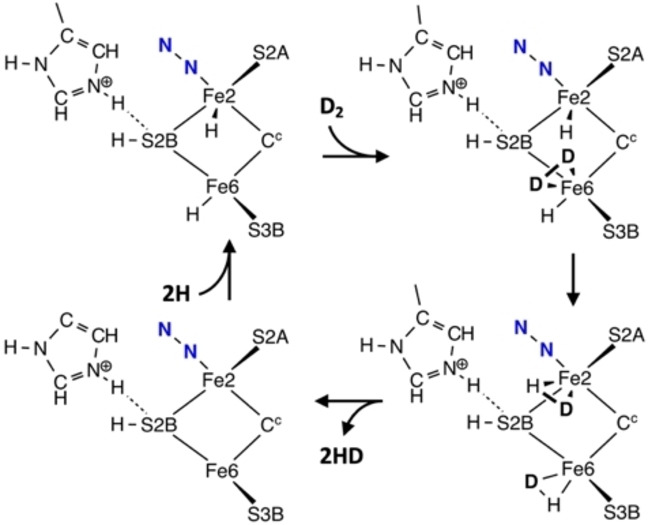

## Introduction

**Figure 1:**
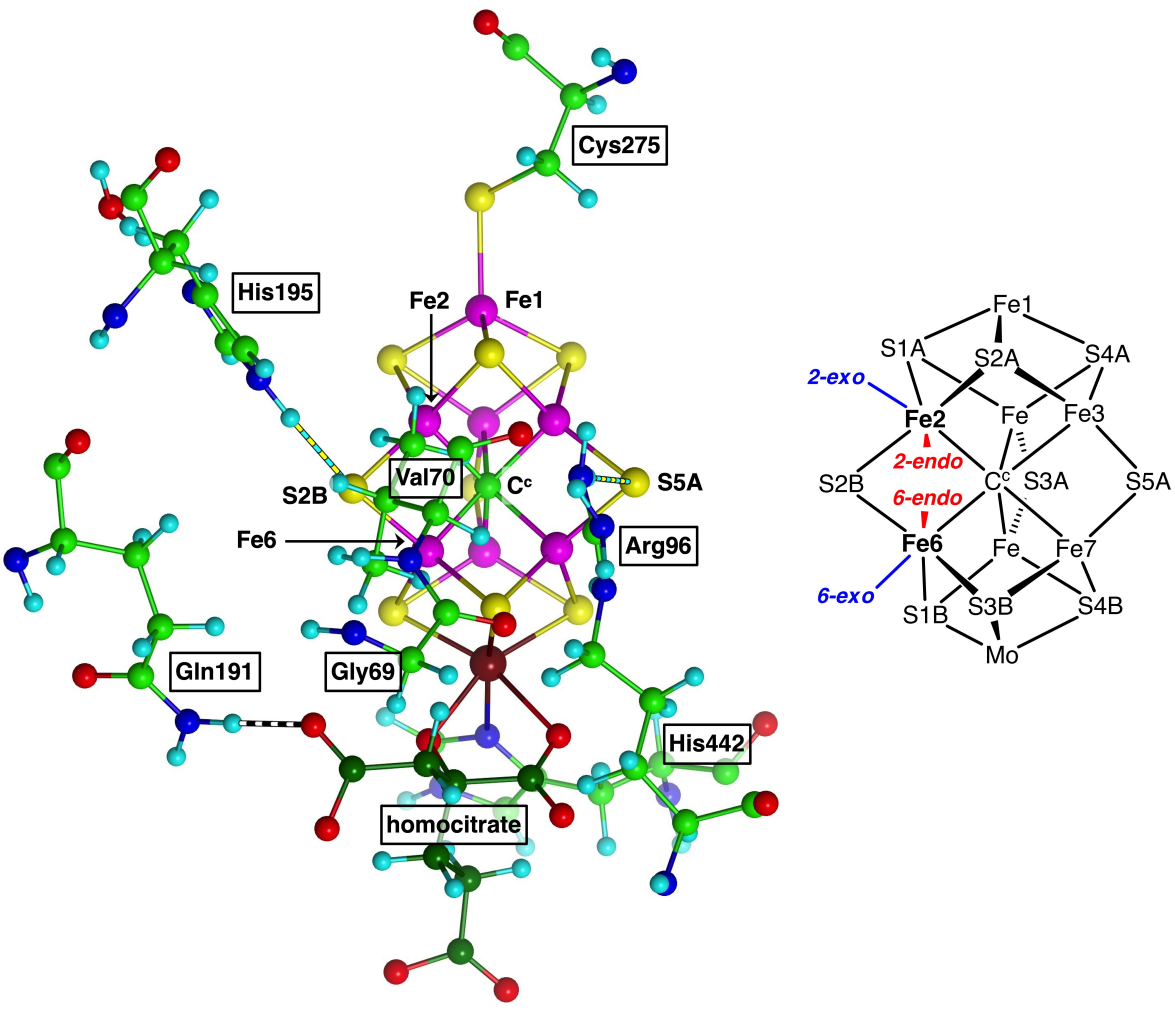
(a) FeMo‐co, the active site of nitrogenase, an Fe_7_MoS_9_C^c^ cluster with bidentate coordination of Mo by homocitrate and ligation by His442 at Mo and Cys275 at Fe1. C^c^ is a central carbon atom bonded equally to six Fe atoms in the resting state. Fe magenta, Mo brown, C atoms of homocitrate are dark green. The positions of the significant amino acids Val70, Arg96, Gln191 and His195 surrounding FeMo‐co are marked. Residue numbering is from the α‐subunit of *Azotobacter vinelandii*, crystal structure PDB 3 U7Q.^[2]^ Hydrogen bonds are striped. (b) Atom labels for the cluster, and identification of the potential coordination positions at Fe2 and Fe6, *exo* or *endo* relative to the Fe−C^c^ bond. S2B, S5A and S3A are often referred to as ‘belt’ atoms.

Nitrogenase is the enzyme that converts unreactive atmospheric nitrogen to biologically usable ammonia. The enzyme is composed of two proteins, the MoFe protein containing the active site, and the Fe protein. While the biochemical mechanism involving association and dissociation of these proteins is quite well understood, there are many uncertainties about the catalytic coordination chemistry which enables the enzyme to reduce unreactive N_2_ under remarkably mild conditions that are not yet replicated in synthetic or industrial conversions of N_2_. See references^[1]^ for reviews. The active site of Mo‐nitrogenase is an unprecedented cluster with the composition Fe_7_MoS_9_C(homocitrate), called FeMo‐co (Figure 1a).

The chemical mechanism, that is the catalysed conversion of N_2_ plus protons and electrons to NH_3_, is unknown, despite numerous investigations involving reaction kinetics,^[3]^ mutations of amino acids surrounding the active site,^[4]^ vibrational and spin resonance spectroscopy,[^1k^, ^5^] crystal structures,[^1l^, ^2^] and density functional simulations.^[6]^ Attempts to trap intermediates under turnover conditions are thwarted because protons are an unavoidable substrate (forming H_2_), and mixtures of transient intermediates occur.^[7]^ The stoichiometry of the reaction is eq 1, and a counting of the required individual steps – binding of N_2_, eight introductions of an electron, eight introductions of protons, breaking the N−N bond, six N−H bond formations, two dissociations of NH_3_, and one formation of H_2_ – yields 27!

(1) $$N{}_{2}+8H++8e{}^{-}+16\mathrm{ATP}\to 2\mathrm{NH}{}_{3}+H{}_{2}+16\mathrm{ADP}+16P$$

A significant aspect of the mechanism is the involvement of H_2_ and the relationship between N_2_ and H_2_. H_2_ is a competitive inhibitor of N_2_ reduction, and, conversely, the amount of H_2_ generated during turnover is controlled by the N_2_ partial pressure. This relationship is embodied in the N_2_/H_2_ exchange equilibria of the mechanistic framework presented by Thorneley and Lowe.^[3]^ In 1960 it was discovered that soybean root nodules containing nitrogenase generated HD when exposed to a D_2_/N_2_ atmosphere.^[8]^ This reaction, known as the HD formation reaction, was subsequently investigated further because it reveals some of the reactivity of the enzyme involving N_2_ and H_2_. The resulting experimental information is summarised in the next section.

## Experimental information relating to the HD formation reaction

1. The HD reaction requires N_2_. N_2_‐independent HD formation is <1 %.^[9]^
2. Other non‐physiological substrates that are reducible by nitrogenase do not activate the HD reaction: these include nitrous oxide,^[8]^ azide, acetylene, cyanide, methylisonitrile,^[10]^ and hydrazine.^[11]^
3. HD formation is inhibited by CO.[^9b^, ^10^, ^11b^, ^12^] The D_2_→HD and N_2_→NH_3_ pathways are inhibited equally by CO.^[9b]^
4. The HD reaction is inhibited by larger pN_2_.[^9b^, ^13^]
5. The HD reaction stoichiometry is D_2_+2H^+^+2e^−^→2HD.[^9b^, ^11b^]
6. The HD reaction and N_2_ conversion to NH_3_ compete for the same electrons. Electrons used for the HD reaction are diverted exclusively from the N_2_ reduction reaction.^[9b]^
7. There is negligible formation of TOH when T_2_ is used instead of D_2_.^[11b]^
8. Nitrogenase turning over with N_2_ and HD does not form D_2_.[^3^, ^14^]
9. The K_m_(N_2_) for HD formation and the K_m_(N_2_) for NH_3_ production are different. Turner and Bergersen, using partially purified bacteroid extracts, found the K_m_(N_2_) for NH_3_ formation was much larger than for HD formation,^[12]^ while Guth and Burris using purified *Klebsiella pneumoniae* measured K_m_(N_2_)=12.3±1.5 kPa for NH_3_ production and 18.9±4.3 kPa for HD formation.^[9b]^ Burgess et al. claimed no difference between the K_m_ values.^[11b]^ The Guth and Burris result from purified protein is apparently more reliable and is adopted here.
10. The protein modified as 195His→195Gln^[15]^ converts D_2_ to HD and binds N_2_ (evinced by N_2_ inhibition of H_2_ evolution and C_2_H_2_ reduction) but has very low conversion of N_2_ to NH_3_.[^4c^, ^4d^]
11. The protein modified as 195His→195Asn does not convert D_2_ to HD or convert N_2_ to NH_3_, but does bind N_2_.^[4d]^
12. Nitrogenase turning over with C_2_H_2_+N_2_+D_2_ forms C_2_H_3_D with minor amounts of C_2_H_2_D_2_.^[16]^

I here propose and computationally simulate a mechanism that accounts for the experimental characteristics of the HD reaction.

### Background, definitions and working hypotheses

Accumulated evidence, mainly from mutant‐reactivity experiments[^4b^, ^4c^, ^4d^, ^4l^, ^17^] indicates that the reaction domain is the 4Fe−4S face containing Fe2, Fe3, Fe6 and Fe7, and more specifically the Fe2−S2B−Fe6 region, because variations of Val70 and His195 have the most significant effects on reactivity. Assumptions that this domain remains intact during the catalytic cycle have been challenged in recent years, by crystal structures and reactivities of nitrogenase derivatives in which S2B (or other belt atom S3A, S5A) has been displaced into the protein by another atom or ligand,^[18]^ and a cryo‐EM structure obtained during turnover.^[19]^ The possible lability of S2B has attracted computational investigations.[^6h^, ^20^] An alternative hemilability of S2B, in which only one of the S2B−Fe bonds is broken during catalysis, has been explored computationally.[^6b^, ^6e^, ^20d^, ^21^] The mechanism presented here assumes that the bonds from S2B to Fe2 and Fe6 remain intact. Possible coordination positions at Fe2 and Fe6 are defined in Figure 1(b), and the intermediates to be described in the following have ligands variously at the *exo*‐Fe2, *endo*‐Fe2, *endo*‐Fe6 and *exo*‐Fe locations.

**Figure 2:**
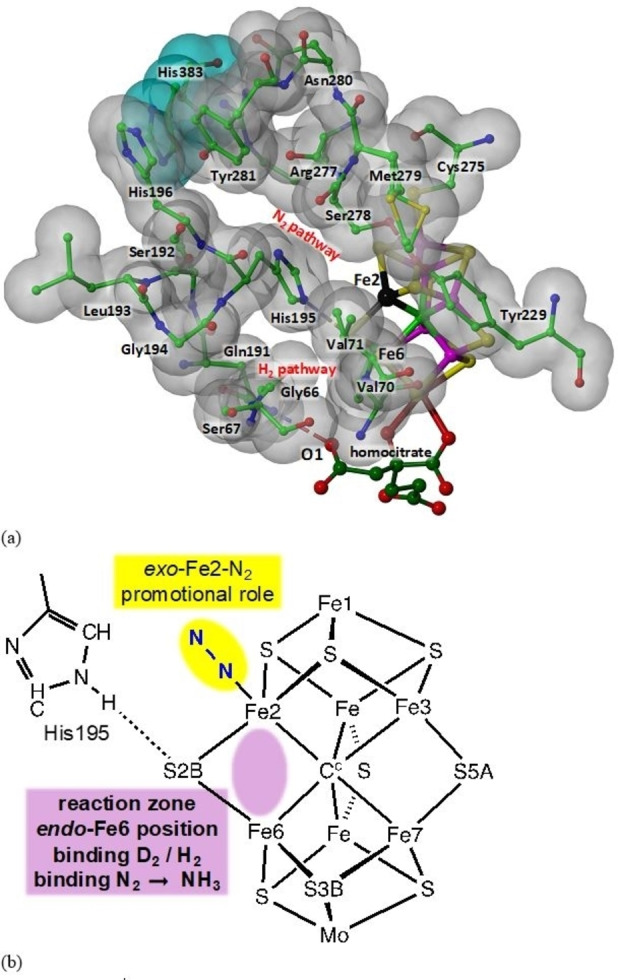
(a) The pathways proposed for N_2_ and H_2_ accessing the active site. His383 (blue surface) is at the channel mouth on the surface of the protein. (b) A working hypothesis, in which the role of *exo*‐Fe2−N_2_ (yellow) is to expand the reaction zone (violet) between Fe2 and Fe6, thereby promoting other reactions including binding of the N_2_ that is subsequently hydrogenated to NH_3_, in competition with the binding of H_2_ at the same site.^[25]^

Several working hypotheses underly the development of mechanism. One is that the H atoms required for the HD reaction and for N_2_ hydrogenation to NH_3_ arrive at S3B as the result of serial translocation of exogenous protons along the ‘proton wire’, and combination with electrons from the P‐cluster.^[22]^ These H atoms migrate vectorially from S3B to atoms Fe6, S2B and Fe2 of FeMo‐co.[^22c^, ^23^] The reducing reactions of nitrogenase involve transfer of these H atoms to substrates.^[24]^ Another working hypothesis is that N_2_ diffuses to FeMo‐co along a well‐defined hydrophobic pathway (Figure 2a) to the *exo* coordination position of Fe2, where it binds end‐on. This N_2_ is proposed to have an unreactive but promotional function.^[25]^ It is outside the reaction zone between Fe2 and Fe6 and less accessible to hydrogenation, but it does expand the reaction zone by lengthening the *trans* Fe2−C^c^ bond and increasing the Fe2−Fe6 separation by varying amounts up to 3.3 Å (from 2.6 Å in the resting state). The *endo*‐Fe6 position is proposed as the binding site for the N_2_ that will be subsequently hydrogenated to NH_3_, and the binding site for exogenous H_2_ that competes with N_2_ (Figure 2b). Therefore, the N_2_‐dependent D_2_→HD reaction is proposed to use promotional N_2_ at *exo*‐Fe2, and D_2_ binding at *endo*‐Fe6. Figure 2 (a) also shows the location of the separate H_2_ pathway, for ingress of D_2_ and egress of HD. See Figure S1 for details of the H_2_ pathway.

### Computational model and procedures

The density functional computed protein model is a 486‐atom extract from crystal 3U7Q, including all relevant amino acids. This is my standard model for simulations of nitrogenase reactions and reactivity.[^6d^, ^6f^] Details, and the rationale for inclusion of amino acids and truncation of uninvolved sidechains, are provided in the Supporting Information. Some constraints on the protein structure are required during optimization calculations because the modelled protein is incomplete and the bonding and dispersion influences of the complete protein outside the computational model are absent. The modelled protein must also maintain flexibility sufficient to accommodate the coordination of N_2_ and of H_2_, and the diffusion of these molecules to and from FeMo‐co. The constraining strategy and details are provided in the Supporting Information. The charge on the [CFe_7_MoS_9_] core of FeMo‐co is −1, in agreement with experimental and computational studies.[^6d^, ^26^]

Density functional (DF) calculations use the DMol methodology of Delley,^[27]^ with accurate DNP (double numerical plus polarisation) basis sets.^[27d]^ The gradient‐corrected functional PBE^[28]^ was used because validation tests demonstrate that when used with DMol it is more accurate than other commonly used functionals.^[29]^ The calculations were all‐electron, spin‐unrestricted, with no imposed symmetry. D was calculated as H and no zero‐point or thermal corrections are included. The conductor‐like screening model (COSMO)^[30]^ was used with a dielectric constant of 5. Constraints on interatomic distances used the Lagrange Multiplier Algorithm. Output spin populations are calculated by the Mulliken method.^[31]^ Further information on the density functional procedures is provided in the Supporting Information.

**Figure 3:**
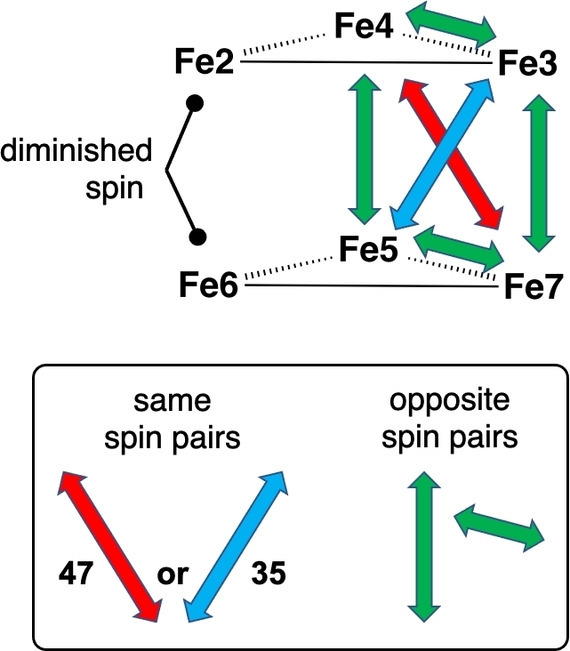
When the spin populations of Fe2 and Fe6 are diminished due to ligation, there are two pairs of negative spin, red *or* blue, that yield four opposite (green) spin pairs along the shorter edges of the Fe3, Fe4, Fe5, Fe7 face where the spin populations have largest magnitude.

The electronic states of FeMo‐co and its ligated forms are described as sets of signed spin populations on the seven Fe atoms of the cluster, together with the net spin S. Labelling consists of a list of the Fe atom numbers having negative spin population, with S appended in parentheses. Relative stabilities of these electronic states are guided by the general principle that oppositely‐signed pairs of adjacent Fe atoms in FeMo‐co are more stable than same‐signed, and that the stabilisation is proportional to the magnitude of the spin populations. Antiferromagnetism (ie weak Fe−Fe bonding) operates in the FeMo‐co cluster. Interactions between the spins at Fe2 and Fe6 are of minor importance because they are diminished by ligation (often almost to zero: see values in the Supporting Information), and additionally the Fe2−Fe6 separation is increased due to extensions of one or both Fe−C^c^ distances. For the same reason interactions between the Fe2 and Fe6 spins and those at Fe3, Fe4, Fe5 and Fe7 are less influential than those between these four Fe atoms. Therefore, interactions within the set Fe3, Fe4, Fe5, Fe7 on the opposite face of the Fe_6_ trigonal prism are dominant, and, as shown in Figure 3, the maximum number of oppositely‐signed shorter edges for this group is obtained with same‐signed spins along one diagonal, Fe4−Fe7 or Fe3−Fe5. Consequently only two electronic states are expected to be more stable than all others.^[25]^ These are the states with the Fe3−Fe5 pair negative (labelled 35), or the Fe4−Fe7 pair negative (labelled 47), and these are the states investigated in the present work.

The spin population specifications input into the DMol calculation involve only Fe1, Fe3, Fe4, Fe5 and Fe7, using the sign patterns of the 47 or 35 states, with magnitudes 3.0 for Fe1 and ca. 2.8 for the other four. The spin populations of Fe2 and Fe6 are unspecified. The spin populations on all atoms are allowed to optimize. The total spin S is initially optimised (using Fermi orbital population) and then variations of +1 and −1 are explored.

The procedure for mapping the topology of reaction energy surfaces and for location of transition states, previously described,[^6d^, ^32^] is outlined in the Supporting Information. Trajectories from the transition state to the reactant and product states were followed to determine energy differences and ensure the continuity of the potential energy surface. For steps involving association of D_2_ or dissociation of HD, the separated limit of the trajectory was taken as the place where the dissociated molecule and the metal site cease their mutual influence (assessed via geometry) and the free molecule begins to tumble and diffuse freely: this Fe‐(D/H)_2_ separation is 3.2±0.2 Å. Energies calculated from these separated positions are reported at lower precision, reflecting uncertainties in defining dissociated limits.

Atomic coordinates for all intermediates and transition states, together with spin populations at the Fe atoms, are provided in the Supporting Information.

## Results

**Scheme 1:**
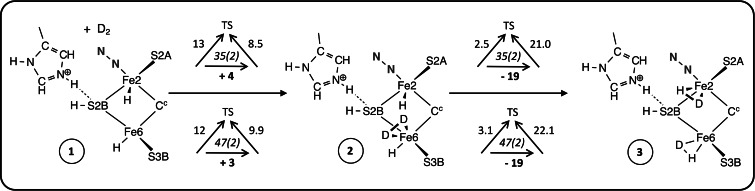
First stages of the mechanism, showing the binding of D_2_ (**2**) and the synchronous double hydrogenation of D_2_ forming two bound HD (**3**). The sketches show only the essential components. The triangular symbols report electronic(S) states and calculated energies (kcal mol^−1^): the reaction energy is on the horizontal arrow, and barrier energies are oblique to the transition state (TS).

**Scheme 2:**
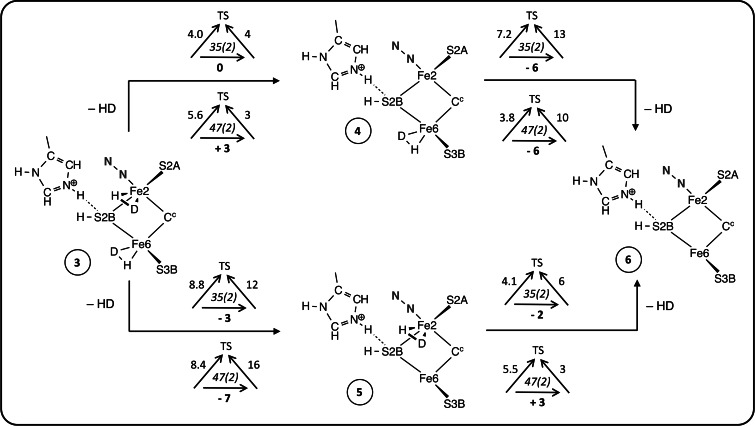
Two routes for the sequential dissociation of two Fe‐bound HD.

**Scheme 3:**
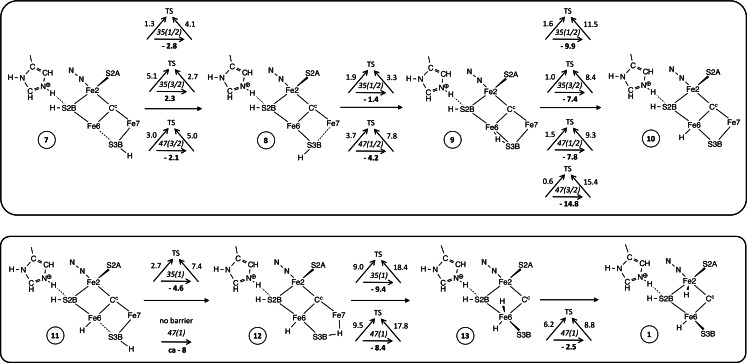
Reaction sequences for the introduction of the H atom at *exo*‐Fe6 (upper panel) and subsequently introduction of the second H atom and its movement to the *endo*‐Fe2 position (lower panel). Both H atoms begin at S3B in the 3b5 conformation. The sketches show only essential components, and the triangular symbols report electronic(S) states and calculated energies (kcal mol^−1^): the reaction energy is on the horizontal arrow, and barrier energies are oblique to the transition state (TS).

There are four stages in the mechanism, first binding of D_2_, then synchronous double hydrogenation of D_2_ to form two bound DH, then dissociation of the two HD, and finally recovery of the starting intermediate. Details are presented in Schemes 1, 2 and 3. For each step the calculated reaction dynamics are presented with triangular symbols, each of which records at its centre the electronic and spin state, with the overall reaction energy under the horizontal reaction arrow, and the barriers for the forward and backward reactions against the diagonal arrows to the transition state (TS): all energies are in kcal mol^−1^. For each intermediate the En level in the Thorneley‐Lowe notation,^[3]^ is evident from the number of added H atoms.

### Association of D_2_ and formation of 2HD

Referring to Scheme 1, the starting structure **1** contains promotional N_2_ bound at *exo*‐Fe2, and H atoms bound at S2B, *exo*‐Fe6 and *endo* Fe2. His195 is in its protonated state, with a donor NϵH hydrogen bond to S2B. Structure **1** is at the E3 level.^[3]^ The Fe6−Fe2 separation in **1** is 2.9–3.2 Å (depending on electronic state), and the ligand binding position, *endo*‐Fe6, is unobstructed. D_2_ binds at *endo*‐Fe6, forming **2**. The optimised total spin for **1** and **2** is S=2. The reaction potential energies (horizontal arrows in Scheme 1) for binding of D_2_ are 3 or 4 kcal mol^−1^, and reaction barriers are 12, 13 kcal mol^−1^. These numbers are combinations of the negative contribution from the formation of the Fe‐D_2_ bonds and the positive adaption energy required to expand the *endo* angle at Fe6 as D_2_ binds.^[6f]^

**Figure 4:**
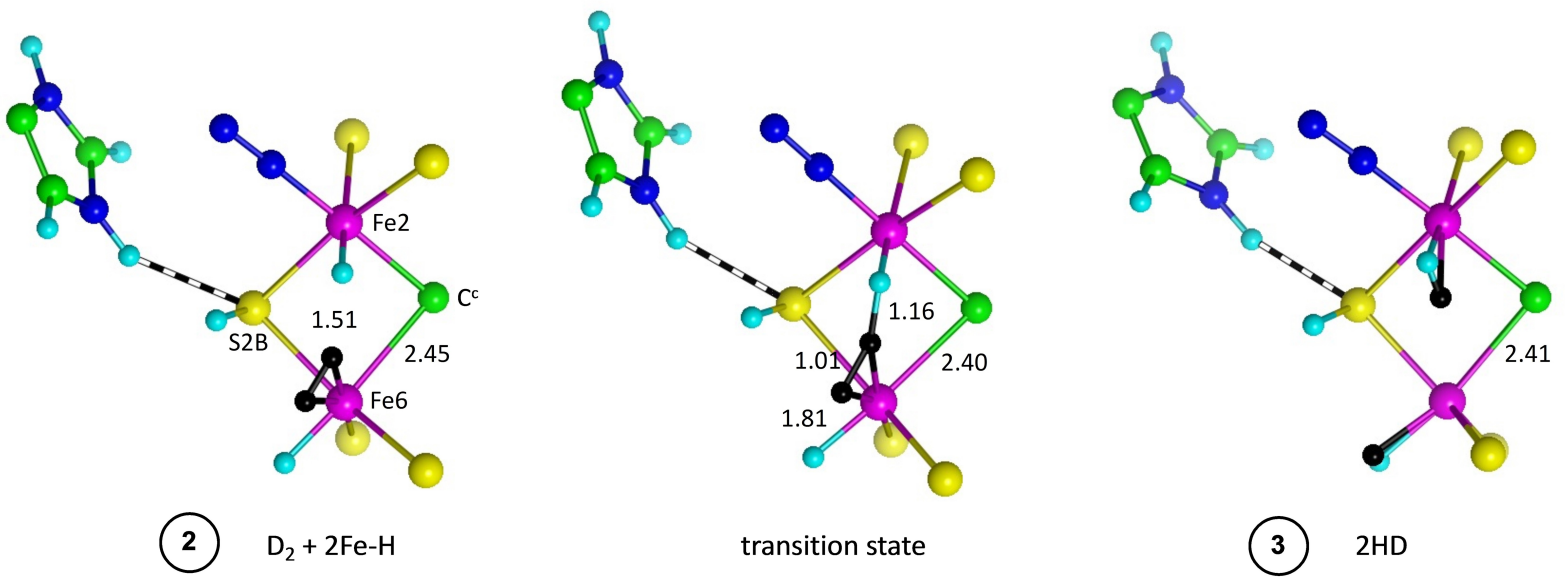
Geometric details of the synchronous conversion of D_2_ to 2HD. D atoms black, distances in Å.

Structure **2** is propitious because there are H atoms contiguous to D_2_ on Fe2 and Fe6. A single transition state occurs for synchronous transfer of these two H atoms to D_2_, which splits to form two HD, one at *endo*‐Fe2 and the other at *exo*‐Fe6. This is the remarkable key step in the overall reaction. Figure 4 shows detail of the reactant, TS, and product structures. The reaction dynamics are very favourable, with reaction energies of −19 kcal mol^−1^ and reaction barriers 2.5 to 3.1 kcal mol^−1^.

### Dissociation of 2HD

The next stage of the mechanism is sequential dissociation of the two Fe‐bound HD. There are two possible sequences, dissociation first from Fe2 (**3**→**4**→**6**) or first from Fe6 (**3**→**5**→**6**). Reaction energies are reported in Scheme 2 for the 35(2) and 47(2) electronic states. All the reaction energies and kinetic barriers are feasible for both pathways.

### Completion of the catalytic cycle

**Figure 5:**
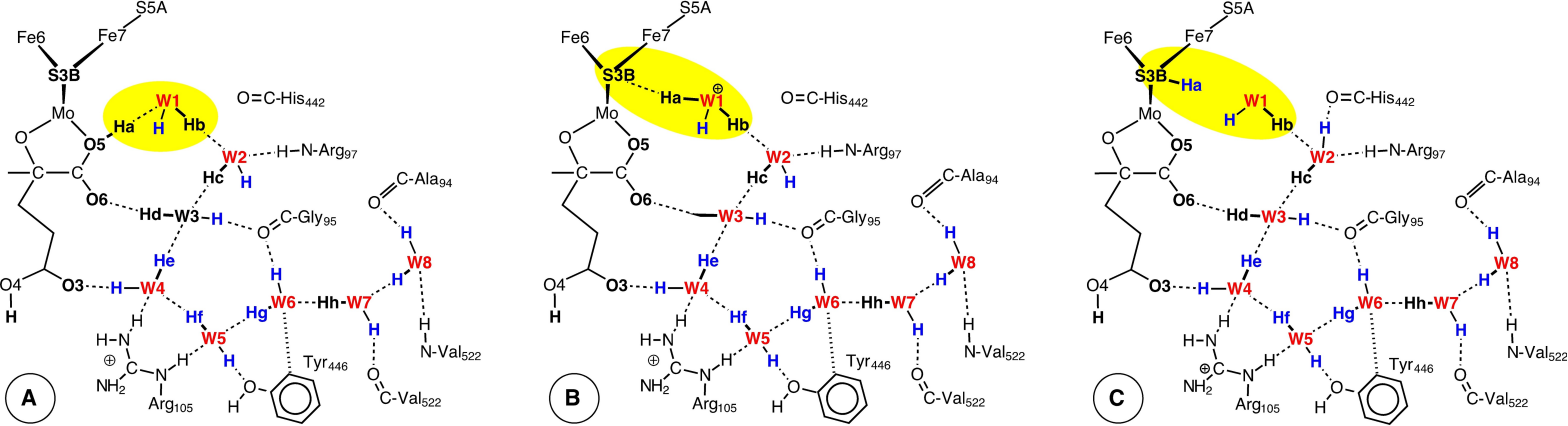
The last three stages in the mechanism for proton translocation along the water chain (red) and protonation of S3B (**C**). Extracted from Ref. [32c].

**Figure 6:**
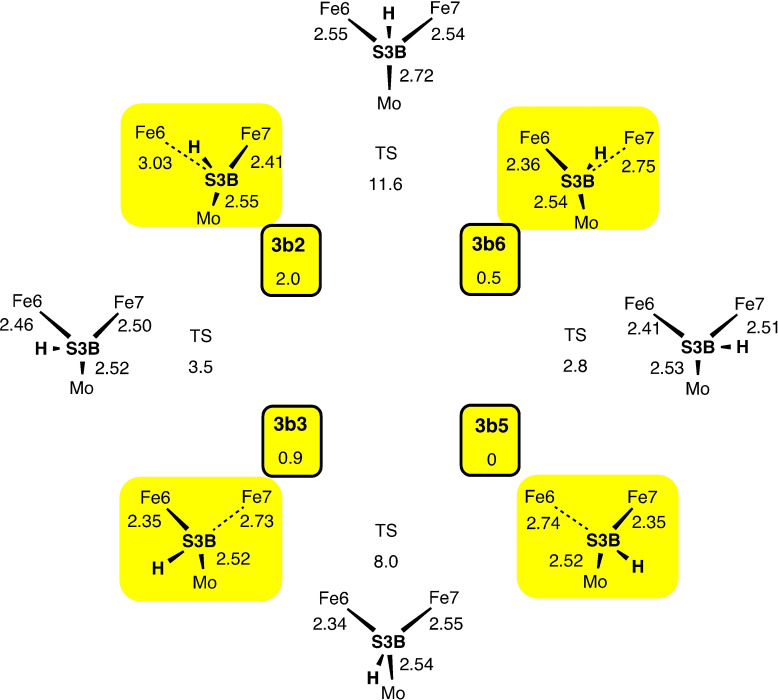
The conformations of S3B−H and their labels (modified from Ref [32c]). The four yellow highlighted structures are energy minima with pyramidal stereochemistry at S3B, and the other four structures are the transition states between them. Distances (Å) and energies (kcal mol^−1^) are those calculated for *unligated* FeMo‐co.^[32c]^

After completion of the conversion of D_2_ to 2HD, the subsequent recovery to the starting point (**1**) of the catalytic cycle needs to be considered. This requires the introduction and placement of two H atoms. As previously described, protons are delivered to FeMo‐co via the ‘proton wire’ from the protein surface, in conjunction with electrons delivered to FeMo‐co via the P‐cluster. A detailed Grotthus mechanism for this delivery of protons to S3B has been calculated, and Figure 5 shows the final intermediates in this multistep process for protonation of S3B.^[32c]^ The starting point for the placement of each H atom in the recovery phase of the HD mechanism is therefore an H atom bonded to S3B. The next steps involve conformational changes for S3B−H, followed by migration of each H to Fe6 or Fe2. The conformations of S3B−H, are illustrated in Figure 6, with labels and transition states for interconversions, together with geometry and energy data calculated for *unligated* FeMo‐co.^[32c]^ When H is first generated on S3B from the water chain (**A** to **C** in Figure 5) S3B−H is in the 3b5 conformation. Details of the dynamics for the reconfiguration of S3B−H depend on the ligation status of FeMo‐co and vary from those of unligated FeMo‐co (Figure 6). The reconfiguration dynamics specific to the formation of **1** are calculated and reported here.

The first H added to S3B is in the 3b5 conformation, intermediate **7** in Scheme 3. From this there is an energetically favourable pathway to **8** (3b3 conformation) and then to **9** as detailed in Scheme 3. From the S3B−H−Fe6 bridge in **9** the H atom can move towards the *exo* or *endo* positions of Fe6. The favourable pathway is to *exo*‐Fe6−H, **10**, with energy changes of −7.4 to −14.8 kcal mol^−1^ and remarkably small kinetic barriers ≤1.6 kcal mol^−1^. The alternative route from **9** towards *endo*‐Fe6 has larger kinetic barriers and leads to an Fe6−H−Fe2 bridge. Concurrently with this bridge formation, S2BH unhooks its bond to Fe2 and eventually is more than 3.2 Å from Fe2. Unhooking of S2B from Fe2 is a general property of ligated FeMo‐co, and the factors that influence it have been expounded, and the potential energy surfaces for interconversions of hooked and unhooked isomers described.^[21e]^ In general the energy differences between hooked and unhooked isomers are <10 kcal mol^−1^, and kinetic barriers for interconversions are up to 13 kcal mol^−1^. When S2B unhooks from Fe2 the tethering Fe6‐S2B bond sterically blocks subsequent formation of *exo*‐Fe6−H, and re‐formation of **1** becomes very unfavourable because the barrier to rehooking is large. However, this negative co‐influence of *exo*‐Fe6−H and unhooking of S2BH is advantageous when *exo*‐Fe6−H is formed first, as in intermediate **10**, because *exo*‐Fe6−H inhibits unhooking of S2BH (by steric interference), and therefore the subsequent introduction of a second H atom and its movement from S3B around Fe6 to *endo*‐Fe2−H is unimpeded by unhooking. Therefore, in the re‐formation of **1** the first H moves from S3B to the *exo*‐Fe6 position, and the second H moves to the *endo*‐Fe position (Scheme 3).

In the sequence that generates **10** from **7** all three steps are exergonic, and the barriers are remarkably small, being 1.3, 1.9 and 1.6 kcal mol^−1^ respectively in the 35(1/2) electronic state, and maximum 5.1 kcal mol^−1^ in other electronic states. The second H joins S3B in conformation 3b5, **11**, then moves around S3B in the opposite direction to the first, forming **12** where it bridges to Fe7. In the 47(1) electronic state there is no barrier to this exergonic shift. The next movement is around Fe6 to the *endo*‐Fe6 position, **13**. The final step for the second H is to the *endo*‐Fe2 position, recovering structure **1**. This last step occurs favourably for the 47(1) electronic state, but in the other electronic states tested the final energy well is an Fe6−H−Fe2 bridge rather than *endo*‐Fe2−H. The key factor in this **13**→**1** step is the distance between Fe2 and Fe6, which is ca. 3.3 Å in **13** and ca. 3.1 Å in **1**. To transfer H from Fe6 to Fe2 requires a closing of the gap, and the Fe6−Fe2 separation decreases to 2.8 Å in the TS for the 47(1) state. This is close to the separation in the Fe6−H−Fe2 bridge, ca. 2.65 Å, which is why this bridge becomes an accessible local energy well in some electronic states.

### Full mechanism

The complete mechanism has 10 steps, with bifurcation from **3** to **6**. The first two steps are the critical components of the mechanism, creating two HD from exogenous D_2_. The large exergonicity of the splitting step, **2**→**3** (−16 to −19 kcal mol^−1^), exceeds the endergonicity of the binding step (+3 or +4 kcal mol^−1^), and the barriers for **2**→**3** are small (2.5 to 4.2 kcal mol^−1^). All subsequent steps in the sequence have exergonic options with surmountable reaction barriers. The first step, D_2_ binding, would be rate‐determining for the complete mechanism. Although the differences between the calculated dynamics for each electronic(spin) state at each step are marginal, the energies indicate the following electronic surfaces: for HD formation and dissociation **1**→47(2) **2**→47(2) **3**→35(2) **5**→35(2) **6**, and for the recovery phase **7**→35(1/2) **8**→35(1/2) **9**→35(1/2) **10**, **11**→35(1) **12**→35(1) **13**→47(1) **1**.

### Testing against experiment

I now return to the experimental information against which the proposed mechanism must be tested.

1. **The HD reaction requires N**_2_. The mechanism involves ‘promotional’ N_2_ bound end‐on at *exo*‐Fe2. Would the mechanism be feasible if this N_2_ is absent, or replaced by H? Additional calculations show that when there is no *exo*‐Fe2 ligand the Fe2−C^c^ distance is short, ca. 1.95 Å, because it is compensating, via coordinative allosterism,^[33]^ for the ligation of Fe6 (*exo*‐Fe6−H and/or *endo*‐Fe6−H_2_) which extends Fe6−C^c^. This results in an unsuitable small reaction domain between Fe2 and Fe6. Also, if the *exo*‐Fe2 position is vacant the H at *endo*‐Fe2 (essential for the proposed mechanism) rotates to the more stable *exo*‐Fe2 position. If H is bonded at *exo*‐Fe2 instead of N_2_, then as D_2_ binds at the *endo*‐Fe6 position in the first step (analogous to **1**→**2**) the H atom at *endo*‐Fe2 moves away from D_2_ and towards the *exo* position of Fe2. The H−Fe2−C^c^ angle increases to 108°, compared with 90–94° in **2**. From this position the two H atoms on Fe2 can combine to form H_2_ at the *exo* position of Fe2 with a very small barrier of 2 kcal mol^−1^. This makes the double hydrogenation of D_2_ impossible. Figure 7
  **Figure 7:**
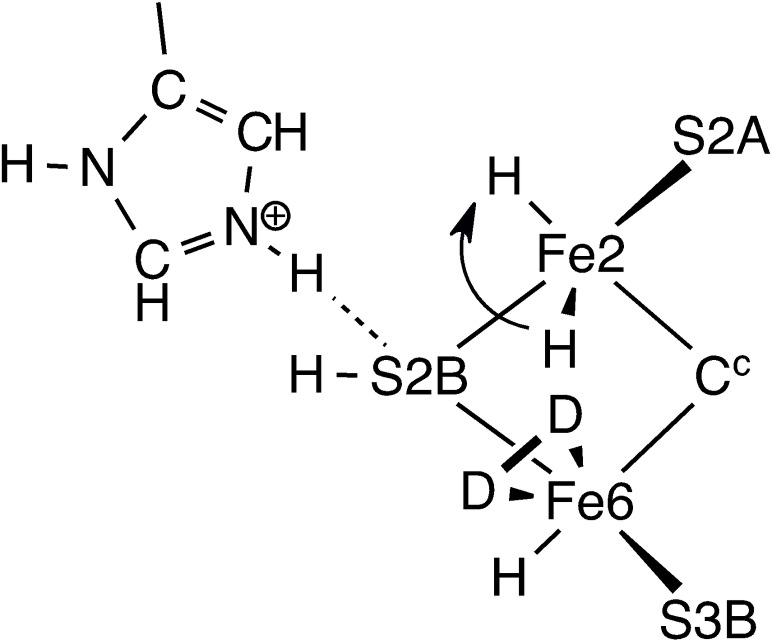
Movement of *endo*‐Fe2−H when the *exo*‐Fe2 position contains H or is void.
  illustrates the adverse movement of *endo*‐Fe2−H towards the *exo*‐Fe2 position when that position is void or contains H. Therefore, of the three possibilities for *exo*‐Fe2, void or occupied by N_2_ or H, only N_2_ permits the double hydrogenation of D_2_, in agreement with experiment.
2. **Other non‐physiological substrates do not activate the HD reaction**. The present mechanism has not been tested computationally with the experimental substrates nitrous oxide, azide, acetylene, cyanide, methylisonitrile or hydrazine.
3. **CO inhibits the HD reaction**. As part of the calculations reported above I made analogous calculations with CO bound at the *exo*‐Fe2 position in place of N_2_. The D_2_ splitting to 2HD step, analogous to **2**→**3**, is similarly feasible with *exo*‐Fe2−CO. It is well established from IR spectroscopy[^4n^, ^34^] and ENDOR,^[35]^ supported by calculations,^[36]^ that CO terminally bound to FeMo‐co readily converts to bridging CO. The CO analogue of **7** would be expected to convert to a Fe2−CO−Fe6 bridged form, which would block completion of the cycle and account for the CO inhibition. The fact that CO inhibits equally the D_2_→HD and N_2_→NH_3_ pathways is explained by its facile occupation of the reaction domain between Fe2 and Fe6, blocking *endo*‐Fe6. The proposed mechanism for the HD reaction is consistent with the inhibition by CO.
4. **The HD reaction is inhibited by larger pN_2_**. One of the working hypotheses is that the *endo*‐Fe6 position is the binding site for the N_2_ that subsequently will be hydrogenated to NH_3_. After the promotional N_2_ is bound at *exo*‐Fe2 a second N_2_ can bind end‐on at *endo*‐Fe6.^[25]^ Increased pN_2_ would shift the competition for the *endo*‐Fe6 location against D_2_ (H_2_) binding, and inhibit HD formation.
5. The HD reaction stoichiometry is D_**2**_+2H^**+**^+2e^−^→2HD. This is embodied in the proposed mechanism.
6. **The HD reaction and N**_2_ **conversion to NH**_3_ **compete for the same electrons**. In contemporary mechanistic concepts for nitrogenase the reducing agents are transferable H atoms on Fe and S atoms of FeMo‐co.^[6d]^ The HD reaction and NH_3_ formation are proposed to bind the substrate at the same e*n*do‐Fe6 site and to use surrounding H atoms, in competition.
7. **There is negligible formation of TOH when T_2_ is used instead of D_2_**. This means that intermediates in the HD reaction cannot access water or protons able to reach the protein surface. In the proposed mechanism the only D‐containing intermediates are reactant D_2_ or product HD, both bound to Fe within hydrophobic surrounds.
8. **Nitrogenase turning over with N**_2_ **and HD does not form D**_2_. The proposed mechanism is not reversible because the D_2_→2HD step, **2**→**3**, is very exergonic, with barriers of 20 to 23 kcal mol^−1^ for the reverse. A reverse process **2**←**3** which could possibly generate D_2_ from 2HD, is not feasible. In the context of this experimental requirement, it is appropriate to consider an alternative but related mechanism, shown in Scheme 4
  **Scheme 4:**
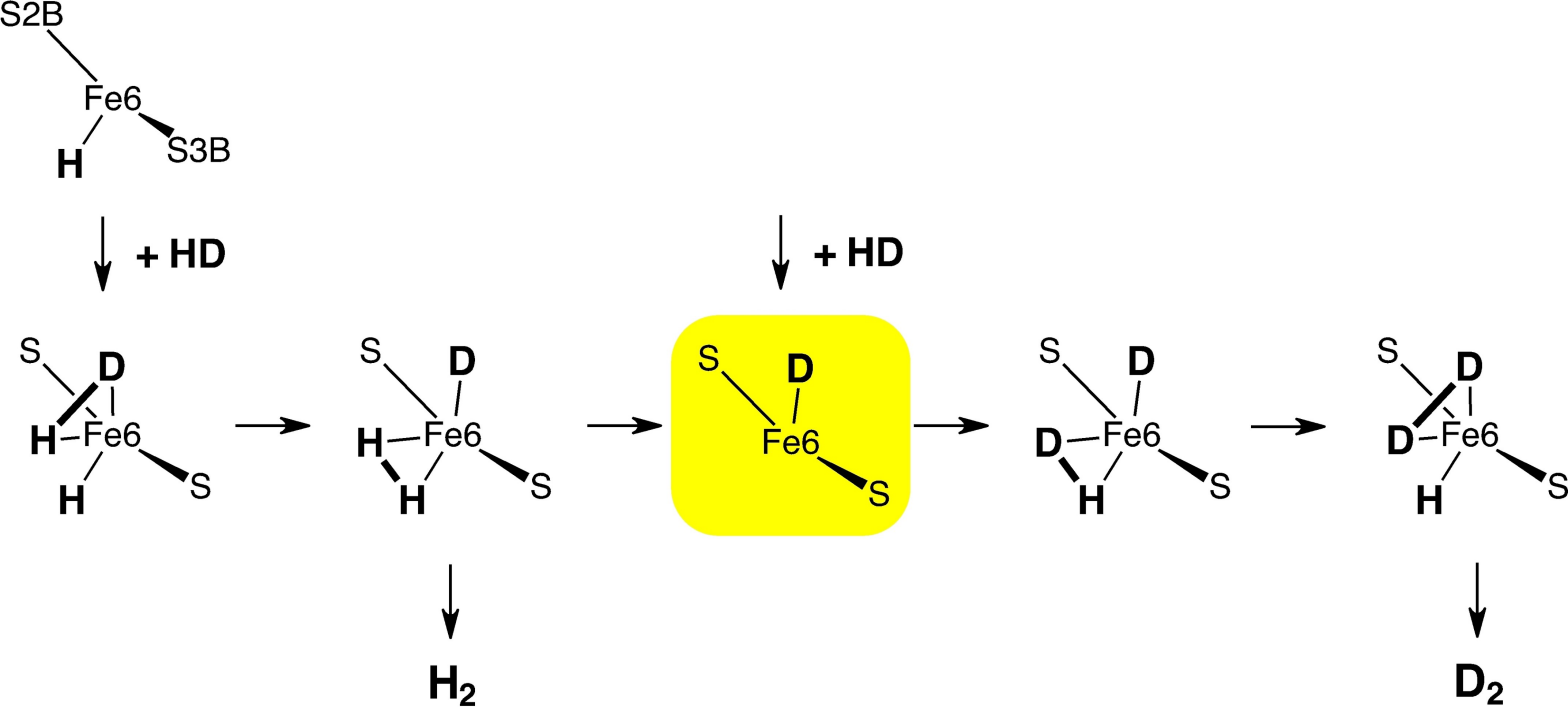
An alternative mechanism that allows the reaction 2HD→D_2_+H_2_. This cannot occur with the proposed mechanism because the essential intermediate, yellow highlight, is absent.
  , that could effect the exchange 2HD→H_2_+D_2_. Here HD is added to the vacant *endo*‐Fe6 site, followed by H⋅⋅H⋅⋅D exchange on Fe6, then dissociation of H_2_ to form the central intermediate (yellow highlight) with a D atom on Fe6. Repetition with a second HD yields D_2_, and completes the exchange. Here the exchange of H and HD both bonded to the same Fe has some similarities with part of the **2**→**3** step in the proposed mechanism. However, there is an important difference. The proposed mechanism has synchronous double hydrogenation of D_2_ in **2**→**3**, but the mechanism in Scheme 4 must have *non*‐synchronous steps. Another statement of this difference is that the highlighted intermediate on Scheme 4 that is essential for the 2HD→H_2_+D_2_ reaction does not occur in the proposed mechanism. In this way only the proposed mechanism is consistent with the experimental observation.
9. **The K_m_(N_2_) for HD formation and the K_m_(N_2_) for NH_3_ production are apparently different**. The two different processes are postulated to involve different N_2_ binding steps at different Fe sites, and so different binding constants are possible, and expected.
10. and 11. **Different reactivities for the 195Gln and 195Asn mutated proteins**. The present results provide no insight into why 195Gln supports the HD reaction but 195Asn does not. The 195Gln side chain can form a distorted hydrogen bond from NH_2_ to S2B^[15]^ but the 195Asn side chain cannot hydrogen bond to S2B. Perhaps a hydrogen bond from the 195 side chain to S2B influences the formation of **1**. Calculations modelling the roles of Gln and Asn side chains are in progress.
11. **Formation of C_2_H_3_D in the presence of C_2_H_2_**. I speculate that intermediate **5** could bind C_2_H_2_ at Fe6 and could then add the nearby HD on Fe2: no computational results are yet available.

## Discussion

The strong component of this mechanism is the energetically advantageous double hydrogenation of D_2_ in one step, **2**→**3**. The reaction energy of −19 kcal mol^−1^ and small barrier of 3 kcal mol^−1^ are sufficient to overcome the endergonic prior binding of D_2_. The entropy penalty for the binding of D_2_ needs to be included. The relevant dissociated state is not gas phase but condensed phase, with D_2_ considered to be at a diffusible position within the protein. Here the ligand will have lost translational and rotational entropy relative to the gas phase. The relevant entropy loss on binding is not known or calculated, but can be estimated through analogy with other relevant systems. Seefeldt, Peters et al. described the structure and dynamics of a modified MoFe protein containing an acetylene molecule in a pocket near the front face of FeMo‐co but not bound to it, and suggested that acetylene in the protein channel leading to its pocket has already lost most of its gas phase entropy content.^[17d]^ Using experimental data for H_2_ binding to Fe complexes, and compendia of information of the entropy of small molecules in proteins,^[37]^ I estimated the entropy change for the binding of H_2_ to FeMo‐co as approximately −6 cal mol^−1^K^−1^.^[6f]^ This corresponds to a free energy penalty of 2 kcal mol^−1^. ΔG for **1**→**2** is thereby estimated to be not greater than 6 kcal mol^−1^.

An advantage of the double hydrogenation of D_2_ in one step is avoidance of the steps in Scheme 4. Separate calculations were made using a start structure without *endo*‐Fe2−H, and therefore potentially able to effect single hydrogenation of D_2_ but unable to achieve synchronous double hydrogenation. The binding energy for D_2_ at *endo*‐Fe6 is 4 kcal mol^−1^, and the hydrogenation energy for H+D_2_→HD+D at Fe6 is −9 kcal mol^−1^. This shows that the binding and H transfer steps in Scheme 4 are feasible: they need to be avoided to satisfy one of the experimental constraints, and the synchronous double hydrogenation mechanism satisfies this requirement. Note that the reaction energy for this single hydrogenation (−9 kcal mol^−1^) is half the double hydrogenation energy of **2**→**3** (−19 kcal mol^−1^). An alternative reaction sequence in which incoming D_2_ binds at an empty *exo*‐Fe6−H position is discounted because previous calculations showed that this binding is very endergonic.^[6f]^

The follow‐up dissociations of the HD are straightforward and will benefit from the small entropic component. Completion of the cycle requires introduction of two H atoms, initially as protons at S3B coupled with electronation. These H atoms move without difficulty around S3B, first to Fe6 at the *exo* position and secondly towards Fe2. The least secure part of the mechanism is the last stage and the regeneration of *endo*‐Fe2−H in **1**: the complication is the nearby energy well of the H bridge between Fe6 and Fe2. An alternative pathway to *endo*‐Fe2−H could be via S2B, and another possibility is the unhooking of S2BH from Fe2, opening the Fe2−Fe6 space. These possibilities are being simulated. *Endo*‐Fe2−H in **1** is significant because if this position is void D_2_ could bind there with favourable dynamics.^[6f]^ Further work involves variation of the state of the His195 side chain (ie NϵH or NδH, rather than NϵH plus NδH as in the present mechanism). An unresolved question is why the Asn195 mutant, incapable of hydrogen bonding with hooked S2B, stymies the HD reaction.

This paper presents the core of a mechanism for the HD reaction, consistent with most of the experimental requirements. The intermediates and reaction steps are closely connected with the intermediates and multiple reaction steps in the N_2_→NH_3_ reaction, involving introduction of H atoms, binding of N_2_ with exchange of H_2_, and H to N transfers. The results presented here inform continuing investigation of these steps, and integration of the HD and N_2_→NH_3_ reaction mechanisms.

## Conflict of interest

The author declares no conflict of interest.

1

## Supporting information

As a service to our authors and readers, this journal provides supporting information supplied by the authors. Such materials are peer reviewed and may be re‐organized for online delivery, but are not copy‐edited or typeset. Technical support issues arising from supporting information (other than missing files) should be addressed to the authors.

Supporting Information

## Data Availability

The data that support the findings of this study are available in the supplementary material of this article.
