# Supplementary material for: The HD Reaction of Nitrogenase: a Detailed Mechanism
Source: Chemistry. 2022 Nov 29;29(4):e202202502. doi: 10.1002/chem.202202502 (PMC10099629; doi:10.1002/chem.202202502)
Supplement: Supplementary file 1 — Supporting Information [file CHEM-29-0-s001.pdf]

Chemistry–A European Journal

Supporting Information

The HD Reaction of Nitrogenase: a Detailed Mechanism

Ian Dance*

Supporting Information

Contents

1. H_2 pathway
2. Protein model
3. Constraints on the computed protein
4. Density functional procedures
5. Determination of transition states
6. References
7. Energies and spin populations for intermediates and transition states
8. Coordinates and spin populations for intermediates and transitions states

1. H_2 pathway

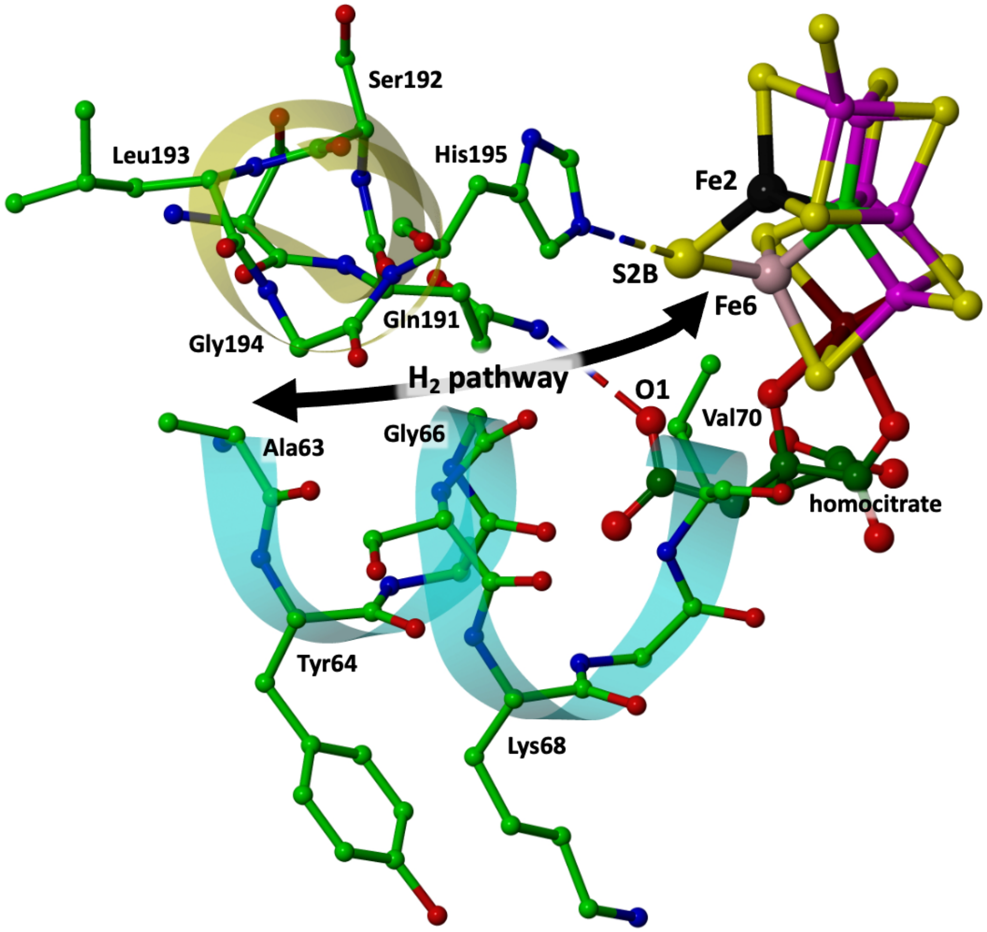

Fig. S1. Residues adjacent to the proximal H_2 pathway, which links to the region between Fe2 (black) and Fe6 (pink).

2. Protein model

The quantum computed protein model is an extract from crystal 3U7Q, shown in Chart S1. The six principal chains surrounding FeMo-co are highlighted with different colours, and the connecting hydrogen bonds are marked. Homocitrate, the His442 ligation of Mo, and relevant nearby water molecules are included (486 atoms total). Details, and the rationale for inclusion of amino acids and for truncation of uninvolved sidechains, are provided in the caption for Chart S1. Investigations of the protonation state of homocitrate through crystal structures at various pH (summarised in ref [11]), vibrational circular dichroism spectroscopy,[2] QM/MM calculations,[1, 3] and quantum refinement,[3] indicate that the coordinated alcoholate O atom is protonated. In the current model this atom is not protonated. This difference is not expected to have significant consequences for the reaction steps of the mechanism described here because there are no potential hydrogen bonds or H transfer possibilities.

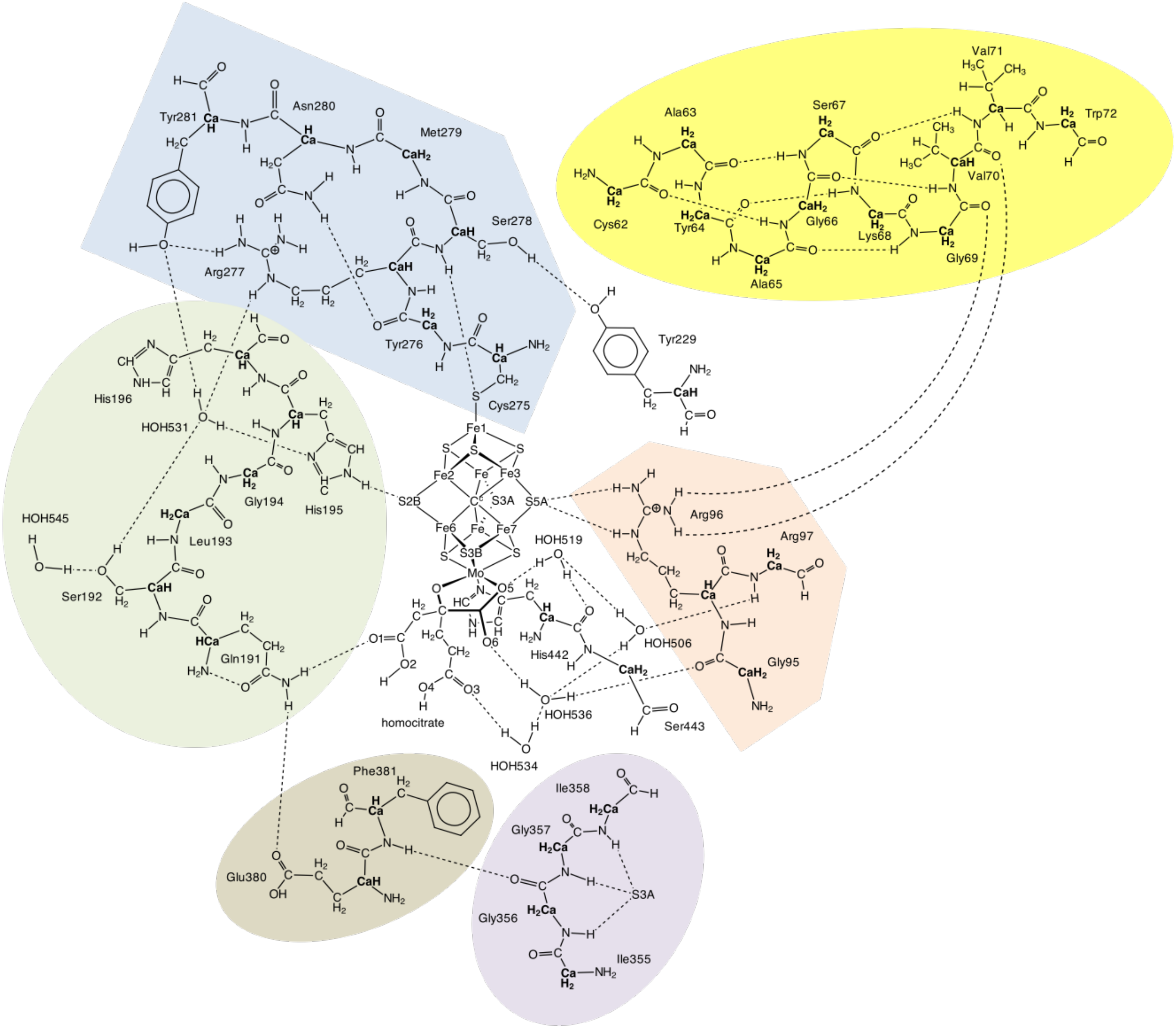

Chart S1. The quantum computed model, with residues and water molecules labelled according to *Azotobacter vinelandii* protein, crystal structure 3U7Q. Cα are labelled as Ca, and hydrogen bonds are broken lines. Non-influential sidechains are truncated to gly. In the ‘front’ chain (yellow highlight) that extends over the Fe2Fe3Fe6Fe7 face of FeMo-co the crucial residues Val70 and Val71 are complete, while 62, 63, 64, 65 67, 68 and 72 are modelled as Gly. In the 191 to 196 chain (highlight green) the significant sidechains of Gln191, Ser193, His195 and His196 are complete. In the 275-281 chain (blue highlight) Cys275, Arg277, Ser278, Asn280, Tyr281 near the N_2 pathway are complete: Tyr229 is included in full because it borders the reaction domain and has an important hydrogen bond with Ser278. In chain 95-97 (salmon highlight) the sidechain of Arg96 is included because it forms crucial hydrogen bonds with S5A, Gly69 and Val70: the sidechain of Arg97 is not nearby and is truncated to gly. In the rear chain (mauve highlight) with hydrogen bonds to S5A, Ile355 and Ile358 are truncated to gly. Glu380 and Phe381 (fawn highlight) are included in full. Ligand His442 is complete and Ser443 is truncated to gly. HOH531 has hydrogen bonds with two chains, Ser192, His195 and Arg277, Tyr281, and is part of the putative proton transfer from surface water (HOH545)

to His195.[4] The four penultimate waters of the proton wire[5] (HOH519, HOH506, HOH536 and HOH534) are included.

Experimental and computational studies are in general agreement that the redox level of the [CFe_7MoS_9] core of resting FeMo-co cluster is described by net charge -1,[1, 6] and so the net charge for the complete model in Chart S1is -2 (-1 when the His195 side chain is protonated).

3. Constraints on the computed protein

Some constraints on the protein structure are required during optimization calculations because the modelled protein is incomplete and the bonding and dispersion influences of the complete protein outside the computational model are absent. The modelled protein must also maintain flexibility sufficient to accommodate the coordination of N_2 and of H_2, and the diffusion of these molecules to and from FeMo-co. The constraints used here involved the Cα atoms at the ends of the modelled protein chains, allowing movement of the intermediate residues and all sidechains. The constraints maintained the crystallographic distances between C^c at the centre of FeMo-co and the Cα atoms of residues 62, 95, 97, 191, 196, 229, 281, 355, 358, 380, 381, 443, and included C^c -- water distances. As previously explained[7] trial calculations on the modes of diffusion and coordination of N_2 / H_2 led to the conclusion that the front chain near Val70 should move about 1Å away from FeMo-co. This is consistent with experimental data on mutation of amino acid 70 with smaller and larger side chains.[8] Therefore the Cα(Trp72)-C^c distance was constrained to 14.1Å (13.08Å in the resting protein structure), and the Cα(Val70)-C^c distance to 8.6Å (7.22Å in the resting protein). The lengths of the hydrogen bonds between the backside water HOH531 and Arg277, Tyr281 and Ser192 (Chart S1) were also constrained.

4. Density functional procedures

Density functional (DF) calculations use the DMol methodology of Delley,[9] with accurate DNP (double numerical plus polarisation) basis sets.[9d] The real-space cutoff in the calculation of atomic basis sets was 9 au (4.76Å), which gives results within 0.1% of the limit for larger extensions of the basis sets. The gradient-corrected functional PBE[10] was used because validation tests demonstrate that when used with DMol it is more accurate than other commonly used functionals.[11] See ref [7] for results describing the accuracy with which the PBE/DMol method reproduces experimental enthalpy data on the binding of N_2. The calculations were all-electron, spin-unrestricted, with no imposed symmetry. The conductor-like screening model (COSMO)[12] was used with a dielectric constant of 5. Constraints on interatomic distances used the Lagrange Multiplier Algorithm. Control of electronic states was via the input spin populations for Fe1, Fe3, Fe4, Fe5 and Fe7. Output spin populations are calculated by the Mulliken method.[13]

5. Determination of transition states

The following pragmatic procedure is used to map reaction energy surfaces and locate transition states. [5, 6c, 14]

A chemically reasonable geometry between energy minima is evaluated by observing the directions and extent of geometry change during an energy minimisation calculation that is restricted to small (ca 0.03 Å) atom displacements at each cycle. This reveals significant geometrical variables (i.e. which atoms are moving, which bond distances and angles are changing) and also the energy gradients involved. This calculation also indicates the direction of the energy saddle relative the 'reactant' and 'product' minima. Then, as illustrated in Figure S2, a point Δ1 that is a short distance along this first optimisation is selected, and from this structure an atomic arrangement (2 on Figure S2) on the other side of the saddle is found (by trial and error and chemical experience) and similarly evaluated with small-displacement energy minimisation steps. A point Δ2 along the second optimisation is selected, and from this the key moving atoms are adjusted to positions just back across the suspected saddle-point towards the 'reactant' state. Repetition of this cycle, with subsequent short, small-step energy minimisations, always starting from a point just on the other side of the barrier, allows the barrier to be

straddled from opposing trial positions that progressively approach each other in geometry and energy, and converge at the transition state (TS). Confirmation of the TS is obtained by following the complete trajectory of geometries from positions nudged just away from the TS, linking the TS with the connected reactant and product energy minima on the same potential energy surface. The protocol requires human participation, which is itself insightful, and with chemical experience the procedure works efficiently.

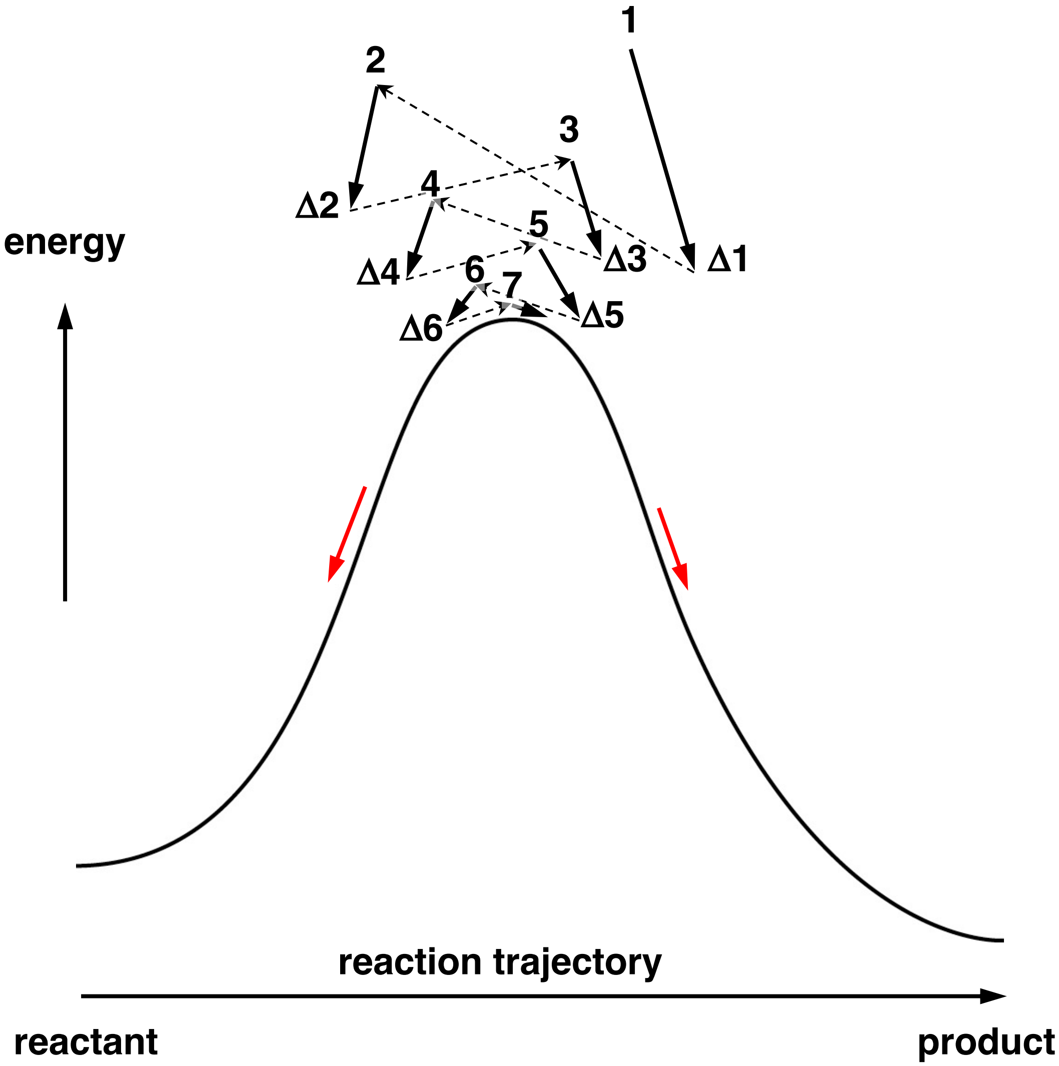

Figure S2. Generalised diagram of the procedure for locating a reaction transition state. The solid arrows represent the course of limited small-step energy reductions from the numbered positions, which are generated (broken arrows) from Δ positions partway along the previous energy reduction, chosen according to geometry and energy gradient. The postulated trials necessarily oscillate across the barrier, and therefore optimise all other variables and decrease the energy and gradient while approaching the top of the barrier. Confirmation that the transition state, reactant and product are on the same potential energy surface is obtained by complete energy minimisations (red arrows).

6. References

[1] B. Benediktsson, R. Bjornsson, *Inorg. Chem.* **2017**, *56*, 13417-13429.

[2] L. Deng, H. Wang, C. H. Dapper, W. E. Newton, S. Shilov, S. Wang, S. P. Cramer, Z.-H. Zhou, *Communications Chemistry* **2020**, *3*, 145.

[3] L. Cao, O. Caldararu, U. Ryde, *J. Phys. Chem. B* **2017**, *121*, 8242-8262.

[4] I. Dance, *J. Inorg. Biochem.* **2017**, *169*, 32-43.

[5] I. Dance, *Dalton Trans.* **2015**, *44*, 18167-18186.

[6] a) T. Spatzal, J. Schlesier, E.-M. Burger, D. Sippel, L. Zhang, S. L. A. Andrade, D. C. Rees, O. Einsle, *Nat Commun* **2016**, *7*, 10902; b) R. Bjornsson, F. Neese, S. DeBeer, *Inorg. Chem.* **2017**, *56*, 1470-1477; c) I. Dance, *Chembiochem* **2020**, *21*, 1671-1709.

[7] I. Dance, *Dalton Trans.* **2021**, *50*, 18212-18237.

[8] a) P. C. Dos Santos, R. Igarashi, H.-I. Lee, B. M. Hoffman, L. C. Seefeldt, D. R. Dean, *Acc. Chem. Res.* **2005**, *38*, 208-214; b) L. C. Seefeldt, B. M. Hoffman, D. R. Dean, *Annu. Rev. Biochem.* **2009**, *78*, 701-722; c) R. Sarma, B. M. Barney, S. Keable, D. R. Dean, L. C. Seefeldt, J. W. Peters, *J. Inorg. Biochem.* **2010**, *104*, 385-389.

[9] a) B. Delley, *J. Chem. Phys.* **1990**, *92*, 508-517; b) B. Delley, in *Modern density functional theory: a tool for chemistry, Vol. 2* (Eds.: J. M. Seminario, P. Politzer), Elsevier, Amsterdam, **1995**, pp. 221-254; c) J. Baker, A. Kessi, B. Delley, *J. Chem. Phys.* **1996**, *105*, 192-212; d) B. Delley, *J. Chem. Phys.* **2000**, *113*, 7756-7764; e) J. Andzelm, R. D. King-Smith, G. Fitzgerald, *Chem. Phys. Lett.* **2001**, *335*, 321-326; f) T. Todorova, B. Delley, *J. Phys. Chem. C* **2010**, *114*, 20523-20530.

[10] J. P. Perdew, K. Burke, M. Ernzerhof, *Phys. Rev. Lett.* **1996**, *77*, 3865-3868.

[11] I. Dance, *Mol. Simul.* **2018**, *44*, 568-581.

[12] a) J. Andzelm, C. Kolmel, A. Klamt, *J. Chem. Phys.* **1995**, *103*, 9312-9320; b) A. Klamt, V. Jonas, T. Burger, J. C. W. Lohrenz, *J. Phys. Chem. A* **1998**, *102*, 5074-5085; c) B. Delley, *Mol. Simul.* **2006**, *32*, 117-123.

[13] R. S. Mulliken, *J. Chem. Phys.* **1955**, *23*, 1833-1846.

[14] a) I. Dance, *Mol. Simul.* **2008**, *34*, 923-929; b) I. Dance, *Mol. Simul.* **2011**, *37*, 257.

7. Energies and spin populations for intermediates and transition states

intermediate	electronic state (S)	energy (kcal mol^{-1})	spin populations	
**1**	35(2)	-53231.61	Fe1	2.79
			Fe2	-0.19
			Fe3	-2.56
			Fe4	2.85
			Fe5	-2.39
			Fe6	1.05
			Fe7	2.14
**TS 1 → 2**	35(2)	-53218.88	Fe1	3.16
			Fe2	0.15
			Fe3	-2.62
			Fe4	2.75
			Fe5	-2.42
			Fe6	0.12
			Fe7	2.22
**2**	35(2)	-53227.41	Fe1	3.20

			Fe2	0.14
			Fe3	-2.61
			Fe4	2.79
			Fe5	-2.50
			Fe6	0.18
			Fe7	2.24
**1**	47(2)	-53230.36	Fe1	3.18
			Fe2	0.20
			Fe3	2.72
			Fe4	-2.66
			Fe5	2.36
			Fe6	-0.21
			Fe7	-2.03
**TS 1 → 2**	47(2)	-53218.66	Fe1	3.14
			Fe2	0.16
			Fe3	2.68
			Fe4	-2.61
			Fe5	2.44
			Fe6	0.07
			Fe7	-2.23
**2**	47(2)	-53227.77	Fe1	3.17
			Fe2	0.15
			Fe3	2.65
			Fe4	-2.63
			Fe5	2.52
			Fe6	-0.07
			Fe7	-2.28
**2**	35(2)	-53228.12	Fe1	3.18
			Fe2	0.17
			Fe3	-2.60
			Fe4	2.78
			Fe5	-2.50
			Fe6	0.09
			Fe7	2.32
**TS 2 → 3**	35(2)	-53225.59	Fe1	3.21
			Fe2	0.11
			Fe3	-2.61
			Fe4	2.82
			Fe5	-2.51
			Fe6	0.14
			Fe7	2.29
**3**	35(2)	-53246.61	Fe1	3.23
			Fe2	0.06
			Fe3	-2.65

			Fe4	2.88
			Fe5	-2.30
			Fe6	-0.19
			Fe7	2.40
**2**	47(2)	-53228.05	Fe1	3.18
			Fe2	0.16
			Fe3	2.64
			Fe4	-2.65
			Fe5	2.53
			Fe6	-0.06
			Fe7	-2.27
**TS 2 → 3**	47(2)	-53224.88	Fe1	3.20
			Fe2	0.13
			Fe3	2.68
			Fe4	-2.66
			Fe5	2.51
			Fe6	-0.09
			Fe7	-2.28
**3**	47(2)	-53246.99	Fe1	3.22
			Fe2	0.14
			Fe3	2.80
			Fe4	-2.65
			Fe5	2.29
			Fe6	0.06
			Fe7	-2.37
**3**	35(2)	-53246.01	Fe1	3.24
			Fe2	0.05
			Fe3	-2.61
			Fe4	2.88
			Fe5	-2.19
			Fe6	-0.38
			Fe7	2.46
**TS 3 → 4**	35(2)	-53241.78	Fe1	3.20
			Fe2	-0.08
			Fe3	-2.67
			Fe4	2.77
			Fe5	-2.42
			Fe6	0.43
			Fe7	2.27
**4**	35(2)	-53245.56	Fe1	3.12
			Fe2	-0.43
			Fe3	-2.68
			Fe4	2.74
			Fe5	-2.45

			Fe6	0.85
			Fe7	2.36
**3**	47(2)	-53247.98	Fe1	3.21
			Fe2	0.12
			Fe3	2.80
			Fe4	-2.66
			Fe5	2.15
			Fe6	0.29
			Fe7	-2.46
**TS 3 → 4**	47(2)	-53242.37	Fe1	3.15
			Fe2	0.39
			Fe3	2.83
			Fe4	-2.53
			Fe5	2.42
			Fe6	-0.43
			Fe7	-2.30
**4**	47(2)	-53245.41	Fe1	3.06
			Fe2	0.73
			Fe3	2.82
			Fe4	-2.52
			Fe5	2.42
			Fe6	-0.60
			Fe7	-2.31
**4**	35(2)	-53250.29	Fe1	2.89
			Fe2	-0.55
			Fe3	-2.51
			Fe4	2.79
			Fe5	-2.40
			Fe6	1.03
			Fe7	2.34
**TS 4 → 6**	35(2)	-53243.07	Fe1	2.94
			Fe2	-0.98
			Fe3	-2.44
			Fe4	2.85
			Fe5	-2.44
			Fe6	1.25
			Fe7	2.36
**6**	35(2)	-53255.82	Fe1	2.91
			Fe2	-1.05
			Fe3	-2.37
			Fe4	3.01
			Fe5	-2.38
			Fe6	1.35
			Fe7	2.16

**4**	47(2)	-53244.74	Fe1	3.01
			Fe2	1.02
			Fe3	2.76
			Fe4	-2.55
			Fe5	2.45
			Fe6	-0.66
			Fe7	-2.34
**TS 4 → 6**	47(2)	-53240.91	Fe1	3.02
			Fe2	1.06
			Fe3	2.78
			Fe4	-2.59
			Fe5	2.46
			Fe6	-0.65
			Fe7	-2.34
**6**	47(2)	-53251.05	Fe1	3.04
			Fe2	0.45
			Fe3	2.82
			Fe4	-2.60
			Fe5	2.35
			Fe6	-0.17
			Fe7	-2.26
**3**	35(2)	-53246.89	Fe1	3.23
			Fe2	0.07
			Fe3	-2.61
			Fe4	2.89
			Fe5	-2.22
			Fe6	-0.40
			Fe7	2.48
**TS 3 → 5**	35(2)	-53238.11	Fe1	3.21
			Fe2	0.13
			Fe3	-2.51
			Fe4	2.97
			Fe5	-2.23
			Fe6	-0.80
			Fe7	2.52
**5**	35(2)	-53250.27	Fe1	3.22
			Fe2	0.08
			Fe3	-2.66
			Fe4	2.95
			Fe5	-2.31
			Fe6	-0.01
			Fe7	2.16
**3**	47(2)	-53245.83	Fe1	3.22
			Fe2	0.13

			Fe3	2.79
			Fe4	-2.65
			Fe5	2.28
			Fe6	0.12
			Fe7	-2.38
**TS 3 → 5**	47(2)	-53237.43	Fe1	3.21
			Fe2	0.16
			Fe3	2.80
			Fe4	-2.68
			Fe5	2.22
			Fe6	0.26
			Fe7	-2.46
**5**	476(2)	-53253.26	Fe1	3.21
			Fe2	0.05
			Fe3	2.88
			Fe4	-2.57
			Fe5	2.34
			Fe6	-0.29
			Fe7	-2.20
**5**	35(2)	-53253.66	Fe1	3.24
			Fe2	0.04
			Fe3	-2.66
			Fe4	2.92
			Fe5	-2.37
			Fe6	0.14
			Fe7	2.16
**TS 5 → 6**	35(2)	-53249.55	Fe1	3.18
			Fe2	-0.06
			Fe3	-2.57
			Fe4	2.90
			Fe5	-2.43
			Fe6	0.28
			Fe7	2.20
**6**	35(2)	-53255.11	Fe1	2.93
			Fe2	-1.02
			Fe3	-2.30
			Fe4	3.03
			Fe5	-2.41
			Fe6	1.26
			Fe7	2.09
**5**	47(2)	-53254.61	Fe1	3.20
			Fe2	0.07
			Fe3	2.89
			Fe4	-2.64

			Fe5	2.37
			Fe6	-0.31
			Fe7	-2.18
**TS 5 → 6**	47(2)	-53249.09	Fe1	3.14
			Fe2	0.26
			Fe3	2.85
			Fe4	-2.61
			Fe5	2.38
			Fe6	-0.33
			Fe7	-2.19
**6**	47(2)	-53251.75	Fe1	3.03
			Fe2	0.45
			Fe3	2.82
			Fe4	-2.61
			Fe5	2.38
			Fe6	-0.27
			Fe7	-2.22
**7**	35(1/2)	-53082.34	Fe1	3.00
			Fe2	-1.75
			Fe3	-2.57
			Fe4	2.93
			Fe5	-2.20
			Fe6	-1.16
			Fe7	2.09
**TS 7 → 8**	35(1/2)	-53081.06	Fe1	2.96
			Fe2	-1.51
			Fe3	-2.61
			Fe4	2.86
			Fe5	-2.25
			Fe6	-1.02
			Fe7	2.04
**8**	35(1/2)	-53085.16	Fe1	2.98
			Fe2	-1.69
			Fe3	-2.56
			Fe4	2.94
			Fe5	-2.25
			Fe6	-0.57
			Fe7	1.77
**7**	35(3/2)	-53084.97	Fe1	3.02
			Fe2	-1.91
			Fe3	-2.61
			Fe4	2.81
			Fe5	-2.37
			Fe6	1.99

			Fe7	2.01
**TS 7 → 8**	35(3/2)	-53079.91	Fe1	3.00
			Fe2	-1.72
			Fe3	-2.61
			Fe4	2.85
			Fe5	-2.36
			Fe6	1.43
			Fe7	2.24
**8**	35(3/2)	-53082.63	Fe1	3.01
			Fe2	-1.00
			Fe3	-2.37
			Fe4	3.03
			Fe5	-2.38
			Fe6	1.16
			Fe7	1.29
**7**	47(3/2)	-53082.97	Fe1	2.92
			Fe2	-1.58
			Fe3	2.84
			Fe4	-2.67
			Fe5	2.24
			Fe6	1.19
			Fe7	-2.26
**TS 7 → 8**	47(3/2)	-53080.01	Fe1	2.96
			Fe2	-1.59
			Fe3	2.76
			Fe4	-2.63
			Fe5	2.13
			Fe6	1.05
			Fe7	-2.23
**8**	47(3/2)	-53085.03	Fe1	2.89
			Fe2	-1.33
			Fe3	2.86
			Fe4	-2.67
			Fe5	2.39
			Fe6	-0.41
			Fe7	-1.42
**8**	35(1/2)	-53083.84	Fe1	2.99
			Fe2	-1.75
			Fe3	-2.60
			Fe4	2.93
			Fe5	-2.26
			Fe6	-0.33
			Fe7	1.70
**TS 8 → 9**	35(1/2)	-53081.92	Fe1	3.00

			Fe2	-1.73
			Fe3	-2.54
			Fe4	2.95
			Fe5	-2.27
			Fe6	-1.14
			Fe7	2.03
**9**	35(1/2)	-53085.23	Fe1	2.97
			Fe2	-1.52
			Fe3	-2.46
			Fe4	2.93
			Fe5	-2.32
			Fe6	-1.65
			Fe7	2.32
**8**	47(1/2)	-53083.05	Fe1	2.92
			Fe2	-1.12
			Fe3	2.59
			Fe4	-2.73
			Fe5	2.34
			Fe6	-1.95
			Fe7	-1.85
**TS 8 → 9**	47(1/2)	-53079.40	Fe1	2.91
			Fe2	-1.38
			Fe3	2.87
			Fe4	-2.55
			Fe5	2.51
			Fe6	-1.85
			Fe7	-2.33
**9**	47(1/2)	-53087.24	Fe1	2.91
			Fe2	-1.56
			Fe3	2.84
			Fe4	-2.48
			Fe5	2.39
			Fe6	-1.66
			Fe7	-2.22
**9**	35(1/2)	-53086.54	Fe1	2.94
			Fe2	-1.56
			Fe3	-2.50
			Fe4	2.88
			Fe5	-2.31
			Fe6	-1.27
			Fe7	2.21
**TS 9 → 10**	35(1/2)	-53084.92	Fe1	2.93
			Fe2	-1.46
			Fe3	-2.52

			Fe4	2.89
			Fe5	-2.28
			Fe6	-1.45
			Fe7	2.28
**10**	35(1/2)	-53096.45	Fe1	2.88
			Fe2	-1.39
			Fe3	-2.59
			Fe4	2.81
			Fe5	-2.26
			Fe6	-1.26
			Fe7	2.35
**9**	35(3/2)	-53087.15	Fe1	2.99
			Fe2	-1.58
			Fe3	-2.60
			Fe4	2.79
			Fe5	-2.41
			Fe6	1.51
			Fe7	2.09
**TS 9 → 10**	35(3/2)	-53086.18	Fe1	3.00
			Fe2	-1.66
			Fe3	-2.60
			Fe4	2.80
			Fe5	-2.42
			Fe6	1.61
			Fe7	2.09
**10**	35(3/2)	-53094.59	Fe1	2.92
			Fe2	-1.29
			Fe3	-2.61
			Fe4	2.84
			Fe5	-2.42
			Fe6	1.09
			Fe7	2.17
**9**	47(1/2)	-53088.01	Fe1	2.92
			Fe2	-1.53
			Fe3	2.83
			Fe4	-2.50
			Fe5	2.38
			Fe6	-1.64
			Fe7	-2.22
**TS 9 → 10**	47(1/2)	-53086.51	Fe1	2.90
			Fe2	-1.55
			Fe3	2.82
			Fe4	-2.54
			Fe5	2.39

			Fe6<br>Fe7	-1.50<br>-2.20
**10**	47(1/2)	-53095.83	Fe1<br>Fe2<br>Fe3<br>Fe4<br>Fe5<br>Fe6<br>Fe7	2.85<br>-1.35<br>2.73<br>-2.60<br>2.44<br>-1.28<br>-2.18
**9**	47(3/2)	-53084.02	Fe1<br>Fe2<br>Fe3<br>Fe4<br>Fe5<br>Fe6<br>Fe7	2.97<br>-1.68<br>2.77<br>-2.61<br>2.23<br>1.40<br>-2.31
**TS 9 → 10**	47(3/2)	-53083.44	Fe1<br>Fe2<br>Fe3<br>Fe4<br>Fe5<br>Fe6<br>Fe7	2.98<br>-1.59<br>2.75<br>-2.65<br>2.18<br>1.36<br>-2.28
**10**	47(3/2)	-53098.79	Fe1<br>Fe2<br>Fe3<br>Fe4<br>Fe5<br>Fe6<br>Fe7	2.85<br>-1.23<br>2.81<br>-2.61<br>2.23<br>1.00<br>-2.31
**11**	35(1)	-53125.89	Fe1<br>Fe2<br>Fe3<br>Fe4<br>Fe5<br>Fe6<br>Fe7	2.86<br>-0.85<br>-2.23<br>2.92<br>-2.31<br>-1.30<br>2.14
**TS 11 → 12**	35(1)	-53123.16	Fe1<br>Fe2<br>Fe3<br>Fe4<br>Fe5<br>Fe6<br>Fe7	2.83<br>-0.66<br>-2.39<br>2.93<br>-2.37<br>-1.07<br>2.15

**12**	35(1)	-53130.52	Fe1 2.89<br>Fe2 -1.14<br>Fe3 -2.33<br>Fe4 2.80<br>Fe5 -2.31<br>Fe6 -0.57<br>Fe7 2.28
**12**	35(1)	-53130.67	Fe1 2.93<br>Fe2 -1.35<br>Fe3 -2.53<br>Fe4 2.76<br>Fe5 -2.35<br>Fe6 0.02<br>Fe7 2.17
**TS 12 → 13**	35(1)	-53121.67	Fe1 2.95<br>Fe2 -1.40<br>Fe3 -2.58<br>Fe4 2.76<br>Fe5 -2.39<br>Fe6 0.40<br>Fe7 1.88
**13**	35(1)	-53140.10	Fe1 2.91<br>Fe2 -1.34<br>Fe3 -2.58<br>Fe4 2.77<br>Fe5 -2.39<br>Fe6 0.04<br>Fe7 2.28
**12**	47(1)	-53129.77	Fe1 2.87<br>Fe2 -1.06<br>Fe3 2.68<br>Fe4 -2.48<br>Fe5 2.40<br>Fe6 -0.67<br>Fe7 -2.23
**TS 12 → 13**	47(1)	-53120.30	Fe1 2.87<br>Fe2 -1.25<br>Fe3 2.76<br>Fe4 -2.54<br>Fe5 2.42<br>Fe6 -0.65<br>Fe7 -1.97
**13**	47(1)	-53138.14	Fe1 2.88<br>Fe2 -1.36

			Fe3 2.79<br>Fe4 -2.54<br>Fe5 2.38<br>Fe6 -0.26<br>Fe7 -2.31
**13**	47(1)	-53138.14	Fe1 2.88<br>Fe2 -1.36<br>Fe3 2.79<br>Fe4 -2.54<br>Fe5 2.38<br>Fe6 -0.26<br>Fe7 -2.31
**TS 13 → 1**	47(1)	-53131.91	Fe1 3.00<br>Fe2 -1.06<br>Fe3 2.80<br>Fe4 -2.62<br>Fe5 2.33<br>Fe6 -0.81<br>Fe7 -2.26
**1**	47(1)	-53140.67	Fe1 2.46<br>Fe2 -0.01<br>Fe3 2.69<br>Fe4 -2.56<br>Fe5 2.38<br>Fe6 -1.12<br>Fe7 -2.21

8. Coordinates and spin populations for intermediates and transitions states

Translation of Fe numbering

Fe1 = Fe143

Fe2 = Fe140

Fe3 = Fe141

Fe4 = Fe142

Fe5 = Fe139

Fe6 = Fe145

Fe7 = Fe144

1 + H_2 35 (S=2)

structure, spin populations	filename, coordinates
**1 + H_2 35 (S=2)**<br>Fe(139) -2.391<br>Fe(140) -0.186<br>Fe(141) -2.564	bm22b2n2x6x6h2n35dtk.car_3<br>C1 2.031428576 -8.212347972 -4.227747854<br>C2 3.427073562 -8.807012578 -4.014109601<br>C3 0.267596913 -7.677742568 -5.849299645<br>C4 -0.939174175 -8.260235996 -5.115856140

Fe(142) 2.853<br>Fe(143) 2.790<br>Fe(144) 2.136<br>Fe(145) 1.045	C5 -1.985687803 -10.221843537 -4.102928174<br>C6 -2.140535335 -9.728412254 -2.661198504<br>C7 -1.030435417 -8.791100494 -0.693012041<br>C8 -1.582639978 -7.362388333 -0.676679972<br>C9 -1.671099980 -5.182860326 -1.769880073<br>C10 -3.175886890 -5.121120065 -2.071711774<br>C11 -5.085561472 -6.091811331 -3.216515818<br>C12 -5.955891756 -6.645969254 -2.080373826<br>C13 -6.346915004 -8.506174613 -0.536844546<br>C14 -6.235988385 -8.094188785 0.935888494<br>C15 -4.759166231 -7.301132778 2.738806700<br>C16 -4.640743213 -5.814835916 3.095491397<br>C17 -4.493329226 -3.510307014 2.166156953<br>C18 -5.775408050 -2.777419660 1.733133061<br>C19 -3.230195952 -3.031328107 1.413951988<br>C20 -3.161292094 -1.500841701 1.358649422<br>C21 -1.978463524 -3.658488304 2.038070019<br>C22 -7.331135335 -2.262295490 -0.109060372<br>C23 -8.614435045 -3.021447894 0.288016197<br>C24 -7.210013172 -2.047250892 -1.637930773<br>C25 -8.511447026 -1.476759086 -2.210573448<br>C26 -6.026746430 -1.127836600 -1.969544094<br>C27 -9.822824812 -5.124878796 0.363734512<br>C28 -10.291915124 -4.928073255 1.791111920<br>C29 0.272662107 -7.819847099 10.940773598<br>C30 0.372449684 -6.304697004 10.746661186<br>C31 -0.921333398 -4.324147790 9.932081771<br>C32 -1.985967547 -3.807621972 10.918881630<br>C33 -1.340883095 -4.096981104 8.467175153<br>C34 -1.592315477 -2.628711579 8.110428865<br>C35 -1.709123462 -2.450536244 6.595363029<br>C36 -2.703494120 -0.496836367 5.383139399<br>C37 -2.541749567 -2.083068615 12.566721309<br>C38 -3.149049995 -0.773304762 12.108597031<br>C39 2.909492634 -3.870069334 -3.623126601<br>C40 2.029703015 -3.058897628 -4.589476041<br>C41 2.762050546 -3.236363123 -2.220264944<br>C42 3.325737138 -4.053734106 -1.037465473<br>C43 4.788061930 -3.772057987 -0.697528721<br>C44 2.081900809 -1.302435709 -6.303044685<br>C45 1.396402865 -1.887350219 -7.546433589<br>C46 3.023189202 -0.144774096 -6.691468050<br>C47 0.602239036 -3.924846213 -8.654931149<br>C48 -0.802337488 -4.233227009 -8.115769931<br>C49 -2.293257271 -4.276737068 -6.160191429<br>C50 -3.051326369 -3.046827457 -5.659615210<br>C51 -3.021670566 -0.652958050 -5.114636898<br>C52 -4.119482000 -0.152344492 -6.088152123<br>C53 -2.037695808 0.520165707 -4.982575067<br>C54 -0.905905767 0.391124007 -4.012354682<br>C55 -0.684372651 -0.346122285 -2.873429320<br>C56 1.045127413 1.024006387 -3.131012537<br>C57 -4.620399691 0.257160578 -8.476015368<br>C58 -6.060656830 -0.247366790 -8.416715940<br>C59 -3.953081001 -0.134388756 -9.815507558<br>C60 -4.277413734 0.727882983 -11.000292371<br>C61 -5.479337328 1.253475262 -11.434066491<br>C62 -3.846058646 1.814643449 -12.821737772<br>C63 -5.001467252 3.613681012 7.611914958

	C64	-4.470584737	3.166716284	8.962336651
	C65	-4.381827149	2.668645544	6.551482167
	C66	-4.850720513	2.976267999	5.148684814
	C67	-6.021093938	2.398845603	4.621855675
	C68	-4.133352008	3.870269813	4.333770702
	C69	-6.459691852	2.698487204	3.327411643
	C70	-4.562338885	4.184505740	3.043480202
	C71	-5.726213050	3.596157712	2.540256906
	C72	-2.330410315	8.702568821	1.453222091
	C73	-2.345476623	9.156629642	-0.011710885
	C74	-2.062123516	7.192053593	1.545116737
	C75	-1.196133405	10.436268770	-1.763215984
	C76	-0.789084166	9.392533227	-2.805745898
	C77	0.149349110	7.138564658	-3.190936706
	C78	-1.027500337	6.169602809	-3.445345856
	C79	1.386036146	6.448109722	-2.588509285
	C80	2.003217280	5.285711427	-3.371725484
	C81	2.728960944	5.633739675	-4.683773084
	C82	1.616172364	6.434445073	-6.774381819
	C83	-3.036359968	5.069650819	-2.592464294
	C84	-4.239527136	5.766995932	-3.253736836
	C85	-3.525310294	4.432519800	-1.282284165
	C86	-5.648428085	7.761722215	-3.212227081
	C87	-5.876116284	8.566955943	-4.488564288
	C88	-4.943176657	9.395384661	-6.605756383
	C89	-5.671920153	8.758171705	-7.808440420
	C90	-3.540204022	9.895741929	-7.016848493
	C91	-2.622446357	8.744858148	-7.411851375
	C92	-6.483692998	6.682495990	-8.838991159
	C93	-7.933939778	7.087178232	-9.088728983
	C94	-6.400464163	5.158214310	-8.557921223
	C95	-4.973109949	4.676781652	-8.441421807
	C96	-4.227373283	4.382534498	-9.592904200
	C97	-4.339452698	4.562403699	-7.193739609
	C98	-2.882720448	4.014180243	-9.512821897
	C99	-2.987395068	4.214357195	-7.093373246
	C100	-2.272495645	3.949569837	-8.259018335
	C101	9.383955448	2.655004041	4.304472520
	C102	8.967746684	1.933799622	3.033796066
	C103	7.274999063	1.845518941	1.222473708
	C104	7.241644435	2.840482050	0.057512939
	C105	6.188912459	4.871026766	-0.813598010
	C106	5.714616670	6.245353292	-0.352728519
	C107	4.836837336	7.684209303	1.424712328
	C108	5.878896766	8.527479406	2.133720022
	C109	9.995969004	-0.295961214	-1.777786707
	C110	9.379379438	-0.081458425	-3.163779418
	C111	9.016414220	-1.116361444	-0.906352600
	C112	9.719238273	-1.716079072	0.325521598
	C113	8.698246736	-2.236570477	1.310667868
	C114	7.800880608	1.179614944	-4.537814752
	C115	7.797182822	2.655788097	-4.878689271
	C116	6.422198496	0.482914226	-4.648416649
	C117	5.549979085	0.751333239	-3.449156121
	C118	4.817632770	1.945727212	-3.336519147
	C119	5.550109755	-0.142201785	-2.362621184
	C120	4.155897130	2.257575380	-2.143649367
	C121	4.883922954	0.169545860	-1.172660954
	C122	4.207553581	1.386871112	-1.051199768

	C123	5.947483111	0.852401144	9.298874371
	C124	4.981629334	0.892839881	10.494299710
	C125	5.806471414	-0.437106495	8.475640040
	C126	6.578014285	-0.481781897	7.193142156
	C127	7.946299483	-0.584231483	7.037825004
	C128	6.965544306	-0.714414664	5.056578053
	C129	3.913839201	-0.240483890	12.361128787
	C130	4.255030734	0.862336860	13.338453920
	C131	2.907834375	-5.148374201	3.860053974
	C132	2.462660085	-4.260943030	4.988559585
	C133	3.652999943	-3.513313279	5.717927281
	C134	4.698267902	-4.554161026	6.206748983
	C135	5.801254953	-3.906261549	7.060358697
	C136	6.679594381	-4.897684887	7.767026754
	C137	3.037119802	-2.854752252	6.992054945
	C138	1.937596178	2.093603769	3.261321131
	Fe139	3.877627191	1.886339724	3.812641230
	Fe140	0.505661793	2.175158202	1.497235127
	Fe141	0.619657198	3.341849526	4.088596601
	Fe142	2.706206131	3.796557546	2.620119221
	Fe143	0.341897717	4.886504486	1.916467061
	Fe144	1.725547672	1.288271044	5.044462081
	Fe145	2.324493167	0.021579398	2.887969363
	H146	3.431437836	-8.650780020	-1.980984635
	H147	4.283303669	-7.472352994	-2.723528240
	H148	2.150603228	-8.608442144	-6.246639256
	H149	4.077359385	-8.509892428	-4.852114321
	H150	3.319118037	-9.903301955	-4.087063396
	H151	0.270703390	-6.601275981	-5.624621677
	H152	-0.087820475	-10.126608789	-5.113713908
	H153	0.113322653	-7.800746509	-6.929402793
	H154	-1.801923965	-11.304587111	-4.088401649
	H155	-0.128930728	-9.320763322	-2.552943157
	H156	-2.944994084	-10.042670168	-4.605794887
	H157	-1.516937035	-4.638496484	-0.829436392
	H158	-0.437937272	-6.859086523	-2.322966549
	H159	-0.008991532	-8.791764395	-0.289397847
	H160	-5.442069005	-5.077163155	-3.430660629
	H161	-3.032229866	-6.717085939	-3.383409815
	H162	-1.668751147	-9.388227682	-0.030735862
	H163	-1.117165417	-4.661395856	-2.563837149
	H164	-4.672062447	-8.251695734	-1.833964390
	H165	-5.238724857	-6.713564600	-4.109031040
	H166	-2.022943393	-4.756094624	1.997983889
	H167	-1.073722517	-3.337065483	1.500596686
	H168	-1.862153314	-3.357637879	3.090807574
	H169	-3.310959688	-3.397755709	0.378361340
	H170	-6.091436654	-9.573833623	-0.596957077
	H171	-5.067111326	-1.544491655	-1.633472804
	H172	-7.866118315	-4.826156933	-0.435872072
	H173	-9.365542585	-2.151950294	-2.059853072
	H174	-2.279616594	-1.180344688	0.784422702
	H175	-3.066529257	-1.062285154	2.361816990
	H176	-6.096354418	3.446853296	7.613328312
	H177	-5.964911395	-0.974893705	-3.056610359
	H178	-8.399514934	-1.305041664	-3.289859712
	H179	-6.162072538	-0.140403229	-1.498763995
	H180	-8.755174572	-0.513356058	-1.736216988
	H181	-7.031531846	-3.034560229	-2.101661923

H182	-7.425056101	-1.282999482	0.384803553
H183	-5.515926437	-3.403182982	-0.227391514
H184	-4.050283270	-1.066024924	0.876979489
H185	-2.651460987	4.074626338	-0.716591444
H186	-4.143519406	3.563405308	-1.559255036
H187	-4.883854358	4.858083567	0.062650274
H188	-6.894997187	3.426493737	0.997956897
H189	-3.983616556	4.863507441	2.417216324
H190	-3.203605899	4.309690189	4.702236073
H191	-6.598908625	1.695373339	5.227265740
H192	-4.731989802	-5.365783604	1.116753940
H193	-3.819693486	-7.782188690	3.053038504
H194	-5.573979794	-7.717311330	3.346489819
H195	-4.231316874	-7.603779678	0.699447909
H196	-7.403092416	-8.388162388	-0.810421821
H197	-7.361433361	2.225383950	2.929538626
H198	-4.385761251	-3.305393190	3.238876233
H199	-10.655131291	-4.891821617	-0.321910633
H200	-9.578401756	-6.192216433	0.244471157
H201	-9.493573095	-4.639593664	2.514438589
H202	-2.031994971	-1.929961470	13.530009519
H203	6.073819307	-3.588284290	0.885172403
H204	2.879788674	-6.628535384	5.103519669
H205	10.353554056	-2.555592832	0.006860792
H206	10.356757570	-0.958833384	0.804850677
H207	8.575020367	-1.934743418	-1.496085244
H208	8.668694222	3.222252727	-4.476057172
H209	10.872796992	-0.930120993	-1.975636130
H210	3.230509191	-2.239087477	-2.239451657
H211	3.244317938	-5.134259768	-1.246286907
H212	8.253828175	1.520434076	-2.466266627
H213	8.453616070	0.725437375	-5.311169906
H214	9.718085846	1.544596653	-0.862472416
H215	4.398280208	-3.879249100	1.344112451
H216	8.182754526	-0.464358912	-0.603274846
H217	11.086961044	0.756929712	-0.396314478
H218	4.512731457	-4.745393870	-4.586140194
H219	1.687502121	-3.084884862	-2.044069172
H220	8.904763551	-0.558855648	2.323485864
H221	2.704422224	-3.870667560	-0.149574372
H222	-3.525898219	-0.852785884	-4.158238790
H223	-1.288572491	-1.102658788	-2.385472269
H224	-2.938181587	-4.758680179	-6.906235352
H225	-0.221778481	-3.761368687	-6.171578531
H226	-3.040651257	-0.931416727	-7.645269160
H227	-6.481035885	1.224760033	-11.021342002
H228	-6.156442600	-1.307954264	-8.079130106
H229	-4.173134622	-1.195679379	-10.028912754
H230	-4.629951681	1.352944762	-8.359703765
H231	-3.355104833	2.266083247	-13.679353803
H232	-2.638181605	1.399563938	-4.696255001
H233	-1.620418713	0.740473007	-5.978966928
H234	-5.845369250	2.464432838	-13.158629812
H235	-2.863051300	-0.069545824	-9.671492673
H236	-1.346984553	-1.884946834	-5.669267157
H237	-2.190620435	-4.983413170	-5.322735616
H238	0.469833797	-3.321214893	-9.561573025
H239	1.991461892	1.528363984	-2.956235107
H240	1.076337310	-4.871673054	-8.945366333

	H241	-5.181784979	5.627451326	7.939522475
	H242	-4.783812835	5.249735424	6.400665043
	H243	-1.593202277	6.211274642	-1.451226826
	H244	-2.739528290	4.257637233	-3.271272329
	H245	-3.735311424	7.534774539	-2.348051463
	H246	0.350984652	1.915713249	-4.932756479
	H247	1.413075904	2.221628497	-6.723299157
	H248	0.050716249	3.010789468	-6.926729576
	H249	3.439493649	0.304006080	-5.778663627
	H250	3.856537577	-0.523507935	-7.306523734
	H251	-3.123425667	10.480204138	-6.183410089
	H252	-3.665263276	10.555928776	-7.884619499
	H253	-1.628369784	8.780509750	-5.551008981
	H254	-1.258571211	7.448203922	-6.617735828
	H255	2.034509572	8.306477751	-7.483135597
	H256	3.594722967	4.968889325	-4.821474947
	H257	-2.319645392	3.774493853	-10.417303686
	H258	-4.892757741	4.742035370	-6.267797267
	H259	0.117423561	5.362897954	-7.706572903
	H260	1.128285497	6.067995921	-1.587611238
	H261	-0.197119726	8.103925931	-1.317521138
	H262	1.256652920	4.497598497	-3.557347493
	H263	2.067418268	-3.749668638	-7.093845663
	H264	-0.457505616	11.248885447	-1.790246151
	H265	3.752894027	-2.380271412	-5.345267030
	H266	2.152931976	7.226109677	-2.441653325
	H267	-2.493963695	4.195789748	-6.119083251
	H268	1.673469235	0.396359731	-7.990101594
	H269	-2.159635717	10.847474011	-2.094923682
	H270	3.180704103	7.696550407	-6.348651798
	H271	0.646079221	6.780136093	-8.541727711
	H272	4.935308562	-3.811243070	-3.264834173
	H273	2.455364313	-4.877471338	-3.587123966
	H274	-4.701544675	4.437805388	-10.575363585
	H275	1.247808992	-0.866298879	-5.727291925
	H276	0.387512654	7.557497469	-4.177995279
	H277	3.122254103	6.655985590	-4.625592932
	H278	2.749891879	4.844262560	-2.698324575
	H279	1.435201109	4.565873127	-6.006402287
	H280	-6.445782490	7.004153960	-3.175934523
	H281	-4.004281027	8.080599023	-5.177213071
	H282	-5.550169840	10.272499678	-6.338654558
	H283	-5.804478539	8.452269522	-2.371561201
	H284	-5.598260503	6.933345213	-6.897441009
	H285	9.055724307	3.703988647	4.269245729
	H286	8.813825894	2.168637645	5.123081898
	H287	5.664264584	1.708526532	8.668990826
	H288	11.077054355	2.877888336	5.422232647
	H289	11.137667014	1.623639808	4.367834938
	H290	-2.010185855	6.920473383	2.606814283
	H291	-4.366696250	8.611497710	1.557724007
	H292	-0.573690099	10.169450894	0.267140308
	H293	-2.892674918	6.651075340	1.069549791
	H294	-3.752477158	10.009681312	2.145909372
	H295	3.981498995	7.513822455	2.095002042
	H296	-1.531527178	9.236012290	1.992280618
	H297	-2.238955389	-4.701939804	8.257748906
	H298	0.034116237	-3.817939732	10.114131113
	H299	5.306643256	5.621243886	1.593666272

	H300	-3.192268333	1.210110099	4.378310369
	H301	7.158512674	5.015194641	-1.309491027
	H302	4.903222246	-0.525454585	-0.329810445
	H303	5.485896142	4.546460090	-1.598684066
	H304	-2.579840275	-2.988664884	6.200621110
	H305	5.676188692	3.756069404	0.988526812
	H306	-0.820147135	-2.879588018	6.104654792
	H307	-0.522756786	-4.490513886	7.842356077
	H308	-0.757374558	-2.005337552	8.463892257
	H309	-2.509805135	-2.258319523	8.595170392
	H310	6.604202032	-0.596643758	-4.749505171
	H311	-1.488255881	-6.400340392	10.018916716
	H312	0.950156393	-8.266537808	10.196197749
	H313	3.717441160	1.666655389	-0.118357020
	H314	-1.596287924	-8.357448334	11.616735817
	H315	-1.033968391	-9.319137077	10.417266028
	H316	-0.922224088	-0.502317056	6.313195602
	H317	3.603303300	3.192099962	-2.035871209
	H318	6.083810964	-1.092313944	-2.442644869
	H319	4.511588863	-3.566210496	11.145006714
	H320	6.134662550	-1.289186012	9.097711382
	H321	2.694096081	-3.297132709	9.552025916
	H322	5.907944274	-4.040774532	10.677448924
	H323	6.938893174	8.221486774	1.955495246
	H324	1.780277270	-0.748646903	7.978437360
	H325	1.896980992	-4.834791642	5.738238898
	H326	7.325027139	3.155136969	2.875422946
	H327	4.180102693	-5.322209862	6.801806507
	H328	9.068110962	-0.855207035	5.231746399
	H329	6.845172693	-0.837771070	3.988849181
	H330	0.707231523	-8.035753189	11.930635990
	H331	-1.547263398	1.158222704	4.957280892
	H332	0.840876399	-0.857017257	10.189684359
	H333	1.010586946	0.599117254	8.058006333
	H334	-0.737047713	-2.211894181	11.394793633
	H335	-4.238294637	-1.788142514	5.837302284
	H336	7.877391866	-6.330973223	7.462604506
	H337	6.424838785	-3.274320930	6.409976254
	H338	5.364236462	-3.266546567	7.835762037
	H339	5.151963906	-5.038028631	5.329448002
	H340	6.258458798	1.505225615	1.474368397
	H341	4.801385668	2.643136923	-4.177439585
	H342	5.954715883	0.839695042	-5.579213454
	H343	1.817801979	-3.476309567	4.570019397
	H344	1.543268381	-2.010942179	10.956772603
	H345	2.257662775	-4.145525033	10.781127426
	H346	-4.581317982	-0.679533233	4.568074023
	H347	8.752300216	-0.570014782	7.761598775
	H348	4.746105142	-0.602942881	8.246210939
	H349	7.878748731	0.989436835	0.915453569
	H350	4.477431707	8.265288138	0.559605411
	H351	-4.657766016	1.638978842	6.826263103
	H352	-3.284557555	2.752122798	6.611011054
	H353	7.403052732	1.961876859	10.226110095
	H354	7.593040501	0.348841323	10.428480618
	H355	4.045315456	-1.197223451	12.891302577
	H356	5.213276956	-1.140594363	10.932789450
	H357	5.327651169	1.174373430	13.336927298
	H358	2.854965763	-0.163132208	12.066579974

	H359	-3.473514650	3.596565717	9.224640192
	H360	-8.445176822	7.572699579	-8.222497312
	H361	-3.374568456	-2.789434929	12.730564462
	H362	-3.189291351	-0.627903842	11.001868105
	H363	-5.919850551	6.909488274	-9.759192403
	H364	-6.962380218	4.928189117	-7.639232494
	H365	-6.910856052	4.648845078	-9.387587947
	H366	2.284409783	-1.443375967	2.506784285
	H367	-0.567378354	3.386622813	-9.019621049
	H368	-0.320565777	1.096024008	2.164980612
	H369	-0.602971593	-1.383423870	2.815456651
	H370	-0.770064006	-0.663454890	2.680246126
	H371	0.544659907	-0.668352842	0.854607583
	H372	0.942563478	-0.169731743	-1.429512065
	Mo373	3.724825993	-0.546610026	5.112780329
	N374	4.075822245	-8.473675996	-2.756261190
	N375	1.541056310	-8.289702115	-5.497341743
	N376	-0.898481052	-9.583478488	-4.826746026
	N377	-0.995778874	-9.384455559	-2.020788963
	N378	-1.149440603	-6.537982686	-1.665632888
	N379	-3.664258511	-6.066735812	-2.910500315
	N380	-5.516426612	-7.787150116	-1.489838311
	N381	-5.027973540	-7.610293773	1.343696049
	N382	-4.671170064	-4.954580393	2.045954898
	N383	-6.135788497	-2.919098333	0.424690096
	N384	-8.624932718	-4.361569366	0.067897049
	N385	-1.078529469	-8.350739294	10.736309555
	N386	-0.714195789	-5.746069202	10.177658957
	N387	-1.788008681	-1.038472892	6.200488056
	N388	-3.883543065	-1.122027326	5.159254992
	N389	-2.426581207	0.644225120	4.738967879
	N390	-1.651003639	-2.676073415	11.587349151
	N391	4.301431418	-3.881631317	-4.082771465
	N392	5.097362846	-3.737951687	0.611897906
	N393	2.730084835	-2.280538365	-5.454028308
	N394	1.468169881	-3.222465353	-7.724739943
	N395	-1.001572197	-4.036561624	-6.779483713
	N396	-2.362356114	-1.862000935	-5.601476465
	N397	0.196017607	1.228625304	-4.146438178
	N398	0.533340287	0.059830554	-2.360107157
	N399	-3.844348191	-0.356231290	-7.402274663
	N400	-3.265593491	1.083033956	-11.878037331
	N401	-5.182296908	1.946079609	-12.588491441
	N402	-4.600304526	5.001818675	7.376853429
	N403	-3.604991139	8.996222343	2.126933543
	N404	-1.306705867	9.948964928	-0.402354154
	N405	-0.222841998	8.260530512	-2.334720507
	N406	1.876567537	5.493016157	-5.871287504
	N407	2.260963729	7.620040799	-6.770123238
	N408	0.623022599	6.250694762	-7.674251207
	N409	-1.879059820	5.944070701	-2.408418737
	N410	-4.357068918	7.110375983	-3.043745004
	N411	-4.880516488	8.543912614	-5.422377291
	N412	-1.838339155	8.260479382	-6.411864496
	N413	-5.919794723	7.428980392	-7.725380224
	N414	10.841013565	2.599207963	4.467047286
	N415	7.885975914	2.433202153	2.407377555
	N416	6.342056363	3.835390117	0.191369945
	N417	5.358142379	6.417445069	0.945802851

N418	10.491625099	0.959775309	-1.202232912
N419	8.495128141	0.949467501	-3.284142759
N420	7.340204369	1.063514755	9.738667563
N421	5.981221681	-0.558930240	5.936227109
N422	8.167436785	-0.731239908	5.685432455
N423	4.809935521	-0.248337763	11.216949562
N424	-1.214138642	2.122755476	-0.866080761
N425	-0.561960766	2.154207649	0.059053212
O426	1.356539821	-7.731180026	-3.298183434
O427	-1.904899134	-7.537473962	-4.821034033
O428	-3.259079656	-9.686989738	-2.123100233
O429	-2.389135711	-6.988245735	0.188870222
O430	-3.893735379	-4.236696083	-1.577706534
O431	-7.021440421	-6.088570877	-1.764621262
O432	-7.204113531	-8.254202286	1.691479935
O433	-4.503477451	-5.477410552	4.280143609
O434	-6.432019730	-2.100221848	2.537903610
O435	-9.590624012	-2.432398352	0.781045963
O436	-11.440958694	-5.135969606	2.150362483
O437	1.413279357	-5.704077098	11.087562920
O438	-3.061745975	-4.414392000	11.066818244
O439	-3.583286565	0.071712843	12.874913160
O440	0.780632640	-3.108614037	-4.576137379
O441	5.648735509	-3.599955843	-1.591410924
O442	0.749665329	-1.127560331	-8.297587519
O443	2.311259445	0.881098062	-7.402164771
O444	-1.669752940	-4.649507415	-8.892601114
O445	-4.240872661	-3.134817620	-5.346707551
O446	-5.096246488	0.482620118	-5.681589041
O447	-7.033452588	0.409524165	-8.742243517
O448	-5.048397747	2.388893916	9.707883741
O449	-6.099406111	3.928732333	1.253257238
O450	-3.256188805	8.838896640	-0.798780667
O451	-0.960499175	9.643825466	-4.015988285
O452	-1.171929676	5.656026014	-4.568038254
O453	-5.074722373	5.102081933	-3.882636899
O454	-4.287050822	5.372642423	-0.518782926
O455	-6.935956849	9.186578575	-4.647610054
O456	-6.008848140	9.449718621	-8.779218053
O457	-2.661177341	8.235493279	-8.542728150
O458	-8.510813815	6.872783654	-10.141858654
O459	-0.903103529	3.650954515	-8.141352081
O460	9.598743152	0.918062048	2.642127840
O461	8.011374230	2.730284180	-0.923046476
O462	5.659386753	7.152855938	-1.200535714
O463	5.600926574	9.481863922	2.841552663
O464	9.651916500	-0.837943618	-4.111420390
O465	8.121511563	-3.313090974	1.184166987
O466	8.387594578	-1.424630740	2.338535702
O467	6.953261271	3.205384291	-5.572895452
O468	4.430536707	1.953331754	10.845236797
O469	3.455059077	1.333682511	14.133103362
O470	3.126061179	-4.793603013	2.710103948
O471	3.094170060	-6.475458398	4.163026767
O472	6.822518667	-4.987592681	8.984194222
O473	7.339312379	-5.723763600	6.911021645
O474	3.143351465	-1.568866434	7.060862154
O475	2.530385887	-3.576534999	7.883531255
O476	4.227663492	-2.575255420	4.886844470

	O477 0.817272886 -1.339059960 11.055536730<br>O478 1.015511686 -0.305190036 8.427407538<br>O479 0.793800617 2.872494109 -6.288943864<br>O480 5.456026644 -3.623090768 11.439123479<br>O481 2.739026159 -3.309044990 10.552493631<br>S482 -0.493266174 6.666530057 0.706014792<br>S483 1.866720891 3.890722043 0.503342267<br>S484 4.534440160 -0.066599327 2.922193983<br>S485 -1.087333390 3.372057014 2.644478834<br>S486 1.563723998 0.231692599 0.754344682<br>S487 4.903871338 3.732521929 3.098630184<br>S488 1.397709975 -0.880190567 4.663737684<br>S489 1.545556486 5.363451072 3.808794453<br>S490 3.685534579 1.648930353 6.044965560<br>S491 0.051618033 2.378818460 6.003946193

TS 1 → 2 35 (S=2)

TS 1 → 2 35 (S=2)	bm22b2n2x6x6h2n35dtm_1_53218.911
Fe(139) -2.423<br>Fe(140) 0.147<br>Fe(141) -2.623<br>Fe(142) 2.748<br>Fe(143) 3.157<br>Fe(144) 2.221<br>Fe(145) 0.123	C1 1.337537297 -7.478039770 -5.611595471<br>C2 2.737049020 -8.103416554 -5.647022738<br>C3 -0.588907078 -6.710129909 -6.923543709<br>C4 -1.718308301 -7.387030080 -6.150876780<br>C5 -2.680888916 -9.468547249 -5.311052450<br>C6 -2.661261234 -9.188011200 -3.806243701<br>C7 -1.307484719 -8.555544690 -1.864591433<br>C8 -1.817304388 -7.139197916 -1.583672103<br>C9 -1.970134476 -4.824743591 -2.332023508<br>C10 -3.497308643 -4.702911781 -2.433217170<br>C11 -5.557274766 -5.466976991 -3.471910003<br>C12 -6.302500012 -6.182728701 -2.336504980<br>C13 -6.571495275 -8.265295093 -1.073116946<br>C14 -6.273529804 -8.102520293 0.421150881<br>C15 -4.582867017 -7.601619828 2.134660728<br>C16 -4.378016071 -6.190517009 2.690573656<br>C17 -4.301744770 -3.778076406 2.121309333<br>C18 -5.587176881 -2.949735632 1.948013357<br>C19 -3.084585913 -3.199359080 1.347050514<br>C20 -2.932869420 -1.688284041 1.555907831<br>C21 -1.797807956 -3.939214676 1.731681485<br>C22 -7.345881746 -2.159395051 0.411715556<br>C23 -8.598637737 -2.952204029 0.836148937<br>C24 -7.393539647 -1.703033774 -1.065820343<br>C25 -8.733416720 -1.032029046 -1.393028009<br>C26 -6.230524190 -0.752536605 -1.377485252<br>C27 -9.870710336 -5.018516283 0.746921386<br>C28 -10.141138558 -5.030114271 2.239057499<br>C29 1.315134016 -9.406454827 9.524388019<br>C30 1.415666710 -7.878769451 9.557386172<br>C31 0.082176137 -5.797649290 9.187657354<br>C32 -0.850793152 -5.436689110 10.359816133<br>C33 -0.506616317 -5.345588569 7.839135778<br>C34 -0.747512888 -3.835223781 7.749027637<br>C35 -1.058489684 -3.413565390 6.313140232<br>C36 -2.138751339 -1.293579718 5.527170177<br>C37 -1.172445392 -3.987240964 12.308183764<br>C38 -1.778987562 -2.609261939 12.153510218<br>C39 2.361601007 -3.285377855 -4.462928584<br>C40 1.398636783 -2.334706068 -5.194069552

	C41	2.397146094	-2.857301253	-2.976049354
	C42	3.063774261	-3.846884128	-1.997046088
	C43	4.565380288	-3.646443571	-1.796740718
	C44	1.303792449	-0.339514176	-6.621220621
	C45	0.466701093	-0.725431007	-7.848028392
	C46	2.227674404	0.850947351	-6.949770375
	C47	-0.481260141	-2.567116327	-9.157815433
	C48	-1.830932906	-2.936480188	-8.520841314
	C49	-3.094530937	-3.273525354	-6.435021929
	C50	-3.754361833	-2.122249138	-5.674433948
	C51	-3.602828390	0.149575421	-4.768477801
	C52	-4.791526257	0.795079390	-5.524670199
	C53	-2.589446231	1.287962135	-4.566649236
	C54	-1.345660456	0.991675576	-3.791339575
	C55	-1.008033406	0.063529311	-2.836399236
	C56	0.721569441	1.440409692	-3.076330768
	C57	-5.522083821	1.576893286	-7.757382244
	C58	-6.954858522	1.067135693	-7.629284350
	C59	-5.000977489	1.392068926	-9.202307399
	C60	-5.429963861	2.425312482	-10.202453436
	C61	-6.651810026	3.037621187	-10.405834702
	C62	-5.165592781	3.768861066	-11.878772778
	C63	-4.057394417	2.441138807	8.579426402
	C64	-3.393495170	1.783808712	9.775750965
	C65	-3.578403751	1.667111758	7.324763232
	C66	-4.185639471	2.185846049	6.041299757
	C67	-5.407581240	1.688362074	5.554336979
	C68	-3.545287834	3.196346584	5.301447136
	C69	-5.972074344	2.177143533	4.370918046
	C70	-4.100915493	3.700445865	4.124729664
	C71	-5.315242331	3.189685762	3.660785670
	C72	-1.936359071	8.439203370	3.011565174
	C73	-2.111870088	9.101559413	1.638595856
	C74	-1.632005545	6.943762809	2.842305161
	C75	-1.139913094	10.612058020	-0.037025172
	C76	-0.861129312	9.728067797	-1.255162651
	C77	-0.020196855	7.553490568	-2.069782172
	C78	-1.232883385	6.632090706	-2.331035395
	C79	1.263652294	6.778383511	-1.720368900
	C80	1.772374044	5.749433564	-2.734100567
	C81	2.333454222	6.290455437	-4.060583032
	C82	1.024968484	7.366044294	-5.896020388
	C83	-3.152449200	5.419268067	-1.431571412
	C84	-4.415537625	6.204518588	-1.830950107
	C85	-3.491118404	4.578739323	-0.192457646
	C86	-5.774884078	8.174532585	-1.336775748
	C87	-6.117496729	9.182860927	-2.429443369
	C88	-5.417643580	10.317279970	-4.495579915
	C89	-6.288405406	9.883035620	-5.694246455
	C90	-4.057571602	10.868077010	-4.977187135
	C91	-3.214094274	9.796737453	-5.660952276
	C92	-7.280103909	7.998554872	-6.918299254
	C93	-8.739974057	8.440247702	-6.933697953
	C94	-7.198930796	6.449191917	-6.888033759
	C95	-5.777878866	5.948805440	-7.009773447
	C96	-5.166905040	5.851043143	-8.269688866
	C97	-5.018729587	5.622585263	-5.875187325
	C98	-3.831467648	5.461314461	-8.395431198
	C99	-3.673597546	5.250517576	-5.979483619

	C100	-3.095475087	5.176747423	-7.245282334
	C101	9.860581683	1.924762876	3.536870974
	C102	9.299807565	1.396070668	2.228686040
	C103	7.422564558	1.591836907	0.613416395
	C104	7.274632968	2.756736584	-0.370992480
	C105	6.146160199	4.883755584	-0.808904595
	C106	5.757517171	6.172499931	-0.093309933
	C107	5.152389781	7.334577809	1.973715902
	C108	6.267562810	8.073343234	2.683545022
	C109	9.698105611	-0.111509327	-2.935533981
	C110	8.928672728	0.312945976	-4.191073340
	C111	8.805893962	-1.042063867	-2.083706824
	C112	9.628004839	-1.812286482	-1.034072776
	C113	8.709580015	-2.460865633	-0.023895614
	C114	7.246762107	1.794441991	-5.167577244
	C115	7.240437386	3.308067628	-5.260079672
	C116	5.849766624	1.131786481	-5.244767393
	C117	5.124331828	1.217249164	-3.929907795
	C118	4.432721221	2.383219255	-3.561666641
	C119	5.236667703	0.175548263	-2.992242294
	C120	3.930052754	2.524635787	-2.263899878
	C121	4.729865859	0.317122862	-1.696919980
	C122	4.103123188	1.509545609	-1.320190963
	C123	6.966310835	-0.640517129	8.571095071
	C124	6.148929717	-0.768168075	9.867419864
	C125	6.700709253	-1.793908978	7.591807588
	C126	7.275002816	-1.632824943	6.217514112
	C127	8.604933581	-1.682507969	5.849683533
	C128	7.346185984	-1.533259521	4.033324629
	C129	5.263045891	-2.153425361	11.660172583
	C130	5.746178703	-1.215167055	12.743281731
	C131	3.205859871	-5.762450671	2.696253468
	C132	2.903109657	-5.050065745	3.985328594
	C133	4.181272146	-4.399230740	4.656062124
	C134	5.277562851	-5.488411201	4.839300131
	C135	6.483255152	-4.987367799	5.653794170
	C136	7.390411408	-6.091217642	6.118147544
	C137	3.762020857	-3.954819306	6.089500390
	C138	2.332700116	1.563780296	3.308199416
	Fe139	4.256099899	1.205403520	3.590092045
	Fe140	0.792342045	1.910659322	1.710998958
	Fe141	1.172616471	2.742434338	4.439033739
	Fe142	3.093678073	3.309731694	2.819135471
	Fe143	0.807154486	4.648813368	2.571138438
	Fe144	2.263443212	0.532319795	5.009860759
	Fe145	2.274142408	-0.654589391	2.612980469
	H146	2.980683525	-8.271422466	-3.628539103
	H147	3.745648857	-6.979888804	-4.269744226
	H148	1.215026438	-7.588021611	-7.664103201
	H149	3.296515086	-7.690042518	-6.500601713
	H150	2.597686039	-9.175433114	-5.869565388
	H151	-0.533965905	-5.678495790	-6.549589408
	H152	-0.898370141	-9.238264290	-6.494621168
	H153	-0.868578817	-6.679040922	-7.985032155
	H154	-2.523690401	-10.542107764	-5.475396295
	H155	-0.642792164	-8.822630189	-3.875623089
	H156	-3.686225645	-9.209480589	-5.666873251
	H157	-1.688476035	-4.421447181	-1.350746664
	H158	-0.852217017	-6.413257144	-3.263026978

	H159	-0.247118695	-8.623216853	-1.583643733
	H160	-5.903931432	-4.426441399	-3.486449453
	H161	-3.554878478	-6.079657072	-3.977958405
	H162	-1.881854875	-9.235917871	-1.225260818
	H163	-1.499715275	-4.197937910	-3.103723864
	H164	-5.050187999	-7.823994428	-2.503709006
	H165	-5.831446550	-5.941812614	-4.424325342
	H166	-1.878616367	-5.019373816	1.547206083
	H167	-0.949502323	-3.560463022	1.139964475
	H168	-1.551647506	-3.784619006	2.794222810
	H169	-3.273248583	-3.373352246	0.275281396
	H170	-6.363907349	-9.312282158	-1.339675947
	H171	-5.251110382	-1.222953524	-1.209729974
	H172	-8.010021395	-4.641573262	-0.236173408
	H173	-9.585013594	-1.711638458	-1.245954808
	H174	-2.099773463	-1.309164627	0.944727369
	H175	-2.694210620	-1.447588452	2.601807292
	H176	-5.149267586	2.283357684	8.674630725
	H177	-6.275201807	-0.433617580	-2.429092391
	H178	-8.738468076	-0.705154296	-2.443386704
	H179	-6.291336457	0.148966292	-0.746126155
	H180	-8.892902269	-0.146390442	-0.758136474
	H181	-7.296312701	-2.602398775	-1.701404626
	H182	-7.350344723	-1.271044005	1.060649771
	H183	-5.604049823	-3.312019810	-0.092078534
	H184	-3.837158333	-1.131072409	1.269940112
	H185	-2.562629037	4.126543225	0.190711999
	H186	-4.158713739	3.767950432	-0.524746953
	H187	-4.690588726	4.796138395	1.341496149
	H188	-6.634417210	3.246044521	2.238472127
	H189	-3.580647451	4.468384253	3.549881715
	H190	-2.577369469	3.579083788	5.632115617
	H191	-5.926031517	0.898120779	6.103902294
	H192	-4.715894370	-5.440439711	0.829765133
	H193	-3.630691583	-8.137221276	2.265114241
	H194	-5.336671396	-8.092909555	2.763964142
	H195	-4.301394548	-7.577926953	0.026675085
	H196	-7.647621288	-8.087629535	-1.190895638
	H197	-6.914600913	1.763457076	4.001803813
	H198	-4.088159184	-3.756053529	3.198571465
	H199	-10.773271450	-4.665802782	0.222513100
	H200	-9.683049924	-6.060770709	0.443271465
	H201	-9.243074450	-4.880600971	2.883344242
	H202	-0.554418369	-4.012988208	13.218849245
	H203	6.029165500	-3.726140702	-0.367085333
	H204	3.279919141	-7.404144383	3.707330418
	H205	10.208916267	-2.601355312	-1.532176200
	H206	10.330291807	-1.134544304	-0.527567689
	H207	8.287371975	-1.768539157	-2.728575440
	H208	8.155098922	3.794532823	-4.845850548
	H209	10.530002413	-0.716628004	-3.327089381
	H210	2.893159478	-1.874909102	-2.908605535
	H211	2.916713190	-4.884047088	-2.345458326
	H212	7.952286162	1.815635304	-3.139532743
	H213	7.804804231	1.476882463	-6.071908875
	H214	9.583723639	1.589356311	-1.748531581
	H215	4.409971293	-4.056780801	0.238824116
	H216	8.023942934	-0.437849276	-1.598660999
	H217	10.966967659	0.715190820	-1.553464131

	H218	3.819544924	-4.025234210	-5.727635784
	H219	1.353391465	-2.718185226	-2.657534559
	H220	9.079353905	-0.951486399	1.186203404
	H221	2.557253991	-3.782483515	-1.023092743
	H222	-4.004580585	-0.196876467	-3.804727372
	H223	-1.572615372	-0.755718512	-2.407510066
	H224	-3.835033032	-3.627411931	-7.163940639
	H225	-1.024246646	-2.781435958	-6.610408947
	H226	-3.884338716	0.276569496	-7.282210017
	H227	-7.602398644	2.967497625	-9.888579132
	H228	-7.027473472	-0.033630970	-7.456213107
	H229	-5.257231256	0.374774886	-9.547460380
	H230	-5.504142804	2.641757483	-7.475534128
	H231	-4.758002397	4.333420797	-12.713368525
	H232	-3.133134222	2.101224409	-4.058409596
	H233	-2.299078263	1.681162909	-5.554345850
	H234	-7.162967039	4.502930126	-11.878673395
	H235	-3.901193841	1.437178227	-9.163112272
	H236	-2.020013201	-1.003029506	-5.658887843
	H237	-2.921278367	-4.100040500	-5.728818347
	H238	-0.690986166	-1.841322108	-9.953379328
	H239	1.695935643	1.901713046	-2.934374444
	H240	-0.066218550	-3.468021916	-9.628455680
	H241	-4.146829925	4.382210626	9.232278332
	H242	-3.936984395	4.242608709	7.616916091
	H243	-1.579316086	6.390164135	-0.301556184
	H244	-2.950109469	4.727425085	-2.261790060
	H245	-3.779870771	7.821428256	-0.742199010
	H246	-0.161766183	2.638445520	-4.593266832
	H247	0.699805503	3.218306987	-6.419090828
	H248	-0.657079581	4.038956235	-6.341931338
	H249	2.771137925	1.148656833	-6.042761546
	H250	2.961758820	0.558944610	-7.719671121
	H251	-3.537469528	11.305342233	-4.112659329
	H252	-4.258604250	11.665232433	-5.704856632
	H253	-1.986909532	9.552629689	-3.960552303
	H254	-1.758752093	8.419388461	-5.273884322
	H255	1.435448976	9.304617382	-6.413258117
	H256	3.173867691	5.663504235	-4.396648403
	H257	-3.370077663	5.379865620	-9.382961615
	H258	-5.467766874	5.649654170	-4.878520898
	H259	-0.609619426	6.460311949	-6.771142346
	H260	1.112902407	6.250284149	-0.765231229
	H261	-0.145067538	8.221537111	-0.043020658
	H262	1.002380169	4.989438774	-2.938967548
	H263	1.154533877	-2.635602816	-7.769354823
	H264	-0.395782296	11.419672089	-0.028274094
	H265	3.042083752	-1.567605104	-6.034964986
	H266	2.055159208	7.525734030	-1.544876303
	H267	-3.079328993	5.060811170	-5.084176566
	H268	0.749110791	1.603128853	-7.971907712
	H269	-2.126913045	11.066517012	-0.201961774
	H270	2.677897742	8.527047138	-5.497961415
	H271	-0.107260432	7.940031185	-7.497392965
	H272	4.416026574	-3.303555631	-4.343001149
	H273	1.885347102	-4.282418162	-4.512288460
	H274	-5.739685041	6.083588650	-9.170165103
	H275	0.551884963	0.015652899	-5.895309479
	H276	0.115886731	8.115146339	-3.004168896

	H277	2.735539348	7.300023765	-3.905262331	
	H278	2.590613941	5.221362757	-2.226827273	
	H279	0.855991407	5.424363428	-5.343174340	
	H280	-6.575886017	7.420134973	-1.344104400	
	H281	-4.359642073	8.781249783	-3.409830848	
	H282	-5.973473514	11.143691875	-4.028319423	
	H283	-5.830200886	8.714162531	-0.381299896	
	H284	-6.189776557	7.942183787	-5.070053574	
	H285	9.540527413	2.965210981	3.692151422	
	H286	9.382423749	1.317442743	4.332838595	
	H287	6.630111542	0.300543955	8.111195873	
	H288	11.672696292	2.021523868	4.471637090	
	H289	11.606931761	0.910849878	3.268676844	
	H290	-1.462947964	6.512054963	3.837237515	
	H291	-3.949222924	8.288661370	3.302016980	
	H292	-0.296823403	10.046428022	1.845670899	
	H293	-2.500642016	6.454411361	2.378305050	
	H294	-3.286978000	9.581963435	4.053029081	
	H295	4.369834475	7.058412117	2.696979254	
	H296	-1.093374672	8.914271845	3.537365639	
	H297	-1.447754937	-5.892495290	7.661830873	
	H298	1.062699266	-5.326718568	9.329744651	
	H299	5.631547401	5.274504481	1.778331243	
	H300	-2.677188907	0.548650259	4.834741230	
	H301	7.052109525	5.093673561	-1.393169798	
	H302	4.828652219	-0.497447363	-0.975036957	
	H303	5.344419659	4.685858972	-1.538684619	
	H304	-1.983993610	-3.882688774	5.954596875	
	H305	5.796300577	3.488990489	0.824324090	
	H306	-0.247405951	-3.747949708	5.645314829	
	H307	0.206864645	-5.656989972	7.059139114	
	H308	0.147267509	-3.287324103	8.081024435	
	H309	-1.580591778	-3.531690142	8.403360963	
	H310	5.996232644	0.081320412	-5.536143907	
	H311	-0.507463320	-7.862727300	9.010474258	
	H312	1.914313424	-9.731224257	8.659482604	
	H313	3.731012165	1.662865890	-0.307326398	
	H314	-0.483579914	-10.052387849	10.290308532	
	H315	-0.062571622	-10.800630474	8.895515345	
	H316	-0.290533152	-1.438618552	6.299103695	
	H317	3.409226908	3.432413393	-1.953603362	
	H318	5.729652750	-0.756976003	-3.277543249	
	H319	5.638046735	-5.261788045	9.892086394	
	H320	7.114371571	-2.723398689	8.024050895	
	H321	3.670382600	-4.752685075	8.574065825	
	H322	6.954659819	-5.676251650	9.198399513	
	H323	7.303278899	7.789900142	2.370892224	
	H324	2.690046788	-1.986837476	7.552884232	
	H325	2.428062001	-5.727924681	4.709856642	
	H326	7.685894535	2.644100014	2.420524184	
	H327	4.823637276	-6.350769663	5.352959304	
	H328	9.450586390	-1.668112829	3.880049743	
	H329	7.071932380	-1.504292166	2.986565851	
	H330	1.837563381	-9.771184767	10.424894310	
	H331	-0.986867869	0.393732389	5.221322880	
	H332	1.956903103	-2.414269280	9.785577270	
	H333	1.986747647	-0.646180780	7.895454274	
	H334	0.476454327	-3.933842026	10.922900311	
	H335	-3.647284831	-2.630084956	5.934242783	

	H336	8.502909812	-7.487318409	5.491898696
	H337	7.060743711	-4.280633063	5.039704325
	H338	6.153044763	-4.460127393	6.555492862
	H339	5.618891700	-5.817350172	3.847031524
	H340	6.435511167	1.218328042	0.927954846
	H341	4.328586815	3.196791087	-4.284351096
	H342	5.295350250	1.636710493	-6.049657058
	H343	2.219736968	-4.220211633	3.761426880
	H344	2.704472568	-3.680797691	10.280519394
	H345	3.345851185	-5.755702771	9.717525735
	H346	-4.080960380	-1.369770018	4.843657995
	H347	9.507451288	-1.770798976	6.442963710
	H348	5.618708116	-1.944679019	7.501101841
	H349	7.974828168	0.794660410	0.112737757
	H350	4.706499064	8.030899578	1.244683030
	H351	-3.844256091	0.608836844	7.472724062
	H352	-2.480393325	1.737288369	7.271216228
	H353	8.544424239	0.320828673	9.459064526
	H354	8.722190063	-1.305302310	9.414683273
	H355	5.421596716	-3.180541552	12.025512987
	H356	6.357213581	-2.849779178	9.968952750
	H357	6.818617451	-0.915504159	12.661362363
	H358	4.181475411	-2.018746467	11.502888286
	H359	-2.361582505	2.154384193	9.987489534
	H360	-9.137583245	8.803375696	-5.956463898
	H361	-2.009212405	-4.695616350	12.442976968
	H362	-1.944050949	-2.279784717	11.099254283
	H363	-6.820086592	8.361141083	-7.852405104
	H364	-7.657452618	6.081841431	-5.956491615
	H365	-7.810518403	6.078779885	-7.723804659
	H366	2.258688755	-2.064425775	2.051381671
	H367	-1.502387194	4.716095109	-8.272197210
	H368	-0.109569164	0.843593948	2.288598500
	H369	0.291832462	-1.429264760	2.616651495
	H370	0.040114468	-0.692166839	2.623743016
	H371	0.609965277	-0.656924066	0.450276902
	H372	0.793667240	-0.051809929	-1.630038016
	Mo373	4.177503056	-1.377646759	4.546616952
	N374	3.528788290	-7.965213889	-4.436571678
	N375	0.701066839	-7.375465706	-6.812485081
	N376	-1.666932075	-8.738439015	-6.054551506
	N377	-1.442816622	-8.952355541	-3.258093081
	N378	-1.479132638	-6.185737876	-2.489440685
	N379	-4.108916295	-5.502096313	-3.341120607
	N380	-5.834631167	-7.410998906	-1.992151121
	N381	-5.016856101	-7.689653400	0.751137270
	N382	-4.532764933	-5.179527908	1.796303172
	N383	-6.117498044	-2.905986705	0.691869890
	N384	-8.690471234	-4.239451792	0.412552941
	N385	-0.058785860	-9.898344924	9.373368976
	N386	0.287990524	-7.240402297	9.189148017
	N387	-1.166432303	-1.954242222	6.175895973
	N388	-3.344532559	-1.873468732	5.329372777
	N389	-1.894710243	-0.074977519	5.025717064
	N390	-0.418994783	-4.419614373	11.146439366
	N391	3.690093293	-3.244464879	-5.081087297
	N392	5.024287338	-3.820735792	-0.543037434
	N393	2.016904664	-1.438996959	-6.005103027
	N394	0.495278672	-2.017320692	-8.236623787

	N395	-1.873255166	-2.960879292	-7.156824299
	N396	-3.026732817	-0.970508247	-5.508937753
	N397	-0.243111394	1.828851110	-3.920677541
	N398	0.276294845	0.360900650	-2.425542167
	N399	-4.650948180	0.807961482	-6.875210179
	N400	-4.510276826	2.890356413	-11.128993533
	N401	-6.460897248	3.889775534	-11.472929973
	N402	-3.650050614	3.845799825	8.516108536
	N403	-3.136197269	8.589428847	3.850153198
	N404	-1.112822862	9.942749567	1.248255862
	N405	-0.274686564	8.534909330	-1.018393334
	N406	1.338398724	6.317261777	-5.141190518
	N407	1.724566374	8.516796180	-5.842568629
	N408	-0.075090550	7.327032592	-6.686811295
	N409	-1.970697214	6.258889695	-1.248702180
	N410	-4.484666066	7.503300781	-1.415099480
	N411	-5.244038062	9.287690057	-3.474858751
	N412	-2.300360435	9.180195809	-4.865694603
	N413	-6.578171761	8.561598957	-5.776964637
	N414	11.326492157	1.862379226	3.522627526
	N415	8.177093164	1.996435204	1.792425138
	N416	6.396579438	3.708373064	0.004458729
	N417	5.610265477	6.157435707	1.256891289
	N418	10.293687709	1.041214033	-2.249715899
	N419	8.065959290	1.358835017	-4.051237193
	N420	8.405498180	-0.500749532	8.864213210
	N421	6.502296137	-1.531276909	5.061714882
	N422	8.626476593	-1.618001356	4.472969594
	N423	6.023552389	-2.003185259	10.429363214
	N424	-1.144332617	2.266498791	-0.434962397
	N425	-0.393704816	2.154264359	0.408408422
	O426	0.784674357	-7.128981689	-4.551336670
	O427	-2.639196066	-6.714980856	-5.657591254
	O428	-3.712586834	-9.209448065	-3.145581109
	O429	-2.509143043	-6.887654918	-0.585241802
	O430	-4.123408739	-3.899614521	-1.722299253
	O431	-7.302773514	-5.669780919	-1.805988917
	O432	-7.146767004	-8.381743549	1.253293264
	O433	-4.073014581	-6.035561179	3.881870429
	O434	-6.097667218	-2.359652156	2.911832061
	O435	-9.486040929	-2.433805523	1.534481532
	O436	-11.242602879	-5.254588538	2.717577164
	O437	2.492188079	-7.334995766	9.884221717
	O438	-1.913996214	-6.057641065	10.536882136
	O439	-2.086840207	-1.899888136	13.098090899
	O440	0.157941203	-2.378262524	-5.043497793
	O441	5.325270475	-3.352000465	-2.747783706
	O442	-0.246390894	0.140823211	-8.396731635
	O443	1.464283537	1.982773594	-7.397001328
	O444	-2.790709839	-3.208433556	-9.250833884
	O445	-4.909253031	-2.236016129	-5.256636734
	O446	-5.715995569	1.344783936	-4.921515544
	O447	-7.947604189	1.766178049	-7.739138215
	O448	-3.909290328	0.905940535	10.452739775
	O449	-5.815788937	3.714019444	2.485193192
	O450	-3.110716527	8.898183512	0.924456349
	O451	-1.151993024	10.155495158	-2.391921394
	O452	-1.505893057	6.280541690	-3.491841096
	O453	-5.327368115	5.640080563	-2.450983185

	O454	-4.127805931	5.387795695	0.801335876
	O455	-7.160455055	9.844508687	-2.354299195
	O456	-6.692729695	10.717860787	-6.514513280
	O457	-3.411666170	9.465297566	-6.840521795
	O458	-9.441907663	8.372307446	-7.929281687
	O459	-1.731174542	4.847287070	-7.331679181
	O460	9.857657224	0.434042403	1.639020001
	O461	7.946379039	2.813571776	-1.424847501
	O462	5.582189458	7.189448178	-0.785194694
	O463	6.068432356	8.930261575	3.528964797
	O464	9.065569637	-0.294990652	-5.266532541
	O465	8.099255647	-3.505167501	-0.233292640
	O466	8.537355959	-1.801925778	1.138553546
	O467	6.354418601	3.966434989	-5.785376393
	O468	5.683008670	0.237184563	10.436573322
	O469	5.056118866	-0.866387332	13.690223970
	O470	3.300726237	-5.242026967	1.594627656
	O471	3.402862115	-7.118495216	2.781653124
	O472	7.633570529	-6.370220468	7.289260512
	O473	7.943064008	-6.788491171	5.090753210
	O474	3.949326843	-2.703049295	6.365391028
	O475	3.336805065	-4.801251568	6.908206633
	O476	4.626559732	-3.336040615	3.899939360
	O477	2.008262991	-3.028515688	10.560347966
	O478	1.981106720	-1.597037831	8.122606010
	O479	0.150341161	3.799539529	-5.822035525
	O480	6.608507499	-5.368395483	10.060888546
	O481	3.819326394	-4.900189103	9.553128722
	S482	-0.150552319	6.618016414	1.775123926
	S483	2.128021941	3.814952623	0.825342185
	S484	4.509952488	-0.648045461	2.323582517
	S485	-0.634207764	3.063320028	3.183252249
	S486	1.722990842	0.127430281	0.525700596
	S487	5.321442568	3.069388967	3.017974461
	S488	1.862129424	-1.629448900	4.558040900
	S489	2.225397987	4.729617251	4.391423215
	S490	4.363677239	0.667420563	5.770861460
	S491	0.758799790	1.528247719	6.269839187

2 35 (S=2)

2 35 (S=2)	bm22b2n2x6x6h2n35dtm.car_3			
Fe(139) -2.500	C1	1.329100640	-7.489834987	-5.619776754
Fe(140) 0.138	C2	2.723624652	-8.123794206	-5.659855873
Fe(141) -2.605	C3	-0.588929044	-6.694520173	-6.932518634
Fe(142) 2.786	C4	-1.721069708	-7.377916366	-6.168242993
Fe(143) 3.195	C5	-2.683891102	-9.469221418	-5.349277153
Fe(144) 2.236	C6	-2.670690369	-9.197844676	-3.841345146
Fe(145) 0.179	C7	-1.325533183	-8.583351955	-1.889754277
	C8	-1.832313622	-7.168252033	-1.597090418
	C9	-1.985149580	-4.848346920	-2.332302079
	C10	-3.513782732	-4.732844814	-2.428722232
	C11	-5.572570105	-5.486318648	-3.475296611
	C12	-6.316300838	-6.209906903	-2.345560309
	C13	-6.592030225	-8.291426983	-1.089169967
	C14	-6.284449932	-8.135453608	0.403929316
	C15	-4.596398524	-7.618697807	2.113111066
	C16	-4.395785598	-6.208029416	2.673101587
	C17	-4.317308733	-3.791731822	2.118275432

	C18	-5.600371881	-2.961111045	1.931743222
	C19	-3.089217194	-3.212927214	1.360711263
	C20	-2.930446923	-1.702830974	1.573815843
	C21	-1.809135444	-3.955977926	1.759360904
	C22	-7.353984791	-2.177683318	0.388019046
	C23	-8.603932242	-2.973644784	0.811114547
	C24	-7.401259387	-1.724351468	-1.090153995
	C25	-8.741262533	-1.053721334	-1.419035743
	C26	-6.236103977	-0.776970948	-1.403426260
	C27	-9.876212698	-5.037208408	0.701991685
	C28	-10.189607990	-5.037245568	2.184538096
	C29	1.313195609	-9.395083015	9.552416753
	C30	1.411381210	-7.867736582	9.574556143
	C31	0.075405266	-5.789014329	9.221790802
	C32	-0.848761296	-5.414356530	10.396615463
	C33	-0.517474596	-5.338356434	7.874818347
	C34	-0.726114938	-3.824274491	7.776179635
	C35	-1.062729201	-3.403989677	6.346057745
	C36	-2.100365635	-1.267808643	5.546969587
	C37	-1.147410809	-3.954817026	12.340057913
	C38	-1.779448191	-2.592103458	12.148597949
	C39	2.400757436	-3.297964926	-4.470263764
	C40	1.435410237	-2.352530449	-5.203157943
	C41	2.427001924	-2.872397640	-2.983312437
	C42	3.080875736	-3.869948966	-2.004778547
	C43	4.580456520	-3.672047118	-1.791225398
	C44	1.323576713	-0.345994913	-6.608084544
	C45	0.474444572	-0.730696283	-7.825650746
	C46	2.238924432	0.848135896	-6.944790749
	C47	-0.456920034	-2.563913537	-9.156721688
	C48	-1.807157785	-2.931482521	-8.520762430
	C49	-3.079904847	-3.252592172	-6.439943774
	C50	-3.739235677	-2.109746050	-5.666989019
	C51	-3.598076986	0.176677483	-4.787217530
	C52	-4.793321054	0.816940782	-5.536650958
	C53	-2.580650388	1.312051396	-4.593325443
	C54	-1.342509742	1.005738757	-3.813231329
	C55	-1.009483986	0.057935704	-2.876143264
	C56	0.722619829	1.438115685	-3.084026190
	C57	-5.546676060	1.586391227	-7.762907170
	C58	-6.973700340	1.062408449	-7.618163488
	C59	-5.043235751	1.397234441	-9.214363075
	C60	-5.484838467	2.430093143	-10.210063211
	C61	-6.712923343	3.032516616	-10.405319872
	C62	-5.238246863	3.788441164	-11.877329298
	C63	-4.040903524	2.455547024	8.607194943
	C64	-3.378822301	1.807197313	9.808922399
	C65	-3.556885193	1.675872228	7.357530041
	C66	-4.159113396	2.192790263	6.071698957
	C67	-5.375670283	1.688994413	5.577265227
	C68	-3.523563403	3.212753220	5.341768679
	C69	-5.938290151	2.179031811	4.393559036
	C70	-4.078071133	3.718108318	4.165537786
	C71	-5.287376212	3.202103489	3.694349265
	C72	-1.945775762	8.466888277	3.010680363
	C73	-2.123055238	9.122793963	1.635870760
	C74	-1.657107500	6.967234368	2.844294716
	C75	-1.145028746	10.611056727	-0.057108502
	C76	-0.854996697	9.716024062	-1.262576854

	C77	-0.025641019	7.535145721	-2.057420400
	C78	-1.248166472	6.628747533	-2.320241159
	C79	1.242564024	6.737652567	-1.706162318
	C80	1.731926922	5.711628280	-2.732655088
	C81	2.310598054	6.262031403	-4.047951285
	C82	1.007641617	7.375719984	-5.867591845
	C83	-3.177416720	5.431704007	-1.423967007
	C84	-4.432068288	6.228068480	-1.829043938
	C85	-3.521901030	4.593583515	-0.184986466
	C86	-5.779563578	8.207772679	-1.328636183
	C87	-6.111680701	9.226792498	-2.415171058
	C88	-5.423758676	10.327849899	-4.505784364
	C89	-6.281918902	9.896132059	-5.714220698
	C90	-4.058072256	10.877772132	-4.974180295
	C91	-3.220972603	9.804243143	-5.659128026
	C92	-7.279988635	8.008308950	-6.933889200
	C93	-8.742910341	8.438110696	-6.957598221
	C94	-7.193730321	6.458694019	-6.900403914
	C95	-5.773091885	5.958619591	-7.022710022
	C96	-5.170112562	5.837134448	-8.283887652
	C97	-5.007107857	5.651933579	-5.887276413
	C98	-3.835669266	5.444044594	-8.411351655
	C99	-3.662266791	5.279085562	-5.992739786
	C100	-3.092014973	5.182001527	-7.260264680
	C101	9.841328571	1.907887466	3.570138871
	C102	9.286200352	1.379938287	2.259312422
	C103	7.420078376	1.577639877	0.632226659
	C104	7.267338778	2.738053513	-0.358416479
	C105	6.141221455	4.872918476	-0.782641463
	C106	5.805056869	6.171816161	-0.055986686
	C107	5.154678830	7.324613536	2.002696253
	C108	6.275021748	8.055768104	2.712673368
	C109	9.694749934	-0.123176961	-2.927332536
	C110	8.928930733	0.307854220	-4.183836867
	C111	8.800336853	-1.051814121	-2.075936981
	C112	9.623052568	-1.827894782	-1.030245068
	C113	8.706597054	-2.476855775	-0.018060393
	C114	7.259727169	1.805251628	-5.158985623
	C115	7.255379132	3.320056513	-5.234583576
	C116	5.861385539	1.145052159	-5.246238827
	C117	5.133936769	1.225453859	-3.932312974
	C118	4.428269937	2.383438181	-3.567624771
	C119	5.265162470	0.190210071	-2.989813346
	C120	3.932356569	2.525590230	-2.267116122
	C121	4.765149401	0.332463551	-1.692377451
	C122	4.127820905	1.520039329	-1.318308267
	C123	6.978892174	-0.626190509	8.562749987
	C124	6.161390491	-0.745728982	9.859375797
	C125	6.687162150	-1.767821426	7.578137056
	C126	7.264872967	-1.611607365	6.204419516
	C127	8.595552350	-1.635453539	5.837834906
	C128	7.336120539	-1.551614377	4.017899440
	C129	5.271558327	-2.125603745	11.656880820
	C130	5.754496707	-1.186016924	12.739166082
	C131	3.158374824	-5.757945726	2.701882199
	C132	2.876075408	-5.052910121	4.001182099
	C133	4.163092764	-4.402184806	4.653420500
	C134	5.262343769	-5.491286089	4.825361351
	C135	6.473657593	-4.995643349	5.636432290

	C136	7.378686109	-6.104487412	6.094378490
	C137	3.760790168	-3.952354032	6.091171386
	C138	2.314507968	1.554953201	3.298091773
	Fe139	4.260897302	1.218136575	3.579705419
	Fe140	0.785227347	1.828999346	1.684790681
	Fe141	1.145015325	2.732943121	4.413312825
	Fe142	3.062414008	3.302119106	2.802068529
	Fe143	0.749260459	4.624278253	2.551473575
	Fe144	2.286400164	0.563231423	5.010273371
	Fe145	2.267588182	-0.722551674	2.617061544
	H146	2.970406362	-8.301380414	-3.642361934
	H147	3.741020861	-7.010647450	-4.279635411
	H148	1.223069707	-7.554290921	-7.672894984
	H149	3.284951602	-7.713403859	-6.513684138
	H150	2.575044545	-9.194531196	-5.883713213
	H151	-0.536938938	-5.664760539	-6.552000780
	H152	-0.900279695	-9.224435187	-6.528165574
	H153	-0.864082016	-6.656950104	-7.995596010
	H154	-2.518772574	-10.541107686	-5.517580413
	H155	-0.651727394	-8.829748962	-3.900468869
	H156	-3.688879154	-9.213796054	-5.708759582
	H157	-1.702001871	-4.447980906	-1.349999126
	H158	-0.866106708	-6.433619736	-3.270308927
	H159	-0.267133362	-8.658164010	-1.605601391
	H160	-5.919795590	-4.445997995	-3.481464956
	H161	-3.570890272	-6.090058558	-3.990654209
	H162	-1.903375163	-9.268648713	-1.258797182
	H163	-1.517729455	-4.217356246	-3.103206425
	H164	-5.070384610	-7.856629391	-2.524083965
	H165	-5.847626610	-5.952772666	-4.431581253
	H166	-1.891609387	-5.037316340	1.582945961
	H167	-0.956468842	-3.584268882	1.169565179
	H168	-1.571060094	-3.794838568	2.822658091
	H169	-3.266853075	-3.385988641	0.287116603
	H170	-6.401259920	-9.340800822	-1.358355343
	H171	-5.258561033	-1.250250131	-1.233874447
	H172	-8.009657314	-4.659223419	-0.263529896
	H173	-9.593732608	-1.732423148	-1.270442995
	H174	-2.082395705	-1.328207776	0.979612864
	H175	-2.718219542	-1.463316557	2.625888920
	H176	-5.132778509	2.298204748	8.699917777
	H177	-6.278335024	-0.459995495	-2.455800490
	H178	-8.745733133	-0.729021442	-2.470212632
	H179	-6.293684735	0.125294131	-0.773013164
	H180	-8.901563671	-0.166472901	-0.786265054
	H181	-7.305151230	-2.624508544	-1.724758024
	H182	-7.362190371	-1.288895037	1.035891520
	H183	-5.609531283	-3.332029884	-0.108429862
	H184	-3.823431118	-1.137446568	1.268012919
	H185	-2.596674667	4.126447793	0.188880643
	H186	-4.208157198	3.796984486	-0.513658336
	H187	-4.681740285	4.823466960	1.377342887
	H188	-6.602230803	3.257737744	2.265049355
	H189	-3.562688500	4.493197390	3.597083164
	H190	-2.560149082	3.602877795	5.676851250
	H191	-5.892799529	0.894340161	6.121615502
	H192	-4.724793135	-5.445922877	0.814548403
	H193	-3.641921300	-8.151050488	2.240765051
	H194	-5.346973751	-8.115987079	2.741992290

	H195	-4.318149230	-7.592424021	0.004151335
	H196	-7.666539287	-8.100627009	-1.201937448
	H197	-6.877635967	1.762527710	4.019451228
	H198	-4.118112682	-3.771672053	3.198196649
	H199	-10.765496049	-4.692678489	0.149609016
	H200	-9.682692836	-6.082747790	0.412694431
	H201	-9.307768342	-4.906101739	2.853953784
	H202	-0.518321086	-3.942223319	13.242541869
	H203	6.030955507	-3.757713172	-0.347356117
	H204	3.267995655	-7.404660331	3.703912167
	H205	10.199667374	-2.618087135	-1.532248633
	H206	10.330586573	-1.154677213	-0.524123023
	H207	8.275549443	-1.775202464	-2.719261016
	H208	8.159995491	3.801794143	-4.792296076
	H209	10.525389904	-0.730024799	-3.319506795
	H210	2.928886412	-1.893450328	-2.910258801
	H211	2.936520593	-4.903594009	-2.363919124
	H212	7.950154529	1.804534835	-3.126625433
	H213	7.820535786	1.496424563	-6.064318546
	H214	9.580047895	1.572931442	-1.735217298
	H215	4.403488964	-4.082714207	0.240676046
	H216	8.022501163	-0.443868826	-1.588773564
	H217	10.965165869	0.701492783	-1.541765160
	H218	3.862793988	-4.017739170	-5.741008482
	H219	1.381762818	-2.727599820	-2.672187297
	H220	9.067473233	-0.962991738	1.195091345
	H221	2.564947836	-3.813930685	-1.035512432
	H222	-3.991805877	-0.168393002	-3.819777706
	H223	-1.573398954	-0.774958090	-2.472845604
	H224	-3.818057531	-3.600534234	-7.174213904
	H225	-1.007383055	-2.765829899	-6.606911495
	H226	-3.898470042	0.298545249	-7.303698603
	H227	-7.662461914	2.944206126	-9.889085464
	H228	-7.032773175	-0.040567137	-7.449445519
	H229	-5.304039136	0.378961903	-9.553711502
	H230	-5.534390907	2.653687921	-7.489843884
	H231	-4.837246813	4.365931898	-12.706554064
	H232	-3.117657520	2.132553870	-4.089295966
	H233	-2.285919375	1.699107018	-5.582648934
	H234	-7.242933547	4.503326677	-11.865860993
	H235	-3.943266230	1.440885981	-9.189283507
	H236	-2.036057141	-0.946194575	-5.757793703
	H237	-2.908619732	-4.084094420	-5.739803585
	H238	-0.665925821	-1.838596433	-9.953407213
	H239	1.695936914	1.897604937	-2.929659489
	H240	-0.041935412	-3.466281477	-9.625116970
	H241	-4.130670764	4.396140611	9.256213973
	H242	-3.920721704	4.255752207	7.641164856
	H243	-1.598805288	6.391354740	-0.290262004
	H244	-2.978439923	4.737710864	-2.253563923
	H245	-3.792176576	7.835614500	-0.728391198
	H246	-0.154491325	2.659371810	-4.588506585
	H247	0.695663371	3.211080575	-6.427030928
	H248	-0.660664140	4.036655374	-6.357184409
	H249	2.784642274	1.153457366	-6.041435011
	H250	2.971043285	0.555365828	-7.716121396
	H251	-3.539376488	11.301269623	-4.102137088
	H252	-4.251116038	11.680116902	-5.697921819
	H253	-2.015126306	9.522646698	-3.951309392

	H254	-1.796830337	8.397101949	-5.269846630
	H255	1.381067082	9.341516840	-6.308230686
	H256	3.146113645	5.630114776	-4.385590581
	H257	-3.382705738	5.337745633	-9.400636262
	H258	-5.450897763	5.695080403	-4.888715561
	H259	-0.599756193	6.468875128	-6.791492731
	H260	1.079793193	6.201447172	-0.757529028
	H261	-0.152578388	8.212989156	-0.034147876
	H262	0.942479862	4.975906352	-2.952032331
	H263	1.186844996	-2.632857240	-7.779368044
	H264	-0.407407662	11.424935561	-0.055457362
	H265	3.076120269	-1.557304229	-6.028432832
	H266	2.047722726	7.468349624	-1.521504164
	H267	-3.063756593	5.105824912	-5.096538582
	H268	0.747251348	1.588144964	-7.958929747
	H269	-2.134402834	11.056042218	-0.234935065
	H270	2.625304353	8.561788612	-5.392095073
	H271	-0.103734456	7.972044400	-7.475018140
	H272	4.453895954	-3.317022749	-4.342323124
	H273	1.929867770	-4.297881017	-4.522338969
	H274	-5.748079575	6.052971109	-9.185210642
	H275	0.578448647	0.003424500	-5.872973814
	H276	0.121178906	8.090400713	-2.993675863
	H277	2.725720576	7.263917718	-3.877772732
	H278	2.533216435	5.153841998	-2.231029921
	H279	0.854251452	5.418709449	-5.364029311
	H280	-6.588803587	7.462429239	-1.343072472
	H281	-4.389825169	8.769082965	-3.434931542
	H282	-5.983602693	11.152340212	-4.040781497
	H283	-5.833685735	8.742583879	-0.370479555
	H284	-6.226786944	7.963173023	-5.063671893
	H285	9.530898454	2.952861823	3.716452476
	H286	9.348674813	1.311248474	4.365939610
	H287	6.657321338	0.321427512	8.106369048
	H288	11.642316542	1.966956056	4.526046226
	H289	11.575469012	0.873185476	3.308002941
	H290	-1.491526530	6.536977818	3.840769265
	H291	-3.954265708	8.315006775	3.317117314
	H292	-0.300201705	10.056003007	1.827487427
	H293	-2.531388652	6.484432055	2.383330120
	H294	-3.292815360	9.622836884	4.040653369
	H295	4.375107132	7.050901087	2.730238983
	H296	-1.095519499	8.936807049	3.529493824
	H297	-1.471310209	-5.867253497	7.712085300
	H298	1.060469110	-5.325939584	9.358646808
	H299	5.565324287	5.251043350	1.796181459
	H300	-2.595089480	0.576132284	4.822106720
	H301	7.025730547	5.061851811	-1.404765006
	H302	4.876944349	-0.477706569	-0.967356895
	H303	5.305686359	4.687388768	-1.477662082
	H304	-2.004311689	-3.858246798	6.012188280
	H305	5.811899935	3.481123732	0.857372733
	H306	-0.275194496	-3.754901583	5.657962365
	H307	0.182002581	-5.669497132	7.090156269
	H308	0.188783157	-3.296252718	8.084653665
	H309	-1.535167273	-3.497904718	8.448783578
	H310	6.009714840	0.095909265	-5.540638092
	H311	-0.521723645	-7.856597636	9.060449757
	H312	1.930420016	-9.727967270	8.703916895

	H313	3.762165826	1.675559662	-0.303517064
	H314	-0.483702822	-10.076176386	10.295217246
	H315	-0.055091254	-10.779445862	8.879197618
	H316	-0.262571216	-1.444561961	6.335666632
	H317	3.397513977	3.424979914	-1.956603724
	H318	5.768901411	-0.736697935	-3.273843932
	H319	5.650630465	-5.250819939	9.892003740
	H320	7.072311760	-2.711233930	8.006450567
	H321	3.681396412	-4.741879078	8.581172296
	H322	6.956535640	-5.675992174	9.185899664
	H323	7.308291453	7.733538591	2.432864406
	H324	2.695892295	-1.982346332	7.557586151
	H325	2.417213487	-5.735333709	4.731716319
	H326	7.683272876	2.644603305	2.431199259
	H327	4.812487455	-6.356853703	5.336375239
	H328	9.441512459	-1.628046088	3.868436715
	H329	7.062877182	-1.545900318	2.970712645
	H330	1.816628288	-9.751164632	10.467229377
	H331	-0.919360545	0.405621687	5.260235646
	H332	1.961555189	-2.402913426	9.789259465
	H333	2.000888996	-0.638595982	7.904553376
	H334	0.498112917	-3.924608828	10.950576144
	H335	-3.636142094	-2.574417948	5.937570325
	H336	8.505269772	-7.485691536	5.458943984
	H337	7.050556062	-4.289345299	5.022102325
	H338	6.147935452	-4.470974105	6.541240188
	H339	5.597094712	-5.815127343	3.828948435
	H340	6.433880011	1.201782858	0.947407822
	H341	4.306356538	3.190674620	-4.294775495
	H342	5.310322805	1.653234663	-6.051622558
	H343	2.185441868	-4.224605754	3.794147520
	H344	2.716577300	-3.665358682	10.285037348
	H345	3.355104708	-5.740273066	9.726753709
	H346	-4.040004770	-1.290966215	4.869058748
	H347	9.498975529	-1.683328673	6.434856551
	H348	5.601942505	-1.889097153	7.484476884
	H349	7.974618640	0.779539108	0.135261204
	H350	4.709725938	8.027787440	1.279829814
	H351	-3.827856896	0.619012137	7.505011985
	H352	-2.457807260	1.741167064	7.310708749
	H353	8.580289220	0.305693848	9.445121665
	H354	8.726224473	-1.324150713	9.400075886
	H355	5.426806328	-3.152385214	12.024945481
	H356	6.363442408	-2.827334070	9.966120091
	H357	6.825525637	-0.882971158	12.653789952
	H358	4.190176147	-1.990011194	11.496241124
	H359	-2.340391445	2.168154836	10.005635920
	H360	-9.152765141	8.785550430	-5.979957664
	H361	-1.970428573	-4.671128179	12.506673147
	H362	-1.959539084	-2.298600977	11.085444166
	H363	-6.817155207	8.373563873	-7.865146848
	H364	-7.651299192	6.094179007	-5.967223613
	H365	-7.806165862	6.085236653	-7.734107907
	H366	2.344199391	-2.134990809	2.103040700
	H367	-1.505849182	4.707630994	-8.289903515
	H368	-0.155147512	0.735007809	2.224737337
	H369	0.794555901	-1.276281443	2.503428280
	H370	0.634051623	-0.396605052	2.585598795
	H371	0.698191072	-0.686863362	0.330884489

H372	0.791261999	-0.086751570	-1.674939762
Mo373	4.178575729	-1.380696400	4.526803017
N374	3.518697614	-7.994403362	-4.450237632
N375	0.701344265	-7.360048662	-6.821880952
N376	-1.670527121	-8.730114538	-6.084994837
N377	-1.454106866	-8.966825133	-3.287249287
N378	-1.490192900	-6.208051420	-2.494052537
N379	-4.124724067	-5.523788896	-3.344120528
N380	-5.852394087	-7.441876586	-2.010054069
N381	-5.032105142	-7.707231936	0.729892093
N382	-4.546825579	-5.191977089	1.783762258
N383	-6.123651770	-2.920177576	0.672356103
N384	-8.688951033	-4.261383257	0.389051811
N385	-0.057070301	-9.892712961	9.384799325
N386	0.274984115	-7.232605193	9.228638465
N387	-1.148358226	-1.942620270	6.211515292
N388	-3.311800167	-1.830337140	5.328053219
N389	-1.829085557	-0.054861666	5.049552293
N390	-0.397654554	-4.406153233	11.183318193
N391	3.731168386	-3.247690285	-5.082217254
N392	5.028777309	-3.856681663	-0.535033420
N393	2.049046248	-1.444845233	-6.005213215
N394	0.519875668	-2.014681546	-8.235509954
N395	-1.857760309	-2.932535314	-7.156574586
N396	-3.026332007	-0.947835782	-5.523694348
N397	-0.239371347	1.844353176	-3.922367470
N398	0.273725384	0.346698067	-2.455595430
N399	-4.660026014	0.829436375	-6.887680447
N400	-4.572136755	2.911722553	-11.135140438
N401	-6.533792173	3.893809836	-11.467075800
N402	-3.634042992	3.859652889	8.540458183
N403	-3.139127677	8.627835299	3.854780332
N404	-1.121103085	9.956423545	1.236161493
N405	-0.273262567	8.523619055	-1.011922774
N406	1.327749578	6.310685912	-5.138323728
N407	1.686841104	8.534984281	-5.773641852
N408	-0.078497654	7.340457376	-6.678207769
N409	-1.989573586	6.261992611	-1.238692319
N410	-4.495783643	7.524524393	-1.405699504
N411	-5.262224661	9.297745109	-3.483578468
N412	-2.336276273	9.156046569	-4.855805667
N413	-6.584804297	8.576936742	-5.791011551
N414	11.305534169	1.825385246	3.571105872
N415	8.171635782	1.987029001	1.811647216
N416	6.398383515	3.694567484	0.024058072
N417	5.600990405	6.142019803	1.286516929
N418	10.291349135	1.027661612	-2.237730271
N419	8.072586147	1.358164360	-4.042622526
N420	8.422098625	-0.513415162	8.851129574
N421	6.491410986	-1.548492240	5.045954962
N422	8.616277340	-1.596780003	4.460552871
N423	6.035101462	-1.978665117	10.426656897
N424	-1.109435000	2.260393295	-0.489011396
N425	-0.377017451	2.105330142	0.362647220
O426	0.771943147	-7.160849871	-4.555320968
O427	-2.642983826	-6.709613918	-5.672007929
O428	-3.723537727	-9.221008609	-3.183166843
O429	-2.524066255	-6.922596048	-0.597139341
O430	-4.142359309	-3.942643360	-1.705864349

	O431	-7.316638640	-5.700992698	-1.811120395
	O432	-7.150000777	-8.429042896	1.239310247
	O433	-4.094978553	-6.057142864	3.865853433
	O434	-6.116902582	-2.367999742	2.890354532
	O435	-9.495873494	-2.458718612	1.506425567
	O436	-11.309033622	-5.238648355	2.631330209
	O437	2.492560265	-7.320853640	9.879896508
	O438	-1.922565817	-6.016437662	10.573697635
	O439	-2.095474291	-1.860737382	13.073349918
	O440	0.195116302	-2.407743757	-5.058537980
	O441	5.346792613	-3.370474013	-2.734409058
	O442	-0.261574812	0.131591309	-8.350537069
	O443	1.468535472	1.973936326	-7.395627570
	O444	-2.761016480	-3.217677667	-9.253287265
	O445	-4.880389033	-2.241727889	-5.217852078
	O446	-5.719953313	1.359844850	-4.930962126
	O447	-7.974480810	1.751893232	-7.714360160
	O448	-3.899947959	0.946748989	10.503760777
	O449	-5.790847607	3.732958636	2.522722842
	O450	-3.126009886	8.920755545	0.927456779
	O451	-1.137176913	10.133008080	-2.405664966
	O452	-1.523639420	6.280011571	-3.481509214
	O453	-5.341637455	5.675491453	-2.463319627
	O454	-4.135221217	5.411223127	0.816134332
	O455	-7.129708635	9.922944921	-2.314207483
	O456	-6.664258036	10.729050047	-6.546539518
	O457	-3.402286759	9.493821724	-6.847033696
	O458	-9.435514645	8.376863338	-7.959991854
	O459	-1.728678655	4.849348951	-7.349493576
	O460	9.842672266	0.411913070	1.677695388
	O461	7.924351205	2.781813153	-1.422355277
	O462	5.703491265	7.207679048	-0.734945098
	O463	6.083495149	8.948961259	3.522130421
	O464	9.067224470	-0.295949185	-5.261534383
	O465	8.103676497	-3.526459229	-0.222835126
	O466	8.528987353	-1.815598339	1.142152832
	O467	6.381386631	3.984547392	-5.772313483
	O468	5.695822300	0.262413395	10.423407893
	O469	5.066036297	-0.838439683	13.687647823
	O470	3.226763408	-5.232981153	1.600154618
	O471	3.369020641	-7.112654404	2.777668363
	O472	7.606883722	-6.402453212	7.264011210
	O473	7.945327181	-6.783902306	5.063064275
	O474	3.946024232	-2.699447573	6.359503660
	O475	3.348162771	-4.797705839	6.917521857
	O476	4.601608673	-3.342008679	3.888238960
	O477	2.018146410	-3.015359993	10.565313226
	O478	1.985365943	-1.590766048	8.123961248
	O479	0.144369315	3.794828872	-5.834046815
	O480	6.622761578	-5.358251148	10.049606913
	O481	3.828345133	-4.885031626	9.561237360
	S482	-0.183537950	6.620107139	1.774397366
	S483	2.085459951	3.785244460	0.806713982
	S484	4.543294200	-0.643395146	2.322458653
	S485	-0.661209657	2.995128217	3.139667120
	S486	1.815127401	0.080830989	0.480674137
	S487	5.299501073	3.100793620	3.016261337
	S488	1.857002334	-1.597048372	4.613127730
	S489	2.168675371	4.726183518	4.363735369

	S490 4.391252424 0.664961571 5.758397986<br>S491 0.788757865 1.560416901 6.278286929

TS 2 → 3 35 (S=2)

2 → 3 TS 35 (S=2)	bm22b2n2x6x2hncnx6h235to_1_53225.622
Fe(139) -2.507<br>Fe(140) 0.105<br>Fe(141) -2.608<br>Fe(142) 2.818<br>Fe(143) 3.214<br>Fe(144) 2.293<br>Fe(145) 0.141	C1 -3.585841373 -5.838312052 -6.526441888<br>C2 -2.749389861 -7.050633388 -6.944745697<br>C3 -4.958637296 -3.948734648 -7.278105131<br>C4 -6.170541107 -4.110644018 -6.362500620<br>C5 -7.965833659 -5.561487696 -5.559712816<br>C6 -7.603226953 -5.654680848 -4.073966981<br>C7 -5.869938259 -6.185063290 -2.426072481<br>C8 -5.519216313 -4.797231578 -1.883169231<br>C9 -4.538195863 -2.610254607 -2.321744702<br>C10 -5.780897086 -1.744229754 -2.061557959<br>C11 -8.047206373 -1.156301863 -2.715709577<br>C12 -8.905897296 -1.623529631 -1.532561161<br>C13 -10.053444861 -3.506078733 -0.466598763<br>C14 -9.513645941 -3.824722993 0.932055547<br>C15 -7.589619846 -4.566306174 2.272365027<br>C16 -6.596434805 -3.594806881 2.912985909<br>C17 -5.331109547 -1.483364666 2.610933921<br>C18 -5.995634194 -0.108565110 2.799058590<br>C19 -4.092746774 -1.452806493 1.673089600<br>C20 -3.126138224 -0.316814200 2.029054658<br>C21 -3.368714850 -2.804178090 1.690484099<br>C22 -7.258932114 1.739988363 1.767945881<br>C23 -8.671199563 1.602498602 2.371226617<br>C24 -7.256654789 2.448031660 0.391621574<br>C25 -8.085972450 3.736820365 0.431795174<br>C26 -5.819731164 2.750223188 -0.054408336<br>C27 -10.841042175 0.515695509 2.332607477<br>C28 -10.902338907 0.332955106 3.836121183<br>C29 -2.560328001 -10.516580448 8.018986936<br>C30 -1.664482014 -9.284927885 8.173264430<br>C31 -1.731839859 -6.791704404 8.299521405<br>C32 -2.165886207 -6.270043806 9.683794485<br>C33 -2.168116778 -5.836122890 7.174368679<br>C34 -1.577736189 -4.430434574 7.308602748<br>C35 -1.823997280 -3.605910646 6.046129426<br>C36 -1.640446350 -1.129763196 5.717403169<br>C37 -1.405069847 -5.284089503 11.793873313<br>C38 -1.231424936 -3.784336835 11.914038724<br>C39 -0.339608891 -3.053374359 -5.209933293<br>C40 -0.744482688 -1.623813933 -5.604449640<br>C41 0.112906302 -3.010949897 -3.730317370<br>C42 0.268820537 -4.373402179 -3.022198367<br>C43 1.653396234 -5.008027979 -3.140504821<br>C44 0.039066068 0.406385055 -6.740723291<br>C45 -1.033413240 0.753306158 -7.782797690<br>C46 1.397320335 1.019612882 -7.138076966<br>C47 -2.977893150 -0.048156120 -9.040492005<br>C48 -4.215009682 0.200686993 -8.162945413<br>C49 -5.183327985 0.101690860 -5.902622880<br>C50 -5.025250531 1.233891353 -4.886470933<br>C51 -3.571711696 2.904848731 -3.826423897<br>C52 -4.338534091 4.193249851 -4.220250812<br>C53 -2.093591247 3.319076998 -3.758710024

	C54	-1.099463472	2.303094057	-3.291043848
	C55	-1.174245886	1.152307758	-2.545388571
	C56	0.973037367	1.529996436	-2.982571397
	C57	-4.863108940	5.674257636	-6.131848689
	C58	-6.310787913	5.946790119	-5.730393458
	C59	-4.735705464	5.556918845	-7.668781086
	C60	-4.693427209	6.845566053	-8.436457705
	C61	-5.435514520	8.004253630	-8.306186812
	C62	-3.992698922	8.194935076	-9.977216855
	C63	-0.939153642	2.311775714	9.488611994
	C64	-0.555015144	1.176415182	10.420273680
	C65	-1.122710276	1.685962441	8.082924473
	C66	-1.536638690	2.692041982	7.034530828
	C67	-2.890620774	2.968257251	6.771632389
	C68	-0.566423113	3.389425200	6.292109759
	C69	-3.268060126	3.905250268	5.802203854
	C70	-0.926683439	4.335782394	5.331530838
	C71	-2.278278282	4.589663426	5.085556104
	C72	3.188746904	7.496846056	4.228534298
	C73	3.226363137	8.413124035	2.999305906
	C74	2.609230559	6.125792230	3.846981001
	C75	4.638775008	9.527604129	1.326669276
	C76	4.241495369	8.888112989	-0.004993612
	C77	3.671451618	6.802156221	-1.197116912
	C78	2.133806555	6.689049187	-1.284966357
	C79	4.375533582	5.432443899	-1.219751216
	C80	4.116097331	4.529453446	-2.429661388
	C81	4.712068774	4.983488798	-3.774212407
	C82	3.948957035	6.925207111	-5.147245864
	C83	0.001474759	6.431448805	-0.126449884
	C84	-0.694108049	7.806875527	-0.143932273
	C85	-0.562369327	5.629493965	1.056102956
	C86	-0.709763434	10.045398785	0.837850765
	C87	-0.617852679	11.289650484	-0.042036189
	C88	0.279122983	12.314395150	-2.092933501
	C89	-0.836676828	12.623744909	-3.113755993
	C90	1.648039455	12.192965258	-2.798485475
	C91	1.690465682	10.994428459	-3.739079449
	C92	-2.832354356	11.776631817	-4.272985929
	C93	-3.832141957	12.873945898	-3.923159874
	C94	-3.568951824	10.418195668	-4.421883421
	C95	-2.654016173	9.317498221	-4.907102878
	C96	-2.378529699	9.177130875	-6.276296989
	C97	-2.019592144	8.443657974	-4.008701446
	C98	-1.477662884	8.216564683	-6.739869402
	C99	-1.098903092	7.488168757	-4.451728550
	C100	-0.835121867	7.390530131	-5.815912924
	C101	9.750388002	-4.011080912	1.463806563
	C102	8.825387905	-3.911341202	0.263297568
	C103	7.151088364	-2.477921296	-0.882821151
	C104	7.520212016	-1.221257090	-1.680358368
	C105	7.674595826	1.220556448	-1.614656733
	C106	8.114991086	2.351197717	-0.690576588
	C107	8.449080349	3.219565393	1.572453203
	C108	9.896629489	3.173586496	2.021985424
	C109	7.690390207	-4.311520966	-5.004140240
	C110	7.107522729	-3.309513652	-6.005739929
	C111	6.559989694	-4.834260968	-4.088609048
	C112	6.991226371	-6.100724568	-3.326056207

	C113	6.008269474	-6.407374360	-2.219160246
	C114	6.361009458	-1.018656061	-6.422076334
	C115	7.151938437	0.272012628	-6.338683760
	C116	4.828506094	-0.854564174	-6.275270541
	C117	4.431611790	-0.697241155	-4.832668892
	C118	4.539980193	0.547398078	-4.189935777
	C119	4.091301744	-1.826842672	-4.068934608
	C120	4.382362071	0.637744132	-2.803193072
	C121	3.931637831	-1.733718278	-2.682838214
	C122	4.107556119	-0.502281098	-2.044958744
	C123	6.674604174	-5.756036570	6.696389132
	C124	6.093869654	-5.707520528	8.119751498
	C125	5.691446900	-6.371092504	5.689540966
	C126	6.074079439	-6.266406430	4.244895045
	C127	7.109738801	-6.912188839	3.601346247
	C128	5.894856321	-5.811379009	2.112801362
	C129	4.868940956	-6.794701428	9.918515453
	C130	5.907786497	-6.450772083	10.963960596
	C131	-0.014492738	-6.995433029	1.321887030
	C132	0.286623820	-6.509777514	2.713712113
	C133	1.789240720	-6.745004745	3.146418602
	C134	2.154588400	-8.249289367	2.978935667
	C135	3.543027037	-8.581350814	3.552816902
	C136	3.806273533	-10.055260030	3.678561884
	C137	1.872349506	-6.437427229	4.672402416
	C138	3.211552166	-0.519876373	2.844645722
	Fe139	4.678140650	-1.839177899	2.613039943
	Fe140	1.787360103	0.736890226	1.707704758
	Fe141	3.001016119	0.820812006	4.315135203
	Fe142	4.666152774	0.694187033	2.361042541
	Fe143	3.347762056	3.001892345	2.790174929
	Fe144	2.896533527	-1.700231150	4.403188125
	Fe145	1.893953357	-2.306230427	1.932749319
	H146	-2.361746984	-7.731415717	-5.061476463
	H147	-1.118774212	-6.902926458	-5.720028892
	H148	-4.023681435	-5.436214066	-8.497637632
	H149	-2.173988966	-6.794525829	-7.848121676
	H150	-3.460513665	-7.837585448	-7.251738819
	H151	-4.313727165	-3.198335316	-6.798316556
	H152	-6.496336892	-6.009762296	-7.068202881
	H153	-5.298952870	-3.542353388	-8.240059567
	H154	-8.426303296	-6.508017179	-5.873192690
	H155	-5.713938229	-6.303053921	-4.551181794
	H156	-8.714927765	-4.764945281	-5.656303363
	H157	-3.957128871	-2.611089197	-1.390104791
	H158	-4.542185298	-4.313367770	-3.641144927
	H159	-4.977241559	-6.824991533	-2.399175538
	H160	-7.799394479	-0.101532941	-2.542160979
	H161	-6.738916708	-2.545116734	-3.713199705
	H162	-6.623080386	-6.614099406	-1.754633033
	H163	-3.925738061	-2.145129543	-3.107676342
	H164	-8.733284319	-3.596461280	-2.140315858
	H165	-8.646020946	-1.228000191	-3.633696116
	H166	-4.029631203	-3.626016566	1.381081751
	H167	-2.508761153	-2.784907901	1.002971032
	H168	-2.979811509	-3.027959770	2.696441821
	H169	-4.466712812	-1.273925613	0.651902921
	H170	-10.468486677	-4.438055084	-0.877750306
	H171	-5.213316016	1.838538761	-0.146191833

	H172	-9.203392023	0.101187759	1.025979668
	H173	-9.137449671	3.551740678	0.696616187
	H174	-2.303836294	-0.280899244	1.298255885
	H175	-2.670009066	-0.476056134	3.016775616
	H176	-1.921594218	2.699787790	9.820142479
	H177	-5.825889679	3.254883620	-1.031551010
	H178	-8.065116073	4.225285123	-0.553418422
	H179	-5.327788353	3.417739068	0.671548960
	H180	-7.671709217	4.442067352	1.169590398
	H181	-7.718469769	1.763833943	-0.343344851
	H182	-6.713840586	2.364541405	2.491175109
	H183	-6.459806267	0.005609295	0.780840388
	H184	-3.614249203	0.669374326	2.030094140
	H185	0.025918203	4.704775098	1.169892946
	H186	-1.597278808	5.354182626	0.796762135
	H187	-1.234869389	6.082933366	2.841719562
	H188	-3.548638800	5.576987705	3.997911035
	H189	-0.161866047	4.845813365	4.744205290
	H190	0.491959997	3.167232132	6.443933995
	H191	-3.670718742	2.443133365	7.329977218
	H192	-6.724972238	-2.404333342	1.265586493
	H193	-7.058377093	-5.518283126	2.124312852
	H194	-8.396074227	-4.736993288	2.997725593
	H195	-7.626285338	-4.244255120	0.170781645
	H196	-10.880495783	-2.798414948	-0.324028766
	H197	-4.325624467	4.092098719	5.597336650
	H198	-5.012820178	-1.784767271	3.617675065
	H199	-11.468377824	1.376875057	2.049493852
	H200	-11.278020653	-0.386767514	1.876376244
	H201	-9.992883276	-0.125099642	4.291581998
	H202	-0.763408088	-5.787988531	12.532885705
	H203	3.029323197	-6.095249882	-2.080669054
	H204	-0.657044592	-8.633269847	2.115391642
	H205	7.010977497	-6.954826448	-4.017885033
	H206	8.000286779	-5.968285153	-2.909129550
	H207	5.666107421	-5.067022976	-4.687884711
	H208	8.224203481	0.133770183	-6.063485622
	H209	8.018726906	-5.152030133	-5.634767513
	H210	1.059768297	-2.449839567	-3.672616281
	H211	-0.455942852	-5.098498870	-3.430981138
	H212	7.212467850	-1.777980360	-4.604674717
	H213	6.547865523	-1.376205320	-7.455036200
	H214	8.643871897	-3.059740678	-3.647733313
	H215	1.589357898	-5.661949246	-1.165733649
	H216	6.269066792	-4.035047862	-3.389456518
	H217	9.368004976	-4.527445302	-3.839152408
	H218	0.322754137	-4.132379986	-6.841747281
	H219	-0.642568706	-2.429049982	-3.181890326
	H220	7.261528444	-5.559863881	-0.956023741
	H221	0.012218620	-4.253013141	-1.959958750
	H222	-3.945891124	2.614777935	-2.834161563
	H223	-2.024002691	0.638006881	-2.114413986
	H224	-6.085731315	0.325732095	-6.485577211
	H225	-3.215978628	-0.497782420	-6.475910581
	H226	-4.116320499	3.667204411	-6.185307916
	H227	-6.208712505	8.305983121	-7.608971425
	H228	-6.946982563	5.030437896	-5.677050767
	H229	-5.537993999	4.897164552	-8.044210793
	H230	-4.235999635	6.500006687	-5.759160060

	H231	-3.462862811	8.643267235	-10.812752346
	H232	-2.045922638	4.188564107	-3.082045785
	H233	-1.782773519	3.683201567	-4.751526097
	H234	-5.304440678	9.794674465	-9.468140659
	H235	-3.785266271	5.041751985	-7.879027169
	H236	-2.990587572	1.366674524	-5.218613333
	H237	-5.379075458	-0.829649824	-5.350459670
	H238	-2.866863322	0.825390115	-9.695147601
	H239	2.053662759	1.413341510	-3.000829107
	H240	-3.174502086	-0.921132152	-9.676694596
	H241	0.102391690	3.851647635	10.349419731
	H242	-0.026684533	3.977742413	8.725288755
	H243	1.980728187	6.223858324	0.731041801
	H244	-0.305448451	5.928257152	-1.054957105
	H245	0.851020795	8.628323442	0.923957351
	H246	0.654872284	3.285696606	-4.139244073
	H247	1.430216483	3.679431998	-6.043129174
	H248	0.730943824	5.036494070	-5.601041862
	H249	2.132279724	0.811422498	-6.348583522
	H250	1.753047100	0.570440777	-8.080501211
	H251	2.431429514	12.133261150	-2.029118261
	H252	1.801768588	13.104125981	-3.390374211
	H253	2.821913517	9.846209942	-2.374871873
	H254	2.210047606	9.020400471	-3.788637139
	H255	5.246588821	8.467120405	-5.507383569
	H256	5.050069199	4.110018340	-4.352256351
	H257	-1.280269167	8.112624938	-7.809200276
	H258	-2.232989470	8.491661826	-2.936890342
	H259	1.992706427	7.146519950	-5.770825981
	H260	4.085907961	4.867193474	-0.319731729
	H261	4.174702339	7.020625322	0.868449039
	H262	3.039553735	4.330979816	-2.545281427
	H263	-1.464030462	-1.212145068	-8.058743277
	H264	5.694368676	9.823792632	1.257408809
	H265	0.934653341	-1.610167398	-6.693083196
	H266	5.458906752	5.621038420	-1.141081369
	H267	-0.566717028	6.861430989	-3.733125224
	H268	0.413304637	2.596827066	-7.718174436
	H269	4.038318427	10.442991455	1.419158415
	H270	5.996399125	7.010100115	-4.960195904
	H271	3.103202800	8.318556500	-6.380620626
	H272	1.380400059	-4.128349801	-5.548570223
	H273	-1.269895190	-3.649224824	-5.258213663
	H274	-2.873719867	9.828408432	-7.001091087
	H275	-0.303779638	0.926621215	-5.830048098
	H276	3.962927295	7.396156781	-2.074060903
	H277	5.597676021	5.604573674	-3.591803544
	H278	4.575267690	3.564513470	-2.178193808
	H279	2.857229658	5.252286017	-4.804512275
	H280	-1.780441030	9.814935847	0.942473235
	H281	0.488807832	10.270744146	-1.437784088
	H282	0.310674765	13.194374840	-1.433470307
	H283	-0.330348730	10.335562485	1.826949699
	H284	-1.705489174	10.821682897	-2.715822281
	H285	10.056749841	-3.004877007	1.784096244
	H286	9.135715030	-4.436791355	2.284237670
	H287	6.852428410	-4.705849902	6.420937282
	H288	11.430315099	-5.040455291	1.988939338
	H289	10.629844251	-5.688067419	0.715063896

	H290	2.649799204	5.478947969	4.733139503
	H291	1.468092680	8.335880767	4.929284040
	H292	5.275103661	8.260075995	2.928389997
	H293	1.560723045	6.253227186	3.539738314
	H294	2.811963598	8.937104212	5.641001794
	H295	7.793277829	3.177294607	2.456016281
	H296	4.213382217	7.354882557	4.605785729
	H297	-3.269598528	-5.790265621	7.151458917
	H298	-0.641974881	-6.911175065	8.267170368
	H299	7.695829582	1.298842058	1.058595982
	H300	-1.154099801	0.808932582	5.295998978
	H301	8.483528591	1.065519850	-2.341824439
	H302	3.674102916	-2.617576547	-2.095062461
	H303	6.814381746	1.603742608	-2.187774686
	H304	-2.895911225	-3.437666023	5.886875815
	H305	6.858280023	-0.118927434	-0.100418023
	H306	-1.451161033	-4.154940353	5.165233846
	H307	-1.837320693	-6.288763721	6.225608388
	H308	-0.491153573	-4.490816860	7.472797231
	H309	-2.010383539	-3.906156465	8.175268137
	H310	4.356274847	-1.746308309	-6.712028911
	H311	-3.337706003	-8.201005754	8.022397159
	H312	-2.330036596	-10.942855931	7.030344412
	H313	4.024376009	-0.407658581	-0.963094497
	H314	-4.328660351	-10.290015907	9.051147823
	H315	-4.528005354	-10.881523297	7.536832772
	H316	-0.104205633	-2.373090830	6.068891901
	H317	4.477456349	1.591847042	-2.282106803
	H318	3.962857840	-2.793956357	-4.561262852
	H319	3.298184092	-9.253701233	7.804152144
	H320	5.565768515	-7.441912214	5.929917128
	H321	1.721010993	-7.573855718	7.020001489
	H322	4.090354206	-10.117941772	6.797823085
	H323	10.532278202	2.416145631	1.502695852
	H324	2.222033140	-4.545705514	6.521018940
	H325	-0.373034382	-6.989648808	3.451999958
	H326	8.152056943	-2.092995968	0.932288739
	H327	1.392518878	-8.855150635	3.494404111
	H328	7.568962259	-6.951358758	1.508047449
	H329	5.547685614	-5.446975075	1.154508262
	H330	-2.213078481	-11.249507454	8.767231426
	H331	0.206383502	-0.275304205	5.347182010
	H332	1.703029231	-4.997261240	8.811211781
	H333	2.408815470	-3.142548159	7.159172091
	H334	-0.184427685	-5.769882088	10.087372218
	H335	-3.573637328	-1.514628041	6.296957498
	H336	3.949093030	-11.648714598	2.667392714
	H337	4.310377671	-8.131641751	2.905754133
	H338	3.661004994	-8.160061710	4.557368491
	H339	2.134212136	-8.501589404	1.908267976
	H340	6.162627431	-2.358533231	-0.411162322
	H341	4.792405309	1.433135061	-4.778030395
	H342	4.536949642	0.017870532	-6.879920769
	H343	0.127436131	-5.423608971	2.736165703
	H344	1.730039034	-6.536820541	8.997981216
	H345	1.089024189	-8.492902514	8.106105307
	H346	-3.354748443	-0.025152987	5.464596878
	H347	7.900372610	-7.548832829	3.980991811
	H348	4.707656676	-5.905551700	5.821910132

	H349	7.129930499	-3.323224065	-1.573453883
	H350	8.283672656	4.190629955	1.078997645
	H351	-1.881825643	0.894031779	8.170902534
	H352	-0.171826989	1.213052048	7.789051515
	H353	8.625235181	-5.957903215	7.300705704
	H354	7.871548085	-7.390837397	7.063818411
	H355	4.511767282	-7.813215104	10.136403987
	H356	5.208184560	-7.600367750	7.975782034
	H357	6.957578766	-6.714381957	10.687822527
	H358	4.008411993	-6.112401769	10.010660582
	H359	0.537484089	0.943187286	10.427114041
	H360	-3.845263713	13.177777472	-2.849498584
	H361	-2.456914870	-5.506847975	12.046541192
	H362	-1.400370739	-3.212220312	10.968593085
	H363	-2.377946467	12.048772136	-5.239535743
	H364	-4.017999682	10.144000642	-3.454263132
	H365	-4.394905096	10.573926213	-5.131264614
	H366	1.137501164	-3.391190747	1.225163356
	H367	0.121590126	6.422907269	-7.213942216
	H368	0.549177669	0.076494581	2.415144301
	H369	0.386394766	-1.985008673	2.074680241
	H370	0.732431799	-1.062105072	2.290407813
	H371	0.276261883	-1.065899406	0.070251109
	H372	0.444476283	-0.078330434	-1.779832160
	Mo373	3.383809651	-4.181883853	3.305601350
	N374	-1.850434822	-7.584762770	-5.935728944
	N375	-4.225026372	-5.179233802	-7.534367985
	N376	-6.826951623	-5.295287417	-6.424362933
	N377	-6.376407432	-6.159187640	-3.790035364
	N378	-4.854025905	-3.970833572	-2.731650312
	N379	-6.827728614	-1.927341911	-2.902738628
	N380	-9.110701637	-2.962632251	-1.431114555
	N381	-8.189724598	-4.137874844	1.019883679
	N382	-6.306787315	-2.481036951	2.190027721
	N383	-6.574749234	0.446789482	1.695244900
	N384	-9.475823323	0.640757044	1.850695881
	N385	-3.993528710	-10.209496357	8.089047749
	N386	-2.318661539	-8.109215339	8.095524127
	N387	-1.126135271	-2.312504158	6.090657881
	N388	-2.979793466	-0.936076278	5.712219564
	N389	-0.826161694	-0.154706356	5.295296161
	N390	-1.161598913	-5.780514287	10.452595482
	N391	0.715921590	-3.552169117	-6.097524044
	N392	2.116770723	-5.630854971	-2.040115716
	N393	0.141879675	-1.003817955	-6.425828210
	N394	-1.746719207	-0.266139447	-8.303196666
	N395	-4.086466401	-0.090626066	-6.835440251
	N396	-3.789269786	1.820523724	-4.781072541
	N397	0.252340674	2.506366552	-3.548962468
	N398	0.118589219	0.694803533	-2.383803107
	N399	-4.427176990	4.405715566	-5.558355344
	N400	-3.797089684	6.977849263	-9.485272315
	N401	-4.973111049	8.849653222	-9.292308542
	N402	0.133680598	3.308470219	9.483221530
	N403	2.379046323	8.067822419	5.315909657
	N404	4.463064905	8.698982834	2.503221331
	N405	4.115542488	7.543466745	-0.020020897
	N406	3.757128688	5.723656521	-4.609754812
	N407	5.145667436	7.543976201	-5.097834316

	N408	2.907727603	7.595237898	-5.693620180
	N409	1.459858993	6.507158192	-0.117562906
	N410	-0.001968708	8.850081632	0.400147866
	N411	0.013958241	11.154454711	-1.246077923
	N412	2.229875241	9.862063055	-3.214629945
	N413	-1.793400355	11.674583273	-3.261883848
	N414	10.937977353	-4.801613550	1.125240018
	N415	8.142190236	-2.756730941	0.148505071
	N416	7.357303186	-0.062190136	-1.013160659
	N417	8.100469845	2.150778045	0.652497811
	N418	8.886948098	-3.778049007	-4.341017214
	N419	6.951318641	-2.029758371	-5.564442214
	N420	7.978197494	-6.448048276	6.675830589
	N421	5.326748567	-5.574724362	3.292201731
	N422	6.977986266	-6.613126943	2.262704157
	N423	5.428945472	-6.805879956	8.576110305
	N424	-0.063469889	2.402508572	0.182110606
	N425	0.659009630	1.760145929	0.772699463
	O426	-3.718391556	-5.493464062	-5.336960761
	O427	-6.523205369	-3.185627757	-5.612669775
	O428	-8.415232493	-5.310626992	-3.199308452
	O429	-5.838162884	-4.445975475	-0.736579824
	O430	-5.805058482	-0.917656304	-1.135225752
	O431	-9.415671684	-0.802405334	-0.751484007
	O432	-10.287538709	-3.823000856	1.898563932
	O433	-6.101101941	-3.863516350	4.016663244
	O434	-5.999087203	0.448597605	3.907434253
	O435	-9.056468411	2.343030806	3.291438670
	O436	-11.892047692	0.593318723	4.503193657
	O437	-0.434670572	-9.429991287	8.336835398
	O438	-3.357837749	-6.314935305	10.033591252
	O439	-0.944137149	-3.218152452	12.956925851
	O440	-1.790317199	-1.077329420	-5.192638489
	O441	2.308100914	-4.968698286	-4.207302397
	O442	-1.242609999	1.952726727	-8.063019970
	O443	1.291450041	2.445653735	-7.278869717
	O444	-5.251884952	0.627753282	-8.683877564
	O445	-5.988757290	1.608403703	-4.214251707
	O446	-4.720498787	5.001360885	-3.370771041
	O447	-6.769557980	7.056543984	-5.524316488
	O448	-1.351128678	0.537252185	11.092939973
	O449	-2.581794402	5.517661069	4.108609416
	O450	2.193401094	8.880964478	2.485844056
	O451	4.090474714	9.620861244	-1.004464406
	O452	1.568003757	6.783546584	-2.388266821
	O453	-1.845106017	7.913746330	-0.589967611
	O454	-0.530086272	6.417445774	2.249885322
	O455	-1.121419738	12.353081957	0.340084776
	O456	-0.840303406	13.691368989	-3.742435105
	O457	1.182558928	11.035294060	-4.870769737
	O458	-4.587503912	13.368704599	-4.743158176
	O459	0.128981080	6.456449389	-6.237565452
	O460	8.708865893	-4.878146090	-0.533575941
	O461	7.960436525	-1.289327758	-2.849762961
	O462	8.462017220	3.425001397	-1.211444625
	O463	10.353351173	3.908456657	2.882814721
	O464	6.763424144	-3.663928079	-7.146139136
	O465	4.924507819	-6.952256264	-2.405820233
	O466	6.353894146	-6.001454243	-0.981740586

	O467 6.699377477 1.379257077 -6.592956195<br>O468 6.291271096 -4.731519982 8.867465634<br>O469 5.632847932 -5.990244577 12.063007221<br>O470 0.177433203 -6.375635001 0.286117544<br>O471 -0.541554450 -8.259747179 1.220702915<br>O472 4.031418832 -10.652772461 4.728303975<br>O473 3.774858033 -10.701122820 2.482637606<br>O474 2.730314490 -5.530855031 5.014013255<br>O475 1.174679349 -7.090902473 5.482096895<br>O476 2.628114643 -5.929809521 2.414751762<br>O477 1.526264282 -5.696933467 9.488890195<br>O478 1.922359525 -3.981753867 7.277058898<br>O479 1.347748326 4.327890929 -5.288489431<br>O480 4.080963502 -9.857854800 7.741857462<br>O481 1.913237884 -7.975044362 7.916819842<br>S482 3.519492841 5.311807960 2.452460752<br>S483 3.701182733 1.966027328 0.717541561<br>S484 3.786766520 -3.319302352 1.148379679<br>S485 1.411128443 2.179644670 3.547374869<br>S486 1.634162540 -0.947899953 0.052740033<br>S487 6.461881567 -0.629298873 2.034720955<br>S488 1.321635609 -3.211288213 3.852144903<br>S489 4.880896972 2.030876663 4.212963193<br>S490 4.817093946 -2.812261792 4.642521242<br>S491 2.378282053 -0.382368464 6.086516945

3 35 (S=2)

3 35 (S=2)<br>Fe(139) -2.298<br>Fe(140) 0.064<br>Fe(141) -2.652<br>Fe(142) 2.877<br>Fe(143) 3.230<br>Fe(144) 2.395<br>Fe(145) -0.189	bm22b2n2x6x2hncnx6h235tn.car_6<br>C1 -3.621154906 -5.861704976 -6.578976827<br>C2 -2.793622552 -7.077943703 -7.006240541<br>C3 -4.953641303 -3.937066953 -7.318599934<br>C4 -6.167438111 -4.086121941 -6.403372094<br>C5 -7.989295559 -5.516225736 -5.621559622<br>C6 -7.630371669 -5.641376867 -4.137530177<br>C7 -5.908872857 -6.213379985 -2.493458960<br>C8 -5.568357992 -4.836667976 -1.915476538<br>C9 -4.605084549 -2.630126955 -2.306017919<br>C10 -5.858290167 -1.778439769 -2.049749918<br>C11 -8.111508622 -1.183601809 -2.738697183<br>C12 -8.986170025 -1.663570978 -1.572695014<br>C13 -10.098693717 -3.567011321 -0.506588344<br>C14 -9.560974452 -3.857518341 0.899706586<br>C15 -7.638123321 -4.576963702 2.254227435<br>C16 -6.643777970 -3.603478889 2.889303285<br>C17 -5.387280596 -1.486763010 2.594546807<br>C18 -6.084268510 -0.123642396 2.761503178<br>C19 -4.127953583 -1.442809194 1.688071771<br>C20 -3.193878174 -0.287990399 2.069167863<br>C21 -3.381447034 -2.781164353 1.732869893<br>C22 -7.307149641 1.724470052 1.692023610<br>C23 -8.728491949 1.588655307 2.275155834<br>C24 -7.286459165 2.424040889 0.311422154<br>C25 -8.111700887 3.716048921 0.336336480<br>C26 -5.844700896 2.720133114 -0.123942328<br>C27 -10.895266520 0.497073334 2.209862797<br>C28 -10.991003851 0.338564778 3.715193414<br>C29 -2.529961064 -10.513207876 8.068903865<br>C30 -1.625502817 -9.285921236 8.212269966

	C31	-1.689577858	-6.790726973	8.367029459
	C32	-2.112182081	-6.244946541	9.744975290
	C33	-2.136741794	-5.857438502	7.226332806
	C34	-1.531243310	-4.453810044	7.310071049
	C35	-1.803841147	-3.660675443	6.031927550
	C36	-1.628845622	-1.177102026	5.746213457
	C37	-1.337863167	-5.228629974	11.834304034
	C38	-1.193643910	-3.723448826	11.925672919
	C39	-0.306672741	-3.154783910	-5.267048476
	C40	-0.721711221	-1.724578482	-5.643020373
	C41	0.118258467	-3.127903525	-3.779879718
	C42	0.282059217	-4.501957412	-3.097731610
	C43	1.681120361	-5.109991546	-3.191464037
	C44	0.066803388	0.346685679	-6.690299644
	C45	-1.033731679	0.722312392	-7.690608994
	C46	1.412752629	0.969357874	-7.116001130
	C47	-2.924372944	-0.048143395	-9.036645154
	C48	-4.156791694	0.226708401	-8.162920819
	C49	-5.139507681	0.149899449	-5.909820694
	C50	-4.941225332	1.217112219	-4.835671161
	C51	-3.503245067	2.972456575	-3.889434201
	C52	-4.267531808	4.272795877	-4.247704862
	C53	-2.020388717	3.368640257	-3.843628246
	C54	-1.042386476	2.379176877	-3.291477637
	C55	-1.134894074	1.280194762	-2.471706560
	C56	1.022763023	1.649292517	-2.852656953
	C57	-4.939621528	5.726132020	-6.127940764
	C58	-6.357064912	6.029833521	-5.652143002
	C59	-4.899453931	5.612321184	-7.673250249
	C60	-4.822888699	6.908181152	-8.424753979
	C61	-5.619595057	8.035613421	-8.361898194
	C62	-4.016652668	8.307083959	-9.866980248
	C63	-0.837650021	2.270205441	9.550484658
	C64	-0.454452963	1.110367216	10.452792401
	C65	-1.036325404	1.675376781	8.134105966
	C66	-1.459785410	2.700627653	7.107249868
	C67	-2.815520008	2.958954680	6.834710843
	C68	-0.494816413	3.431368341	6.390827132
	C69	-3.199316980	3.905437634	5.876470520
	C70	-0.861647499	4.387433156	5.442828529
	C71	-2.214138751	4.619769162	5.182651161
	C72	3.206914977	7.545752148	4.246915939
	C73	3.258560529	8.448112108	3.008321271
	C74	2.615531939	6.176657432	3.877250537
	C75	4.687894414	9.516933478	1.320083079
	C76	4.273668028	8.870018936	-0.002156232
	C77	3.658901046	6.794036782	-1.179889611
	C78	2.119869473	6.715746962	-1.270647237
	C79	4.330597277	5.406938987	-1.209346859
	C80	4.066820598	4.526687642	-2.436862347
	C81	4.693598837	4.983584795	-3.767762362
	C82	3.960519122	6.970432744	-5.095483977
	C83	-0.026562286	6.542239781	-0.117811073
	C84	-0.689666262	7.935342073	-0.119276045
	C85	-0.618935919	5.735282299	1.045227581
	C86	-0.658206449	10.169351172	0.868831804
	C87	-0.601434248	11.393641732	-0.041771016
	C88	0.242043154	12.363879801	-2.145833964
	C89	-0.869032502	12.652307262	-3.178184308

	C90	1.612696790	12.216856953	-2.847831879
	C91	1.655063977	10.972155740	-3.725129608
	C92	-2.868706847	11.787598496	-4.321290184
	C93	-3.887314938	12.876635438	-3.999224689
	C94	-3.583371372	10.415448882	-4.461885690
	C95	-2.652418149	9.324697613	-4.939997073
	C96	-2.402996384	9.158364025	-6.311136346
	C97	-1.975645384	8.486568355	-4.037230964
	C98	-1.488893078	8.209843531	-6.773284751
	C99	-1.041727209	7.544376443	-4.479421993
	C100	-0.803335464	7.423667752	-5.846565194
	C101	9.680770495	-4.064252434	1.483814625
	C102	8.779475931	-3.945854284	0.265698294
	C103	7.152165576	-2.486365390	-0.912837290
	C104	7.555980475	-1.242004638	-1.711658871
	C105	7.762527139	1.199764148	-1.634399940
	C106	8.225329804	2.301272565	-0.682905460
	C107	8.445670960	3.168630868	1.591102885
	C108	9.911391882	3.148191035	1.984278169
	C109	7.662413869	-4.344305950	-5.023411170
	C110	7.083965790	-3.330600007	-6.016445580
	C111	6.534121767	-4.862769019	-4.101755455
	C112	6.962584174	-6.141206966	-3.357004466
	C113	5.984138760	-6.464966887	-2.251639595
	C114	6.412358912	-1.015429968	-6.436238106
	C115	7.226619159	0.260816807	-6.353347093
	C116	4.885043071	-0.827543059	-6.250424211
	C117	4.490062289	-0.703483401	-4.800703031
	C118	4.610990541	0.520125070	-4.118712167
	C119	4.094770432	-1.840163643	-4.074964347
	C120	4.385470151	0.585977047	-2.739404474
	C121	3.867303854	-1.771702972	-2.695684616
	C122	4.031352971	-0.558587771	-2.019750187
	C123	6.717553048	-5.772311247	6.702490899
	C124	6.128722141	-5.679294155	8.119167601
	C125	5.711250109	-6.358897329	5.701497158
	C126	6.118428714	-6.308581050	4.262057977
	C127	7.138296120	-7.007985449	3.648530444
	C128	5.988610630	-5.887830852	2.123259651
	C129	4.874804099	-6.708081682	9.928905203
	C130	5.910922274	-6.360497209	10.975421036
	C131	0.048176576	-6.902244854	1.302498518
	C132	0.308388072	-6.483202864	2.721511061
	C133	1.803791070	-6.735703367	3.183202746
	C134	2.142389446	-8.249342757	3.058033352
	C135	3.522032919	-8.574619093	3.652674992
	C136	3.818434466	-10.042231165	3.762464469
	C137	1.851567106	-6.382793712	4.703825377
	C138	3.155885593	-0.519969585	2.793599426
	Fe139	4.669822807	-1.813210480	2.581871606
	Fe140	1.598738214	0.763814403	1.875781434
	Fe141	2.992484021	0.801621998	4.322292345
	Fe142	4.580031205	0.723987391	2.292780786
	Fe143	3.304310346	3.039402352	2.853313019
	Fe144	2.823442105	-1.743267280	4.327081271
	Fe145	2.250100937	-2.450779228	1.678889583
	H146	-2.453366207	-7.809911005	-5.132839794
	H147	-1.191804854	-6.971557727	-5.740065127
	H148	-4.028733251	-5.422121388	-8.548338210

	H149	-2.197561500	-6.811804267	-7.893266468
	H150	-3.508103991	-7.850086396	-7.340912107
	H151	-4.295846484	-3.198686507	-6.836917805
	H152	-6.528573852	-5.966360495	-7.137301960
	H153	-5.290041217	-3.521828067	-8.278542302
	H154	-8.464107057	-6.451184217	-5.948022580
	H155	-5.739563151	-6.284285133	-4.619226961
	H156	-8.725798037	-4.706280185	-5.706275637
	H157	-4.036339316	-2.641104178	-1.367077461
	H158	-4.594539585	-4.305416781	-3.658721913
	H159	-5.013981185	-6.850277148	-2.476824778
	H160	-7.868853901	-0.129157090	-2.555302544
	H161	-6.776059474	-2.542424214	-3.739502336
	H162	-6.664348968	-6.660785468	-1.836435243
	H163	-3.985736821	-2.149255106	-3.076954655
	H164	-8.757795956	-3.634488503	-2.166761877
	H165	-8.696594072	-1.250624935	-3.665898608
	H166	-4.021880584	-3.617077667	1.417501912
	H167	-2.508592857	-2.752846090	1.061775673
	H168	-3.009692240	-2.991551722	2.747813449
	H169	-4.476493397	-1.275310760	0.656098636
	H170	-10.479217322	-4.514890683	-0.914773528
	H171	-5.241111634	1.806541372	-0.213960905
	H172	-9.237635579	0.071330962	0.937240952
	H173	-9.164843294	3.536299967	0.598033137
	H174	-2.341612347	-0.252249193	1.373593970
	H175	-2.785538524	-0.426632109	3.080983079
	H176	-1.815294232	2.656795984	9.896971230
	H177	-5.842509996	3.223116712	-1.102001604
	H178	-8.084839860	4.197210835	-0.652456738
	H179	-5.354632332	3.385773256	0.605181147
	H180	-7.698629745	4.425610980	1.070826381
	H181	-7.742999675	1.737558417	-0.424455125
	H182	-6.775004881	2.355947307	2.419366895
	H183	-6.462114988	-0.002768753	0.724509902
	H184	-3.694800941	0.691264347	2.033289360
	H185	-0.073457277	4.781166306	1.127987101
	H186	-1.667571022	5.518257349	0.784620507
	H187	-1.198249282	6.116852880	2.880555202
	H188	-3.486182683	5.591685754	4.085126770
	H189	-0.099817579	4.918742897	4.870688993
	H190	0.565342577	3.221184323	6.545451803
	H191	-3.592045267	2.405499809	7.370001551
	H192	-6.756017766	-2.421430063	1.231444493
	H193	-7.110483872	-5.532590539	2.115465711
	H194	-8.446911857	-4.737753367	2.979641203
	H195	-7.670724780	-4.278351240	0.149527619
	H196	-10.946163046	-2.881365023	-0.377827202
	H197	-4.257260105	4.074408683	5.660337581
	H198	-5.088353346	-1.778303226	3.610483658
	H199	-11.517841049	1.352948762	1.900730057
	H200	-11.320755563	-0.413067126	1.757233005
	H201	-10.095551018	-0.119479423	4.198166259
	H202	-0.676148833	-5.703737248	12.574097047
	H203	3.019266842	-6.251383820	-2.139930915
	H204	-0.557949298	-8.594683623	1.993344279
	H205	6.979391248	-6.984973040	-4.061309831
	H206	7.972808760	-6.016480231	-2.940401891
	H207	5.631280785	-5.079991149	-4.693563099

H208	8.305842923	0.100470437	-6.121070645
H209	7.975663609	-5.184994022	-5.661866574
H210	1.054744865	-2.553023333	-3.696785872
H211	-0.418063202	-5.231565223	-3.540062399
H212	7.286177224	-1.790973461	-4.636006866
H213	6.570776002	-1.368788528	-7.474440308
H214	8.643322290	-3.120850108	-3.659268838
H215	1.521558138	-5.907577418	-1.275541619
H216	6.261594216	-4.067032243	-3.391346941
H217	9.348826498	-4.593628591	-3.880032013
H218	0.406503627	-4.226040177	-6.884378049
H219	-0.652708367	-2.564681035	-3.233679871
H220	7.217588598	-5.599732522	-0.979386291
H221	-0.007262309	-4.414352671	-2.040141192
H222	-3.863347830	2.669606127	-2.896562898
H223	-1.997971580	0.788484428	-2.038545080
H224	-6.014026126	0.451406816	-6.502845335
H225	-3.184162298	-0.498435136	-6.469066309
H226	-4.273353501	3.693103477	-6.213713617
H227	-6.477517802	8.296795379	-7.751755266
H228	-7.000756573	5.123314192	-5.535028532
H229	-5.754117942	5.001341898	-8.012305731
H230	-4.275457188	6.536996814	-5.790607685
H231	-3.418407327	8.788972532	-10.636289665
H232	-1.963120403	4.282967833	-3.230770574
H233	-1.701356049	3.659482281	-4.858891021
H234	-5.435772757	9.848144008	-9.482283031
H235	-3.991120467	5.049848828	-7.937678777
H236	-3.029117933	1.617305242	-5.499020516
H237	-5.404325524	-0.793253934	-5.409077824
H238	-2.800582545	0.816842969	-9.700849135
H239	2.102705547	1.540837252	-2.822514130
H240	-3.131091529	-0.924480547	-9.663866774
H241	0.220820137	3.783738778	10.438047592
H242	0.076739775	3.949717834	8.819638115
H243	1.943640116	6.305280217	0.753071504
H244	-0.340822469	6.061413291	-1.055669763
H245	0.878985672	8.722768367	0.944132662
H246	0.722562026	3.326933086	-4.134299098
H247	1.451701532	3.677123797	-6.070648556
H248	0.756711608	5.057689341	-5.669350859
H249	2.164463216	0.763627972	-6.340863402
H250	1.750354947	0.517847798	-8.063673492
H251	2.396398109	12.197632294	-2.076335374
H252	1.762118572	13.098238356	-3.483676588
H253	2.765197596	9.880518316	-2.301265247
H254	2.131596840	8.987283973	-3.653162656
H255	5.255586247	8.531384779	-5.362206864
H256	5.024688431	4.108605544	-4.346860264
H257	-1.313441443	8.086445669	-7.843959594
H258	-2.170545067	8.551549503	-2.963346669
H259	2.014927997	7.208462538	-5.751652146
H260	4.016129813	4.838825187	-0.319301887
H261	4.173149298	7.007329649	0.885635749
H262	2.987233149	4.359774705	-2.571799985
H263	-1.390951679	-1.235354676	-8.108428883
H264	5.748439448	9.792898809	1.244265754
H265	0.982645173	-1.661591269	-6.695045796
H266	5.416469247	5.572324627	-1.115509465

	H267	-0.482798324	6.941368823	-3.760849923
	H268	0.400052530	2.551274605	-7.648325149
	H269	4.102910451	10.443328051	1.401513729
	H270	6.001151199	7.056993662	-4.869657321
	H271	3.129772088	8.422984998	-6.273744161
	H272	1.452022640	-4.175598158	-5.583455491
	H273	-1.227801209	-3.761834407	-5.342354364
	H274	-2.930879674	9.780204741	-7.038561363
	H275	-0.247636636	0.837234666	-5.752777475
	H276	3.963114069	7.381996196	-2.056521964
	H277	5.587359070	5.587122599	-3.566859402
	H278	4.496779250	3.544536766	-2.198402240
	H279	2.875152213	5.274646318	-4.850076854
	H280	-1.723816418	9.945868665	1.027492603
	H281	0.445932845	10.336728099	-1.454322041
	H282	0.277063912	13.260120322	-1.508762965
	H283	-0.231502625	10.479171118	1.832589713
	H284	-1.770490994	10.883605578	-2.712485177
	H285	10.017948052	-3.066288126	1.800126063
	H286	9.038328219	-4.458161390	2.298426235
	H287	6.943316121	-4.735187315	6.414415049
	H288	11.298179888	-5.171302836	2.050344834
	H289	10.508155130	-5.770668592	0.744985242
	H290	2.643945941	5.540259501	4.771108534
	H291	1.496208215	8.419218654	4.930271933
	H292	5.303483763	8.255143279	2.934249038
	H293	1.570291968	6.308844220	3.561326887
	H294	2.846586473	9.000295451	5.646784307
	H295	7.834078081	3.061738024	2.500131368
	H296	4.228296837	7.397127406	4.630389585
	H297	-3.238722085	-5.804516368	7.222379212
	H298	-0.600180406	-6.911259257	8.325558066
	H299	7.615590030	1.289721313	1.042806724
	H300	-1.178754929	0.786239940	5.442691560
	H301	8.576746262	1.029825145	-2.352242170
	H302	3.575462594	-2.666341142	-2.140900123
	H303	6.921460627	1.620044980	-2.212322575
	H304	-2.878849811	-3.490592874	5.895099638
	H305	6.887935278	-0.131461852	-0.141466267
	H306	-1.458665076	-4.234234788	5.155677650
	H307	-1.824662010	-6.335726411	6.283315278
	H308	-0.441177843	-4.518736453	7.444995462
	H309	-1.934036461	-3.900575434	8.173578699
	H310	4.392394368	-1.699600570	-6.704668284
	H311	-3.302622968	-8.202020144	8.150597566
	H312	-2.322951342	-10.929755401	7.071060069
	H313	3.886651906	-0.491881828	-0.940926970
	H314	-4.280420441	-10.316596245	9.140185982
	H315	-4.508771369	-10.849406222	7.607826396
	H316	-0.076150899	-2.435256113	5.943387657
	H317	4.497363112	1.525870225	-2.195077294
	H318	3.965396894	-2.794916599	-4.590189489
	H319	3.340259873	-9.207840686	7.824424867
	H320	5.529776883	-7.415623627	5.967971658
	H321	1.727507203	-7.534061343	7.036279469
	H322	4.152612907	-10.073069168	6.834994558
	H323	10.526655892	2.358044748	1.490022029
	H324	2.203575934	-4.483562159	6.504451588
	H325	-0.367995889	-7.000018605	3.418566939

	H326	8.129378884	-2.113487194	0.919542687	
	H327	1.369208480	-8.834534570	3.580950042	
	H328	7.622893641	-7.107372287	1.562604659	
	H329	5.668070738	-5.524520831	1.155553470	
	H330	-2.168857661	-11.255275934	8.801094866	
	H331	0.208659104	-0.271215408	5.470327133	
	H332	1.727170101	-4.963200826	8.804981059	
	H333	2.362764852	-3.073218482	7.140899831	
	H334	-0.128506626	-5.735675622	10.123957275	
	H335	-3.550449197	-1.616301791	6.324971650	
	H336	3.965415586	-11.630225136	2.744441476	
	H337	4.292596898	-8.105105817	3.022493879	
	H338	3.619362284	-8.161874884	4.663279684	
	H339	2.130803134	-8.530244888	1.994455453	
	H340	6.158772058	-2.350351595	-0.456323266	
	H341	4.912911421	1.413256184	-4.671276460	
	H342	4.598274865	0.065463183	-6.826388073	
	H343	0.144728547	-5.399846444	2.798321629	
	H344	1.761208191	-6.502702345	9.001883652	
	H345	1.119203935	-8.477235423	8.113066944	
	H346	-3.337798461	-0.052262656	5.653396977	
	H347	7.895823234	-7.668280042	4.053923961	
	H348	4.751758914	-5.837961257	5.809024057	
	H349	7.126504749	-3.337450447	-1.596300000	
	H350	8.228059596	4.156879802	1.155668999	
	H351	-1.794592900	0.882145207	8.214678391	
	H352	-0.089207891	1.206340994	7.821905135	
	H353	8.654953467	-6.060196277	7.320250504	
	H354	7.836893809	-7.458145351	7.084928811	
	H355	4.497653520	-7.715606750	10.165039732	
	H356	5.229914564	-7.569021897	8.012797072	
	H357	6.958657942	-6.641070000	10.708268052	
	H358	4.024263597	-6.010941015	9.996678655	
	H359	0.637696720	0.876514774	10.456330219	
	H360	-3.912497080	13.200009812	-2.931434963	
	H361	-2.380320845	-5.469114749	12.106291838	
	H362	-1.365753688	-3.171355204	10.968986113	
	H363	-2.406287879	12.053057696	-5.285877489	
	H364	-4.030810612	10.140764245	-3.493466689	
	H365	-4.410677537	10.552402476	-5.173715116	
	H366	1.752397950	-3.594424322	0.597624791	
	H367	0.171419499	6.477824458	-7.244426010	
	H368	0.247847105	0.152700531	2.759159705	
	H369	1.075386258	-3.432363453	1.055563389	
	H370	0.738013032	-0.484706660	2.692184110	
	H371	0.050791067	-1.178703847	0.381098517	
	H372	0.476081862	0.123982796	-1.539102082	
	Mo373	3.459988140	-4.198025059	3.294188291	
	N374	-1.921338892	-7.643034743	-5.990762673	
	N375	-4.240782331	-5.178987676	-7.583636422	
	N376	-6.846319898	-5.256931979	-6.481669968	
	N377	-6.407373191	-6.157590775	-3.859153813	
	N378	-4.903954369	-3.986826795	-2.740494793	
	N379	-6.887947374	-1.950624052	-2.912925368	
	N380	-9.160791956	-3.006189999	-1.466286596	
	N381	-8.234783569	-4.159232957	0.996217933	
	N382	-6.340905098	-2.499278689	2.157481641	
	N383	-6.617351227	0.433502674	1.635944136	
	N384	-9.519614601	0.614945521	1.756259239	

	N385	-3.960413924	-10.203138078	8.176293957
	N386	-2.281525163	-8.110201987	8.193010332
	N387	-1.096006494	-2.374035034	6.022641865
	N388	-2.970792600	-0.991993305	5.773224817
	N389	-0.829036576	-0.167270677	5.372409549
	N390	-1.101742260	-5.747912247	10.500494600
	N391	0.772177197	-3.627796303	-6.141116623
	N392	2.097216900	-5.804108102	-2.114001626
	N393	0.174755316	-1.075008719	-6.432218757
	N394	-1.696462204	-0.282929258	-8.297986651
	N395	-4.034709357	-0.051055070	-6.831693950
	N396	-3.749023597	1.889866797	-4.834162113
	N397	0.316637801	2.577662424	-3.513180921
	N398	0.155333235	0.849303494	-2.225308102
	N399	-4.496989239	4.451440501	-5.573873035
	N400	-3.827373801	7.090272883	-9.372774208
	N401	-5.087731975	8.914336285	-9.281405779
	N402	0.240759324	3.260830623	9.558895798
	N403	2.399559975	8.136957802	5.324833175
	N404	4.499138859	8.706439303	2.507123796
	N405	4.120335032	7.527270884	-0.005152502
	N406	3.767219543	5.744273504	-4.615352974
	N407	5.149828115	7.597719772	-4.978042340
	N408	2.929894730	7.651051744	-5.643480680
	N409	1.434217903	6.576973028	-0.103882977
	N410	0.025497090	8.960164403	0.428010367
	N411	-0.022478963	11.225825772	-1.269044164
	N412	2.147746041	9.856400378	-3.121489938
	N413	-1.837430896	11.711509609	-3.299126522
	N414	10.842730367	-4.903354511	1.175083407
	N415	8.120419660	-2.778326860	0.137568874
	N416	7.395020445	-0.078914008	-1.052421003
	N417	8.087081510	2.118007964	0.654345651
	N418	8.870311741	-3.832123456	-4.365268982
	N419	7.001782968	-2.038854066	-5.590477307
	N420	7.988417599	-6.522392446	6.694714050
	N421	5.413305663	-5.602781204	3.288563135
	N422	7.036554726	-6.730402371	2.302450454
	N423	5.447849110	-6.759423254	8.594293445
	N424	-0.452250941	2.374381635	0.499874151
	N425	0.344636988	1.775408224	1.031936807
	O426	-3.763133714	-5.532977576	-5.386214068
	O427	-6.501837019	-3.162962230	-5.642579199
	O428	-8.443974392	-5.311964633	-3.258627463
	O429	-5.895536558	-4.515408765	-0.762709468
	O430	-5.904975781	-0.974072973	-1.104932007
	O431	-9.532421616	-0.850356034	-0.807763034
	O432	-10.337901260	-3.849015007	1.863753332
	O433	-6.157924593	-3.863428241	3.999382392
	O434	-6.151979420	0.420895383	3.873169514
	O435	-9.133580603	2.340698744	3.177414802
	O436	-11.994023492	0.618525199	4.354038180
	O437	-0.389931561	-9.438413822	8.314364317
	O438	-3.301997573	-6.281435638	10.104071430
	O439	-0.924187254	-3.135158676	12.960628289
	O440	-1.779962898	-1.199135718	-5.241362255
	O441	2.389110147	-4.981140683	-4.214822386
	O442	-1.308212305	1.929704933	-7.868580975
	O443	1.297629599	2.394635650	-7.256928212

	O444 -5.184730525 0.669466007 -8.689123899<br>O445 -5.841401320 1.460480688 -4.027754841<br>O446 -4.538808647 5.114659412 -3.387239208<br>O447 -6.789787097 7.151247125 -5.450994431<br>O448 -1.252216179 0.453171857 11.105228639<br>O449 -2.520150269 5.551967460 4.210499123<br>O450 2.231892953 8.926295333 2.491448941<br>O451 4.131999044 9.593557800 -1.009336729<br>O452 1.563560108 6.796865291 -2.380263668<br>O453 -1.839533739 8.070996307 -0.561068532<br>O454 -0.539893303 6.488880687 2.258151734<br>O455 -1.082386879 12.468256624 0.337276679<br>O456 -0.856498972 13.700811554 -3.837622621<br>O457 1.175934339 10.962526009 -4.869757260<br>O458 -4.639918464 13.348814380 -4.834986443<br>O459 0.176755227 6.506301880 -6.268038064<br>O460 8.660850630 -4.909348930 -0.534732412<br>O461 8.015221387 -1.323444242 -2.873639289<br>O462 8.689761966 3.343310175 -1.176911494<br>O463 10.399579258 3.928418199 2.784573266<br>O464 6.683407269 -3.683129784 -7.139242023<br>O465 4.911435868 -7.032598267 -2.440222307<br>O466 6.318660776 -6.056299282 -1.014011827<br>O467 6.782765407 1.381369216 -6.558187094<br>O468 6.327643220 -4.686153273 8.843081633<br>O469 5.636991918 -5.879321765 12.065908375<br>O470 0.246630089 -6.217216033 0.306579049<br>O471 -0.439048725 -8.170634602 1.121524066<br>O472 4.106050507 -10.637375606 4.799027740<br>O473 3.754697364 -10.688359238 2.567899688<br>O474 2.680603132 -5.449800453 5.029827837<br>O475 1.145897578 -7.029187100 5.516531118<br>O476 2.679408229 -5.961704398 2.452067249<br>O477 1.563580659 -5.659612475 9.489623357<br>O478 1.917220360 -3.932556098 7.278336150<br>O479 1.369235945 4.352923636 -5.341835112<br>O480 4.131599170 -9.802093533 7.776225113<br>O481 1.936419221 -7.948062024 7.924724517<br>S482 3.526991894 5.339454316 2.496760371<br>S483 3.362317764 1.956729867 0.760166163<br>S484 4.139132959 -3.414976751 1.122390124<br>S485 1.399585289 2.257237425 3.716614388<br>S486 1.375694784 -0.873096610 0.232737359<br>S487 6.415156191 -0.545129444 1.977605303<br>S488 1.282524506 -3.166932886 3.543633805<br>S489 4.880217525 2.007633347 4.179913008<br>S490 4.790619301 -2.756807617 4.635396202<br>S491 2.342490508 -0.445934314 6.056793469

TS 3 → 4 35 (S=2)

TS 3 → 4 35 (S=2)	bm22b2n2x6h2x2h2n35dtg_1_53241.781
Fe(139) 0.546 -2.424<br>Fe(140) 0.378 -0.077<br>Fe(141) 0.542 -2.671<br>Fe(142) 0.564 2.773<br>Fe(143) 0.613 3.199	C1 -3.164203554 -4.246350455 7.914223073<br>C2 -2.966211345 -3.539329132 9.258731828<br>C3 -2.953272139 -6.342420535 6.658354974<br>C4 -4.329117528 -6.399810996 5.995864952<br>C5 -6.755291966 -6.376384253 6.280996386<br>C6 -7.109611894 -5.102276466 5.508212440<br>C7 -6.694964699 -2.708915147 5.213894757

Fe(144) 0.511 2.268<br>Fe(145) 0.293 0.425	

C8	-5.958158218	-2.676664937	3.871563393
C9	-3.953060357	-3.260688011	2.613065374
C10	-4.583673490	-4.212935653	1.585222019
C11	-5.780576680	-6.284514118	1.165651194
C12	-7.142633989	-5.813606730	0.637203375
C13	-9.373837738	-4.983855714	1.209269988
C14	-9.650109323	-3.582817158	0.650713754
C15	-9.076007766	-1.192452523	0.727390675
C16	-8.073051023	-0.565359543	-0.243982712
C17	-5.916869296	-0.880500022	-1.432541356
C18	-5.850295679	-1.742710991	-2.706094966
C19	-4.571449588	-0.778632345	-0.667525174
C20	-3.409764227	-0.440801280	-1.607060507
C21	-4.672030504	0.249674286	0.464826027
C22	-5.445648956	-3.962793895	-3.688696359
C23	-6.836900856	-4.369481529	-4.214840423
C24	-4.553827685	-5.183696626	-3.358744337
C25	-4.572958717	-6.208288769	-4.499104645
C26	-3.114713396	-4.741808215	-3.061277325
C27	-9.033939629	-5.290445747	-3.746312000
C28	-9.783955678	-4.230077569	-4.529669954
C29	-10.358942209	8.274329642	2.556755938
C30	-9.113021080	8.415066423	1.677644431
C31	-7.963104335	7.418375872	-0.303119600
C32	-8.562539256	7.874620943	-1.648150741
C33	-7.391309251	5.992416745	-0.390578347
C34	-6.276297738	5.846975284	-1.428781160
C35	-5.564773667	4.502248182	-1.289278711
C36	-4.116622424	3.387350946	-3.002675432
C37	-8.288340548	9.357999374	-3.575573749
C38	-7.452529199	8.946901837	-4.768421075
C39	0.260619334	-2.486895396	5.596516995
C40	0.797286647	-3.584895877	4.664361466
C41	0.007842815	-1.230793678	4.729198965
C42	-0.818784376	-0.105719413	5.386700898
C43	-0.003822185	0.932007574	6.156209155
C44	2.853508896	-4.699236962	3.912459208
C45	2.606029475	-6.203659304	4.090046573
C46	4.363247270	-4.386538430	3.921616230
C47	1.208132329	-7.922336728	5.134694500
C48	0.028003662	-8.171395946	4.183118159
C49	-1.633522178	-7.188187931	2.659422160
C50	-1.353120700	-6.825717350	1.200361023
C51	0.247778301	-5.980772770	-0.463410617
C52	0.448882746	-7.230302265	-1.359826702
C53	1.576141041	-5.213035260	-0.528834120
C54	1.635348222	-3.852294119	0.093164498
C55	0.699771492	-2.914457221	0.458765593
C56	2.708928033	-2.019144623	0.783326678
C57	1.439091181	-9.496481395	-1.413225391
C58	0.288796702	-10.188584078	-2.137130348
C59	2.064594951	-10.439496219	-0.358371716
C60	3.036466166	-11.460668234	-0.872569950
C61	2.976567541	-12.291707811	-1.974500995
C62	4.854347855	-12.635685430	-0.849279482
C63	-3.217159317	5.009453860	-7.824748888
C64	-3.850465599	6.337624861	-7.452406049
C65	-3.120453102	4.180286886	-6.520838653
C66	-2.541255581	2.800353100	-6.732058363

C67	-3.364456992	1.691827587	-7.006199320
C68	-1.152584179	2.586174141	-6.658328249
C69	-2.825576288	0.413739577	-7.190805714
C70	-0.600346751	1.321548524	-6.854707480
C71	-1.438116348	0.234248384	-7.117333536
C72	4.567396769	1.711973354	-7.825805548
C73	5.532431764	0.522764528	-7.744635512
C74	3.589675885	1.680025336	-6.640163667
C75	7.827135409	-0.212981089	-7.268013136
C76	7.712521085	-1.096506475	-6.023924498
C77	6.688851450	-1.390784254	-3.798401284
C78	5.478103624	-2.339454643	-3.943261611
C79	6.546268820	-0.421250081	-2.610227807
C80	6.356837014	-1.038572271	-1.221494561
C81	7.571001563	-1.753905701	-0.600274598
C82	8.519200084	-3.924925785	-1.408582711
C83	3.261903238	-2.753559166	-4.879683399
C84	3.430728811	-3.755506344	-6.039455554
C85	1.961751251	-1.968959159	-5.106504082
C86	4.157781208	-4.021196450	-8.358619786
C87	5.197344017	-5.077459874	-8.725546472
C88	7.186874173	-6.351356444	-8.041067382
C89	6.872154554	-7.848468553	-7.832719150
C90	8.458001644	-5.945631402	-7.261552794
C91	8.259765715	-6.052353380	-5.753878282
C92	5.364415679	-9.496594528	-6.808606331
C93	4.999887330	-10.317993663	-8.039806948
C94	4.170458457	-9.487496480	-5.814763870
C95	4.512622069	-8.803639475	-4.511400947
C96	5.160365463	-9.509300934	-3.486445207
C97	4.252610100	-7.437510462	-4.311957055
C98	5.559838767	-8.872860056	-2.308722899
C99	4.669671362	-6.775861526	-3.152143254
C100	5.328571635	-7.504691382	-2.164000698
C101	4.920998932	8.576748754	3.901940894
C102	4.752664121	7.152540666	4.403685179
C103	4.638053434	4.768344378	3.715939900
C104	5.878929185	3.949663554	3.343177439
C105	7.219422951	3.095727856	1.475214790
C106	7.765013738	3.555811101	0.125586063
C107	7.500009631	4.888455620	-1.907574094
C108	8.449931548	6.072719522	-1.902992396
C109	5.748936523	3.024080055	7.800632236
C110	6.185327568	1.578005320	7.535139389
C111	4.261838285	3.189792878	7.410665042
C112	3.673328941	4.493936580	7.977036281
C113	2.320851728	4.773054266	7.360414920
C114	6.972312938	-0.069052167	5.908799604
C115	8.204530786	-0.047166171	5.026749522
C116	5.812055156	-0.953287651	5.388286709
C117	5.005119431	-0.260021560	4.322432783
C118	5.434927439	-0.242757767	2.984549535
C119	3.866498383	0.485090474	4.674176512
C120	4.772564796	0.552047515	2.042046465
C121	3.204415672	1.276918276	3.729636924
C122	3.674740517	1.331171349	2.414552844
C123	-0.300610341	11.014518341	1.408577764
C124	-1.274370888	11.597765744	0.374759551
C125	-0.976858546	9.979686873	2.320656185

	C126	-0.065841833	9.217211282	3.230351853
	C127	0.622616748	9.695013016	4.327747227
	C128	0.879134318	7.499422775	4.190744431
	C129	-3.477373599	12.473062520	-0.158037279
	C130	-2.923958643	13.675456709	-0.889645825
	C131	-3.881224084	3.737104670	4.397481380
	C132	-4.043172316	4.714912168	3.269409953
	C133	-3.185479294	6.035502174	3.452194594
	C134	-3.621755396	6.760371766	4.757426752
	C135	-2.937560623	8.129104580	4.915980791
	C136	-3.527595797	8.964915209	6.014408900
	C137	-3.575273054	6.968000112	2.262017409
	C138	1.094976939	4.053520868	-0.762130859
	Fe139	1.725827344	5.397530643	0.588106819
	Fe140	1.083592410	1.913394939	-1.402236932
	Fe141	1.000256756	4.354648989	-2.779710660
	Fe142	3.009284257	4.103622063	-1.188486977
	Fe143	2.980301069	2.687882372	-3.491324765
	Fe144	-0.345500538	5.412262562	-0.909298020
	Fe145	-0.127532211	3.556459016	1.080501243
	H146	-3.750730186	-1.744300215	8.690449024
	H147	-2.119445839	-1.766671862	8.693025446
	H148	-2.489091766	-6.016817105	8.719344960
	H149	-2.039527009	-3.907874791	9.726586019
	H150	-3.792076495	-3.867638440	9.913156515
	H151	-2.273451574	-5.908446503	5.911437807
	H152	-5.256818159	-6.333417524	7.825618653
	H153	-2.626275704	-7.369093492	6.869706011
	H154	-7.462678841	-6.493308497	7.113021118
	H155	-5.773307111	-4.014187126	6.629008815
	H156	-6.897895868	-7.216342613	5.588237714
	H157	-3.898222473	-2.275524925	2.131343085
	H158	-4.261158499	-3.483620795	4.734451317
	H159	-6.339366245	-1.883158293	5.845329068
	H160	-5.134972881	-6.460131330	0.296466242
	H161	-5.050391379	-5.583433615	3.064854867
	H162	-7.757046342	-2.547035078	4.995764273
	H163	-2.929340931	-3.590888990	2.840303603
	H164	-7.735585298	-5.275098486	2.547061216
	H165	-5.923685740	-7.236686602	1.695153071
	H166	-5.483430606	0.006375853	1.165618496
	H167	-3.729951272	0.281212317	1.033705264
	H168	-4.850342911	1.260351511	0.064680184
	H169	-4.375245588	-1.767044971	-0.220440288
	H170	-9.992646886	-5.109668823	2.109383970
	H171	-3.061445838	-4.025209941	-2.229323353
	H172	-7.468704010	-4.990242029	-2.325721658
	H173	-5.575025982	-6.628484012	-4.667996437
	H174	-2.464659104	-0.419963898	-1.044459139
	H175	-3.544667878	0.552593224	-2.059565277
	H176	-3.921496279	4.488666941	-8.502247795
	H177	-2.502995853	-5.614978095	-2.790470452
	H178	-3.891454106	-7.039622089	-4.265744328
	H179	-2.663293827	-4.272185765	-3.950647272
	H180	-4.235982342	-5.748508156	-5.441685442
	H181	-4.965617901	-5.665325926	-2.453257013
	H182	-4.990391172	-3.401534421	-4.518232403
	H183	-5.274098074	-3.412598138	-1.616393844
	H184	-3.296584299	-1.175302167	-2.418557052

	H185	1.901820177	-1.159435173	-4.360614557
	H186	1.127854544	-2.667364588	-4.931405087
	H187	0.979777218	-1.308883971	-6.670956916
	H188	-1.523218990	-1.682852623	-7.411416470
	H189	0.475656339	1.168460957	-6.772511718
	H190	-0.490064661	3.417194999	-6.406523329
	H191	-4.448742977	1.822507350	-7.066944278
	H192	-6.922322430	-2.242941428	-0.115144214
	H193	-9.083803019	-0.563691739	1.630337495
	H194	-10.067173199	-1.124616078	0.259542362
	H195	-8.138259440	-2.761179358	1.816411554
	H196	-9.724934885	-5.692809428	0.449004852
	H197	-3.482301579	-0.439436374	-7.380488827
	H198	-6.210864464	0.118998875	-1.779255154
	H199	-8.972804707	-6.204806497	-4.358202598
	H200	-9.633466277	-5.519973751	-2.851176723
	H201	-9.490712214	-3.177625615	-4.305192231
	H202	-8.338715670	10.456640507	-3.527755343
	H203	0.094009382	2.940800485	6.551906713
	H204	-5.449380808	4.512450459	5.207073300
	H205	3.542884113	4.394240569	9.064257550
	H206	4.358045137	5.332677510	7.786753380
	H207	3.672071418	2.338802731	7.785163810
	H208	8.879768812	0.821794526	5.207201490
	H209	5.817467945	3.119161287	8.895265724
	H210	0.980934464	-0.833761631	4.395986891
	H211	-1.547887415	-0.536449374	6.094316174
	H212	6.559361443	2.005728927	5.537169098
	H213	7.329362137	-0.528212260	6.853244390
	H214	6.581751112	4.059426734	6.204785723
	H215	-1.213671056	2.477051122	5.464134677
	H216	4.180335886	3.169321750	6.312940545
	H217	6.511698176	4.919151914	7.607951695
	H218	0.932489260	-2.727798767	7.535170031
	H219	-0.529643378	-1.563068197	3.829289575
	H220	3.200173751	6.067044212	6.166372692
	H221	-1.416548093	0.398013580	4.613393722
	H222	-0.564635140	-5.401859496	-0.925447125
	H223	-0.383335281	-2.940469225	0.435238618
	H224	-1.965396206	-8.234960680	2.667526282
	H225	-0.195128870	-6.128657666	3.829259715
	H226	0.988301458	-8.296148777	0.303751097
	H227	2.241636717	-12.412623568	-2.762581998
	H228	-0.681338987	-10.149560386	-1.584092356
	H229	1.255579044	-10.919584768	0.220322422
	H230	2.194783998	-9.198059111	-2.156339240
	H231	5.817397332	-13.068237152	-0.589509404
	H232	1.827061689	-5.110892232	-1.597660002
	H233	2.367127226	-5.840816842	-0.085123310
	H234	4.414358543	-13.739249505	-2.617005518
	H235	2.622294334	-9.811262962	0.353304799
	H236	0.526501116	-6.115944069	1.669828727
	H237	-2.481806712	-6.578754528	3.005164626
	H238	1.982717431	-8.657121964	4.882697631
	H239	3.485248727	-1.305333644	1.043394035
	H240	0.872962197	-8.125829642	6.160147096
	H241	-1.989481241	5.611355624	-9.348725487
	H242	-1.392144999	4.360691423	-8.484534271
	H243	4.358167013	-0.882499673	-4.904733004

	H244	3.131358527	-3.364520330	-3.974978826
	H245	4.602708981	-2.417133138	-7.061561895
	H246	3.808647580	-3.742176892	0.185833252
	H247	5.304654535	-4.766972994	1.235484823
	H248	5.285937829	-5.454131369	-0.201952994
	H249	4.512784668	-3.322735630	3.691243612
	H250	4.785467535	-4.593046351	4.919632751
	H251	8.734399308	-4.923746076	-7.560507452
	H252	9.262758586	-6.631047271	-7.557063085
	H253	7.971170509	-3.973777569	-5.551782090
	H254	7.645601807	-4.953224661	-4.146002085
	H255	10.408493238	-3.970830971	-2.197074401
	H256	7.584740346	-1.587342830	0.487372108
	H257	6.048157100	-9.441403773	-1.513668036
	H258	3.716402768	-6.855312957	-5.067369486
	H259	7.387026225	-5.649047539	-1.508173927
	H260	5.687394490	0.243301278	-2.795075402
	H261	6.397796226	0.235440974	-5.156185689
	H262	5.486356462	-1.713031047	-1.211276164
	H263	1.420019698	-5.863613525	5.704314698
	H264	8.817122481	0.262664431	-7.253638349
	H265	2.548591693	-3.297278865	5.587573723
	H266	7.443730573	0.219191757	-2.601532664
	H267	4.517612949	-5.700032462	-3.048632459
	H268	4.607369803	-6.040814232	2.916249413
	H269	7.792338498	-0.890590471	-8.132580392
	H270	10.046506974	-2.551114524	-1.281002343
	H271	9.060953643	-5.839563810	-1.880398539
	H272	1.220282243	-1.213402896	6.896058105
	H273	-0.721840701	-2.854662551	5.947977812
	H274	5.359299730	-10.577312800	-3.601710385
	H275	2.476831796	-4.493988088	2.895585792
	H276	7.550958462	-2.054353404	-3.647015704
	H277	8.498622301	-1.319814448	-0.995268924
	H278	6.097547058	-0.202806594	-0.557530740
	H279	6.729697862	-3.712542410	-0.482503775
	H280	3.175539809	-4.510672891	-8.441454338
	H281	6.067124458	-4.928944939	-6.871027768
	H282	7.402327468	-6.255205380	-9.115064886
	H283	4.212964055	-3.244201016	-9.132947137
	H284	5.145113654	-7.360279877	-6.858634998
	H285	5.541155075	8.584435945	2.993893348
	H286	3.904998573	8.907608850	3.600646366
	H287	0.481151608	10.512597010	0.820108270
	H288	5.432903027	10.392927556	4.675441031
	H289	5.031072270	9.279868883	5.808756006
	H290	2.955019169	2.573740588	-6.698187914
	H291	3.380105328	0.779972038	-9.194447129
	H292	7.060251172	1.780991670	-7.186934333
	H293	2.957852749	0.783383862	-6.718693734
	H294	4.432704849	1.832844299	-9.869914041
	H295	6.614937949	5.150029297	-2.507901702
	H296	5.142303253	2.649986878	-7.782415217
	H297	-8.214391536	5.290370342	-0.606819949
	H298	-7.165597759	8.105334854	0.004823404
	H299	6.158539334	4.832061800	-0.262702275
	H300	-2.684526534	2.620840368	-4.236977505
	H301	8.042818454	3.153775488	2.199457069
	H302	2.330172783	1.865024356	4.018837746

	H303	6.981361787	2.025025646	1.359930690
	H304	-6.250537352	3.669426143	-1.491193743
	H305	5.313499828	4.149079921	1.397936087
	H306	-5.207171350	4.375405633	-0.254040439
	H307	-6.999459047	5.746538251	0.609472649
	H308	-5.530004580	6.643930524	-1.293528795
	H309	-6.676683450	5.943972285	-2.450493221
	H310	5.175121365	-1.202020276	6.249490326
	H311	-9.806973110	6.844695356	0.653305727
	H312	-9.998437499	7.927660854	3.537283645
	H313	3.196347104	1.967226842	1.669685580
	H314	-12.018546937	7.788665100	1.436563547
	H315	-11.865309600	6.894253856	2.800936722
	H316	-3.605636330	5.004001042	-1.922592113
	H317	5.101778466	0.590940053	1.002053681
	H318	3.501098233	0.455286507	5.702985213
	H319	-5.096881819	11.127871854	2.771735236
	H320	-1.727317647	10.495184868	2.946275635
	H321	-5.181949351	8.939677662	1.685851904
	H322	-4.530373118	11.278723861	4.200350366
	H323	8.741782519	6.447477051	-0.891315913
	H324	-3.084446784	7.609859430	-0.253512519
	H325	-5.098387204	4.996488325	3.135197379
	H326	4.884574245	6.459442814	2.478531630
	H327	-4.714276676	6.901081813	4.731514765
	H328	1.768710196	8.593778293	5.766438684
	H329	1.185149997	6.493866124	4.448935281
	H330	-10.751670511	9.294088323	2.706954522
	H331	-2.123308756	3.909892872	-3.195184133
	H332	-4.593105551	9.072893233	-1.390318319
	H333	-2.504120337	7.570209005	-1.693900043
	H334	-6.926179593	9.135059266	-1.921606026
	H335	-6.055107252	2.802169123	-3.345724935
	H336	-3.806784037	9.008599233	7.885582557
	H337	-1.868988005	7.968977740	5.119360288
	H338	-3.024927274	8.714730925	3.994702556
	H339	-3.367981259	6.126860111	5.620197381
	H340	3.762844536	4.433268901	3.137266480
	H341	6.312394409	-0.827790957	2.697321918
	H342	6.262237438	-1.886625751	5.015861790
	H343	-3.677866790	4.240812561	2.348027319
	H344	-5.407389151	9.855605579	-0.320851145
	H345	-6.548746430	9.685967189	1.609327028
	H346	-4.860948601	1.804478258	-4.066782125
	H347	0.731982658	10.698901238	4.721688840
	H348	-1.524467947	9.258934384	1.700221388
	H349	4.444398562	4.631923325	4.781310591
	H350	8.008440188	4.049299150	-2.408430728
	H351	-4.136373711	4.103371511	-6.104609725
	H352	-2.500160292	4.739103354	-5.801724959
	H353	0.845893716	12.710258302	1.549977142
	H354	-0.367630039	12.658824822	2.646355218
	H355	-4.357763288	12.815168505	0.409112437
	H356	-2.864024074	11.706586662	1.731988530
	H357	-2.147280330	14.253958489	-0.334048955
	H358	-3.822148516	11.721333281	-0.886190751
	H359	-3.125762710	7.112461310	-7.102478181
	H360	4.700858295	-9.720061258	-8.932866640
	H361	-9.309912975	8.978316596	-3.753633075

	H362	-6.887963429	7.989860298	-4.648438974
	H363	6.228408919	-9.985288372	-6.329052656
	H364	3.308429052	-8.995988866	-6.293576171
	H365	3.891730888	-10.536649398	-5.636635066
	H366	-0.832420579	3.068523250	2.515946798
	H367	6.153380646	-7.472733350	-0.399302828
	H368	-1.158429318	1.739435282	-1.765996753
	H369	-1.322847871	2.758466411	1.926402857
	H370	-1.160713511	2.175375132	-1.143704583
	H371	-0.485745401	0.758050427	0.701808201
	H372	1.016689287	-0.856767421	1.110040498
	Mo373	-0.697160365	6.097864455	1.716601558
	N374	-2.940104161	-2.086108238	9.213780177
	N375	-2.911960379	-5.585419556	7.901313713
	N376	-5.404512247	-6.380477597	6.820351827
	N377	-6.522679995	-3.957845832	5.939981177
	N378	-4.695283790	-3.176765281	3.863198773
	N379	-5.147190641	-5.343580930	2.075423100
	N380	-7.996723634	-5.294114188	1.557685136
	N381	-8.839732163	-2.579048151	1.092558926
	N382	-7.000830354	-1.334701873	-0.567952887
	N383	-5.540637748	-3.060751325	-2.538124972
	N384	-7.731236511	-4.828112493	-3.300723779
	N385	-11.338231800	7.314717902	2.034450218
	N386	-9.003038629	7.478849586	0.715351313
	N387	-4.397022803	4.403015738	-2.172796254
	N388	-5.082881316	2.511563216	-3.372230929
	N389	-2.859288345	3.201802555	-3.419957955
	N390	-7.828816281	8.795953263	-2.320559949
	N391	1.206393615	-2.228378137	6.686715167
	N392	-0.405899057	2.212090499	6.031634448
	N393	2.118193658	-3.855412176	4.831800954
	N394	1.765735030	-6.583360290	5.073952878
	N395	-0.513234762	-7.072731212	3.578371803
	N396	-0.112665953	-6.324869903	0.906852133
	N397	2.876310313	-3.262809369	0.314683809
	N398	1.394990064	-1.799265958	0.888370675
	N399	0.938100676	-8.318388238	-0.712003119
	N400	4.214711153	-11.685736678	-0.178424064
	N401	4.141524070	-13.028744273	-1.943137724
	N402	-1.891874754	5.249954007	-8.396695295
	N403	3.795683413	1.710047918	-9.077337504
	N404	6.819258706	0.818143511	-7.406391971
	N405	6.952681433	-0.623701868	-5.012388329
	N406	7.566482162	-3.205753797	-0.820296377
	N407	9.712227449	-3.392634450	-1.737344912
	N408	8.273001164	-5.207558740	-1.763767726
	N409	4.421585415	-1.888236952	-4.672188249
	N410	4.266685960	-3.385620217	-7.052825952
	N411	6.071805585	-5.455041496	-7.746741576
	N412	7.842669598	-4.908910870	-5.145020803
	N413	5.720536784	-8.135805699	-7.177548030
	N414	5.546587974	9.410134521	4.933572720
	N415	4.847593666	6.189329466	3.468053511
	N416	6.082239588	3.816330182	2.017935185
	N417	7.074530197	4.487893294	-0.578955319
	N418	6.692221481	3.989229769	7.223944200
	N419	6.592507878	1.285963373	6.267868663
	N420	0.349093977	12.084471263	2.190815291

	N421	0.113245175	7.837623948	3.157369521
	N422	1.210418836	8.596746704	4.917384635
	N423	-2.527013963	11.926583235	0.795200965
	N424	0.867937480	-0.939406647	-2.042534949
	N425	0.947819397	0.163145417	-1.790061214
	O426	-3.576362754	-3.654206725	6.898599958
	O427	-4.432909531	-6.469230467	4.760205010
	O428	-7.915329090	-5.134948639	4.563364825
	O429	-6.495151509	-2.225585738	2.847645033
	O430	-4.562822456	-3.950905516	0.372071360
	O431	-7.447859091	-5.953980510	-0.559501224
	O432	-10.597488553	-3.410978241	-0.128081162
	O433	-8.262560009	0.584867550	-0.664835283
	O434	-6.081892039	-1.250047393	-3.819643982
	O435	-7.122560025	-4.291845302	-5.421004390
	O436	-10.691294739	-4.492039183	-5.305095508
	O437	-8.296082429	9.333042852	1.904098917
	O438	-9.642224964	7.407001163	-2.049961533
	O439	-7.404307580	9.588767324	-5.805484529
	O440	0.081320394	-4.168423575	3.823432664
	O441	0.971585617	0.606854562	6.869771343
	O442	3.131672889	-7.005761742	3.288463774
	O443	5.055298626	-5.154210302	2.923635020
	O444	-0.379999549	-9.326615107	4.014329373
	O445	-2.215194114	-7.006496366	0.337901458
	O446	0.275901911	-7.176089971	-2.580176880
	O447	0.396464543	-10.766266124	-3.204740692
	O448	-5.051842745	6.560501171	-7.498597065
	O449	-0.839928868	-1.000029032	-7.279515409
	O450	5.169784159	-0.644339874	-7.980016440
	O451	8.340337247	-2.175018183	-5.989864609
	O452	5.509400004	-3.466364670	-3.418244951
	O453	2.787382784	-4.814591814	-6.042080013
	O454	1.920702959	-1.451524224	-6.439295798
	O455	5.202946449	-5.568860626	-9.861598763
	O456	7.640055271	-8.721099023	-8.260728925
	O457	8.385260838	-7.129870688	-5.151142121
	O458	4.997700261	-11.538419843	-8.049861795
	O459	5.787826699	-6.818756807	-1.026147799
	O460	4.508182538	6.933150723	5.618604624
	O461	6.640791292	3.474513212	4.214719497
	O462	8.810360431	3.029632686	-0.293797463
	O463	8.876143129	6.589365108	-2.923413696
	O464	6.138112960	0.715276279	8.428444576
	O465	1.292469076	4.189174830	7.691447533
	O466	2.290000407	5.680221279	6.365990009
	O467	8.492411792	-0.911618479	4.210798589
	O468	-0.912952172	11.841261159	-0.791631089
	O469	-3.344173276	14.053131784	-1.974283488
	O470	-2.999186219	2.892590027	4.487507093
	O471	-4.799438527	3.813044267	5.412679702
	O472	-4.086643626	10.049523829	5.868650206
	O473	-3.389249718	8.397056278	7.241980661
	O474	-2.604289715	7.317468609	1.486266794
	O475	-4.760852744	7.361427223	2.142835081
	O476	-1.838700017	5.747950121	3.455860586
	O477	-5.334100655	9.724678307	-1.303457647
	O478	-3.337208733	7.721139593	-1.206206467
	O479	5.291142713	-4.576594358	0.255613735

	O480 -4.767807502 11.808774036 3.411621289<br>O481 -5.577500727 9.836848472 1.479132998<br>S482 4.435517352 1.640449405 -4.991312914<br>S483 3.427563713 1.852794554 -1.339187907<br>S484 1.030668845 4.684595604 2.592521196<br>S485 0.778122619 2.322782581 -3.694452463<br>S486 0.704415348 1.420847411 0.841080308<br>S487 3.934254113 5.527796886 0.294727072<br>S488 -1.960884519 4.566742632 0.354077383<br>S489 3.088628464 4.970903052 -3.323344940<br>S490 0.536411154 7.240925781 0.029665740<br>S491 -0.770126513 5.674009290 -3.067533145

4 35 (S=2)

4 35 (S=2)	bm22b2n2x6h2x2h2n35dte.car_4
Fe(139) 0.550 -2.446<br>Fe(140) 0.402 -0.427<br>Fe(141) 0.548 -2.681<br>Fe(142) 0.558 2.741<br>Fe(143) 0.590 3.115<br>Fe(144) 0.522 2.362<br>Fe(145) 0.317 0.845	C1 -3.186569678 -4.276208561 7.943940583<br>C2 -3.013242353 -3.597170162 9.305515960<br>C3 -2.921215948 -6.341614583 6.647424657<br>C4 -4.297294694 -6.424242863 5.988535940<br>C5 -6.723412118 -6.443356485 6.277610090<br>C6 -7.106301411 -5.163938752 5.523453375<br>C7 -6.728155981 -2.760368503 5.253299116<br>C8 -6.007066489 -2.700355248 3.903963609<br>C9 -4.021466643 -3.269022948 2.611487348<br>C10 -4.660514983 -4.232843814 1.601142831<br>C11 -5.824799823 -6.315973006 1.193178045<br>C12 -7.184399125 -5.843568139 0.662498328<br>C13 -9.405918235 -4.984093062 1.227171404<br>C14 -9.670565340 -3.588672742 0.644565395<br>C15 -9.098452544 -1.195599590 0.717051671<br>C16 -8.075641495 -0.567656302 -0.234060231<br>C17 -5.926766532 -0.912477589 -1.434358394<br>C18 -5.904998185 -1.790993234 -2.696786915<br>C19 -4.553065200 -0.838993575 -0.718546323<br>C20 -3.411127979 -0.616450455 -1.716767079<br>C21 -4.562037403 0.247601043 0.364278469<br>C22 -5.460029176 -4.008861057 -3.660258282<br>C23 -6.846082367 -4.424033173 -4.187784035<br>C24 -4.561194526 -5.219004073 -3.310613514<br>C25 -4.569295049 -6.257521997 -4.438045922<br>C26 -3.129017360 -4.758057146 -3.006071913<br>C27 -9.040808396 -5.331858714 -3.697984889<br>C28 -9.805145916 -4.287216927 -4.486702878<br>C29 -10.369143769 8.309251871 2.545856112<br>C30 -9.107642111 8.432540785 1.684787494<br>C31 -7.967639497 7.460144360 -0.317640160<br>C32 -8.556530598 7.904606347 -1.671824975<br>C33 -7.391806680 6.033262112 -0.386505709<br>C34 -6.273466863 5.868306105 -1.418863870<br>C35 -5.562216557 4.524789763 -1.249315290<br>C36 -4.193018594 3.377611639 -3.006869328<br>C37 -8.264256547 9.369760416 -3.611700920<br>C38 -7.418601336 8.955023197 -4.795916351<br>C39 0.261376373 -2.466708096 5.681310783<br>C40 0.779567265 -3.568820224 4.745080578<br>C41 -0.029160266 -1.219523924 4.814652720<br>C42 -0.839177200 -0.099214599 5.500194317<br>C43 -0.005921723 0.956303242 6.224292609

C44	2.818700410	-4.658063275	3.926045597
C45	2.541555498	-6.160535411	4.056589268
C46	4.335736076	-4.380881156	3.934735552
C47	1.228070328	-7.914262343	5.147934656
C48	0.053269920	-8.160675166	4.190090858
C49	-1.603817741	-7.177601382	2.663025404
C50	-1.322262739	-6.782169511	1.214545113
C51	0.311302512	-5.998792052	-0.443358411
C52	0.507928448	-7.218901976	-1.380329935
C53	1.633425995	-5.221755197	-0.485230612
C54	1.673923445	-3.869851881	0.155237952
C55	0.730654147	-2.961731699	0.571138222
C56	2.730492774	-2.022915392	0.833047713
C57	1.422977913	-9.515529757	-1.485120780
C58	0.271517481	-10.160217746	-2.255154315
C59	2.013601060	-10.502234985	-0.450404571
C60	2.982004265	-11.519208938	-0.978210931
C61	2.946606418	-12.296979600	-2.119013995
C62	4.773583189	-12.732689627	-0.943735440
C63	-3.132047506	5.136318217	-7.828750583
C64	-3.767384570	6.459338224	-7.440286525
C65	-3.044375801	4.287633571	-6.536510183
C66	-2.468988545	2.908627426	-6.762584382
C67	-3.297271123	1.809297605	-7.053515903
C68	-1.082837679	2.682379960	-6.676570505
C69	-2.768134868	0.527970711	-7.244031534
C70	-0.539214198	1.413194937	-6.873447609
C71	-1.383516985	0.334374018	-7.153178576
C72	4.562442783	1.715201296	-7.806070531
C73	5.521236406	0.521274376	-7.737208764
C74	3.554615885	1.652336592	-6.648531189
C75	7.814690051	-0.227427271	-7.277867607
C76	7.704526589	-1.115262895	-6.035624559
C77	6.681609111	-1.415542814	-3.809120478
C78	5.468652314	-2.363217719	-3.948383485
C79	6.554174595	-0.440603717	-2.624376415
C80	6.341232249	-1.043098225	-1.233347456
C81	7.543559327	-1.744882037	-0.576402394
C82	8.527982113	-3.873198448	-1.459993281
C83	3.253975537	-2.786096303	-4.884364352
C84	3.429525821	-3.777504601	-6.053636178
C85	1.949799354	-2.009024621	-5.106963685
C86	4.169617890	-4.013565422	-8.374834390
C87	5.187611469	-5.095127761	-8.728588089
C88	7.182757344	-6.360666745	-8.050931848
C89	6.870888534	-7.856262717	-7.834543718
C90	8.457388876	-5.951098456	-7.280544500
C91	8.266263044	-6.060187686	-5.772021114
C92	5.374645556	-9.502846033	-6.793088471
C93	5.006414299	-10.329454670	-8.019935347
C94	4.185915014	-9.493917309	-5.793128264
C95	4.538445906	-8.808790679	-4.493067563
C96	5.184534694	-9.512445200	-3.465474898
C97	4.294240712	-7.438159669	-4.303932916
C98	5.594653085	-8.869415210	-2.294415011
C99	4.724537589	-6.770528751	-3.152176321
C100	5.378725338	-7.497236144	-2.159610482
C101	4.900877914	8.603938119	3.888522907
C102	4.716692877	7.179780161	4.387327504

	C103	4.613623143	4.792983295	3.705074081
	C104	5.863663188	3.977954605	3.356231863
	C105	7.233520371	3.135025731	1.499200306
	C106	7.790888722	3.621799403	0.161540458
	C107	7.472274072	4.875097791	-1.912315991
	C108	8.437028315	6.046720832	-1.911877765
	C109	5.750943274	3.028385182	7.829150809
	C110	6.191817302	1.582744765	7.562588348
	C111	4.269610797	3.205816186	7.422428778
	C112	3.679870575	4.514285728	7.979182735
	C113	2.319005133	4.770130163	7.368683806
	C114	6.995091643	-0.056189493	5.931593472
	C115	8.228725772	-0.026969363	5.050640484
	C116	5.838993965	-0.941094198	5.402533155
	C117	5.032583596	-0.246105526	4.336753672
	C118	5.463087946	-0.226269525	2.999092125
	C119	3.890353345	0.494233697	4.687830005
	C120	4.797313592	0.564080337	2.055696749
	C121	3.226356482	1.284980980	3.743206703
	C122	3.698168463	1.341080659	2.428520124
	C123	-0.280132021	11.039529321	1.384542366
	C124	-1.252540549	11.611316750	0.341152992
	C125	-0.951575377	9.979208632	2.270913679
	C126	-0.047566664	9.217822481	3.190001505
	C127	0.642249764	9.695554242	4.286686065
	C128	0.883343178	7.496999868	4.163797020
	C129	-3.465710706	12.469817286	-0.197091397
	C130	-2.935691776	13.667572245	-0.952349335
	C131	-3.808139444	3.712490734	4.353896218
	C132	-4.019085352	4.705676846	3.246059703
	C133	-3.159909359	6.024865704	3.429418040
	C134	-3.590029763	6.746106120	4.738520089
	C135	-2.911831062	8.118438981	4.896861709
	C136	-3.513626393	8.957345593	5.987294523
	C137	-3.546027812	6.971470897	2.248611840
	C138	1.076229731	4.028768696	-0.725747647
	Fe139	1.749471697	5.423753591	0.537024056
	Fe140	1.287367503	1.859485320	-1.347822563
	Fe141	0.929256938	4.250495025	-2.759444571
	Fe142	2.994025234	4.056056000	-1.219570797
	Fe143	2.907851200	2.580465359	-3.462951816
	Fe144	-0.328533431	5.438228782	-0.937951644
	Fe145	-0.126147390	3.557415261	0.985140958
	H146	-3.801435466	-1.795956044	8.762769936
	H147	-2.169656253	-1.808269496	8.790568422
	H148	-2.467632305	-6.044017450	8.715089986
	H149	-2.092282288	-3.969812517	9.780919840
	H150	-3.848173150	-3.944184484	9.938981877
	H151	-2.255516114	-5.874089412	5.907447132
	H152	-5.221381939	-6.385983226	7.819309061
	H153	-2.562857144	-7.361965587	6.838861111
	H154	-7.426570233	-6.589311402	7.109015433
	H155	-5.776699100	-4.067923905	6.645923289
	H156	-6.848351262	-7.277980471	5.574438149
	H157	-3.987984927	-2.284571857	2.126694650
	H158	-4.300868649	-3.522776778	4.732149277
	H159	-6.375482742	-1.939517435	5.892095899
	H160	-5.178635599	-6.492667857	0.324440830
	H161	-5.065879289	-5.622086778	3.086929507

	H162	-7.794663849	-2.609339008	5.050651156
	H163	-2.989476584	-3.585145261	2.820334824
	H164	-7.770661644	-5.283813686	2.566855813
	H165	-5.966029407	-7.267743729	1.722102623
	H166	-5.373221196	0.092948474	1.091094177
	H167	-3.605832612	0.245922989	0.910483947
	H168	-4.684960425	1.247319386	-0.080924295
	H169	-4.390807550	-1.812890404	-0.229397109
	H170	-10.022589565	-5.083196451	2.132374962
	H171	-3.094008478	-4.036650882	-2.177501131
	H172	-7.475806580	-5.011750937	-2.288517817
	H173	-5.570688229	-6.680787588	-4.603499562
	H174	-2.452060303	-0.559926132	-1.181662555
	H175	-3.538203579	0.328963798	-2.263937017
	H176	-3.834068923	4.628644805	-8.518398984
	H177	-2.505960740	-5.619297617	-2.723296221
	H178	-3.886106766	-7.084194232	-4.192930938
	H179	-2.677277891	-4.286533341	-3.893914525
	H180	-4.232677784	-5.810687227	-5.386832524
	H181	-4.975176056	-5.692461824	-2.401882135
	H182	-5.000494627	-3.451909705	-4.490582754
	H183	-5.294228770	-3.436081984	-1.593549182
	H184	-3.330066099	-1.430571744	-2.451903308
	H185	1.874912616	-1.215227994	-4.345801490
	H186	1.119539105	-2.718883192	-4.957212164
	H187	0.987856744	-1.266384829	-6.647372460
	H188	-1.484279831	-1.583545053	-7.443453798
	H189	0.533961763	1.247019327	-6.775774925
	H190	-0.417389312	3.507371029	-6.411820495
	H191	-4.379302718	1.951485871	-7.118927735
	H192	-6.919966124	-2.239334655	-0.073760568
	H193	-9.133856997	-0.563755020	1.616964346
	H194	-10.076488816	-1.134879632	0.220635530
	H195	-8.183413782	-2.757898508	1.834080457
	H196	-9.769225175	-5.702897843	0.481992846
	H197	-3.431717022	-0.316291511	-7.449851730
	H198	-6.205294789	0.089790743	-1.785197137
	H199	-8.976941935	-6.251333004	-4.301695877
	H200	-9.634879655	-5.563215120	-2.800013570
	H201	-9.513816895	-3.229801211	-4.286181706
	H202	-8.313390627	10.468380859	-3.570599781
	H203	0.081306157	2.971213442	6.584440396
	H204	-5.313025123	4.501886656	5.263521655
	H205	3.560913033	4.430314855	9.069666331
	H206	4.356901866	5.355821405	7.771102541
	H207	3.666219225	2.358552648	7.784207548
	H208	8.911567995	0.832327601	5.246052065
	H209	5.811632807	3.121574847	8.924035565
	H210	0.929265674	-0.816581354	4.447222329
	H211	-1.528403839	-0.533139590	6.245359830
	H212	6.565756467	2.015474507	5.567230222
	H213	7.352012464	-0.519205676	6.873803322
	H214	6.578184520	4.077484461	6.241826008
	H215	-1.236006455	2.477018671	5.516629213
	H216	4.206602808	3.191852028	6.323205480
	H217	6.533419950	4.917675220	7.658555979
	H218	0.990321502	-2.683486060	7.600423117
	H219	-0.597697153	-1.560332127	3.936947247
	H220	3.171439582	6.059284749	6.147482848

	H221	-1.474675676	0.393115242	4.750448440
	H222	-0.507396899	-5.408799001	-0.878279471
	H223	-0.352402646	-3.007073646	0.581634759
	H224	-1.924397942	-8.228395403	2.657090883
	H225	-0.177827955	-6.115753669	3.842549993
	H226	0.934898555	-8.370803543	0.258365345
	H227	2.240467750	-12.360937293	-2.939537501
	H228	-0.714856726	-10.102022439	-1.731990024
	H229	1.187074326	-10.990235377	0.095774469
	H230	2.197958240	-9.209908979	-2.205327801
	H231	5.718640227	-13.196167108	-0.671267135
	H232	1.899931808	-5.097684762	-1.547510709
	H233	2.423894160	-5.848890837	-0.038784553
	H234	4.386517966	-13.733179697	-2.783222897
	H235	2.565685717	-9.902867988	0.290825341
	H236	0.636905786	-6.312500220	1.666414366
	H237	-2.459433942	-6.581412595	3.013366091
	H238	1.998225441	-8.657297171	4.905017589
	H239	3.497991530	-1.293685652	1.073173404
	H240	0.882005215	-8.111709524	6.170845469
	H241	-1.900472356	5.763184404	-9.338433802
	H242	-1.304653672	4.499996236	-8.493293292
	H243	4.334623664	-0.906410587	-4.898577878
	H244	3.124715024	-3.403816301	-3.984695885
	H245	4.598079405	-2.423390573	-7.056591940
	H246	3.839775654	-3.723652303	0.184933145
	H247	5.327604759	-4.761314822	1.248375887
	H248	5.329305151	-5.459342053	-0.185003368
	H249	4.505840617	-3.318742328	3.712000082
	H250	4.756808786	-4.602576940	4.929713948
	H251	8.728538035	-4.927784418	-7.578837825
	H252	9.263566028	-6.633037477	-7.579004363
	H253	7.964073322	-3.983645925	-5.566267972
	H254	7.641826922	-4.968028525	-4.162507812
	H255	10.369535565	-3.842559831	-2.357623549
	H256	7.503296110	-1.605702726	0.514580682
	H257	6.080962719	-9.436436727	-1.496962368
	H258	3.758713243	-6.857170208	-5.061501158
	H259	7.443811418	-5.627432344	-1.533465121
	H260	5.710699692	0.240586218	-2.818379986
	H261	6.381003642	0.206858639	-5.167411298
	H262	5.472939420	-1.720776140	-1.235514537
	H263	1.497203899	-5.878932338	5.776749098
	H264	8.807978387	0.241354050	-7.270230801
	H265	2.556721804	-3.257579389	5.611306736
	H266	7.465133976	0.180213565	-2.612751510
	H267	4.587319351	-5.691510557	-3.060832741
	H268	4.520957709	-6.016472266	2.878864293
	H269	7.768251882	-0.900585868	-8.145657511
	H270	10.010354082	-2.438185795	-1.410317833
	H271	9.108739930	-5.766295044	-1.964801975
	H272	1.249638127	-1.173551962	6.941434958
	H273	-0.707669933	-2.837288467	6.066535381
	H274	5.371001853	-10.583805807	-3.572513274
	H275	2.442306862	-4.418656053	2.917295877
	H276	7.540505384	-2.084033801	-3.662229201
	H277	8.480451369	-1.281634982	-0.912319895
	H278	6.060530781	-0.199932458	-0.586679004
	H279	6.763935221	-3.721969279	-0.471831694

H280	3.173251110	-4.465211006	-8.498036957
H281	6.071606578	-4.928749725	-6.883658817
H282	7.390575203	-6.269627101	-9.126651927
H283	4.275818309	-3.226890113	-9.134298739
H284	5.158108516	-7.365738559	-6.835804675
H285	5.568439077	8.611953707	3.014086034
H286	3.901663555	8.930683261	3.530830428
H287	0.520204089	10.559766258	0.803346440
H288	5.329956960	10.427957370	4.696207319
H289	4.927166565	9.289119187	5.804869277
H290	2.930985507	2.554787851	-6.690774700
H291	3.385631684	0.832280852	-9.218714806
H292	7.057640404	1.771008589	-7.183745500
H293	2.921322223	0.763116233	-6.778148755
H294	4.479118454	1.871441392	-9.851286523
H295	6.582064925	5.168764028	-2.489403302
H296	5.137869029	2.650397373	-7.725401346
H297	-8.214198585	5.328828292	-0.596143647
H298	-7.174640307	8.151650886	-0.008180826
H299	6.100113200	4.761417851	-0.297280899
H300	-2.820172137	2.580247351	-4.292344669
H301	8.045735530	3.197110175	2.235308035
H302	2.350081137	1.870458581	4.032196066
H303	7.010360290	2.061682657	1.368877815
H304	-6.251924171	3.685491717	-1.407571818
H305	5.316458224	4.159811636	1.403353022
H306	-5.184059412	4.431607742	-0.218553827
H307	-7.004186799	5.802951750	0.618781782
H308	-5.528212732	6.668876452	-1.300205944
H309	-6.673837754	5.944167100	-2.442241677
H310	5.199483142	-1.197257996	6.259777135
H311	-9.841182726	6.919852344	0.603286498
H312	-10.031977126	7.939806275	3.526352979
H313	3.218383682	1.972926781	1.681120152
H314	-12.046684433	7.885156056	1.423889151
H315	-11.889744013	6.938457954	2.752828452
H316	-3.588993626	4.945679075	-1.902810480
H317	5.124610343	0.602129796	1.014886729
H318	3.523167249	0.460326298	5.716027761
H319	-5.102331315	11.156740823	2.745831627
H320	-1.726516194	10.471485994	2.885052536
H321	-5.162866477	8.964373970	1.674952873
H322	-4.521808783	11.293121821	4.169165448
H323	8.601723581	6.530605287	-0.918724979
H324	-3.042956107	7.634353964	-0.269179264
H325	-5.080265428	4.980144629	3.148341654
H326	4.889315622	6.483950152	2.469128473
H327	-4.683525302	6.879667478	4.718668345
H328	1.786688389	8.594563304	5.728808760
H329	1.177070772	6.488435797	4.426694954
H330	-10.736155061	9.337069410	2.705345177
H331	-2.184813145	3.812779185	-3.225285124
H332	-4.577806303	9.080542433	-1.405574415
H333	-2.492617989	7.557224049	-1.720251189
H334	-6.914310253	9.156645968	-1.947791297
H335	-6.160370925	2.872281186	-3.315105800
H336	-3.825984239	8.999599682	7.852723873
H337	-1.844144507	7.963173724	5.109793783
H338	-2.990764211	8.701239557	3.972938822

	H339	-3.327097096	6.113191576	5.598848921
	H340	3.748417544	4.453992943	3.114098574
	H341	6.343531776	-0.806126338	2.711806951
	H342	6.292258414	-1.871321911	5.026216649
	H343	-3.681046219	4.243427013	2.308766445
	H344	-5.397165644	9.867234002	-0.341875133
	H345	-6.544437938	9.680180983	1.617902288
	H346	-5.021479857	1.845915469	-4.081685613
	H347	0.759066234	10.699697934	4.677557362
	H348	-1.474235887	9.257029004	1.632371795
	H349	4.402457753	4.660056870	4.767283423
	H350	7.949110601	4.035570793	-2.440564997
	H351	-4.064723015	4.207686981	-6.132272756
	H352	-2.429410236	4.830315218	-5.800204607
	H353	0.820495301	12.763477523	1.565891847
	H354	-0.401635538	12.660647207	2.648812383
	H355	-4.346526011	12.809679935	0.370779686
	H356	-2.841629807	11.725677414	1.696341301
	H357	-2.157217564	14.262678850	-0.418135629
	H358	-3.806875014	11.704628609	-0.912952319
	H359	-3.043207434	7.228410664	-7.074657444
	H360	4.675014489	-9.737696675	-8.905449690
	H361	-9.284070320	8.990093795	-3.796299006
	H362	-6.842666764	8.006614765	-4.663496183
	H363	6.242975997	-9.989234646	-6.319260230
	H364	3.322160601	-8.999829219	-6.265803482
	H365	3.904249158	-10.542314363	-5.615454738
	H366	-0.898102972	3.121916575	2.443079184
	H367	6.234873399	-7.455782917	-0.412034822
	H368	-1.914476145	1.700190587	-1.352282467
	H369	-1.329419446	2.751597637	1.857368098
	H370	-1.843635223	2.186119363	-0.782441095
	H371	-0.436252171	0.767442835	0.646045134
	H372	1.021970172	-0.909080835	1.229564778
	Mo373	-0.678076258	6.101019197	1.690770332
	N374	-2.996492073	-2.142757344	9.291748060
	N375	-2.897417464	-5.607189737	7.903750442
	N376	-5.371428953	-6.426678094	6.814154851
	N377	-6.529618661	-4.016097410	5.960243881
	N378	-4.742385274	-3.194256874	3.873642305
	N379	-5.193952738	-5.372863992	2.102665551
	N380	-8.030827244	-5.302037268	1.577299866
	N381	-8.869196861	-2.580561749	1.094146376
	N382	-6.994298397	-1.333856231	-0.533160147
	N383	-5.572339800	-3.100626812	-2.517869387
	N384	-7.741012028	-4.860013624	-3.264178196
	N385	-11.366355716	7.383801242	1.998398919
	N386	-9.019551198	7.528388099	0.689970435
	N387	-4.410136449	4.384016913	-2.147788420
	N388	-5.200673304	2.545031131	-3.366736316
	N389	-2.956177242	3.154539571	-3.463666826
	N390	-7.815286797	8.816687054	-2.348950239
	N391	1.236905991	-2.190064893	6.740327345
	N392	-0.429759310	2.229049245	6.095047875
	N393	2.107330902	-3.829418666	4.877597150
	N394	1.787737290	-6.574872735	5.093425289
	N395	-0.486083255	-7.062068602	3.584654483
	N396	-0.050114776	-6.371287444	0.917849264
	N397	2.907926838	-3.255201051	0.340804517

	N398	1.416617989	-1.836447423	0.990738921
	N399	0.936078555	-8.347518558	-0.758156639
	N400	4.127997858	-11.802121735	-0.251412256
	N401	4.096089713	-13.058296034	-2.080627225
	N402	-1.804778234	5.387267211	-8.391739352
	N403	3.820003216	1.750411116	-9.076474695
	N404	6.810462155	0.809094225	-7.400367577
	N405	6.942520928	-0.647833851	-5.023647419
	N406	7.582116562	-3.192052407	-0.819514617
	N407	9.692230542	-3.305864895	-1.826502698
	N408	8.306396504	-5.159522295	-1.819435778
	N409	4.407782889	-1.915299874	-4.671262337
	N410	4.266533444	-3.393219221	-7.060743029
	N411	6.069836344	-5.463519218	-7.753847836
	N412	7.838217441	-4.920946885	-5.162046889
	N413	5.726100747	-8.141916505	-7.166761268
	N414	5.465740170	9.446274213	4.947942602
	N415	4.824514796	6.213412266	3.455703540
	N416	6.078688753	3.835709860	2.033390592
	N417	7.046206203	4.475000551	-0.584837075
	N418	6.701134469	3.991889552	7.258792338
	N419	6.608107197	1.295741730	6.296532522
	N420	0.332676714	12.113339611	2.189002869
	N421	0.122322944	7.836101976	3.127008690
	N422	1.221189388	8.596238331	4.884076894
	N423	-2.505082120	11.945779101	0.759565582
	N424	1.004060562	-0.994207289	-1.884656260
	N425	1.112949568	0.116342176	-1.665991144
	O426	-3.614469023	-3.673286168	6.941468281
	O427	-4.401536648	-6.488847121	4.752464436
	O428	-7.921571422	-5.198970325	4.586979299
	O429	-6.555680075	-2.236037368	2.892331554
	O430	-4.683177445	-3.972850589	0.387414728
	O431	-7.492075357	-5.994790231	-0.532002587
	O432	-10.603365351	-3.423607233	-0.152985080
	O433	-8.263968027	0.579119221	-0.664945158
	O434	-6.167759461	-1.314577477	-3.810796974
	O435	-7.128975859	-4.367024046	-5.395482613
	O436	-10.721309313	-4.565670383	-5.246049471
	O437	-8.262735135	9.312662004	1.954672496
	O438	-9.634473089	7.435732462	-2.077029716
	O439	-7.372904104	9.589162531	-5.837866186
	O440	0.045919070	-4.161610638	3.927310336
	O441	0.996106597	0.652244788	6.910130535
	O442	2.976574589	-6.932005929	3.172558506
	O443	5.009570366	-5.154530669	2.928694030
	O444	-0.351351458	-9.316070989	4.014738917
	O445	-2.212690831	-6.848027220	0.363819656
	O446	0.384580724	-7.112224598	-2.602459721
	O447	0.391442529	-10.722586570	-3.329469642
	O448	-4.968394864	6.684022044	-7.490859053
	O449	-0.795490524	-0.905505008	-7.314332975
	O450	5.153912987	-0.641553440	-7.985277441
	O451	8.337257700	-2.191334358	-6.003064329
	O452	5.508387831	-3.489659076	-3.421765523
	O453	2.791127372	-4.839408196	-6.068816405
	O454	1.921837237	-1.467415028	-6.430793226
	O455	5.177375797	-5.603261484	-9.857150612
	O456	7.633284946	-8.729909243	-8.270256533

	O457	8.399263671	-7.137870987	-5.170850602
	O458	5.036584753	-11.549552554	-8.033408930
	O459	5.849392011	-6.805363498	-1.030160833
	O460	4.449990622	6.963987339	5.597733130
	O461	6.619906929	3.516448686	4.239364114
	O462	8.892709999	3.181985342	-0.209696978
	O463	8.989028918	6.452907000	-2.921997642
	O464	6.149570923	0.719496425	8.455284360
	O465	1.299052832	4.183946758	7.720144590
	O466	2.270321845	5.656112269	6.355656958
	O467	8.511315736	-0.879694577	4.220733447
	O468	-0.893142184	11.838278444	-0.828591702
	O469	-3.375548416	14.022835053	-2.037028849
	O470	-2.931673797	2.857452765	4.384678390
	O471	-4.664030468	3.789575363	5.421301783
	O472	-4.060469581	10.046650958	5.833347138
	O473	-3.402304964	8.385274279	7.215876104
	O474	-2.568333969	7.339457335	1.487105561
	O475	-4.730031941	7.363675079	2.123734072
	O476	-1.812907196	5.732443584	3.429806361
	O477	-5.318614149	9.732924855	-1.322970454
	O478	-3.307851286	7.747830077	-1.216989449
	O479	5.322493380	-4.579608489	0.268027401
	O480	-4.765888814	11.831373227	3.388303354
	O481	-5.578314663	9.850685502	1.468432053
	S482	4.350608716	1.540287232	-4.978697411
	S483	3.596727715	1.867227051	-1.340751703
	S484	1.046687323	4.678990755	2.537823331
	S485	0.682728280	2.182385329	-3.556333425
	S486	0.743318301	1.428351275	0.864533717
	S487	3.947382573	5.513614296	0.213635435
	S488	-1.975976305	4.629278359	0.321051326
	S489	2.988856631	4.864949803	-3.377921985
	S490	0.549252135	7.270455994	0.007502862
	S491	-0.797056813	5.612715923	-3.090349990

TS 4 → 6 35 (S=2)

TS 4 → 6 35 (S=2)	bm22b2n2x6h2x2h2nd235th_1_53243.077			
Fe(139) -2.439	C1	-9.164963282	0.010245189	-2.609401038
Fe(140) -0.976	C2	-9.919553556	-1.322006684	-2.652927275
Fe(141) -2.442	C3	-8.684872281	2.123547100	-3.762399486
Fe(142) 2.851	C4	-8.918334024	3.106521560	-2.615683810
Fe(143) 2.938	C5	-10.457636334	3.951294753	-0.914526858
Fe(144) 2.361	C6	-9.683684767	3.609290167	0.362668993
Fe(145) 1.245	C7	-8.547630282	1.871844006	1.658732280
	C8	-7.081186183	2.295614631	1.522431843
	C9	-5.157954164	2.585387040	0.049937870
	C10	-4.973783490	4.104224785	0.195415782
	C11	-5.881693468	6.327382072	-0.153267038
	C12	-6.089752052	6.854750032	1.273355631
	C13	-7.533431256	6.866258031	3.251781674
	C14	-6.840355065	6.297124672	4.495776682
	C15	-5.894163292	4.304419607	5.586391561
	C16	-4.409413661	3.934894260	5.522396420
	C17	-2.379885998	3.910452893	4.085763369
	C18	-1.597288377	5.217363053	3.851754065
	C19	-2.209386297	2.872176772	2.950893341
	C20	-0.736088211	2.720157248	2.563659764

	C21	-2.827744564	1.528332656	3.353263632
	C22	-1.226229358	7.178208187	2.413062526
	C23	-1.692042385	8.343142494	3.305460818
	C24	-1.340525582	7.499273809	0.902639176
	C25	-0.747574009	8.877066011	0.583091627
	C26	-0.653662774	6.411461602	0.065711015
	C27	-3.526258139	9.663135866	4.182109198
	C28	-3.003794320	9.657244568	5.605004216
	C29	-5.349091956	-2.988081348	12.052476773
	C30	-3.935607468	-3.122212730	11.484184652
	C31	-2.035816405	-1.788852224	10.562190613
	C32	-1.169937267	-1.118435157	11.647230776
	C33	-2.068836263	-0.957692818	9.268711177
	C34	-0.684767087	-0.752615617	8.650231434
	C35	-0.782235183	-0.211018231	7.224856895
	C36	1.060263990	0.952281637	5.988687537
	C37	0.923971118	-1.249583205	12.915334681
	C38	2.186143643	-0.627896854	12.354843298
	C39	-4.977029579	-1.442199545	-3.306018252
	C40	-4.255575473	-0.364614236	-4.131451968
	C41	-4.086847679	-1.758282778	-2.081164730
	C42	-4.745629194	-2.585795539	-0.957863392
	C43	-4.595578223	-4.100568790	-1.100736073
	C44	-2.825049900	-0.012379200	-6.092310338
	C45	-3.511182859	1.104647438	-6.886416953
	C46	-1.939321943	-0.866704411	-7.021700445
	C47	-5.585728393	2.318082592	-7.334845065
	C48	-5.546739847	3.527498531	-6.388894456
	C49	-5.010907026	4.364791286	-4.138578648
	C50	-3.612107399	4.785051448	-3.689094876
	C51	-1.183523502	4.452703665	-3.846858830
	C52	-0.716101237	5.760713672	-4.532498618
	C53	-0.165722827	3.386204960	-4.279039763
	C54	-0.244903494	2.038056703	-3.634216625
	C55	-0.751103245	1.563002405	-2.449072994
	C56	0.285308296	-0.122828315	-3.458266674
	C57	-0.754222919	6.994211283	-6.673011598
	C58	-0.969378939	8.379664393	-6.071093397
	C59	-1.527321415	6.850699625	-8.006494434
	C60	-0.884404385	7.458396949	-9.218966940
	C61	-0.313522624	8.701525782	-9.411862089
	C62	-0.226694017	7.513935165	-11.281052284
	C63	5.840690357	2.047901791	7.641599123
	C64	5.567364467	1.183037968	8.859861701
	C65	4.635292573	1.871162186	6.688213473
	C66	4.717012786	2.747895692	5.460259301
	C67	4.185234893	4.052111702	5.450459137
	C68	5.336284181	2.281937481	4.285760180
	C69	4.258781569	4.860073804	4.310941709
	C70	5.428721369	3.080875407	3.145851987
	C71	4.887644011	4.370034005	3.158016324
	C72	9.131503025	1.165599557	-0.041873997
	C73	9.271620833	1.571700493	-1.514278573
	C74	7.648665215	0.973991938	0.317074449
	C75	9.946573200	0.867974184	-3.768273629
	C76	8.655866841	0.869067830	-4.589066080
	C77	6.256094739	0.294999751	-4.649297721
	C78	5.440765765	1.570780549	-4.342467133
	C79	5.516852344	-0.997654574	-4.255455646

	C80	4.149633947	-1.255108665	-4.897456716
	C81	4.134707830	-1.604773204	-6.396347200
	C82	4.696971931	-0.000459279	-8.235931815
	C83	4.870687870	3.345232689	-2.762810850
	C84	5.609867277	4.623071821	-3.206208280
	C85	4.556212308	3.469896698	-1.265074068
	C86	7.775913649	5.756012964	-3.269393989
	C87	8.339601342	6.269827510	-4.591304709
	C88	8.483419913	5.980843038	-7.032438572
	C89	7.705214304	7.067200732	-7.806065927
	C90	8.672048465	4.718871165	-7.906668477
	C91	7.340553120	4.045361782	-8.216699127
	C92	5.599275199	8.313302339	-8.012704300
	C93	6.126371344	9.743280582	-7.951814916
	C94	4.170905481	8.244875828	-7.410316599
	C95	3.535799546	6.886499223	-7.597434774
	C96	2.912196600	6.555471068	-8.811803205
	C97	3.600284732	5.905765127	-6.594497238
	C98	2.385470174	5.280939278	-9.028896134
	C99	3.098853854	4.616377579	-6.801982880
	C100	2.504836015	4.318257619	-8.027206774
	C101	2.062655420	-10.418371198	0.820252783
	C102	1.168966495	-9.602367035	-0.099258125
	C103	0.967341645	-7.468964967	-1.353594675
	C104	1.717745341	-7.182775220	-2.658698306
	C105	3.691504947	-6.111368005	-3.638440707
	C106	5.183569623	-5.939978472	-3.366167809
	C107	7.005390214	-5.615792118	-1.769344370
	C108	7.866493497	-6.863955929	-1.756117583
	C109	-2.129132373	-8.919276931	-4.389805929
	C110	-2.086194150	-7.915984738	-5.549813292
	C111	-2.587346555	-8.196377186	-3.101207994
	C112	-3.007026094	-9.201049546	-2.012008215
	C113	-3.182301332	-8.505329212	-0.679991251
	C114	-0.827311330	-6.167224272	-6.711093973
	C115	0.557053961	-6.220793566	-7.329131398
	C116	-1.292560294	-4.750323982	-6.289780068
	C117	-0.722403160	-4.327774208	-4.959346859
	C118	0.575575248	-3.797125444	-4.856755321
	C119	-1.446393392	-4.565663462	-3.777220132
	C120	1.149485422	-3.568477189	-3.601752608
	C121	-0.871584597	-4.336682909	-2.523182096
	C122	0.438271246	-3.857824735	-2.433054880
	C123	1.822507441	-8.537347205	6.824131592
	C124	2.268174656	-7.968497993	8.181233119
	C125	0.444128840	-8.008167862	6.395456609
	C126	0.023583734	-8.340292324	4.997235010
	C127	-0.315963018	-9.581536594	4.497542610
	C128	-0.690801165	-8.036327698	2.955901188
	C129	1.737685874	-7.415715413	10.491237317
	C130	2.970293373	-8.098470033	11.041069060
	C131	-4.592292156	-3.496892535	3.973745706
	C132	-3.481271301	-3.465684783	4.986633022
	C133	-2.759370510	-4.864231244	5.152505039
	C134	-3.811458229	-5.933297361	5.571037276
	C135	-3.174813322	-7.290186775	5.912934250
	C136	-4.126243914	-8.257891279	6.559685838
	C137	-1.769652773	-4.719003622	6.351830972
	C138	2.330315452	-2.976074819	2.000160728

	Fe139	2.084565518	-4.983044500	2.070528836
	Fe140	2.472796166	-1.148440456	0.588812535
	Fe141	3.952077001	-2.051859328	2.771076995
	Fe142	3.758308151	-3.605983742	0.734769794
	Fe143	5.116650275	-1.276957649	0.496284077
	Fe144	2.083909458	-3.255926101	3.961794228
	Fe145	0.282508766	-3.031801275	2.031268102
	H146	-9.391981191	-1.952107748	-0.787267482
	H147	-8.510008078	-2.637883343	-1.976855011
	H148	-9.966697006	0.545833210	-4.428216765
	H149	-9.897125066	-1.718089886	-3.680201158
	H150	-10.978086228	-1.090892384	-2.440202188
	H151	-7.599972004	1.948914894	-3.800819276
	H152	-10.844161897	2.439010184	-2.395997550
	H153	-8.991648669	2.605110784	-4.701553231
	H154	-11.533056100	3.874196754	-0.706896806
	H155	-9.528912656	1.645338253	-0.221978220
	H156	-10.228087941	4.997077105	-1.157183965
	H157	-4.464716242	2.116049911	0.760129828
	H158	-7.050937418	1.696223985	-0.457933991
	H159	-8.607117739	0.777746214	1.734082888
	H160	-4.890919677	6.663107095	-0.483102509
	H161	-6.767157255	4.466105689	-0.774289242
	H162	-8.917986074	2.307858236	2.594432315
	H163	-4.873875178	2.277319876	-0.966861033
	H164	-7.752849627	5.645873356	1.514906300
	H165	-6.640265122	6.779993552	-0.807180649
	H166	-3.893507058	1.636662079	3.600902115
	H167	-2.740665392	0.804877274	2.527124713
	H168	-2.310664649	1.098637148	4.225134536
	H169	-2.757863356	3.252957280	2.075022895
	H170	-8.612379184	6.692883460	3.369954428
	H171	-1.100787223	5.420225513	0.225914243
	H172	-3.698177792	8.014882353	2.838969654
	H173	-1.269872056	9.688949283	1.108784018
	H174	-0.629765292	1.994170791	1.745031929
	H175	-0.141589423	2.348091054	3.410120783
	H176	5.847364375	3.102767824	7.979901473
	H177	-0.740090522	6.650787760	-1.004009242
	H178	-0.818639815	9.070706501	-0.497131306
	H179	0.417794383	6.353308338	0.316784666
	H180	0.315715410	8.918695228	0.866876379
	H181	-2.414590828	7.515079981	0.642922937
	H182	-0.164674796	7.042866386	2.670930493
	H183	-2.601149173	5.560821934	2.066858161
	H184	-0.294113981	3.669101644	2.225950702
	H185	4.154851667	2.507590239	-0.909342036
	H186	3.770806090	4.234541395	-1.162130013
	H187	5.435979437	4.262618382	0.297851728
	H188	4.541362597	5.967839747	2.110103941
	H189	5.893027613	2.696698152	2.236809548
	H190	5.723465636	1.261864266	4.248649119
	H191	3.695650944	4.444487498	6.345841179
	H192	-4.349636445	4.603703810	3.600196035
	H193	-6.448797064	3.370097125	5.759725119
	H194	-6.033297919	4.949679139	6.463663270
	H195	-6.678664218	4.424059713	3.611772577
	H196	-7.354102097	7.948984498	3.265065338
	H197	3.820454378	5.861312343	4.315210377

H198	-1.978735319	3.496154195	5.020935624
H199	-3.287151524	10.634699490	3.720187880
H200	-4.622785354	9.575776567	4.240083816
H201	-2.739944959	8.651198873	6.008091625
H202	1.196138836	-2.052750022	13.616864861
H203	-4.311318689	-5.806484211	-0.002911967
H204	-5.820828593	-3.811706493	5.428275398
H205	-3.966946127	-9.658766004	-2.292108540
H206	-2.257749082	-10.001113221	-1.923148006
H207	-3.436804904	-7.529975104	-3.317029022
H208	1.026113996	-7.232173807	-7.315108537
H209	-2.926169564	-9.620854998	-4.680052279
H210	-3.172903863	-2.264032713	-2.433239553
H211	-5.826707289	-2.369316017	-0.910295100
H212	-0.188142454	-7.283003210	-4.993294387
H213	-1.499876368	-6.502020094	-7.526107250
H214	-0.122733232	-9.132084666	-3.896017529
H215	-4.336503936	-4.323424992	0.951551733
H216	-1.766985788	-7.554428172	-2.746113272
H217	-1.021135979	-10.496194244	-3.680954289
H218	-6.195801687	-2.626288917	-4.479099303
H219	-3.773317228	-0.795407840	-1.652314787
H220	-1.387135641	-9.140711547	-0.171149013
H221	-4.331174214	-2.268970980	0.009768947
H222	-1.128722226	4.635665255	-2.764602788
H223	-1.303783640	2.058837143	-1.659909572
H224	-5.486561894	5.254946524	-4.571989311
H225	-4.856872182	2.347071280	-4.814766705
H226	-2.020614638	5.377179530	-6.065042496
H227	-0.150961005	9.546099692	-8.751609749
H228	-1.919571767	8.478154816	-5.491245734
H229	-2.554056452	7.233412341	-7.868109536
H230	0.324062355	6.848009956	-6.841280218
H231	-0.013832545	7.265497366	-12.317400629
H232	0.831818993	3.809360485	-4.075316730
H233	-0.228120893	3.260863590	-5.372808079
H234	0.567389655	9.487418395	-11.194841276
H235	-1.622175352	5.774039462	-8.215210448
H236	-2.730644739	3.278001284	-4.790312480
H237	-5.588014566	4.096042871	-3.242226630
H238	-5.172671485	2.652968567	-8.295068313
H239	0.686034004	-1.114235514	-3.653741049
H240	-6.635163349	2.040235016	-7.498885683
H241	7.884666369	1.936853526	7.553323649
H242	7.172340104	2.056729324	6.087983594
H243	6.000861506	1.542185415	-2.346230194
H244	3.914296817	3.346068959	-3.305309292
H245	7.423151279	3.702939783	-2.930113275
H246	0.866524592	0.984475415	-5.182290301
H247	0.608984906	0.480219193	-7.187530730
H248	1.524267733	1.784343480	-7.231251218
H249	-1.388997367	-1.601156989	-6.417961749
H250	-2.566911840	-1.405751500	-7.750908118
H251	9.359879936	4.038244253	-7.384448696
H252	9.132260976	5.033249127	-8.852501243
H253	7.631261743	2.573830547	-6.734968721
H254	6.057705429	2.636275592	-7.483738279
H255	6.354306458	-0.332759321	-9.392908813
H256	3.328795952	-2.325179667	-6.603006992

	H257	1.896914984	5.038252069	-9.975215368
	H258	4.045267775	6.133627073	-5.621503836
	H259	3.668410055	1.770757326	-8.505997440
	H260	5.371875397	-1.006914372	-3.163603093
	H261	7.618113503	0.003782547	-3.027914317
	H262	3.465031127	-0.413903973	-4.707179885
	H263	-5.351116146	0.416890803	-6.364829754
	H264	10.611491388	0.100788958	-4.188315835
	H265	-3.917122705	-1.823411229	-5.461971890
	H266	6.188940704	-1.839581421	-4.490831876
	H267	3.216415593	3.848529750	-6.035126073
	H268	-1.451778614	0.795186166	-7.922998888
	H269	10.418063943	1.847195670	-3.931721175
	H270	5.825552769	-1.703183539	-8.483623484
	H271	4.846878047	1.405043656	-9.713494830
	H272	-5.103522825	-3.481250864	-3.550130049
	H273	-5.900865233	-0.960553761	-2.935209336
	H274	2.832608474	7.302747201	-9.604554385
	H275	-2.157633540	0.528952694	-5.400522588
	H276	6.426272784	0.321208624	-5.734260512
	H277	5.075658405	-2.094840015	-6.675857581
	H278	3.729032951	-2.114459500	-4.358122451
	H279	3.036837936	0.093122335	-7.076093006
	H280	7.177258702	6.578058445	-2.848886263
	H281	7.349484401	4.802401356	-5.630072632
	H282	9.475187483	6.419817811	-6.848111611
	H283	8.633155047	5.590818226	-2.603296283
	H284	6.165298548	6.928341928	-6.471416962
	H285	3.118763746	-10.216359341	0.587863876
	H286	1.879110567	-10.025172729	1.841427221
	H287	2.576106613	-8.189027898	6.103081361
	H288	2.211818940	-12.350180550	1.450988181
	H289	0.778124114	-11.995725398	0.740189902
	H290	7.594151426	0.635863058	1.359355399
	H291	9.335610592	3.086603874	0.613903438
	H292	9.999964433	-0.294925063	-1.974164471
	H293	7.130773436	1.939974730	0.222095786
	H294	10.724950203	2.229036083	0.691363217
	H295	7.057192399	-5.147229140	-0.774135873
	H296	9.658776136	0.212914838	0.121949945
	H297	-2.545712763	0.014666238	9.476217072
	H298	-1.642664106	-2.788327235	10.339061255
	H299	4.956404300	-5.888342629	-1.289094075
	H300	2.611565660	1.610856230	4.836126001
	H301	3.583237336	-6.897155997	-4.396848507
	H302	-1.438335742	-4.543208797	-1.612685813
	H303	3.357315796	-5.173126211	-4.113844647
	H304	-1.264750526	0.774737006	7.204994415
	H305	3.005230901	-6.036026480	-1.571778027
	H306	-1.405040427	-0.883015086	6.611147497
	H307	-2.714876805	-1.498144536	8.557885717
	H308	-0.143891984	-1.710282608	8.611090201
	H309	-0.082084414	-0.062169614	9.261718526
	H310	-2.391095902	-4.764763025	-6.243014743
	H311	-3.958792752	-1.133367043	11.278204284
	H312	-6.021066061	-3.429252472	11.299842086
	H313	0.912705433	-3.706591967	-1.462865383
	H314	-5.508911713	-1.330071569	13.261787062
	H315	-6.763791869	-1.504952589	12.223913520

	H316	0.944081451	-1.028782358	6.315033007
	H317	2.164403144	-3.178028693	-3.507633651
	H318	-2.468247841	-4.944986218	-3.837251614
	H319	-1.841905171	-7.389152086	10.001881657
	H320	-0.315991531	-8.418745249	7.084192371
	H321	-1.649986214	-5.176750107	8.963781002
	H322	-2.597087195	-8.530020766	9.285818272
	H323	7.317362795	-7.825069792	-1.910201260
	H324	0.655593608	-3.998764082	7.126250828
	H325	-3.851817409	-3.135776219	5.968493550
	H326	2.563128095	-8.098824698	-0.119810353
	H327	-4.354948414	-5.555183237	6.451226921
	H328	-1.117911201	-10.071594054	2.571349696
	H329	-1.008621735	-7.588710512	2.021953856
	H330	-5.391702503	-3.648109053	12.936224942
	H331	2.475042499	-0.137320553	4.935447463
	H332	1.122670151	-3.816285178	9.468160563
	H333	2.104390323	-3.442465029	7.110377847
	H334	0.287059171	-2.576825947	11.346155043
	H335	0.058668838	2.364112775	7.095091755
	H336	-5.754568630	-9.179523134	6.281762590
	H337	-2.783562296	-7.741741956	4.989157159
	H338	-2.338830231	-7.162734249	6.609794261
	H339	-4.529588698	-6.067160223	4.748532516
	H340	0.818905372	-6.542511785	-0.775720181
	H341	1.145423002	-3.601982396	-5.768959555
	H342	-0.992665584	-4.062101141	-7.094606596
	H343	-2.712641007	-2.763777641	4.636651571
	H344	0.063456983	-4.627814097	10.265134322
	H345	-2.135288977	-5.080979677	10.443814407
	H346	1.028203537	2.993052466	5.830401032
	H347	-0.285065154	-10.567144141	4.947382604
	H348	0.423153645	-6.917602167	6.516147564
	H349	-0.003863481	-7.897971632	-1.608736014
	H350	7.436047928	-4.906932311	-2.493011536
	H351	3.727334556	2.108825037	7.262911612
	H352	4.582203531	0.811626735	6.391117965
	H353	2.812454723	-10.321012816	7.041900588
	H354	1.270089005	-10.369638786	7.584933306
	H355	0.911475541	-7.621829881	11.190076162
	H356	0.373358856	-8.181755128	9.045828807
	H357	3.141966132	-9.135201864	10.665699986
	H358	1.895332003	-6.325330945	10.473142285
	H359	5.903884500	0.123714578	8.754747867
	H360	6.883398448	9.935960280	-7.156262933
	H361	0.402442528	-0.462059922	13.486810278
	H362	2.092228886	-0.208982259	11.323589738
	H363	5.552162679	8.030984499	-9.077486548
	H364	4.216980362	8.504000941	-6.340713890
	H365	3.571860882	9.021852487	-7.907224485
	H366	-1.847562065	-3.209326712	1.842171541
	H367	1.575186040	2.975138967	-9.092234235
	H368	0.960095723	0.397993890	2.720012220
	H369	-1.712523541	-2.486627533	2.051301569
	H370	0.564095723	-0.226006110	2.857012220
	H371	-0.432343316	-0.561260778	0.821082596
	H372	-0.505552935	-0.411934759	-1.550665807
	Mo373	-0.007602423	-4.977281546	3.874499234
	N374	-9.458262762	-2.345053510	-1.729639267

	N375	-9.413701644	0.869109053	-3.637947203
	N376	-10.144809620	3.085932375	-2.040390888
	N377	-9.377497408	2.300671514	0.543997155
	N378	-6.521524292	2.139089325	0.293404810
	N379	-5.981207582	4.881699357	-0.268875425
	N380	-7.129323001	6.320971502	1.965558534
	N381	-6.439642948	4.994658406	4.428252802
	N382	-3.784115143	4.209214484	4.348960895
	N383	-1.926349903	5.933745769	2.737706931
	N384	-3.031198265	8.539599838	3.409016292
	N385	-5.750640417	-1.600530998	12.306229100
	N386	-3.385132571	-1.960491157	11.083398241
	N387	0.532882331	-0.128927665	6.582158448
	N388	0.550933148	2.188069570	6.225026160
	N389	2.063473553	0.804165944	5.122951413
	N390	0.014096757	-1.732704740	11.894616144
	N391	-5.236812649	-2.630206704	-4.125977770
	N392	-4.398317189	-4.786291528	0.042258006
	N393	-3.724371274	-0.822638714	-5.296479269
	N394	-4.857788997	1.153046638	-6.864642087
	N395	-5.082102277	3.300256530	-5.124851998
	N396	-2.550450685	4.087927157	-4.202091803
	N397	0.392166871	0.959931982	-4.239385753
	N398	-0.416949442	0.223868699	-2.375662054
	N399	-1.262957076	5.981198141	-5.756423519
	N400	-0.824009837	6.726282320	-10.394273374
	N401	0.100941537	8.714903082	-10.727219675
	N402	7.084544093	1.610461874	7.005309035
	N403	9.716912956	2.167422374	0.861751422
	N404	9.789937054	0.626855780	-2.348156947
	N405	7.571915182	0.301121870	-4.015973726
	N406	3.905222162	-0.439074359	-7.260113426
	N407	5.766388191	-0.705421045	-8.654111043
	N408	4.481889810	1.218448936	-8.783456403
	N409	5.574344944	2.109067535	-3.099078791
	N410	6.969211822	4.544416552	-3.299961545
	N411	7.913892480	5.647740692	-5.730399301
	N412	6.969636917	3.069097480	-7.345325055
	N413	6.486276540	7.396974776	-7.314868403
	N414	1.789015765	-11.850061808	0.665975675
	N415	1.685359351	-8.435301528	-0.530524043
	N416	2.837096390	-6.446208246	-2.513036093
	N417	5.612558547	-5.880510983	-2.081089472
	N418	-0.884480093	-9.692841673	-4.298191177
	N419	-0.947025477	-7.179348962	-5.676181039
	N420	1.860010383	-10.013041293	6.826287622
	N421	-0.211150657	-7.382675992	4.010956763
	N422	-0.763026995	-9.366741476	3.211417312
	N423	1.354102190	-7.942634894	9.191419045
	N424	1.883792405	1.446977152	-0.617674702
	N425	2.147322625	0.418979728	-0.213968558
	O426	-8.406139725	0.321136666	-1.672600793
	O427	-8.018431625	3.883168282	-2.255261835
	O428	-9.393815752	4.494418335	1.184591598
	O429	-6.448600028	2.765114139	2.480504624
	O430	-3.946834556	4.580340768	0.705407008
	O431	-5.362981227	7.751548896	1.734701903
	O432	-6.712128039	7.004820676	5.503354882
	O433	-3.866174291	3.404387783	6.501961347

O434	-0.720785810	5.586913052	4.647021219
O435	-0.879681708	9.073031057	3.898115200
O436	-2.935866186	10.661590304	6.297223582
O437	-3.403023011	-4.250255727	11.411693572
O438	-1.564697972	-0.094934750	12.232867741
O439	3.234290743	-0.562503207	12.976805926
O440	-4.155887001	0.822503184	-3.758860930
O441	-4.665513361	-4.667780146	-2.213903553
O442	-2.805536021	1.947138558	-7.483463233
O443	-0.978019504	-0.049770963	-7.709991589
O444	-5.933211337	4.628968920	-6.796809861
O445	-3.467696006	5.727746170	-2.906430420
O446	0.167039731	6.467180063	-4.039845813
O447	-0.210332333	9.321547980	-6.222470352
O448	5.004686687	1.578731694	9.870777176
O449	4.991794819	5.110451218	1.997335868
O450	8.944178372	2.699609373	-1.928121070
O451	8.670384406	1.352706036	-5.739902493
O452	4.718102997	2.065888560	-5.225297291
O453	4.974250196	5.667811185	-3.407679391
O454	5.729991421	3.846718421	-0.538680788
O455	9.122866071	7.228248884	-4.595227709
O456	8.189797429	7.593727759	-8.817728423
O457	6.613063302	4.432247315	-9.144550106
O458	5.732048177	10.627018602	-8.694628135
O459	2.054255086	3.003057224	-8.241299893
O460	0.014539053	-10.007858293	-0.390864489
O461	1.321001222	-7.627151014	-3.759078110
O462	5.950542272	-5.822803220	-4.338000194
O463	9.073456325	-6.837862971	-1.577146553
O464	-3.056547062	-7.770171937	-6.313033192
O465	-4.177206579	-7.849100040	-0.386843373
O466	-2.155485815	-8.594966627	0.187182093
O467	1.122177962	-5.269388302	-7.849714872
O468	3.445997241	-7.619508054	8.383119592
O469	3.694327699	-7.601198063	11.891810321
O470	-4.463394336	-3.355116448	2.765515893
O471	-5.856354202	-3.722908400	4.456522606
O472	-3.987759267	-8.761941424	7.670880900
O473	-5.199632669	-8.547288849	5.777384260
O474	-0.526866244	-4.943324153	6.077430872
O475	-2.207792160	-4.454659196	7.496847529
O476	-2.101232193	-5.208399087	3.989400365
O477	0.836622338	-4.018063706	10.394875731
O478	1.294314089	-3.456538767	7.656021024
O479	1.418697758	0.905502632	-6.787604048
O480	-1.969627380	-8.371644982	10.021053103
O481	-1.461857553	-5.538139700	9.877174599
S482	6.779712362	-0.250422662	-0.773079817
S483	3.638097402	-2.184519708	-1.048720235
S484	-0.073638158	-5.173939720	1.481393857
S485	3.934607437	-0.014976955	1.914734527
S486	0.184679178	-1.618853108	0.218435415
S487	3.546923115	-5.842235024	0.620092407
S488	-0.047598549	-2.588691768	4.205480824
S489	5.723652512	-3.047381428	1.819494490
S490	2.301470948	-5.450495122	4.272134552
S491	3.624793209	-2.043407688	4.980043724

6 (from 4) 35 (S=2)	bm22b2n2x6h2x2h2nd235tf.car_3			
Fe(139) -2.378	C1	-9.114097042	0.002745981	-2.584228925
Fe(140) -1.051	C2	-9.881583792	-1.322776649	-2.621516887
Fe(141) -2.372	C3	-8.618994314	2.103589495	-3.755356283
Fe(142) 3.007	C4	-8.878321427	3.104374556	-2.631537383
Fe(143) 2.907	C5	-10.444605156	3.963018062	-0.963868617
Fe(144) 2.155	C6	-9.694118119	3.625455770	0.328668651
Fe(145) 1.352	C7	-8.589061936	1.886549928	1.651795611
	C8	-7.117598269	2.296859655	1.530515808
	C9	-5.182717307	2.598181689	0.077214480
	C10	-5.006218165	4.116255241	0.230808566
	C11	-5.905770380	6.338195149	-0.140984567
	C12	-6.121851071	6.867141826	1.283565389
	C13	-7.553497094	6.848658087	3.270868347
	C14	-6.839502075	6.274627653	4.500696188
	C15	-5.866464041	4.280141942	5.561161211
	C16	-4.374733991	3.938935028	5.497739409
	C17	-2.352712631	3.880575205	4.052795101
	C18	-1.576491703	5.189299132	3.814678969
	C19	-2.159974760	2.840415849	2.922161119
	C20	-0.687052983	2.733466820	2.519580392
	C21	-2.729875310	1.480250522	3.341481087
	C22	-1.235638003	7.160239901	2.381782108
	C23	-1.707784972	8.322457150	3.274941608
	C24	-1.356282702	7.477595056	0.872032577
	C25	-0.763140309	8.854773372	0.548535376
	C26	-0.673322685	6.390750428	0.032455530
	C27	-3.560449907	9.606341421	4.171886209
	C28	-3.013209889	9.629925418	5.584730288
	C29	-5.337367619	-2.991568084	11.998752339
	C30	-3.924768732	-3.131083271	11.433244159
	C31	-1.999129578	-1.799770956	10.551534867
	C32	-1.130355527	-1.126535943	11.633270753
	C33	-2.042327002	-0.970491877	9.255782132
	C34	-0.669020145	-0.749918527	8.616170112
	C35	-0.809977931	-0.219254543	7.187813051
	C36	0.969892721	1.104427105	6.028351482
	C37	0.966858644	-1.258394551	12.899709392
	C38	2.249097872	-0.682058312	12.338400303
	C39	-4.946214425	-1.505712851	-3.286090884
	C40	-4.231829672	-0.412544396	-4.099386725
	C41	-4.091884397	-1.829651939	-2.044357512
	C42	-4.797023577	-2.667326791	-0.956590898
	C43	-4.633650865	-4.180890322	-1.097833295
	C44	-2.816781841	-0.035762418	-6.069023144
	C45	-3.505039623	1.091864471	-6.843241098
	C46	-1.945062500	-0.882317693	-7.019331625
	C47	-5.575779809	2.299237794	-7.307275377
	C48	-5.526881872	3.514652123	-6.371621419
	C49	-5.005850000	4.361540513	-4.121855647
	C50	-3.604925074	4.771709552	-3.671465653
	C51	-1.178917904	4.431473262	-3.864011868
	C52	-0.683494541	5.733447554	-4.539890133
	C53	-0.186116502	3.341194063	-4.293582986
	C54	-0.310601101	2.001123822	-3.640634728
	C55	-0.834468394	1.552636178	-2.452224720
	C56	0.130603879	-0.178910763	-3.452772171

	C57	-0.707827451	7.003177955	-6.657173328
	C58	-0.842173404	8.381142169	-6.015507816
	C59	-1.531853676	6.943033689	-7.967254191
	C60	-0.887377240	7.551582848	-9.177935175
	C61	-0.317529291	8.796032420	-9.366715779
	C62	-0.209161372	7.606961803	-11.233456729
	C63	5.947769504	1.981996325	7.652546414
	C64	5.680968838	1.106341322	8.862885131
	C65	4.731367900	1.810346374	6.707222313
	C66	4.803493622	2.688685784	5.479538397
	C67	4.300350238	4.003779969	5.490634918
	C68	5.369282109	2.211288951	4.283718933
	C69	4.348081669	4.811095569	4.349965706
	C70	5.435073606	3.009467738	3.142084052
	C71	4.922068422	4.309778512	3.174941298
	C72	9.111000012	1.124361735	-0.035295661
	C73	9.260609394	1.539513965	-1.504829870
	C74	7.625299899	0.959003941	0.318568982
	C75	9.933956260	0.852317754	-3.763218251
	C76	8.648325544	0.851918392	-4.590506671
	C77	6.241067912	0.307664140	-4.646019047
	C78	5.439417920	1.594127708	-4.350336572
	C79	5.488585162	-0.974296069	-4.240821912
	C80	4.119206201	-1.223018164	-4.879160833
	C81	4.102912879	-1.596890676	-6.371612421
	C82	4.705753737	0.006523606	-8.201165214
	C83	4.889462312	3.392712989	-2.788344499
	C84	5.658495619	4.657951519	-3.218416441
	C85	4.547586433	3.521564390	-1.298400916
	C86	7.841516493	5.757640460	-3.286408459
	C87	8.368233974	6.287203330	-4.617584958
	C88	8.479871930	5.995470995	-7.061594236
	C89	7.694743641	7.078525347	-7.833077299
	C90	8.650104707	4.727192754	-7.931786850
	C91	7.311325370	4.052292501	-8.201312337
	C92	5.589352565	8.331741204	-8.017661365
	C93	6.086723525	9.771663752	-7.941147812
	C94	4.158681143	8.238224517	-7.424280735
	C95	3.535491542	6.874253246	-7.612830143
	C96	2.855150912	6.557297281	-8.799497629
	C97	3.653829345	5.877670432	-6.631184795
	C98	2.327998489	5.281497218	-9.013119796
	C99	3.152361155	4.587999386	-6.835145083
	C100	2.501938170	4.304370873	-8.034282304
	C101	2.042355395	-10.431290262	0.850689670
	C102	1.162454241	-9.586342860	-0.056786525
	C103	0.991523493	-7.432591411	-1.275815370
	C104	1.740810908	-7.159135110	-2.581126754
	C105	3.711588428	-6.096895988	-3.573442461
	C106	5.208637170	-5.934462105	-3.325624695
	C107	7.064758743	-5.661064248	-1.764233932
	C108	7.941910760	-6.896355126	-1.819102453
	C109	-2.116964208	-8.899148273	-4.392800285
	C110	-2.060200434	-7.897527259	-5.553887025
	C111	-2.570296735	-8.183506887	-3.099028795
	C112	-3.012416491	-9.198735204	-2.028274706
	C113	-3.214257416	-8.521817270	-0.690887579
	C114	-0.767747752	-6.175020842	-6.722291380
	C115	0.625180764	-6.247191680	-7.320337153

	C116	-1.221085503	-4.754322466	-6.300972343
	C117	-0.688638285	-4.343072534	-4.950350020
	C118	0.600420454	-3.804072036	-4.791465729
	C119	-1.458210000	-4.585557014	-3.798729851
	C120	1.113862663	-3.556230179	-3.511804294
	C121	-0.943324377	-4.344712579	-2.521044692
	C122	0.352547741	-3.841628168	-2.373273927
	C123	1.814045281	-8.516305189	6.808027302
	C124	2.277792433	-7.959880561	8.164635006
	C125	0.477149413	-7.908790725	6.353693308
	C126	0.042920277	-8.261740258	4.964207426
	C127	-0.314757849	-9.506746382	4.485198369
	C128	-0.692196947	-7.977687443	2.927844591
	C129	1.770964474	-7.414042039	10.482376048
	C130	2.987441601	-8.136018926	11.017240466
	C131	-4.821513212	-3.574663343	4.140337084
	C132	-3.548755181	-3.480323581	4.934328059
	C133	-2.788314108	-4.859563712	5.099765030
	C134	-3.811078840	-5.953828307	5.523849989
	C135	-3.146975831	-7.288649790	5.897150668
	C136	-4.087815285	-8.270912189	6.538504047
	C137	-1.798346034	-4.677129930	6.294978551
	C138	2.395641043	-2.985874591	1.987606303
	Fe139	2.102820184	-4.980882863	2.081659684
	Fe140	2.442198740	-1.248431601	0.427458314
	Fe141	3.854988460	-1.895421672	2.759493465
	Fe142	3.940628698	-3.659637187	0.867829224
	Fe143	5.097512771	-1.250403407	0.510211913
	Fe144	1.974967533	-3.148955928	3.935785907
	Fe145	0.472426276	-3.026279994	1.936726044
	H146	-9.397337599	-1.930168827	-0.735809126
	H147	-8.505031169	-2.646199905	-1.898810378
	H148	-9.888480222	0.517529021	-4.421954101
	H149	-9.841245467	-1.733809263	-3.642140627
	H150	-10.941790118	-1.075187120	-2.438029168
	H151	-7.533488263	1.929938461	-3.768127415
	H152	-10.804562148	2.428963663	-2.428813840
	H153	-8.907994860	2.569830992	-4.706978959
	H154	-11.523958667	3.895941839	-0.774848522
	H155	-9.552875550	1.655354579	-0.236231239
	H156	-10.203228186	5.005111070	-1.210470922
	H157	-4.497114510	2.126228780	0.793056384
	H158	-7.072370117	1.711107178	-0.453651643
	H159	-8.660513288	0.793262460	1.731708179
	H160	-4.914383312	6.676626613	-0.467482515
	H161	-6.776153005	4.478264621	-0.781233269
	H162	-8.967686527	2.330188023	2.580962202
	H163	-4.890172566	2.294102873	-0.938353097
	H164	-7.769205369	5.638316819	1.525560898
	H165	-6.661954744	6.789264189	-0.798636884
	H166	-3.793559107	1.554043260	3.611620388
	H167	-2.634972364	0.754717812	2.519018993
	H168	-2.180475731	1.071084674	4.203398586
	H169	-2.728464386	3.202342770	2.050645543
	H170	-8.629344225	6.660037905	3.395925606
	H171	-1.098932983	5.394311670	0.216058351
	H172	-3.710876577	7.969288303	2.821325191
	H173	-1.295952133	9.670389876	1.058746337
	H174	-0.567507678	2.009594336	1.701107203

	H175	-0.071195804	2.379668866	3.358166313
	H176	5.960511086	3.034830265	7.996071882
	H177	-0.792012286	6.614077741	-1.037322150
	H178	-0.816636970	9.038487370	-0.535074780
	H179	0.405556957	6.356150029	0.254595399
	H180	0.296192089	8.901503934	0.848083648
	H181	-2.431284935	7.494449128	0.615285630
	H182	-0.173223634	7.028761447	2.636940532
	H183	-2.626686974	5.550791559	2.062169814
	H184	-0.278105162	3.694290125	2.175273123
	H185	4.086148155	2.579372704	-0.959401745
	H186	3.805571934	4.331232637	-1.206622162
	H187	5.436202296	4.221807974	0.302881388
	H188	4.577821529	5.919347762	2.145020884
	H189	5.861557366	2.620155807	2.217126232
	H190	5.732479513	1.183300574	4.228941495
	H191	3.845700158	4.401492956	6.402375613
	H192	-4.336952401	4.506214422	3.538633860
	H193	-6.402550454	3.332410653	5.718929990
	H194	-6.019161930	4.905713560	6.449656757
	H195	-6.675883283	4.412441323	3.599383360
	H196	-7.387260142	7.933498025	3.291655852
	H197	3.928490889	5.819805505	4.369496876
	H198	-1.952934466	3.474824625	4.991759591
	H199	-3.360336307	10.583917663	3.701176338
	H200	-4.653532986	9.487359738	4.247735328
	H201	-2.696070361	8.639586936	5.987894479
	H202	1.210975268	-2.054589049	13.619133668
	H203	-4.401688503	-5.894511314	0.002024909
	H204	-5.772493803	-3.604323552	5.818119116
	H205	-3.968198726	-9.649419970	-2.332595103
	H206	-2.265400919	-10.001052192	-1.934712911
	H207	-3.407525723	-7.500242776	-3.309972338
	H208	1.076738218	-7.267827460	-7.308990806
	H209	-2.919106663	-9.592855517	-4.690059805
	H210	-3.168290693	-2.334830032	-2.370327531
	H211	-5.881713355	-2.458611050	-0.959374169
	H212	-0.159027802	-7.284429346	-4.991942199
	H213	-1.436314091	-6.503812409	-7.543778240
	H214	-0.123343813	-9.136458815	-3.869869883
	H215	-4.459346424	-4.420998514	0.965205580
	H216	-1.740528366	-7.560726250	-2.730234946
	H217	-1.032675555	-10.498519387	-3.704890066
	H218	-6.144712587	-2.694744617	-4.478225429
	H219	-3.790904880	-0.872793128	-1.593769176
	H220	-1.400923998	-9.103730014	-0.178339553
	H221	-4.431676003	-2.352471304	0.030195144
	H222	-1.124697464	4.609251331	-2.780765446
	H223	-1.365639660	2.074041963	-1.664483258
	H224	-5.471293910	5.252319610	-4.565378417
	H225	-4.860776860	2.338266528	-4.785240219
	H226	-2.001807948	5.400250650	-6.070954766
	H227	-0.161313573	9.640604634	-8.704978747
	H228	-1.776265734	8.508927242	-5.414480442
	H229	-2.529274516	7.379776656	-7.784192284
	H230	0.356489963	6.813080661	-6.865911223
	H231	0.015646348	7.358803164	-12.267390726
	H232	0.823762418	3.734874878	-4.092081444
	H233	-0.253663715	3.211091573	-5.386623127

	H234	0.581240396	9.581788082	-11.140542372
	H235	-1.694344740	5.880871026	-8.207199759
	H236	-2.740190667	3.311191469	-4.844350583
	H237	-5.589583011	4.103922150	-3.226456525
	H238	-5.164624600	2.625094460	-8.271092491
	H239	0.500915784	-1.183372041	-3.640405426
	H240	-6.626989799	2.024503825	-7.466277304
	H241	7.991058333	1.856796138	7.542291431
	H242	7.262933454	1.991798505	6.084648109
	H243	5.995218187	1.579206887	-2.354205110
	H244	3.943903344	3.409067046	-3.348806644
	H245	7.457810494	3.709291317	-2.937751138
	H246	0.776227076	0.911836060	-5.168405111
	H247	0.593744726	0.472358401	-7.206660142
	H248	1.503623201	1.777750603	-7.223191727
	H249	-1.385515215	-1.620053756	-6.428177451
	H250	-2.585524433	-1.418434996	-7.738911183
	H251	9.353066380	4.051893565	-7.422717135
	H252	9.086194127	5.038870875	-8.889314507
	H253	7.645826277	2.580036012	-6.729500875
	H254	6.045638414	2.651823484	-7.413481460
	H255	6.415662505	-0.290347401	-9.287375082
	H256	3.290318028	-2.311915577	-6.568556819
	H257	1.787826762	5.054935785	-9.935247356
	H258	4.135923976	6.093349207	-5.673497365
	H259	3.653825562	1.759434702	-8.518223893
	H260	5.346180264	-0.973317649	-3.148442842
	H261	7.600485443	0.007352566	-3.025155368
	H262	3.446860072	-0.368402275	-4.707367140
	H263	-5.347313047	0.391492407	-6.344419350
	H264	10.609678935	0.098333672	-4.188395604
	H265	-3.895271956	-1.856167034	-5.444862627
	H266	6.153133184	-1.824255296	-4.468223977
	H267	3.308730715	3.809357542	-6.085332234
	H268	-1.467514012	0.789751691	-7.908647234
	H269	10.396322170	1.837452803	-3.913453263
	H270	5.895209411	-1.664237468	-8.379761802
	H271	4.884582577	1.409207422	-9.682631437
	H272	-5.046501622	-3.545462933	-3.554306160
	H273	-5.882820563	-1.034359701	-2.932442694
	H274	2.724902147	7.318666705	-9.571778734
	H275	-2.141752115	0.496045256	-5.378396444
	H276	6.414896638	0.323606526	-5.731078895
	H277	5.037608106	-2.102561717	-6.644175968
	H278	3.683858505	-2.065687597	-4.324575216
	H279	3.004774720	0.080440180	-7.098752222
	H280	7.273543715	6.578495716	-2.823671664
	H281	7.369753368	4.817998000	-5.641293256
	H282	9.475886035	6.432037317	-6.891165561
	H283	8.720201852	5.564699770	-2.656582516
	H284	6.185850471	6.971102268	-6.464447652
	H285	3.100814963	-10.257024966	0.605366837
	H286	1.882159137	-10.038802970	1.876614726
	H287	2.595610071	-8.218645974	6.094422652
	H288	2.140150367	-12.370515445	1.472331376
	H289	0.713041718	-11.970850417	0.770132346
	H290	7.564299544	0.626923096	1.362245430
	H291	9.328013331	3.034829821	0.639589965
	H292	9.975518224	-0.328657252	-1.980333489

	H293	7.123602236	1.932207500	0.217476133
	H294	10.713658268	2.170801446	0.712802024
	H295	7.142794993	-5.228513726	-0.754632341
	H296	9.616476390	0.159210677	0.124169566
	H297	-2.524320190	-0.001795185	9.469634854
	H298	-1.607080322	-2.799718866	10.330457466
	H299	5.027728505	-5.975621471	-1.244908129
	H300	2.528978706	1.906505569	4.977525992
	H301	3.588375224	-6.881740462	-4.331001547
	H302	-1.547752472	-4.560934055	-1.637191133
	H303	3.375926668	-5.156790779	-4.043978793
	H304	-1.354904060	0.734513771	7.170378443
	H305	3.048393264	-6.016339278	-1.505851617
	H306	-1.393324900	-0.931422503	6.581835453
	H307	-2.692536210	-1.516372018	8.553393875
	H308	-0.111003925	-1.697304872	8.574158515
	H309	-0.071493996	-0.043076072	9.214518802
	H310	-2.320348180	-4.765290189	-6.272181128
	H311	-3.910642576	-1.138203026	11.291644308
	H312	-6.011343162	-3.413588919	11.237967496
	H313	0.774645917	-3.673398002	-1.380561710
	H314	-5.505834795	-1.362762185	13.248599850
	H315	-6.734743588	-1.491188978	12.173448432
	H316	0.948896222	-0.899679972	6.199127215
	H317	2.121610966	-3.155069672	-3.374461310
	H318	-2.473016726	-4.975839682	-3.900397190
	H319	-1.837639822	-7.378765786	9.966856041
	H320	-0.313000231	-8.232685143	7.054181449
	H321	-1.660984782	-5.166212564	8.919898826
	H322	-2.578701816	-8.532171139	9.259322203
	H323	7.402071854	-7.860809428	-1.976821636
	H324	0.627406762	-3.979712973	7.093885949
	H325	-3.744473860	-3.067289816	5.934693910
	H326	2.575085724	-8.100379089	-0.038743205
	H327	-4.377954759	-5.580013843	6.390464499
	H328	-1.141584674	-10.014303646	2.573600322
	H329	-1.013974902	-7.537755983	1.991605580
	H330	-5.388847609	-3.666811874	12.870610977
	H331	2.416678397	0.154259217	4.911903043
	H332	1.123987333	-3.830146901	9.443266131
	H333	2.084413524	-3.447110268	7.062559869
	H334	0.320480845	-2.586984399	11.331015209
	H335	-0.088098808	2.383390469	7.244213350
	H336	-5.685686748	-9.238374696	6.241252342
	H337	-2.722769009	-7.742507391	4.989327714
	H338	-2.330081548	-7.128635503	6.610348280
	H339	-4.515237285	-6.113700345	4.693959909
	H340	0.858885091	-6.505165446	-0.696611913
	H341	1.212500217	-3.607698222	-5.675486446
	H342	-0.899054257	-4.061506501	-7.092437802
	H343	-2.848228443	-2.820830000	4.405812781
	H344	0.056364802	-4.636283426	10.234168431
	H345	-2.154441974	-5.073337832	10.394567627
	H346	0.836549475	3.148837276	6.016297798
	H347	-0.289738026	-10.486387610	4.948219822
	H348	0.535125096	-6.815898461	6.430324468
	H349	0.011294357	-7.841783955	-1.526852557
	H350	7.466388790	-4.921628566	-2.473174668
	H351	3.829275066	2.051048987	7.290025695

	H352	4.665403757	0.752166403	6.405815007
	H353	2.697853869	-10.353412822	7.045390794
	H354	1.154826963	-10.305867076	7.586874190
	H355	0.942421874	-7.607537832	11.182845906
	H356	0.382246198	-8.139640255	9.037171762
	H357	3.129265024	-9.172849774	10.628975874
	H358	1.955435053	-6.327278326	10.476477330
	H359	5.974284050	0.037364638	8.727141294
	H360	6.822597788	9.974511664	-7.127992851
	H361	0.458013074	-0.447390037	13.448713063
	H362	2.168164130	-0.264264162	11.305621710
	H363	5.556694867	8.056656031	-9.083927375
	H364	4.194898056	8.497159119	-6.354153857
	H365	3.551478977	9.007382410	-7.923224146
	H366	-2.988297059	-2.599368054	1.804593935
	H367	1.564381647	2.961829982	-9.090881475
	H368	0.958774304	0.399234931	2.723118769
	H369	-2.352314810	-2.295538894	1.530573267
	H370	0.562774304	-0.224765069	2.860118769
	H371	-0.296327790	-0.398687704	0.978544686
	H372	-0.649856435	-0.410072494	-1.527371776
	Mo373	-0.045680126	-4.902591088	3.802351701
	N374	-9.452876199	-2.336213137	-1.673407768
	N375	-9.347317862	0.849422834	-3.626723924
	N376	-10.114913245	3.088719946	-2.077821018
	N377	-9.401775988	2.315985339	0.524880633
	N378	-6.549185868	2.149342707	0.305459930
	N379	-6.003292453	4.892704713	-0.255268480
	N380	-7.153223882	6.320145625	1.976544191
	N381	-6.426484534	4.977840965	4.415587516
	N382	-3.763116338	4.160977875	4.305367220
	N383	-1.926802573	5.913539994	2.712783459
	N384	-3.048614305	8.494080636	3.396027764
	N385	-5.725949122	-1.605524661	12.280301371
	N386	-3.346822615	-1.968002488	11.080148947
	N387	0.482190834	-0.044244576	6.518216026
	N388	0.416257418	2.293531684	6.368387569
	N389	1.970711505	1.071772173	5.141417068
	N390	0.055690644	-1.738497516	11.877235453
	N391	-5.187306495	-2.689212838	-4.120666234
	N392	-4.493619990	-4.873929963	0.050108013
	N393	-3.709470160	-0.854148908	-5.275885998
	N394	-4.851382715	1.135828842	-6.828870625
	N395	-5.080818258	3.290117810	-5.100384764
	N396	-2.550387315	4.089737887	-4.217484719
	N397	0.278006617	0.894838746	-4.241235437
	N398	-0.556469672	0.201761991	-2.371445460
	N399	-1.238003655	5.990705459	-5.751559312
	N400	-0.813916200	6.818935704	-10.351895273
	N401	0.110593830	8.809167609	-10.677089410
	N402	7.182449744	1.540919475	7.000608218
	N403	9.704874972	2.111518917	0.878302748
	N404	9.770907971	0.597338276	-2.346563123
	N405	7.556201484	0.305531438	-4.012094820
	N406	3.882684933	-0.446081795	-7.257750290
	N407	5.814827939	-0.669892262	-8.562337850
	N408	4.479204068	1.212002463	-8.771375468
	N409	5.575685171	2.143252191	-3.111797353
	N410	7.016711947	4.557103694	-3.308463309

	N411	7.925289766	5.668052974	-5.751081887	
	N412	6.967079142	3.076392783	-7.318242890	
	N413	6.489065838	7.426185316	-7.321636110	
	N414	1.727442581	-11.853733261	0.692763999	
	N415	1.692287621	-8.413663726	-0.456634357	
	N416	2.868538923	-6.430044228	-2.438507520	
	N417	5.666963867	-5.929010138	-2.048935460	
	N418	-0.878434747	-9.683228949	-4.301434931	
	N419	-0.907985920	-7.182311732	-5.684797434	
	N420	1.765350805	-9.991489044	6.825982431	
	N421	-0.196057220	-7.315617920	3.968389092	
	N422	-0.774530966	-9.304454431	3.201388799	
	N423	1.367081168	-7.913458657	9.177128259	
	N424	2.227137969	1.009985351	-1.410565015	
	N425	2.293434451	0.118475957	-0.714699658	
	O426	-8.367504224	0.321968607	-1.640398850	
	O427	-7.988449298	3.891706788	-2.268831036	
	O428	-9.405357624	4.515812713	1.145379251	
	O429	-6.488618118	2.750938516	2.498907617	
	O430	-3.994489744	4.594098490	0.769676636	
	O431	-5.406685564	7.775113370	1.741862752	
	O432	-6.708864606	6.974834499	5.513324550	
	O433	-3.811735201	3.469755896	6.498001558	
	O434	-0.674611617	5.544212926	4.589022104	
	O435	-0.899710956	9.065284934	3.857000194	
	O436	-2.976280998	10.642395639	6.267581188	
	O437	-3.416340524	-4.267497974	11.326471573	
	O438	-1.525860184	-0.103379675	12.219178595	
	O439	3.299370248	-0.651363549	12.960018497	
	O440	-4.141025189	0.771492583	-3.716589771	
	O441	-4.647051019	-4.743918371	-2.215644726	
	O442	-2.800353345	1.947542242	-7.423653240	
	O443	-0.997845739	-0.063052195	-7.721132570	
	O444	-5.889275231	4.619276707	-6.791759399	
	O445	-3.454362741	5.690659618	-2.862388354	
	O446	0.222595483	6.408983869	-4.044099521	
	O447	-0.047712987	9.293472868	-6.166736087	
	O448	5.164967231	1.503823267	9.897672202	
	O449	4.994010728	5.048000275	2.010353439	
	O450	8.945989898	2.674980806	-1.908053234	
	O451	8.671315728	1.318490540	-5.748150088	
	O452	4.727764043	2.089281359	-5.242527085	
	O453	5.043260820	5.715086513	-3.419190461	
	O454	5.725590173	3.827243787	-0.545775843	
	O455	9.145063242	7.250294597	-4.630357028	
	O456	8.164862286	7.586893477	-8.860078714	
	O457	6.554544646	4.440906323	-9.104211174	
	O458	5.691178035	10.652857855	-8.686140976	
	O459	2.049300113	2.988043619	-8.243234677	
	O460	0.007597394	-9.976277555	-0.365515505	
	O461	1.340534270	-7.599889478	-3.680550935	
	O462	5.952186282	-5.779514340	-4.309986541	
	O463	9.154696129	-6.857513586	-1.689577222	
	O464	-3.027868883	-7.740354975	-6.317913460	
	O465	-4.237136965	-7.913147868	-0.388430309	
	O466	-2.181491184	-8.570608581	0.172696960	
	O467	1.214727386	-5.300483037	-7.821219408	
	O468	3.464654592	-7.641670322	8.364989458	
	O469	3.726633629	-7.666814054	11.871235398	

	O470 -4.912535906 -3.605089829 2.919980564<br>O471 -5.984260485 -3.642865267 4.865340316<br>O472 -3.963526929 -8.747935648 7.664058370<br>O473 -5.131935192 -8.605948868 5.735444212<br>O474 -0.551844419 -4.847126519 6.007244411<br>O475 -2.239385425 -4.433563172 7.444117957<br>O476 -2.120266365 -5.195772909 3.938857904<br>O477 0.835370562 -4.034418815 10.369384840<br>O478 1.289537922 -3.471314565 7.629750132<br>O479 1.393970758 0.896157938 -6.790148754<br>O480 -1.954343179 -8.362042248 9.994531985<br>O481 -1.476191128 -5.530494623 9.831452639<br>S482 6.750681096 -0.256428066 -0.770978423<br>S483 3.773389531 -2.396437871 -1.016402197<br>S484 -0.043774183 -5.096749079 1.395966327<br>S485 3.764382731 0.069609635 1.730889003<br>S486 0.053532509 -1.536482958 0.311101323<br>S487 3.687638386 -5.894987722 0.792816175<br>S488 -0.182831187 -2.500822900 3.985300123<br>S489 5.753078845 -2.830291065 2.034396345<br>S490 2.254086444 -5.326084022 4.311022524<br>S491 3.417276470 -1.817886050 4.956571591

TS 3 $\rightarrow$ 5 35 (S=2)

TS 3 $\rightarrow$ 5 35 (S=2)	bm22b2n2x2h2n6h2x35dti_1_53238.099
Fe(139) -2.233<br>Fe(140) 0.128<br>Fe(141) -2.510<br>Fe(142) 2.973<br>Fe(143) 3.214<br>Fe(144) 2.520<br>Fe(145) -0.797	C1 -9.240958981 -0.008176296 -2.230131050<br>C2 -9.999501352 -1.336115575 -2.141157110<br>C3 -8.750952623 1.983742765 -3.577884305<br>C4 -8.929271287 3.067284147 -2.517395027<br>C5 -10.381184446 4.096938672 -0.847288144<br>C6 -9.559592050 3.856895636 0.423326079<br>C7 -8.404219996 2.226282849 1.833468718<br>C8 -6.935347454 2.585586450 1.584440353<br>C9 -5.080544311 2.670987610 0.006252719<br>C10 -4.844271248 4.188571972 0.005382180<br>C11 -5.711582924 6.401847726 -0.499353386<br>C12 -5.847761073 7.062320329 0.878704808<br>C13 -7.214465173 7.319146457 2.893772775<br>C14 -6.506359473 6.827345648 4.160144567<br>C15 -5.571298221 4.900512156 5.370026696<br>C16 -4.089833692 4.518242055 5.299254220<br>C17 -2.109835580 4.318488887 3.815926417<br>C18 -1.285114153 5.554372448 3.415555059<br>C19 -2.016968677 3.147139561 2.800699315<br>C20 -0.566429192 2.840750790 2.410495963<br>C21 -2.703708833 1.894115218 3.356591226<br>C22 -0.949743691 7.395907472 1.816421988<br>C23 -1.357314492 8.645438474 2.619818761<br>C24 -1.109869896 7.586287790 0.289201506<br>C25 -0.492944608 8.911067634 -0.174139948<br>C26 -0.477682253 6.411565405 -0.469509519<br>C27 -3.132511817 10.081526582 3.448693413<br>C28 -2.545703395 10.199724059 4.841940591<br>C29 -4.951245429 -1.735189453 12.439934427<br>C30 -3.569917152 -1.967641619 11.822412433<br>C31 -1.664709987 -0.785377342 10.716319749<br>C32 -0.751725162 -0.040347839 11.712015540<br>C33 -1.708802837 -0.072629497 9.352603315

	C34	-0.338388000	0.041550764	8.678631909
	C35	-0.472924595	0.507807261	7.228867384
	C36	1.317323684	1.432280011	5.740914167
	C37	1.369740169	-0.100504013	12.934991205
	C38	2.632687373	0.425781794	12.286528556
	C39	-5.097531612	-1.562853555	-2.965711460
	C40	-4.398107967	-0.588450253	-3.926486458
	C41	-4.132401624	-1.803023451	-1.779511584
	C42	-4.733057830	-2.500135662	-0.539814357
	C43	-4.646595563	-4.025959085	-0.554987539
	C44	-3.069689012	-0.464988674	-5.987337938
	C45	-3.778029818	0.566835555	-6.876477191
	C46	-2.223469479	-1.429149445	-6.843438129
	C47	-5.842776705	1.817959360	-7.290613675
	C48	-5.750534478	3.102579477	-6.454256584
	C49	-5.103283211	4.120327835	-4.311543875
	C50	-3.675654375	4.565494028	-3.991845871
	C51	-1.262701899	4.128484183	-4.158484755
	C52	-0.812403413	5.356094519	-4.987944260
	C53	-0.287885573	3.003942415	-4.542382957
	C54	-0.369656310	1.716918503	-3.785975842
	C55	-0.842470236	1.352643897	-2.549004917
	C56	0.132659455	-0.430323957	-3.443516222
	C57	-0.898217567	6.350444703	-7.252117838
	C58	-1.114751480	7.789840922	-6.793487027
	C59	-1.707664344	6.064251000	-8.541077756
	C60	-1.107452967	6.543908501	-9.831140571
	C61	-0.502032506	7.741824439	-10.159045357
	C62	-0.552600632	6.395499623	-11.918843130
	C63	6.095485809	2.668051841	7.201988710
	C64	5.875718040	1.899916829	8.492084955
	C65	4.844322640	2.429095132	6.316761221
	C66	4.903041099	3.173423975	5.003905663
	C67	4.371472188	4.469137106	4.864177771
	C68	5.509505777	2.585030680	3.879398162
	C69	4.433111298	5.152960046	3.643992627
	C70	5.593695967	3.260803838	2.662634197
	C71	5.048487027	4.541268635	2.544055689
	C72	9.157189307	0.933093963	-0.501689249
	C73	9.240670221	1.203200176	-2.009862677
	C74	7.688104799	0.787774338	-0.074140197
	C75	9.817972712	0.285339872	-4.215120495
	C76	8.496367383	0.228711457	-4.982324167
	C77	6.086487766	-0.302803144	-4.899104151
	C78	5.308081368	1.013224110	-4.678227927
	C79	5.336785903	-1.535718782	-4.360062197
	C80	3.941769972	-1.813879609	-4.929947280
	C81	3.872204765	-2.274369861	-6.396577876
	C82	4.336363320	-0.851207733	-8.400124283
	C83	4.799251596	2.909228367	-3.226240805
	C84	5.527346431	4.137577661	-3.806291750
	C85	4.551660327	3.160229905	-1.732459703
	C86	7.694633642	5.244191452	-4.040299581
	C87	8.226617621	5.629498732	-5.417495565
	C88	8.317009741	5.110830253	-7.821860866
	C89	7.546073538	6.147467019	-8.665757858
	C90	8.452836351	3.774027746	-8.586893500
	C91	7.098684424	3.112790150	-8.807165589
	C92	5.463656588	7.419734003	-8.934018559

	C93	6.035390658	8.831006769	-9.022659269
	C94	4.057232525	7.460180801	-8.280220517
	C95	3.381229966	6.109209258	-8.312006389
	C96	2.703539488	5.681980169	-9.464322450
	C97	3.459839710	5.229573647	-7.220473518
	C98	2.137087831	4.408297192	-9.540541221
	C99	2.924076869	3.938853636	-7.289010172
	C100	2.272694376	3.542606857	-8.454519842
	C101	1.890948927	-10.345665986	1.668246020
	C102	0.978948352	-9.587674269	0.717764504
	C103	0.760336651	-7.565352594	-0.702125418
	C104	1.463686627	-7.403610923	-2.055296866
	C105	3.409366107	-6.451852579	-3.201337965
	C106	4.912085694	-6.266675897	-3.008442890
	C107	6.820792094	-5.925221380	-1.517974846
	C108	7.643597354	-7.192070494	-1.371707334
	C109	-2.466111184	-9.220640432	-3.466514120
	C110	-2.456611284	-8.335469653	-4.718000445
	C111	-2.874062514	-8.371770454	-2.240330189
	C112	-3.261422135	-9.258907908	-1.042246888
	C113	-3.357614469	-8.436732472	0.224487182
	C114	-1.236013398	-6.699404435	-6.070858852
	C115	0.122175236	-6.826827776	-6.735369534
	C116	-1.669307671	-5.244368695	-5.761675787
	C117	-1.016861074	-4.710412570	-4.512656033
	C118	0.289798787	-4.190571099	-4.541829033
	C119	-1.662645382	-4.832617194	-3.270529008
	C120	0.944912838	-3.859086111	-3.350339300
	C121	-1.006394300	-4.498914941	-2.080664092
	C122	0.311404523	-4.034846117	-2.116539783
	C123	1.907853922	-7.922782251	7.489584067
	C124	2.417120042	-7.261459580	8.779406335
	C125	0.522660996	-7.405287009	7.071026144
	C126	0.043031432	-7.853302421	5.726566353
	C127	-0.334327816	-9.126821742	5.351329550
	C128	-0.749058154	-7.713265826	3.697089241
	C129	1.981087015	-6.501063853	11.048524866
	C130	3.210725131	-7.180055676	11.609752492
	C131	-4.584507637	-3.032167399	4.524769634
	C132	-3.384746871	-2.934574316	5.426688110
	C133	-2.672188477	-4.325294566	5.676134473
	C134	-3.721051956	-5.339093221	6.220776218
	C135	-3.090947472	-6.667033898	6.669275188
	C136	-4.029977630	-7.556539174	7.435109277
	C137	-1.636468913	-4.086350288	6.820641372
	C138	2.370215936	-2.838551921	2.183166114
	Fe139	2.030147516	-4.802184491	2.447924410
	Fe140	2.229944283	-0.910433931	0.918569750
	Fe141	4.034528456	-1.888842102	2.803717814
	Fe142	3.698477234	-3.619126950	0.918911622
	Fe143	5.149377992	-1.348805982	0.440153484
	Fe144	2.185757512	-2.929500009	4.191736421
	Fe145	0.289342472	-2.884743964	2.249848080
	H146	-9.402318576	-1.819973919	-0.252554990
	H147	-8.563869435	-2.599209314	-1.416444305
	H148	-10.069180859	0.373458622	-4.073550325
	H149	-10.012252954	-1.809725659	-3.134884303
	H150	-11.048850265	-1.087446771	-1.906750998
	H151	-7.671819402	1.779008275	-3.628757742

	H152	-10.856337125	2.457325397	-2.160994906
	H153	-9.071994511	2.388304112	-4.547222992
	H154	-11.446704338	4.060930075	-0.584888401
	H155	-9.469626510	1.839267734	0.027359380
	H156	-10.149070348	5.111479403	-1.196947697
	H157	-4.366081379	2.239049494	0.719313743
	H158	-7.024560362	1.802117423	-0.328355211
	H159	-8.487934698	1.148186615	2.024038794
	H160	-4.727544181	6.682123702	-0.894774919
	H161	-6.677241033	4.519868873	-0.897938200
	H162	-8.718607790	2.765915248	2.734715544
	H163	-4.860739349	2.264200790	-0.991395138
	H164	-7.551904913	5.957044702	1.287471533
	H165	-6.483050641	6.819179524	-1.161546784
	H166	-3.758184523	2.082338174	3.603820798
	H167	-2.671154693	1.079974939	2.615950598
	H168	-2.194789098	1.537265990	4.265873628
	H169	-2.553967735	3.460149601	1.890852812
	H170	-8.296631667	7.221893379	3.063757671
	H171	-0.929195013	5.449562655	-0.188405256
	H172	-3.387240936	8.319943021	2.272339347
	H173	-0.970078520	9.785076047	0.292432208
	H174	-0.547370977	2.045151086	1.649364144
	H175	0.013027004	2.482144769	3.273674638
	H176	6.120219004	3.745052156	7.459915222
	H177	-0.609361142	6.546309020	-1.552837478
	H178	-0.595149955	9.005644058	-1.264846092
	H179	0.604359397	6.360768438	-0.264933363
	H180	0.581649522	8.941386994	0.068486181
	H181	-2.192348886	7.607735779	0.065062955
	H182	0.115866204	7.256989221	2.051265913
	H183	-2.391965650	5.804975382	1.676031013
	H184	-0.049689281	3.715897620	1.989647035
	H185	4.175965080	2.230032049	-1.277364114
	H186	3.765678498	3.927593260	-1.659414223
	H187	5.494095663	4.106677598	-0.297906804
	H188	4.673381060	6.011019761	1.332695703
	H189	6.045613275	2.784179013	1.792623932
	H190	5.894105632	1.565657250	3.949328594
	H191	3.901002591	4.954106957	5.724715068
	H192	-4.078145999	5.008246633	3.321182347
	H193	-6.125694393	3.983406770	5.618806455
	H194	-5.687662376	5.602199812	6.206746843
	H195	-6.405803371	4.890889425	3.415248025
	H196	-6.978717745	8.386935424	2.803720099
	H197	4.007160603	6.155628942	3.547917824
	H198	-1.690739089	3.992638948	4.777056805
	H199	-2.897034363	11.000671066	2.888342731
	H200	-4.227125269	10.020052086	3.557460847
	H201	-2.257589089	9.234504059	5.320683097
	H202	1.636163559	-0.832706311	13.711215117
	H203	-4.380406865	-5.642158168	0.675899943
	H204	-5.680847398	-3.100589397	6.111079646
	H205	-4.243122450	-9.718509822	-1.230049898
	H206	-2.524923503	-10.065592631	-0.912442146
	H207	-3.727349592	-7.724049709	-2.495016712
	H208	0.600518528	-7.826121883	-6.606111465
	H209	-3.279149700	-9.937793963	-3.658141070
	H210	-3.267982825	-2.380442107	-2.146200559

	H211	-5.797315013	-2.229431577	-0.425133221
	H212	-0.545880365	-7.653932270	-4.276345291
	H213	-1.948068581	-7.099657001	-6.821009875
	H214	-0.439121348	-9.404370350	-3.045679274
	H215	-4.324259251	-4.089273830	1.501384049
	H216	-2.038889880	-7.703598076	-1.977814311
	H217	-1.339333742	-10.720719172	-2.630281851
	H218	-6.420626877	-2.806316425	-3.949890284
	H219	-3.750526136	-0.822430742	-1.460398675
	H220	-1.558220922	-9.076591958	0.712590378
	H221	-4.229740981	-2.120412998	0.361648309
	H222	-1.140020335	4.411493255	-3.102579530
	H223	-1.350855588	1.928443042	-1.784674527
	H224	-5.594961642	4.977486432	-4.790158805
	H225	-5.006517826	2.047955276	-4.818497667
	H226	-2.137892234	4.801197856	-6.445463468
	H227	-0.271397437	8.627755341	-9.579172223
	H228	-2.041075702	7.943339257	-6.187323058
	H229	-2.733652528	6.451760678	-8.412224806
	H230	0.176354499	6.201892673	-7.438047367
	H231	-0.396042327	6.049962714	-12.938380062
	H232	0.726564422	3.417232742	-4.413752956
	H233	-0.400377530	2.791149773	-5.617833749
	H234	0.319478924	8.334835141	-12.043279105
	H235	-1.795650188	4.970767952	-8.630642069
	H236	-2.868703617	2.836055704	-4.781456449
	H237	-5.622568336	3.945193027	-3.357839222
	H238	-5.459388669	2.053529869	-8.291459098
	H239	0.514356041	-1.440293663	-3.567357128
	H240	-6.901972516	1.545722095	-7.393777986
	H241	8.132041570	2.518304790	7.045514771
	H242	7.368773847	2.563974825	5.601537634
	H243	5.915763827	1.128461537	-2.699811725
	H244	3.822365169	2.875450258	-3.730309019
	H245	7.343799627	3.230291207	-3.515582110
	H246	0.670742175	0.512893724	-5.276196029
	H247	0.340058979	-0.166937503	-7.210668478
	H248	1.277594048	1.105265953	-7.390769468
	H249	-1.667205958	-2.109628688	-6.185039980
	H250	-2.881855230	-2.029787518	-7.492678449
	H251	9.136985230	3.125805146	-8.021046009
	H252	8.897187652	3.990454454	-9.566674169
	H253	7.416488691	1.737770650	-7.240081391
	H254	5.817530608	1.778704585	-7.943440752
	H255	5.898345136	-1.335250784	-9.633918037
	H256	3.067239934	-3.014505720	-6.519110368
	H257	1.590672674	4.091724158	-10.432332227
	H258	3.946818317	5.536478188	-6.289588647
	H259	3.346229125	0.928290653	-8.746828508
	H260	5.235444275	-1.443612093	-3.266794015
	H261	7.507318241	-0.472073085	-3.312503957
	H262	3.279386813	-0.947957252	-4.777656764
	H263	-5.590125390	0.023915928	-6.135928441
	H264	10.460493330	-0.526699062	-4.582002478
	H265	-4.179387520	-2.170652006	-5.131528644
	H266	5.978489187	-2.413126602	-4.543418175
	H267	3.049838121	3.242495704	-6.456991964
	H268	-1.750499009	0.106708615	-7.946667842
	H269	10.288098351	1.238463308	-4.492831344

	H270	5.387460200	-2.610250149	-8.585954738
	H271	4.419810991	0.419341357	-10.000720586
	H272	-5.290808610	-3.609517987	-3.016752851
	H273	-5.980271750	-1.018234215	-2.583207350
	H274	2.609802403	6.355858492	-10.318843461
	H275	-2.374176847	0.145041244	-5.385654635
	H276	6.211241413	-0.374880576	-5.987783394
	H277	4.808792792	-2.775308934	-6.672802139
	H278	3.527961348	-2.624198154	-4.314976799
	H279	2.761678387	-0.611222680	-7.147666783
	H280	7.106683178	6.102500195	-3.681134597
	H281	7.210279215	4.072824993	-6.289691436
	H282	9.322033688	5.541933239	-7.698474279
	H283	8.565233772	5.131639152	-3.379731192
	H284	6.030917281	6.148471786	-7.297918520
	H285	2.942447562	-10.149565700	1.412421817
	H286	1.715798796	-9.901681586	2.670672551
	H287	2.636023106	-7.643742534	6.715170757
	H288	2.082239921	-12.242468085	2.385860085
	H289	0.619975757	-11.934968033	1.712609694
	H290	7.670855812	0.550158976	0.997571527
	H291	9.386417549	2.902725636	-0.036995280
	H292	9.943813928	-0.701067710	-2.322125083
	H293	7.168215352	1.741987223	-0.240123660
	H294	10.776565666	2.049954880	0.079486804
	H295	6.936095022	-5.320571849	-0.605215818
	H296	9.684483183	-0.005533915	-0.271353373
	H297	-2.154561429	0.926921808	9.489030876
	H298	-1.304829276	-1.811829781	10.575748238
	H299	4.794848677	-6.154592985	-0.924566503
	H300	2.810258452	1.866945880	4.426633942
	H301	3.271432207	-7.301837035	-3.883414489
	H302	-1.515044111	-4.615743729	-1.121045992
	H303	3.058791570	-5.559162333	-3.745972356
	H304	-0.880402881	1.525769611	7.181209205
	H305	2.814433593	-6.198266552	-1.118075076
	H306	-1.181675498	-0.143133160	6.690035936
	H307	-2.389616938	-0.654801625	8.711970843
	H308	0.165121429	-0.936495567	8.681278135
	H309	0.308157352	0.745852793	9.225632143
	H310	-2.763189500	-5.243064426	-5.649902558
	H311	-3.544205907	-0.007976018	11.428609279
	H312	-5.673473636	-2.231819792	11.773269462
	H313	0.852347988	-3.798416426	-1.200831356
	H314	-5.027224085	0.038271407	13.485916715
	H315	-6.317526929	-0.196446577	12.503700807
	H316	1.150246924	-0.487552740	6.303218442
	H317	1.962674279	-3.464519989	-3.357300558
	H318	-2.690536575	-5.197336547	-3.229596091
	H319	-1.614503959	-6.406619329	10.684985084
	H320	-0.218501206	-7.734255631	7.821452973
	H321	-1.412454157	-4.308960911	9.445358898
	H322	-2.421347152	-7.587717122	10.104228916
	H323	7.091898435	-8.145658716	-1.562394397
	H324	0.833727982	-3.374994121	7.437800140
	H325	-3.662245158	-2.496867589	6.397009856
	H326	2.386729563	-8.103992980	0.529771032
	H327	-4.230223468	-4.873593915	7.079815286
	H328	-1.216336141	-9.769057433	3.505999614

	H329	-1.093358516	-7.338728814	2.740851436
	H330	-4.961609805	-2.297845782	13.388471189
	H331	2.624418676	0.168992123	4.757354286
	H332	1.410872935	-2.985376874	9.731685574
	H333	2.298730069	-2.872454318	7.312676596
	H334	0.665520761	-1.554847151	11.509440759
	H335	0.455949726	3.013275311	6.740301715
	H336	-5.663474157	-8.498659868	7.293967048
	H337	-2.733122782	-7.208674530	5.781724801
	H338	-2.233479357	-6.485490299	7.325851490
	H339	-4.467205405	-5.535631255	5.437145959
	H340	0.644978020	-6.590635556	-0.200810491
	H341	0.798576195	-4.073004617	-5.502212903
	H342	-1.413952902	-4.636844937	-6.643506775
	H343	-2.634324516	-2.294712889	4.943110485
	H344	0.360423524	-3.685322284	10.636573611
	H345	-1.843398140	-4.066360961	10.922739607
	H346	1.346137644	3.445405830	5.333465684
	H347	-0.291055381	-10.072326026	5.879685644
	H348	0.527015328	-6.308677269	7.088228978
	H349	-0.225805369	-7.998739969	-0.885216439
	H350	7.231407033	-5.341997408	-2.358676839
	H351	3.967284578	2.749466037	6.899141884
	H352	4.754772138	1.347900931	6.127737756
	H353	2.880781011	-9.702514121	7.805534063
	H354	1.363464091	-9.680750579	8.416772449
	H355	1.172057790	-6.624940386	11.785630364
	H356	0.544310445	-7.327025886	9.709381486
	H357	3.337876865	-8.249548920	11.314648358
	H358	2.171532882	-5.421850102	10.940570676
	H359	6.190894680	0.829268471	8.454221849
	H360	6.790781776	9.081777616	-8.240840890
	H361	0.877638501	0.757861251	13.426016739
	H362	2.517538343	0.755865065	11.225051446
	H363	5.368529612	7.035697726	-9.962258897
	H364	4.152718917	7.823946914	-7.245101530
	H365	3.459906192	8.202907505	-8.829610652
	H366	-1.973441820	-2.974136799	2.295736386
	H367	1.264734533	2.135323654	-9.347076466
	H368	1.636447140	0.161170996	2.115238394
	H369	-1.821617691	-2.229025307	2.308763532
	H370	1.261506891	-0.537661492	2.289417703
	H371	-0.683592638	-0.624183346	0.859349421
	H372	-0.577211706	-0.543146849	-1.489286571
	Mo373	0.031488607	-4.602994503	4.307166822
	N374	-9.502596586	-2.284880052	-1.159009423
	N375	-9.504025138	0.762637934	-3.322503794
	N376	-10.134381464	3.124910309	-1.900579281
	N377	-9.274997742	2.563152876	0.718394837
	N378	-6.443508539	2.293078152	0.352655910
	N379	-5.851370787	4.954376362	-0.480173713
	N380	-6.882591813	6.640420085	1.651142597
	N381	-6.137817245	5.514431072	4.182069202
	N382	-3.492576967	4.689514760	4.090391004
	N383	-1.661567088	6.198863242	2.273076704
	N384	-2.686845799	8.881388757	2.762014635
	N385	-5.305908099	-0.316946736	12.569077025
	N386	-3.004432923	-0.867947360	11.285801692
	N387	0.805939371	0.456710488	6.506142885

	N388	0.901709951	2.712909087	5.879493222
	N389	2.206245225	1.127802418	4.782744754
	N390	0.425520184	-0.657233069	11.985002424
	N391	-5.441760091	-2.809372956	-3.657394237
	N392	-4.423822564	-4.619323674	0.634044632
	N393	-3.942964234	-1.166882561	-5.068311340
	N394	-5.114353703	0.687225192	-6.743353865
	N395	-5.246582324	2.973744784	-5.191515706
	N396	-2.650925685	3.761187825	-4.418751455
	N397	0.226596104	0.580063926	-4.319365100
	N398	-0.528590493	0.019026585	-2.372590842
	N399	-1.366432899	5.427200571	-6.225608492
	N400	-1.133628046	5.712688336	-10.940452678
	N401	-0.152267151	7.625656313	-11.487783289
	N402	7.307794805	2.174396633	6.546445696
	N403	9.774287016	2.010166483	0.285097000
	N404	9.715394818	0.180675989	-2.774012452
	N405	7.427826009	-0.267896168	-4.321608690
	N406	3.607065339	-1.176351900	-7.335740557
	N407	5.352297611	-1.626276212	-8.829228436
	N408	4.118755861	0.324458927	-9.034350921
	N409	5.481125672	1.642825360	-3.484415461
	N410	6.882054801	4.039575956	-3.943010333
	N411	7.773390698	4.902968382	-6.482172482
	N412	6.744481069	2.195600343	-7.868437852
	N413	6.347702129	6.549924561	-8.177034343
	N414	1.626068056	-11.785602441	1.592990195
	N415	1.495960638	-8.470107015	0.172226410
	N416	2.600178795	-6.680776474	-2.017709408
	N417	5.410395625	-6.182480964	-1.748708701
	N418	-1.223933316	-9.997934058	-3.344924379
	N419	-1.324742615	-7.610909598	-4.942675849
	N420	1.924376293	-9.394368478	7.607889854
	N421	-0.216232302	-6.981024643	4.672114145
	N422	-0.834503876	-9.015035868	4.071349293
	N423	1.536717770	-7.113734782	9.808024138
	N424	1.899273610	1.352311078	-0.919579564
	N425	2.033618284	0.482507171	-0.210426265
	O426	-8.454543023	0.372281242	-1.341889811
	O427	-8.005542499	3.855491307	-2.254272789
	O428	-9.217750995	4.805859639	1.147725115
	O429	-6.239177538	3.123530498	2.459597481
	O430	-3.778202858	4.674413868	0.415011087
	O431	-5.067886249	7.965504988	1.227711252
	O432	-6.338214387	7.607778771	5.106590994
	O433	-3.523110662	4.066725416	6.305238914
	O434	-0.331967576	5.936232784	4.110652416
	O435	-0.504507342	9.405308089	3.107380034
	O436	-2.447568818	11.262671788	5.436411460
	O437	-3.072907757	-3.113752635	11.833115088
	O438	-1.106292483	1.043416162	12.208271427
	O439	3.702984568	0.512272051	12.866662909
	O440	-4.247698427	0.624928545	-3.669309220
	O441	-4.783755822	-4.683081239	-1.611147124
	O442	-3.098486613	1.288194146	-7.637856905
	O443	-1.270764994	-0.706197675	-7.638926438
	O444	-6.137405302	4.171279289	-6.939698731
	O445	-3.480516609	5.625335262	-3.391678052
	O446	0.068266041	6.118204520	-4.581934746

	O447 -0.380424162 8.718845702 -7.082642361<br>O448 5.373492504 2.381798243 9.497099501<br>O449 5.136320936 5.153403529 1.308986923<br>O450 8.902312708 2.292915155 -2.508114896<br>O451 8.472931942 0.610153683 -6.170518895<br>O452 4.576243430 1.457848453 -5.580230701<br>O453 4.890322544 5.166026031 -4.074040696<br>O454 5.754085041 3.599379081 -1.094142660<br>O455 9.010008742 6.580339021 -5.537665415<br>O456 8.025625968 6.574754734 -9.724753160<br>O457 6.343487249 3.457090467 -9.729939400<br>O458 5.678111371 9.645049957 -9.858423378<br>O459 1.777240348 2.227413200 -8.520751756<br>O460 -0.191800685 -9.995613335 0.503017185<br>O461 1.012815363 -7.931155454 -3.097290797<br>O462 5.622900557 -6.172146531 -4.023633694<br>O463 8.823367688 -7.189412029 -1.061370998<br>O464 -3.447009588 -8.264683920 -5.465218102<br>O465 -4.319135465 -7.722711121 0.495125570<br>O466 -2.297639623 -8.480095448 1.053414126<br>O467 0.656179240 -5.950107204 -7.399706412<br>O468 3.613438220 -6.943810670 8.917207055<br>O469 3.980243867 -6.638983333 12.391392481<br>O470 -4.566424805 -3.048589365 3.301278651<br>O471 -5.806168873 -3.122955528 5.142846562<br>O472 -3.863614206 -7.947558403 8.588714116<br>O473 -5.123568067 -7.920287891 6.713883669<br>O474 -0.405814394 -4.334341648 6.516808204<br>O475 -2.033749886 -3.726407792 7.955479022<br>O476 -2.063189604 -4.773142644 4.521022596<br>O477 1.150397331 -3.084717662 10.682551096<br>O478 1.512399167 -2.807970365 7.888962821<br>O479 1.174112271 0.266686728 -6.874942378<br>O480 -1.763187045 -7.379202899 10.799074045<br>O481 -1.195226178 -4.586564212 10.382201157<br>S482 6.781383382 -0.528748934 -1.012213686<br>S483 3.332800801 -2.139306132 -0.825399519<br>S484 -0.143544801 -5.026468100 1.948389659<br>S485 4.135419606 0.103217838 1.815549040<br>S486 0.042417376 -1.641524814 0.309333939<br>S487 3.414934471 -5.849818719 1.012621614<br>S488 0.072971938 -2.177138951 4.368477427<br>S489 5.716822295 -3.048442368 1.867836613<br>S490 2.342183094 -5.102437165 4.665606181<br>S491 3.818693906 -1.697174463 5.019408338

5 35 (S=2)

5 35 (S=2)	bm22b2n2x2h2n6h2x35dti.car_5
Fe(139) 0.531 -2.307<br>Fe(140) 0.276 0.078<br>Fe(141) 0.549 -2.662<br>Fe(142) 0.586 2.953<br>Fe(143) 0.613 3.219<br>Fe(144) 0.499 2.159<br>Fe(145) 0.436 -0.007	C1 -9.176845512 -0.015643831 -2.241464466<br>C2 -9.974208103 -1.321012396 -2.148563209<br>C3 -8.720593742 1.989752800 -3.587046614<br>C4 -8.906908482 3.072780059 -2.526775271<br>C5 -10.374788983 4.098576169 -0.865361232<br>C6 -9.572468299 3.853059062 0.416482816<br>C7 -8.420845482 2.212860824 1.822470230<br>C8 -6.953082056 2.586387767 1.586611291<br>C9 -5.084093841 2.684345500 0.025767669<br>C10 -4.862616876 4.204410851 0.029700615

	C11	-5.740938533	6.406719740	-0.504090602
	C12	-5.880129424	7.075494502	0.869776542
	C13	-7.228965141	7.310334646	2.898713061
	C14	-6.498869370	6.834371656	4.159873985
	C15	-5.551520685	4.918323249	5.378199380
	C16	-4.078291596	4.507119509	5.301261195
	C17	-2.101541509	4.288531449	3.810390292
	C18	-1.294321147	5.536845008	3.408501995
	C19	-2.002672892	3.121945401	2.793103391
	C20	-0.550745113	2.839929597	2.389936994
	C21	-2.668380002	1.860232459	3.354595507
	C22	-0.974598946	7.363574131	1.789149299
	C23	-1.386154328	8.614160870	2.589128566
	C24	-1.150318630	7.543357357	0.262285949
	C25	-0.555256859	8.872851156	-0.216413170
	C26	-0.510127999	6.371458673	-0.494516981
	C27	-3.160699930	10.049451342	3.415226451
	C28	-2.536105456	10.206036084	4.786922944
	C29	-4.929473838	-1.736782635	12.407343946
	C30	-3.549862275	-1.975665299	11.795231410
	C31	-1.626977914	-0.788908280	10.721500492
	C32	-0.703995360	-0.034961268	11.699838862
	C33	-1.700379121	-0.086102895	9.354040808
	C34	-0.343619445	0.028781137	8.654693059
	C35	-0.510072411	0.468065478	7.199768673
	C36	1.275737828	1.454459824	5.746408986
	C37	1.413183999	-0.101933117	12.931541968
	C38	2.686805558	0.390821014	12.277760915
	C39	-5.062250609	-1.601813316	-2.937697017
	C40	-4.358575108	-0.618137712	-3.886895341
	C41	-4.135148237	-1.865675598	-1.732392267
	C42	-4.795027294	-2.576646924	-0.532542437
	C43	-4.716406854	-4.102206350	-0.563161885
	C44	-3.042947956	-0.473935894	-5.953539277
	C45	-3.749651427	0.579235315	-6.815630805
	C46	-2.222529180	-1.429885793	-6.843715893
	C47	-5.819561827	1.795254654	-7.275794081
	C48	-5.730221079	3.082305531	-6.443689365
	C49	-5.084114093	4.106185510	-4.301050433
	C50	-3.654559730	4.529325809	-3.960166281
	C51	-1.245999741	4.100567933	-4.170841843
	C52	-0.779001673	5.328489501	-4.991088821
	C53	-0.282768201	2.965489597	-4.548795924
	C54	-0.367936820	1.690981518	-3.770631762
	C55	-0.855450701	1.350522864	-2.533010392
	C56	0.125574934	-0.452325492	-3.384019229
	C57	-0.872439918	6.362011216	-7.235064600
	C58	-1.052922978	7.796455009	-6.745657857
	C59	-1.698192230	6.107609473	-8.520199062
	C60	-1.102113132	6.601607085	-9.806421854
	C61	-0.476787839	7.794245958	-10.116875483
	C62	-0.560385565	6.479079460	-11.898534778
	C63	6.120511681	2.654660791	7.247088419
	C64	5.876206314	1.887729801	8.534041670
	C65	4.894709212	2.387528465	6.334373635
	C66	4.959620530	3.130450093	5.020126898
	C67	4.395133275	4.410310204	4.869394384
	C68	5.597943593	2.557869915	3.904198075
	C69	4.455585280	5.094177708	3.649416772

	C70	5.680720484	3.234010067	2.687304251
	C71	5.101874760	4.499025198	2.558963596
	C72	9.142953968	0.919690716	-0.503607811
	C73	9.237345494	1.183739696	-2.012221817
	C74	7.669506768	0.807888809	-0.078785336
	C75	9.812796377	0.257763003	-4.212835608
	C76	8.488944780	0.225612835	-4.979704202
	C77	6.075222952	-0.295252043	-4.897755757
	C78	5.308835499	1.028135682	-4.676288317
	C79	5.321792849	-1.526746171	-4.358534420
	C80	3.927326044	-1.807842595	-4.927825715
	C81	3.854052649	-2.270065572	-6.394088472
	C82	4.364993945	-0.835327612	-8.377612475
	C83	4.830987190	2.939478324	-3.232919144
	C84	5.570809592	4.160857325	-3.814856810
	C85	4.582660443	3.192365225	-1.739030099
	C86	7.743317744	5.253790412	-4.066078912
	C87	8.255375678	5.640859062	-5.450961760
	C88	8.320485691	5.115030762	-7.855455720
	C89	7.547493609	6.150138484	-8.702941612
	C90	8.442382083	3.772250905	-8.612312919
	C91	7.082153762	3.112627326	-8.801592970
	C92	5.455136546	7.422173507	-8.958941905
	C93	6.020988638	8.835123746	-9.047473453
	C94	4.057604499	7.447552235	-8.280926501
	C95	3.381282044	6.095204896	-8.309273208
	C96	2.647100068	5.693164005	-9.434833129
	C97	3.512294073	5.187358571	-7.244943102
	C98	2.079168587	4.418804659	-9.514484251
	C99	2.973932614	3.897827052	-7.316002309
	C100	2.265981389	3.527121406	-8.457034504
	C101	1.884958136	-10.337484408	1.682791620
	C102	0.981023737	-9.573700261	0.729275689
	C103	0.785150774	-7.542120518	-0.682747999
	C104	1.481179798	-7.404362667	-2.040885539
	C105	3.423957826	-6.473419971	-3.208089905
	C106	4.929514097	-6.303601924	-3.022012585
	C107	6.840899100	-5.964084014	-1.536905816
	C108	7.697252682	-7.214306833	-1.460676682
	C109	-2.465654651	-9.188649354	-3.445755479
	C110	-2.451447746	-8.296243632	-4.692722833
	C111	-2.861676232	-8.352700996	-2.209933933
	C112	-3.276664822	-9.256911977	-1.033967377
	C113	-3.380090367	-8.450714432	0.239590874
	C114	-1.193122453	-6.700721863	-6.061941391
	C115	0.166992199	-6.848926917	-6.717777503
	C116	-1.604308122	-5.238757325	-5.756213888
	C117	-0.982546927	-4.716723005	-4.486524955
	C118	0.326567626	-4.202268330	-4.478421510
	C119	-1.666670751	-4.831592686	-3.264149685
	C120	0.942866724	-3.854180078	-3.270820862
	C121	-1.048629301	-4.483444687	-2.057254561
	C122	0.267645926	-4.012030680	-2.055688608
	C123	1.921781017	-7.888117028	7.472582196
	C124	2.429743269	-7.231124458	8.767132459
	C125	0.561846281	-7.325745861	7.025798589
	C126	0.071495985	-7.789375310	5.687753379
	C127	-0.302855347	-9.069000197	5.328026031
	C128	-0.737532067	-7.675064572	3.661984416

	C129	1.993927153	-6.492735820	11.044628877
	C130	3.225301756	-7.168943893	11.604822950
	C131	-4.733639845	-3.067339316	4.638704957
	C132	-3.424266675	-2.930737952	5.369694117
	C133	-2.679029913	-4.309089191	5.613926106
	C134	-3.708385312	-5.335424605	6.167516202
	C135	-3.064122800	-6.652861443	6.626137533
	C136	-3.990639198	-7.538881750	7.411799649
	C137	-1.635214468	-4.058150742	6.748258072
	C138	2.434110626	-2.833971512	2.180110667
	Fe139	2.066151830	-4.816848149	2.420665916
	Fe140	2.053550241	-0.944093732	0.941244782
	Fe141	4.092835536	-1.833938744	2.823665473
	Fe142	3.756657370	-3.555325749	0.897581944
	Fe143	5.083249533	-1.285924845	0.413870246
	Fe144	2.114950110	-2.873838128	4.133307409
	Fe145	0.540193884	-2.871580644	2.200127506
	H146	-9.458060939	-1.796420437	-0.233500574
	H147	-8.600721505	-2.610756662	-1.359242304
	H148	-10.061021149	0.391360181	-4.056189279
	H149	-9.974035286	-1.811174707	-3.135050324
	H150	-11.024221524	-1.039233933	-1.954317211
	H151	-7.639597651	1.798252099	-3.642355784
	H152	-10.839367319	2.468037447	-2.190852555
	H153	-9.050102681	2.392509017	-4.554582513
	H154	-11.445383295	4.070205887	-0.621869873
	H155	-9.475355363	1.842867947	0.003909446
	H156	-10.128663703	5.112443750	-1.206356724
	H157	-4.372890114	2.258949744	0.745232946
	H158	-7.011350357	1.784687694	-0.319951490
	H159	-8.498690584	1.132378847	2.003994402
	H160	-4.761724151	6.692322807	-0.908183996
	H161	-6.680422802	4.515869941	-0.910677053
	H162	-8.745358085	2.742018278	2.726296231
	H163	-4.849769350	2.280135255	-0.969693402
	H164	-7.555147866	5.935435516	1.298486198
	H165	-6.519573285	6.806595696	-1.168348364
	H166	-3.719992688	2.039809172	3.621496389
	H167	-2.642773201	1.048879455	2.610667712
	H168	-2.140536460	1.502546136	4.252410655
	H169	-2.553352343	3.431391520	1.890164163
	H170	-8.306543698	7.190844878	3.081485173
	H171	-0.927029570	5.402652821	-0.184393783
	H172	-3.416522047	8.283704480	2.248428517
	H173	-1.039112015	9.743708287	0.248981915
	H174	-0.525193899	2.045882390	1.628059235
	H175	0.044007883	2.492962319	3.247666904
	H176	6.127335692	3.733416012	7.498187907
	H177	-0.679592180	6.480704085	-1.575345952
	H178	-0.670581567	8.959188701	-1.306660878
	H179	0.578087113	6.351733695	-0.319078280
	H180	0.520913949	8.920505470	0.014347189
	H181	-2.234585603	7.550781318	0.047082083
	H182	0.094868451	7.237838936	2.013317579
	H183	-2.394252556	5.752048852	1.660817116
	H184	-0.055216005	3.724343992	1.962555716
	H185	4.189741426	2.267391854	-1.285372548
	H186	3.810794283	3.974462857	-1.664872232
	H187	5.541501821	4.095568182	-0.286321679

	H188	4.695144147	5.955632911	1.341022698
	H189	6.155340684	2.766628387	1.823946815
	H190	6.007450065	1.548533769	3.978533162
	H191	3.896911291	4.886463245	5.718437325
	H192	-4.069471148	4.986532755	3.321586021
	H193	-6.123131816	4.017419867	5.646606239
	H194	-5.647828382	5.635749430	6.204212805
	H195	-6.399750377	4.893377909	3.429288863
	H196	-7.016026565	8.382243880	2.801865749
	H197	3.995918291	6.080691966	3.543828864
	H198	-1.676389938	3.967562198	4.770781105
	H199	-2.961086205	10.959187670	2.825404697
	H200	-4.250051941	9.973209013	3.560620782
	H201	-2.241860421	9.251869223	5.282677790
	H202	1.662593912	-0.822476402	13.725342175
	H203	-4.482226143	-5.734699740	0.656152364
	H204	-5.614922863	-2.838702436	6.338468293
	H205	-4.261897544	-9.699036579	-1.241974144
	H206	-2.550872697	-10.073677916	-0.907205224
	H207	-3.698150466	-7.679024592	-2.452723266
	H208	0.622055820	-7.861286293	-6.604459026
	H209	-3.289586607	-9.892456799	-3.642655401
	H210	-3.265104696	-2.445484505	-2.079840881
	H211	-5.863371743	-2.304942614	-0.470185384
	H212	-0.514859284	-7.679012081	-4.276715443
	H213	-1.904069240	-7.086230880	-6.820652543
	H214	-0.446939884	-9.413752207	-3.012877995
	H215	-4.410768015	-4.190404582	1.492943775
	H216	-2.012457308	-7.711577565	-1.927117521
	H217	-1.369643170	-10.727768292	-2.643140200
	H218	-6.388404699	-2.815736808	-3.954084775
	H219	-3.759473820	-0.890486270	-1.389629696
	H220	-1.567108357	-9.060560678	0.710882635
	H221	-4.336196843	-2.211788031	0.396590787
	H222	-1.130033223	4.378885864	-3.113279748
	H223	-1.374623152	1.941233089	-1.786734536
	H224	-5.561752279	4.965922032	-4.790059352
	H225	-4.993747948	2.032477354	-4.801037960
	H226	-2.132916652	4.823158661	-6.440706482
	H227	-0.230009908	8.668290820	-9.525161915
	H228	-1.951858631	7.947292313	-6.099773908
	H229	-2.718062441	6.505482978	-8.376002991
	H230	0.197222063	6.190979745	-7.432961448
	H231	-0.415749604	6.149346751	-12.924673227
	H232	0.736582076	3.369709420	-4.435318271
	H233	-0.407100248	2.738871007	-5.620682676
	H234	0.345365272	8.403760796	-11.995534872
	H235	-1.800677945	5.016564595	-8.627765267
	H236	-2.861856672	2.860690474	-4.880303529
	H237	-5.620478658	3.939241094	-3.355341553
	H238	-5.432673261	2.032113891	-8.274930080
	H239	0.502270514	-1.466384589	-3.485570101
	H240	-6.877659302	1.521803053	-7.382736983
	H241	8.162664529	2.519706175	7.142161195
	H242	7.433203775	2.550482769	5.680343382
	H243	5.935887161	1.150830789	-2.704341532
	H244	3.853814049	2.914930952	-3.736964812
	H245	7.383641661	3.244083474	-3.523710178
	H246	0.678625839	0.465490865	-5.227329327

	H247	0.333699587	-0.171446374	-7.210568407
	H248	1.274493596	1.098548224	-7.385930673
	H249	-1.660057607	-2.124984055	-6.206222616
	H250	-2.898855796	-2.013834932	-7.489800215
	H251	9.134741468	3.125464020	-8.054300409
	H252	8.871487823	3.980228237	-9.601012997
	H253	7.402851957	1.777324570	-7.202974683
	H254	5.795176329	1.825508679	-7.873999504
	H255	5.963909270	-1.298327693	-9.569056789
	H256	3.037197430	-2.997163997	-6.516922715
	H257	1.496166853	4.127730748	-10.391816960
	H258	4.034437883	5.476408398	-6.328366849
	H259	3.352938744	0.924952086	-8.765102794
	H260	5.222356721	-1.431525988	-3.265697250
	H261	7.498986178	-0.482056355	-3.315389833
	H262	3.262584771	-0.943587493	-4.776035976
	H263	-5.574291892	-0.012680381	-6.139178430
	H264	10.433779120	-0.571420484	-4.578671926
	H265	-4.148685381	-2.188672087	-5.110500296
	H266	5.965669670	-2.402480289	-4.540781647
	H267	3.140085964	3.186019355	-6.504076555
	H268	-1.745346981	0.126904092	-7.920076627
	H269	10.305760923	1.199698666	-4.491138892
	H270	5.455714218	-2.573777774	-8.521632318
	H271	4.494431944	0.442403026	-9.970822363
	H272	-5.280360528	-3.647191401	-3.021723456
	H273	-5.947147871	-1.054337558	-2.563289233
	H274	2.508913297	6.385140675	-10.269654240
	H275	-2.330369633	0.114721100	-5.351315282
	H276	6.196746804	-0.370094059	-5.986882073
	H277	4.781557277	-2.789260648	-6.665656738
	H278	3.515464503	-2.620043659	-4.313705484
	H279	2.752008184	-0.613211401	-7.166315798
	H280	7.169940440	6.116562264	-3.694889806
	H281	7.225551376	4.087479858	-6.311566563
	H282	9.329540680	5.538268594	-7.742503928
	H283	8.627218611	5.135670490	-3.424679068
	H284	6.039355857	6.146866764	-7.332659155
	H285	2.938325263	-10.167151431	1.415814192
	H286	1.727798983	-9.876939118	2.680182590
	H287	2.673254010	-7.643699963	6.708735499
	H288	2.032120441	-12.229224321	2.428125311
	H289	0.580114583	-11.899435921	1.744026977
	H290	7.644465593	0.558685857	0.990170903
	H291	9.409450955	2.886918235	-0.032056649
	H292	9.928128915	-0.726628600	-2.317434472
	H293	7.174588805	1.775828447	-0.241914066
	H294	10.784350373	2.009026300	0.075672334
	H295	6.951764177	-5.403961524	-0.595341182
	H296	9.649003605	-0.029706151	-0.268277243
	H297	-2.148414277	0.912117589	9.494323610
	H298	-1.267483491	-1.815690973	10.582363669
	H299	4.818015085	-6.223649260	-0.939366416
	H300	2.784939098	1.948253183	4.471096299
	H301	3.269476313	-7.326703108	-3.881973963
	H302	-1.584497783	-4.594588502	-1.111950066
	H303	3.081475592	-5.580534417	-3.757469724
	H304	-0.953268595	1.470559683	7.138204630
	H305	2.834866073	-6.183734435	-1.129919911

	H306	-1.201594894	-0.215891364	6.681217581
	H307	-2.392738420	-0.674643717	8.730682526
	H308	0.169861312	-0.944440245	8.660741450
	H309	0.305290165	0.747584469	9.180620867
	H310	-2.700769494	-5.211816541	-5.680350435
	H311	-3.486969992	-0.004913790	11.475460708
	H312	-5.655275775	-2.199957893	11.721897841
	H313	0.774579092	-3.758710756	-1.123147424
	H314	-4.997983739	-0.000634835	13.514259947
	H315	-6.268055522	-0.174920693	12.494415247
	H316	1.113878776	-0.487997137	6.226966529
	H317	1.961136320	-3.460838241	-3.247473312
	H318	-2.694404876	-5.200263067	-3.251710754
	H319	-1.594287629	-6.411730708	10.663021463
	H320	-0.195484676	-7.603779098	7.780067977
	H321	-1.415119854	-4.317881141	9.403582576
	H322	-2.368433521	-7.608589385	10.073479846
	H323	7.150164551	-8.178968182	-1.601690698
	H324	0.838277999	-3.367083468	7.402818899
	H325	-3.577256548	-2.439671132	6.341703858
	H326	2.405639391	-8.107493821	0.547882149
	H327	-4.226503136	-4.871549041	7.021400026
	H328	-1.199760862	-9.733765072	3.498547690
	H329	-1.091171042	-7.314520560	2.704753488
	H330	-4.959243860	-2.325768222	13.339657968
	H331	2.606746799	0.237160717	4.743664965
	H332	1.401115357	-2.996384654	9.717698777
	H333	2.304882625	-2.863771469	7.303685032
	H334	0.692959931	-1.566609789	11.522436347
	H335	0.407462022	2.986551868	6.809602597
	H336	-5.654173993	-8.433724502	7.318303815
	H337	-2.711358856	-7.208621708	5.744532591
	H338	-2.199152447	-6.460627708	7.270686010
	H339	-4.450721785	-5.543392910	5.382721424
	H340	0.697906064	-6.562649939	-0.186274282
	H341	0.865755187	-4.098659089	-5.423783675
	H342	-1.307353336	-4.634034006	-6.626720135
	H343	-2.739507620	-2.322264789	4.763873462
	H344	0.348227426	-3.698409904	10.618339626
	H345	-1.864057112	-4.069254178	10.874993073
	H346	1.306940051	3.481327708	5.433397746
	H347	-0.255581104	-10.007244370	5.869077460
	H348	0.610021633	-6.229953450	7.017891710
	H349	-0.211680957	-7.954628061	-0.853511616
	H350	7.233957577	-5.333515145	-2.350063273
	H351	3.996719149	2.688725355	6.894623352
	H352	4.831525322	1.303162410	6.147886107
	H353	2.828616807	-9.697188739	7.815109561
	H354	1.305363408	-9.617847324	8.408084755
	H355	1.186597260	-6.625126591	11.782374329
	H356	0.558719279	-7.318545896	9.706159525
	H357	3.356585341	-8.237319161	11.309059285
	H358	2.179158566	-5.411508711	10.944037090
	H359	6.191315374	0.816627516	8.499094740
	H360	6.800713962	9.081082637	-8.288489642
	H361	0.935435820	0.775251815	13.403533395
	H362	2.585990445	0.680566034	11.203406211
	H363	5.346488290	7.046083953	-9.988880408
	H364	4.170097434	7.799849254	-7.243736769

	H365	3.448607257	8.192260460	-8.813326336
	H366	-3.009728441	-2.511848779	2.305240291
	H367	1.244206220	2.123833993	-9.345447824
	H368	1.500952228	0.173662616	2.096678656
	H369	-2.381789937	-2.427215346	1.895616897
	H370	1.203277199	-0.536151654	2.367437300
	H371	-0.786976114	-0.544509131	1.079059261
	H372	-0.650856803	-0.530804625	-1.445486072
	Mo373	0.002792583	-4.546508956	4.211790366
	N374	-9.538407868	-2.268262922	-1.137977240
	N375	-9.456143749	0.761557062	-3.327015368
	N376	-10.115128488	3.126612261	-1.915232772
	N377	-9.286815492	2.557768497	0.705127796
	N378	-6.447064582	2.295722491	0.360359928
	N379	-5.869198295	4.958858317	-0.472889048
	N380	-6.898528625	6.635310692	1.653513779
	N381	-6.117991233	5.524615780	4.185239464
	N382	-3.487002146	4.652662551	4.086782475
	N383	-1.671806900	6.162718577	2.256566260
	N384	-2.716000254	8.841115619	2.742729961
	N385	-5.260652543	-0.316653805	12.578562260
	N386	-2.956117361	-0.867894881	11.313583726
	N387	0.757567704	0.444982866	6.459219825
	N388	0.848045243	2.725734425	5.933226379
	N389	2.178413575	1.193518509	4.788254328
	N390	0.463151338	-0.662738691	11.989389578
	N391	-5.412789906	-2.834877624	-3.651255957
	N392	-4.537234249	-4.711286159	0.624653522
	N393	-3.911677538	-1.185396605	-5.039090249
	N394	-5.092238245	0.666252994	-6.724167919
	N395	-5.227370675	2.957648732	-5.179433241
	N396	-2.636112537	3.747060049	-4.435039826
	N397	0.232635531	0.543131232	-4.275699199
	N398	-0.544466038	0.019462914	-2.327934602
	N399	-1.351483167	5.434796758	-6.217235050
	N400	-1.147514085	5.789133534	-10.928843199
	N401	-0.137754322	7.694924051	-11.449772962
	N402	7.353023811	2.166563842	6.625835145
	N403	9.781720536	1.985247706	0.283589265
	N404	9.706714218	0.155396819	-2.771664023
	N405	7.418001916	-0.270392553	-4.322555739
	N406	3.605456807	-1.173294965	-7.338582225
	N407	5.409949960	-1.591720469	-8.770583231
	N408	4.144656257	0.332670082	-9.022928246
	N409	5.498698900	1.665006142	-3.489534789
	N410	6.924634998	4.053982113	-3.953126445
	N411	7.785217684	4.918494635	-6.510770561
	N412	6.724449858	2.240277549	-7.820627799
	N413	6.348611496	6.548823955	-8.214061952
	N414	1.589607194	-11.772309902	1.627905268
	N415	1.507536741	-8.455557681	0.193799305
	N416	2.618550915	-6.682760639	-2.019053995
	N417	5.432978777	-6.236799616	-1.763435279
	N418	-1.238106912	-9.985506833	-3.333394265
	N419	-1.299179064	-7.611142429	-4.935143278
	N420	1.885716371	-9.358090026	7.604102332
	N421	-0.203819462	-6.929778557	4.625011206
	N422	-0.810344460	-8.973529879	4.049490283
	N423	1.550679166	-7.099095934	9.800062179

	N424	1.835600445	1.375418406	-0.863902579
	N425	1.923438869	0.476123444	-0.185840593
	O426	-8.370094906	0.348933548	-1.366600077
	O427	-7.985248772	3.861333373	-2.258220339
	O428	-9.242767820	4.798665622	1.150387937
	O429	-6.271801902	3.135799844	2.466136010
	O430	-3.810426409	4.702780867	0.461150111
	O431	-5.118574910	7.998263190	1.206940155
	O432	-6.326939360	7.621689707	5.099911651
	O433	-3.513603004	4.056312397	6.308752180
	O434	-0.359122420	5.946009228	4.112866651
	O435	-0.534763537	9.382664166	3.065858321
	O436	-2.419681657	11.284100697	5.350383115
	O437	-3.083160814	-3.134659137	11.765080955
	O438	-1.045784998	1.061854468	12.175533027
	O439	3.750487656	0.493747377	12.867375901
	O440	-4.212594120	0.593697258	-3.622179245
	O441	-4.833364001	-4.748511376	-1.629332570
	O442	-3.064057940	1.341778199	-7.530906335
	O443	-1.278287641	-0.703628225	-7.645456327
	O444	-6.113369400	4.149805519	-6.934272094
	O445	-3.450554619	5.550371668	-3.298557017
	O446	0.123669073	6.066079295	-4.587154082
	O447	-0.320221703	8.722997900	-7.047734060
	O448	5.355349839	2.366609221	9.531120692
	O449	5.185310983	5.112905615	1.324349486
	O450	8.914915805	2.275373800	-2.516674787
	O451	8.467183868	0.621071142	-6.163433564
	O452	4.574598297	1.472441256	-5.577158301
	O453	4.941817548	5.192785917	-4.089012689
	O454	5.791810231	3.609550103	-1.099212635
	O455	9.041185594	6.588862657	-5.576720735
	O456	8.023588031	6.576738547	-9.763669603
	O457	6.324071155	3.428222536	-9.732035728
	O458	5.635594755	9.654946382	-9.864572296
	O459	1.771331062	2.212075428	-8.527783102
	O460	-0.189564646	-9.974958370	0.503568195
	O461	1.025541616	-7.947951255	-3.072283423
	O462	5.638265803	-6.203508555	-4.037750524
	O463	8.900022521	-7.182649889	-1.255792162
	O464	-3.453479507	-8.188909168	-5.419763519
	O465	-4.354413964	-7.760098772	0.523528448
	O466	-2.310826672	-8.474074772	1.057426609
	O467	0.725191446	-5.973031362	-7.362115797
	O468	3.623766055	-6.905909403	8.906016795
	O469	3.992749463	-6.628012304	12.388745391
	O470	-4.880205155	-3.251403421	3.437698678
	O471	-5.866761860	-2.988882514	5.407260817
	O472	-3.786238388	-7.958284190	8.548886015
	O473	-5.120576038	-7.860102423	6.728194358
	O474	-0.405712303	-4.287423392	6.426951034
	O475	-2.022147918	-3.710488518	7.890006464
	O476	-2.074893334	-4.753326248	4.453585327
	O477	1.143938678	-3.105627245	10.668323663
	O478	1.514863833	-2.817643154	7.876391994
	O479	1.167552543	0.259623665	-6.872402796
	O480	-1.724538895	-7.386780016	10.777434038
	O481	-1.209396820	-4.591559941	10.342698305
	S482	6.732623904	-0.480762999	-1.025822302

	S483	3.237436922	-2.114918727	-0.830247929
	S484	-0.106688628	-4.949418521	1.859357825
	S485	4.029234632	0.129880955	1.786961980
	S486	-0.176774684	-1.614595547	0.487952678
	S487	3.474608385	-5.813013238	0.990974367
	S488	-0.019913516	-2.135440585	4.214594037
	S489	5.768912801	-2.957158222	1.841081400
	S490	2.305265512	-5.032764390	4.656460856
	S491	3.712727827	-1.636708774	5.028873408

TS 5 → 6 35 (S=2)

TS 5 → 6 35 (S=2)	bm22b2n2x2h2n6h2xd35th_1_53249.576			
Fe(139) -2.432	C1	-2.481788194	-2.988745294	-8.599536829
Fe(140) -0.061	C2	-2.157844401	-4.436343657	-8.983861133
Fe(141) -2.569	C3	-2.421761475	-0.632898148	-9.291974122
Fe(142) 2.899	C4	-3.854558280	-0.223765590	-8.956386094
Fe(143) 3.180	C5	-6.241912212	-0.629225738	-9.278262777
Fe(144) 2.195	C6	-6.651499065	-0.963884832	-7.840232124
Fe(145) 0.284	C7	-6.264431133	-2.382224379	-5.883292041
	C8	-5.649384766	-1.383564581	-4.899799748
	C9	-3.772558350	0.074079450	-4.359751548
	C10	-4.510547087	1.422118588	-4.367979632
	C11	-5.736570830	3.083293988	-5.645979568
	C12	-7.135221027	3.044991743	-5.012742921
	C13	-9.290895694	1.881012127	-4.914922755
	C14	-9.591852729	1.305761451	-3.525915540
	C15	-8.954962954	-0.330798167	-1.804251852
	C16	-8.033462151	-0.003390420	-0.626807297
	C17	-5.974215436	1.200014851	0.069517404
	C18	-6.019135821	2.718325265	0.322414660
	C19	-4.568203376	0.675658949	-0.321691936
	C20	-3.485954449	1.206184109	0.622908835
	C21	-4.557859264	-0.856869564	-0.368346338
	C22	-5.755880543	4.980772145	-0.616973464
	C23	-7.196307012	5.529731248	-0.623951526
	C24	-4.861034275	5.640799450	-1.693882577
	C25	-5.005551008	7.166705282	-1.682098429
	C26	-3.392958870	5.237605300	-1.500298116
	C27	-9.353184381	5.649215743	-1.728455532
	C28	-10.153156091	5.488319570	-0.451757077
	C29	-9.968487505	-8.231800684	3.689841525
	C30	-8.807176809	-7.611184153	4.467594247
	C31	-7.853915679	-5.417933105	5.194855414
	C32	-8.550776715	-4.789677767	6.418292559
	C33	-7.308183024	-4.348040265	4.233380880
	C34	-6.275558568	-3.418202869	4.876383604
	C35	-5.549781010	-2.588182390	3.817285887
	C36	-4.375074763	-0.420668194	4.249997739
	C37	-8.429336336	-4.447243689	8.843593727
	C38	-7.707124657	-3.227440269	9.375447716
	C39	0.734519481	-2.339227722	-5.558587633
	C40	1.165092973	-0.868649483	-5.686224040
	C41	0.418355831	-2.614311133	-4.073207298
	C42	-0.330709810	-3.929779464	-3.776858416
	C43	0.564352683	-5.139660789	-3.514004985
	C44	3.121256684	0.598073878	-5.900287628
	C45	2.842531984	1.488793557	-7.117447629
	C46	4.638179325	0.498558994	-5.641118436

	C47	1.556805241	1.795658676	-9.174808640
	C48	0.309085265	2.590129229	-8.766375443
	C49	-1.474664677	2.909617974	-7.103359552
	C50	-1.332352126	3.716019072	-5.813446839
	C51	0.130624435	4.458268782	-3.980434745
	C52	0.254309822	5.979691771	-4.248028471
	C53	1.445980024	4.052521470	-3.299347777
	C54	1.556292228	2.662522080	-2.756217714
	C55	0.657270147	1.691344615	-2.388157497
	C56	2.689575305	0.942065354	-1.894485065
	C57	1.215154743	7.650335973	-5.797099678
	C58	-0.005210376	8.566982082	-5.838193441
	C59	1.913050262	7.588421136	-7.177090229
	C60	2.804903306	8.745255879	-7.522008906
	C61	2.614542326	10.107832607	-7.388754137
	C62	4.589607259	9.689523806	-8.302800064
	C63	-3.846513643	1.974726185	8.855574993
	C64	-4.438920034	0.758233956	9.542915985
	C65	-3.598198069	1.579230439	7.378465456
	C66	-3.059111511	2.717435777	6.543570553
	C67	-3.919449762	3.594658220	5.856699147
	C68	-1.673223723	2.930223084	6.424449130
	C69	-3.418847309	4.640951761	5.071805803
	C70	-1.159632707	3.979275176	5.660962204
	C71	-2.033825697	4.829338553	4.976806266
	C72	3.901624056	4.766719230	6.839601304
	C73	4.844235123	5.611370646	5.972257743
	C74	3.037726162	3.864950900	5.946114010
	C75	7.162695557	5.954140533	5.221450972
	C76	7.157518067	5.630319179	3.726387822
	C77	6.314136014	4.161104641	1.934787117
	C78	5.100210478	4.858485943	1.284410330
	C79	6.255815952	2.627346937	1.809527178
	C80	6.187723804	2.045046006	0.393188073
	C81	7.456648736	2.179220168	-0.471152806
	C82	8.308563133	4.326452946	-1.434180530
	C83	2.810140514	5.679630188	1.499868929
	C84	2.863785435	7.216349263	1.612106632
	C85	1.494687762	5.194622312	2.122595677
	C86	3.397949150	9.110324481	3.060125972
	C87	4.382066569	10.175911302	2.581990497
	C88	6.402821076	10.691830261	1.268200583
	C89	6.090270926	11.539447363	0.017688330
	C90	7.735660983	9.930358737	1.079523082
	C91	7.627859193	8.888321655	-0.027605969
	C92	4.633869402	11.833398574	-1.940114141
	C93	4.165893253	13.268784657	-1.727709455
	C94	3.531329164	11.016549037	-2.665639819
	C95	3.997521938	9.630126545	-3.045624936
	C96	4.721986408	9.422505939	-4.230680956
	C97	3.768925358	8.526429169	-2.206684974
	C98	5.228789609	8.162574371	-4.557788618
	C99	4.289463704	7.262039572	-2.504528134
	C100	5.022486753	7.099251835	-3.678321907
	C101	5.347062709	-8.388728259	3.801083049
	C102	5.198468880	-7.764593532	2.423685711
	C103	4.978931881	-5.625014209	1.187185913
	C104	6.168390811	-4.701824867	0.908256005
	C105	7.337622063	-2.674489325	1.634378935

	C106	7.803844356	-1.986700507	2.915343771
	C107	7.424136567	-1.447177745	5.271375639
	C108	8.383811928	-2.207637700	6.167970172
	C109	6.427376771	-7.276053529	-2.857589730
	C110	6.818791433	-6.053466323	-3.695153852
	C111	4.916160296	-7.229353167	-2.536939069
	C112	4.403106467	-8.597013215	-2.049417607
	C113	3.009730890	-8.466096760	-1.479725079
	C114	7.464236587	-3.694812781	-3.717556606
	C115	8.611138055	-2.986359288	-3.023899117
	C116	6.252393343	-2.787489087	-4.047558100
	C117	5.339856207	-2.588461111	-2.864947619
	C118	5.616266787	-1.624063875	-1.880396438
	C119	4.238751641	-3.443560910	-2.676345263
	C120	4.835479429	-1.553252283	-0.721481746
	C121	3.460448034	-3.372728656	-1.517199473
	C122	3.768151038	-2.436631299	-0.525384775
	C123	-0.061525511	-8.546982176	6.965105459
	C124	-1.118527775	-8.291483442	8.050020249
	C125	-0.655420189	-8.460970535	5.550706505
	C126	0.323120419	-8.545120494	4.420967453
	C127	1.096302295	-9.630491465	4.060723438
	C128	1.306500831	-8.035072217	2.539455938
	C129	-3.321968107	-8.745174682	8.965504281
	C130	-2.780455025	-9.012634747	10.352964312
	C131	-3.681626072	-6.300673578	-0.611403971
	C132	-3.674459459	-5.901068100	0.840377046
	C133	-2.752947872	-6.813230002	1.751617759
	C134	-3.025637863	-8.303636155	1.399846156
	C135	-2.337630912	-9.290808232	2.356251673
	C136	-2.811901465	-10.707194593	2.194831063
	C137	-3.238739467	-6.597288255	3.218833550
	C138	1.121729180	-2.147405351	3.591214158
	Fe139	1.873252796	-4.049643118	3.740714883
	Fe140	0.978425030	-0.323803823	2.375460129
	Fe141	0.825062676	-0.858071091	5.121348046
	Fe142	3.026437838	-1.757482006	4.046405970
	Fe143	2.768328033	0.860955061	4.471357816
	Fe144	-0.407081855	-3.039115916	4.481221926
	Fe145	0.318488615	-2.992412570	2.088080701
	H146	-3.005710160	-5.319002842	-7.350259454
	H147	-1.380685227	-5.192492457	-7.248573959
	H148	-1.770062703	-2.330424671	-10.416936271
	H149	-1.188557381	-4.461088694	-9.506673501
	H150	-2.912499886	-4.744096271	-9.728256502
	H151	-1.804972137	-0.315216046	-8.438923288
	H152	-4.602363901	-1.659296253	-10.218835522
	H153	-2.099748913	-0.071516521	-10.179290362
	H154	-6.865885004	-1.212046809	-9.968569271
	H155	-5.245063592	-2.460541175	-7.754433707
	H156	-6.460813103	0.435526282	-9.432181576
	H157	-3.740143249	-0.258608416	-3.314032390
	H158	-3.895673771	-1.320044555	-5.997257826
	H159	-5.843162119	-3.380613667	-5.701264976
	H160	-5.168905280	3.876476259	-5.144399643
	H161	-4.828066699	1.288580531	-6.408577505
	H162	-7.340355236	-2.414079733	-5.675223457
	H163	-2.740128010	0.215859372	-4.712181493
	H164	-7.539865929	1.248473774	-5.956715204

	H165	-5.841930557	3.347360954	-6.707674421
	H166	-5.314583552	-1.246790968	-1.064179928
	H167	-3.573699725	-1.224189843	-0.698972306
	H168	-4.749757991	-1.285228584	0.628738805
	H169	-4.353940527	1.052556610	-1.334881995
	H170	-9.816278452	1.252380760	-5.648900275
	H171	-3.247513958	4.150011256	-1.566661990
	H172	-7.663829445	4.539741572	-2.399140653
	H173	-6.037272896	7.489121693	-1.882498078
	H174	-2.497658310	0.858419694	0.288034356
	H175	-3.633024226	0.835975671	1.647297125
	H176	-4.613498421	2.773006277	8.869623158
	H177	-2.768947814	5.710009452	-2.273190801
	H178	-4.356285390	7.609421716	-2.451889938
	H179	-3.025528179	5.574746422	-0.517170065
	H180	-4.703972113	7.582654745	-0.707879289
	H181	-5.197065624	5.270611043	-2.679300131
	H182	-5.369245441	5.238151295	0.379930004
	H183	-5.421113572	3.111785965	-1.626069890
	H184	-3.457967841	2.305296770	0.653891650
	H185	1.499448334	4.092766112	2.146655020
	H186	0.679556740	5.526887096	1.460010453
	H187	0.384762659	5.732215251	3.648348350
	H188	-2.179478149	6.344357491	3.772778869
	H189	-0.082677918	4.120554684	5.571008077
	H190	-0.978456987	2.243392156	6.912133833
	H191	-5.002743990	3.458619068	5.924308148
	H192	-6.876627697	1.117715991	-1.874089782
	H193	-8.876380257	-1.413638381	-1.982443581
	H194	-9.983115915	-0.115966284	-1.484891902
	H195	-7.943717428	0.049128747	-3.635506760
	H196	-9.738429777	2.882559804	-4.939226468
	H197	-4.103349630	5.298942179	4.530234930
	H198	-6.283132098	0.740300885	1.018021281
	H199	-9.349642716	6.714083634	-2.014799442
	H200	-9.881793496	5.086769828	-2.514485356
	H201	-9.853580984	4.624217672	0.186318707
	H202	-8.424450983	-5.236981954	9.609921672
	H203	0.692440816	-6.840999954	-2.380052053
	H204	-5.407269979	-7.097683350	-0.296926977
	H205	4.365082843	-9.292166880	-2.900726744
	H206	5.083943104	-9.013334038	-1.292716664
	H207	4.344237701	-6.934475344	-3.429657500
	H208	9.304948188	-3.664508888	-2.474136207
	H209	6.593623922	-8.120906314	-3.543052263
	H210	1.359609665	-2.582216170	-3.501634089
	H211	-0.993837970	-4.189276957	-4.620990973
	H212	7.039350278	-4.892469875	-1.987101062
	H213	7.896556294	-4.026087127	-4.682738745
	H214	7.129331692	-6.787823481	-0.966585351
	H215	-0.728506066	-5.857816239	-2.048191169
	H216	4.739077860	-6.450654355	-1.779489805
	H217	7.202742488	-8.394851935	-1.320577002
	H218	1.565660295	-3.523278619	-7.035198989
	H219	-0.204080699	-1.780826823	-3.716594696
	H220	3.808770647	-8.402423590	0.321703425
	H221	-0.990675959	-3.776278903	-2.912065228
	H222	-0.722004406	4.334703927	-3.297179114
	H223	-0.425180795	1.665988990	-2.435527597

	H224	-1.802301223	3.608954600	-7.884179007
	H225	0.066655245	1.433526909	-7.049061744
	H226	0.929149490	5.553970772	-6.134739745
	H227	1.801364184	10.698614490	-6.981522537
	H228	-0.927967370	8.078507301	-6.236929247
	H229	1.151809721	7.427138673	-7.960376841
	H230	1.912720611	8.030517793	-5.035295520
	H231	5.565631441	9.878572291	-8.743642128
	H232	1.601646170	4.761653682	-2.468897696
	H233	2.275427294	4.222059461	-4.006963418
	H234	3.949369652	11.686441277	-7.936294361
	H235	2.555122126	6.693868020	-7.179208243
	H236	0.624058159	3.104876934	-5.585268107
	H237	-2.292011379	2.187223775	-6.959762935
	H238	2.305187154	2.525417497	-9.509025235
	H239	3.491382263	0.299646015	-1.541936632
	H240	1.295779301	1.162950245	-10.033516431
	H241	-2.787085004	2.781711040	10.414597259
	H242	-2.093513997	3.028166212	8.955579194
	H243	3.903571142	4.473607911	2.931113744
	H244	2.774666059	5.462346895	0.422197434
	H245	3.963593937	7.094005720	3.341571501
	H246	3.724472657	2.651541432	-2.636502579
	H247	5.330551748	2.764504527	-4.020877622
	H248	5.166845882	4.269747050	-3.531720916
	H249	4.808708414	-0.062849922	-4.712835814
	H250	5.127557341	-0.037309330	-6.471195671
	H251	8.017968653	9.474377955	2.039699933
	H252	8.499628798	10.664215890	0.794902471
	H253	7.416493731	7.311161546	1.355055450
	H254	7.136335081	6.925171868	-0.321693450
	H255	10.119516754	5.059994557	-0.820430600
	H256	7.586147533	1.281316841	-1.093605546
	H257	5.775631441	8.012570342	-5.491919230
	H258	3.176536704	8.634125879	-1.294294412
	H259	7.149775952	5.508725284	-2.667197675
	H260	5.380823823	2.255907910	2.366126931
	H261	5.916818303	4.006458452	4.030264815
	H262	5.323027998	2.450513238	-0.154743920
	H263	1.867173501	-0.020925252	-8.067880231
	H264	8.158210890	5.710420633	5.617299598
	H265	2.991756630	-1.599354649	-6.050380581
	H266	7.147942264	2.225927141	2.317561899
	H267	4.156242178	6.431199242	-1.808540055
	H268	4.749792587	2.385095823	-6.127829810
	H269	7.028608143	7.042144268	5.298527763
	H270	9.856900940	3.392796302	-0.452406521
	H271	8.780076699	6.035800556	-2.455642675
	H272	1.840489749	-4.073503860	-5.482333685
	H273	-0.216175538	-2.413171714	-6.119508444
	H274	4.894750126	10.256589655	-4.915481434
	H275	2.672736825	1.158106818	-5.062491331
	H276	7.187119575	4.552376981	1.395889914
	H277	8.339968173	2.241964569	0.177365124
	H278	5.987938314	0.972405817	0.518587380
	H279	6.608679638	3.384234949	-2.015226416
	H280	2.400336333	9.439736026	2.733004726
	H281	5.398877020	8.794806768	1.454989015
	H282	6.536770370	11.420595325	2.081435226

	H283	3.416962463	9.134604683	4.158049322
	H284	4.423604482	10.417874473	-0.339008435
	H285	5.879903787	-7.696956451	4.469585385
	H286	4.315720341	-8.489378079	4.198809298
	H287	0.679826526	-7.744704302	7.088389135
	H288	5.971167849	-10.160804302	4.592780984
	H289	5.642324811	-10.231620773	2.989341314
	H290	2.410741489	3.240534757	6.595245669
	H291	2.577794467	6.300180714	7.066722435
	H292	6.438462929	4.466961964	6.574740258
	H293	2.393645855	4.491951170	5.312361801
	H294	3.588014987	6.129763704	8.340768602
	H295	6.501890514	-1.248165870	5.838525865
	H296	4.499027304	4.129676090	7.510465622
	H297	-8.154138809	-3.764753016	3.832384737
	H298	-7.028465163	-6.060203768	5.525186569
	H299	6.205005568	-2.692252009	4.056889218
	H300	-3.151016553	1.162468580	4.650701906
	H301	8.211158447	-3.176071501	1.198616175
	H302	2.620230503	-4.055966149	-1.374941596
	H303	7.065246674	-1.865516889	0.934699157
	H304	-6.253123351	-1.956307436	3.259537613
	H305	5.465191871	-3.490095224	2.389542746
	H306	-5.073937750	-3.256405402	3.081409136
	H307	-6.845510322	-4.886258130	3.390721109
	H308	-5.522535097	-4.008759006	5.419756921
	H309	-6.755209587	-2.747807673	5.607721616
	H310	5.700796232	-3.260438229	-4.872216219
	H311	-9.636750562	-5.838868256	4.059383289
	H312	-9.528045695	-8.641291766	2.767679663
	H313	3.191512225	-2.382140393	0.399285179
	H314	-11.743785471	-7.255291370	4.062261123
	H315	-11.481396538	-7.549663015	2.472308702
	H316	-3.644571310	-2.239776606	4.693635634
	H317	5.044743018	-0.820249305	0.061092367
	H318	3.988983878	-4.180117411	-3.442760459
	H319	-4.694801475	-10.018051697	5.883226346
	H320	-1.382854304	-9.282964317	5.426232512
	H321	-4.877527416	-7.739251725	5.011715761
	H322	-3.966783427	-11.100718043	5.060865228
	H323	8.726485027	-3.193929082	5.770553699
	H324	-3.005719320	-5.232708767	5.480924197
	H325	-4.695200441	-5.915569477	1.248903419
	H326	5.143461310	-5.896332326	3.274816859
	H327	-4.113445823	-8.470574902	1.433520748
	H328	2.321282830	-9.875906206	2.319791272
	H329	1.614534820	-7.532763850	1.630932136
	H330	-10.315769245	-9.093540520	4.284839920
	H331	-2.401847219	-0.405309114	4.847512670
	H332	-4.578054944	-5.592456530	7.273277188
	H333	-2.549237884	-4.178435255	6.523667801
	H334	-6.952975436	-5.403413548	7.601032211
	H335	-6.373975054	-0.017037312	3.977303719
	H336	-2.947831290	-12.110440176	0.933205192
	H337	-1.251626113	-9.262337713	2.182998881
	H338	-2.521907063	-9.016151343	3.401190711
	H339	-2.679293226	-8.490088831	0.372813672
	H340	4.050547040	-5.044062699	1.302421325
	H341	6.466035966	-0.949196981	-2.018116644

	H342	6.659606842	-1.830658187	-4.408862752
	H343	-3.268164880	-4.884700469	0.929767079
	H344	-5.287892418	-6.957625136	7.060918792
	H345	-6.253125413	-8.275684652	5.512877375
	H346	-5.331133293	1.334633217	3.775173474
	H347	1.249997456	-10.588765235	4.542345740
	H348	-1.219627596	-7.525450281	5.454563735
	H349	4.873193202	-6.310202702	0.343508604
	H350	7.880995625	-0.475482357	5.026208095
	H351	-4.557978389	1.231988925	6.965790433
	H352	-2.894834796	0.731274627	7.357123512
	H353	1.078130140	-9.811543447	8.110980731
	H354	-0.060643710	-10.589179147	7.231230713
	H355	-4.126676963	-9.475486509	8.785067218
	H356	-2.563725691	-9.496331855	7.123241024
	H357	-1.914453060	-9.715865150	10.395024636
	H358	-3.771564211	-7.739943455	8.930442578
	H359	-3.680400245	0.011592205	9.884136785
	H360	3.876651630	13.524055386	-0.681183310
	H361	-9.477950617	-4.153203412	8.663817010
	H362	-7.179074267	-2.613847262	8.605822087
	H363	5.529918918	11.874615511	-2.580549803
	H364	2.643816028	10.959933219	-2.015535899
	H365	3.243910996	11.585924523	-3.560944479
	H366	-1.737299389	-4.069808554	-0.675670061
	H367	5.991212582	5.860842527	-4.828791653
	H368	-1.264415082	0.042869832	2.434873716
	H369	-1.195299389	-3.612808554	-0.413670061
	H370	-1.288847104	-0.715702418	2.398949387
	H371	-0.782420386	-1.101369312	0.195687023
	H372	1.022046578	-0.196966629	-1.362749889
	Mo373	-0.453869663	-5.347421507	3.093132616
	N374	-2.144266614	-5.400650807	-7.896339333
	N375	-2.248008796	-2.052710728	-9.563058834
	N376	-4.846069897	-0.915541355	-9.569291435
	N377	-6.037254624	-2.033841508	-7.276901727
	N378	-4.403410268	-0.935113590	-5.199486580
	N379	-5.017784091	1.822838533	-5.558068053
	N380	-7.892647184	1.956205699	-5.307133351
	N381	-8.704973798	0.389982521	-3.041026484
	N382	-6.987181433	0.816343550	-0.907935357
	N383	-5.729890262	3.521706790	-0.741918658
	N384	-8.015791912	5.096012669	-1.616469622
	N385	-11.021002256	-7.270453838	3.339743847
	N386	-8.815501236	-6.265696372	4.501039505
	N387	-4.490822063	-1.748287922	4.389013477
	N388	-5.428594899	0.325626434	3.839946772
	N389	-3.187469426	0.163924757	4.453730363
	N390	-7.884857529	-4.935578298	7.590521072
	N391	1.778364678	-3.228974864	-6.080000369
	N392	0.128368220	-6.009346387	-2.582096288
	N393	2.488545875	-0.702779225	-5.950184786
	N394	2.114645076	0.964385427	-8.123353000
	N395	-0.277403718	2.240130734	-7.582961666
	N396	-0.111372240	3.689509514	-5.194927554
	N397	2.813745345	2.160464418	-2.438912424
	N398	1.388099487	0.639818913	-1.868960641
	N399	0.791735657	6.296716479	-5.454020085
	N400	4.043332054	8.498700224	-8.094097705

	N401	3.760735983	10.688892795	-7.887912700
	N402	-2.589407727	2.330819742	9.517838504
	N403	3.021450942	5.600869100	7.671424485
	N404	6.163167579	5.273818323	6.021053251
	N405	6.484434060	4.524763712	3.339284009
	N406	7.420257504	3.336526512	-1.374140496
	N407	9.472004492	4.281045240	-0.754844917
	N408	8.015880222	5.450932245	-2.127902372
	N409	3.983433316	5.001367555	2.047466421
	N410	3.616362890	7.737532512	2.622936018
	N411	5.327508788	9.789960469	1.674828116
	N412	7.289237300	7.638746523	0.389699466
	N413	4.975216456	11.203512227	-0.674509408
	N414	6.082852553	-9.654249831	3.711762340
	N415	5.190349642	-6.418283282	2.391624641
	N416	6.261930583	-3.643545489	1.739166797
	N417	7.083988653	-2.158158541	4.052510177
	N418	7.331903061	-7.461081707	-1.715803678
	N419	7.130758471	-4.918365239	-3.009017021
	N420	0.629010024	-9.831900827	7.190848842
	N421	0.475754956	-7.550093302	3.457143225
	N422	1.706506636	-9.287394835	2.874167944
	N423	-2.311564787	-8.941219670	7.940751864
	N424	0.815845224	2.114184306	0.727295662
	N425	0.872410976	1.162540451	1.339008155
	O426	-2.973147255	-2.681133108	-7.497957969
	O427	-4.075864406	0.710563232	-8.168577843
	O428	-7.522047101	-0.297190210	-7.256809005
	O429	-6.261300094	-1.002617228	-3.889684294
	O430	-4.622211171	2.102857866	-3.335724467
	O431	-7.549654914	3.985830621	-4.314358664
	O432	-10.622376272	1.647917973	-2.931328164
	O433	-8.260686943	-0.498171250	0.486253905
	O434	-6.312096588	3.170695678	1.439743154
	O435	-7.585926658	6.337707142	0.234710142
	O436	-11.099187588	6.204755042	-0.160412335
	O437	-7.942490288	-8.353533223	4.979877389
	O438	-9.644606151	-4.210802299	6.298529393
	O439	-7.704186980	-2.908677520	10.552946128
	O440	0.370243957	0.084463546	-5.554303901
	O441	1.632888551	-5.317252493	-4.141679284
	O442	3.246908502	2.672013771	-7.110972440
	O443	5.222005927	1.800400206	-5.479977811
	O444	-0.113588400	3.483938599	-9.508582886
	O445	-2.285577797	4.364321328	-5.375000375
	O446	-0.017032538	6.811911565	-3.378754797
	O447	0.002076561	9.737056130	-5.498864786
	O448	-5.636305615	0.573197336	9.704265222
	O449	-1.474506622	5.830701502	4.208593935
	O450	4.432609696	6.554766855	5.271616743
	O451	7.791070454	6.373104354	2.948541076
	O452	5.180205642	5.274719082	0.115166990
	O453	2.208121739	7.914769388	0.825745755
	O454	1.340498224	5.743922925	3.434220864
	O455	4.283967732	11.335298142	3.004106712
	O456	6.834806328	12.474209840	-0.308490717
	O457	7.746247306	9.192589389	-1.224914685
	O458	4.080253944	14.080613331	-2.634278963
	O459	5.580657318	5.835792212	-3.942771566

	O460 5.064462359 -8.492933300 1.407039604<br>O461 6.986310320 -4.937005499 -0.008900421<br>O462 8.811018492 -1.261021167 2.854284530<br>O463 8.757917285 -1.784812950 7.249675917<br>O464 6.822925369 -6.097931845 -4.937461747<br>O465 1.998906711 -8.406342621 -2.173468087<br>O466 2.913241794 -8.362326856 -0.140786440<br>O467 8.821591758 -1.783274614 -3.085451378<br>O468 -0.873787655 -7.586704386 9.046671673<br>O469 -3.288085336 -8.564416569 11.371686715<br>O470 -2.800646949 -6.077162137 -1.430732421<br>O471 -4.782765012 -6.996939215 -1.040775944<br>O472 -3.333567992 -11.394461653 3.070045985<br>O473 -2.605436164 -11.191222498 0.941790593<br>O474 -2.350334913 -6.128774623 4.031308777<br>O475 -4.404460402 -6.918092637 3.550711193<br>O476 -1.422030458 -6.476927613 1.600974835<br>O477 -5.299599990 -6.157637522 7.649867837<br>O478 -3.336217435 -4.681389743 6.236688632<br>O479 5.219869769 3.335687408 -3.210508973<br>O480 -4.294621187 -10.920099143 5.965954641<br>O481 -5.295297965 -8.235403621 5.770695711<br>S482 4.048779247 2.773874258 4.838577823<br>S483 3.333670021 -0.166661311 2.419528350<br>S484 1.415788677 -4.854956569 1.688444216<br>S485 0.564335009 1.124068563 4.163489132<br>S486 0.479079375 -1.607715660 0.358454117<br>S487 4.056555925 -3.773702862 4.178822556<br>S488 -1.770244513 -3.356476715 2.710487490<br>S489 2.831321081 -0.751714632 6.108972689<br>S490 0.462279832 -4.940417987 5.264592646<br>S491 -1.046415628 -1.641954819 6.074048418

6 (from 5) 35 (S=2)

6 (from 5) 35 (S=2)	bm22b2n2x2h2n6h2xd35tf.car_6
Fe(139) -2.411<br>Fe(140) -1.019<br>Fe(141) -2.301<br>Fe(142) 3.032<br>Fe(143) 2.928<br>Fe(144) 2.089<br>Fe(145) 1.260	C1 -2.478851508 -3.296366077 -8.506084303<br>C2 -2.152993135 -4.754055772 -8.847135715<br>C3 -2.398997735 -0.961969406 -9.266271875<br>C4 -3.832498797 -0.529083732 -8.963650780<br>C5 -6.218633933 -0.934402273 -9.289783040<br>C6 -6.649693675 -1.235918571 -7.850168925<br>C7 -6.279780236 -2.604215215 -5.852952011<br>C8 -5.669344648 -1.583033843 -4.888820249<br>C9 -3.808399872 -0.095064427 -4.381191621<br>C10 -4.552785018 1.247732040 -4.444349953<br>C11 -5.773143567 2.862898973 -5.781668014<br>C12 -7.171017225 2.841620078 -5.146395697<br>C13 -9.313991862 1.669443491 -4.973479428<br>C14 -9.601613412 1.139819419 -3.562792813<br>C15 -8.942173722 -0.420980125 -1.778982158<br>C16 -8.004125231 -0.042672300 -0.628842511<br>C17 -5.964808018 1.227952456 0.003595446<br>C18 -6.053699681 2.754474877 0.179939687<br>C19 -4.541418795 0.735047975 -0.359645680<br>C20 -3.475640187 1.432890478 0.491395874<br>C21 -4.455192193 -0.790489527 -0.236549361<br>C22 -5.753963288 4.969361360 -0.855146617<br>C23 -7.189614522 5.525538684 -0.880266940

	C24	-4.853544149	5.566985116	-1.964026805
	C25	-4.985314303	7.092568209	-2.032399418
	C26	-3.388406446	5.167396317	-1.741211072
	C27	-9.357905293	5.570420771	-1.964913122
	C28	-10.142366919	5.463953672	-0.672610117
	C29	-9.972348291	-8.101612979	3.996724371
	C30	-8.813935813	-7.455939994	4.757895308
	C31	-7.857313273	-5.236488281	5.405293792
	C32	-8.549066040	-4.545603680	6.596200509
	C33	-7.299809136	-4.224886703	4.388843215
	C34	-6.296042037	-3.232047726	4.980726495
	C35	-5.516440982	-2.521981168	3.871044082
	C36	-4.547922776	-0.245140353	4.241928375
	C37	-8.429454231	-4.099190152	9.006269805
	C38	-7.691136230	-2.864560999	9.480324141
	C39	0.738480148	-2.627382499	-5.482248453
	C40	1.150024455	-1.157689073	-5.676865047
	C41	0.397118983	-2.846947012	-3.991732424
	C42	-0.344403834	-4.160466614	-3.664862202
	C43	0.557416236	-5.349557627	-3.337359152
	C44	3.098900901	0.321713293	-5.906674129
	C45	2.811310191	1.178249211	-7.144763030
	C46	4.618523855	0.237282415	-5.651371829
	C47	1.538046044	1.428781197	-9.214797707
	C48	0.306138052	2.255790088	-8.822718555
	C49	-1.471284074	2.646243543	-7.171809690
	C50	-1.317983030	3.493431178	-5.910004203
	C51	0.171673667	4.336362755	-4.144680863
	C52	0.296610449	5.853141184	-4.428127434
	C53	1.477525686	3.934306818	-3.446501925
	C54	1.562230924	2.547979719	-2.893989344
	C55	0.639463806	1.617548986	-2.478662832
	C56	2.656536247	0.807599260	-2.024926580
	C57	1.217059866	7.513503603	-6.006554525
	C58	0.016353896	8.453577341	-5.961841474
	C59	1.816197872	7.455966576	-7.435213698
	C60	2.699626009	8.603717919	-7.828443011
	C61	2.472581183	9.966850681	-7.806939713
	C62	4.499951327	9.534952856	-8.589823348
	C63	-3.710122382	2.248134434	8.931484753
	C64	-4.301041444	1.054490876	9.659517053
	C65	-3.484251724	1.808336897	7.464448611
	C66	-2.960307559	2.914345455	6.579380100
	C67	-3.832464301	3.744181249	5.850697848
	C68	-1.577261819	3.127050390	6.434466821
	C69	-3.345941250	4.741897133	4.996639185
	C70	-1.078130030	4.130096570	5.603236723
	C71	-1.963893346	4.932632058	4.877283981
	C72	3.909693954	4.962514903	6.651370460
	C73	4.847437765	5.792183912	5.765960289
	C74	3.012481210	4.077578189	5.774735186
	C75	7.155869343	6.132525725	4.995477677
	C76	7.157173181	5.762513204	3.511647050
	C77	6.315277449	4.233874684	1.769208572
	C78	5.109720816	4.921486788	1.091640807
	C79	6.247689651	2.696610762	1.700120845
	C80	6.124004151	2.056798691	0.313472004
	C81	7.373642207	2.094776529	-0.584850620
	C82	8.281468760	4.225699330	-1.542398048

	C83	2.835968777	5.796404052	1.267012567
	C84	2.909531631	7.333896952	1.349286682
	C85	1.508607579	5.344103208	1.886086277
	C86	3.461913983	9.244642242	2.773097062
	C87	4.439319867	10.290108001	2.241397516
	C88	6.422978804	10.748219360	0.853731196
	C89	6.088895473	11.552758918	-0.420572901
	C90	7.743283610	9.965446930	0.662599011
	C91	7.588961719	8.870124996	-0.385531951
	C92	4.637367818	11.745354822	-2.391409123
	C93	4.185230660	13.196826539	-2.269004913
	C94	3.533595196	10.897981160	-3.079489895
	C95	4.003449826	9.497865428	-3.402565189
	C96	4.708333534	9.238226834	-4.588162420
	C97	3.803838621	8.435763748	-2.504889784
	C98	5.225774212	7.968501714	-4.861792438
	C99	4.336396628	7.166021878	-2.747294075
	C100	5.049850983	6.950381896	-3.925043496
	C101	5.318180236	-8.261345856	4.125068034
	C102	5.177340219	-7.676177816	2.730525599
	C103	4.939231233	-5.573280709	1.433219630
	C104	6.133686316	-4.667507993	1.123236578
	C105	7.302419642	-2.612070985	1.765932474
	C106	7.769559820	-1.876511190	3.018474639
	C107	7.403462486	-1.264047796	5.355740682
	C108	8.415813060	-1.950806569	6.253868005
	C109	6.458753602	-7.363243358	-2.589374804
	C110	6.852047816	-6.163175431	-3.460281104
	C111	4.946049430	-7.310936464	-2.272038146
	C112	4.439790929	-8.665234399	-1.741843730
	C113	3.041207626	-8.536994481	-1.181536304
	C114	7.528767583	-3.809162117	-3.540071272
	C115	8.660861592	-3.090960382	-2.831167322
	C116	6.324661112	-2.902551586	-3.907396859
	C117	5.370117009	-2.699273722	-2.757106113
	C118	5.594890957	-1.715775694	-1.778945411
	C119	4.275285697	-3.567529428	-2.594019459
	C120	4.771085229	-1.634487929	-0.649907432
	C121	3.456682763	-3.491074488	-1.462979083
	C122	3.709379174	-2.531013689	-0.479512004
	C123	-0.056559296	-8.357230154	7.265924026
	C124	-1.093752108	-8.029176018	8.352690127
	C125	-0.653129870	-8.246355196	5.853782872
	C126	0.319635682	-8.370007290	4.721840414
	C127	1.081400334	-9.470092146	4.382131251
	C128	1.306675675	-7.906727943	2.829901103
	C129	-3.297734805	-8.393420681	9.313130879
	C130	-2.750692803	-8.607826143	10.707885984
	C131	-3.682690058	-6.272754713	-0.385912070
	C132	-3.676193757	-5.840817826	1.056098603
	C133	-2.758160066	-6.733382331	1.990733840
	C134	-3.031528264	-8.230547107	1.671583533
	C135	-2.331537090	-9.185090632	2.651386155
	C136	-2.792424229	-10.610170706	2.537562212
	C137	-3.245345207	-6.481737682	3.453209594
	C138	1.114418461	-2.020018830	3.649858632
	Fe139	1.860176541	-3.877040529	3.884787765
	Fe140	1.387223090	-0.076456080	2.319179411
	Fe141	0.726884162	-0.627719444	4.974603261

Fe142	3.014789183	-1.601655481	4.227589098
Fe143	2.668498228	1.068735862	4.362204759
Fe144	-0.437284477	-2.855272611	4.562366120
Fe145	0.264737241	-2.881891706	2.157156536
H146	-3.033373066	-5.587756812	-7.207455135
H147	-1.411085882	-5.460005889	-7.077353481
H148	-1.747094653	-2.695311534	-10.333713419
H149	-1.175120295	-4.793477245	-9.353179287
H150	-2.895220051	-5.079155831	-9.597256131
H151	-1.791157407	-0.624782538	-8.414088510
H152	-4.576338362	-2.015865966	-10.165910337
H153	-2.060645449	-0.428949027	-10.165003018
H154	-6.840634643	-1.522833434	-9.977061392
H155	-5.245843978	-2.730703648	-7.713011009
H156	-6.422043559	0.129575518	-9.466845824
H157	-3.788208101	-0.392809649	-3.324705253
H158	-3.910228088	-1.542767902	-5.974952468
H159	-5.862580048	-3.599145644	-5.645951002
H160	-5.209015904	3.671940934	-5.301417845
H161	-4.840839127	1.055501227	-6.486328787
H162	-7.358098829	-2.628812200	-5.653048511
H163	-2.771644070	0.039222188	-4.723194112
H164	-7.565638428	0.999013054	-6.001861940
H165	-5.876320889	3.092791866	-6.851214779
H166	-5.215165889	-1.286910856	-0.858440504
H167	-3.465982695	-1.148889524	-0.560258998
H168	-4.596034737	-1.111806528	0.807283272
H169	-4.365765637	1.006587192	-1.413460490
H170	-9.851906881	1.026461591	-5.685700772
H171	-3.242430055	4.078238322	-1.768130420
H172	-7.668488823	4.438397330	-2.593202455
H173	-6.013923235	7.413869673	-2.250066428
H174	-2.476244560	1.064338119	0.220171154
H175	-3.617183265	1.227504725	1.561311933
H176	-4.473894450	3.050172478	8.931513755
H177	-2.754413110	5.609933892	-2.523444330
H178	-4.334655640	7.484497925	-2.827661189
H179	-3.035318378	5.541651050	-0.766399679
H180	-4.677927364	7.555504740	-1.080866310
H181	-5.188262634	5.147933268	-2.929987472
H182	-5.357912132	5.274535236	0.125007177
H183	-5.414122551	3.053728479	-1.769533213
H184	-3.479422964	2.522751988	0.342851178
H185	1.490647352	4.243235246	1.918153105
H186	0.705177754	5.688205740	1.214235799
H187	0.416725793	5.833924877	3.439695697
H188	-2.131145345	6.359249887	3.571744760
H189	-0.003571409	4.269613288	5.489014429
H190	-0.871237654	2.472292835	6.950139082
H191	-4.913777932	3.604046190	5.936049543
H192	-6.866524795	1.036079676	-1.932484857
H193	-8.870656640	-1.511637923	-1.907509759
H194	-9.964046990	-0.186939789	-1.453408066
H195	-7.955443454	-0.125850195	-3.639395152
H196	-9.755661426	2.672837702	-5.019740261
H197	-4.039607115	5.361369432	4.421928621
H198	-6.261377476	0.804788029	0.972222226
H199	-9.369330273	6.620699559	-2.300006290
H200	-9.889227171	4.965384198	-2.716982782

H201	-9.819142657	4.642852408	0.008829404
H202	-8.433946513	-4.850292941	9.810384586
H203	0.688777607	-7.002455442	-2.138331982
H204	-5.451554704	-6.974378990	-0.084240292
H205	4.414592617	-9.390050568	-2.568250085
H206	5.122255799	-9.044860790	-0.967448290
H207	4.373904562	-7.044145762	-3.173496819
H208	9.356253990	-3.765333619	-2.278478378
H209	6.629168536	-8.227281448	-3.249169330
H210	1.327835090	-2.785853086	-3.406119579
H211	-0.977213983	-4.460890496	-4.518531297
H212	7.084658448	-4.962631859	-1.785665128
H213	7.979015167	-4.159565119	-4.489847060
H214	7.129824541	-6.840283599	-0.695726293
H215	-0.742443171	-6.017749359	-1.851634559
H216	4.766771340	-6.510186215	-1.537916181
H217	7.251445458	-8.446415647	-1.037010723
H218	1.624630173	-3.852929789	-6.891624868
H219	-0.239506133	-2.007675636	-3.675540192
H220	3.828875980	-8.385914890	0.622090276
H221	-1.033119577	-3.985894993	-2.826889746
H222	-0.684678877	4.222486737	-3.465391898
H223	-0.444345466	1.627087115	-2.497780706
H224	-1.792440332	3.320773900	-7.977013434
H225	0.050268245	1.150869184	-7.072260123
H226	0.908295884	5.418253157	-6.335306836
H227	1.629246493	10.567606633	-7.483121090
H228	-0.940719015	7.979971990	-6.290849992
H229	0.995662701	7.315018316	-8.160232512
H230	1.973901608	7.873264374	-5.292341677
H231	5.491230868	9.718625470	-8.997625990
H232	1.627071595	4.646584424	-2.618327463
H233	2.316929132	4.092179453	-4.143837323
H234	3.799524753	11.535823200	-8.400973483
H235	2.443876872	6.552940401	-7.488759002
H236	0.678971894	2.997301936	-5.758667182
H237	-2.295652090	1.937973678	-7.002616881
H238	2.294554147	2.134608160	-9.579826173
H239	3.446410344	0.151362271	-1.670208321
H240	1.259728657	0.769619118	-10.047873791
H241	-2.636997386	3.107802468	10.449926022
H242	-1.948896913	3.290109989	8.979320450
H243	3.899412032	4.614208534	2.743828761
H244	2.812995892	5.535096990	0.198141447
H245	3.993250528	7.223432660	3.090143082
H246	3.725054806	2.484497934	-2.805611420
H247	5.322084963	2.597572975	-4.153204147
H248	5.155378743	4.120736748	-3.707336790
H249	4.793749427	-0.284904983	-4.701029225
H250	5.103717377	-0.333110260	-6.460602136
H251	8.048986600	9.553013261	1.635294875
H252	8.507474064	10.675560406	0.322142282
H253	7.402405935	7.368621064	1.084312943
H254	7.056077820	6.901054512	-0.557183761
H255	10.063298990	4.966992746	-0.863317210
H256	7.418786655	1.183692977	-1.199373239
H257	5.753884719	7.782553220	-5.800123135
H258	3.224709432	8.582311311	-1.589204461
H259	7.189959669	5.388916253	-2.855852768

	H260	5.391724574	2.351128328	2.300676526
	H261	5.915490056	4.151595933	3.866962573
	H262	5.264507050	2.472527797	-0.235728701
	H263	1.848045105	-0.359738017	-8.059565446
	H264	8.156049774	5.924094491	5.399225068
	H265	2.995104749	-1.881353409	-5.951271942
	H266	7.154490531	2.309687588	2.193564995
	H267	4.224556900	6.368736033	-2.009634134
	H268	4.711921837	2.106504662	-6.207819131
	H269	6.994098188	7.218532204	5.041526805
	H270	9.777946440	3.313919248	-0.470011221
	H271	8.813055464	5.916707564	-2.565446228
	H272	1.860892349	-4.344392604	-5.313556992
	H273	-0.200212064	-2.742466889	-6.056214767
	H274	4.857366473	10.038999760	-5.316927643
	H275	2.649926779	0.901659458	-5.082875833
	H276	7.192662048	4.600675112	1.219369961
	H277	8.279929662	2.096375416	0.033592235
	H278	5.881035353	0.999450870	0.492684900
	H279	6.603165945	3.278145478	-2.178132979
	H280	2.456598829	9.575637506	2.472391537
	H281	5.412135785	8.868306243	1.128080047
	H282	6.582516079	11.504365290	1.636137620
	H283	3.516164918	9.291274357	3.869245613
	H284	4.426738964	10.407880877	-0.723831014
	H285	5.844236759	-7.549421018	4.777925334
	H286	4.283580094	-8.351015851	4.515871023
	H287	0.728271910	-7.595967138	7.380015490
	H288	5.933342998	-10.011530681	4.969617913
	H289	5.620230135	-10.123425368	3.365655272
	H290	2.385602423	3.467831584	6.437367252
	H291	2.602543611	6.506916327	6.900222961
	H292	6.453423553	4.667427117	6.386066626
	H293	2.372994186	4.717604647	5.149340227
	H294	3.647280280	6.342153112	8.147548808
	H295	6.486191397	-1.080290811	5.936407344
	H296	4.509848836	4.315823157	7.310583815
	H297	-8.140324058	-3.683678652	3.923127672
	H298	-7.036468625	-5.867461674	5.766800650
	H299	6.197371376	-2.574843344	4.197638942
	H300	-3.497363382	1.470000874	4.636479712
	H301	8.172851848	-3.135772649	1.351174892
	H302	2.626956172	-4.190841170	-1.337895467
	H303	7.030968791	-1.830363501	1.036590230
	H304	-6.190178497	-1.996583248	3.180001334
	H305	5.425910499	-3.384765034	2.543259714
	H306	-4.969188659	-3.265046458	3.269861710
	H307	-6.810974438	-4.815776213	3.597094644
	H308	-5.568322874	-3.753724252	5.620933653
	H309	-6.811415158	-2.486079144	5.608085235
	H310	5.799574399	-3.378101768	-4.747899915
	H311	-9.643474458	-5.697566650	4.291563697
	H312	-9.529806494	-8.535981700	3.087255517
	H313	3.090537500	-2.480705123	0.418583949
	H314	-11.752707809	-7.123884533	4.338247772
	H315	-11.485811894	-7.459222437	2.757421776
	H316	-3.640279925	-1.975797448	4.723435690
	H317	4.948147868	-0.885869996	0.126329341
	H318	4.065570046	-4.322166234	-3.355152546

	H319	-4.684254505	-9.808692938	6.232121033
	H320	-1.419999567	-9.031875039	5.733108014
	H321	-4.879233076	-7.561667635	5.282020335
	H322	-3.962395661	-10.917906627	5.440705222
	H323	8.778943470	-2.943821057	5.892198749
	H324	-3.013008415	-5.026249943	5.654447898
	H325	-4.696761501	-5.839368266	1.465415844
	H326	5.113726523	-5.786722990	3.528668015
	H327	-4.118964102	-8.399554513	1.711840750
	H328	2.303286603	-9.762782808	2.645354275
	H329	1.620114912	-7.424730494	1.911671782
	H330	-10.318983922	-8.948370895	4.613416970
	H331	-2.596709850	-0.026868428	4.860833370
	H332	-4.581086100	-5.325040652	7.466442688
	H333	-2.568093392	-3.909848136	6.635623608
	H334	-6.961422705	-5.125088599	7.808504652
	H335	-6.565140121	-0.059236295	3.890447850
	H336	-2.879018496	-12.070149906	1.337328766
	H337	-1.247033214	-9.147099056	2.473834478
	H338	-2.517993123	-8.881561882	3.687336564
	H339	-2.686406997	-8.436576747	0.647630268
	H340	4.013951594	-4.984851249	1.532275687
	H341	6.439974860	-1.031806725	-1.897001144
	H342	6.740797628	-1.945791519	-4.258484873
	H343	-3.265927310	-4.824084966	1.122128346
	H344	-5.285826730	-6.698829222	7.301906572
	H345	-6.251759805	-8.081150695	5.810655512
	H346	-5.664366295	1.391734510	3.717298369
	H347	1.225490961	-10.420800373	4.881457792
	H348	-1.174587811	-7.285867263	5.759167595
	H349	4.824801967	-6.281238555	0.609515860
	H350	7.819073885	-0.286485646	5.064984185
	H351	-4.450727977	1.449822921	7.079804984
	H352	-2.784677445	0.957296167	7.452776381
	H353	1.006713426	-9.680550548	8.418163445
	H354	-0.158347741	-10.398778495	7.522678018
	H355	-4.121514393	-9.111471680	9.172093246
	H356	-2.565862530	-9.254483611	7.505954373
	H357	-1.895570627	-9.322896318	10.772931572
	H358	-3.722327215	-7.380048012	9.228448100
	H359	-3.542816511	0.312999259	10.011527601
	H360	3.858639563	13.509521560	-1.248130432
	H361	-9.473736244	-3.799587454	8.810485273
	H362	-7.182841605	-2.277241028	8.678065586
	H363	5.536315962	11.742931187	-3.029299759
	H364	2.645786189	10.866777942	-2.427878487
	H365	3.245006973	11.426721345	-3.999079622
	H366	-1.738674981	-4.087508115	-0.537991323
	H367	6.013564914	5.659014019	-5.022694020
	H368	-1.780852077	0.014914334	2.416938235
	H369	-1.196674981	-3.621508115	-0.291991323
	H370	-2.026720331	-0.681931455	2.283598283
	H371	-0.788810747	-0.796182044	0.480020809
	H372	0.960095181	-0.238212095	-1.389997231
	Mo373	-0.470947722	-5.219969798	3.293999728
	N374	-2.161617442	-5.687371302	-7.733670429
	N375	-2.233909086	-2.389797328	-9.494499134
	N376	-4.822412488	-1.239297038	-9.556825619
	N377	-6.040587389	-2.290328293	-7.252729013

	N378	-4.423913412	-1.136006625	-5.192317074
	N379	-5.050687361	1.607379373	-5.651367686
	N380	-7.920100658	1.733837614	-5.383472205
	N381	-8.710298384	0.241691354	-3.051890016
	N382	-6.964042876	0.766874636	-0.955388414
	N383	-5.743310020	3.507206229	-0.914394997
	N384	-8.014693378	5.038811664	-1.841392539
	N385	-11.028169626	-7.154427899	3.617837129
	N386	-8.824976386	-6.110022088	4.751957454
	N387	-4.522563658	-1.577078229	4.385651606
	N388	-5.656570490	0.377705747	3.773274597
	N389	-3.444530184	0.463771244	4.499300674
	N390	-7.889274973	-4.651074245	7.777324611
	N391	1.807482227	-3.522057707	-5.942097131
	N392	0.116441550	-6.186506180	-2.377883356
	N393	2.478013744	-0.986912145	-5.918730371
	N394	2.094200728	0.624238766	-8.141418249
	N395	-0.281941837	1.948408830	-7.628822928
	N396	-0.072541976	3.542939614	-5.342289872
	N397	2.808148578	2.007654732	-2.602561799
	N398	1.346149046	0.549476113	-1.961060661
	N399	0.791158995	6.163436617	-5.653142719
	N400	3.969939401	8.348469674	-8.320436516
	N401	3.629232947	10.539836303	-8.290613821
	N402	-2.445939961	2.619434566	9.571747066
	N403	3.058849998	5.810935442	7.499346450
	N404	6.167468791	5.456493573	5.812139218
	N405	6.486595190	4.643306570	3.161264792
	N406	7.395161579	3.232526916	-1.512367722
	N407	9.402197697	4.200043241	-0.790268975
	N408	8.034093236	5.328142322	-2.282813798
	N409	3.995585326	5.117426350	1.846168977
	N410	3.659024683	7.860795936	2.359837431
	N411	5.350928429	9.870821402	1.314670691
	N412	7.251560244	7.648121343	0.107654090
	N413	4.968808991	11.186549301	-1.090218317
	N414	6.054344171	-9.527909203	4.077014862
	N415	5.150773056	-6.331189871	2.660214540
	N416	6.224588820	-3.573853903	1.909490355
	N417	7.067858376	-2.029036564	4.169193988
	N418	7.358177971	-7.512699900	-1.438225420
	N419	7.179350585	-5.014099158	-2.805565416
	N420	0.566914174	-9.675201973	7.493421464
	N421	0.481400299	-7.396270581	3.737906990
	N422	1.694675668	-9.156805318	3.188341883
	N423	-2.298722670	-8.662218324	8.292438271
	N424	1.531125601	2.226073697	0.532569170
	N425	1.483228626	1.311635980	1.200996595
	O426	-2.978714011	-2.957447542	-7.417860472
	O427	-4.053783547	0.439561038	-8.218027885
	O428	-7.528478636	-0.556646527	-7.294704892
	O429	-6.291147496	-1.177247667	-3.894540953
	O430	-4.677532337	1.961250195	-3.436199261
	O431	-7.590614261	3.812451584	-4.493939215
	O432	-10.626099931	1.505690224	-2.971682496
	O433	-8.212680843	-0.491999856	0.507286570
	O434	-6.376531650	3.255180535	1.267773909
	O435	-7.571598878	6.376468517	-0.060455161
	O436	-11.102654521	6.172973446	-0.412979576

	O437 -7.952057954 -8.180665522 5.299769607<br>O438 -9.634409738 -3.957044956 6.448949267<br>O439 -7.652588733 -2.511047793 10.647739167<br>O440 0.338379288 -0.210932308 -5.623657466<br>O441 1.636137269 -5.542039802 -3.942920942<br>O442 3.203061975 2.366350443 -7.163969062<br>O443 5.202802470 1.545958548 -5.551534209<br>O444 -0.104411972 3.135143727 -9.588607260<br>O445 -2.284402702 4.105432547 -5.448734644<br>O446 0.057312987 6.689969655 -3.553300015<br>O447 0.073532108 9.628250726 -5.642255556<br>O448 -5.497629320 0.878307055 9.837313544<br>O449 -1.419485007 5.891735806 4.046706339<br>O450 4.431113038 6.722076786 5.050433305<br>O451 7.787459894 6.482350970 2.709694878<br>O452 5.202026778 5.284881384 -0.094906204<br>O453 2.270641774 8.029369872 0.546019187<br>O454 1.361392314 5.909709122 3.191101496<br>O455 4.366667503 11.459183278 2.639911703<br>O456 6.824771770 12.478419274 -0.789486191<br>O457 7.667282451 9.107031136 -1.601124556<br>O458 4.159415238 13.964622413 -3.217238236<br>O459 5.615083299 5.676666106 -4.130943897<br>O460 5.060638869 -8.433038851 1.733149458<br>O461 6.959262055 -4.946067852 0.226782612<br>O462 8.764174461 -1.137115663 2.924465604<br>O463 8.812351392 -1.466672184 7.301146162<br>O464 6.849415358 -6.239617380 -4.700466677<br>O465 2.031027707 -8.536036528 -1.878671154<br>O466 2.936865308 -8.373990727 0.151341799<br>O467 8.856439448 -1.885099011 -2.880135692<br>O468 -0.820345514 -7.272500643 9.302874979<br>O469 -3.241619455 -8.107960232 11.709710347<br>O470 -2.778037652 -6.113639143 -1.194955668<br>O471 -4.810714026 -6.922069361 -0.818960785<br>O472 -3.335263501 -11.261798338 3.426772932<br>O473 -2.541907332 -11.149152783 1.314986792<br>O474 -2.356940661 -5.996228923 4.254949276<br>O475 -4.412923293 -6.791421563 3.791286690<br>O476 -1.425622987 -6.409104248 1.840932616<br>O477 -5.299200056 -5.879760497 7.863644586<br>O478 -3.343764190 -4.450048097 6.390746305<br>O479 5.207098129 3.194583624 -3.363014862<br>O480 -4.287468112 -10.709959700 6.340759818<br>O481 -5.292765900 -8.030450094 6.061249235<br>S482 3.973724619 2.961931114 4.650239113<br>S483 3.653755002 -0.107975369 2.626996832<br>S484 1.393242811 -4.748027691 1.852016946<br>S485 0.485839872 1.289173580 3.880617630<br>S486 0.451499736 -1.370011505 0.518517689<br>S487 4.020956045 -3.611486153 4.427906283<br>S488 -1.805885442 -3.272398037 2.820383634<br>S489 2.622860538 -0.374945840 6.141013125<br>S490 0.443154672 -4.700505711 5.446145667<br>S491 -1.154128314 -1.308289127 5.973518292

7 35 (S = 1/2)

7 35 (S = 1/2)	bm22b2n2x3b4to3td35b.car_2

Fe(139) -2.198<br>Fe(140) -1.751<br>Fe(141) -2.571<br>Fe(142) 2.925<br>Fe(143) 2.996<br>Fe(144) 2.087<br>Fe(145) -1.159	C1 -7.353927423 -5.382585655 2.424006955<br>C2 -7.744835320 -6.540481666 1.499149250<br>C3 -8.138328591 -3.423326352 3.678645205<br>C4 -7.479173635 -3.681443244 5.031808044<br>C5 -7.156345486 -5.213269615 6.907361546<br>C6 -5.654102681 -5.496024430 6.795765984<br>C7 -3.817977737 -6.216808496 5.341200441<br>C8 -3.060603050 -4.903466352 5.124872284<br>C9 -3.081048282 -2.648981413 4.204715825<br>C10 -2.947850646 -1.861143551 5.516483280<br>C11 -3.933165533 -1.231468622 7.644511669<br>C12 -2.938828654 -1.825257170 8.648999032<br>C13 -2.158907450 -3.831057101 9.811424330<br>C14 -0.704103609 -4.148930558 9.446275173<br>C15 0.849154412 -4.876839264 7.686050320<br>C16 1.665074157 -3.922198251 6.811881035<br>C17 1.669317009 -1.797037281 5.543086107<br>C18 1.836680494 -0.457253782 6.284254211<br>C19 0.956381092 -1.653722331 4.171336349<br>C20 1.556265180 -0.518037068 3.334318748<br>C21 0.994720288 -2.979501476 3.403071643<br>C22 0.720561433 1.416429153 7.437222170<br>C23 1.104122475 1.214946615 8.917781387<br>C24 -0.618146856 2.174203126 7.265098319<br>C25 -0.666014263 3.431098062 8.140393487<br>C26 -0.848471115 2.540392125 5.792828895<br>C27 0.678844772 0.076036288 11.021027204<br>C28 2.136729556 -0.209651622 11.317329376<br>C29 6.721914038 -11.112654463 3.280147483<br>C30 7.149017746 -9.895053113 2.461139317<br>C31 7.539848981 -7.431049650 2.637420086<br>C32 8.894732276 -7.012556040 3.238996733<br>C33 6.442115606 -6.405570271 2.975248903<br>C34 6.763403453 -4.982545696 2.508099570<br>C35 5.526889787 -4.090222941 2.600344960<br>C36 5.370826383 -1.592039654 2.592806615<br>C37 11.126648418 -6.129487510 2.745080348<br>C38 11.373420307 -4.646299098 2.573352421<br>C39 -5.391227792 -2.814383664 -0.634251609<br>C40 -5.729563998 -1.381158083 -0.188786456<br>C41 -3.860946983 -2.911786683 -0.813070573<br>C42 -3.280484028 -4.334330897 -0.947496423<br>C43 -3.225499611 -4.891130829 -2.369050333<br>C44 -6.527198515 0.793154379 -0.997005890<br>C45 -7.603677908 1.193538543 0.015729199<br>C46 -6.791537273 1.490992551 -2.347188614<br>C47 -9.269022811 0.473711918 1.645818861<br>C48 -8.559440077 0.674469893 2.992199311<br>C49 -6.470585614 0.457643889 4.270763682<br>C50 -5.308258204 1.447672039 4.236834644<br>C51 -4.102045042 3.190612374 2.994979770<br>C52 -4.404934022 4.522610592 3.720455371<br>C53 -3.815429232 3.568802919 1.535366133<br>C54 -3.281970747 2.490887241 0.650212887<br>C55 -2.629476617 1.303564287 0.874442490<br>C56 -2.720119243 1.613586594 -1.323563657<br>C57 -6.176605303 6.146349629 4.256719799<br>C58 -5.774046700 6.390871749 5.709207073<br>C59 -7.716335889 6.190750799 4.101870076

	C60	-8.337531082	7.554976438	4.015324798
	C61	-8.162071941	8.679818616	4.800019578
	C62	-9.653765131	9.072939814	3.209055093
	C63	9.591660683	1.509232399	2.104144916
	C64	10.480194282	0.313848423	1.812502795
	C65	8.125893925	1.010092598	2.029245282
	C66	7.116677166	2.089902768	2.344463985
	C67	6.714531514	2.351382750	3.667202252
	C68	6.542828788	2.861922408	1.317451490
	C69	5.766447197	3.339102310	3.957033165
	C70	5.603412406	3.856742987	1.592072335
	C71	5.212268748	4.091792830	2.913279640
	C72	5.168910529	7.164428313	-2.318625887
	C73	4.048409541	8.195590482	-2.503131636
	C74	4.585947470	5.845947367	-1.791861748
	C75	2.666945606	9.436559156	-4.112163924
	C76	1.247671903	8.902669840	-3.909371845
	C77	-0.177557272	6.925082138	-3.530267974
	C78	-0.495986874	6.835364765	-2.022586015
	C79	-0.213644579	5.555220517	-4.235248525
	C80	-1.496992304	4.726261061	-4.123721429
	C81	-2.725176083	5.204746206	-4.918716881
	C82	-3.861144383	7.366834524	-4.342372727
	C83	0.308630560	6.560720543	0.266505363
	C84	0.333125068	7.948835391	0.942056757
	C85	1.288668084	5.643894993	1.007550873
	C86	1.475430384	10.110597602	1.060557194
	C87	0.657880953	11.390396203	0.885341924
	C88	-1.283735577	12.467972208	-0.192497710
	C89	-2.456888824	12.793472413	0.757374370
	C90	-1.787488970	12.372597494	-1.654047085
	C91	-2.684200889	11.156476260	-1.852111596
	C92	-3.864369519	11.986996339	2.612939854
	C93	-3.621135028	13.022815331	3.706201672
	C94	-4.186669972	10.606151699	3.245183052
	C95	-4.601815218	9.575989032	2.218039983
	C96	-5.933227749	9.511435031	1.779557333
	C97	-3.672062687	8.692053435	1.648419223
	C98	-6.327146964	8.611379563	0.785283806
	C99	-4.042077536	7.798433258	0.636922311
	C100	-5.368946035	7.774137139	0.213050154
	C101	2.586188058	-4.006183453	-9.529662985
	C102	1.279764174	-3.757132373	-8.794294024
	C103	0.101737441	-2.248597046	-7.214394051
	C104	-0.529171379	-0.929773202	-7.673399464
	C105	-0.224211662	1.504604202	-7.753658143
	C106	0.867572697	2.468998543	-8.210376602
	C107	3.206252004	3.138787980	-8.218387733
	C108	3.538833544	3.205243418	-9.696062639
	C109	-4.125185981	-3.678553432	-8.540659770
	C110	-5.115196500	-2.603296775	-8.073748746
	C111	-3.450744974	-4.329065963	-7.309929922
	C112	-2.787138814	-5.670424393	-7.677347096
	C113	-1.871344506	-6.137541096	-6.569096915
	C114	-5.453061736	-0.278708638	-7.377449499
	C115	-5.131516557	1.010651289	-8.108252179
	C116	-5.488634743	-0.158819776	-5.832728336
	C117	-4.120743091	-0.245996372	-5.199488106
	C118	-3.268378456	0.871168028	-5.144618356

	C119	-3.640935722	-1.478498755	-4.725992019
	C120	-1.964066278	0.743216270	-4.653665929
	C121	-2.334711746	-1.607364312	-4.239611538
	C122	-1.487905250	-0.495700173	-4.211838706
	C123	7.049713579	-6.143567717	-5.850836660
	C124	8.381850508	-6.173964762	-5.079083525
	C125	5.864415904	-6.461253120	-4.924401836
	C126	4.497407784	-6.284184900	-5.513579747
	C127	3.965673159	-6.908918790	-6.624486745
	C128	2.380594398	-5.737902258	-5.612282449
	C129	9.956619021	-7.392697542	-3.682406391
	C130	11.145394595	-7.053458809	-4.555107316
	C131	0.661904827	-7.002465335	0.011286867
	C132	2.114685324	-6.619867690	-0.035777676
	C133	2.751411503	-6.803016243	-1.475052956
	C134	2.529010102	-8.270110463	-1.945313051
	C135	3.313020235	-8.605598430	-3.222657322
	C136	3.338850465	-10.066946888	-3.572280188
	C137	4.290196153	-6.616052491	-1.301241147
	C138	3.222400237	-0.604078365	-2.830967960
	Fe139	3.259067960	-1.891958401	-4.366391413
	Fe140	1.846168865	0.762786987	-1.865548771
	Fe141	4.681766470	0.601522597	-2.163178343
	Fe142	3.232471757	0.692416726	-4.351885570
	Fe143	3.410513225	2.840496298	-2.728487193
	Fe144	4.575767880	-1.951253951	-2.145628192
	Fe145	2.042127462	-1.913715144	-2.138742040
	H146	-5.947549486	-7.504296511	1.520676791
	H147	-6.172920867	-6.584546955	0.192653795
	H148	-9.314835463	-4.765389606	2.501725113
	H149	-8.461161218	-6.173134615	0.746974828
	H150	-8.300725752	-7.266066812	2.117821335
	H151	-7.479030919	-2.723282268	3.145886763
	H152	-8.423618672	-5.496470504	5.189473235
	H153	-9.101473408	-2.924462826	3.852057491
	H154	-7.655550375	-6.109066978	7.298941803
	H155	-5.882247854	-6.057940641	4.833782854
	H156	-7.276558014	-4.403300823	7.638560148
	H157	-2.069731735	-2.745165688	3.789157606
	H158	-4.576972439	-4.171476826	3.920033672
	H159	-3.722867709	-6.844817876	4.444861186
	H160	-3.641539613	-0.186362262	7.475778376
	H161	-4.846680734	-2.433681692	6.111999816
	H162	-3.335563182	-6.732474546	6.179733357
	H163	-3.696408543	-2.079208505	3.493624286
	H164	-3.654036735	-3.734640022	8.285814943
	H165	-4.936933136	-1.245364127	8.091097495
	H166	0.524009093	-3.792129219	3.974915199
	H167	0.459973253	-2.883501408	2.445094211
	H168	2.031701844	-3.272034997	3.174252631
	H169	-0.096440165	-1.403278873	4.379102124
	H170	-2.647390420	-4.780472800	10.074467915
	H171	-0.854328250	1.656275111	5.139530104
	H172	-0.376138437	-0.228604595	9.196751520
	H173	-0.581345966	3.198350140	9.211581595
	H174	0.981978508	-0.394968384	2.403331710
	H175	2.595210630	-0.732596672	3.045668517
	H176	9.787566764	1.816833421	3.150026414
	H177	-1.816787139	3.048391024	5.678260604

	H178	-1.618661520	3.957556727	7.982038840
	H179	-0.059056048	3.224924076	5.442799720
	H180	0.151232401	4.122332316	7.878984130
	H181	-1.429760190	1.497876340	7.589455251
	H182	1.537020384	2.024819559	7.021157461
	H183	-0.216215131	-0.259004913	6.471026256
	H184	1.538355318	0.449347133	3.859165858
	H185	1.338682547	4.677454085	0.478399336
	H186	0.870464723	5.471477922	2.013689653
	H187	3.098518351	5.786308823	1.756598267
	H188	4.041927973	5.108103586	4.081051082
	H189	5.147349820	4.427039764	0.783290933
	H190	6.806623583	2.657532118	0.277151124
	H191	7.134655718	1.764034644	4.488251329
	H192	0.053796207	-2.671698621	6.654594408
	H193	0.733411672	-5.811534811	7.116842638
	H194	1.456014134	-5.097586830	8.573766746
	H195	-1.228065266	-4.486411329	7.467427844
	H196	-2.123108029	-3.203771085	10.711242214
	H197	5.451596409	3.514523831	4.989479124
	H198	2.691357657	-2.162199973	5.377423158
	H199	0.356061664	0.939483859	11.625276541
	H200	0.101049819	-0.801621150	11.351093628
	H201	2.708261008	-0.678166116	10.481947290
	H202	11.906696456	-6.693264019	2.210987686
	H203	-2.077977434	-6.057329626	-3.601065742
	H204	1.204050086	-8.724852496	0.689150693
	H205	-3.565547171	-6.431579420	-7.827735751
	H206	-2.218708575	-5.565220699	-8.613114958
	H207	-4.191559636	-4.504483937	-6.514606765
	H208	-4.801313808	0.864736279	-9.163696522
	H209	-4.771129685	-4.438751589	-9.006554677
	H210	-3.570256805	-2.303500701	-1.684753590
	H211	-3.873933910	-5.043954414	-0.344865010
	H212	-3.604120627	-1.186223861	-7.980210962
	H213	-6.479757970	-0.531896180	-7.709339583
	H214	-2.485613972	-2.584100930	-9.190387783
	H215	-1.398210350	-5.803445764	-1.990370835
	H216	-2.710095077	-3.625096551	-6.900491489
	H217	-2.760054766	-3.963669263	-10.044694098
	H218	-6.972941105	-3.673035955	-1.647876104
	H219	-3.402723332	-2.445879208	0.071639029
	H220	-0.365822582	-5.313317459	-7.544520211
	H221	-2.270971354	-4.347526260	-0.513855459
	H222	-3.201002487	2.783176351	3.474281898
	H223	-2.363625039	0.798188053	1.795306756
	H224	-7.157693891	0.790209239	5.061145801
	H225	-6.782955858	-0.114527101	2.238250017
	H226	-6.412250912	4.125143516	3.583773559
	H227	-7.521130891	8.885690446	5.649576498
	H228	-5.792227454	5.470630086	6.342419315
	H229	-8.178950680	5.595989032	4.908855353
	H230	-5.706794412	6.918649195	3.627675922
	H231	-10.383272624	9.615195752	2.612437361
	H232	-3.084716983	4.393418189	1.558398667
	H233	-4.734024677	3.981383967	1.085548440
	H234	-9.112418870	10.585763601	4.606578573
	H235	-7.965425156	5.680189367	3.158628805
	H236	-5.820208847	2.064777133	2.333874683

	H237	-6.069479314	-0.516911785	4.584798441	
	H238	-9.866131778	1.376692558	1.463382115	
	H239	-2.583364340	1.462702692	-2.390391536	
	H240	-9.963093335	-0.371433313	1.746344655	
	H241	10.719609365	3.030939578	1.321839658	
	H242	9.109247522	3.265966369	1.171978970	
	H243	1.437973095	6.252305815	-1.558986486	
	H244	-0.704515609	6.167405759	0.431136115	
	H245	1.670169651	8.646950357	-0.450050814	
	H246	-3.792582488	3.461302298	-1.212080922	
	H247	-5.572211794	4.094527985	-2.130818577	
	H248	-5.135013835	5.407980098	-1.340823938	
	H249	-5.962121090	1.278345826	-3.035903391	
	H250	-7.725914936	1.109254253	-2.792277435	
	H251	-0.911519960	12.338813695	-2.318774047	
	H252	-2.366497407	13.278753311	-1.870452904	
	H253	-1.123469767	10.013903500	-2.676686561	
	H254	-2.561292805	9.146186096	-2.235382003	
	H255	-3.600400849	9.005036143	-5.543863136	
	H256	-3.319331036	4.335091957	-5.235996803	
	H257	-7.370746744	8.566311276	0.465263911	
	H258	-2.631292486	8.685425906	1.983510020	
	H259	-4.878699786	7.624502613	-2.558008367	
	H260	0.609676658	4.936223419	-3.844476270	
	H261	1.949743810	6.967845113	-3.742448605	
	H262	-1.777914173	4.581639391	-3.067959793	
	H263	-8.255550568	-0.726192835	0.175152012	
	H264	2.766754906	9.742923118	-5.162474011	
	H265	-6.505129658	-1.162542085	-2.020723838	
	H266	0.010815113	5.733413473	-5.300162281	
	H267	-3.292692862	7.162306509	0.160964187	
	H268	-7.321925825	3.063837244	-1.314204143	
	H269	2.749023188	10.337371870	-3.488668705	
	H270	-3.110628906	7.511124971	-6.251579871	
	H271	-4.909137567	8.987792008	-3.634202449	
	H272	-5.519612154	-3.744587701	-2.463895870	
	H273	-5.669424181	-3.454186865	0.224836469	
	H274	-6.683767429	10.169250134	2.224641132	
	H275	-5.601529751	1.217198218	-0.573318344	
	H276	-0.949837808	7.587083556	-3.944357035	
	H277	-2.409650665	5.707681365	-5.841975804	
	H278	-1.236397787	3.727833185	-4.502618668	
	H279	-4.076249824	5.619804683	-3.332661818	
	H280	1.476305825	9.889875120	2.139191941	
	H281	-0.695971847	10.408046639	-0.303774268	
	H282	-0.608358553	13.333860903	-0.120225575	
	H283	2.505195312	10.356644135	0.768833817	
	H284	-2.127302287	11.051061929	1.765037729	
	H285	3.044216704	-3.047631824	-9.815122308	
	H286	3.262045745	-4.472301057	-8.782682489	
	H287	6.950057203	-5.108892341	-6.209002904	
	H288	3.255566089	-5.182392004	-11.053714408	
	H289	1.796853715	-5.634973311	-10.458932908	
	H290	5.405201912	5.119448053	-1.713561656	
	H291	5.746316923	7.945507882	-0.524845835	
	H292	4.217007047	8.029250135	-4.545112331	
	H293	4.151258638	6.014936495	-0.795703051	
	H294	6.661042553	8.464192153	-1.777353941	
	H295	4.118318000	2.828023659	-7.684750032	

	H296	5.647968446	6.974672508	-3.291893819
	H297	6.268971498	-6.419301490	4.064744820
	H298	7.627087950	-7.520437938	1.548010971
	H299	2.418953936	1.355775031	-7.355689430
	H300	5.201603873	0.403659349	2.223799116
	H301	-0.944936991	1.406853203	-8.575389426
	H302	-1.967380071	-2.578496636	-3.901343060
	H303	-0.753305417	2.005955985	-6.925711041
	H304	5.187488299	-3.989547292	3.638532365
	H305	1.027567869	0.029670193	-6.765678436
	H306	4.698394053	-4.561959389	2.044213364
	H307	5.515959706	-6.756776327	2.491293927
	H308	7.099764604	-4.996381884	1.459953475
	H309	7.576862326	-4.547486739	3.109911337
	H310	-6.124123634	-0.973426952	-5.456503873
	H311	6.971829085	-8.850793284	4.154990749
	H312	5.698375224	-11.352153293	2.952666737
	H313	-0.457385107	-0.588015942	-3.862769820
	H314	7.627833531	-11.097302475	5.129821056
	H315	6.035937768	-11.483048957	5.183407951
	H316	5.932189032	-2.718227123	1.025288829
	H317	-1.285950077	1.599628443	-4.622005148
	H318	-4.294041678	-2.354747862	-4.742319694
	H319	7.430441331	-9.721673658	-2.510681729
	H320	5.960923107	-7.506659027	-4.581995037
	H321	6.526960862	-8.027596060	-0.968031984
	H322	6.504473949	-10.452677021	-3.504760234
	H323	3.159335743	2.350399010	-10.306071160
	H324	6.340413540	-4.904947315	-1.335427782
	H325	2.705200736	-7.195982673	0.692351279
	H326	2.071876807	-2.058391378	-7.949388447
	H327	2.853658066	-8.941116556	-1.135110260
	H328	1.946077249	-6.866336187	-7.344516822
	H329	1.402110540	-5.326392533	-5.398129562
	H330	7.361185790	-11.950972253	2.955216518
	H331	5.336897393	-0.587950516	0.786834673
	H332	8.521166743	-5.612806633	-0.648612274
	H333	7.127023647	-3.571436932	-1.421022353
	H334	9.545535894	-6.506069638	1.328634155
	H335	5.625861224	-2.164339465	4.553961621
	H336	2.224154808	-11.556952307	-3.908820604
	H337	2.871684938	-8.055233773	-4.066824177
	H338	4.359539393	-8.294434848	-3.132072179
	H339	1.455058525	-8.427126476	-2.119894686
	H340	0.456107204	-2.173108695	-6.174455202
	H341	-3.626370560	1.835387509	-5.514045036
	H342	-5.979777633	0.796859934	-5.594203290
	H343	2.186329837	-5.550161712	0.198890644
	H344	8.574494730	-7.164508686	-0.707891043
	H345	7.439770648	-9.050825358	-0.233006492
	H346	4.865171200	-0.633440000	4.340707730
	H347	4.422196169	-7.565104861	-7.356473899
	H348	5.937171367	-5.837694444	-4.025749802
	H349	-0.658458256	-3.028985003	-7.281778573
	H350	2.944895164	4.148822050	-7.868522582
	H351	8.024607451	0.180563022	2.746144175
	H352	7.937541385	0.608100847	1.020249122
	H353	7.845493186	-6.742463368	-7.644686690
	H354	7.273959401	-7.986748369	-6.750152782

	H355	10.088151004	-8.433980728	-3.347312655
	H356	8.055163936	-8.105669992	-4.327119956
	H357	10.997134706	-7.238317926	-5.646285934
	H358	9.959630293	-6.751428985	-2.786133137
	H359	10.689038010	0.146593651	0.728235792
	H360	-2.560208263	13.347110423	3.827216539
	H361	11.227054955	-6.349071438	3.822338624
	H362	10.463016916	-4.000643193	2.611022967
	H363	-4.735420246	12.329779875	2.031574657
	H364	-3.307614323	10.255359804	3.808432536
	H365	-4.995044001	10.766362753	3.973710752
	H366	-6.673055011	6.945169677	-0.975959048
	H367	4.318748811	-4.128008580	-0.025439771
	H368	0.725156103	-1.103818024	0.201945784
	H369	-1.643792413	-0.007837151	-0.548474643
	Mo370	3.363762616	-4.214194369	-2.941966165
	N371	-6.650665219	-7.223780217	0.832368987
	N372	-8.383585308	-4.618180137	2.884178998
	N373	-7.780614205	-4.852238118	5.643696681
	N374	-5.233849451	-6.027167374	5.619662652
	N375	-3.690236648	-3.959407423	4.379673261
	N376	-3.987331493	-1.947881253	6.380185916
	N377	-2.960437025	-3.175803903	8.789999812
	N378	-0.451807153	-4.407819045	8.130959681
	N379	1.031424562	-2.793802948	6.396575388
	N380	0.691816461	0.147269789	6.708159873
	N381	0.424392112	0.259978310	9.603482567
	N382	6.715105079	-10.871650340	4.727893709
	N383	7.169915851	-8.741270032	3.154643311
	N384	5.749784909	-2.754841531	2.032852552
	N385	5.165279942	-1.511813653	3.927292993
	N386	5.142715233	-0.527592810	1.816502532
	N387	9.798086583	-6.547362527	2.339582162
	N388	-6.120152908	-3.151949453	-1.861741879
	N389	-2.147913177	-5.642895471	-2.666375532
	N390	-6.345024360	-0.635710390	-1.146145920
	N391	-8.384650819	0.221237201	0.523160549
	N392	-7.239096834	0.329190166	3.044915027
	N393	-5.169163461	2.205362786	3.104762032
	N394	-3.328064810	2.650001932	-0.728366997
	N395	-2.305401537	0.780779682	-0.362191836
	N396	-5.721615276	4.824526958	3.843819893
	N397	-9.273734444	7.813493232	3.026812484
	N398	-9.005479632	9.632769369	4.269227804
	N399	9.840886615	2.552805033	1.108433840
	N400	6.205523350	7.634349364	-1.387154159
	N401	3.737474807	8.514173939	-3.791548017
	N402	1.107997383	7.563875859	-3.800516981
	N403	-3.634244650	6.067197000	-4.154145638
	N404	-3.378840385	8.022521974	-5.417892279
	N405	-4.536893676	8.069632346	-3.411384898
	N406	0.541322117	6.598609786	-1.175996075
	N407	1.058973957	8.924640078	0.324625366
	N408	-0.507036788	11.289320608	0.178935102
	N409	-2.024285511	10.011213991	-2.182027015
	N410	-2.719890590	11.876971592	1.721184021
	N411	2.357292499	-4.820485070	-10.726174506
	N412	1.222722320	-2.624594949	-8.067469977
	N413	0.189018970	0.169791876	-7.361683516

	N414	2.158974287	2.180242353	-7.913441610
	N415	-3.225534417	-3.177361400	-9.586580548
	N416	-4.610977544	-1.351752883	-7.880895015
	N417	7.072184513	-7.023152237	-7.034733824
	N418	3.485328583	-5.545875480	-4.896959278
	N419	2.634102105	-6.552377591	-6.665895272
	N420	8.701842099	-7.320250770	-4.413821727
	N421	0.108988620	2.626295741	-0.298173221
	N422	0.739449087	1.913135549	-0.909179927
	O423	-6.179296349	-5.174926746	2.780025445
	O424	-6.717571948	-2.838457068	5.532936689
	O425	-4.892705772	-5.273150388	7.751282611
	O426	-1.937182840	-4.713075862	5.615476331
	O427	-1.937732262	-1.180690647	5.758019376
	O428	-2.188368780	-1.092388228	9.314971261
	O429	0.155582337	-4.199739177	10.335500934
	O430	2.829023342	-4.221144130	6.508155805
	O431	2.963205782	0.028247564	6.470729598
	O432	1.987100582	1.900297257	9.459223122
	O433	2.646587827	-0.007532571	12.408850107
	O434	7.415270259	-10.029768454	1.247359110
	O435	9.101543057	-7.096360592	4.462229840
	O436	12.485587186	-4.166718947	2.423864050
	O437	-5.463042805	-0.944222522	0.949292687
	O438	-4.136383599	-4.678094433	-3.201028123
	O439	-7.699176561	2.393008989	0.359347455
	O440	-6.871918387	2.916229104	-2.184438023
	O441	-9.198030928	1.121655013	3.951884264
	O442	-4.553345718	1.553057109	5.207380612
	O443	-3.492579434	5.283904100	4.055582618
	O444	-5.487717129	7.480526994	6.174157365
	O445	10.924971065	-0.434782663	2.671199310
	O446	4.262019417	5.071059449	3.131934626
	O447	3.457954334	8.716084807	-1.538859305
	O448	0.296031860	9.711086351	-3.888878636
	O449	-1.666840441	7.000508867	-1.631713346
	O450	-0.260767916	8.121305817	2.016283216
	O451	2.572173922	6.269649229	1.085932313
	O452	1.053225564	12.444513812	1.398638047
	O453	-3.099578725	13.844339645	0.624323575
	O454	-3.902837628	11.190660574	-1.620340982
	O455	-4.510315227	13.453470200	4.422573279
	O456	-5.706977443	6.900882990	-0.837661057
	O457	0.347511234	-4.600165575	-8.850096029
	O458	-1.611752628	-0.902081070	-8.299454062
	O459	0.537936781	3.511959777	-8.801734478
	O460	4.194305373	4.105713254	-10.195991980
	O461	-6.309089192	-2.877067505	-7.865332792
	O462	-2.258860335	-6.747158259	-5.576389963
	O463	-0.570049196	-5.807899251	-6.688213433
	O464	-5.254059488	2.129674821	-7.632404113
	O465	9.178176898	-5.217776067	-5.108488796
	O466	12.222534311	-6.683158385	-4.111103763
	O467	-0.271612898	-6.284265021	-0.319110377
	O468	0.374435651	-8.270060300	0.446847197
	O469	4.351842251	-10.734074913	-3.769697073
	O470	2.098340808	-10.613413762	-3.670644980
	O471	4.808410207	-5.653313533	-1.991792283
	O472	4.942804382	-7.398389616	-0.570977193

	O473 2.220783232 -5.888191483 -2.360026378<br>O474 9.113032740 -6.376838212 -0.430177817<br>O475 7.112545884 -4.423205232 -0.940481793<br>O476 -4.796929296 4.687119925 -1.927599501<br>O477 7.452162943 -10.289326150 -3.322377027<br>O478 7.399599468 -8.503984798 -1.060491879<br>S479 3.256701003 5.157326195 -2.881705037<br>S480 1.475804317 2.030009513 -3.769693223<br>S481 1.436202828 -3.110570693 -3.851953125<br>S482 3.571720371 1.893146597 -0.715780514<br>S483 0.315278085 -0.996536491 -1.098485474<br>S484 3.205752422 -0.608883100 -6.195436181<br>S485 3.442490721 -3.252015527 -0.677387334<br>S486 5.153746557 1.893200098 -3.915723517<br>S487 5.162729649 -3.041898583 -3.988919457<br>S488 6.194362448 -0.758764526 -1.221223051

TS 7 → 8 35 (S=1/2)

TS 7 → 8 35 (S=1/2)<br>Fe(139) -2.249<br>Fe(140) -1.508<br>Fe(141) -2.612<br>Fe(142) 2.861<br>Fe(143) 2.964<br>Fe(144) 2.040<br>Fe(145) -1.020	bm22b2n2x3b4to3tc35.car_1<br>C1 -7.587885324 -5.267017368 1.812054094<br>C2 -8.001726222 -6.333590290 0.792195644<br>C3 -8.342606032 -3.402136627 3.225720545<br>C4 -7.715591182 -3.811933031 4.556970946<br>C5 -7.480797069 -5.523247925 6.283692460<br>C6 -5.984564915 -5.839606427 6.177695788<br>C7 -4.140694693 -6.484570277 4.695380560<br>C8 -3.339753100 -5.182398403 4.607456935<br>C9 -3.277996957 -2.854575438 3.889426723<br>C10 -3.136468819 -2.199225839 5.270703986<br>C11 -4.136646907 -1.730836647 7.431646714<br>C12 -3.182727980 -2.449196047 8.395247970<br>C13 -2.496424555 -4.578773652 9.382837285<br>C14 -1.044531528 -4.918070554 9.021662214<br>C15 0.512643486 -5.560024168 7.225476191<br>C16 1.380929162 -4.566715549 6.449337704<br>C17 1.487656420 -2.334356354 5.383258619<br>C18 1.690652945 -1.080791070 6.255247749<br>C19 0.802755667 -2.036704228 4.021729137<br>C20 1.454644958 -0.847498128 3.306421803<br>C21 0.810946704 -3.284314046 3.132648225<br>C22 0.627941448 0.727941396 7.549453318<br>C23 0.969275886 0.379785709 9.010765428<br>C24 -0.672355722 1.558386496 7.424720531<br>C25 -0.682330399 2.736126549 8.406734302<br>C26 -0.858377214 2.061320568 5.987307973<br>C27 0.469155200 -0.932785251 10.988890606<br>C28 1.915558573 -1.285418810 11.274342717<br>C29 6.238126995 -11.618566282 2.382805863<br>C30 6.710271444 -10.345856588 1.682421462<br>C31 7.176833069 -7.923032174 2.091195935<br>C32 8.522570715 -7.603664173 2.768837324<br>C33 6.089619752 -6.906038442 2.481074075<br>C34 6.452549587 -5.459810580 2.137418159<br>C35 5.225326520 -4.555378758 2.221059870<br>C36 5.221610975 -2.063850246 2.454642079<br>C37 10.807048511 -6.791174313 2.414978791<br>C38 11.114973558 -5.309114310 2.402858016<br>C39 -5.465924807 -2.517172151 -0.969945965

	C40	-5.762911733	-1.116065601	-0.408616745
	C41	-3.935834140	-2.661348847	-1.124132013
	C42	-3.415016710	-4.092817704	-1.367840930
	C43	-3.346813422	-4.524150805	-2.832312432
	C44	-6.468106108	1.145985348	-1.044116699
	C45	-7.555587250	1.499048187	-0.025607751
	C46	-6.674104567	1.965708423	-2.335462468
	C47	-9.280011255	0.706452349	1.510297612
	C48	-8.590629073	0.758487514	2.881738222
	C49	-6.540057761	0.329830794	4.171860375
	C50	-5.338037726	1.267656208	4.253069602
	C51	-4.029274693	3.064194015	3.201814904
	C52	-4.313241372	4.336523883	4.035165168
	C53	-3.692625080	3.560018325	1.788916685
	C54	-3.175717212	2.545650581	0.821840801
	C55	-2.575457873	1.317431372	0.952591577
	C56	-2.619249112	1.816335607	-1.211446553
	C57	-6.048823215	5.980923226	4.633251952
	C58	-5.706138527	6.060838055	6.119078919
	C59	-7.577717724	6.096463777	4.415293656
	C60	-8.152500364	7.481785246	4.451640607
	C61	-7.961767535	8.515474810	5.349189651
	C62	-9.404564677	9.112779046	3.776841972
	C63	9.586943776	0.953752087	2.415091692
	C64	10.441963616	-0.246346312	2.052049921
	C65	8.107418387	0.506158830	2.271106677
	C66	7.123325414	1.583977456	2.659512393
	C67	6.698620215	1.737537169	3.992928074
	C68	6.602333302	2.468161373	1.698009303
	C69	5.783954363	2.733248165	4.355572702
	C70	5.698163720	3.473332954	2.046522115
	C71	5.287548442	3.603447214	3.376797465
	C72	5.479116702	7.130572863	-1.573397036
	C73	4.401588699	8.214241437	-1.693867253
	C74	4.840180671	5.788032953	-1.182350902
	C75	3.112269391	9.650268641	-3.210742814
	C76	1.667960379	9.163205873	-3.086438820
	C77	0.162721776	7.214509344	-2.911308625
	C78	-0.189559432	7.004287304	-1.422356209
	C79	0.093548651	5.914968083	-3.736711173
	C80	-1.222601713	5.130376293	-3.716032826
	C81	-2.411837304	5.733334348	-4.486279751
	C82	-3.502234277	7.866324765	-3.745575455
	C83	0.555420199	6.480112713	0.843525045
	C84	0.614140175	7.794288706	1.650129788
	C85	1.492538414	5.460872219	1.504359720
	C86	1.836670139	9.888770543	1.992640733
	C87	1.086445869	11.218601133	1.915155810
	C88	-0.775271648	12.485445978	0.900003031
	C89	-1.951102274	12.771192962	1.859238747
	C90	-1.253757142	12.547300969	-0.570648624
	C91	-2.208153279	11.403138093	-0.890721893
	C92	-3.441546462	11.859105116	3.592117305
	C93	-3.182433050	12.792622158	4.770356054
	C94	-3.833212830	10.445532717	4.102153573
	C95	-4.266887163	9.525171409	2.984422750
	C96	-5.590829948	9.544118348	2.517338046
	C97	-3.355192600	8.659544234	2.357361152
	C98	-5.988859522	8.750398535	1.439047823

	C99	-3.729570722	7.873180483	1.263595062
	C100	-5.046518941	7.932647041	0.813543838
	C101	2.633018735	-3.238520331	-9.810137974
	C102	1.323467796	-3.024227370	-9.067653543
	C103	0.157533171	-1.617930323	-7.387206862
	C104	-0.413893632	-0.237859988	-7.730732267
	C105	-0.022235169	2.177393043	-7.585239917
	C106	1.099329952	3.163357855	-7.901943006
	C107	3.459387979	3.760930713	-7.825686844
	C108	3.899092699	3.892077141	-9.271162799
	C109	-4.091386605	-2.760783068	-8.875765486
	C110	-5.049306421	-1.694572750	-8.328958347
	C111	-3.454596339	-3.536097038	-7.697860947
	C112	-2.827874525	-4.860239756	-8.174137639
	C113	-1.956637877	-5.457936358	-7.093066227
	C114	-5.309978113	0.573166801	-7.440167264
	C115	-4.919301016	1.907476490	-8.047520795
	C116	-5.371633843	0.563350097	-5.891188792
	C117	-4.019145640	0.376533109	-5.249259931
	C118	-3.133320996	1.455228891	-5.073190428
	C119	-3.584312652	-0.911367317	-4.890899220
	C120	-1.840830819	1.239563110	-4.581277698
	C121	-2.291076709	-1.127149217	-4.402379096
	C122	-1.408934085	-0.052286103	-4.258258354
	C123	6.946176256	-5.861391744	-6.213667885
	C124	8.266453377	-6.020419735	-5.440566722
	C125	5.735018983	-6.244129905	-5.349604640
	C126	4.388436557	-5.952822758	-5.942137401
	C127	3.872088909	-6.431196139	-7.129567709
	C128	2.295138692	-5.325940403	-6.034117248
	C129	9.765092607	-7.423977720	-4.135599326
	C130	10.973851013	-7.040224486	-4.959798571
	C131	0.392801523	-7.117160107	-0.594179021
	C132	1.853403090	-6.758348718	-0.569583392
	C133	2.525204751	-6.764932844	-2.004858623
	C134	2.269105601	-8.159110531	-2.650884441
	C135	3.075016305	-8.396707242	-3.937378336
	C136	3.068096632	-9.823705343	-4.410654078
	C137	4.065909030	-6.647082846	-1.775934055
	C138	3.269880211	-0.477870026	-2.822662225
	Fe139	3.278740912	-1.628058545	-4.457027202
	Fe140	1.903046218	0.840823462	-1.727136453
	Fe141	4.774033498	0.618197212	-2.053411405
	Fe142	3.346921953	0.944570705	-4.204486210
	Fe143	3.543907403	2.925100308	-2.398665961
	Fe144	4.604326382	-1.898962692	-2.267706105
	Fe145	2.044007974	-1.831870839	-2.223575888
	H146	-6.214637829	-7.312146413	0.724167784
	H147	-6.446909015	-6.298313399	-0.534146447
	H148	-9.530148859	-4.599764177	1.913054912
	H149	-8.712600672	-5.889510906	0.078474411
	H150	-8.570202608	-7.098387503	1.350054802
	H151	-7.657422688	-2.669956942	2.775907657
	H152	-8.727166110	-5.597912977	4.531277783
	H153	-9.297950416	-2.900233708	3.428845626
	H154	-8.014807787	-6.436625751	6.576917269
	H155	-6.188009036	-6.218176063	4.171261777
	H156	-7.595210187	-4.785462666	7.088605988
	H157	-2.265850672	-2.942176390	3.474281449

	H158	-4.806904486	-4.303684358	3.441684221
	H159	-4.046050482	-7.035898993	3.750041514
	H160	-3.803657435	-0.687702750	7.361801223
	H161	-5.062586868	-2.759185627	5.783671933
	H162	-3.686151746	-7.084384938	5.492970191
	H163	-3.869284851	-2.203475504	3.230357421
	H164	-3.950374626	-4.291435446	7.842715081
	H165	-5.147602336	-1.745811639	7.861576208
	H166	0.322411612	-4.138696825	3.623551341
	H167	0.282155172	-3.089644389	2.186718354
	H168	1.841981233	-3.577966961	2.878483678
	H169	-0.244766330	-1.771546737	4.238646333
	H170	-3.023484560	-5.529749747	9.546457760
	H171	-0.893524775	1.236399100	5.262057565
	H172	-0.568400960	-1.026924934	9.131948672
	H173	-0.652444811	2.408787068	9.455931123
	H174	0.893057432	-0.604763788	2.391694009
	H175	2.487494235	-1.072860047	3.004484569
	H176	9.767647572	1.181039942	3.483717355
	H177	-1.800260565	2.622026377	5.906142763
	H178	-1.597577115	3.329156892	8.263639464
	H179	-0.032489193	2.734864148	5.705153450
	H180	0.180371920	3.397989648	8.232968996
	H181	-1.519571354	0.893115485	7.673726489
	H182	1.476713659	1.337067785	7.204093212
	H183	-0.356391401	-0.806271724	6.411469112
	H184	1.469293912	0.058569359	3.930597030
	H185	1.528777484	4.557228754	0.873888349
	H186	1.046026595	5.194196216	2.476673778
	H187	3.278192641	5.481083653	2.321067649
	H188	4.138606888	4.565410909	4.610168540
	H189	5.280970942	4.132118483	1.284761576
	H190	6.876549850	2.343756282	0.647431024
	H191	7.078525906	1.062207418	4.764132383
	H192	-0.176563403	-3.243801378	6.387183852
	H193	0.363296934	-6.430774275	6.569249318
	H194	1.098187384	-5.889599538	8.093888614
	H195	-1.541010919	-5.061949412	7.015476732
	H196	-2.460680600	-4.038813026	10.337910273
	H197	5.452053012	2.827616197	5.392862517
	H198	2.498934591	-2.719917101	5.197465916
	H199	0.163369255	-0.117501570	11.664889350
	H200	-0.138816870	-1.818352857	11.231855968
	H201	2.490008354	-1.677114326	10.402513628
	H202	11.576332701	-7.322423423	1.833829480
	H203	-2.235342868	-5.647304626	-4.133524591
	H204	0.892193549	-8.899243788	-0.054246341
	H205	-3.628893484	-5.575913684	-8.408759546
	H206	-2.233464997	-4.691504762	-9.084155763
	H207	-4.212304408	-3.756160151	-6.930542699
	H208	-4.547728218	1.838613487	-9.096909001
	H209	-4.754776247	-3.459271507	-9.408854142
	H210	-3.600300841	-1.997785188	-1.936967197
	H211	-4.056198619	-4.824835658	-0.846385305
	H212	-3.487663737	-0.349231851	-8.100078963
	H213	-6.338388928	0.394675769	-7.812671518
	H214	-2.406720724	-1.673590423	-9.413428972
	H215	-1.593859857	-5.586793922	-2.490252885
	H216	-2.699045506	-2.894115633	-7.218841494

	H217	-2.719760186	-2.963447190	-10.387325484
	H218	-7.050278769	-3.226384928	-2.089844817
	H219	-3.479737846	-2.291662404	-0.193912403
	H220	-0.396302878	-4.632199102	-7.972998658
	H221	-2.417922781	-4.196259007	-0.916017268
	H222	-3.156993735	2.584626916	3.668095910
	H223	-2.342442724	0.726605704	1.830175329
	H224	-7.229463175	0.613732496	4.979525638
	H225	-6.831624278	-0.031377228	2.088493395
	H226	-6.323399012	4.046731499	3.754566131
	H227	-7.331200165	8.618833023	6.224859803
	H228	-5.790436293	5.082664555	6.653686116
	H229	-8.093178472	5.437195469	5.135864906
	H230	-5.525338758	6.795133386	4.109062991
	H231	-10.107548687	9.734277298	3.228176982
	H232	-2.937106534	4.354560130	1.901716967
	H233	-4.586246983	4.040782373	1.357592297
	H234	-8.850313784	10.461311075	5.327919703
	H235	-7.794579998	5.694201497	3.413438046
	H236	-5.760926102	2.061743991	2.394723131
	H237	-6.187086164	-0.687061432	4.395698209
	H238	-9.833060426	1.647859530	1.396734707
	H239	-2.464739283	1.755321982	-2.285719293
	H240	-10.010538750	-0.112609249	1.520004082
	H241	10.777595840	2.490329864	1.766095676
	H242	9.179649365	2.790380757	1.612079825
	H243	1.707391099	6.295182312	-0.983712039
	H244	-0.472985734	6.108477864	0.953152393
	H245	2.003635152	8.566386107	0.353858922
	H246	-3.612749465	3.691738110	-0.957680060
	H247	-5.356783156	4.483315818	-1.859877252
	H248	-4.883035668	5.696380563	-0.940214114
	H249	-5.837964764	1.777512168	-3.022927399
	H250	-7.612330275	1.658581893	-2.827466236
	H251	-0.368269553	12.525216721	-1.222708992
	H252	-1.779355942	13.499153954	-0.715341247
	H253	-0.693068042	10.271258880	-1.815911779
	H254	-2.185667508	9.443618595	-1.487273859
	H255	-3.196717368	9.589329617	-4.808771222
	H256	-3.029091611	4.924865082	-4.904911248
	H257	-7.027709279	8.763091499	1.100442357
	H258	-2.325311808	8.582067242	2.716013883
	H259	-4.523877995	8.004492860	-1.952257981
	H260	0.885389205	5.232956757	-3.389519738
	H261	2.292568430	7.196731368	-3.077545968
	H262	-1.528009629	4.912189760	-2.680622285
	H263	-8.271376118	-0.404612720	-0.031894285
	H264	3.249276475	10.047049448	-4.225899502
	H265	-6.489774261	-0.715540805	-2.230499433
	H266	0.342148025	6.178934910	-4.778304269
	H267	-2.991205610	7.253752715	0.749673353
	H268	-7.166477523	3.463754828	-1.181507921
	H269	3.220535685	10.485835623	-2.504742631
	H270	-2.738652397	8.146429140	-5.635792943
	H271	-4.547612198	9.437076163	-2.933045079
	H272	-5.580879450	-3.271120782	-2.881097769
	H273	-5.787141424	-3.219009434	-0.177303493
	H274	-6.331113042	10.185557821	3.002610977
	H275	-5.538277009	1.497838591	-0.567457635

	H276	-0.579629299	7.937702140	-3.275333359
	H277	-2.051397194	6.318188677	-5.342068369
	H278	-0.996984307	4.157145778	-4.174665841
	H279	-3.771289621	6.050120616	-2.882117441
	H280	1.796696202	9.571332372	3.045558409
	H281	-0.273850451	10.418079116	0.603799024
	H282	-0.065197394	13.309611618	1.065429194
	H283	2.885438118	10.107406139	1.749795036
	H284	-1.734633963	10.919313760	2.688164768
	H285	3.115939002	-2.268085672	-9.996340354
	H286	3.287586891	-3.789064789	-9.102820459
	H287	6.882509232	-4.790373109	-6.453154542
	H288	3.293078485	-4.285086144	-11.428819620
	H289	1.813610449	-4.749351255	-10.897922239
	H290	5.631239211	5.027307117	-1.174835044
	H291	6.018236265	7.728064287	0.299251887
	H292	4.615796365	8.223965197	-3.738340455
	H293	4.409223146	5.880084636	-0.174922814
	H294	6.991155004	8.326345383	-0.869065208
	H295	4.325533583	3.422728444	-7.235437585
	H296	5.983164189	7.010384522	-2.545140570
	H297	5.882448935	-7.002648304	3.559635267
	H298	7.301213325	-7.919360764	1.001559621
	H299	2.611704228	1.915625582	-7.180505308
	H300	5.190054315	-0.033560465	2.296521722
	H301	-0.713506875	2.184446833	-8.437176424
	H302	-1.962530872	-2.138582538	-4.155188477
	H303	-0.573593037	2.602058886	-6.729276274
	H304	4.808633456	-4.530619928	3.236154621
	H305	1.168911433	0.573961325	-6.726060256
	H306	4.430702759	-4.963932513	1.572363597
	H307	5.174400479	-7.199871837	1.941803933
	H308	6.845670182	-5.399070271	1.111755845
	H309	7.236425664	-5.082614489	2.813149928
	H310	-6.040843656	-0.256125368	-5.591849172
	H311	6.546227979	-9.456385802	3.463890254
	H312	5.216675221	-11.801883339	2.015377072
	H313	-0.386605045	-0.208771938	-3.907004594
	H314	7.106997225	-11.801420582	4.239775114
	H315	5.503022797	-12.131126846	4.234966830
	H316	5.686401824	-3.069010856	0.773318612
	H317	-1.137478149	2.066405678	-4.454779466
	H318	-4.262476680	-1.760611250	-5.002202073
	H319	7.128161277	-9.746504533	-3.235792289
	H320	5.786887128	-7.326191220	-5.132240444
	H321	6.244050941	-8.167071812	-1.562755486
	H322	6.200488836	-10.342564946	-4.313138088
	H323	3.449272277	3.147836496	-9.971850284
	H324	6.174504491	-5.004320840	-1.623807208
	H325	2.419999082	-7.435572977	0.085534370
	H326	2.146749838	-1.428001244	-8.067616539
	H327	2.540684239	-8.931769917	-1.914540163
	H328	1.878073468	-6.234405152	-7.894966177
	H329	1.326608914	-4.902414109	-5.800797307
	H330	6.863882062	-12.442228121	1.998073393
	H331	5.273066291	-0.873353087	0.768122482
	H332	8.316611573	-5.869009590	-0.980905104
	H333	7.049582620	-3.722695669	-1.618512906
	H334	9.256166172	-6.971045910	0.928457713

	H335	5.442643619	-2.863600355	4.333300316
	H336	1.913930938	-11.235902343	-4.914027669
	H337	2.671052666	-7.763572582	-4.740698225
	H338	4.126898896	-8.126066769	-3.791891075
	H339	1.194995194	-8.250175134	-2.867986033
	H340	0.485278018	-1.651385272	-6.335962226
	H341	-3.455386854	2.462600914	-5.348237848
	H342	-5.836337851	1.512301815	-5.581623237
	H343	1.934036920	-5.734408637	-0.185851239
	H344	8.319387894	-7.407968935	-1.189384159
	H345	7.103310975	-9.283280883	-0.909204725
	H346	4.840770402	-1.251425646	4.305075126
	H347	4.332763580	-7.014783919	-7.917959974
	H348	5.807528065	-5.732692396	-4.381925989
	H349	-0.629772971	-2.358233719	-7.544013874
	H350	3.173398926	4.756655984	-7.453923594
	H351	7.968967574	-0.378507324	2.911090904
	H352	7.932031510	0.201534227	1.226442405
	H353	7.751901999	-6.266438626	-8.056726124
	H354	7.148917110	-7.593628178	-7.315945942
	H355	9.855009124	-8.498973258	-3.912321199
	H356	7.839658448	-7.988374718	-4.851326997
	H357	10.833112413	-7.104951758	-6.065218533
	H358	9.780871532	-6.882810585	-3.175426691
	H359	10.672517061	-0.334219103	0.962840349
	H360	-2.113231092	13.064240902	4.937352823
	H361	10.873785732	-7.129793987	3.463393860
	H362	10.227007063	-4.634796089	2.474856175
	H363	-4.286381807	12.281693482	3.024504431
	H364	-2.979480289	10.013087206	4.646589267
	H365	-4.648246835	10.579384625	4.828084188
	H366	-6.354698036	7.269221629	-0.467978928
	H367	3.299858498	-4.291885139	-0.091796311
	H368	0.632315330	-1.110667017	0.126772470
	H369	-1.622214668	0.093062398	-0.569334743
	Mo370	3.252839479	-4.065395760	-3.233961649
	N371	-6.922079985	-6.977223833	0.065266280
	N372	-8.600141345	-4.507340138	2.313960046
	N373	-8.064830852	-5.025329487	5.048814600
	N374	-5.554490025	-6.277932674	4.967550076
	N375	-3.926946798	-4.157021713	3.938438392
	N376	-4.193361973	-2.327643130	6.107374128
	N377	-3.249219744	-3.805520749	8.408104916
	N378	-0.774566439	-5.076943072	7.694553465
	N379	0.799439244	-3.381114222	6.130480946
	N380	0.561440375	-0.466749630	6.707265006
	N381	0.243859108	-0.610454687	9.591859627
	N382	6.210823485	-11.506947533	3.845626733
	N383	6.763329719	-9.263378985	2.480024387
	N384	5.502965067	-3.186738599	1.774229651
	N385	5.032008068	-2.108079352	3.793951198
	N386	5.066819902	-0.910278394	1.793582276
	N387	9.473946923	-7.108884488	1.938542318
	N388	-6.176468769	-2.717829521	-2.237873429
	N389	-2.312830576	-5.320346903	-3.165224565
	N390	-6.331021829	-0.270851841	-1.311202709
	N391	-8.381933770	0.517347646	0.384374527
	N392	-7.288339918	0.348751669	2.926825172
	N393	-5.135014623	2.114406940	3.196267561

N394	-3.193236069	2.823239849	-0.538178275
N395	-2.254779696	0.887808211	-0.320466408
N396	-5.620768475	4.690009677	4.109658769
N397	-9.056593489	7.867954282	3.475548688
N398	-8.763462947	9.543315263	4.899785934
N399	9.887822425	2.059223913	1.503128885
N400	6.498423894	7.479103446	-0.571829092
N401	4.133909707	8.655852167	-2.954648728
N402	1.474420699	7.826440175	-3.101300678
N403	-3.302708197	6.552621565	-3.656333340
N404	-3.002415425	8.594254121	-4.764871838
N405	-4.165121431	8.511558344	-2.763145147
N406	0.819367184	6.648596946	-0.584173333
N407	1.387002558	8.795691644	1.140201437
N408	-0.058536766	11.241611037	1.169872056
N409	-1.605532527	10.269394807	-1.343948284
N410	-2.282204752	11.775989528	2.717456139
N411	2.399698803	-3.929621363	-11.081057382
N412	1.284743673	-1.957277542	-8.246558402
N413	0.345727243	0.798949168	-7.319158350
N414	2.380591328	2.806960075	-7.639358925
N415	-3.161862440	-2.204204683	-9.865252984
N416	-4.500754097	-0.486564373	-8.019812377
N417	6.966128382	-6.600493316	-7.491079238
N418	3.383662076	-5.250604647	-5.273834057
N419	2.554480423	-6.026064903	-7.165748269
N420	8.521244549	-7.228531300	-4.863821057
N421	0.206622065	2.637143047	-0.088584133
N422	0.839268713	1.948700234	-0.726117657
O423	-6.413146387	-5.130770122	2.202793738
O424	-6.932976899	-3.049456675	5.147358171
O425	-5.240099847	-5.721718239	7.164593179
O426	-2.221979953	-5.074646231	5.134853564
O427	-2.105737508	-1.585206193	5.590504363
O428	-2.425480147	-1.807662333	9.142627014
O429	-0.211112270	-5.077209101	9.923008317
O430	2.536209028	-4.887220794	6.133889937
O431	2.831370856	-0.668017833	6.518229436
O432	1.858895087	0.983532896	9.632567478
O433	2.414123081	-1.212074139	12.387466738
O434	6.978740247	-10.379041138	0.462492141
O435	8.685524533	-7.797207351	3.986351927
O436	12.248535185	-4.862276715	2.333019013
O437	-5.506947645	-0.783765084	0.766189966
O438	-4.209980009	-4.175916931	-3.668625248
O439	-7.618239509	2.669602134	0.412468470
O440	-6.702997215	3.373908561	-2.052960360
O441	-9.229521610	1.148301588	3.865357861
O442	-4.604545570	1.254671153	5.244615209
O443	-3.391888438	5.019179830	4.493361045
O444	-5.399531700	7.084504868	6.704963528
O445	10.833909233	-1.077573640	2.858278717
O446	4.377141950	4.601211664	3.665568576
O447	3.809291229	8.675753354	-0.700378139
O448	0.750034209	10.006226144	-3.010892766
O449	-1.359836825	7.186540559	-1.037941742
O450	0.005188832	7.889381383	2.725393606
O451	2.790524547	6.034356051	1.676273482
O452	1.513523458	12.199660847	2.537195380

	O453 -2.539377341 13.861277012 1.827251840<br>O454 -3.423910519 11.470692141 -0.651970361<br>O455 -4.070771069 13.203375278 5.498861797<br>O456 -5.395453083 7.166811194 -0.314883622<br>O457 0.372792937 -3.836161108 -9.207772025<br>O458 -1.489037820 -0.105946495 -8.356662836<br>O459 0.794994488 4.278943403 -8.359590859<br>O460 4.702022588 4.728114761 -9.652686435<br>O461 -6.255259807 -1.940080935 -8.154865367<br>O462 -2.393751064 -6.119448546 -6.155450191<br>O463 -0.638021040 -5.189014368 -7.166752055<br>O464 -5.032244855 2.986033040 -7.484112471<br>O465 9.112607110 -5.108171985 -5.399519124<br>O466 12.059013407 -6.756332461 -4.471356592<br>O467 -0.522753337 -6.359088503 -0.884824792<br>O468 0.076457539 -8.413896387 -0.283357988<br>O469 4.064581265 -10.508700617 -4.627138196<br>O470 1.814102017 -10.311564700 -4.600644303<br>O471 4.628669041 -5.636729768 -2.350939001<br>O472 4.673882447 -7.522539318 -1.114037849<br>O473 2.040769346 -5.729335336 -2.775481561<br>O474 8.877805836 -6.670810334 -0.825974829<br>O475 6.966036409 -4.595531677 -1.186755143<br>O476 -4.563344201 5.021047124 -1.587751067<br>O477 7.149300583 -10.238284477 -4.095771545<br>O478 7.100799548 -8.664725844 -1.686198904<br>S479 3.490187220 5.253302147 -2.332995448<br>S480 1.610673766 2.266743165 -3.532075175<br>S481 1.406882141 -2.820786222 -4.076575918<br>S482 3.663346715 1.773153835 -0.492693143<br>S483 0.330100754 -0.898200606 -1.191160521<br>S484 3.289969832 -0.178984129 -6.162403141<br>S485 3.285642578 -3.169885587 -0.928501288<br>S486 5.282781370 2.076832779 -3.657986998<br>S487 5.128708617 -2.909331259 -4.157883655<br>S488 6.253467601 -0.836726385 -1.242683513

8 35 (S=1/2)

8 35 (S=1/2)	bm22b2n2x3b4to3tb35.car_3
Fe(139) -2.248<br>Fe(140) -1.688<br>Fe(141) -2.561<br>Fe(142) 2.940<br>Fe(143) 2.976<br>Fe(144) 1.774<br>Fe(145) -0.568	C1 -7.615343675 -5.194732651 1.895017111<br>C2 -8.031029010 -6.264333102 0.879463037<br>C3 -8.349784689 -3.287795616 3.257249505<br>C4 -7.748105669 -3.664746429 4.610323575<br>C5 -7.546473091 -5.334191311 6.382338029<br>C6 -6.053132858 -5.669681623 6.288609571<br>C7 -4.215418578 -6.371701990 4.821345843<br>C8 -3.389403480 -5.085753646 4.724489655<br>C9 -3.279111858 -2.772516411 3.964896633<br>C10 -3.150331951 -2.083535214 5.330220597<br>C11 -4.165767657 -1.534844516 7.462609349<br>C12 -3.232468945 -2.247353890 8.450106817<br>C13 -2.579445190 -4.370694295 9.473466872<br>C14 -1.122443596 -4.712794434 9.139528923<br>C15 0.459711264 -5.379216213 7.378207659<br>C16 1.335675113 -4.414571856 6.576116168<br>C17 1.457810102 -2.211297500 5.449702435<br>C18 1.658587319 -0.936140686 6.290643658<br>C19 0.783809707 -1.947936453 4.077074263

	C20	1.447918161	-0.782447015	3.336110168
	C21	0.790262129	-3.218876705	3.221182399
	C22	0.590554569	0.905344202	7.533824717
	C23	0.920639587	0.592968522	9.005723987
	C24	-0.707023266	1.736320299	7.379677801
	C25	-0.715742227	2.944143124	8.324642886
	C26	-0.886786167	2.190789105	5.925693367
	C27	0.402079345	-0.668313699	11.012709625
	C28	1.846991443	-0.995031720	11.332011855
	C29	6.156913482	-11.576872070	2.714037935
	C30	6.642704294	-10.329519477	1.976913965
	C31	7.153874095	-7.904609358	2.316920810
	C32	8.508351004	-7.585334744	2.977040098
	C33	6.081256037	-6.861298827	2.684966732
	C34	6.450319497	-5.425448294	2.298140739
	C35	5.220170015	-4.519318300	2.331508251
	C36	5.215834650	-2.022160326	2.492282418
	C37	10.790562920	-6.778341215	2.600019303
	C38	11.104942523	-5.297636863	2.558767619
	C39	-5.464702136	-2.531531890	-0.938326825
	C40	-5.761222276	-1.117453976	-0.408806115
	C41	-3.935068214	-2.679140891	-1.093806081
	C42	-3.412856771	-4.116333089	-1.300720247
	C43	-3.347067598	-4.583194318	-2.753342330
	C44	-6.447441679	1.132620155	-1.095297152
	C45	-7.517309469	1.515137124	-0.069785269
	C46	-6.669679878	1.925394553	-2.400416692
	C47	-9.276543203	0.781596215	1.453749454
	C48	-8.596561131	0.867019314	2.827450872
	C49	-6.550924566	0.467402736	4.137377438
	C50	-5.333718561	1.389034745	4.182266749
	C51	-4.032540578	3.165549849	3.088570146
	C52	-4.298405573	4.465672306	3.884860507
	C53	-3.687373743	3.608579596	1.660686423
	C54	-3.167127969	2.558598673	0.733739349
	C55	-2.562293138	1.337795789	0.908936389
	C56	-2.587572927	1.764099580	-1.270422211
	C57	-6.018862212	6.124747379	4.488503129
	C58	-5.645433139	6.228787844	5.965929808
	C59	-7.552441023	6.238953953	4.307834943
	C60	-8.115629435	7.629022199	4.297761451
	C61	-7.905096593	8.694405710	5.153469085
	C62	-9.344405461	9.251828704	3.562907989
	C63	9.599316987	0.960820256	2.429781382
	C64	10.447270149	-0.252649301	2.094097957
	C65	8.115919365	0.527533523	2.275777520
	C66	7.139195280	1.620471052	2.637503082
	C67	6.700548544	1.797668278	3.963650350
	C68	6.636817860	2.495503495	1.657913469
	C69	5.790272574	2.806657362	4.300165367
	C70	5.736467531	3.512440583	1.979912456
	C71	5.310598146	3.665732722	3.302792444
	C72	5.526316621	7.069447042	-1.736334690
	C73	4.456324793	8.157993260	-1.880002263
	C74	4.870997487	5.750474529	-1.300732540
	C75	3.166071275	9.560081464	-3.427728884
	C76	1.719480581	9.079973812	-3.300991762
	C77	0.205178724	7.143267758	-3.078672442
	C78	-0.159026207	6.971157778	-1.587251035

	C79	0.133283449	5.825664242	-3.873951044
	C80	-1.183368773	5.043376092	-3.836842282
	C81	-2.373643170	5.620484695	-4.626173343
	C82	-3.440068756	7.782689803	-3.935976723
	C83	0.571799868	6.506929525	0.698681246
	C84	0.643051567	7.846984169	1.461420862
	C85	1.495279408	5.501057167	1.397700836
	C86	1.876284127	9.943506884	1.741155817
	C87	1.120058955	11.267521862	1.637714760
	C88	-0.749089089	12.499458677	0.592633297
	C89	-1.935591844	12.810725642	1.530704976
	C90	-1.217163112	12.521486544	-0.883342286
	C91	-2.157769348	11.361143075	-1.182665775
	C92	-3.425347725	11.951277448	3.291171810
	C93	-3.164686813	12.906738785	4.452426591
	C94	-3.834011859	10.553238475	3.830048485
	C95	-4.258909550	9.606116318	2.731598710
	C96	-5.579779640	9.614254262	2.255088279
	C97	-3.343130462	8.727544138	2.130139142
	C98	-5.971116114	8.795091186	1.193061643
	C99	-3.710425292	7.916711259	1.051784355
	C100	-5.024394622	7.964502460	0.592034118
	C101	2.665212648	-3.491413627	-9.705382313
	C102	1.345963369	-3.240244633	-8.992721565
	C103	0.182803403	-1.793080480	-7.344029840
	C104	-0.372577196	-0.416057381	-7.724075570
	C105	0.048767975	1.998057643	-7.643178601
	C106	1.190485154	2.948763476	-7.997326114
	C107	3.554230160	3.516909894	-7.925632069
	C108	3.918201534	3.654151979	-9.390892655
	C109	-4.043775590	-2.938964164	-8.841049315
	C110	-5.000148524	-1.855848668	-8.328269311
	C111	-3.424197220	-3.695000628	-7.640754251
	C112	-2.814996634	-5.036670910	-8.090817988
	C113	-1.945788666	-5.628367084	-7.005674554
	C114	-5.253230126	0.434528935	-7.494818333
	C115	-4.844860024	1.751242645	-8.126926450
	C116	-5.324163709	0.456351593	-5.946750250
	C117	-3.977782667	0.268607361	-5.291264088
	C118	-3.080255828	1.339374502	-5.128122298
	C119	-3.563551171	-1.015589023	-4.899342714
	C120	-1.799634160	1.120870525	-4.607478358
	C121	-2.281443421	-1.235337163	-4.385166307
	C122	-1.389726025	-0.167986984	-4.246564293
	C123	6.929412982	-6.049404573	-6.043378468
	C124	8.246609826	-6.193186964	-5.259660683
	C125	5.714346673	-6.364795087	-5.155880258
	C126	4.367269891	-6.089013531	-5.756456392
	C127	3.848446831	-6.597202973	-6.930469744
	C128	2.276084846	-5.458231239	-5.864276666
	C129	9.738815542	-7.571127618	-3.921132323
	C130	10.954322962	-7.209836783	-4.745972985
	C131	0.365050537	-7.088606024	-0.398907218
	C132	1.835155487	-6.782627145	-0.369386645
	C133	2.493531803	-6.818848675	-1.812164458
	C134	2.230415339	-8.226776806	-2.425286703
	C135	3.034160246	-8.495890377	-3.705952448
	C136	3.029875898	-9.934743844	-4.141190985
	C137	4.033609964	-6.689659835	-1.589742798

	C138	3.285826922	-0.554240437	-2.788775360
	Fe139	3.239286452	-1.758622495	-4.403387894
	Fe140	1.974879149	0.808003077	-1.752080170
	Fe141	4.764372341	0.585738519	-2.017150496
	Fe142	3.375112281	0.829090544	-4.245573589
	Fe143	3.583439428	2.887548165	-2.466850639
	Fe144	4.610953405	-1.916833977	-2.244970274
	Fe145	2.085330937	-1.867021463	-2.169267123
	H146	-6.281823333	-7.313034748	0.898252743
	H147	-6.425883981	-6.315423495	-0.383665775
	H148	-9.550366188	-4.497334069	1.967447698
	H149	-8.690070853	-5.801480558	0.128168751
	H150	-8.655533065	-6.995008739	1.421667513
	H151	-7.636362047	-2.592883542	2.791705116
	H152	-8.782814831	-5.436307703	4.623977517
	H153	-9.293283625	-2.753214638	3.430617128
	H154	-8.092975134	-6.234643855	6.692719630
	H155	-6.258232660	-6.091669191	4.290697173
	H156	-7.656555034	-4.577072587	7.169250919
	H157	-2.261151405	-2.887562542	3.571190957
	H158	-4.836744657	-4.195504789	3.543122661
	H159	-4.132678419	-6.927622460	3.877731747
	H160	-3.810914028	-0.500906855	7.367815322
	H161	-5.095906518	-2.589330626	5.831925085
	H162	-3.772955771	-6.976913355	5.621800893
	H163	-3.845629686	-2.129391486	3.276606370
	H164	-4.011639500	-4.089884223	7.913353127
	H165	-5.179766177	-1.520898954	7.884623996
	H166	0.287228388	-4.054559477	3.729097065
	H167	0.273429421	-3.042266449	2.265496591
	H168	1.820629808	-3.529041655	2.986700489
	H169	-0.264070336	-1.672191382	4.277989081
	H170	-3.109437505	-5.321062384	9.630919138
	H171	-0.926689905	1.340254675	5.230583234
	H172	-0.609310413	-0.818473992	9.146558271
	H173	-0.697046625	2.645772234	9.382956720
	H174	0.903612361	-0.575121417	2.402701155
	H175	2.485105375	-1.018473388	3.058719498
	H176	9.771328568	1.204391404	3.495848687
	H177	-1.824070181	2.755350636	5.820954270
	H178	-1.625680469	3.539140888	8.157484895
	H179	-0.054126128	2.846100253	5.621725502
	H180	0.152871229	3.595111201	8.139859836
	H181	-1.558097257	1.084581252	7.650044110
	H182	1.442181776	1.505006395	7.179123438
	H183	-0.388849722	-0.659125868	6.432237918
	H184	1.451592090	0.145058921	3.927568974
	H185	1.510576569	4.567515990	0.811600417
	H186	1.050663100	5.292200319	2.385238344
	H187	3.290589579	5.518432349	2.192041686
	H188	4.155222730	4.653160265	4.510639975
	H189	5.336020962	4.164914462	1.204541247
	H190	6.921290603	2.354308902	0.612404188
	H191	7.063180982	1.130069687	4.749793442
	H192	-0.215947174	-3.087591034	6.468662549
	H193	0.315455120	-6.273369292	6.753538759
	H194	1.036073661	-5.675232931	8.263895348
	H195	-1.591167129	-4.889662425	7.131862938
	H196	-2.561144875	-3.826002378	10.426301948

	H197	5.447068320	2.918592595	5.332669450
	H198	2.468968723	-2.605630182	5.282057387
	H199	0.073006231	0.163045134	11.657852115
	H200	-0.199003700	-1.554236529	11.271487789
	H201	2.442827532	-1.391919368	10.477015842
	H202	11.557534146	-7.327413973	2.031846717
	H203	-2.232793726	-5.730733760	-4.031063391
	H204	0.797809700	-8.882581530	0.158478803
	H205	-3.627477075	-5.744864397	-8.307550912
	H206	-2.225118385	-4.892407713	-9.007963973
	H207	-4.188700438	-3.889563633	-6.873111245
	H208	-4.468525614	1.655242833	-9.172027833
	H209	-4.705382466	-3.645424267	-9.366494990
	H210	-3.604751027	-2.039288763	-1.927825396
	H211	-4.052038623	-4.836543018	-0.760792352
	H212	-3.432902483	-0.514204320	-8.118997927
	H213	-6.280693755	0.257475598	-7.870459438
	H214	-2.347321175	-1.872717348	-9.380922439
	H215	-1.576864168	-5.610554850	-2.396169150
	H216	-2.662939768	-3.052644645	-7.171372241
	H217	-2.652624693	-3.182901331	-10.328648940
	H218	-7.055444189	-3.258972812	-2.038400995
	H219	-3.474637120	-2.281481335	-0.177197123
	H220	-0.384029997	-4.821592510	-7.899487719
	H221	-2.415671896	-4.205952022	-0.846931210
	H222	-3.166236176	2.688959921	3.568885682
	H223	-2.333973560	0.776607082	1.806360388
	H224	-7.236276204	0.796018095	4.931352790
	H225	-6.842185474	0.037406166	2.068388450
	H226	-6.314542288	4.168704786	3.664926697
	H227	-7.266651083	8.824789824	6.020467820
	H228	-5.701570720	5.251909662	6.506373270
	H229	-8.050599804	5.614384145	5.069444195
	H230	-5.509399716	6.926165179	3.931874093
	H231	-10.037094275	9.865170825	2.992276173
	H232	-2.929306557	4.404145226	1.748196327
	H233	-4.576165840	4.077001842	1.205483044
	H234	-8.772511276	10.647774519	5.065786467
	H235	-7.803251058	5.797216698	3.331725231
	H236	-5.788744942	2.169897658	2.323964755
	H237	-6.216375515	-0.545910114	4.402741269
	H238	-9.823801949	1.722160237	1.310477820
	H239	-2.420782611	1.669986419	-2.340232848
	H240	-10.010951692	-0.033242599	1.478066977
	H241	10.816136285	2.469161067	1.761027935
	H242	9.222753855	2.789735476	1.592572240
	H243	1.729582396	6.259815206	-1.117669400
	H244	-0.461013736	6.150226651	0.819239054
	H245	2.038262698	8.572762142	0.142004949
	H246	-3.590705229	3.644227828	-1.083044542
	H247	-5.343467103	4.445774220	-1.987747486
	H248	-4.857615935	5.677704230	-1.100355015
	H249	-5.840033616	1.723348810	-3.092083501
	H250	-7.612905738	1.608135825	-2.876308458
	H251	-0.327975607	12.493863188	-1.530351400
	H252	-1.751853616	13.464750066	-1.048682688
	H253	-0.625815141	10.212846396	-2.056185738
	H254	-2.107190197	9.378504332	-1.701858096
	H255	-3.084342601	9.485210906	-5.014846290

	H256	-2.989408489	4.795330090	-5.013783922
	H257	-7.007599931	8.799883139	0.847631859
	H258	-2.316263320	8.656369264	2.498336476
	H259	-4.478849488	7.969122245	-2.154853979
	H260	0.922227843	5.149644942	-3.509567484
	H261	2.337338137	7.112096923	-3.222259224
	H262	-1.491420040	4.852728157	-2.796785453
	H263	-8.287087592	-0.366899186	-0.075602817
	H264	3.307053039	9.938933752	-4.448973192
	H265	-6.474857481	-0.752273138	-2.243973917
	H266	0.385729363	6.063973263	-4.920613788
	H267	-2.968731861	7.285692040	0.557536452
	H268	-7.140316809	3.448156553	-1.267370552
	H269	3.273349606	10.407175097	-2.735744274
	H270	-2.640681407	8.025852309	-5.815624755
	H271	-4.437085404	9.398427291	-3.146589756
	H272	-5.586098459	-3.338553952	-2.826356038
	H273	-5.784274015	-3.213792058	-0.128131613
	H274	-6.323478374	10.265066270	2.721958376
	H275	-5.508790785	1.487607287	-0.639734761
	H276	-0.528477705	7.862617943	-3.466597159
	H277	-2.016104902	6.172461723	-5.504397329
	H278	-0.956695372	4.058137576	-4.269393945
	H279	-3.754680205	5.982687588	-3.051342047
	H280	1.852215645	9.650533169	2.801667751
	H281	-0.246100540	10.427133619	0.359670321
	H282	-0.041214956	13.328846385	0.740854532
	H283	2.920365593	10.159949423	1.478463791
	H284	-1.703271359	10.995717233	2.433031378
	H285	3.164054000	-2.532244109	-9.907488136
	H286	3.300124701	-4.030832308	-8.971917947
	H287	6.888438100	-4.990965935	-6.338126189
	H288	3.336580671	-4.610306915	-11.270635264
	H289	1.828031027	-5.009430803	-10.769279430
	H290	5.649094028	4.977754646	-1.261856983
	H291	6.089794331	7.715636144	0.113475705
	H292	4.659534026	8.113871421	-3.925413103
	H293	4.430699516	5.879526122	-0.301239789
	H294	7.064621752	8.260441953	-1.081210037
	H295	4.440194155	3.142109691	-7.389050142
	H296	6.020628191	6.913282088	-2.707844845
	H297	5.882110536	-6.923131770	3.768132486
	H298	7.269174059	-7.934919631	1.226669521
	H299	2.677867308	1.711979607	-7.201153510
	H300	5.182273216	0.004779681	2.267582299
	H301	-0.645856016	1.990452703	-8.492153042
	H302	-1.968558733	-2.244369919	-4.109684689
	H303	-0.488583191	2.462583357	-6.798805907
	H304	4.796142986	-4.459976038	3.342092160
	H305	1.214954549	0.404895434	-6.734678795
	H306	4.436395430	-4.940035297	1.680638382
	H307	5.157833738	-7.162536497	2.163722233
	H308	6.864824885	-5.401462645	1.279650226
	H309	7.221777163	-5.025951800	2.975658561
	H310	-6.001669280	-0.352011675	-5.635725865
	H311	6.507114637	-9.389728916	3.735030792
	H312	5.136227498	-11.766254664	2.347273385
	H313	-0.378813205	-0.332416671	-3.866684192
	H314	7.015258611	-11.716453764	4.580424112

	H315	5.407217740	-12.026808911	4.576586530
	H316	5.659472325	-3.077344211	0.839050876
	H317	-1.089644672	1.943448992	-4.488629462
	H318	-4.251213026	-1.858398397	-5.002392834
	H319	7.082927331	-9.854835565	-2.962973067
	H320	5.754647001	-7.433222076	-4.876517165
	H321	6.202867226	-8.224853241	-1.330814004
	H322	6.150180189	-10.464502164	-4.028150735
	H323	3.481086700	2.874937499	-10.060662462
	H324	6.134202856	-5.076370553	-1.458275350
	H325	2.378104546	-7.480300282	0.284974566
	H326	2.184540446	-1.649734180	-7.995013667
	H327	2.497343156	-8.985904956	-1.673871130
	H328	1.852239993	-6.422668635	-7.694757363
	H329	1.311567409	-5.016151908	-5.647701498
	H330	6.779040214	-12.415538571	2.356409238
	H331	5.220874125	-0.884843699	0.773009632
	H332	8.287651517	-5.939051146	-0.813625608
	H333	7.038538353	-3.819979186	-1.537506234
	H334	9.231541750	-6.981256925	1.122565810
	H335	5.445351191	-2.755977662	4.400784547
	H336	1.877114638	-11.363216257	-4.598936631
	H337	2.631978742	-7.885148386	-4.527532220
	H338	4.085044120	-8.219102733	-3.566989724
	H339	1.155232681	-8.316124010	-2.636765634
	H340	0.501788130	-1.807460736	-6.288999345
	H341	-3.386491184	2.343800178	-5.430939328
	H342	-5.783008372	1.414095936	-5.655680692
	H343	1.962358458	-5.762375951	0.010573390
	H344	8.282329190	-7.482948192	-0.974621856
	H345	7.044129784	-9.338472739	-0.647351632
	H346	4.821885959	-1.150902040	4.313822183
	H347	4.305308366	-7.207308117	-7.700574839
	H348	5.799825450	-5.801502802	-4.218929752
	H349	-0.610056323	-2.529457511	-7.491586488
	H350	3.324098946	4.516343483	-7.524599787
	H351	7.961284395	-0.347623010	2.924951428
	H352	7.951822595	0.210862400	1.232945680
	H353	7.725592384	-6.566259985	-7.861632644
	H354	7.092208630	-7.840111920	-7.055472110
	H355	9.825526874	-8.639353659	-3.665625019
	H356	7.813440068	-8.146588800	-4.628444096
	H357	10.817942643	-7.284768377	-5.851221666
	H358	9.749060128	-7.001430867	-2.977977987
	H359	10.683638478	-0.362015043	1.007895616
	H360	-2.095111501	13.184142945	4.610192717
	H361	10.856876313	-7.093940704	3.656298589
	H362	10.219090526	-4.619220692	2.615079029
	H363	-4.264638451	12.368391343	2.711177763
	H364	-2.992049774	10.129266222	4.399395133
	H365	-4.658621652	10.712488641	4.540267384
	H366	-6.320917665	7.270004355	-0.684788866
	H367	1.907304034	-3.779552275	-0.188500755
	H368	0.771615328	-1.036583192	0.274769526
	H369	-1.579695407	0.070610559	-0.553553653
	Mo370	3.206689473	-4.136402695	-3.079752468
	N371	-6.950787007	-6.963468476	0.207684365
	N372	-8.623811296	-4.417164842	2.379816183
	N373	-8.118695736	-4.858317691	5.133345547

	N374	-5.625505878	-6.135903475	5.088437183
	N375	-3.950599215	-4.061954862	4.032068326
	N376	-4.222194754	-2.165268589	6.153715997
	N377	-3.312084786	-3.602831699	8.479520434
	N378	-0.831009293	-4.879063668	7.817931711
	N379	0.760922012	-3.236094673	6.220822265
	N380	0.527586681	-0.309387487	6.720578822
	N381	0.198924780	-0.390724555	9.602614435
	N382	6.124719575	-11.422621207	4.173132092
	N383	6.722450879	-9.227790938	2.745488293
	N384	5.505223754	-3.162201683	1.847570438
	N385	5.039921920	-2.018743724	3.833434662
	N386	5.042430176	-0.888087583	1.798947026
	N387	9.454031772	-7.099279897	2.134390504
	N388	-6.177463443	-2.761761396	-2.200249453
	N389	-2.304754755	-5.374419657	-3.073137732
	N390	-6.327511987	-0.291382808	-1.330981474
	N391	-8.371319141	0.558208949	0.340097760
	N392	-7.297611448	0.450690846	2.891114021
	N393	-5.147465575	2.226679421	3.114412436
	N394	-3.173825147	2.789935484	-0.635304465
	N395	-2.225013594	0.868271302	-0.345539984
	N396	-5.603494524	4.821813451	3.983256279
	N397	-9.016288276	7.991047288	3.309408384
	N398	-8.692869907	9.715827990	4.666646690
	N399	9.921154908	2.046819162	1.501219920
	N400	6.558343811	7.432762054	-0.753548403
	N401	4.186031975	8.569859280	-3.150201708
	N402	1.521738440	7.744235813	-3.272568450
	N403	-3.269499107	6.465666992	-3.828194057
	N404	-2.901707386	8.488866323	-4.951740949
	N405	-4.118856964	8.448473825	-2.981714162
	N406	0.840131413	6.624979887	-0.733493295
	N407	1.421635699	8.828119528	0.920375669
	N408	-0.033062713	11.265355267	0.904906570
	N409	-1.538172465	10.216038138	-1.583326066
	N410	-2.264237332	11.844236057	2.422458340
	N411	2.446351995	-4.215351040	-10.959976535
	N412	1.315073026	-2.163992746	-8.183585285
	N413	0.394012690	0.623342589	-7.333096459
	N414	2.461914905	2.586688680	-7.698933786
	N415	-3.098915862	-2.409890674	-9.830944460
	N416	-4.447812462	-0.642975598	-8.046791521
	N417	6.932420145	-6.853199547	-7.280582193
	N418	3.364223025	-5.366662061	-5.106016935
	N419	2.532341200	-6.188806572	-6.977067143
	N420	8.500582257	-7.392413120	-4.663440249
	N421	0.232818516	2.618161857	-0.147689540
	N422	0.861850360	1.916610993	-0.772993144
	O423	-6.443256413	-5.064922471	2.294753175
	O424	-6.962630181	-2.894289874	5.186792384
	O425	-5.309142686	-5.539640964	7.274386405
	O426	-2.273199277	-4.991184898	5.257846902
	O427	-2.112152456	-1.481961935	5.651067949
	O428	-2.480204421	-1.602695159	9.199574031
	O429	-0.303857247	-4.867001576	10.055314048
	O430	2.491012209	-4.749992498	6.276531099
	O431	2.798667235	-0.517697677	6.547103208
	O432	1.799063691	1.219494010	9.620858042

	O433	2.322670196	-0.896489712	12.452931307
	O434	6.901361344	-10.398459753	0.756371780
	O435	8.683465768	-7.771123620	4.193947904
	O436	12.240262044	-4.855655130	2.485429216
	O437	-5.504616942	-0.758432014	0.757864636
	O438	-4.220412060	-4.267442786	-3.592238943
	O439	-7.546129048	2.687672152	0.367867912
	O440	-6.696235684	3.338658610	-2.145866693
	O441	-9.237838508	1.289220273	3.796501470
	O442	-4.579194000	1.376766349	5.158646138
	O443	-3.365886652	5.159583022	4.301690156
	O444	-5.345398888	7.260950379	6.540155000
	O445	10.829590037	-1.071982270	2.917032463
	O446	4.400093605	4.670869124	3.566841147
	O447	3.868442352	8.643787444	-0.895651257
	O448	0.803524717	9.927732962	-3.261111525
	O449	-1.330142839	7.172931656	-1.215630683
	O450	0.037105015	7.979600388	2.534517334
	O451	2.804784261	6.058107705	1.534491625
	O452	1.551575476	12.268554120	2.224661655
	O453	-2.533334303	13.892541407	1.449684250
	O454	-3.378430384	11.426433547	-0.968393036
	O455	-4.050909164	13.328119319	5.177512215
	O456	-5.363084093	7.171163107	-0.520688103
	O457	0.381195973	-4.034138536	-9.140384509
	O458	-1.443624161	-0.288881953	-8.358402636
	O459	0.913657771	4.046597701	-8.512359433
	O460	4.652637175	4.529354516	-9.820696287
	O461	-6.208244800	-2.091186027	-8.155140451
	O462	-2.383717050	-6.277159527	-6.059802823
	O463	-0.625052021	-5.371833766	-7.088581001
	O464	-4.946062923	2.842996834	-7.587305692
	O465	9.090708039	-5.278672399	-5.226397868
	O466	12.039645873	-6.928635074	-4.256446968
	O467	-0.522002312	-6.301865723	-0.702398729
	O468	0.000793855	-8.371175518	-0.079402732
	O469	4.027317695	-10.621581787	-4.348339568
	O470	1.776094815	-10.432047060	-4.307226357
	O471	4.590828912	-5.684713328	-2.178133284
	O472	4.646794752	-7.550862195	-0.913419323
	O473	2.004844629	-5.805299675	-2.606968797
	O474	8.845832088	-6.737889329	-0.635422391
	O475	6.945646055	-4.665744378	-1.058411422
	O476	-4.545236037	4.984759017	-1.732158998
	O477	7.099838684	-10.369960529	-3.809242594
	O478	7.053537216	-8.737849991	-1.437852027
	S479	3.524468255	5.202784418	-2.447501580
	S480	1.657744651	2.193308009	-3.581345661
	S481	1.348629784	-2.895366138	-3.974137533
	S482	3.684187198	1.813437191	-0.508308533
	S483	0.411824833	-0.969963211	-1.044719214
	S484	3.323480967	-0.353873605	-6.157725533
	S485	3.072094948	-3.269847345	-0.756821390
	S486	5.296558248	1.971417702	-3.688811066
	S487	5.100092714	-3.022819316	-4.037595774
	S488	6.246099080	-0.890442403	-1.245054877

TS 8 → 9 35 (S=1/2)	bm22b2n2x3b33b235th_1_53081.918			
Fe(139) -2.270	C1	-7.947460982	-5.034035658	0.660339057
Fe(140) -1.725	C2	-8.443339634	-5.770826791	-0.589196747
Fe(141) -2.543	C3	-8.543892165	-3.544494854	2.519275946
Fe(142) 2.945	C4	-7.921690463	-4.313495455	3.685059328
Fe(143) 2.998	C5	-7.782800147	-6.413566033	4.927199099
Fe(144) 2.026	C6	-6.312871404	-6.782823564	4.694077908
Fe(145) -1.141	C7	-4.539995392	-7.148771218	3.043125702
	C8	-3.653707032	-5.919384995	3.265238481
	C9	-3.440315611	-3.494344032	3.151384889
	C10	-3.234250469	-3.212276652	4.646610199
	C11	-4.169448567	-3.240257757	6.886938835
	C12	-3.261026544	-4.238367543	7.616934930
	C13	-2.722043226	-6.592504243	8.014497411
	C14	-1.306813599	-6.939350735	7.533847443
	C15	0.180322264	-7.218177803	5.594901970
	C16	1.114880664	-6.125317696	5.071896115
	C17	1.377279210	-3.705585330	4.601289883
	C18	1.676479422	-2.726514764	5.751144632
	C19	0.696793388	-3.028379473	3.381778952
	C20	1.419770151	-1.739680583	2.973985538
	C21	0.605409466	-4.004485861	2.203612760
	C22	0.758841473	-1.226307265	7.477608241
	C23	1.095832782	-1.948509643	8.796663537
	C24	-0.481094567	-0.307613576	7.600986346
	C25	-0.394064551	0.580320435	8.847814422
	C26	-0.646288148	0.550368205	6.339812124
	C27	0.525829623	-3.673557112	10.405951553
	C28	1.947503272	-4.174700674	10.558364662
	C29	5.428809977	-12.248067973	-0.742152967
	C30	5.974526034	-10.870978480	-1.122844517
	C31	6.592801619	-8.651820387	-0.145040931
	C32	7.961587610	-8.597035332	0.560983295
	C33	5.576827576	-7.696619272	0.505840351
	C34	6.024847064	-6.232590129	0.523697660
	C35	4.849864302	-5.308371475	0.843436177
	C36	5.054048963	-2.994716433	1.776599297
	C37	10.302750976	-7.901434572	0.361108381
	C38	10.721529815	-6.494688080	0.730696512
	C39	-5.658861002	-1.823985574	-1.410548526
	C40	-5.841868566	-0.585541742	-0.514773113
	C41	-4.145542987	-2.031456479	-1.637366354
	C42	-3.732951696	-3.391429334	-2.237808085
	C43	-3.701892205	-3.450377043	-3.764357092
	C44	-6.381258840	1.807665368	-0.549683834
	C45	-7.435518559	1.971478703	0.547812834
	C46	-6.526759353	2.934628119	-1.592983581
	C47	-9.186730728	0.933784521	1.893374640
	C48	-8.478668541	0.593149652	3.212632966
	C49	-6.456591017	-0.285116955	4.296742977
	C50	-5.202449750	0.533614343	4.592231524
	C51	-3.763712085	2.425974507	3.976251829
	C52	-3.947584541	3.466031008	5.107299247
	C53	-3.394617409	3.236334134	2.725967566
	C54	-2.969478738	2.460924380	1.520000312
	C55	-2.431649963	1.212107911	1.326236174
	C56	-2.488904073	2.228908675	-0.647279989

	C57	-5.565988282	5.023781396	6.132984085
	C58	-5.240620546	4.691610736	7.586749062
	C59	-7.078695808	5.301229353	5.959438879
	C60	-7.562235123	6.661161285	6.369995562
	C61	-7.275545977	7.427913045	7.483639289
	C62	-8.710934411	8.484156250	6.165902093
	C63	9.637697449	-0.326973763	2.313759142
	C64	10.398933427	-1.456623572	1.645288031
	C65	8.127986582	-0.625829537	2.112612397
	C66	7.230740633	0.387215796	2.783335605
	C67	6.840037108	0.241865574	4.128399341
	C68	6.768034262	1.516148324	2.082082338
	C69	6.003276563	1.177544786	4.748858737
	C70	5.944332788	2.463765384	2.691630468
	C71	5.561946502	2.292481897	4.025826216
	C72	5.987028097	6.936334522	0.113402439
	C73	4.975198805	8.075007252	0.290963963
	C74	5.264076328	5.580865166	0.164579800
	C75	3.774228481	9.921789416	-0.793791676
	C76	2.302511950	9.510352362	-0.751646243
	C77	0.665065051	7.684743450	-1.033014113
	C78	0.315198780	7.133647605	0.366896048
	C79	0.494102515	6.636767297	-2.148673686
	C80	-0.878707666	5.973108882	-2.296217270
	C81	-2.024243630	6.836578982	-2.856073087
	C82	-2.974638571	8.773795187	-1.579716484
	C83	1.041470841	6.005013884	2.406760075
	C84	1.201956555	7.067263644	3.513530481
	C85	1.904712081	4.792678646	2.772403769
	C86	2.570493584	8.922032732	4.341220786
	C87	1.922968753	10.280484210	4.608897631
	C88	0.149999073	11.882397317	3.992388913
	C89	-0.985877276	12.000609791	5.031677812
	C90	-0.344739131	12.344506237	2.600088626
	C91	-1.387080265	11.386734937	2.035888672
	C92	-2.524469526	10.788063810	6.518370843
	C93	-2.199620011	11.382624976	7.885063425
	C94	-3.012314256	9.322167569	6.676662366
	C95	-3.524465280	8.741012127	5.377226330
	C96	-4.842354194	8.986686484	4.960094474
	C97	-2.694207406	7.983313007	4.536384826
	C98	-5.314198710	8.515049060	3.733207703
	C99	-3.141601720	7.518843421	3.295844300
	C100	-4.448750409	7.799323337	2.905179000
	C101	2.295831556	-0.854797240	-10.381342264
	C102	1.008927251	-0.743846349	-9.579712988
	C103	-0.051368895	0.273466349	-7.579507550
	C104	-0.521285815	1.730528017	-7.542958449
	C105	0.044257563	3.996945365	-6.803501163
	C106	1.226482032	4.957167463	-6.884558114
	C107	3.626704291	5.353782331	-6.715781182
	C108	4.107176840	5.807265919	-8.080174098
	C109	-4.385654058	-0.185012072	-9.163789356
	C110	-5.256785726	0.769078786	-8.335558431
	C111	-3.784019396	-1.267658346	-8.234678308
	C112	-3.251142012	-2.465385776	-9.043634235
	C113	-2.404875077	-3.371526232	-8.177009263
	C114	-5.348030593	2.754233107	-6.902458237
	C115	-4.885136280	4.173223540	-7.175397979

	C116	-5.376422785	2.365052956	-5.402183051
	C117	-4.029450427	1.939118872	-4.869577146
	C118	-3.070378543	2.879668747	-4.452246963
	C119	-3.679692548	0.578249450	-4.854441092
	C120	-1.788901407	2.465947489	-4.068942795
	C121	-2.397983628	0.165187174	-4.475365378
	C122	-1.443277912	1.110532908	-4.087701413
	C123	6.478827161	-4.565404692	-7.681877289
	C124	7.783298756	-5.005695134	-6.993854235
	C125	5.242338989	-5.070578714	-6.919306439
	C126	3.918186138	-4.557057289	-7.397911344
	C127	3.356719135	-4.712135441	-8.649511033
	C128	1.876317404	-3.778636491	-7.291305761
	C129	9.173679827	-6.796834129	-6.109909638
	C130	10.407792747	-6.335746339	-6.853408288
	C131	-0.055474820	-6.746155185	-2.350009748
	C132	1.428614821	-6.516550455	-2.312669703
	C133	2.052305258	-6.232066300	-3.738921307
	C134	1.699760651	-7.426336954	-4.672929568
	C135	2.441014196	-7.371983433	-6.017814921
	C136	2.347914892	-8.637775495	-6.822155396
	C137	3.599531773	-6.244300915	-3.538979858
	C138	3.184783563	0.005136893	-2.943474447
	Fe139	3.060190488	-0.698887860	-4.836840340
	Fe140	1.997245956	1.138312959	-1.590191411
	Fe141	4.757663611	0.759620031	-1.912892022
	Fe142	3.414106101	1.717537649	-3.974328295
	Fe143	3.789396052	3.197726448	-1.711707728
	Fe144	4.342858169	-1.620591939	-2.752194967
	Fe145	1.856249135	-1.327010839	-2.728287290
	H146	-6.734696346	-6.826461230	-0.933279237
	H147	-6.893142908	-5.517169703	-1.895613038
	H148	-9.836193721	-4.284617773	0.986436148
	H149	-9.115755993	-5.106243084	-1.153492479
	H150	-9.071964649	-6.606272694	-0.235221998
	H151	-7.814200672	-2.771617453	2.239801706
	H152	-9.065649506	-5.964648118	3.256834225
	H153	-9.454586419	-3.046462408	2.878601225
	H154	-8.369920623	-7.338279312	4.996116198
	H155	-6.575578397	-6.636966746	2.664219612
	H156	-7.832491028	-5.897221758	5.894980355
	H157	-2.442933091	-3.537510053	2.694336085
	H158	-5.076030838	-4.685600600	2.409573432
	H159	-4.495175205	-7.446100234	1.986452105
	H160	-3.761050467	-2.237374151	7.066035303
	H161	-5.183718265	-3.768626381	5.063883590
	H162	-4.121122633	-7.959796937	3.650388894
	H163	-3.992808823	-2.662315540	2.691245970
	H164	-4.154114450	-5.822952025	6.629598879
	H165	-5.174201691	-3.292481902	7.327700330
	H166	0.055395312	-4.916613040	2.475194791
	H167	0.084750305	-3.533077450	1.355699735
	H168	1.608076156	-4.298077174	1.855665579
	H169	-0.327383496	-2.763239013	3.689889983
	H170	-3.322116414	-7.511417537	7.954858817
	H171	-0.747174428	-0.062965572	5.433434684
	H172	-0.539119347	-3.230488856	8.616103749
	H173	-0.364087687	-0.006624088	9.777042490
	H174	0.869116587	-1.243021216	2.160741343

	H175	2.434090587	-1.942293304	2.601766754
	H176	9.848899435	-0.378248208	3.399430697
	H177	-1.547290834	1.174814502	6.424417295
	H178	-1.270440037	1.243952161	8.893343317
	H179	0.221083998	1.218105077	6.210816909
	H180	0.508066138	1.211525231	8.816537246
	H181	-1.370630932	-0.956263346	7.701145135
	H182	1.643840958	-0.607743994	7.266791235
	H183	-0.344751381	-2.355428285	6.017244109
	H184	1.496757211	-1.019990803	3.803142208
	H185	1.866644760	4.066458659	1.943287845
	H186	1.452758799	4.332037379	3.666772278
	H187	3.696485271	4.493766742	3.517751722
	H188	4.506809338	3.000713631	5.493443274
	H189	5.574269372	3.320004094	2.127085510
	H190	7.020526079	1.639738733	1.026053454
	H191	7.183728315	-0.623810310	4.702461320
	H192	-0.337277261	-4.725548505	5.393640138
	H193	-0.031507074	-7.890286747	4.749442677
	H194	0.742906009	-7.791112000	6.343862684
	H195	-1.834265244	-6.543452029	5.566050896
	H196	-2.629893795	-6.310694243	9.071244490
	H197	5.698180073	1.037877292	5.789904260
	H198	2.354911246	-4.099675607	4.294182756
	H199	0.274405791	-3.038003272	11.271464192
	H200	-0.133520583	-4.555514022	10.432601850
	H201	2.480783534	-4.376045394	9.599784966
	H202	11.024415296	-8.319652283	-0.355930845
	H203	-2.671000432	-4.276102052	-5.327033874
	H204	0.301495463	-8.636392189	-2.217401851
	H205	-4.100657890	-3.050354301	-9.425789929
	H206	-2.659458482	-2.109090032	-9.899827964
	H207	-4.545791433	-1.626335380	-7.525176266
	H208	-4.534473444	4.345728956	-8.219846348
	H209	-5.108671604	-0.682000479	-9.829430462
	H210	-3.772623683	-1.211543228	-2.271555264
	H211	-4.424385556	-4.181811116	-1.897837863
	H212	-3.603120357	1.914261054	-7.825971458
	H213	-6.395190347	2.731151907	-7.265422184
	H214	-2.641078383	0.896937174	-9.469555623
	H215	-2.012499518	-4.663085202	-3.736257766
	H216	-2.984127033	-0.808652520	-7.633009633
	H217	-3.054386316	-0.088269553	-10.721087436
	H218	-7.303954687	-2.120939938	-2.625784946
	H219	-3.654966501	-1.930718441	-0.657674199
	H220	-0.802644974	-2.460688891	-8.881733783
	H221	-2.745835994	-3.671888535	-1.844650917
	H222	-2.928564302	1.782052259	4.286743096
	H223	-2.223079933	0.409569129	2.024757165
	H224	-7.121526855	-0.176578879	5.165033043
	H225	-6.795523726	-0.102923716	2.196276827
	H226	-5.973743388	3.380016971	4.821420583
	H227	-6.620308903	7.270706258	8.333309514
	H228	-5.422629126	3.622472098	7.856741929
	H229	-7.650659091	4.507236532	6.471956272
	H230	-4.978040004	5.904558580	5.832427308
	H231	-9.376831439	9.267002432	5.812806010
	H232	-2.573174348	3.911202600	3.018446722
	H233	-4.246056391	3.881436283	2.452714388

H234	-8.031234960	9.366772870	7.979957027
H235	-7.307783200	5.202812288	4.886828154
H236	-5.549041616	1.754565102	2.962596062
H237	-6.164212936	-1.344058510	4.247909276
H238	-9.669395926	1.908991778	2.037733466
H239	-2.356905522	2.428152621	-1.707252405
H240	-9.975229290	0.188570143	1.724400569
H241	10.930964196	1.235192119	2.011030779
H242	9.357279159	1.675344124	1.991821583
H243	2.162383785	6.212780283	0.562636373
H244	-0.010986160	5.691080432	2.444294842
H245	2.627507289	8.054310995	2.414541420
H246	-3.357518536	4.036168568	0.091513421
H247	-5.035908026	5.166560184	-0.534213517
H248	-4.458604701	6.068713607	0.645310447
H249	-5.705104729	2.865610985	-2.319063108
H250	-7.483896984	2.827509913	-2.130647423
H251	0.528683520	12.423693668	1.935847438
H252	-0.798874929	13.336447044	2.719558587
H253	0.026503650	10.415883423	0.814689954
H254	-1.525562821	9.647016901	0.965594613
H255	-2.596628914	10.685841326	-2.204888832
H256	-2.699024940	6.212192410	-3.459802000
H257	-6.347487950	8.702712321	3.429705126
H258	-1.673085595	7.738088619	4.839354399
H259	-3.950309559	8.520853203	0.226725524
H260	1.231033495	5.832686458	-1.995900533
H261	2.787387984	7.568268101	-1.259009010
H262	-1.195222877	5.516362018	-1.345469275
H263	-8.277473690	0.175909079	0.098455159
H264	3.926771063	10.541828180	-1.686764046
H265	-6.552735730	0.307735504	-2.160092658
H266	0.762437938	7.127062182	-3.098856346
H267	-2.462639641	6.988045543	2.626794016
H268	-6.912091959	4.132329848	-0.096318195
H269	3.944517287	10.555596510	0.086978351
H270	-2.251245877	9.466924446	-3.375402962
H271	-3.904586477	10.149015633	-0.371124628
H272	-5.848790055	-2.077263607	-3.443172249
H273	-6.023945747	-2.673791984	-0.803230671
H274	-5.520863729	9.550173092	5.604226071
H275	-5.426585379	1.960852644	-0.020269874
H276	-0.027667815	8.523652791	-1.186684832
H277	-1.622302598	7.598319028	-3.535322149
H278	-0.729117454	5.138541826	-2.996013523
H279	-3.338218353	6.816351648	-1.181157370
H280	2.502768779	8.353511416	5.281148465
H281	0.496189573	9.925127096	3.178335927
H282	0.923607678	12.586500643	4.332809056
H283	3.633241978	9.116269375	4.142615080
H284	-0.904345222	9.987373493	5.358138213
H285	2.851020295	0.093369302	-10.330463495
H286	2.911520111	-1.613604608	-9.854354121
H287	6.491741219	-3.466853264	-7.644080791
H288	2.866782567	-1.500045794	-12.228160117
H289	1.361569399	-1.980222459	-11.794618505
H290	6.005462462	4.792929653	-0.021487959
H291	6.575264664	7.009489625	2.065523137
H292	5.179240501	8.579414044	-1.690333131

	H293	4.827327097	5.442290653	1.164612371
	H294	7.575272802	7.823631882	1.060946337
	H295	4.455405607	4.826441138	-6.217705908
	H296	6.478407742	7.037245611	-0.867582485
	H297	5.370838524	-8.043062668	1.532098778
	H298	6.709594517	-8.387189869	-1.203092384
	H299	2.652449525	3.466016592	-6.570941370
	H300	5.204590518	-1.003819776	2.189748341
	H301	-0.656150018	4.265974620	-7.604529134
	H302	-2.134119561	-0.894693139	-4.491579483
	H303	-0.465562851	4.217668879	-5.850895735
	H304	4.420593458	-5.532240247	1.829770642
	H305	1.130606125	2.151996977	-6.418787809
	H306	4.045815584	-5.462235760	0.105188183
	H307	4.641135319	-7.793268386	-0.067717194
	H308	6.427127988	-5.940711553	-0.457918899
	H309	6.826837535	-6.081080247	1.264320758
	H310	-6.093696842	1.538688258	-5.289790720
	H311	5.867228432	-10.432504454	0.824206685
	H312	4.397700361	-12.281095220	-1.125474758
	H313	-0.430782019	0.803672702	-3.815163349
	H314	6.288318731	-12.932819299	0.998536523
	H315	4.665786668	-13.147988270	0.944161491
	H316	5.354132122	-3.492292769	-0.144998161
	H317	-1.030338025	3.188297681	-3.758635359
	H318	-4.414793211	-0.171927726	-5.151827179
	H319	6.404288244	-9.089269712	-5.750991720
	H320	5.225365635	-6.174029421	-6.975418186
	H321	5.664036797	-7.908117242	-3.721950299
	H322	5.425679230	-9.346343943	-6.915459199
	H323	3.609196939	5.314549967	-8.951179268
	H324	5.788083707	-4.853100703	-3.021007489
	H325	1.953328968	-7.374923259	-1.868815176
	H326	1.942999005	0.500786127	-8.239916785
	H327	1.963574021	-8.360813697	-4.153594960
	H328	1.379360560	-4.204215172	-9.301044125
	H329	0.942730705	-3.354013683	-6.942296779
	H330	6.005915489	-12.985704208	-1.326002354
	H331	5.108896245	-1.389289547	0.493502260
	H332	7.876750056	-5.978963478	-2.636631592
	H333	6.730461338	-3.670785250	-2.675617199
	H334	8.735424255	-7.584778569	-1.085182631
	H335	5.273572994	-4.311220837	3.343894141
	H336	1.108509289	-9.823066325	-7.620555926
	H337	2.035664356	-6.543529759	-6.616899682
	H338	3.508364549	-7.181367240	-5.864012545
	H339	0.614120880	-7.419825536	-4.850847418
	H340	0.278072843	-0.058782821	-6.581411539
	H341	-3.327515949	3.942718047	-4.457506280
	H342	-5.768005960	3.234720081	-4.852574496
	H343	1.621436143	-5.626715952	-1.700486305
	H344	7.777648413	-7.415103754	-3.214437364
	H345	6.434139826	-9.218272476	-3.387772966
	H346	4.783451179	-2.722217738	3.799935265
	H347	3.762434010	-5.129044377	-9.563799672
	H348	5.350219903	-4.818915995	-5.857492338
	H349	-0.890058872	-0.345235773	-7.906871802
	H350	3.393712546	6.246399851	-6.114864074
	H351	7.937411447	-1.629628592	2.521770706

H352	7.915558213	-0.653779331	1.031406937
H353	7.256212627	-4.546466738	-9.579647780
H354	6.564043513	-5.974289822	-9.184808690
H355	9.168417457	-7.897496178	-6.139689970
H356	7.207285195	-7.019746139	-6.897367747
H357	10.254978929	-6.120795279	-7.938137624
H358	9.240122466	-6.493903145	-5.052228226
H359	10.627632787	-1.285079192	0.565674797
H360	-1.113416176	11.490768311	8.113024366
H361	10.350235010	-8.504928701	1.283469473
H362	9.887893148	-5.801881071	1.001006318
H363	-3.341906067	11.393037659	6.093438087
H364	-2.185784292	8.709960789	7.071767389
H365	-3.808924584	9.327180639	7.435477416
H366	-5.815196426	7.560709201	1.534616822
H367	1.577280955	-3.142534963	-1.419995150
H368	0.678401334	-1.059593383	-0.079064948
H369	-1.579323256	0.348096497	-0.474244671
Mo370	2.895912462	-3.355912667	-4.264481722
N371	-7.416128622	-6.288915870	-1.475583196
N372	-8.899004157	-4.367347240	1.372512362
N373	-8.351435448	-5.585322109	3.874586727
N374	-5.933251430	-6.927715620	3.400130275
N375	-4.178919928	-4.723509115	2.895833929
N376	-4.283837858	-3.484416477	5.458562394
N377	-3.420364436	-5.545961615	7.286683353
N378	-1.065789858	-6.780447675	6.200472528
N379	0.622636034	-4.859006852	5.080984055
N380	0.597123405	-2.169865668	6.369491746
N381	0.309779782	-3.002108590	9.137903060
N382	5.412738154	-12.494197287	0.704998592
N383	6.092506298	-10.018504801	-0.087405618
N384	5.212183955	-3.887074485	0.789777721
N385	4.924672704	-3.402385582	3.058718977
N386	4.950952753	-1.688970048	1.479856784
N387	8.946371972	-7.988788831	-0.146923698
N388	-6.394474731	-1.656416822	-2.669254078
N389	-2.720624437	-4.197140612	-4.306638456
N390	-6.350263092	0.496540704	-1.164370031
N391	-8.318944973	0.972094454	0.730643077
N392	-7.210127817	0.096100542	3.115545218
N393	-4.939223748	1.591328312	3.761675231
N394	-2.993245965	3.066522113	0.270286718
N395	-2.161816571	1.091792689	-0.023339421
N396	-5.225816116	3.885951062	5.288509207
N397	-8.459414826	7.332444814	5.555568232
N398	-8.014651940	8.582165497	7.333896620
N399	10.005644020	0.944550677	1.685600732
N400	7.032000468	6.959805121	1.148573111
N401	4.732203198	8.834436810	-0.813571569
N402	2.016296465	8.236793823	-1.100316318
N403	-2.849874482	7.469024878	-1.819335699
N404	-2.459015652	9.701761694	-2.411122315
N405	-3.573351441	9.191692189	-0.445095391
N406	1.304139921	6.506680866	1.059380888
N407	2.037663510	8.113235322	3.251243674
N408	0.773823543	10.564458364	3.925910659
N409	-0.879815921	10.363114840	1.295576628
N410	-1.378018697	10.846806343	5.625889095

	N411	2.004692485	-1.183243258	-11.778972923
	N412	1.044390025	0.091512968	-8.524247330
	N413	0.320716537	2.575862939	-6.911719143
	N414	2.483292874	4.460165090	-6.772641627
	N415	-3.437289647	0.543815176	-10.015262297
	N416	-4.622377276	1.826382949	-7.754274048
	N417	6.449182774	-4.959233920	-9.103342927
	N418	2.972107817	-3.961598408	-6.559843021
	N419	2.073890476	-4.215976640	-8.559446750
	N420	7.948055291	-6.335078367	-6.742068370
	N421	0.380541302	2.634245283	0.438872852
	N422	0.943922306	2.051408004	-0.350220854
	O423	-6.762800637	-5.079685412	1.040813032
	O424	-7.073782104	-3.775386142	4.415919262
	O425	-5.541188809	-6.958087802	5.651510845
	O426	-2.522893256	-6.014882606	3.767104597
	O427	-2.165473641	-2.747680840	5.074697579
	O428	-2.457140141	-3.856797398	8.484253278
	O429	-0.472066510	-7.376345336	8.336564556
	O430	2.241375302	-6.434163719	4.655033610
	O431	2.844676903	-2.469971682	6.081012903
	O432	2.037894222	-1.578239863	9.516363569
	O433	2.461368921	-4.406802120	11.642387210
	O434	6.247280594	-10.626022795	-2.317384785
	O435	8.112250519	-9.085848430	1.694405168
	O436	11.886069541	-6.129633236	0.759438643
	O437	-5.550688776	-0.575830943	0.698457256
	O438	-4.548245665	-2.853804523	-4.467575445
	O439	-7.417175981	3.002946539	1.256538638
	O440	-6.453430697	4.227777952	-0.970084522
	O441	-9.074130638	0.765134687	4.282831402
	O442	-4.480647732	0.245705459	5.550121988
	O443	-2.977793653	3.941945093	5.703549704
	O444	-4.851718258	5.501007708	8.410722371
	O445	10.730538248	-2.487274695	2.214257361
	O446	4.729602790	3.247319596	4.576805535
	O447	4.414433305	8.304163003	1.378705745
	O448	1.445816576	10.360837263	-0.434143317
	O449	-0.833923641	7.294734985	0.817762547
	O450	0.614561584	6.921283966	4.595080110
	O451	3.244653847	5.217385700	3.036053634
	O452	2.427138850	11.044633346	5.440991533
	O453	-1.491422178	13.101205685	5.290681050
	O454	-2.590829526	11.481092723	2.321618668
	O455	-3.058485268	11.704383092	8.689851964
	O456	-4.862952783	7.365697040	1.631525406
	O457	0.004944551	-1.435611449	-9.893362664
	O458	-1.595543258	2.086581381	-8.075746411
	O459	0.993664399	6.172600363	-7.008709440
	O460	4.990079989	6.635565846	-8.234511724
	O461	-6.473856455	0.565083364	-8.187616465
	O462	-2.874677787	-4.217805356	-7.421964426
	O463	-1.071413803	-3.187341250	-8.234286462
	O464	-4.918264544	5.083186971	-6.359281158
	O465	8.694550803	-4.196483848	-6.739427186
	O466	11.514924376	-6.264547210	-6.339342892
	O467	-0.910605322	-5.875644335	-2.435594679
	O468	-0.475333431	-8.050595974	-2.296967815
	O469	3.300685376	-9.304986085	-7.218219673

	O470	1.065899732	-8.994808539	-7.097039405
	O471	4.207721476	-5.154176081	-3.867701376
	O472	4.170544662	-7.280083535	-3.120868472
	O473	1.614270880	-5.027155319	-4.250914383
	O474	8.385979776	-6.826644756	-2.693639661
	O475	6.601882201	-4.623209398	-2.502172244
	O476	-4.199611461	5.559907512	-0.161796254
	O477	6.382263078	-9.351883299	-6.706278969
	O478	6.479599461	-8.424454829	-3.981873040
	S479	3.897932700	5.437129059	-1.079561587
	S480	1.821749479	3.000774768	-2.963673559
	S481	1.093784709	-1.815007322	-4.727268261
	S482	3.754009020	1.623911323	-0.120828395
	S483	0.283619811	-0.652709888	-1.327002575
	S484	3.220661279	1.122667378	-6.136511100
	S485	2.921170210	-3.262155582	-1.782802889
	S486	5.430194520	2.484252904	-3.158891725
	S487	4.850290789	-2.104251191	-4.838931491
	S488	6.063902377	-1.017003913	-1.527188743

9 35 (S=1/2)

9 35 (S=1/2)	bm22b2n2x3b33b235th.car_2			
Fe(139) 0.537 -2.321	C1	-7.956808993	-5.042372362	0.664194343
Fe(140) 0.434 -1.524	C2	-8.448806965	-5.779767523	-0.585596752
Fe(141) 0.536 -2.460	C3	-8.552910338	-3.548428460	2.518596216
Fe(142) 0.587 2.934	C4	-7.926232335	-4.312167852	3.684368095
Fe(143) 0.567 2.967	C5	-7.779612002	-6.410576352	4.928907308
Fe(144) 0.508 2.323	C6	-6.311011163	-6.780534044	4.690712508
Fe(145) 0.439 -1.650	C7	-4.542489902	-7.155229938	3.038058289
	C8	-3.656606868	-5.925200738	3.262727190
	C9	-3.448039368	-3.499422145	3.146665229
	C10	-3.239594144	-3.212112795	4.640798701
	C11	-4.165658494	-3.240541248	6.885595736
	C12	-3.258054854	-4.243115279	7.610628792
	C13	-2.724763070	-6.595885240	8.013352603
	C14	-1.307693377	-6.932898109	7.532371125
	C15	0.181701890	-7.222211412	5.599032131
	C16	1.116790465	-6.126916460	5.083565310
	C17	1.375465479	-3.710382968	4.611745767
	C18	1.678771885	-2.730123951	5.759482338
	C19	0.696416892	-3.032689043	3.390909666
	C20	1.418970610	-1.745449808	2.976376356
	C21	0.602053587	-4.008885118	2.213013450
	C22	0.756906081	-1.227426891	7.485038879
	C23	1.089073041	-1.950550502	8.806250787
	C24	-0.485997240	-0.311610522	7.599430065
	C25	-0.410713017	0.578592313	8.844996110
	C26	-0.647184305	0.544898535	6.336255092
	C27	0.514964767	-3.679442165	10.413345687
	C28	1.939396414	-4.175747005	10.558845103
	C29	5.436357477	-12.252920870	-0.733481544
	C30	5.972068631	-10.872481680	-1.117179641
	C31	6.599663833	-8.652779794	-0.143986411
	C32	7.967097168	-8.589592739	0.562889037
	C33	5.583161410	-7.697035246	0.506563908
	C34	6.032161080	-6.232861837	0.526936860
	C35	4.859443510	-5.309751733	0.858236847
	C36	5.052295600	-2.996863559	1.796166188

	C37	10.308958675	-7.899547775	0.360661604
	C38	10.712446256	-6.485305273	0.720937423
	C39	-5.668486105	-1.831927020	-1.410382374
	C40	-5.852938601	-0.595299646	-0.513170863
	C41	-4.154435161	-2.031497318	-1.638580778
	C42	-3.737402114	-3.390338681	-2.238650652
	C43	-3.701997927	-3.446989915	-3.765307855
	C44	-6.386067321	1.800892124	-0.548754969
	C45	-7.441174871	1.970074272	0.546644309
	C46	-6.528479311	2.923858066	-1.597235621
	C47	-9.189496181	0.931737794	1.895422634
	C48	-8.480388261	0.584796816	3.212332580
	C49	-6.454219045	-0.284597285	4.294715205
	C50	-5.193446535	0.525877352	4.585555966
	C51	-3.763833728	2.431643456	3.981305619
	C52	-3.950445075	3.474305669	5.108343189
	C53	-3.395644312	3.239362308	2.728945661
	C54	-2.972056956	2.460908639	1.525716570
	C55	-2.426035840	1.215395426	1.336004722
	C56	-2.502624849	2.220704558	-0.641889304
	C57	-5.568400090	5.024898998	6.140970781
	C58	-5.231820005	4.697421695	7.593626814
	C59	-7.080445741	5.302671647	5.967938766
	C60	-7.560440272	6.662443646	6.383070910
	C61	-7.258629421	7.428689641	7.492433469
	C62	-8.705701842	8.488278361	6.191246609
	C63	9.639735569	-0.329549552	2.308596156
	C64	10.405930103	-1.460991517	1.648118187
	C65	8.130711823	-0.627144810	2.102420988
	C66	7.233998013	0.385737170	2.774676443
	C67	6.843779409	0.239114613	4.119484185
	C68	6.767802614	1.514128237	2.074453650
	C69	6.007738935	1.173955146	4.741921675
	C70	5.946024515	2.461895441	2.686206605
	C71	5.565422029	2.289346973	4.020648027
	C72	5.988704239	6.934977340	0.113111957
	C73	4.976892806	8.074362044	0.288171420
	C74	5.263016682	5.581244254	0.160241792
	C75	3.775292588	9.919350910	-0.796915761
	C76	2.302185546	9.508648499	-0.757798562
	C77	0.665217142	7.682271329	-1.035814853
	C78	0.316070332	7.136762915	0.365864536
	C79	0.496005585	6.625993799	-2.143720101
	C80	-0.878172340	5.965228409	-2.291301245
	C81	-2.022430253	6.830303963	-2.852709777
	C82	-2.968492087	8.767836056	-1.575757375
	C83	1.040598226	6.007239164	2.405311216
	C84	1.200347620	7.068415459	3.513649659
	C85	1.901177125	4.792701093	2.770009075
	C86	2.567880545	8.923420281	4.343501545
	C87	1.917411127	10.279948503	4.611678029
	C88	0.147716547	11.883286786	3.989894096
	C89	-0.985614584	12.002500394	5.032718998
	C90	-0.353913012	12.341621072	2.598945637
	C91	-1.390985209	11.374829845	2.041120460
	C92	-2.526502838	10.789219873	6.519024760
	C93	-2.197511664	11.376861973	7.887283795
	C94	-3.012026512	9.321064972	6.667883323
	C95	-3.526517798	8.745231749	5.367625660

	C96	-4.843668238	8.996976702	4.951674364
	C97	-2.700985256	7.980295817	4.529387285
	C98	-5.319799415	8.521580204	3.727948647
	C99	-3.153619866	7.511375797	3.292205846
	C100	-4.460761925	7.794288956	2.903086561
	C101	2.299604487	-0.852057653	-10.376719718
	C102	1.013433714	-0.752028215	-9.573527766
	C103	-0.048054899	0.267463364	-7.573508026
	C104	-0.517792345	1.725557958	-7.542206292
	C105	0.053332280	3.993113413	-6.802885697
	C106	1.242070312	4.944905041	-6.898585766
	C107	3.640766667	5.332585951	-6.724403002
	C108	4.102121581	5.816862854	-8.083869365
	C109	-4.383220275	-0.179678470	-9.170012106
	C110	-5.255762328	0.774979225	-8.344187352
	C111	-3.787618714	-1.264063767	-8.241412247
	C112	-3.255490831	-2.464669149	-9.046754465
	C113	-2.412670619	-3.367872332	-8.173318365
	C114	-5.346002524	2.759277662	-6.908443444
	C115	-4.885281248	4.178321651	-7.183984792
	C116	-5.369923160	2.367976196	-5.409832996
	C117	-4.023584426	1.937871587	-4.879884334
	C118	-3.066233307	2.876186935	-4.455324416
	C119	-3.676726288	0.576231940	-4.868728702
	C120	-1.788384151	2.458615554	-4.064572317
	C121	-2.398086333	0.159663790	-4.483502736
	C122	-1.444056004	1.102475267	-4.087616084
	C123	6.470816971	-4.567650561	-7.687687200
	C124	7.775279609	-5.007230310	-6.997569251
	C125	5.234951622	-5.058489237	-6.914801896
	C126	3.910603457	-4.553797425	-7.402691375
	C127	3.352217525	-4.717281173	-8.654710068
	C128	1.869597592	-3.770246480	-7.309132080
	C129	9.166524844	-6.798245715	-6.114482335
	C130	10.400482804	-6.336878076	-6.858815145
	C131	-0.066258780	-6.696601858	-2.352798054
	C132	1.420143030	-6.482140113	-2.321966010
	C133	2.048973309	-6.241191668	-3.752723290
	C134	1.695204354	-7.443778742	-4.674677360
	C135	2.428958200	-7.376475067	-6.022704387
	C136	2.343819776	-8.640412566	-6.829991063
	C137	3.596455956	-6.261641766	-3.544473687
	C138	3.176409629	-0.002888907	-2.936173688
	Fe139	3.064161159	-0.723720343	-4.812356709
	Fe140	2.016928591	1.198327826	-1.551807377
	Fe141	4.751834615	0.754333617	-1.930568215
	Fe142	3.430671263	1.689872982	-3.975289555
	Fe143	3.813597238	3.205999855	-1.739594082
	Fe144	4.379424649	-1.610347942	-2.714598161
	Fe145	1.840145198	-1.381764855	-2.690690501
	H146	-6.734916784	-6.828547592	-0.923399158
	H147	-6.891398545	-5.520153310	-1.884399148
	H148	-9.845147224	-4.289011379	0.985565596
	H149	-9.123034928	-5.118504228	-1.152232862
	H150	-9.074226821	-6.619201554	-0.234331236
	H151	-7.826361719	-2.774953566	2.233306045
	H152	-9.061359422	-5.967778481	3.256843817
	H153	-9.463928775	-3.051020717	2.877732604
	H154	-8.369956971	-7.332894150	5.004410655

	H155	-6.578525087	-6.648112349	2.659828030
	H156	-7.823000803	-5.892023294	5.895891953
	H157	-2.450551467	-3.540705588	2.690166683
	H158	-5.080364457	-4.695140309	2.405796842
	H159	-4.501444649	-7.449799835	1.980574397
	H160	-3.750221827	-2.240134953	7.063202351
	H161	-5.185306129	-3.775164427	5.066976903
	H162	-4.123522047	-7.969808797	3.640955305
	H163	-4.002273590	-2.670027469	2.684348298
	H164	-4.164387508	-5.827810320	6.632785733
	H165	-5.169256039	-3.285576819	7.330742152
	H166	0.038829050	-4.914148917	2.480143275
	H167	0.093945174	-3.530317408	1.361310683
	H168	1.603467844	-4.314797227	1.871298488
	H169	-0.327487093	-2.763862662	3.697066279
	H170	-3.321962815	-7.516878131	7.954703028
	H171	-0.742220409	-0.068064815	5.429170643
	H172	-0.547815789	-3.230798651	8.622429351
	H173	-0.382287230	-0.006539742	9.775387667
	H174	0.854405148	-1.247664873	2.173432768
	H175	2.426416041	-1.949053083	2.586093674
	H176	9.844180714	-0.378845768	3.395542336
	H177	-1.550485851	1.166303919	6.416530862
	H178	-1.290964703	1.237286439	8.884538746
	H179	0.218495821	1.215646019	6.211643587
	H180	0.487370277	1.215838686	8.816944341
	H181	-1.373503510	-0.964003368	7.695016728
	H182	1.642087544	-0.607769144	7.278814406
	H183	-0.341093490	-2.355220015	6.022176146
	H184	1.511479100	-1.026447606	3.804269252
	H185	1.865985753	4.069834808	1.937940961
	H186	1.445396469	4.329114699	3.660900691
	H187	3.690924338	4.487226475	3.519352382
	H188	4.512251033	2.997915008	5.490108223
	H189	5.575118443	3.318052677	2.122191637
	H190	7.020643078	1.639389147	1.019023898
	H191	7.188663543	-0.625375604	4.693915848
	H192	-0.342308223	-4.732524351	5.396028191
	H193	-0.023878244	-7.890936415	4.749522826
	H194	0.739352054	-7.796610140	6.350744748
	H195	-1.836990981	-6.557660184	5.559688184
	H196	-2.630397541	-6.313965282	9.070001180
	H197	5.702449490	1.032868400	5.782610215
	H198	2.351244823	-4.107089958	4.302107330
	H199	0.264390849	-3.050423880	11.283559312
	H200	-0.143052467	-4.562818612	10.433888782
	H201	2.474131718	-4.363486068	9.598428243
	H202	11.038077796	-8.316213481	-0.350323666
	H203	-2.657986588	-4.266662373	-5.324434667
	H204	0.276399042	-8.592272786	-2.274295444
	H205	-4.102675443	-3.052257988	-9.428936494
	H206	-2.661313445	-2.110994515	-9.902753330
	H207	-4.551465315	-1.619114574	-7.532648765
	H208	-4.558503560	4.353528721	-8.235669215
	H209	-5.106936333	-0.673666895	-9.836984206
	H210	-3.788210074	-1.208682353	-2.273507119
	H211	-4.430675857	-4.181510644	-1.903478357
	H212	-3.603081705	1.919694774	-7.834497217
	H213	-6.394497254	2.737136905	-7.267596519

	H214	-2.636339329	0.898420828	-9.474427300
	H215	-2.003857122	-4.645598876	-3.729462892
	H216	-2.986787245	-0.807227852	-7.639959710
	H217	-3.051005419	-0.085730082	-10.727187874
	H218	-7.311821280	-2.134397442	-2.627936005
	H219	-3.663899507	-1.926518988	-0.659494283
	H220	-0.805402661	-2.456162903	-8.870730884
	H221	-2.751897056	-3.672072367	-1.841754221
	H222	-2.926724947	1.792076869	4.294404861
	H223	-2.201819269	0.420866584	2.038374665
	H224	-7.119368610	-0.170815634	5.162544534
	H225	-6.792744363	-0.100326653	2.194365160
	H226	-5.979197306	3.374559369	4.836690973
	H227	-6.593748438	7.270051216	8.334068651
	H228	-5.398501357	3.626571442	7.866739561
	H229	-7.653124344	4.507132627	6.477122247
	H230	-4.980014603	5.903903166	5.835409964
	H231	-9.373411272	9.272754868	5.845897850
	H232	-2.574764993	3.916534311	3.017098593
	H233	-4.248355639	3.882509860	2.454698110
	H234	-7.998007761	9.372707011	7.993818820
	H235	-7.307273655	5.207762133	4.894595342
	H236	-5.555178019	1.768140391	2.976486645
	H237	-6.166421470	-1.344765557	4.249821563
	H238	-9.668787633	1.908435353	2.042221849
	H239	-2.377763877	2.414701683	-1.703653403
	H240	-9.981683065	0.190801218	1.725468168
	H241	10.934845571	1.231962237	2.008697386
	H242	9.360610245	1.671557605	1.978330438
	H243	2.164310957	6.219820950	0.563839881
	H244	-0.012708883	5.695294955	2.441827358
	H245	2.628686050	8.054746786	2.417860166
	H246	-3.377946434	4.027093195	0.092122243
	H247	-5.044236531	5.164616542	-0.541850523
	H248	-4.470769714	6.063521865	0.641908108
	H249	-5.704804236	2.849522093	-2.320458863
	H250	-7.483473515	2.813695210	-2.137811626
	H251	0.516163327	12.427724707	1.930730230
	H252	-0.815019231	13.330246982	2.719495792
	H253	0.024342722	10.422886323	0.803505826
	H254	-1.518269905	9.638057959	0.964935541
	H255	-2.581573121	10.677440552	-2.194507431
	H256	-2.696540967	6.206757137	-3.458436440
	H257	-6.352689520	8.714544494	3.426127640
	H258	-1.680135739	7.732697583	4.831982399
	H259	-3.961523782	8.520102791	0.222375660
	H260	1.229539047	5.820608354	-1.980793048
	H261	2.787127354	7.567581373	-1.267370469
	H262	-1.196186655	5.508813887	-1.340626375
	H263	-8.277368697	0.171480886	0.103612862
	H264	3.929268666	10.542987546	-1.687500889
	H265	-6.557557724	0.303113812	-2.158414165
	H266	0.770050823	7.109325212	-3.096183952
	H267	-2.479767362	6.971053345	2.625554436
	H268	-6.919029785	4.130353374	-0.110096652
	H269	3.944499738	10.550027890	0.086562598
	H270	-2.242073581	9.463664862	-3.367389072
	H271	-3.889222142	10.150988186	-0.368804210
	H272	-5.857176908	-2.082819838	-3.443778446

	H273	-6.029853790	-2.684267511	-0.804237649
	H274	-5.517310029	9.570637145	5.592438359
	H275	-5.431541074	1.955154581	-0.019594677
	H276	-0.028876323	8.518966818	-1.195105309
	H277	-1.618508710	7.592576725	-3.530560621
	H278	-0.728660373	5.130364410	-2.990675509
	H279	-3.342713268	6.811390440	-1.182245534
	H280	2.503837415	8.355372840	5.283589029
	H281	0.498320211	9.928182783	3.171807280
	H282	0.921546692	12.589443278	4.325732236
	H283	3.629359691	9.121099164	4.143545551
	H284	-0.910576470	9.988246541	5.354123596
	H285	2.844595304	0.101896627	-10.327550896
	H286	2.925522464	-1.605729903	-9.855258831
	H287	6.490255571	-3.468691533	-7.661027314
	H288	2.864011499	-1.485398764	-12.232658675
	H289	1.366923677	-1.982205924	-11.787208382
	H290	6.004973045	4.791772250	-0.016318045
	H291	6.573354785	7.004928198	2.066212831
	H292	5.181277067	8.578876471	-1.694157811
	H293	4.817843235	5.445663664	1.157014460
	H294	7.577137497	7.819449944	1.065081233
	H295	4.475214930	4.790856795	-6.252186988
	H296	6.482041857	7.036716711	-0.866364832
	H297	5.376978141	-8.045748728	1.532030612
	H298	6.715842671	-8.393046243	-1.203328450
	H299	2.660141582	3.446976918	-6.575619758
	H300	5.200352308	-1.009914503	2.215791856
	H301	-0.654702700	4.263674694	-7.596392470
	H302	-2.136335598	-0.900302906	-4.501778692
	H303	-0.443015431	4.222507920	-5.844836137
	H304	4.439401055	-5.536257886	1.847387158
	H305	1.129288322	2.145021829	-6.410976762
	H306	4.048304056	-5.464821822	0.128064170
	H307	4.647733305	-7.790578346	-0.067883659
	H308	6.428787224	-5.938461797	-0.456231094
	H309	6.838139379	-6.082542209	1.263529711
	H310	-6.088248967	1.542757691	-5.295953491
	H311	5.886128476	-10.436678239	0.830991566
	H312	4.406158525	-12.295363035	-1.118091495
	H313	-0.433475616	0.794255815	-3.809875268
	H314	6.295532850	-12.936436170	1.009971880
	H315	4.672636825	-13.148727152	0.954846826
	H316	5.374008889	-3.490213980	-0.123162002
	H317	-1.029950937	3.178821085	-3.747994646
	H318	-4.413354444	-0.171037129	-5.171762736
	H319	6.409984512	-9.092338724	-5.754870349
	H320	5.218184484	-6.162575417	-6.947805779
	H321	5.662898220	-7.914069796	-3.728159373
	H322	5.427437569	-9.346421918	-6.916484745
	H323	3.608366700	5.328278903	-8.959469729
	H324	5.797086067	-4.856181261	-3.032233957
	H325	1.933664001	-7.333682515	-1.851287954
	H326	1.943983548	0.495073348	-8.236115350
	H327	1.975750979	-8.374651352	-4.157031352
	H328	1.378042545	-4.209229811	-9.317072638
	H329	0.936291819	-3.340350825	-6.966272170
	H330	6.019410900	-12.986666269	-1.316126855
	H331	5.133191945	-1.390645858	0.515260083

	H332	7.875811826	-5.981555193	-2.638651342
	H333	6.737761796	-3.672247180	-2.677932805
	H334	8.745668992	-7.599281733	-1.094014898
	H335	5.258578292	-4.308706646	3.371893237
	H336	1.108281794	-9.832143994	-7.627133418
	H337	2.010121448	-6.548900650	-6.613850142
	H338	3.494807739	-7.174892835	-5.871185758
	H339	0.608711266	-7.456456733	-4.848031669
	H340	0.284611499	-0.061691744	-6.575739457
	H341	-3.324266679	3.938893626	-4.458403182
	H342	-5.756121323	3.236724451	-4.855173230
	H343	1.622716739	-5.573544699	-1.740106663
	H344	7.778694582	-7.416537152	-3.221231494
	H345	6.432730317	-9.219343426	-3.389539025
	H346	4.750037796	-2.719741734	3.811441890
	H347	3.758719143	-5.144145653	-9.563805076
	H348	5.344665351	-4.785283100	-5.858432427
	H349	-0.888125494	-0.350860754	-7.897361024
	H350	3.423941445	6.211155000	-6.097328244
	H351	7.937895099	-1.632281550	2.507545530
	H352	7.919231557	-0.652151884	1.021119705
	H353	7.230322977	-4.561223032	-9.593684465
	H354	6.553602160	-5.990894378	-9.178587370
	H355	9.161716132	-7.899034816	-6.142272775
	H356	7.203605900	-7.023864693	-6.909006672
	H357	10.245256743	-6.110881696	-7.940947788
	H358	9.234867068	-6.493625818	-5.057670522
	H359	10.639551188	-1.293527007	0.568803508
	H360	-1.109977790	11.485269555	8.109408413
	H361	10.355881986	-8.496644916	1.287637572
	H362	9.869604830	-5.793970017	0.964763382
	H363	-3.344621956	11.395127631	6.097281433
	H364	-2.182928941	8.708058532	7.055784302
	H365	-3.805991776	9.316605504	7.428980013
	H366	-5.832976095	7.544527583	1.540657985
	H367	1.827410639	-2.586356433	-1.372631436
	H368	0.631772314	-0.993070688	-0.053029937
	H369	-1.566507708	0.354260073	-0.466123164
	Mo370	2.897342492	-3.370035815	-4.282278812
	N371	-7.415999665	-6.293736353	-1.469025910
	N372	-8.909198081	-4.374909936	1.374330216
	N373	-8.353056958	-5.583988446	3.877998649
	N374	-5.935137152	-6.933453349	3.396818550
	N375	-4.183799620	-4.730290853	2.893156411
	N376	-4.285792067	-3.484306598	5.457311653
	N377	-3.428629538	-5.550833466	7.288054375
	N378	-1.068905329	-6.787677931	6.197286372
	N379	0.621043213	-4.862052896	5.092438273
	N380	0.600127842	-2.170664461	6.376472115
	N381	0.297707297	-2.999507136	9.148699280
	N382	5.421012658	-12.497235751	0.713828411
	N383	6.104963288	-10.022051948	-0.081789590
	N384	5.220323872	-3.887609391	0.809009143
	N385	4.910207370	-3.402949866	3.076968548
	N386	4.952571182	-1.691086845	1.499841753
	N387	8.956264903	-8.000679872	-0.154882977
	N388	-6.404512654	-1.665444307	-2.669018215
	N389	-2.714499775	-4.188108730	-4.304154876
	N390	-6.356840670	0.488775888	-1.162026659

	N391	-8.324366117	0.971029386	0.731218149
	N392	-7.208140838	0.097358794	3.113962251
	N393	-4.935877793	1.593174650	3.765575277
	N394	-3.009990965	3.059201812	0.273456801
	N395	-2.162851830	1.089726862	-0.014143492
	N396	-5.230160859	3.885080319	5.297168282
	N397	-8.465578186	7.335244617	5.579075712
	N398	-7.995268016	8.585579763	7.350483116
	N399	10.011439419	0.940035928	1.679325360
	N400	7.031611847	6.956720368	1.150162014
	N401	4.732382152	8.831442906	-0.817639084
	N402	2.015727166	8.234905533	-1.105860018
	N403	-2.850605543	7.462997966	-1.818257523
	N404	-2.432384047	9.694244798	-2.397980352
	N405	-3.576742275	9.187775937	-0.447761655
	N406	1.304795237	6.510142493	1.058900711
	N407	2.037554836	8.113810955	3.253450614
	N408	0.773210452	10.565923992	3.921745037
	N409	-0.879706333	10.363310860	1.287795990
	N410	-1.380525961	10.848783218	5.625434169
	N411	2.002805821	-1.179420443	-11.774180117
	N412	1.045533868	0.085467736	-8.520604032
	N413	0.321643616	2.570350251	-6.907052579
	N414	2.495553899	4.441317398	-6.780965770
	N415	-3.432631771	0.546590456	-10.020555863
	N416	-4.622349446	1.833626081	-7.764334791
	N417	6.432176062	-4.976106289	-9.104523808
	N418	2.963067075	-3.951281390	-6.573261038
	N419	2.069692738	-4.218540487	-8.572345082
	N420	7.940581831	-6.336756057	-6.745929453
	N421	0.359521473	2.654427700	0.419630616
	N422	0.966429323	2.084771728	-0.348639263
	O423	-6.773351234	-5.088871600	1.047733181
	O424	-7.079626970	-3.768838973	4.412878357
	O425	-5.536102538	-6.949977103	5.646725665
	O426	-2.527037752	-6.018834551	3.767627645
	O427	-2.170077350	-2.744818088	5.063837568
	O428	-2.443485550	-3.864310145	8.469209679
	O429	-0.468339801	-7.351461605	8.339869733
	O430	2.245674723	-6.432559041	4.670624790
	O431	2.847852194	-2.474827500	6.087350993
	O432	2.033908705	-1.586062305	9.525545315
	O433	2.456036420	-4.416896373	11.639738883
	O434	6.222214030	-10.622691050	-2.315671644
	O435	8.114094312	-9.056694330	1.706125248
	O436	11.873348042	-6.110982744	0.765524460
	O437	-5.564323367	-0.588652011	0.700716518
	O438	-4.550922087	-2.856392683	-4.470569815
	O439	-7.423906425	3.004921117	1.250422089
	O440	-6.457386872	4.220391333	-0.982517863
	O441	-9.078400462	0.748857015	4.282436511
	O442	-4.462419602	0.222186093	5.531512598
	O443	-2.981316603	3.961286969	5.696987408
	O444	-4.844523185	5.511852199	8.412777593
	O445	10.734885829	-2.489548756	2.222091059
	O446	4.734799035	3.244391394	4.573457125
	O447	4.415233129	8.304792156	1.375310059
	O448	1.445626047	10.360213709	-0.442959831
	O449	-0.833028238	7.297246396	0.816488779

	O450 0.609561322 6.922858570 4.593406857<br>O451 3.240767862 5.214256831 3.041105619<br>O452 2.415617842 11.043698654 5.447678764<br>O453 -1.485607626 13.104720478 5.295793607<br>O454 -2.591806850 11.449830679 2.344068507<br>O455 -3.053516077 11.692131119 8.697842724<br>O456 -4.879389776 7.354226195 1.633056400<br>O457 0.013841083 -1.450244857 -9.886978085<br>O458 -1.586736659 2.082591671 -8.084951813<br>O459 1.019603199 6.160921299 -7.035846544<br>O460 4.966416192 6.665785555 -8.228519798<br>O461 -6.472603545 0.569584135 -8.197011864<br>O462 -2.883739467 -4.214833996 -7.419764053<br>O463 -1.080184279 -3.178340451 -8.220388173<br>O464 -4.899776575 5.086402272 -6.365339591<br>O465 8.684096853 -4.196782742 -6.738173714<br>O466 11.509952381 -6.275718762 -6.348101447<br>O467 -0.914883837 -5.816793360 -2.407479966<br>O468 -0.496425784 -7.998203492 -2.332860125<br>O469 3.300043940 -9.301553718 -7.227565498<br>O470 1.063334015 -9.003606384 -7.103994569<br>O471 4.211770097 -5.172856113 -3.866019079<br>O472 4.159185064 -7.300033763 -3.123505489<br>O473 1.619089634 -5.039316304 -4.282674203<br>O474 8.386343986 -6.828467760 -2.699198058<br>O475 6.604251767 -4.624165769 -2.504202243<br>O476 -4.208897585 5.556034352 -0.165233450<br>O477 6.384780222 -9.351377523 -6.710894736<br>O478 6.480771210 -8.427374150 -3.985622620<br>S479 3.908625549 5.440299540 -1.098761811<br>S480 1.846576200 3.007470444 -2.975547638<br>S481 1.094500717 -1.868683271 -4.721766965<br>S482 3.769587092 1.638785822 -0.138604485<br>S483 0.252434807 -0.611403686 -1.311329623<br>S484 3.196236135 1.088699821 -6.132426158<br>S485 3.034474312 -3.262787267 -1.842734828<br>S486 5.451482320 2.468852922 -3.172584069<br>S487 4.856603287 -2.118362236 -4.826808889<br>S488 6.075059097 -0.998804602 -1.466752480

TS 9→ 10 35 (S=1/2)

TS 9→ 10 35 (S=1/2)	bm22b2n2x3b36hx35ti_1_53084.71
	C1 -7.754614538 -5.338180902 0.823471238<br>C2 -7.933687399 -6.509640767 -0.147924051<br>C3 -8.793370779 -3.356799413 1.836753570<br>C4 -8.432577386 -3.596354243 3.302126274<br>C5 -8.531272322 -5.088770611 5.232736464<br>C6 -7.039786439 -5.360304350 5.453796415<br>C7 -4.929762355 -6.094096052 4.454613792<br>C8 -4.145666632 -4.780750560 4.392196711<br>C9 -3.965957089 -2.541886033 3.447554034<br>C10 -4.121701126 -1.732373354 4.744145388<br>C11 -5.550142066 -1.066677544 6.590179400<br>C12 -4.810090168 -1.647363304 7.802653210<br>C13 -4.314443211 -3.639451207 9.132897934<br>C14 -2.816482950 -3.969445153 9.120698697<br>C15 -0.907120588 -4.748148175 7.780216867<br>C16 0.086830188 -3.808281458 7.092555712

	C17	0.377680801	-1.696154250	5.831983864
	C18	0.376880101	-0.345790775	6.571803746
	C19	-0.018539382	-1.574962830	4.334023923
	C20	0.743261363	-0.445671721	3.632279945
	C21	0.186834865	-2.911379138	3.611396449
	C22	-0.958316617	1.553613109	7.402474141
	C23	-0.913446358	1.385552612	8.934215894
	C24	-2.217850435	2.312056038	6.919368521
	C25	-2.463100148	3.579521878	7.747281821
	C26	-2.103823687	2.659370260	5.430129816
	C27	-1.792392687	0.291691025	10.916102323
	C28	-0.427758100	0.024394075	11.519380246
	C29	5.812542158	-11.054915330	4.920915017
	C30	6.372555858	-9.845421916	4.172506278
	C31	6.699725309	-7.374024065	4.373963561
	C32	7.877135087	-6.937186291	5.264817276
	C33	5.542488079	-6.360307227	4.422969725
	C34	5.935436202	-4.944352732	3.991530335
	C35	4.691658074	-4.101173436	3.706269057
	C36	4.590326384	-1.601426142	3.750555870
	C37	10.178156608	-6.100185832	5.299743092
	C38	10.456497703	-4.614716028	5.213807749
	C39	-5.144078650	-2.821478995	-1.768377451
	C40	-5.565466110	-1.379657576	-1.440284280
	C41	-3.612495612	-2.924788364	-1.596096684
	C42	-3.029903422	-4.354207451	-1.560993467
	C43	-2.653518513	-4.939149182	-2.921182848
	C44	-6.146770716	0.779127322	-2.451661007
	C45	-7.422838144	1.203263319	-1.720160367
	C46	-6.087636649	1.444520914	-3.842497631
	C47	-9.408315066	0.514416946	-0.480339212
	C48	-9.006707046	0.734864993	0.984714817
	C49	-7.253037723	0.528723965	2.693760654
	C50	-6.120996911	1.528681202	2.917882944
	C51	-4.650778340	3.254437925	1.969390699
	C52	-5.122903982	4.593190015	2.584802590
	C53	-4.033239197	3.613296380	0.610444627
	C54	-3.324588341	2.521431495	-0.123758751
	C55	-2.703009943	1.355040728	0.251418434
	C56	-2.396647531	1.604978600	-1.934807985
	C57	-6.964941847	6.229318354	2.629446726
	C58	-6.942690248	6.471636955	4.136101297
	C59	-8.414164984	6.266327186	2.089916877
	C60	-9.008097656	7.625990311	1.862319996
	C61	-9.004870515	8.758307897	2.653520282
	C62	-10.110006457	9.135259389	0.770377969
	C63	8.874532080	1.552532138	4.174587618
	C64	9.805344822	0.354231262	4.126695062
	C65	7.464033031	1.041771243	3.779379209
	C66	6.409075445	2.121963299	3.842702062
	C67	5.724718741	2.399350662	5.040089251
	C68	6.076446563	2.879928762	2.706202509
	C69	4.741622049	3.393064691	5.107702369
	C70	5.107488689	3.883624018	2.761549584
	C71	4.437220583	4.137383853	3.961649279
	C72	5.618800706	7.133011567	-1.209522955
	C73	4.566983416	8.149793727	-1.671594868
	C74	4.938584019	5.812134830	-0.817658881
	C75	3.589414121	9.349224854	-3.576111731

C76	2.156116383	8.825828091	-3.678178912
C77	0.675705247	6.851873856	-3.596850292
C78	0.028708124	6.795878453	-2.196062136
C79	0.796319056	5.465705844	-4.257098943
C80	-0.490500055	4.651769530	-4.427155827
C81	-1.489131228	5.138718548	-5.494930854
C82	-2.760759107	7.282905981	-5.226256277
C83	0.295271228	6.544639927	0.216345912
C84	0.161786496	7.942831183	0.855519143
C85	1.085388292	5.646172397	1.175381976
C86	1.256174070	10.107723518	1.200732158
C87	0.502476605	11.384371213	0.827736346
C88	-1.120221519	12.460267412	-0.688746713
C89	-2.471216187	12.811172623	-0.028232372
C90	-1.289046772	12.346946099	-2.223847574
C91	-2.131995605	11.136949493	-2.603676547
C92	-4.280459584	12.045805761	1.453624931
C93	-4.260249556	13.101825670	2.555313054
C94	-4.757797186	10.686641614	2.031096245
C95	-4.933609416	9.629699365	0.965073704
C96	-6.131313941	9.542796556	0.238862313
C97	-3.896150233	8.739664086	0.639676615
C98	-6.289265980	8.612683146	-0.791658645
C99	-4.026331878	7.817473206	-0.403631585
C100	-5.221876166	7.775262892	-1.117544465
C101	4.642797851	-4.174465817	-8.628970822
C102	3.200524115	-3.927542696	-8.216561621
C103	1.682471890	-2.401965503	-6.982116388
C104	1.179247239	-1.084762098	-7.582010156
C105	1.515138804	1.347859082	-7.621939176
C106	2.680046609	2.315537085	-7.822369541
C107	4.961762670	2.988781232	-7.317055229
C108	5.639573861	3.054193367	-8.669999928
C109	-2.147011164	-3.844048471	-9.160211701
C110	-3.210438065	-2.759101981	-8.953181830
C111	-1.755984555	-4.460743010	-7.795626793
C112	-1.014508243	-5.798970921	-7.981763951
C113	-0.370013991	-6.243934454	-6.687899055
C114	-3.687591429	-0.427076167	-8.377794774
C115	-3.209080884	0.847642040	-9.047410790
C116	-4.056193454	-0.272150956	-6.879179365
C117	-2.858526266	-0.341661140	-5.963319769
C118	-2.048391947	0.783829557	-5.728229543
C119	-2.482096989	-1.569209106	-5.388366275
C120	-0.874830477	0.669838767	-4.973297553
C121	-1.305936280	-1.684410490	-4.638960267
C122	-0.488942662	-0.565900299	-4.442806459
C123	8.203909825	-6.262789153	-4.014959552
C124	9.331598677	-6.273021668	-2.968596067
C125	6.844398740	-6.601308748	-3.380181708
C126	5.648893238	-6.445234369	-4.269800629
C127	5.396734242	-7.085197119	-5.466802860
C128	3.623722760	-5.892679678	-4.882860333
C129	10.523922474	-7.435397787	-1.199617448
C130	11.885143492	-7.172135507	-1.807308143
C131	0.661458475	-7.011352659	0.283313858
C132	2.096550425	-6.646560898	0.543754201
C133	3.035085753	-6.883610297	-0.709455077
C134	2.927469017	-8.368382259	-1.161893767

	C135	3.942214864	-8.713883992	-2.263128828
	C136	4.073730976	-10.182989035	-2.549085522
	C137	4.494893918	-6.668883476	-0.198024203
	C138	3.757351132	-0.673949465	-2.009758664
	Fe139	4.076372837	-1.996767230	-3.480052518
	Fe140	2.253390167	0.808232824	-1.450151276
	Fe141	5.034004562	0.532673438	-1.057193674
	Fe142	4.213387444	0.560955155	-3.517401787
	Fe143	4.021109909	2.787076918	-1.957479429
	Fe144	4.921474675	-1.972152185	-1.016912252
	Fe145	2.425408542	-2.103176943	-1.500728611
	H146	-6.172562296	-7.447805127	0.270571659
	H147	-6.122176483	-6.558519825	-1.096841802
	H148	-9.684710440	-4.723945621	0.455561316
	H149	-8.477351046	-6.157800029	-1.038762042
	H150	-8.601834731	-7.235157671	0.347754355
	H151	-8.037574077	-2.660477996	1.446889598
	H152	-9.413680065	-5.398000115	3.293969894
	H153	-9.774196421	-2.862594207	1.798296818
	H154	-9.101980616	-5.980108619	5.522179103
	H155	-6.831940975	-5.961503470	3.501298096
	H156	-8.809774488	-4.270017620	5.909279756
	H157	-2.889027702	-2.637207875	3.261740150
	H158	-5.359125275	-4.081565790	2.870883799
	H159	-4.632234747	-6.734632248	3.612979521
	H160	-5.216114640	-0.028153061	6.471154998
	H161	-6.102065165	-2.308386222	4.920132566
	H162	-4.642366437	-6.591314185	5.388733362
	H163	-4.410639478	-1.983591897	2.611440548
	H164	-5.405129226	-3.565766064	7.296441681
	H165	-6.628718876	-1.066274296	6.800405045
	H166	-0.396530713	-3.717918532	4.078225123
	H167	-0.125646130	-2.827311755	2.558877068
	H168	1.248213942	-3.206277593	3.617074713
	H169	-1.093031867	-1.329476897	4.307288861
	H170	-4.855318333	-4.584014760	9.286691080
	H171	-1.941347134	1.768843650	4.807103065
	H172	-2.436587803	-0.041534394	8.913164369
	H173	-2.619393747	3.357984948	8.813046185
	H174	0.390812955	-0.344642891	2.594488703
	H175	1.823602979	-0.645934388	3.591782457
	H176	8.826816598	1.890721740	5.227842854
	H177	-3.028317064	3.146202879	5.087779228
	H178	-3.357874015	4.099089444	7.373913646
	H179	-1.265876856	3.356480068	5.262890471
	H180	-1.608670644	4.269896214	7.664188850
	H181	-3.083635963	1.639059578	7.057665485
	H182	-0.065552695	2.150389016	7.164422593
	H183	-1.663999959	-0.139279258	6.280045638
	H184	0.595000102	0.527795808	4.124014602
	H185	1.250923324	4.669741748	0.690142109
	H186	0.453094324	5.492774912	2.065062477
	H187	2.675162790	5.814773316	2.313890084
	H188	3.054736471	5.187316785	4.831037816
	H189	4.843930151	4.448622468	1.867994032
	H190	6.559184916	2.665953812	1.749793832
	H191	5.957548646	1.821478558	5.939185872
	H192	-1.443446258	-2.557674282	6.575797756
	H193	-0.891153496	-5.696403366	7.221600726

	H194	-0.517222365	-4.944304877	8.787633736
	H195	-2.879109815	-4.366152009	7.085708646
	H196	-4.484728550	-2.998240843	10.006711701
	H197	4.210777676	3.576842195	6.045363149
	H198	1.410483491	-2.060657131	5.901429173
	H199	-2.240024270	1.165419432	11.416648635
	H200	-2.423406811	-0.584698282	11.132885797
	H201	0.306926941	-0.457903845	10.833129391
	H202	11.056075019	-6.658740024	4.940328417
	H203	-1.245566612	-6.104246034	-3.844137606
	H204	1.024582326	-8.721801683	1.096825506
	H205	-1.732161360	-6.571244014	-8.292948295
	H206	-0.251768094	-5.700799333	-8.767409524
	H207	-2.651737537	-4.632935640	-7.179833235
	H208	-2.669602333	0.680174117	-10.009165059
	H209	-2.675927279	-4.619075391	-9.735360636
	H210	-3.129327807	-2.340889895	-2.395916886
	H211	-3.755946694	-5.046459292	-1.100115695
	H212	-1.752518123	-1.343673261	-8.528543969
	H213	-4.616045404	-0.694606481	-8.920911033
	H214	-0.406378980	-2.757315521	-9.466286619
	H215	-0.937955213	-5.805039120	-2.130391730
	H216	-1.129980770	-3.738115071	-7.250307363
	H217	-0.488206293	-4.160310592	-10.324389898
	H218	-6.464807267	-3.690737535	-3.097581202
	H219	-3.360756137	-2.436963884	-0.643320297
	H220	1.326843821	-5.477580465	-7.335736860
	H221	-2.148515184	-4.364142900	-0.904304058
	H222	-3.889467039	2.857924445	2.654589116
	H223	-2.595173495	0.881531181	1.220436130
	H224	-8.101780871	0.861293399	3.308603366
	H225	-7.110853629	-0.069447693	0.648658474
	H226	-7.022794145	4.216000094	1.902707947
	H227	-8.560019044	8.974607388	3.618998813
	H228	-7.131189894	5.551862247	4.743151601
	H229	-9.056844506	5.656903484	2.748895820
	H230	-6.354534373	7.002698045	2.139593343
	H231	-10.695540179	9.669634749	0.025590766
	H232	-3.322453828	4.435316708	0.790492678
	H233	-4.822390206	4.023996152	-0.039520352
	H234	-9.887344494	10.662824553	2.237478279
	H235	-8.411313827	5.761324286	1.111267546
	H236	-6.145252688	2.093483854	0.931288834
	H237	-6.928101761	-0.440900636	3.096939349
	H238	-9.941570079	1.418250626	-0.801161615
	H239	-2.059321538	1.432303298	-2.953757872
	H240	-10.113960778	-0.325311782	-0.522428177
	H241	10.150821390	3.060625869	3.630397384
	H242	8.618669802	3.279275882	3.103971921
	H243	1.802551867	6.199896877	-1.304650541
	H244	-0.726858143	6.146703806	0.151811091
	H245	1.782314720	8.620432177	-0.204444713
	H246	-3.472895499	3.443019842	-2.079382393
	H247	-4.946135996	4.063645901	-3.425729159
	H248	-4.670719994	5.374325880	-2.560589086
	H249	-5.121416747	1.217519696	-4.313038100
	H250	-6.893751145	1.047535214	-4.481234016
	H251	-0.289268110	12.299218641	-2.678769905
	H252	-1.795082866	13.256933366	-2.570187426

	H253	-0.437381888	9.969307084	-3.053216661
	H254	-1.951369970	9.125095614	-2.939335104
	H255	-2.266519377	8.908211723	-6.363646398
	H256	-1.978562742	4.273923264	-5.966452356
	H257	-7.232266657	8.551276119	-1.340689441
	H258	-2.959173019	8.747451653	1.203891944
	H259	-4.137975983	7.560674112	-3.703963990
	H260	1.499764973	4.851891765	-3.672501959
	H261	2.796071481	6.895752870	-3.332180822
	H262	-1.008716184	4.533665383	-3.462686228
	H263	-8.099796313	-0.712235380	-1.666311459
	H264	3.917212979	9.624582660	-4.587487596
	H265	-5.905246794	-1.196685214	-3.405573734
	H266	1.260200712	5.617012681	-5.246236524
	H267	-3.185018149	7.181327823	-0.687063296
	H268	-6.837210584	3.042321914	-2.999230433
	H269	3.543760664	10.266931037	-2.974100088
	H270	-1.642649130	7.411656843	-6.945826467
	H271	-3.971627698	8.889028077	-4.811284289
	H272	-4.864653968	-3.799332683	-3.556150109
	H273	-5.611694339	-3.444480406	-0.982595861
	H274	-6.964573610	10.206471155	0.482321813
	H275	-5.338711389	1.208950812	-1.837452544
	H276	0.013907026	7.498139156	-4.188880090
	H277	-0.954127824	5.661078120	-6.297659990
	H278	-0.167684310	3.642320153	-4.719588534
	H279	-3.177716229	5.545823108	-4.264432284
	H280	1.016889819	9.904278421	2.255894971
	H281	-0.532727271	10.395035712	-0.643935574
	H282	-0.466802266	13.320274433	-0.480405508
	H283	2.325930381	10.350553645	1.140255942
	H284	-2.417731929	11.065303492	1.026343025
	H285	5.152177129	-3.213798763	-8.793059019
	H286	5.127138853	-4.647695501	-7.748662666
	H287	8.171617782	-5.230266835	-4.390981784
	H288	5.647290804	-5.336546497	-9.968507068
	H289	4.091822323	-5.796052300	-9.728272935
	H290	5.724594248	5.097720817	-0.543885402
	H291	5.740664822	7.938540077	0.659818286
	H292	5.195072981	7.936856126	-3.618254040
	H293	4.287790738	5.987506575	0.050770883
	H294	6.923919592	8.456815685	-0.342087125
	H295	5.721054596	2.686479785	-6.578061698
	H296	6.319081234	6.936764424	-2.036454858
	H297	5.125361005	-6.346016641	5.444179121
	H298	7.036289095	-7.489113003	3.335812791
	H299	4.005353869	1.200874135	-6.653105034
	H300	4.582137172	0.412102545	3.440044578
	H301	0.996403925	1.251009886	-8.584005058
	H302	-1.013268900	-2.648061098	-4.216694379
	H303	0.813529842	1.852161466	-6.935337124
	H304	4.055790252	-4.016364749	4.597208367
	H305	2.517349241	-0.124557409	-6.376221322
	H306	4.083065207	-4.594081072	2.931109799
	H307	4.755506564	-6.745624478	3.754448533
	H308	6.539246439	-4.977379971	3.072288402
	H309	6.545290259	-4.454162538	4.767437064
	H310	-4.761920294	-1.077748674	-6.630819371
	H311	5.837036205	-8.769925916	5.769171013

	H312	4.897159103	-11.347444432	4.383156852
	H313	0.448908970	-0.650357497	-3.890991941
	H314	6.280981618	-10.981815335	6.925542725
	H315	4.718702934	-11.379767047	6.633748775
	H316	5.369839662	-2.694966162	2.255716518
	H317	-0.225616532	1.531449408	-4.801563200
	H318	-3.114351095	-2.447689520	-5.535594400
	H319	7.808280552	-9.759974545	-0.598767095
	H320	6.873698428	-7.648307866	-3.027111285
	H321	6.585026260	-8.042724657	0.661465932
	H322	7.131427794	-10.513155997	-1.763281337
	H323	5.422937377	2.198753445	-9.353965905
	H324	6.468523106	-4.932463951	0.208154540
	H325	2.500003075	-7.208023301	1.399847356
	H326	3.771821271	-2.218156055	-7.229485604
	H327	3.106655173	-9.011576259	-0.285624136
	H328	3.611195711	-7.048296306	-6.652297601
	H329	2.627902461	-5.469205959	-4.908975583
	H330	6.535597578	-11.874963666	4.776223752
	H331	4.930924873	-0.542801395	2.021952338
	H332	8.444300833	-5.615377384	1.387994715
	H333	7.236161604	-3.585205329	0.303161334
	H334	8.969542238	-6.476924092	3.553963157
	H335	4.448442608	-2.259668384	5.697736963
	H336	3.087962815	-11.707940074	-3.080948763
	H337	3.645783806	-8.204481205	-3.192354653
	H338	4.943745617	-8.363213741	-1.992551795
	H339	1.908334777	-8.554796907	-1.530530342
	H340	1.777544424	-2.324785412	-5.887159660
	H341	-2.330762517	1.743302680	-6.169784936
	H342	-4.583785375	0.689131392	-6.775864807
	H343	2.139789101	-5.570995706	0.759848972
	H344	8.513514814	-7.165928263	1.363722423
	H345	7.299283124	-9.051496753	1.601430431
	H346	3.824520252	-0.676600409	5.420727494
	H347	6.012437663	-7.749822023	-6.060247916
	H348	6.698433253	-5.979120444	-2.488599916
	H349	0.959292651	-3.182607185	-7.226703185
	H350	4.616310851	3.997414328	-7.045359807
	H351	7.208439218	0.225709297	4.472773809
	H352	7.506757458	0.617971276	2.763154294
	H353	9.389352814	-6.838828339	-5.587161937
	H354	8.676220892	-8.098272997	-4.826228726
	H355	10.545060530	-8.454987720	-0.782219976
	H356	8.801214275	-8.160287732	-2.221471062
	H357	11.963816031	-7.392431945	-2.899066471
	H358	10.347638992	-6.741234554	-0.362789152
	H359	10.245793570	0.156048190	3.118949009
	H360	-3.245014174	13.371308820	2.931550166
	H361	10.033590973	-6.341796053	6.367821112
	H362	9.559903933	-3.971816569	5.037302343
	H363	-4.996924414	12.385530435	0.688639666
	H364	-4.036998128	10.347525547	2.791827129
	H365	-5.710291768	10.869492646	2.548217094
	H366	-6.224945780	6.926249609	-2.554458052
	H367	1.888687916	-2.870466505	-0.200704434
	H368	0.537758799	-0.869659167	0.280134194
	H369	-1.463325196	0.020771675	-0.943871148
	Mo370	3.921482873	-4.327329065	-2.080377241

	N371	-6.717078105	-7.190823609	-0.556782870
	N372	-8.857192026	-4.563297580	1.025088641
	N373	-8.870120083	-4.751436569	3.859871485
	N374	-6.371629039	-5.911279355	4.409791954
	N375	-4.596450089	-3.852414615	3.509985984
	N376	-5.326305688	-1.805920935	5.358183319
	N377	-4.851047014	-2.997879238	7.942643717
	N378	-2.274795388	-4.274804978	7.907258748
	N379	-0.433719177	-2.681757335	6.538740684
	N380	-0.832391237	0.267255685	6.713957744
	N381	-1.735130300	0.448003257	9.473089844
	N382	5.481311608	-10.776535119	6.322431651
	N383	6.233998452	-8.677774009	4.827853736
	N384	5.011791309	-2.755782479	3.214365398
	N385	4.157296793	-1.553219930	5.030241442
	N386	4.532678510	-0.499171671	2.986858388
	N387	8.975270948	-6.503934577	4.596521033
	N388	-5.577595685	-3.184192011	-3.122887469
	N389	-1.525328073	-5.674388451	-2.956281286
	N390	-5.944125010	-0.653252516	-2.527381876
	N391	-8.302275181	0.242409158	-1.379473652
	N392	-7.731565996	0.386851446	1.329879539
	N393	-5.716026638	2.268912963	1.837999511
	N394	-3.119238057	2.642886138	-1.491926479
	N395	-2.153116620	0.805463419	-0.891219568
	N396	-6.420484183	4.907007147	2.344216318
	N397	-9.704320385	7.874067661	0.689482109
	N398	-9.707997471	9.706928887	1.940815886
	N399	9.345886744	2.570363269	3.233648689
	N400	6.397009630	7.624587180	-0.062318034
	N401	4.553576207	8.431676982	-3.004499995
	N402	1.990198158	7.488430326	-3.587852539
	N403	-2.559665588	5.992195813	-4.963872700
	N404	-2.060021163	7.930930143	-6.180923452
	N405	-3.635262885	7.989533577	-4.482445564
	N406	0.848793004	6.562488776	-1.136330985
	N407	1.011179566	8.911162318	0.405046931
	N408	-0.460394557	11.279311145	-0.136048690
	N409	-1.427948142	9.984845110	-2.780213467
	N410	-2.968733854	11.900262848	0.843575826
	N411	4.696741109	-4.978641516	-9.852279503
	N412	2.973016141	-2.784238271	-7.542697752
	N413	1.826197071	0.013703356	-7.139973714
	N414	3.878147042	2.021920868	-7.260770351
	N415	-1.041222672	-3.364835307	-9.998244196
	N416	-2.756290133	-1.507320673	-8.660856466
	N417	8.509517325	-7.143157841	-5.159499839
	N418	4.525003408	-5.696489210	-3.924150824
	N419	4.116996408	-6.724996127	-5.832182683
	N420	9.464851510	-7.384919531	-2.193193696
	N421	0.191487328	2.742754032	-0.589946444
	N422	0.962460740	1.987488979	-0.934018543
	O423	-6.688857800	-5.129186501	1.432248980
	O424	-7.780743598	-2.750598789	3.936359055
	O425	-6.505600329	-5.105958096	6.545589554
	O426	-3.157603267	-4.579125012	5.115277247
	O427	-3.190516740	-1.041521754	5.189896997
	O428	-4.243048885	-0.903825114	8.621848784
	O429	-2.181585232	-3.985728467	10.183643942

	O430	1.286565611	-4.116193162	7.050149951
	O431	1.432195950	0.146584550	7.000179988
	O432	-0.158933273	2.073266391	9.641745490
	O433	-0.156813669	0.246086832	12.689805592
	O434	6.876321405	-9.999828381	3.039596032
	O435	7.786150403	-6.989227045	6.504063836
	O436	11.570067585	-4.132654841	5.341891580
	O437	-5.560173285	-0.919710991	-0.280180464
	O438	-3.360373600	-4.759801103	-3.938290858
	O439	-7.584575520	2.410032956	-1.431980254
	O440	-6.200881859	2.873498646	-3.739565819
	O441	-9.838589402	1.198067872	1.773180559
	O442	-5.622126459	1.654929409	4.038572978
	O443	-4.331668717	5.347347968	3.158809439
	O444	-6.774300740	7.558547381	4.659193635
	O445	10.055089869	-0.358071638	5.088176122
	O446	3.472478019	5.125913841	3.952177296
	O447	3.773160350	8.693439797	-0.881723485
	O448	1.226808837	9.637202712	-3.868076373
	O449	-1.195556300	6.987601520	-2.077590503
	O450	-0.658238951	8.128735784	1.765813093
	O451	2.320173809	6.275493033	1.525501040
	O452	0.767747849	12.443879915	1.409910082
	O453	-3.046826415	13.873533830	-0.301909112
	O454	-3.372208876	11.178072882	-2.634523508
	O455	-5.271496300	13.601402607	3.019711228
	O456	-5.316997402	6.878116659	-2.197939095
	O457	2.311281121	-4.779467267	-8.474025429
	O458	0.252338702	-1.057634382	-8.421704330
	O459	2.482729756	3.365176845	-8.460055117
	O460	6.391085190	3.958798211	-8.998110669
	O461	-4.422732920	-3.021911691	-9.024081843
	O462	-0.983767063	-6.803924251	-5.784789126
	O463	0.936480165	-5.951424743	-6.534618093
	O464	-3.409568159	1.976280013	-8.622278681
	O465	10.141799342	-5.332716287	-2.871233101
	O466	12.856262232	-6.811190206	-1.158627579
	O467	-0.173589412	-6.295181840	-0.252030411
	O468	0.270738686	-8.266490644	0.675769662
	O469	5.116704497	-10.829786924	-2.493527244
	O470	2.896517003	-10.761592767	-2.907101789
	O471	5.149469125	-5.717175459	-0.776527852
	O472	4.967286155	-7.422279909	0.685763010
	O473	2.710689997	-6.009613039	-1.728136164
	O474	8.974875420	-6.372092468	1.744799444
	O475	7.136312096	-4.445253788	0.756608370
	O476	-4.225159824	4.645876008	-3.059270851
	O477	8.013888709	-10.339187061	-1.376142027
	O478	7.456898022	-8.517688984	0.779722169
	S479	3.899993205	5.091731343	-2.172089705
	S480	2.417838322	1.972428908	-3.440040404
	S481	2.216818966	-3.284965255	-3.376185739
	S482	3.645727268	1.878702948	0.048710861
	S483	0.567790804	-0.914018647	-1.085820187
	S484	4.476119661	-0.766695507	-5.312505929
	S485	3.479280418	-3.322156751	0.085147277
	S486	5.967315287	1.780999277	-2.655785954
	S487	5.878505141	-3.109683898	-2.683604087
	S488	6.276513916	-0.796529378	0.253586797

10 35 (S=1/2)

**10 35 (S=1/2)**	bm22b2n2x3b36hx35ti.car_4
Fe(139) -2.262<br>Fe(140) -1.386<br>Fe(141) -2.587<br>Fe(142) 2.807<br>Fe(143) 2.884<br>Fe(144) 2.349<br>Fe(145) -1.262	C1 -7.772029204 -5.357032683 0.827500492<br>C2 -7.958649759 -6.529093599 -0.141908088<br>C3 -8.786113520 -3.357864858 1.829701918<br>C4 -8.430313075 -3.597668277 3.295538022<br>C5 -8.543549900 -5.083007666 5.230457140<br>C6 -7.054390435 -5.361191722 5.461786597<br>C7 -4.941882482 -6.112949467 4.478566977<br>C8 -4.153213129 -4.802169143 4.408399145<br>C9 -3.978933480 -2.563631340 3.456463403<br>C10 -4.131764970 -1.748588138 4.749945707<br>C11 -5.559802943 -1.073116507 6.593231636<br>C12 -4.818600901 -1.647948165 7.806865360<br>C13 -4.315602013 -3.634699610 9.142887030<br>C14 -2.818323042 -3.968035001 9.128732244<br>C15 -0.909990936 -4.757035948 7.795216165<br>C16 0.088602875 -3.813263269 7.119974691<br>C17 0.377028650 -1.701772236 5.853955248<br>C18 0.367034211 -0.344064493 6.580674838<br>C19 -0.023616030 -1.605449791 4.356097442<br>C20 0.724266445 -0.478811227 3.638512904<br>C21 0.193670207 -2.950703985 3.654071343<br>C22 -0.981772307 1.544612911 7.410378701<br>C23 -0.936808551 1.375276096 8.942079421<br>C24 -2.245932409 2.295902388 6.930005545<br>C25 -2.498719727 3.555878258 7.767897574<br>C26 -2.133827456 2.652429466 5.442725650<br>C27 -1.828800188 0.296080094 10.925098838<br>C28 -0.467709368 0.070434821 11.553463703<br>C29 5.826677372 -11.041294340 4.927491599<br>C30 6.394216067 -9.833283915 4.179851593<br>C31 6.705719646 -7.359499924 4.371803819<br>C32 7.879945401 -6.915670359 5.263404508<br>C33 5.539524728 -6.354745716 4.408518973<br>C34 5.918959831 -4.933562501 3.979655264<br>C35 4.663071766 -4.105120555 3.696006133<br>C36 4.574453314 -1.605268075 3.767306601<br>C37 10.183967169 -6.083197704 5.289230586<br>C38 10.492188883 -4.606067411 5.177374765<br>C39 -5.165055202 -2.838703797 -1.781267698<br>C40 -5.582663861 -1.398047132 -1.445373506<br>C41 -3.633920788 -2.939372101 -1.600123420<br>C42 -3.049121993 -4.367058775 -1.566009539<br>C43 -2.654152859 -4.938930943 -2.926754196<br>C44 -6.149015918 0.768879700 -2.449947628<br>C45 -7.421279934 1.188073473 -1.708154580<br>C46 -6.099847803 1.438097496 -3.839264278<br>C47 -9.411330292 0.490894279 -0.480174777<br>C48 -9.013965499 0.736672578 0.982056968<br>C49 -7.262063275 0.542178401 2.698258593<br>C50 -6.128519405 1.543885457 2.912398328<br>C51 -4.677139700 3.279213978 1.949830849<br>C52 -5.131963491 4.621986984 2.567232823<br>C53 -4.067347632 3.630476473 0.584944356<br>C54 -3.324378584 2.541952171 -0.120397269<br>C55 -2.679283146 1.401950902 0.293067326

	C56	-2.329513684	1.611618072	-1.889582298
	C57	-6.969549202	6.259559771	2.662567504
	C58	-6.902062551	6.520286087	4.165939349
	C59	-8.433895176	6.300712521	2.164152397
	C60	-9.019805823	7.663721606	1.936428804
	C61	-9.002674260	8.798987801	2.722807958
	C62	-10.121700498	9.175826583	0.847530325
	C63	8.899643618	1.547698378	4.149175666
	C64	9.825242463	0.347617377	4.077484955
	C65	7.489294629	1.052153672	3.733276550
	C66	6.437175345	2.133395649	3.820222077
	C67	5.762544216	2.396709753	5.025859219
	C68	6.096132680	2.905338581	2.696658557
	C69	4.776612552	3.386020364	5.111326886
	C70	5.125052301	3.905421548	2.769806975
	C71	4.459526732	4.141798862	3.975794307
	C72	5.643795612	7.179042519	-1.197974775
	C73	4.588198486	8.183311599	-1.678099311
	C74	4.971959155	5.845245322	-0.835298734
	C75	3.612818632	9.356463214	-3.597648573
	C76	2.177429236	8.833548466	-3.666960942
	C77	0.694226746	6.864699435	-3.589043877
	C78	0.049794551	6.806441581	-2.187783558
	C79	0.802747864	5.480024401	-4.257303924
	C80	-0.486946883	4.665871855	-4.411409070
	C81	-1.492792686	5.133643781	-5.481033468
	C82	-2.749914092	7.284234572	-5.201585914
	C83	0.321894899	6.574988623	0.228388525
	C84	0.178968475	7.971426870	0.867078937
	C85	1.116442396	5.682793412	1.190157438
	C86	1.239834170	10.152966007	1.200935248
	C87	0.490569140	11.424754062	0.802454064
	C88	-1.157707603	12.461541693	-0.716183910
	C89	-2.515271156	12.809663443	-0.065897850
	C90	-1.321419219	12.323488565	-2.250047440
	C91	-2.146132055	11.095062318	-2.610308174
	C92	-4.304690023	12.060852782	1.452106898
	C93	-4.302553601	13.119457618	2.553601796
	C94	-4.776075842	10.696469195	2.022408725
	C95	-4.950314168	9.640780860	0.954616449
	C96	-6.148252508	9.552913904	0.228641508
	C97	-3.914086506	8.747731214	0.632299590
	C98	-6.307465637	8.619788401	-0.798753089
	C99	-4.045386465	7.823975312	-0.409302960
	C100	-5.241321126	7.780029500	-1.122299996
	C101	4.644338246	-4.184135381	-8.602180584
	C102	3.199009918	-3.933132285	-8.202568481
	C103	1.673074469	-2.401385771	-6.986616480
	C104	1.180815907	-1.077893131	-7.582800425
	C105	1.554255072	1.351012120	-7.614864793
	C106	2.748661460	2.276704774	-7.838546537
	C107	5.023483249	2.933246862	-7.327949024
	C108	5.539258047	3.129596351	-8.737882229
	C109	-2.117789613	-3.825517703	-9.197337985
	C110	-3.188906013	-2.748208950	-8.991528259
	C111	-1.744104079	-4.449911613	-7.831033612
	C112	-1.003770364	-5.789025490	-8.010083935
	C113	-0.381529391	-6.236589205	-6.706231263
	C114	-3.683551014	-0.424223442	-8.401216100

	C115	-3.210536357	0.863098784	-9.049063410
	C116	-4.048225432	-0.297357081	-6.898319473
	C117	-2.850075950	-0.363870074	-5.981127549
	C118	-2.053534743	0.768118137	-5.731527970
	C119	-2.464093676	-1.590821464	-5.411348265
	C120	-0.888079793	0.664235984	-4.962421812
	C121	-1.297384665	-1.696377604	-4.645809274
	C122	-0.496957015	-0.569647696	-4.429022662
	C123	8.233432025	-6.315960088	-4.044820743
	C124	9.357826899	-6.296781484	-2.997779589
	C125	6.875256217	-6.630623560	-3.397659246
	C126	5.683546040	-6.493987038	-4.291949665
	C127	5.442264431	-7.124197116	-5.497035619
	C128	3.651065098	-5.968932255	-4.892874119
	C129	10.516928354	-7.404420881	-1.174178124
	C130	11.888004484	-7.178943562	-1.775540305
	C131	0.653708585	-6.992320564	0.256083193
	C132	2.092047037	-6.664618555	0.540480338
	C133	3.045707649	-6.928049926	-0.696292816
	C134	2.946033320	-8.417633224	-1.129187777
	C135	3.961734039	-8.756731657	-2.230156217
	C136	4.093931952	-10.221991051	-2.525076935
	C137	4.500917327	-6.698631211	-0.178144454
	C138	3.730533070	-0.664408778	-1.997024638
	Fe139	4.038481494	-2.016286507	-3.431273897
	Fe140	2.279431512	0.941032330	-1.474931676
	Fe141	5.025580978	0.498926617	-1.053165985
	Fe142	4.198547273	0.519807538	-3.500365697
	Fe143	4.119200615	2.811977862	-1.976589785
	Fe144	4.796707806	-2.011517549	-0.986096764
	Fe145	2.241282898	-2.365931996	-1.614129742
	H146	-6.189218838	-7.455872277	0.266193418
	H147	-6.158196189	-6.580728362	-1.110101554
	H148	-9.683045849	-4.706655934	0.434910641
	H149	-8.508497400	-6.179958436	-1.029781538
	H150	-8.623111051	-7.253238059	0.360693078
	H151	-8.023022165	-2.669298677	1.440295779
	H152	-9.419265199	-5.395468258	3.289582228
	H153	-9.761154790	-2.852844145	1.789730663
	H154	-9.118782206	-5.972283675	5.519167474
	H155	-6.841344008	-5.985733530	3.516935341
	H156	-8.821567131	-4.260658356	5.903044566
	H157	-2.902534094	-2.657067304	3.264863255
	H158	-5.374097173	-4.105911584	2.893744124
	H159	-4.644206417	-6.757892307	3.640382069
	H160	-5.228353958	-0.034068155	6.469471449
	H161	-6.114326858	-2.317166870	4.926621613
	H162	-4.657955477	-6.605606436	5.416672395
	H163	-4.428367273	-2.008994381	2.620463841
	H164	-5.410111681	-3.568169288	7.307769989
	H165	-6.638690271	-1.073185722	6.803607988
	H166	-0.378433188	-3.755755326	4.137145702
	H167	-0.123611492	-2.890852303	2.601772141
	H168	1.257934524	-3.234776870	3.658857706
	H169	-1.100158066	-1.370624924	4.329255675
	H170	-4.860725058	-4.576527507	9.303309437
	H171	-1.963521554	1.765740212	4.816400159
	H172	-2.459470574	-0.050788560	8.920316251
	H173	-2.678353348	3.319273106	8.827172142

	H174	0.363850260	-0.397606570	2.602693089
	H175	1.806210373	-0.668730575	3.596983437
	H176	8.852104259	1.865650753	5.208382213
	H177	-3.063616790	3.131105102	5.103564990
	H178	-3.379540590	4.091601781	7.384640768
	H179	-1.302752443	3.357446538	5.278318173
	H180	-1.635663758	4.239182774	7.715589660
	H181	-3.107516714	1.616488167	7.064182746
	H182	-0.093058626	2.148478060	7.173965213
	H183	-1.674967830	-0.153833183	6.286550381
	H184	0.566438297	0.498986544	4.117263701
	H185	1.287867786	4.707199327	0.706164993
	H186	0.484742743	5.524555674	2.080468620
	H187	2.700693297	5.858075274	2.333780291
	H188	3.071589049	5.168863384	4.861862378
	H189	4.855434665	4.480875216	1.884564148
	H190	6.573571612	2.702201843	1.736064227
	H191	6.003359501	1.809952481	5.916175140
	H192	-1.441597729	-2.562724834	6.606567439
	H193	-0.892197325	-5.700105786	7.229389288
	H194	-0.527089904	-4.961535131	8.803282291
	H195	-2.880326591	-4.375572046	7.094032679
	H196	-4.480672089	-2.987774795	10.013640865
	H197	4.252661203	3.556509006	6.055059255
	H198	1.411596425	-2.062085808	5.926855298
	H199	-2.305821236	1.165049633	11.408220407
	H200	-2.441022350	-0.593325595	11.142341880
	H201	0.289383180	-0.411423682	10.892085030
	H202	11.051416159	-6.663909418	4.940078939
	H203	-1.219531940	-6.069275378	-3.848661013
	H204	0.970977568	-8.736512547	1.018693643
	H205	-1.717727341	-6.559154637	-8.334542322
	H206	-0.228740455	-5.691548533	-8.783169490
	H207	-2.648794023	-4.621853888	-7.227237644
	H208	-2.673472427	0.711634818	-10.014818483
	H209	-2.634635619	-4.599371483	-9.785745260
	H210	-3.149870615	-2.351175490	-2.396540030
	H211	-3.781769183	-5.063677605	-1.123092633
	H212	-1.741853385	-1.324335871	-8.563668367
	H213	-4.610616115	-0.689217644	-8.947172967
	H214	-0.376305835	-2.731095792	-9.471840884
	H215	-0.913192928	-5.752915275	-2.136776649
	H216	-1.122191855	-3.731509650	-7.274808297
	H217	-0.443393011	-4.126928807	-10.342828501
	H218	-6.477554898	-3.694098694	-3.126700976
	H219	-3.388693570	-2.451928959	-0.645153764
	H220	1.324403249	-5.474004900	-7.328533007
	H221	-2.176070163	-4.381915650	-0.898631290
	H222	-3.910281586	2.880324572	2.628230259
	H223	-2.582904769	0.952216746	1.273837972
	H224	-8.108214211	0.877446260	3.314745500
	H225	-7.123299759	-0.074834214	0.659360483
	H226	-7.057866720	4.233689317	1.972742285
	H227	-8.546113101	9.017562918	3.681762130
	H228	-7.085333481	5.611004037	4.790635248
	H229	-9.061859398	5.704268565	2.849068379
	H230	-6.366620225	7.025080104	2.149900463
	H231	-10.712564747	9.709693182	0.106503051
	H232	-3.374145501	4.470604306	0.752668524

	H233	-4.864482940	4.008678962	-0.075648459
	H234	-9.874757248	10.708109296	2.306084326
	H235	-8.462176207	5.786055203	1.191318027
	H236	-6.198201926	2.132806389	0.933286296
	H237	-6.930857014	-0.423719937	3.106171752
	H238	-9.964784883	1.379357057	-0.810195991
	H239	-1.968071843	1.426331676	-2.898227837
	H240	-10.096583355	-0.365950882	-0.509472645
	H241	10.178892135	3.067039819	3.642173895
	H242	8.652020744	3.287687260	3.098733401
	H243	1.833756909	6.264577749	-1.292915564
	H244	-0.696297755	6.166784545	0.163975890
	H245	1.783465285	8.671974256	-0.206393732
	H246	-3.451889328	3.419436041	-2.093629776
	H247	-4.976486782	4.057198065	-3.426547194
	H248	-4.698259663	5.370679660	-2.563111460
	H249	-5.132014110	1.222805036	-4.312579981
	H250	-6.901852492	1.030483154	-4.476772984
	H251	-0.319383792	12.281384747	-2.701314102
	H252	-1.838962783	13.220344642	-2.612312293
	H253	-0.430111232	9.947968129	-3.033247387
	H254	-1.927206774	9.076258626	-2.897328172
	H255	-2.218440497	8.920573138	-6.305775427
	H256	-1.978326723	4.259114518	-5.937285487
	H257	-7.251952515	8.556286005	-1.344622558
	H258	-2.978205764	8.756482294	1.198029968
	H259	-4.155421900	7.551330514	-3.701108867
	H260	1.514839808	4.865168212	-3.683370908
	H261	2.821986927	6.896543271	-3.355215817
	H262	-0.999968132	4.563967177	-3.441986024
	H263	-8.105731833	-0.726630490	-1.677872682
	H264	3.930027836	9.604990565	-4.619529829
	H265	-5.903810302	-1.199652249	-3.412553344
	H266	1.254452563	5.634264835	-5.252112683
	H267	-3.206236908	7.183971979	-0.689875203
	H268	-6.857098301	3.029143254	-2.986025314
	H269	3.575820884	10.289971660	-3.020087229
	H270	-1.576030104	7.433208252	-6.880217316
	H271	-3.918717047	8.918623298	-4.750993872
	H272	-4.873867497	-3.807738272	-3.570676146
	H273	-5.635536218	-3.466952583	-1.001579644
	H274	-6.979963413	10.219703285	0.469863536
	H275	-5.337793109	1.197801151	-1.839365341
	H276	0.033698772	7.516496113	-4.176270369
	H277	-0.965359672	5.644007991	-6.296136412
	H278	-0.166018507	3.652572069	-4.691672247
	H279	-3.202164947	5.535026865	-4.277506961
	H280	0.970724442	9.953626596	2.250073674
	H281	-0.557863905	10.403791241	-0.636484610
	H282	-0.514174242	13.331471808	-0.519382795
	H283	2.310780766	10.395357522	1.168541193
	H284	-2.419578009	11.110342173	1.058918342
	H285	5.158582496	-3.225264370	-8.761474434
	H286	5.120833382	-4.659327093	-7.719019570
	H287	8.202343230	-5.296156790	-4.455075485
	H288	5.651171967	-5.355623891	-9.933656109
	H289	4.087816815	-5.798220786	-9.708230250
	H290	5.759477457	5.146748361	-0.525626842
	H291	5.707062572	7.994913970	0.667604023

H292	5.226336960	7.950349055	-3.618731526
H293	4.283840755	6.007363885	0.007195938
H294	6.926103511	8.499896031	-0.296475573
H295	5.856087945	2.543280060	-6.720670269
H296	6.365967812	6.997927242	-2.009532641
H297	5.109131932	-6.344436295	5.423665208
H298	7.047739781	-7.476584648	3.336432805
H299	4.040108881	1.170417615	-6.622119844
H300	4.582264621	0.412436069	3.487252914
H301	1.017821339	1.262821907	-8.567709240
H302	-0.998074514	-2.660819457	-4.228991043
H303	0.881782109	1.880064033	-6.917945586
H304	4.028251546	-4.027549852	4.589335208
H305	2.518066154	-0.139463303	-6.361024310
H306	4.058004783	-4.607290900	2.924043804
H307	4.765912834	-6.750421275	3.731071171
H308	6.522550337	-4.959155195	3.059799796
H309	6.524183685	-4.438859415	4.757027524
H310	-4.744651676	-1.114947334	-6.660390805
H311	5.841945087	-8.754938628	5.768352137
H312	4.904644654	-11.322704936	4.395676688
H313	0.433007033	-0.648838805	-3.863039946
H314	6.313798190	-10.964189055	6.927122847
H315	4.750070255	-11.369207986	6.651268703
H316	5.261447102	-2.688472721	2.220182786
H317	-0.251091056	1.534265362	-4.786641813
H318	-3.081388796	-2.477160593	-5.572314482
H319	7.811638900	-9.738344503	-0.605063354
H320	6.900600261	-7.663856096	-3.006374423
H321	6.585299571	-8.027295229	0.676122997
H322	7.152759915	-10.507578617	-1.770955606
H323	5.318754382	2.290793383	-9.441017913
H324	6.458598635	-4.929180998	0.199167710
H325	2.468833629	-7.237093834	1.401129943
H326	3.764059564	-2.223409624	-7.213902780
H327	3.128966283	-9.053194842	-0.248771692
H328	3.655730968	-7.096897100	-6.681324288
H329	2.645954340	-5.568727656	-4.910319051
H330	6.542289568	-11.867455532	4.775647048
H331	4.885223213	-0.527233842	2.050357003
H332	8.441319699	-5.593735121	1.386552704
H333	7.192149130	-3.561940890	0.311838312
H334	8.966681704	-6.450911087	3.549807986
H335	4.465233280	-2.300083960	5.707730844
H336	3.101784906	-11.750640875	-3.037757807
H337	3.663279595	-8.240408877	-3.154934478
H338	4.962758290	-8.405285560	-1.959211353
H339	1.928736384	-8.612166162	-1.498473566
H340	1.759805999	-2.328642051	-5.890872738
H341	-2.342098873	1.726669781	-6.170744031
H342	-4.587844503	0.654792169	-6.776617051
H343	2.159729394	-5.589531623	0.755473978
H344	8.520498339	-7.141282195	1.363401125
H345	7.320080635	-9.035870903	1.599367561
H346	3.870149294	-0.695716740	5.473736556
H347	6.069278851	-7.766853697	-6.102649414
H348	6.729932942	-5.978738873	-2.527398983
H349	0.949774049	-3.179213930	-7.238192215
H350	4.733755686	3.909515372	-6.908294114

	H351	7.227432422	0.215311837	4.399147751	
	H352	7.537226420	0.660589214	2.704743204	
	H353	9.425693250	-6.961211818	-5.585590791	
	H354	8.671239477	-8.182569609	-4.799038200	
	H355	10.519739122	-8.410034982	-0.724505749	
	H356	8.811339100	-8.159170117	-2.200370550	
	H357	11.985924579	-7.467310598	-2.850088220	
	H358	10.339325653	-6.682329926	-0.361772091	
	H359	10.243865978	0.154380704	3.060327635	
	H360	-3.291896161	13.444931004	2.898627277	
	H361	10.036576602	-6.304171793	6.360880046	
	H362	9.606468152	-3.945606499	5.013803657	
	H363	-5.018750970	12.396599230	0.682190253	
	H364	-4.052676670	10.356661073	2.780733165	
	H365	-5.728316662	10.872001763	2.542666701	
	H366	-6.244277653	6.931750968	-2.560011844	
	H367	1.100801797	-3.340050144	-1.435845664	
	H368	1.015773981	-0.693811744	0.424297069	
	H369	-1.370674068	0.079475205	-0.822038677	
	Mo370	3.920973219	-4.380674176	-2.065480887	
	N371	-6.744492974	-7.210630782	-0.557630105	
	N372	-8.862966231	-4.564811325	1.019572456	
	N373	-8.873628164	-4.749588121	3.854528251	
	N374	-6.384131904	-5.926722007	4.426654547	
	N375	-4.607023229	-3.874684912	3.526345720	
	N376	-5.336225259	-1.818048356	5.364643584	
	N377	-4.855039902	-2.997792628	7.951132015	
	N378	-2.277930887	-4.282840107	7.917305158	
	N379	-0.431056440	-2.683111967	6.572767852	
	N380	-0.847097604	0.260644669	6.720381890	
	N381	-1.757097061	0.437194408	9.481163868	
	N382	5.506984524	-10.762853900	6.332294750	
	N383	6.244022057	-8.663645973	4.828732465	
	N384	4.956370312	-2.756449907	3.197161314	
	N385	4.194222625	-1.565872777	5.062676969	
	N386	4.503253687	-0.490055291	3.021503958	
	N387	8.973955063	-6.477031230	4.592550901	
	N388	-5.587773672	-3.190910327	-3.141139142	
	N389	-1.506986037	-5.643337062	-2.961690033	
	N390	-5.949443703	-0.662920974	-2.531037419	
	N391	-8.302698480	0.225532846	-1.378170576	
	N392	-7.741971354	0.389700529	1.336097471	
	N393	-5.747116675	2.296479337	1.832114470	
	N394	-3.091453466	2.639441352	-1.486457696	
	N395	-2.086089367	0.843635375	-0.822204542	
	N396	-6.438606782	4.931485822	2.377165686	
	N397	-9.723584077	7.912094866	0.768717997	
	N398	-9.704846713	9.749688959	2.011946036	
	N399	9.380379719	2.579974789	3.228355995	
	N400	6.386430552	7.671766190	-0.028856532	
	N401	4.581237095	8.451392789	-3.013642607	
	N402	2.012283901	7.494378855	-3.587008766	
	N403	-2.570709237	5.984571330	-4.962810175	
	N404	-2.010450509	7.944222961	-6.118962918	
	N405	-3.647139718	7.979350401	-4.476666628	
	N406	0.872131228	6.593834205	-1.126250896	
	N407	1.012508031	8.950230964	0.409170457	
	N408	-0.487617602	11.296836661	-0.143858745	
	N409	-1.423046505	9.952274660	-2.768098731	

	N410	-2.990065591	11.925205998	0.845984170
	N411	4.703413818	-4.988001433	-9.825432494
	N412	2.966867854	-2.786708262	-7.535720216
	N413	1.834668713	0.012139602	-7.129796030
	N414	3.933785759	1.972850485	-7.257293966
	N415	-1.003001183	-3.335717420	-10.017394631
	N416	-2.744075861	-1.493230390	-8.699523127
	N417	8.534168344	-7.233687115	-5.161224225
	N418	4.551794687	-5.763723989	-3.934949248
	N419	4.155813312	-6.779527774	-5.855075034
	N420	9.470049668	-7.379246608	-2.179479515
	N421	0.238531977	2.926955400	-0.703218007
	N422	1.018175281	2.158229152	-0.993091578
	O423	-6.706758453	-5.162171446	1.442522379
	O424	-7.776383933	-2.752839280	3.929099878
	O425	-6.524739969	-5.099844272	6.554300999
	O426	-3.161628159	-4.602534513	5.127193320
	O427	-3.198786944	-1.059107737	5.193902855
	O428	-4.253910826	-0.900207847	8.623836634
	O429	-2.182589492	-3.979792229	10.191279474
	O430	1.288700044	-4.119704185	7.079212310
	O431	1.418064354	0.159127633	7.007077911
	O432	-0.185122418	2.065395895	9.650243635
	O433	-0.223353888	0.324933966	12.723214551
	O434	6.915570684	-9.990845580	3.055331543
	O435	7.794445095	-6.971273444	6.502892809
	O436	11.619462061	-4.147861492	5.274203121
	O437	-5.581690791	-0.945339648	-0.282526673
	O438	-3.365025516	-4.774786402	-3.943934196
	O439	-7.580209998	2.391624821	-1.404459412
	O440	-6.228445096	2.864543588	-3.733310680
	O441	-9.849181344	1.213221228	1.758624355
	O442	-5.607639445	1.659010789	4.024147058
	O443	-4.319516832	5.384067547	3.099265270
	O444	-6.704660398	7.612676232	4.667906203
	O445	10.092379637	-0.373726738	5.027570875
	O446	3.487740134	5.123527151	3.981284063
	O447	3.782533522	8.726266687	-0.899435925
	O448	1.243410557	9.644662758	-3.831920378
	O449	-1.177412366	6.976154749	-2.071363329
	O450	-0.636599657	8.149092402	1.782659366
	O451	2.349080188	6.318262134	1.543325254
	O452	0.762905198	12.496095666	1.359220760
	O453	-3.112186861	13.848726538	-0.378772622
	O454	-3.387030517	11.115623761	-2.643648799
	O455	-5.323330029	13.571935082	3.044795058
	O456	-5.337672022	6.879552293	-2.199932274
	O457	2.310314664	-4.783535880	-8.466433890
	O458	0.260316305	-1.038902945	-8.428670353
	O459	2.591900920	3.310210562	-8.513449464
	O460	6.187733459	4.102377140	-9.088379334
	O461	-4.398944186	-3.021444266	-9.059958508
	O462	-1.009704246	-6.797154928	-5.813385577
	O463	0.922626805	-5.946281452	-6.532279115
	O464	-3.411596780	1.983672368	-8.604238783
	O465	10.176872408	-5.361165113	-2.930736857
	O466	12.851236990	-6.784235249	-1.133839761
	O467	-0.161324120	-6.243526206	-0.263659739
	O468	0.231399350	-8.250374880	0.606403459

	O469 5.141403869 -10.863714106 -2.496602990<br>O470 2.911293497 -10.805340830 -2.857549934<br>O471 5.154974112 -5.755335325 -0.772235883<br>O472 4.971109161 -7.436159035 0.719895823<br>O473 2.741381751 -6.080032879 -1.741132204<br>O474 8.975082027 -6.345141178 1.746247439<br>O475 7.131040377 -4.439081231 0.738171526<br>O476 -4.255223008 4.645949469 -3.069542864<br>O477 8.030505251 -10.311697129 -1.383455696<br>O478 7.463144394 -8.497400661 0.778574564<br>S479 4.009261728 5.109050288 -2.238329989<br>S480 2.495819738 2.037444233 -3.457188375<br>S481 2.281528777 -3.418098751 -3.527069136<br>S482 3.702345037 1.955628556 0.036931148<br>S483 0.753788265 -0.768400853 -0.907850464<br>S484 4.426079342 -0.808613950 -5.287433721<br>S485 3.320255604 -3.358524747 0.031407876<br>S486 5.998709749 1.688096150 -2.671262318<br>S487 5.841393405 -3.101664731 -2.640944611<br>S488 6.201215727 -0.854649476 0.277859790

11 35 (S=1)

**11 35 (S=1)**	bm22b2n2x6x3b5635tl.car_3
Fe(139) -2.306<br>Fe(140) -0.847<br>Fe(141) -2.231<br>Fe(142) 2.924<br>Fe(143) 2.856<br>Fe(144) 2.138<br>Fe(145) -1.297	C1 -7.518459921 -5.507418575 1.928217588<br>C2 -7.984592279 -6.535587666 0.890164909<br>C3 -8.187805273 -3.696557131 3.447024483<br>C4 -7.473349643 -4.150390813 4.718117184<br>C5 -7.130832656 -5.918313867 6.369625419<br>C6 -5.642428143 -6.215039077 6.161627837<br>C7 -3.879838730 -6.787096863 4.561666731<br>C8 -3.080997141 -5.481548525 4.502100547<br>C9 -3.058245338 -3.117952413 3.903162008<br>C10 -2.865001505 -2.526642781 5.307218869<br>C11 -3.782525776 -2.139425645 7.517140815<br>C12 -2.790276754 -2.894607966 8.410275144<br>C13 -2.048111355 -5.064518776 9.259419135<br>C14 -0.612524113 -5.381661926 8.822368042<br>C15 0.881518290 -5.927297945 6.947433035<br>C16 1.709604833 -4.878727032 6.201717355<br>C17 1.744691844 -2.610408455 5.221068735<br>C18 1.985602271 -1.377132495 6.110899423<br>C19 0.992264166 -2.265601357 3.906038773<br>C20 1.578420838 -1.028591756 3.216007093<br>C21 0.981822364 -3.469732053 2.957278686<br>C22 0.962227413 0.347974283 7.556273442<br>C23 1.372674203 -0.070445563 8.980277617<br>C24 -0.357129922 1.157645342 7.528286808<br>C25 -0.350194024 2.274168424 8.578844679<br>C26 -0.611665439 1.734954347 6.130047306<br>C27 0.949091256 -1.457306410 10.923175549<br>C28 2.404467936 -1.824039711 11.127982296<br>C29 6.558770093 -11.651156872 1.526934859<br>C30 6.951621691 -10.326870524 0.870617450<br>C31 7.374399945 -7.913055569 1.378019825<br>C32 8.760977487 -7.609547004 1.978027497<br>C33 6.309062924 -6.936589930 1.911009702<br>C34 6.613246184 -5.461996034 1.630221908<br>C35 5.392954524 -4.596021412 1.945532014

	C36	5.317686433	-2.134692092	2.420597810
	C37	11.004050544	-6.739263750	1.520337648
	C38	11.260957826	-5.250195487	1.623774293
	C39	-5.573348398	-2.564828750	-0.826336268
	C40	-5.831067634	-1.191321652	-0.187851403
	C41	-4.051342235	-2.695649047	-1.062857492
	C42	-3.537133051	-4.106772757	-1.420106826
	C43	-3.527887770	-4.434903846	-2.911839466
	C44	-6.568920114	1.096889782	-0.673402910
	C45	-7.595538289	1.397450578	0.422372719
	C46	-6.850046370	1.968359060	-1.916128838
	C47	-9.223222978	0.514331434	2.012289940
	C48	-8.456288423	0.487050454	3.343255698
	C49	-6.326779034	0.040447165	4.487737857
	C50	-5.149402353	1.008399035	4.568991246
	C51	-3.922563758	2.881706777	3.552664153
	C52	-4.180043270	4.106140782	4.461625087
	C53	-3.660442188	3.450988292	2.150292485
	C54	-3.128866388	2.498767956	1.128749036
	C55	-2.367202515	1.357738951	1.195598549
	C56	-2.648139591	1.844385156	-0.950994453
	C57	-5.893896862	5.694332884	5.234873290
	C58	-5.460462216	5.725729996	6.697882495
	C59	-7.434756714	5.796637663	5.114332872
	C60	-8.023180873	7.169473589	5.256654883
	C61	-7.802314708	8.153275042	6.200894421
	C62	-9.314514273	8.824367016	4.727147455
	C63	9.658396640	0.958070136	1.926302462
	C64	10.486431518	-0.219783039	1.446654775
	C65	8.173146636	0.512674601	1.884562645
	C66	7.225571546	1.575205659	2.387609287
	C67	6.905184909	1.680371135	3.753291526
	C68	6.630177769	2.488091638	1.499544952
	C69	6.015189770	2.654538635	4.219526363
	C70	5.754598965	3.475130602	1.952183011
	C71	5.442947047	3.553263924	3.312214927
	C72	5.396936858	7.396117650	-1.451023170
	C73	4.271690425	8.439866508	-1.476459256
	C74	4.807815781	5.995617689	-1.219225810
	C75	2.865377759	9.887356180	-2.870801973
	C76	1.448769913	9.347621314	-2.671007060
	C77	-0.010860200	7.369099029	-2.516716769
	C78	-0.286599994	7.083697374	-1.026771636
	C79	-0.107725210	6.108925371	-3.396854854
	C80	-1.406640859	5.296840950	-3.341921335
	C81	-2.647575567	5.898356649	-4.029458155
	C82	-3.719121025	7.976922753	-3.122221383
	C83	0.587373077	6.498254096	1.182343106
	C84	0.657408940	7.779932284	2.039676856
	C85	1.573157846	5.474010507	1.754888036
	C86	1.847236082	9.886914368	2.416749313
	C87	1.046509056	11.188503587	2.426618459
	C88	-0.901485345	12.421242749	1.543189841
	C89	-2.043872021	12.628038359	2.561900495
	C90	-1.448388217	12.523745826	0.097803515
	C91	-2.380078595	11.362769928	-0.228039807
	C92	-3.409262052	11.603921821	4.335874504
	C93	-3.100261916	12.470021964	5.554060916
	C94	-3.762225191	10.159985499	4.782211321

	C95	-4.242878350	9.295923954	3.639111052
	C96	-5.589756960	9.315502340	3.246306715
	C97	-3.353542910	8.483698378	2.915835099
	C98	-6.036761767	8.564280159	2.155834862
	C99	-3.776084803	7.741805027	1.809128661
	C100	-5.119063024	7.794220407	1.440889945
	C101	2.190008886	-2.698129407	-10.092113007
	C102	0.912297952	-2.548511019	-9.281640319
	C103	-0.205924247	-1.249865844	-7.484238674
	C104	-0.794835133	0.142673913	-7.727307200
	C105	-0.391796083	2.549423117	-7.461915258
	C106	0.730561763	3.528519101	-7.798379752
	C107	3.089582640	4.104522161	-7.780921930
	C108	3.343577271	4.467021080	-9.230673240
	C109	-4.534025630	-2.326846533	-8.857780668
	C110	-5.478417158	-1.302526707	-8.212175042
	C111	-3.827157274	-3.147003828	-7.751747983
	C112	-3.175478849	-4.420651129	-8.324895669
	C113	-2.267558874	-5.067232431	-7.302209028
	C114	-5.711393883	0.906844806	-7.171115776
	C115	-5.380906937	2.272806903	-7.744117557
	C116	-5.660660995	0.830647638	-5.622443279
	C117	-4.279721297	0.589191194	-5.058561343
	C118	-3.354642510	1.633383891	-4.878495457
	C119	-3.871308245	-0.721550338	-4.754124125
	C120	-2.053935941	1.366388168	-4.430860951
	C121	-2.570739648	-0.990127581	-4.313213807
	C122	-1.652470492	0.052717469	-4.156575777
	C123	6.781544106	-5.474375159	-6.910678220
	C124	8.130726229	-5.628222561	-6.192328926
	C125	5.616471307	-5.891952372	-5.999010468
	C126	4.242952788	-5.622729993	-6.531291796
	C127	3.688425856	-6.096534283	-7.704705822
	C128	2.141060432	-5.025548036	-6.536238308
	C129	9.673953683	-7.029913112	-4.940930959
	C130	10.864856627	-6.625676521	-5.781671401
	C131	0.451215685	-7.045892956	-1.106823218
	C132	1.906095964	-6.666971750	-1.130145883
	C133	2.539755689	-6.695424870	-2.583043593
	C134	2.291381794	-8.088672951	-3.220942032
	C135	3.023073144	-8.261221510	-4.563343081
	C136	3.037525277	-9.675620314	-5.070883124
	C137	4.080941310	-6.547813481	-2.392646261
	C138	3.053910672	-0.361119324	-2.975677250
	Fe139	3.112339292	-1.380409281	-4.671308871
	Fe140	1.834273046	1.461979720	-1.793725811
	Fe141	4.441747828	0.724955268	-2.120945101
	Fe142	3.166553963	1.142733814	-4.319388297
	Fe143	3.554492743	3.204135476	-2.593526661
	Fe144	4.298074281	-1.769619581	-2.424033606
	Fe145	1.672875241	-1.837014879	-2.570848845
	H146	-6.188154774	-7.471072458	0.650763519
	H147	-6.525202545	-6.399783803	-0.533976042
	H148	-9.452581091	-4.841559254	2.158245777
	H149	-8.753482600	-6.075494172	0.249811501
	H150	-8.493294645	-7.338048958	1.451092708
	H151	-7.535325509	-2.945252329	2.979589855
	H152	-8.481612198	-5.937821828	4.692746317
	H153	-9.127681411	-3.206201304	3.735009523

	H154	-7.637256813	-6.849306252	6.657836237
	H155	-5.953971506	-6.503705394	4.152220533
	H156	-7.203666783	-5.212360165	7.207817824
	H157	-2.064549312	-3.176619324	3.441351594
	H158	-4.609497163	-4.546784258	3.469953204
	H159	-3.832792109	-7.291763955	3.586913446
	H160	-3.454643540	-1.093056563	7.476773643
	H161	-4.775997978	-3.098146940	5.865015425
	H162	-3.391788468	-7.425650813	5.308338198
	H163	-3.677341640	-2.438375452	3.300302214
	H164	-3.557369992	-4.713702335	7.788034784
	H165	-4.775136194	-2.174667157	7.987072992
	H166	0.519903559	-4.352327162	3.423553859
	H167	0.414027564	-3.229825368	2.045668254
	H168	2.004974930	-3.736414445	2.647573666
	H169	-0.047959901	-2.036084787	4.187485617
	H170	-2.567464531	-6.022217944	9.407541027
	H171	-0.647290519	0.949988532	5.361274152
	H172	-0.185741336	-1.453989866	9.123394135
	H173	-0.240589968	1.885128766	9.601317631
	H174	0.957114461	-0.759189831	2.348405698
	H175	2.596130435	-1.211617016	2.842521913
	H176	9.924592656	1.136878294	2.985670795
	H177	-1.574100834	2.266457192	6.114217375
	H178	-1.293540187	2.837202247	8.529849712
	H179	0.181925445	2.450238868	5.860671670
	H180	0.476006764	2.978850480	8.394814770
	H181	-1.181268899	0.461815419	7.772125003
	H182	1.786758038	0.984131491	7.202429179
	H183	-0.047059459	-1.178281883	6.429903758
	H184	1.610060669	-0.151522745	3.879968615
	H185	1.577666821	4.587017215	1.098873728
	H186	1.190968889	5.176265538	2.745517851
	H187	3.407113206	5.487912316	2.452393368
	H188	4.385933556	4.465460091	4.660653491
	H189	5.283514585	4.161226854	1.248426861
	H190	6.821915553	2.402373149	0.428102933
	H191	7.348940773	0.981809315	4.467869033
	H192	0.123015599	-3.586090655	6.233326616
	H193	0.729439264	-6.766978928	6.252076489
	H194	1.496491741	-6.287959861	7.782014879
	H195	-1.187514719	-5.460576425	6.829777807
	H196	-1.971306597	-4.562598141	10.232354701
	H197	5.763560006	2.707603606	5.281698329
	H198	2.746812161	-2.984008319	4.974759511
	H199	0.676852959	-0.673761370	11.647668682
	H200	0.350901189	-2.351925187	11.153910913
	H201	2.935434800	-2.186650515	10.218079774
	H202	11.746434824	-7.189546971	0.844338550
	H203	-2.415382700	-5.359772633	-4.359014331
	H204	0.992811541	-8.807739051	-0.542168780
	H205	-3.963529253	-5.140772344	-8.588244862
	H206	-2.607556377	-4.177166258	-9.234246285
	H207	-4.546336032	-3.434920878	-6.969028052
	H208	-5.146976926	2.262896224	-8.835075502
	H209	-5.213126661	-3.007021795	-9.395217779
	H210	-3.755450408	-1.978668318	-1.845694608
	H211	-4.158589281	-4.872495292	-0.923539454
	H212	-3.917079557	0.038465342	-7.961379028

	H213	-6.759647558	0.723099779	-7.477046613
	H214	-2.883488362	-1.196049308	-9.414725427
	H215	-1.666461867	-5.353216383	-2.761302633
	H216	-3.072882755	-2.507919789	-7.266809191
	H217	-3.242670969	-2.424154849	-10.450164817
	H218	-7.215600016	-3.218779045	-1.892891136
	H219	-3.556198587	-2.379106022	-0.133683807
	H220	-0.732491157	-4.231566005	-8.210645851
	H221	-2.523735052	-4.230608465	-1.012940710
	H222	-3.023068583	2.393166420	3.953060466
	H223	-1.993722427	0.797840450	2.045308720
	H224	-6.979155655	0.248587211	5.346625324
	H225	-6.731413452	-0.219799503	2.407384586
	H226	-6.198751190	3.793434539	4.289842357
	H227	-7.135801124	8.212953872	7.053998625
	H228	-5.499990083	4.729531315	7.202055472
	H229	-7.896006934	5.089954188	5.826331029
	H230	-5.413343933	6.533380861	4.708150662
	H231	-10.046036476	9.468114610	4.243682453
	H232	-2.937380481	4.274013344	2.270705529
	H233	-4.590422878	3.906872228	1.772678457
	H234	-8.703756651	10.089212986	6.327981159
	H235	-7.705704984	5.446673483	4.106367018
	H236	-5.670589851	1.882424986	2.771627485
	H237	-5.933762431	-0.976839681	4.626000854
	H238	-9.776900487	1.462204752	1.989027765
	H239	-2.579091181	1.804164825	-2.035333273
	H240	-9.954863162	-0.303167879	2.018562186
	H241	10.799808383	2.521983857	1.252064049
	H242	9.195042398	2.828538047	1.237171831
	H243	1.662571803	6.479169171	-0.694926700
	H244	-0.426489939	6.098111819	1.321505944
	H245	1.976877994	8.635579643	0.718127047
	H246	-3.786597773	3.618557705	-0.596849514
	H247	-5.554234946	4.484605360	-1.400671280
	H248	-5.041241768	5.641235349	-0.432890770
	H249	-6.040117844	1.827550999	-2.645263204
	H250	-7.801207665	1.659807810	-2.381627688
	H251	-0.590892902	12.554715524	-0.590561213
	H252	-2.007834957	13.463529646	0.016347746
	H253	-0.864820014	10.293945377	-1.218948412
	H254	-2.313839863	9.410546012	-0.849527597
	H255	-3.427866512	9.766759431	-4.070970042
	H256	-3.262393415	5.090137536	-4.451697203
	H257	-7.091716868	8.579991411	1.868551989
	H258	-2.302575668	8.415630244	3.209894412
	H259	-4.714016741	7.998781944	-1.305580375
	H260	0.716743747	5.428473335	-3.130338141
	H261	2.109462066	7.391641238	-2.822196603
	H262	-1.657118636	5.029076370	-2.303391514
	H263	-8.312492901	-0.502313572	0.350799342
	H264	2.926259191	10.311066917	-3.882168691
	H265	-6.659248605	-0.704746647	-1.943773169
	H266	0.075650159	6.424504097	-4.437480279
	H267	-3.058334707	7.163052244	1.223616651
	H268	-7.311320714	3.410387989	-0.677773306
	H269	2.984591637	10.712037050	-2.154205835
	H270	-2.980376519	8.386029568	-4.996932143
	H271	-4.699289964	9.511571998	-2.163542335

	H272	-5.789732723	-3.240923836	-2.758543165
	H273	-5.856289861	-3.301704382	-0.051712357
	H274	-6.309013684	9.925459454	3.798399577
	H275	-5.613504926	1.429520005	-0.234684806
	H276	-0.778015746	8.097458264	-2.810381753
	H277	-2.345014190	6.522207125	-4.880497722
	H278	-1.181707848	4.347979940	-3.851045148
	H279	-3.984522901	6.102350482	-2.386220848
	H280	1.865569424	9.527153633	3.456566895
	H281	-0.346373184	10.386077692	1.150072137
	H282	-0.212653841	13.263058520	1.708629530
	H283	2.874840828	10.153119738	2.135209854
	H284	-1.704936781	10.767811327	3.329937688
	H285	2.646889381	-1.710049675	-10.252008248
	H286	2.890645094	-3.270714060	-9.448975491
	H287	6.690770137	-4.400821569	-7.129501644
	H288	2.788990295	-3.649894242	-11.792064041
	H289	1.341916339	-4.164474020	-11.219295167
	H290	5.635325801	5.275779461	-1.240537882
	H291	5.888768543	7.825637102	0.477333240
	H292	4.389983323	8.536489469	-3.523813927
	H293	4.330816233	5.966404185	-0.228227909
	H294	6.856223551	8.565015531	-0.611408769
	H295	4.013392630	3.645953821	-7.393438215
	H296	5.913914102	7.398249319	-2.423794311
	H297	6.197710116	-7.099766986	2.995805460
	H298	7.416779472	-7.847023209	0.283933231
	H299	2.256870270	2.228959543	-7.205226423
	H300	5.247352149	-0.101840946	2.428019808
	H301	-1.128856697	2.597799010	-8.272937872
	H302	-2.263351557	-2.017826569	-4.106473310
	H303	-0.883720761	2.948959141	-6.558737010
	H304	5.112534817	-4.686442285	3.002883149
	H305	0.812165507	0.903393586	-6.722092924
	H306	4.528213734	-4.955345474	1.360309364
	H307	5.355292725	-7.219324990	1.435961489
	H308	6.879463410	-5.317578215	0.571546126
	H309	7.467648244	-5.118261977	2.235340947
	H310	-6.326800364	0.012781865	-5.311537758
	H311	6.853918027	-9.532220959	2.701680193
	H312	5.528760312	-11.857534934	1.198784045
	H313	-0.628064646	-0.157102912	-3.836792183
	H314	7.514305199	-11.921950082	3.330476210
	H315	5.912112221	-12.263320645	3.382085787
	H316	5.721905134	-2.949122964	0.629687123
	H317	-1.326295179	2.173312443	-4.305730921
	H318	-4.575941115	-1.548677291	-4.869583437
	H319	7.129062248	-9.483842863	-4.053833398
	H320	5.701091422	-6.973817607	-5.793586982
	H321	6.293615451	-8.010662023	-2.281900258
	H322	6.155514159	-10.064843615	-5.098816591
	H323	2.958060191	3.728590054	-9.975924653
	H324	6.136801955	-4.870793560	-2.171185984
	H325	2.501290333	-7.317978210	-0.473700024
	H326	1.756950312	-1.001113757	-8.225642083
	H327	2.638332245	-8.859953844	-2.516288667
	H328	1.664609417	-5.916661961	-8.390836048
	H329	1.173255963	-4.629698141	-6.256310860
	H330	7.197435924	-12.425283418	1.069080592

	H331	5.181879526	-0.825777207	0.835816105
	H332	8.327497209	-5.688594421	-1.643620613
	H333	6.903743533	-3.534753904	-2.019520547
	H334	9.369475075	-6.879898468	0.125121531
	H335	5.647269848	-3.097302602	4.214839994
	H336	1.903330546	-11.103762635	-5.573168179
	H337	2.543799994	-7.619842645	-5.317766103
	H338	4.071311767	-7.953655025	-4.478600412
	H339	1.210681438	-8.216808273	-3.377707510
	H340	0.158715120	-1.346867547	-6.449997645
	H341	-3.655578226	2.657679850	-5.112847171
	H342	-6.086126033	1.773901366	-5.245015463
	H343	1.982911782	-5.628919277	-0.782284119
	H344	8.355650330	-7.212183373	-1.949144114
	H345	7.207998579	-9.127476363	-1.705040406
	H346	4.899668038	-1.539333014	4.353688514
	H347	4.122907402	-6.662971757	-8.519796572
	H348	5.720629063	-5.387076461	-5.030946474
	H349	-0.993041153	-1.987028615	-7.657274399
	H350	2.908575646	5.026280681	-7.206943415
	H351	8.088671403	-0.394052246	2.503151185
	H352	7.911207096	0.241142786	0.848971621
	H353	7.515601353	-5.841247333	-8.792927868
	H354	6.953425575	-7.186911809	-8.050364028
	H355	9.780171610	-8.105986458	-4.732038383
	H356	7.740744595	-7.607499204	-5.622740549
	H357	10.704137313	-6.683297689	-6.885244672
	H358	9.699802745	-6.501708215	-3.973779602
	H359	10.623414288	-0.266097170	0.338868967
	H360	-2.022270224	12.714269065	5.704919864
	H361	11.161181470	-7.164124886	2.527566526
	H362	10.356636127	-4.619833530	1.805595584
	H363	-4.284055134	12.053631392	3.839725523
	H364	-2.879293211	9.706358028	5.260205893
	H365	-4.540679813	10.243279827	5.553684183
	H366	-6.483747227	7.180423147	0.193912886
	H367	0.270057481	-2.462681493	-2.459522577
	H368	3.934672956	-4.287662364	-0.699230585
	H369	1.364436595	-0.675713360	0.076503200
	H370	-1.372269508	0.261632959	-0.384528538
	Mo371	3.158874412	-3.932130217	-3.659063769
	N372	-6.945281286	-7.118205718	0.060006303
	N373	-8.503009660	-4.770057439	2.515602545
	N374	-7.792254059	-5.379643911	5.190985873
	N375	-5.278260384	-6.591890429	4.910443261
	N376	-3.697837720	-4.425366691	3.912281619
	N377	-3.893480561	-2.682537390	6.173391680
	N378	-2.841600374	-4.250182817	8.353716441
	N379	-0.397366371	-5.491041998	7.480129572
	N380	1.098249080	-3.689192653	5.957865462
	N381	0.877839552	-0.810249649	6.664882398
	N382	0.658698852	-1.072146675	9.555159959
	N383	6.599391442	-11.616612878	2.992912860
	N384	7.003948096	-9.281310201	1.718165717
	N385	5.598880850	-3.178344216	1.620928348
	N386	5.243104370	-2.288146275	3.757555366
	N387	5.067109001	-0.936404114	1.872817350
	N388	9.652681080	-7.067346306	1.111663698
	N389	-6.347039244	-2.708286947	-2.064332307

	N390	-2.447671298	-5.096362775	-3.368558416
	N391	-6.450079026	-0.305921335	-1.013736887
	N392	-8.393092788	0.391726258	0.829450512
	N393	-7.142711356	0.115319879	3.288193882
	N394	-5.013443636	1.913366456	3.549496206
	N395	-3.293554211	2.769192983	-0.223049252
	N396	-2.095486396	0.971038876	-0.101806564
	N397	-5.485052011	4.429898675	4.635535545
	N398	-8.972418640	7.600531017	4.342945645
	N399	-8.628249361	9.197926540	5.844380906
	N400	9.890142056	2.102638663	1.043154552
	N401	6.389834152	7.676987767	-0.404809841
	N402	3.937914572	8.928674376	-2.702172689
	N403	1.283008637	8.008784379	-2.740268746
	N404	-3.527943753	6.657058541	-3.131682327
	N405	-3.219166918	8.773236902	-4.090343687
	N406	-4.377996969	8.550496859	-2.096851108
	N407	0.772418532	6.731276876	-0.248974728
	N408	1.392590876	8.814626167	1.541238610
	N409	-0.130535952	11.195905738	1.735222099
	N410	-1.754395913	10.250472010	-0.704871191
	N411	-2.297007532	11.591964951	3.399728052
	N412	1.908427232	-3.327600675	-11.384556701
	N413	0.893145854	-1.528597600	-8.401776356
	N414	-0.027331722	1.157292095	-7.274140292
	N415	2.015850001	3.141470527	-7.615141206
	N416	-3.660070044	-1.701902032	-9.859068849
	N417	-4.925478768	-0.109086949	-7.853092553
	N418	6.756422537	-6.192224629	-8.201176720
	N419	3.257288758	-4.943398295	-5.814954328
	N420	2.365443718	-5.708320318	-7.684983931
	N421	8.416043278	-6.842332316	-5.644325313
	N422	0.026253545	3.243827762	-0.363012498
	N423	0.714859094	2.538119968	-0.924239971
	O424	-6.324495925	-5.380658199	2.258251055
	O425	-6.657145327	-3.404613826	5.285028222
	O426	-4.843251840	-6.141404540	7.109600455
	O427	-1.937874844	-5.398626128	4.977812416
	O428	-1.821685034	-1.928993623	5.618794709
	O429	-2.017387684	-2.281724016	9.166277815
	O430	0.265370161	-5.564684108	9.675367182
	O431	2.862158978	-5.157314862	5.838099392
	O432	3.133626676	-0.934568127	6.283274399
	O433	2.313165280	0.480769021	9.575355048
	O434	2.951491486	-1.793628409	12.220613122
	O435	7.162499150	-10.286647713	-0.360092355
	O436	9.000130064	-7.851655124	3.174107195
	O437	12.372815226	-4.754624351	1.537295617
	O438	-5.496986668	-0.911936462	0.981196288
	O439	-4.476902183	-4.116636750	-3.664473159
	O440	-7.631885001	2.543468535	0.922949627
	O441	-6.895716138	3.361730850	-1.575311135
	O442	-9.046886060	0.793918417	4.385673331
	O443	-4.376503550	0.969563423	5.530144227
	O444	-3.242472111	4.781828547	4.896603082
	O445	-5.130353220	6.733127317	7.298186183
	O446	10.954295746	-1.075807440	2.183864768
	O447	4.550773412	4.532904744	3.702267727
	O448	3.698208220	8.823371190	-0.439514456

	O449 0.516359378 10.159220979 -2.492895268<br>O450 -1.442468705 7.202506991 -0.581876740<br>O451 0.092836144 7.816785120 3.142766665<br>O452 2.874854867 6.058134717 1.859380838<br>O453 1.465066986 12.160154869 3.068596148<br>O454 -2.677827455 13.691715006 2.583649912<br>O455 -3.594541289 11.398832726 0.024813622<br>O456 -3.959884253 12.864030585 6.324495521<br>O457 -5.518315335 7.075773608 0.299071650<br>O458 -0.032797035 -3.365341062 -9.428994285<br>O459 -1.887088029 0.302042100 -8.314016489<br>O460 0.430517046 4.668453407 -8.196168074<br>O461 3.955368693 5.464854022 -9.576950357<br>O462 -6.675808192 -1.566791394 -8.013826999<br>O463 -2.680886989 -5.752209759 -6.370293590<br>O464 -0.948297661 -4.824067500 -7.422340645<br>O465 -5.394763482 3.317866713 -7.109543396<br>O466 8.965981677 -4.705520467 -6.164708619<br>O467 11.953787933 -6.330068504 -5.309206946<br>O468 -0.482669294 -6.306365928 -1.381942645<br>O469 0.162930642 -8.339270701 -0.754475343<br>O470 4.042933955 -10.336884153 -5.317724246<br>O471 1.790122288 -10.186377111 -5.244225916<br>O472 4.601214887 -5.492303881 -2.927795394<br>O473 4.733476069 -7.432913078 -1.791163224<br>O474 2.025306269 -5.676686592 -3.356728581<br>O475 8.916565836 -6.481345207 -1.575601815<br>O476 6.911028175 -4.460465783 -1.706462084<br>O477 -4.750185280 5.007288234 -1.132097300<br>O478 7.112302096 -9.932951598 -4.936770559<br>O479 7.161296319 -8.475753641 -2.452041978<br>S480 3.548285098 5.519909927 -2.495240653<br>S481 1.583164538 2.666282184 -3.705719565<br>S482 1.307329008 -2.717783190 -4.508692515<br>S483 3.598551128 2.138422493 -0.625957146<br>S484 0.671920729 -0.407223548 -1.063675776<br>S485 3.030787651 0.130770383 -6.326984713<br>S486 3.107490578 -3.318663641 -1.263778468<br>S487 5.199504870 2.088798306 -3.712781958<br>S488 4.998693679 -2.603998497 -4.379298579<br>S489 5.868872814 -0.765918682 -1.252694466

TS 11 → 12 35 (S=1)

**TS 11 → 12 35 (S=1)**	bm22b2n2x6x3b5635tm.car_1
Fe(139) -2.373<br>Fe(140) -0.657<br>Fe(141) -2.385<br>Fe(142) 2.934<br>Fe(143) 2.826<br>Fe(144) 2.151<br>Fe(145) -1.072	C1 -4.144469522 -8.481355055 1.134460534<br>C2 -4.230981807 -9.433037474 -0.062742081<br>C3 -5.358473380 -7.417898478 2.985167003<br>C4 -4.386868566 -7.694668995 4.129867962<br>C5 -3.130651744 -9.351594355 5.411263800<br>C6 -1.693668547 -8.911320604 5.111217049<br>C7 -0.043954954 -8.377373871 3.380636858<br>C8 0.085789676 -6.858671999 3.532953281<br>C9 -0.990038371 -4.678299108 3.360776187<br>C10 -0.921460227 -4.271224507 4.840696408<br>C11 -1.642906664 -4.684566284 7.119796922<br>C12 -0.325296234 -5.032571662 7.825912842<br>C13 1.370772846 -6.744477049 8.252713310<br>C14 2.742055219 -6.327469203 7.705281707

	C15	4.094820991	-5.880641491	5.702526990
	C16	4.310104948	-4.477976107	5.130016933
	C17	3.264375051	-2.295295708	4.605595501
	C18	3.041059658	-1.248833365	5.711857211
	C19	2.299692527	-2.124437330	3.401110539
	C20	2.226212995	-0.667208783	2.932846562
	C21	2.702339880	-3.051165859	2.248363171
	C22	1.530185748	-0.386958270	7.462597984
	C23	2.237383045	-0.777869749	8.775285199
	C24	-0.002940018	-0.252653581	7.629614530
	C25	-0.360140596	0.589898576	8.858673915
	C26	-0.635047654	0.346550884	6.367134784
	C27	2.684999457	-2.485160274	10.442442853
	C28	4.170427846	-2.196355805	10.528488382
	C29	11.044817618	-7.625884111	-0.831909890
	C30	10.766283864	-6.181986230	-1.251356798
	C31	10.165898038	-3.934064956	-0.329470605
	C32	11.341751644	-3.141616191	0.275013508
	C33	8.846282548	-3.627874456	0.401750193
	C34	8.457617802	-2.147324851	0.389986844
	C35	7.019574289	-1.959933309	0.873535099
	C36	5.955487964	0.125345051	1.778450266
	C37	12.926263940	-1.307374458	-0.085383156
	C38	12.534366848	0.109670506	0.279744257
	C39	-3.966735260	-4.611709389	-1.095979953
	C40	-4.727786834	-3.614811327	-0.205348188
	C41	-2.576105802	-4.008803230	-1.400268219
	C42	-1.544809451	-4.971268459	-2.027439049
	C43	-1.558735965	-5.032784230	-3.553607519
	C44	-6.420500803	-1.836665418	-0.247669758
	C45	-7.350540751	-2.191848832	0.915711528
	C46	-7.183266945	-1.002600938	-1.298956885
	C47	-8.268402827	-3.933606316	2.359672106
	C48	-7.432724271	-3.808834077	3.642075723
	C49	-5.210850347	-3.436697679	4.625114706
	C50	-4.554487345	-2.073587695	4.832953197
	C51	-4.371729090	0.275737931	4.132650271
	C52	-5.022436646	1.113264217	5.258980735
	C53	-4.539484987	1.099329533	2.847030657
	C54	-3.758147569	0.648834501	1.654895449
	C55	-2.588026300	-0.052203541	1.496980392
	C56	-3.250946156	0.600874347	-0.517197248
	C57	-7.155880310	1.632133662	6.378239938
	C58	-6.636183096	1.616479524	7.813712076
	C59	-8.594222764	1.061681450	6.308906990
	C60	-9.705168807	1.989236496	6.705146542
	C61	-9.831607343	2.843573567	7.783839186
	C62	-11.645688583	2.928870940	6.513397879
	C63	8.447688163	4.838014627	1.775206704
	C64	9.638704689	4.246446468	1.043404112
	C65	7.304655639	3.793722604	1.682051205
	C66	6.050297855	4.231578652	2.400787644
	C67	5.881257988	4.005211743	3.779647524
	C68	5.012144951	4.879377829	1.709327368
	C69	4.714101157	4.397967557	4.445145035
	C70	3.849497485	5.291610782	2.361989752
	C71	3.697416605	5.043086538	3.729284889
	C72	1.482118905	9.131909336	-0.260025109
	C73	0.025275007	9.564375687	-0.049011156

	C74	1.583636219	7.597990833	-0.242089138
	C75	-2.013495198	10.433667761	-1.102641554
	C76	-3.035499575	9.304482742	-0.966602342
	C77	-3.474011220	6.883885708	-1.139789946
	C78	-3.426184130	6.285986602	0.281994368
	C79	-3.112120723	5.860572545	-2.234304955
	C80	-3.923983661	4.562958044	-2.297149176
	C81	-5.371900725	4.654167218	-2.812281451
	C82	-7.132764274	5.870720158	-1.508925670
	C83	-2.148955816	5.811316477	2.309199603
	C84	-2.546846444	6.847368401	3.382278186
	C85	-0.764950810	5.256530767	2.660911357
	C86	-2.334993039	9.174019997	4.111237828
	C87	-3.603150312	9.980970155	4.386614984
	C88	-5.972249393	10.358777735	3.813937953
	C89	-6.972682157	9.895352672	4.895449417
	C90	-6.673210788	10.424286922	2.434389806
	C91	-7.057155005	9.032873555	1.943888731
	C92	-7.590385935	8.111660235	6.472181274
	C93	-7.563789483	8.833190074	7.815774395
	C94	-7.227545924	6.615141091	6.670009019
	C95	-7.407468441	5.801661563	5.408360786
	C96	-8.673095508	5.304120398	5.059448037
	C97	-6.336205981	5.560710074	4.532384166
	C98	-8.874073766	4.605064481	3.866168229
	C99	-6.522352013	4.882363182	3.322868793
	C100	-7.795158216	4.415209372	3.001273379
	C101	2.032276111	0.077004620	-10.471909021
	C102	0.900485659	-0.467866138	-9.616401640
	C103	-0.473794457	-0.078699363	-7.586884118
	C104	-1.632428621	0.923116156	-7.544084891
	C105	-2.284994254	3.190618203	-6.866346699
	C106	-1.744741159	4.607196614	-7.043178788
	C107	0.114520644	6.169447537	-6.972850506
	C108	0.095657091	6.789510880	-8.355356205
	C109	-4.016913967	-2.769322740	-8.960594615
	C110	-5.230722336	-2.379347427	-8.106440305
	C111	-2.909041421	-3.348859445	-8.048027252
	C112	-1.837444941	-4.091449104	-8.869782470
	C113	-0.629404674	-4.411728226	-8.017126499
	C114	-6.284012385	-0.682313932	-6.682993636
	C115	-6.640615784	0.755034165	-7.014114301
	C116	-6.035455043	-0.949813391	-5.174835856
	C117	-4.632734568	-0.627633414	-4.716367162
	C118	-4.251884887	0.683116944	-4.376082475
	C119	-3.654452839	-1.637511659	-4.675786374
	C120	-2.926120220	0.974006624	-4.032725191
	C121	-2.327801266	-1.346139834	-4.339589253
	C122	-1.955660167	-0.036454459	-4.021591317
	C123	7.669240986	-0.801101075	-8.000450835
	C124	9.026353489	-0.448772620	-7.366966610
	C125	6.908316634	-1.842902592	-7.163954223
	C126	5.501448632	-2.129823224	-7.593745543
	C127	5.075484622	-2.613267012	-8.815258256
	C128	3.356485495	-2.525600095	-7.422083238
	C129	11.153045319	-1.187525483	-6.446835759
	C130	11.948569444	-0.192528094	-7.263640439
	C131	3.338163047	-5.879065580	-2.365473400
	C132	4.466787557	-4.887304247	-2.380594217

	C133	4.883117119	-4.417940282	-3.833701105
	C134	5.198935535	-5.666334228	-4.702694241
	C135	5.787102541	-5.289406074	-6.070835203
	C136	6.340004218	-6.453921011	-6.842289332
	C137	6.216702084	-3.622203241	-3.673053562
	C138	2.568911243	1.490311577	-3.085016157
	Fe139	2.809815169	0.831392663	-4.952172995
	Fe140	0.793157456	2.369412749	-1.532742875
	Fe141	3.445568988	2.925081541	-2.078935997
	Fe142	1.861200301	3.065388327	-4.127767673
	Fe143	1.512652388	4.767669954	-2.045903829
	Fe144	4.333309625	0.711159349	-2.885422310
	Fe145	2.080369191	-0.476872039	-2.899090427
	H146	-2.239670396	-9.435844109	-0.505976350
	H147	-3.119162786	-8.464438068	-1.478618148
	H148	-6.133654527	-8.805047037	1.552612666
	H149	-5.180515363	-9.259214579	-0.593270880
	H150	-4.299824265	-10.454376092	0.351050593
	H151	-5.146874044	-6.394120980	2.645284954
	H152	-4.508351311	-9.717454266	3.796382383
	H153	-6.380479334	-7.444787223	3.386777034
	H154	-3.150492143	-10.441624806	5.541837486
	H155	-2.060492801	-9.013130956	3.093465560
	H156	-3.414395178	-8.887378692	6.364844956
	H157	-0.122420818	-4.220920605	2.867840328
	H158	-1.795921066	-6.573266239	2.723752813
	H159	0.114779831	-8.652816717	2.329007962
	H160	-1.819562218	-3.611001759	7.258973809
	H161	-2.292587567	-5.734066200	5.356798472
	H162	0.752701364	-8.831901383	3.981692928
	H163	-1.902133226	-4.266079016	2.904907070
	H164	-0.298215821	-6.897649103	6.925814986
	H165	-2.457392089	-5.238107449	7.607159039
	H166	2.698540179	-4.106571563	2.556879615
	H167	2.001039997	-2.936944795	1.407389546
	H168	3.709078829	-2.806664970	1.873940569
	H169	1.297337211	-2.423598110	3.747809224
	H170	1.334802508	-7.843821622	8.247383373
	H171	-0.425472253	-0.258611516	5.474071532
	H172	1.491916576	-2.724922434	8.696879283
	H173	0.006988729	0.143786408	9.794308959
	H174	1.471759113	-0.570319545	2.137460494
	H175	3.183998523	-0.326717471	2.513847852
	H176	8.727365647	4.941040959	2.841938626
	H177	-1.726459545	0.402610977	6.485882470
	H178	-1.453307549	0.684417288	8.936015544
	H179	-0.254626971	1.366301929	6.194129534
	H180	0.063732747	1.603231096	8.774662497
	H181	-0.413715191	-1.267918898	7.779287209
	H182	1.954148096	0.593860641	7.201804943
	H183	1.144937505	-1.991159649	6.083966984
	H184	1.940728770	0.022800365	3.741520250
	H185	-0.449127711	4.563638679	1.863814751
	H186	-0.874882757	4.686614763	3.598879318
	H187	0.945004783	5.977082234	3.293885972
	H188	2.509625252	5.187165908	5.259079611
	H189	3.045285308	5.769621712	1.802150401
	H190	5.092983692	5.034571219	0.631226860
	H191	6.669010079	3.504000332	4.349460891

	H192	2.349010291	-4.025225474	5.487200225
	H193	4.227963831	-6.589187101	4.870625672
	H194	4.898600645	-6.058393959	6.428861776
	H195	2.022045091	-6.346069361	5.758620142
	H196	1.338533845	-6.405779476	9.296092052
	H197	4.593813238	4.193926587	5.512516739
	H198	4.296069307	-2.140071572	4.264311550
	H199	2.185466555	-2.021945428	11.309174140
	H200	2.559804486	-3.576702638	10.519944212
	H201	4.699997242	-2.159312679	9.548073962
	H202	13.703725011	-1.280047907	-0.864140226
	H203	-0.315763109	-5.156059643	-5.176507638
	H204	4.658442692	-7.260526631	-2.120405175
	H205	-2.256637701	-5.040043195	-9.235969792
	H206	-1.537001010	-3.485136702	-9.736599658
	H207	-3.345226435	-4.047430028	-7.317956924
	H208	-6.515673664	1.018281705	-8.090762913
	H209	-4.390905396	-3.592471284	-9.589132927
	H210	-2.710271961	-3.127922263	-2.048955367
	H211	-1.713625087	-5.997399868	-1.657958562
	H212	-4.388200760	-0.534121716	-7.676998575
	H213	-7.172837731	-1.271567859	-6.981986529
	H214	-3.094969493	-0.951529401	-9.353552110
	H215	0.519400407	-5.064057251	-3.625286603
	H216	-2.456742582	-2.528067549	-7.468637561
	H217	-2.989944630	-2.042757367	-10.581502827
	H218	-5.272196318	-5.756637783	-2.212333477
	H219	-2.163553896	-3.649999955	-0.445619939
	H220	0.272794253	-2.848316256	-8.805826158
	H221	-0.538478919	-4.691030209	-1.685152289
	H222	-3.310850559	0.180535800	4.403645835
	H223	-1.938764660	-0.523555228	2.226186181
	H224	-5.798886505	-3.651702407	5.528356957
	H225	-5.679402386	-3.548073178	2.544616881
	H226	-6.706627170	-0.048300732	5.130763849
	H227	-9.167116841	3.090715368	8.604176253
	H228	-6.187490469	0.641971163	8.125289945
	H229	-8.634116421	0.123907104	6.890677110
	H230	-7.139091808	2.671064090	6.014472491
	H231	-12.631896281	3.231335586	6.172637894
	H232	-4.244018833	2.133188943	3.088046314
	H233	-5.609848324	1.134722201	2.585444267
	H234	-11.479267301	4.119798735	8.269317423
	H235	-8.781456316	0.783820963	5.259773241
	H236	-5.611623903	-1.258573490	3.257503866
	H237	-4.410994925	-4.189931564	4.576336390
	H238	-9.183342720	-3.348134602	2.519461377
	H239	-3.272053423	0.772858908	-1.590523595
	H240	-8.559255665	-4.985591068	2.237998576
	H241	8.714838854	6.834391219	1.393467319
	H242	7.144756659	6.388629398	1.482137087
	H243	-1.374600185	6.531732901	0.419668412
	H244	-2.869365780	4.987126655	2.406312757
	H245	-1.871843075	8.389651290	2.205219657
	H246	-4.992305546	1.625912473	0.178954373
	H247	-7.051503298	1.721642379	-0.372929463
	H248	-6.991930309	2.833100224	0.768093369
	H249	-6.479178035	-0.659633855	-2.069580264
	H250	-7.955992044	-1.625139792	-1.781144495

	H251	-5.998638216	10.924497555	1.724453333
	H252	-7.583660198	11.026031792	2.548264048
	H253	-5.366857049	8.900402121	0.694538107
	H254	-6.253285472	7.427362785	0.956806863
	H255	-7.772268863	7.709057453	-2.134999904
	H256	-5.628108329	3.735175797	-3.358579857
	H257	-9.862294759	4.208030359	3.621307377
	H258	-5.326384389	5.898011989	4.781199700
	H259	-7.840048476	5.174895966	0.309772693
	H260	-2.054170187	5.578646033	-2.112944656
	H261	-1.619763645	7.892344888	-1.493853610
	H262	-3.911224187	4.050157058	-1.322673415
	H263	-7.183774001	-4.178390227	0.519303024
	H264	-2.252476644	10.996194058	-2.015179356
	H265	-5.858145856	-3.279095997	-1.822820231
	H266	-3.184065091	6.380782151	-3.204129353
	H267	-5.690612103	4.755065787	2.626701139
	H268	-8.082852336	-0.117293435	0.195484100
	H269	-2.179358861	11.100216316	-0.245015104
	H270	-6.852151678	6.839202978	-3.300844032
	H271	-8.579876333	6.639984959	-0.265201111
	H272	-4.079079761	-5.011325872	-3.111321087
	H273	-3.807491444	-5.505338962	-0.462698322
	H274	-9.520237398	5.458331206	5.731990494
	H275	-5.661025958	-1.186066784	0.216572395
	H276	-4.509491542	7.227424324	-1.265576890
	H277	-5.466013317	5.472239756	-3.538151504
	H278	-3.374043413	3.910663756	-2.990900577
	H279	-6.463403994	3.996274678	-1.106855516
	H280	-2.053838923	8.696222769	5.062096048
	H281	-4.654797544	8.857105796	3.028548384
	H282	-5.688324971	11.381589940	4.102956368
	H283	-1.549803608	9.898330245	3.857415821
	H284	-5.823526362	8.247384103	5.257828258
	H285	2.017591688	1.176581245	-10.453821366
	H286	2.970279717	-0.237146067	-9.968432650
	H287	7.099075690	0.139082034	-7.998855707
	H288	2.795159869	-0.250596436	-12.333211688
	H289	1.757230343	-1.413348759	-11.825837887
	H290	2.630685947	7.326809799	-0.429024087
	H291	1.971553893	9.474437467	1.687706340
	H292	-0.140377493	10.011934321	-2.046414357
	H293	1.289445263	7.231262007	0.751710875
	H294	2.389945899	10.702433742	0.694338060
	H295	1.165251685	6.136165602	-6.643015631
	H296	1.827513942	9.496469142	-1.240247293
	H297	8.928425124	-3.990995385	1.439501417
	H298	10.056339217	-3.690372419	-1.393283760
	H299	0.246412206	4.056601704	-6.719848289
	H300	5.016518783	1.896524971	2.141950659
	H301	-3.057098075	3.028554081	-7.628244886
	H302	-1.578824665	-2.140610878	-4.339045732
	H303	-2.795124988	3.179408426	-5.887874361
	H304	6.898235251	-2.324302304	1.901440329
	H305	-0.403743808	2.172582257	-6.495628104
	H306	6.338341738	-2.562057432	0.248028658
	H307	8.063003749	-4.223524663	-0.094509923
	H308	8.536644370	-1.741943126	-0.630325765
	H309	9.136757441	-1.561751654	1.029354129

	H310	-6.249926970	-2.012438364	-4.990600953
	H311	10.526411542	-5.792445580	0.694626639
	H312	10.170886489	-8.212840800	-1.154430925
	H313	-0.917896449	0.199452582	-3.773872356
	H314	12.214387928	-7.685007738	0.860828810
	H315	10.956633411	-8.732698665	0.900964522
	H316	6.522356629	-0.141169507	-0.127936450
	H317	-2.619931209	1.993765307	-3.784279341
	H318	-3.930913222	-2.667465346	-4.914875258
	H319	10.016565944	-4.626229981	-5.933403509
	H320	7.474530111	-2.791387184	-7.185976049
	H321	8.832881966	-3.947039753	-3.886851296
	H322	9.285490376	-5.405438929	-7.047941423
	H323	-0.050057304	6.068038752	-9.195228986
	H324	7.362833673	-1.272385066	-3.246883769
	H325	5.355740756	-5.289186270	-1.873019856
	H326	1.095203396	1.123806815	-8.334875807
	H327	5.920452688	-6.297207977	-4.160144859
	H328	3.112253344	-3.244206736	-9.394207529
	H329	2.348640284	-2.642934276	-7.043174765
	H330	11.895549240	-7.969271065	-1.445089293
	H331	5.107284423	1.449467416	0.448946526
	H332	9.723688568	-1.102383958	-2.918473776
	H333	7.527040332	0.230407462	-2.896884775
	H334	11.364332747	-1.938854456	-1.424219200
	H335	6.847099723	-0.835451367	3.368362131
	H336	5.884198190	-8.156562292	-7.530932006
	H337	5.007038666	-4.802555264	-6.675178307
	H338	6.611787979	-4.577563272	-5.953037165
	H339	4.272626337	-6.241254660	-4.844369003
	H340	0.011721097	-0.160179809	-6.601065621
	H341	-4.997768489	1.481905661	-4.400681239
	H342	-6.777519719	-0.352542007	-4.622094605
	H343	4.130479593	-3.982509070	-1.857145390
	H344	10.381232131	-2.385852889	-3.495021127
	H345	10.195013493	-4.624154892	-3.564474284
	H346	5.533460105	0.207676455	3.796707541
	H347	5.615199948	-2.795309575	-9.736305683
	H348	6.894422873	-1.520963714	-6.115288887
	H349	-0.881111761	-1.051594008	-7.870377083
	H350	-0.429747081	6.824803760	-6.276014752
	H351	7.683551460	2.855130255	2.114870002
	H352	7.077411721	3.607154163	0.620248480
	H353	8.265912672	-0.471915339	-9.936181850
	H354	8.453893771	-2.026418193	-9.463087098
	H355	11.729189208	-2.126536774	-6.427461957
	H356	9.585094244	-2.454659526	-7.136807482
	H357	11.714286873	-0.187224118	-8.355023111
	H358	11.062775251	-0.830991031	-5.408303857
	H359	9.660747978	4.474685158	-0.049790504
	H360	-6.664469490	9.468438314	7.998018278
	H361	13.367725535	-1.766593268	0.816427782
	H362	11.466538774	0.246234378	0.578884571
	H363	-8.619100408	8.189191936	6.086115742
	H364	-6.188122587	6.542100736	7.026872220
	H365	-7.872696424	6.229611158	7.472997385
	H366	-8.887209120	3.447642347	1.708350292
	H367	1.416462696	-1.839194026	-2.630507563
	H368	5.450160386	-1.377315168	-1.691271669

	H369	1.483389476	-0.106447360	-0.113366863
	H370	-1.388970843	-0.350302973	-0.289120143
	Mo371	4.108074687	-1.553048357	-4.438198924
	N372	-3.129206723	-9.372569133	-1.008137958
	N373	-5.278547572	-8.362104900	1.880145591
	N374	-4.082499118	-8.993585186	4.370687145
	N375	-1.337521183	-8.892713348	3.802018148
	N376	-0.993462880	-6.121288429	3.163358214
	N377	-1.655341453	-5.013229555	5.703695074
	N378	0.204093784	-6.249665408	7.538560686
	N379	2.822862708	-6.128026466	6.358823908
	N380	3.232379150	-3.649918731	5.147143454
	N381	1.854441664	-1.316371916	6.378146641
	N382	2.111256603	-2.068180639	9.176912039
	N383	11.227890919	-7.791187972	0.614323758
	N384	10.456911218	-5.358394158	-0.232252000
	N385	6.580115969	-0.560123491	0.804847858
	N386	6.075959278	-0.249081803	3.068114778
	N387	5.170262712	1.161180430	1.454163618
	N388	11.804045632	-2.127340649	-0.497079882
	N389	-4.732523107	-4.894987783	-2.314554964
	N390	-0.354830288	-5.078690524	-4.154697815
	N391	-5.744759480	-2.971815075	-0.842269378
	N392	-7.597539439	-3.494944587	1.149321937
	N393	-6.100065658	-3.557691647	3.482918944
	N394	-4.934395009	-1.061257616	3.992581371
	N395	-4.150699023	1.032013011	0.379734297
	N396	-2.305400531	-0.075027106	0.144582435
	N397	-6.310197284	0.793437500	5.540050176
	N398	-10.845360690	2.051851090	5.918873492
	N399	-11.070459417	3.433068235	7.641017789
	N400	8.059714723	6.094130989	1.130382940
	N401	2.376686464	9.681086183	0.768695763
	N402	-0.623965534	10.028234693	-1.152256992
	N403	-2.618812414	8.059657358	-1.285251299
	N404	-6.380131516	4.799268426	-1.754864369
	N405	-7.140884094	6.939291600	-2.333656267
	N406	-7.862665181	5.930058254	-0.377527633
	N407	-2.235415768	6.314121099	0.939089704
	N408	-2.386028323	8.163733994	3.062678095
	N409	-4.742016670	9.575937106	3.749967839
	N410	-6.091029733	8.397795933	1.223927762
	N411	-6.679986515	8.729210050	5.521948361
	N412	1.905458733	-0.399991179	-11.852459594
	N413	0.528105218	0.302406531	-8.575976913
	N414	-1.330548865	2.100231243	-6.955894956
	N415	-0.409643607	4.815423991	-6.948335226
	N416	-3.615045062	-1.679847729	-9.858875058
	N417	-5.214711155	-1.129888244	-7.561662336
	N418	7.817207538	-1.225075634	-9.406845858
	N419	4.402245620	-2.072728219	-6.734607345
	N420	3.725868344	-2.856539825	-8.683079816
	N421	9.861631325	-1.476418106	-7.047814624
	N422	-1.435252085	2.882697620	0.258060878
	N423	-0.574813596	2.658163868	-0.450059518
	O424	-3.095368686	-7.886928406	1.444400055
	O425	-3.924088401	-6.760452387	4.805515195
	O426	-0.914830532	-8.617602205	6.033141984
	O427	1.118334849	-6.335400961	3.980889059

	O428	-0.228867537	-3.311175703	5.215776128
	O429	0.191890209	-4.251963471	8.642646759
	O430	3.706768967	-6.231831710	8.475780668
	O431	5.419246853	-4.170101917	4.669076162
	O432	3.905820556	-0.394073916	5.964419453
	O433	2.881747400	0.051677267	9.438124870
	O434	4.766261726	-2.075580532	11.588510269
	O435	10.820035547	-5.869145986	-2.459623053
	O436	11.802850992	-3.442488453	1.390052813
	O437	13.314663529	1.048368279	0.268537152
	O438	-4.433878083	-3.412524192	0.989814906
	O439	-2.628123645	-5.045133532	-4.204938929
	O440	-7.816746371	-1.271542636	1.624057191
	O441	-7.783671300	0.159776601	-0.706573790
	O442	-7.989923575	-3.939711271	4.738121278
	O443	-3.735094458	-1.914733593	5.741740566
	O444	-4.426233512	2.067486215	5.765833854
	O445	-6.718157532	2.555887880	8.585480176
	O446	10.496327546	3.552454856	1.570239619
	O447	2.512783253	5.440217490	4.317590242
	O448	-0.531956930	9.503359848	1.062846310
	O449	-4.196183393	9.584353239	-0.600449644
	O450	-4.464030102	5.823047126	0.788778152
	O451	-2.951573042	6.465669168	4.490024487
	O452	0.165695157	6.331474371	2.817805853
	O453	-3.570982527	10.933337831	5.176354620
	O454	-7.976885332	10.572514409	5.152708536
	O455	-8.120718157	8.483523865	2.272584844
	O456	-8.445241318	8.713013337	8.650328326
	O457	-7.967225103	3.771467752	1.761330766
	O458	0.391417763	-1.587960415	-9.877005913
	O459	-2.750328616	0.654792125	-8.035198174
	O460	-2.551384051	5.537582467	-7.217809433
	O461	0.266150903	7.981208768	-8.556764066
	O462	-6.163464719	-3.176650487	-7.911458958
	O463	-0.592350211	-5.347787708	-7.223315823
	O464	0.426557736	-3.582149833	-8.130388623
	O465	-7.059595637	1.571805562	-6.207701022
	O466	9.387724050	0.733399134	-7.216236769
	O467	12.840412416	0.504147289	-6.799065923
	O468	2.149462642	-5.612967739	-2.472118226
	O469	3.689190257	-7.197623015	-2.220769154
	O470	7.485380002	-6.541617910	-7.278331166
	O471	5.431020953	-7.446905545	-7.027223890
	O472	6.174166929	-2.390253758	-4.063014503
	O473	7.242712771	-4.192148787	-3.232188966
	O474	3.896266225	-3.636117558	-4.401969738
	O475	10.604005578	-1.546131734	-3.011727887
	O476	7.915491674	-0.646575675	-2.709926278
	O477	-6.532708889	2.505562124	-0.043260468
	O478	10.103365407	-4.892417889	-6.883590276
	O479	9.789195730	-3.956608099	-4.176188950
	S480	0.497367008	6.778240306	-1.502204355
	S481	-0.150140969	3.588859323	-3.190538834
	S482	1.792865765	-1.186817659	-4.929289652
	S483	2.233752178	3.532104250	-0.311499546
	S484	0.667615342	0.090478377	-1.190990504
	S485	1.936649369	2.404639049	-6.286208420
	S486	4.108267756	-1.456025185	-1.977462826

	S487	3.337008916	4.706025351	-3.417214482
	S488	5.080043587	0.550823322	-4.983313513
	S489	5.476830597	2.098278810	-1.614191633

12 35 (S=1)

**12 35 (S=1)**	bm22b2n2x6x3b5635tk.car_3			
Fe(139) -2.312	C1	-7.540879146	-5.251923923	2.439387733
Fe(140) -1.137	C2	-7.981651403	-6.397439804	1.523123140
Fe(141) -2.333	C3	-8.246911043	-3.278867925	3.717311926
Fe(142) 2.798	C4	-7.572133574	-3.562216832	5.058691729
Fe(143) 2.893	C5	-7.250492781	-5.120155864	6.913658297
Fe(144) 2.279	C6	-5.755968384	-5.430304178	6.774747130
Fe(145) -0.572	C7	-3.959518172	-6.174295657	5.285035808
	C8	-3.167959723	-4.880628890	5.074432495
	C9	-3.140123648	-2.612767939	4.186055130
	C10	-2.977717662	-1.847986790	5.508221163
	C11	-3.938037710	-1.217050792	7.646441132
	C12	-2.950078619	-1.842967151	8.638393410
	C13	-2.213782312	-3.879215704	9.777203076
	C14	-0.772204818	-4.239377090	9.398104873
	C15	0.733648860	-5.026662092	7.621163328
	C16	1.580201856	-4.081515639	6.766856297
	C17	1.644864559	-1.934899991	5.538889278
	C18	1.864095115	-0.604304865	6.281539829
	C19	0.922296930	-1.754921142	4.175806129
	C20	1.533703650	-0.614155745	3.353940794
	C21	0.928923684	-3.064997386	3.380143646
	C22	0.810480301	1.284389150	7.477132099
	C23	1.198500595	1.048180477	8.950803960
	C24	-0.510612889	2.077172617	7.327415515
	C25	-0.522533501	3.321084477	8.221754130
	C26	-0.747968473	2.469200533	5.863133393
	C27	0.740306243	-0.096723693	11.042321775
	C28	2.190823324	-0.429093257	11.326354155
	C29	6.516935537	-11.348286279	3.073423434
	C30	6.934103512	-10.119051889	2.269304340
	C31	7.349341012	-7.662289821	2.484307635
	C32	8.719664567	-7.274887595	3.074454319
	C33	6.264444585	-6.636200917	2.861016952
	C34	6.584423162	-5.204701493	2.422875473
	C35	5.358868476	-4.305449368	2.575962505
	C36	5.283776976	-1.804007960	2.753931814
	C37	10.973180880	-6.465305263	2.554768603
	C38	11.242102624	-4.975803421	2.483866366
	C39	-5.542866199	-2.667728016	-0.611820102
	C40	-5.821725963	-1.226716503	-0.150106916
	C41	-4.016764090	-2.813965174	-0.802067936
	C42	-3.488407029	-4.253880096	-0.978280084
	C43	-3.445321584	-4.756292609	-2.420421012
	C44	-6.550267007	0.981975665	-0.932765274
	C45	-7.605614682	1.411441197	0.091522828
	C46	-6.801552858	1.694960288	-2.277752764
	C47	-9.266712580	0.731195904	1.742612423
	C48	-8.530482599	0.888140223	3.080210662
	C49	-6.428487472	0.583274693	4.319913765
	C50	-5.230645571	1.531756491	4.288484892
	C51	-4.003842193	3.287923638	3.077323231
	C52	-4.271244892	4.619468089	3.817044020

	C53	-3.715300807	3.670381396	1.617885187
	C54	-3.150481985	2.599610008	0.742460069
	C55	-2.405287277	1.470585678	0.982507928
	C56	-2.609960571	1.680378790	-1.218028151
	C57	-6.008089855	6.270028382	4.390030416
	C58	-5.606313148	6.490744052	5.846062773
	C59	-7.543852633	6.350874444	4.222580537
	C60	-8.136005361	7.729332668	4.166708635
	C61	-7.919357717	8.840572983	4.960353517
	C62	-9.425336692	9.288751669	3.397429531
	C63	9.620916736	1.217726245	2.001233934
	C64	10.454268684	-0.004843529	1.662337596
	C65	8.136131136	0.768509044	1.976196021
	C66	7.175470992	1.880874396	2.323690780
	C67	6.856465060	2.180133888	3.660145890
	C68	6.565906012	2.644577166	1.312622119
	C69	5.953572018	3.200756747	3.981161041
	C70	5.673978546	3.673738295	1.617242334
	C71	5.364203661	3.946627385	2.952083462
	C72	5.412999038	7.164767045	-2.247898691
	C73	4.292874187	8.199095503	-2.414853039
	C74	4.821340674	5.809561860	-1.827298801
	C75	2.911736626	9.473052958	-3.994876244
	C76	1.489586621	8.959114327	-3.765695481
	C77	0.028525133	7.006572076	-3.413485134
	C78	-0.273493959	6.904487246	-1.903884156
	C79	-0.047948863	5.647948592	-4.135037247
	C80	-1.346030196	4.843551462	-4.010654259
	C81	-2.577228634	5.353174225	-4.784781110
	C82	-3.670710991	7.521968778	-4.159035912
	C83	0.551064797	6.585589998	0.373573143
	C84	0.602744780	7.961789769	1.071020286
	C85	1.520270727	5.640604517	1.091351824
	C86	1.787901551	10.099103777	1.220369360
	C87	0.995337370	11.396428056	1.059145094
	C88	-0.928247840	12.520221637	-0.003940876
	C89	-2.086016249	12.858405732	0.959931387
	C90	-1.451767409	12.446255666	-1.459648755
	C91	-2.383944295	11.255732941	-1.651732197
	C92	-3.514669212	12.055088628	2.796758692
	C93	-3.246926942	13.076688769	3.896632076
	C94	-3.865415762	10.675151213	3.417040929
	C95	-4.319784970	9.670645895	2.381636079
	C96	-5.655515873	9.653243106	1.950337228
	C97	-3.422700884	8.762001466	1.798994327
	C98	-6.084336824	8.769343111	0.957009899
	C99	-3.827752375	7.884949296	0.786334622
	C100	-5.158668288	7.902610423	0.374854502
	C101	2.400645177	-3.924457962	-9.618133246
	C102	1.098496877	-3.666904856	-8.877227594
	C103	-0.069894589	-2.160288063	-7.290755621
	C104	-0.650768519	-0.803705219	-7.704226468
	C105	-0.240524370	1.616212149	-7.725784453
	C106	0.886193642	2.545615724	-8.169845527
	C107	3.238137528	3.150414368	-8.155628152
	C108	3.529451678	3.296438343	-9.633921259
	C109	-4.347711142	-3.426119550	-8.571593582
	C110	-5.307077294	-2.335509620	-8.077151886
	C111	-3.665683006	-4.106594010	-7.362030376

C112	-3.003187189	-5.437493226	-7.767354824
C113	-2.107449078	-5.948000028	-6.660543374
C114	-5.567225424	-0.013751617	-7.327316516
C115	-5.234065633	1.269142053	-8.065700563
C116	-5.545834630	0.105417167	-5.781276166
C117	-4.174084454	-0.053959813	-5.170142510
C118	-3.268039461	1.020375008	-5.106562194
C119	-3.750625692	-1.309554234	-4.700641060
C120	-1.973654870	0.834787848	-4.604415570
C121	-2.455789528	-1.496866607	-4.203052341
C122	-1.557476607	-0.425771490	-4.159614449
C123	6.906562312	-6.258274330	-6.040720088
C124	8.239886847	-6.317663579	-5.277585213
C125	5.719028058	-6.587953092	-5.120600352
C126	4.357554656	-6.391984124	-5.714031922
C127	3.829219814	-6.996264898	-6.837503044
C128	2.257897414	-5.793286171	-5.840982287
C129	9.767125167	-7.548787150	-3.844386380
C130	10.964927688	-7.254992610	-4.721229417
C131	0.471913970	-7.169729834	-0.208651065
C132	1.928907118	-6.794109380	-0.250508521
C133	2.586912937	-6.976112627	-1.677033963
C134	2.360950740	-8.439221612	-2.152349166
C135	3.130955811	-8.769162845	-3.439394651
C136	3.147134039	-10.230641618	-3.790505747
C137	4.122702291	-6.793316508	-1.475908906
C138	3.119675508	-0.695483738	-2.842565275
Fe139	3.131579473	-1.958372342	-4.375145176
Fe140	1.852816474	1.135831422	-1.942753990
Fe141	4.530942590	0.468269939	-2.110455155
Fe142	3.279834419	0.581871730	-4.361313481
Fe143	3.603564264	2.852561505	-2.852920591
Fe144	4.416861130	-2.038319933	-2.136058893
Fe145	1.843991737	-2.218472905	-2.175641492
H146	-6.177709140	-7.344331588	1.452788028
H147	-6.491075262	-6.445680173	0.126716874
H148	-9.482118334	-4.582284924	2.558147107
H149	-8.727524148	-6.019984332	0.806466047
H150	-8.513495337	-7.126353916	2.159273845
H151	-7.575724986	-2.596666733	3.176383564
H152	-8.555757302	-5.355607881	5.216582516
H153	-9.192123204	-2.753165526	3.907253586
H154	-7.759570345	-6.012708780	7.300092813
H155	-6.025030905	-5.955898322	4.807727108
H156	-7.346103971	-4.318836533	7.657658147
H157	-2.135450367	-2.730862786	3.759566360
H158	-4.684826431	-4.087991374	3.910073047
H159	-3.893922175	-6.792281459	4.379376824
H160	-3.625282592	-0.176984829	7.485554489
H161	-4.887443627	-2.382005728	6.107802571
H162	-3.479438056	-6.715403478	6.108788416
H163	-3.744686086	-2.017315033	3.486895672
H164	-3.711439310	-3.731056226	8.258979871
H165	-4.938636580	-1.214653273	8.100117727
H166	0.443994064	-3.879922652	3.936079486
H167	0.394244719	-2.932855334	2.427320504
H168	1.958088015	-3.374705926	3.139547941
H169	-0.123241524	-1.487749902	4.398908454
H170	-2.729413404	-4.813782973	10.040979076

	H171	-0.774909991	1.594538944	5.198195468
	H172	-0.343096370	-0.330363668	9.224215785
	H173	-0.433843136	3.070056948	9.288719098
	H174	0.948117674	-0.465026383	2.434092897
	H175	2.564753125	-0.840238953	3.047047831
	H176	9.865539209	1.507499666	3.041795205
	H177	-1.709538825	2.993258127	5.766259628
	H178	-1.465040539	3.869845078	8.079282702
	H179	0.048492012	3.146043906	5.514821138
	H180	0.306444656	4.000107723	7.964995261
	H181	-1.335032114	1.415556639	7.649182657
	H182	1.639042482	1.878507443	7.064397451
	H183	-0.176083421	-0.360548640	6.509668812
	H184	1.539921701	0.343296529	3.896670559
	H185	1.528943392	4.672319045	0.563012130
	H186	1.120079723	5.482958492	2.107057421
	H187	3.341651551	5.738835668	1.813475040
	H188	4.286348802	5.023096479	4.156972096
	H189	5.190131635	4.240658123	0.821693239
	H190	6.761313891	2.407021245	0.264729183
	H191	7.310736233	1.600253854	4.467942339
	H192	0.009992724	-2.779131471	6.645721599
	H193	0.576031372	-5.938636808	7.025633331
	H194	1.337385929	-5.296188014	8.497805957
	H195	-1.330728950	-4.573143430	7.426607734
	H196	-2.153217488	-3.256403920	10.678469413
	H197	5.696804124	3.402413556	5.024896705
	H198	2.651697573	-2.334019180	5.358338559
	H199	0.446454283	0.768676090	11.658335829
	H200	0.138017002	-0.960300872	11.365682514
	H201	2.738682625	-0.916347481	10.485682377
	H202	11.720577620	-6.992498805	1.942591597
	H203	-2.306845618	-5.859808063	-3.715919699
	H204	1.005717586	-8.843644435	0.579991654
	H205	-3.784044418	-6.189875776	-7.949326727
	H206	-2.425258203	-5.304601672	-8.693599357
	H207	-4.402278033	-4.301866661	-6.567312569
	H208	-4.947793497	1.116653802	-9.132890828
	H209	-5.013958160	-4.168843435	-9.037439763
	H210	-3.711184771	-2.193997927	-1.660495698
	H211	-4.116598036	-4.961741015	-0.410338646
	H212	-3.753342359	-0.965490240	-7.963211857
	H213	-6.608901019	-0.244235242	-7.627381763
	H214	-2.694650738	-2.355039904	-9.230318133
	H215	-1.599914870	-5.665842941	-2.111456318
	H216	-2.922141956	-3.413830226	-6.938263038
	H217	-3.018107195	-3.710343115	-10.107059821
	H218	-7.161012181	-3.460554210	-1.619305574
	H219	-3.540249252	-2.383258195	0.090672340
	H220	-0.562705592	-5.215344169	-7.640576654
	H221	-2.484281070	-4.322749494	-0.536428647
	H222	-3.112711121	2.852308357	3.550066324
	H223	-2.069942292	1.022058126	1.910733007
	H224	-7.089479546	0.925024203	5.128952269
	H225	-6.791841062	0.052945660	2.287524245
	H226	-6.286964483	4.254310075	3.718293797
	H227	-7.258614323	9.021962760	5.800686759
	H228	-5.648202763	5.567963691	6.474089271
	H229	-8.026782025	5.742488485	5.007686033

	H230	-5.517610143	7.039267485	3.773693949
	H231	-10.148296722	9.857462112	2.818143990
	H232	-3.004828038	4.512583418	1.642722376
	H233	-4.640919815	4.059837493	1.161955767
	H234	-8.829746538	10.768533182	4.808243170
	H235	-7.790697725	5.870795088	3.262900564
	H236	-5.760173494	2.222250181	2.418300984
	H237	-6.057309719	-0.410483186	4.609104787
	H238	-9.826397347	1.661258110	1.579203082
	H239	-2.505531464	1.500479137	-2.284270462
	H240	-9.992537669	-0.086449586	1.842533829
	H241	10.773671695	2.701966432	1.182540769
	H242	9.170378485	3.009503155	1.121275837
	H243	1.663436901	6.323589080	-1.465538453
	H244	-0.466401890	6.203937281	0.537013762
	H245	1.950551195	8.657699049	-0.313860447
	H246	-3.747571911	3.488891815	-1.128866986
	H247	-5.523516340	4.265072956	-2.047506394
	H248	-5.037895247	5.538030024	-1.219553987
	H249	-5.977847147	1.469792726	-2.969662231
	H250	-7.743180281	1.331665596	-2.722542633
	H251	-0.584543275	12.393660732	-2.134551382
	H252	-2.007928102	13.369821559	-1.662476593
	H253	-0.863954539	10.072685845	-2.497410253
	H254	-2.322171607	9.244279071	-2.039567490
	H255	-3.367570360	9.184693872	-5.314808970
	H256	-3.185397527	4.496644143	-5.111767018
	H257	-7.130963542	8.759690630	0.642406923
	H258	-2.380198629	8.722042670	2.126481139
	H259	-4.707414032	7.759972848	-2.381448350
	H260	0.771899212	5.009230295	-3.770260912
	H261	2.154240852	7.003448324	-3.673370472
	H262	-1.613982610	4.693134603	-2.952837532
	H263	-8.308647084	-0.489486779	0.251375004
	H264	2.994828549	9.777283018	-5.047146379
	H265	-6.608685304	-0.964073990	-1.970623471
	H266	0.158871322	5.836285629	-5.201805021
	H267	-3.099752026	7.230080693	0.302313891
	H268	-7.289825810	3.272309252	-1.231418462
	H269	3.017574572	10.373546217	-3.374357056
	H270	-2.892868560	7.702642686	-6.053671476
	H271	-4.695021327	9.146527957	-3.428942428
	H272	-5.718453723	-3.571344190	-2.451166897
	H273	-5.836018721	-3.305504166	0.243218398
	H274	-6.382994578	10.332275867	2.401242469
	H275	-5.606504567	1.367682834	-0.512341433
	H276	-0.734671817	7.691005571	-3.807150643
	H277	-2.264565331	5.867154882	-5.703218159
	H278	-1.111329945	3.843855415	-4.405315347
	H279	-3.940687912	5.753919020	-3.199729897
	H280	1.783349415	9.865123176	2.296278537
	H281	-0.374881727	10.452582808	-0.141235784
	H282	-0.237985289	13.373999069	0.067733588
	H283	2.822873993	10.327482269	0.932186374
	H284	-1.806800767	11.083730361	1.927903915
	H285	2.877624724	-2.968696461	-9.880666249
	H286	3.066326081	-4.422089523	-8.882345771
	H287	6.811711023	-5.216645995	-6.379842372
	H288	3.042524742	-5.089487510	-11.163369235

H289	1.564735882	-5.505282691	-10.588586232
H290	5.649227736	5.092937208	-1.748150348
H291	5.899971424	7.841999961	-0.390550269
H292	4.443832666	8.055371383	-4.458779992
H293	4.342899674	5.917631184	-0.842489260
H294	6.868313677	8.436184988	-1.563668029
H295	4.154044874	2.768441479	-7.676081783
H296	5.933903785	7.035251671	-3.209348318
H297	6.110239248	-6.671751377	3.952747861
H298	7.419943552	-7.733476202	1.391971765
H299	2.397006118	1.376912392	-7.318415385
H300	5.222114719	0.219886382	2.510050577
H301	-0.973831473	1.573919949	-8.540619014
H302	-2.137304443	-2.485813129	-3.867195912
H303	-0.737349217	2.117882771	-6.877476246
H304	5.027839153	-4.273248592	3.621348733
H305	0.955689354	0.063125430	-6.789705812
H306	4.520095670	-4.713233881	1.986829067
H307	5.328283422	-6.972702574	2.386256079
H308	6.894272902	-5.192530151	1.366959404
H309	7.416297529	-4.794825241	3.017710194
H310	-6.216406236	-0.669114300	-5.383027271
H311	6.795595324	-9.103702117	3.985729026
H312	5.489476385	-11.582949885	2.756229619
H313	-0.537186411	-0.574598105	-3.796990742
H314	7.446326485	-11.373154755	4.911029260
H315	5.852059296	-11.744838315	4.978903166
H316	5.842132391	-2.829066842	1.118195412
H317	-1.259372198	1.661444614	-4.570131878
H318	-4.439188732	-2.157795644	-4.721702859
H319	7.197515496	-9.879111596	-2.712193248
H320	5.807040494	-7.640526542	-4.797391910
H321	6.326118416	-8.202798231	-1.144440666
H322	6.259187071	-10.593501531	-3.709458609
H323	3.161213251	2.456599226	-10.271512592
H324	6.155247492	-5.092857272	-1.417005047
H325	2.511935167	-7.372505973	0.481874395
H326	1.913370146	-1.995007980	-8.005970582
H327	2.694121286	-9.113156075	-1.348206465
H328	1.825187237	-6.900484621	-7.588500488
H329	1.287985508	-5.355649871	-5.637478240
H330	7.151493582	-12.181918063	2.728294682
H331	5.222921285	-0.695168634	1.018074678
H332	8.339858850	-5.828288048	-0.762590161
H333	6.968860485	-3.774328262	-1.484252253
H334	9.363362150	-6.775258061	1.158759714
H335	5.530425294	-2.520648434	4.668948040
H336	2.021067826	-11.713409900	-4.120362487
H337	2.681290070	-8.215235990	-4.277270796
H338	4.178689527	-8.459453573	-3.356720449
H339	1.285664993	-8.595909538	-2.317945841
H340	0.264411158	-2.144149440	-6.241050750
H341	-3.579732030	2.000922256	-5.474715235
H342	-5.978690330	1.086716991	-5.532651997
H343	2.009511682	-5.728305467	-0.004359696
H344	8.391339895	-7.375741728	-0.876381506
H345	7.231271136	-9.241835240	-0.425761087
H346	4.778831884	-0.964726746	4.572545063
H347	4.282348062	-7.653688138	-7.570584206

	H348	5.790838178	-5.980329790	-4.210454643
	H349	-0.852770094	-2.912484756	-7.405813766
	H350	3.030430946	4.144826910	-7.730042898
	H351	8.033847100	-0.061614439	2.692135101
	H352	7.902804411	0.377353441	0.972944145
	H353	7.690650468	-6.815673984	-7.852683594
	H354	7.136453587	-8.080867476	-6.977875729
	H355	9.871983110	-8.587694792	-3.492878776
	H356	7.841321342	-8.212509449	-4.471629057
	H357	10.818265770	-7.474687562	-5.805591751
	H358	9.783861967	-6.893355703	-2.958718209
	H359	10.664639191	-0.132213365	0.572964191
	H360	-2.172063641	13.324685333	4.066000878
	H361	11.117125240	-6.766669422	3.607117486
	H362	10.339743055	-4.319269242	2.535233757
	H363	-4.379040869	12.421370933	2.220035294
	H364	-2.989847005	10.295840268	3.966559922
	H365	-4.661699122	10.846149996	4.155739318
	H366	-6.502639383	7.129618648	-0.804292952
	H367	0.689491669	-3.079708448	-1.666866305
	H368	4.352596838	-3.010961064	-0.612603270
	H369	1.225633761	-0.652264389	0.203167327
	H370	-1.383370086	0.168556683	-0.426024120
	Mo371	3.205819421	-4.375257451	-3.106754692
	N372	-6.922361763	-7.081281378	0.802024540
	N373	-8.540312673	-4.462927828	2.923793524
	N374	-7.888151505	-4.732358082	5.664732717
	N375	-5.365376793	-5.951241128	5.584761139
	N376	-3.781671132	-3.908928369	4.350900711
	N377	-4.015299017	-1.919738410	6.376014336
	N378	-3.000969908	-3.194509593	8.764076154
	N379	-0.546401922	-4.520216321	8.082810333
	N380	0.985479443	-2.919515060	6.388884369
	N381	0.744567330	0.025756082	6.731892893
	N382	0.483985811	0.113893989	9.629145116
	N383	6.530794154	-11.131683171	4.525120426
	N384	6.973812972	-8.978210565	2.983531275
	N385	5.609916789	-2.936021351	2.108914401
	N386	5.109723886	-1.799055493	4.092280457
	N387	5.079205452	-0.678958081	2.052472820
	N388	9.626235399	-6.834539963	2.167006130
	N389	-6.293143142	-2.966719476	-1.835818475
	N390	-2.358471371	-5.471535048	-2.768194702
	N391	-6.420735106	-0.451037767	-1.093526516
	N392	-8.408459358	0.459729678	0.604099663
	N393	-7.220102363	0.504178816	3.105476656
	N394	-5.094610697	2.327607099	3.182394180
	N395	-3.267447136	2.696153279	-0.637898626
	N396	-2.096483089	0.919026546	-0.245492460
	N397	-5.580435274	4.943809750	3.961853412
	N398	-9.079645614	8.022972791	3.194775345
	N399	-8.746519434	9.819864104	4.453313771
	N400	9.864813531	2.265743058	1.008788044
	N401	6.403411336	7.581163429	-1.244854127
	N402	3.976987633	8.538043915	-3.695818228
	N403	1.324826177	7.620440173	-3.689852453
	N404	-3.472501544	6.213648337	-4.000678744
	N405	-3.158622149	8.197060770	-5.209121337
	N406	-4.349378849	8.214516111	-3.223018444

	N407	0.770156165	6.645162076	-1.070302712
	N408	1.348367546	8.932151920	0.468990219
	N409	-0.170286582	11.325035875	0.350487496
	N410	-1.761224921	10.094463186	-1.996037671
	N411	-2.372851645	11.928798770	1.903928506
	N412	2.154359657	-4.704465614	-10.834328001
	N413	1.052601486	-2.539801897	-8.141197018
	N414	0.119101264	0.254938251	-7.373461191
	N415	2.166356704	2.209071530	-7.877769931
	N416	-3.455648100	-2.923130768	-9.624065670
	N417	-4.763657401	-1.103703751	-7.858026136
	N418	6.924643008	-7.113814175	-7.242279040
	N419	3.355723638	-5.634236824	-5.105347601
	N420	2.508642647	-6.605167182	-6.897126045
	N421	8.517833167	-7.455915476	-4.581191382
	N422	0.021910547	3.106938729	-0.800184454
	N423	0.716819977	2.324294428	-1.239932083
	O424	-6.353244576	-5.078610870	2.770601013
	O425	-6.783981481	-2.741299606	5.555719057
	O426	-4.974845614	-5.241391573	7.721883153
	O427	-2.030179491	-4.730741757	5.547109419
	O428	-1.949048204	-1.198069969	5.756147835
	O429	-2.179272905	-1.133947341	9.307503535
	O430	0.098566581	-4.303127437	10.275329845
	O431	2.731068417	-4.415154520	6.448832011
	O432	3.008807775	-0.147291668	6.437675971
	O433	2.117065102	1.686653225	9.489463751
	O434	2.717663606	-0.247168891	12.413177563
	O435	7.174250002	-10.230603124	1.048153295
	O436	8.936703229	-7.368053503	4.295626831
	O437	12.364372037	-4.505160949	2.394093117
	O438	-5.524749939	-0.808825991	0.987970469
	O439	-4.375239829	-4.526124272	-3.227038395
	O440	-7.663764994	2.611685881	0.440565021
	O441	-6.855280462	3.119586028	-2.108691239
	O442	-9.140172128	1.340397811	4.056399483
	O443	-4.450079742	1.572442514	5.243127647
	O444	-3.341099157	5.361046616	4.145964720
	O445	-5.290348325	7.568957831	6.318660877
	O446	10.849576828	-0.814834244	2.488262384
	O447	4.456398683	4.958546393	3.199215769
	O448	3.705830649	8.704820244	-1.440087320
	O449	0.554537734	9.784539261	-3.699072754
	O450	-1.435525451	7.079786834	-1.495152478
	O451	0.011704833	8.129120127	2.147775268
	O452	2.825140291	6.225763785	1.137963458
	O453	1.410400450	12.436921521	1.585224178
	O454	-2.699910854	13.928665227	0.850287678
	O455	-3.597799201	11.320813761	-1.401918822
	O456	-4.134368389	13.582555658	4.564003289
	O457	-5.537952093	7.045872017	-0.675037690
	O458	0.157289328	-4.499667070	-8.941949895
	O459	-1.739773330	-0.711002952	-8.312393127
	O460	0.593920719	3.607189002	-8.748774258
	O461	4.150530504	4.235500808	-10.106714438
	O462	-6.508254153	-2.575946814	-7.870747531
	O463	-2.531716120	-6.519248547	-5.658757901
	O464	-0.788566453	-5.709105707	-6.789226207
	O465	-5.301309267	2.389062885	-7.580667612

	O466	9.075404560	-5.396922512	-5.344028059
	O467	12.046772547	-6.890491704	-4.283244745
	O468	-0.457769992	-6.471995321	-0.587080546
	O469	0.177675339	-8.411523087	0.295156131
	O470	4.154850300	-10.905021486	-3.990944027
	O471	1.902598902	-10.768918747	-3.882454217
	O472	4.654006373	-5.818217725	-2.133109225
	O473	4.759212497	-7.594660881	-0.750367278
	O474	2.075345314	-6.056865469	-2.575967957
	O475	8.937337467	-6.598047051	-0.583659376
	O476	6.925114215	-4.622536689	-1.001426525
	O477	-4.729630540	4.826049499	-1.830850966
	O478	7.208830957	-10.428611674	-3.536415807
	O479	7.197736086	-8.683618132	-1.245307979
	S480	3.557630071	5.166892085	-3.022780278
	S481	1.673393261	2.159172927	-3.964404921
	S482	1.312474784	-3.243915606	-4.039400677
	S483	3.577059117	1.994540318	-0.788254626
	S484	0.653874934	-0.637488596	-1.035601790
	S485	3.114704857	-0.670450911	-6.213470609
	S486	2.991070150	-3.501415521	-0.811979462
	S487	5.262830248	1.652357238	-3.858710981
	S488	5.043763177	-3.148735606	-3.965267028
	S489	5.972695786	-0.894438983	-1.086430245

TS 12 → 13 35 (S=1)

**TS 12 → 13 35 (S=1)**	bm22b2n2x6x3b135te_1_53121.667			
Fe(139) 0.539 -2.392	C1	-6.952182727	-6.073290478	2.123540919
Fe(140) 0.436 -1.398	C2	-7.235724674	-7.230067468	1.160004172
Fe(141) 0.549 -2.580	C3	-7.916499641	-4.240632051	3.442199935
Fe(142) 0.563 2.755	C4	-7.232405280	-4.492311857	4.784312855
Fe(143) 0.564 2.948	C5	-6.786292603	-6.055581290	6.610225859
Fe(144) 0.371 1.878	C6	-5.262584052	-6.186353891	6.506632833
Fe(145) 0.318 0.403	C7	-3.349970271	-6.676214298	5.057644839
	C8	-2.717471950	-5.291885832	4.893884533
	C9	-2.941775293	-3.017404639	4.045873230
	C10	-2.888592540	-2.268951018	5.385718746
	C11	-3.946508947	-1.802293596	7.520580453
	C12	-2.922772479	-2.348844305	8.523520092
	C13	-1.998055379	-4.328912047	9.625813638
	C14	-0.515437379	-4.522082904	9.282697440
	C15	1.126478682	-5.080405797	7.541222796
	C16	1.882903447	-4.014448102	6.744431174
	C17	1.723800232	-1.854687814	5.544829574
	C18	1.765498088	-0.531204392	6.329866255
	C19	1.018463684	-1.725815320	4.167837635
	C20	1.507761323	-0.502214406	3.384351758
	C21	1.195729994	-3.008813666	3.347883684
	C22	0.477911453	1.200096106	7.528962425
	C23	0.853185299	0.981280174	9.008122696
	C24	-0.913399859	1.852990714	7.353729847
	C25	-1.079953484	3.068653584	8.273630096
	C26	-1.143561328	2.252491322	5.890514148
	C27	0.496250085	-0.257464111	11.064089409
	C28	1.972551330	-0.426409390	11.361207914
	C29	7.637027884	-10.620771842	3.008379979
	C30	7.941077010	-9.337223347	2.237115806
	C31	8.072969401	-6.854393784	2.508213093

C32	9.373580756	-6.339017414	3.153499083
C33	6.869264537	-5.962339332	2.858689891
C34	7.037131916	-4.498373046	2.441784814
C35	5.705056628	-3.755753867	2.536882735
C36	5.385384592	-1.280374406	2.754040658
C37	11.545354100	-5.283968239	2.735993270
C38	11.652277269	-3.775035899	2.665600551
C39	-5.170626213	-3.229884773	-0.821335046
C40	-5.631780698	-1.846590279	-0.333489951
C41	-3.632809327	-3.193755359	-0.974742869
C42	-2.942067762	-4.562765799	-1.150877846
C43	-2.807881461	-5.044290066	-2.594550925
C44	-6.589133385	0.283734402	-1.088106447
C45	-7.710713219	0.570878329	-0.087572663
C46	-6.878331480	0.994582953	-2.426661314
C47	-9.324874614	-0.319947844	1.510384025
C48	-8.635160414	-0.121597693	2.867487513
C49	-6.540179567	-0.242297615	4.147304818
C50	-5.478924479	0.856281782	4.187058428
C51	-4.405215580	2.752646848	3.048798379
C52	-4.855377182	4.023041146	3.809269197
C53	-4.104767965	3.207350066	1.612580495
C54	-3.441917678	2.218151321	0.709161966
C55	-2.598441279	1.152778519	0.914168868
C56	-2.803396529	1.428548071	-1.278844640
C57	-6.786380714	5.455606021	4.348305118
C58	-6.464454897	5.645138499	5.828105075
C59	-8.314769618	5.374974971	4.123014240
C60	-9.048368333	6.683660924	4.087375115
C61	-8.958472412	7.795897359	4.902727178
C62	-10.492229545	8.111214369	3.334992988
C63	9.389453170	2.260369879	2.304894534
C64	10.386677782	1.157377722	1.998333838
C65	7.976749279	1.628317279	2.208015115
C66	6.871770617	2.600170372	2.549122034
C67	6.470134825	2.809789398	3.882149593
C68	6.206971411	3.319185312	1.540733985
C69	5.435291386	3.697886761	4.198843197
C70	5.181072010	4.216475612	1.842202372
C71	4.792822403	4.400554779	3.172961230
C72	4.641513527	7.751342012	-1.937207176
C73	3.414591437	8.651341528	-2.127502253
C74	4.198904785	6.329769945	-1.553681500
C75	1.959653814	9.799218039	-3.736861887
C76	0.595161505	9.132507402	-3.558043001
C77	-0.655535336	7.026421863	-3.283728347
C78	-0.991311421	6.861684742	-1.786672255
C79	-0.566269745	5.681851075	-4.029778568
C80	-1.783571641	4.751979320	-3.965314844
C81	-3.034090132	5.164737986	-4.764877110
C82	-4.394427165	7.169080951	-4.122396900
C83	-0.209672788	6.567626489	0.507921592
C84	-0.320759706	7.922651703	1.238480139
C85	0.827985764	5.707719791	1.236429488
C86	0.614694610	10.173182004	1.470546288
C87	-0.303176366	11.384998248	1.314977597
C88	-2.300234049	12.335509590	0.221031372
C89	-3.509985198	12.539278092	1.160883514
C90	-2.773900988	12.240067329	-1.249538314

	C91	-3.568073103	10.962138282	-1.492033434
	C92	-4.895283157	11.552791464	2.945165065
	C93	-4.749379208	12.558062392	4.082672166
	C94	-5.115002422	10.128461217	3.525226234
	C95	-5.421927218	9.100302312	2.458229735
	C96	-6.735352486	8.933818647	1.989172795
	C97	-4.408224474	8.316571584	1.881942509
	C98	-7.030546951	8.027690905	0.967766348
	C99	-4.680952717	7.421121581	0.842181109
	C100	-5.992970256	7.293718746	0.391266906
	C101	3.047228129	-3.414046000	-9.598404197
	C102	1.705942807	-3.334865967	-8.886713378
	C103	0.337626817	-2.003217973	-7.299027881
	C104	-0.381001766	-0.713940536	-7.710491867
	C105	-0.248988868	1.734325128	-7.714409277
	C106	0.765759455	2.810097909	-8.092174240
	C107	3.023855221	3.702783599	-7.994232461
	C108	3.393536213	3.888995368	-9.451140577
	C109	-3.714743809	-3.698260950	-8.704807256
	C110	-4.794989755	-2.725748038	-8.212664276
	C111	-2.989577562	-4.328698170	-7.492370188
	C112	-2.195121476	-5.580892828	-7.905864060
	C113	-1.269046240	-6.021337903	-6.794110521
	C114	-5.333520074	-0.460642706	-7.449238858
	C115	-5.115379211	0.873801892	-8.138292867
	C116	-5.374928379	-0.383540506	-5.900427451
	C117	-4.007424751	-0.392220981	-5.257557816
	C118	-3.237089743	0.779966815	-5.146677435
	C119	-3.446163010	-1.603847913	-4.820526554
	C120	-1.931827943	0.729155636	-4.640668240
	C121	-2.140340883	-1.656111354	-4.319040503
	C122	-1.373766223	-0.490210880	-4.238140048
	C123	7.680148508	-5.330217909	-5.972088172
	C124	8.998120630	-5.260151362	-5.185399178
	C125	6.523163236	-5.830034156	-5.091962607
	C126	5.159526028	-5.754625243	-5.707124414
	C127	4.721269718	-6.365240346	-6.865558764
	C128	3.013536909	-5.370810140	-5.863763595
	C129	10.620746710	-6.340766507	-3.736835092
	C130	11.795484114	-5.911647439	-4.588749351
	C131	1.303331735	-7.087267950	-0.287560364
	C132	2.705161413	-6.544528897	-0.297808463
	C133	3.408994886	-6.634707070	-1.710534154
	C134	3.355012845	-8.102083562	-2.216623534
	C135	4.153212857	-8.302627200	-3.514909689
	C136	4.348113978	-9.745614625	-3.885082301
	C137	4.910676858	-6.281836642	-1.471317743
	C138	3.269340998	-0.244567590	-2.760519102
	Fe139	3.326402090	-1.528928646	-4.282337652
	Fe140	1.805746045	1.257550017	-1.956629289
	Fe141	4.625352629	1.018066338	-2.041437099
	Fe142	3.337932600	1.003875116	-4.285045116
	Fe143	3.380785675	3.274190310	-2.717962253
	Fe144	4.606176680	-1.490603115	-2.021007480
	Fe145	2.036386730	-2.090538752	-2.072358816
	H146	-5.345882480	-7.996979402	1.144893099
	H147	-5.686695153	-7.086401018	-0.165099351
	H148	-8.962364325	-5.637702211	2.207802163
	H149	-7.985594650	-6.905379834	0.421445267

	H150	-7.720400237	-8.023961773	1.754104355
	H151	-7.324956258	-3.460817915	2.940663951
	H152	-8.030905523	-6.381138550	4.884094024
	H153	-8.922242722	-3.841750291	3.633841171
	H154	-7.201317473	-7.011292670	6.957223304
	H155	-5.414887822	-6.705385731	4.524403341
	H156	-6.988300710	-5.292978186	7.373135483
	H157	-1.926465403	-3.003639645	3.630397933
	H158	-4.287472282	-4.666023454	3.702086642
	H159	-3.182016691	-7.265157011	4.145452821
	H160	-3.736584063	-0.733356205	7.390749092
	H161	-4.732946643	-3.032654334	5.938875593
	H162	-2.831702041	-7.168644332	5.888835216
	H163	-3.603571502	-2.483066743	3.349214895
	H164	-3.457822909	-4.295509432	8.065577038
	H165	-4.951492738	-1.915287720	7.950219255
	H166	0.791578763	-3.885583953	3.872470858
	H167	0.676907078	-2.919733321	2.380503808
	H168	2.259737205	-3.195821112	3.132508874
	H169	-0.055501092	-1.590484743	4.372554465
	H170	-2.419094916	-5.321350514	9.842232490
	H171	-1.071228987	1.391357838	5.210815320
	H172	-0.521467198	-0.575150366	9.220060765
	H173	-1.004074944	2.799898872	9.337359586
	H174	0.920681417	-0.396471743	2.460074710
	H175	2.562482619	-0.604839315	3.088879526
	H176	9.549312341	2.565031107	3.357767239
	H177	-2.145491046	2.689965800	5.772318498
	H178	-2.066659199	3.526411776	8.109569493
	H179	-0.402536204	3.005935907	5.576720433
	H180	-0.312898948	3.829097683	8.061397013
	H181	-1.674444151	1.103045820	7.635731971
	H182	1.247972616	1.886117212	7.146965961
	H183	-0.297812686	-0.520059438	6.499782357
	H184	1.399966291	0.433729452	3.952215753
	H185	0.951811734	4.759233899	0.687940786
	H186	0.415219847	5.489297433	2.235616292
	H187	2.608528698	5.973367981	2.016315693
	H188	3.549585482	5.292771498	4.368654736
	H189	4.659760371	4.746281745	1.045323170
	H190	6.469132246	3.149623324	0.493559887
	H191	6.962401913	2.259945319	4.689101240
	H192	0.164362499	-2.921269409	6.566489199
	H193	1.099836534	-5.985819706	6.916907081
	H194	1.726928885	-5.302695719	8.432627199
	H195	-0.972800358	-4.860675462	7.285818786
	H196	-2.027417823	-3.732375747	10.546147094
	H197	5.122989411	3.833847938	5.238263486
	H198	2.775645649	-2.126599773	5.385566374
	H199	0.099140185	0.554692969	11.695279432
	H200	-0.003505987	-1.194100888	11.359604576
	H201	2.584198248	-0.814169680	10.513884453
	H202	12.371871952	-5.735329833	2.166766618
	H203	-1.541710518	-6.043153457	-3.854952465
	H204	2.020880396	-8.704550064	0.477724866
	H205	-2.897021086	-6.403042556	-8.111498346
	H206	-1.617282371	-5.381238997	-8.819970458
	H207	-3.716804804	-4.609032978	-6.714828332
	H208	-4.793086898	0.785952361	-9.202623069

	H209	-4.292510498	-4.496226718	-9.195949370
	H210	-3.380483159	-2.532811306	-1.819677607
	H211	-3.503551937	-5.341240270	-0.605351196
	H212	-3.410494988	-1.189901886	-8.060733628
	H213	-6.334668886	-0.790246912	-7.791827260
	H214	-2.176271941	-2.446019536	-9.314683162
	H215	-0.905895974	-5.804604110	-2.228491461
	H216	-2.326565228	-3.573237288	-7.043600610
	H217	-2.327238963	-3.818329790	-10.210833885
	H218	-6.655965450	-4.182261342	-1.895147108
	H219	-3.228101162	-2.724762722	-0.065803666
	H220	0.198498681	-5.089885912	-7.723036905
	H221	-1.947214374	-4.527944903	-0.684884749
	H222	-3.487344771	2.412366095	3.548808274
	H223	-2.232533043	0.698798210	1.828435428
	H224	-7.254135566	-0.027831270	4.955173707
	H225	-6.803459345	-0.750481961	2.091961804
	H226	-6.814595348	3.453690372	3.583651351
	H227	-8.326192148	8.031099151	5.751216404
	H228	-6.425700443	4.688556022	6.404301059
	H229	-8.756647582	4.694175718	4.872051629
	H230	-6.364313521	6.301350983	3.783310663
	H231	-11.269120588	8.609063317	2.759884169
	H232	-3.456189259	4.096078403	1.690147604
	H233	-5.042483292	3.548652535	1.144209923
	H234	-10.063119380	9.623034821	4.773002426
	H235	-8.476086387	4.901594062	3.141569592
	H236	-5.979889730	1.487739860	2.286546754
	H237	-6.047839854	-1.191824193	4.404336837
	H238	-9.976360171	0.549449173	1.354763926
	H239	-2.671009573	1.301906855	-2.349575438
	H240	-9.964541454	-1.210153039	1.572510971
	H241	10.376454726	3.893011573	1.555931459
	H242	8.751838816	3.981441047	1.398593094
	H243	0.980948677	6.467597984	-1.297750800
	H244	-1.186782239	6.081209758	0.634746964
	H245	0.970961699	8.799965104	-0.094996787
	H246	-4.103830398	3.117086131	-1.141735270
	H247	-5.909664364	3.687519742	-2.109452568
	H248	-5.573792895	4.987543929	-1.249485942
	H249	-6.012349912	0.877233299	-3.092691038
	H250	-7.758299167	0.537229178	-2.908682233
	H251	-1.890019131	12.292982101	-1.902243582
	H252	-3.420358822	13.103767122	-1.449725346
	H253	-1.911796198	9.981802147	-2.351157701
	H254	-3.280614185	8.985223626	-1.956480805
	H255	-4.295085704	8.867633251	-5.254669379
	H256	-3.534581156	4.265625496	-5.152636140
	H257	-8.060273732	7.898807455	0.624072592
	H258	-3.378702041	8.387841667	2.242830469
	H259	-5.470542404	7.256385700	-2.354012741
	H260	0.298753116	5.118256176	-3.646006800
	H261	1.464775822	7.258725731	-3.476418778
	H262	-2.064528325	4.551328062	-2.919219777
	H263	-8.209934403	-1.396826480	0.017956703
	H264	2.041915421	10.126032721	-4.782084876
	H265	-6.395414790	-1.629773177	-2.169925968
	H266	-0.344138881	5.912320894	-5.085110027
	H267	-3.872959052	6.863051841	0.365078089

H268	-7.570723528	2.483317462	-1.364837620
H269	1.948067277	10.696962430	-3.102555584
H270	-3.635681800	7.469304779	-6.007246250
H271	-5.607125129	8.655111835	-3.378537869
H272	-5.189112919	-4.095699047	-2.687834228
H273	-5.403640867	-3.924137092	0.008738902
H274	-7.547279712	9.513891843	2.433534022
H275	-5.705796669	0.760812462	-0.632571371
H276	-1.478148495	7.632474145	-3.688644825
H277	-2.742999505	5.754427816	-5.643109684
H278	-1.441517791	3.791550305	-4.377268220
H279	-4.464254469	5.363739711	-3.197847548
H280	0.600916102	9.908328128	2.539096202
H281	-1.533736471	10.338194511	0.051370539
H282	-1.699044984	13.250565260	0.327807386
H283	1.629367456	10.514232791	1.226430760
H284	-3.081972136	10.778029293	2.094865540
H285	3.418547412	-2.400816774	-9.809565137
H286	3.747348550	-3.863885927	-8.863939663
H287	7.471466737	-4.294149988	-6.275777344
H288	3.853017395	-4.439258476	-11.166478115
H289	2.418107676	-5.038532391	-10.648375424
H290	5.098274899	5.706843967	-1.470121014
H291	4.995066519	8.480735838	-0.067815174
H292	3.657081405	8.571493892	-4.166094769
H293	3.688926592	6.361273789	-0.579555142
H294	5.932850902	9.169483096	-1.214773895
H295	3.944285862	3.452978709	-7.443289125
H296	5.199453098	7.698796570	-2.884847229
H297	6.684992739	-6.027524193	3.943772822
H298	8.188510132	-6.896618797	1.418343106
H299	2.387884944	1.814163230	-7.233151320
H300	5.150010598	0.736031793	2.570629587
H301	-0.934357549	1.613251915	-8.562663476
H302	-1.708702059	-2.610743162	-4.008450678
H303	-0.845247359	2.151806826	-6.884353249
H304	5.316935842	-3.770791719	3.563490225
H305	1.099898961	0.318917714	-6.758830931
H306	4.954272378	-4.257837665	1.904997012
H307	5.994521940	-6.397093891	2.349537200
H308	7.390933105	-4.434682491	1.401879260
H309	7.785296004	-3.996341968	3.076907329
H310	-5.956323685	-1.245537157	-5.543255414
H311	7.628711829	-8.378669933	3.963194050
H312	6.652689887	-10.963250794	2.653576510
H313	-0.344439817	-0.528787256	-3.876996966
H314	8.513482738	-10.569197035	4.870006676
H315	6.976348886	-11.132807859	4.888727841
H316	5.968625355	-2.225915391	1.078539993
H317	-1.319761822	1.631922123	-4.571541548
H318	-4.031870413	-2.523597654	-4.877851849
H319	8.306983795	-8.940439433	-2.721174292
H320	6.719895132	-6.881095448	-4.814207856
H321	7.232897545	-7.407514156	-1.131717009
H322	7.472023738	-9.746279890	-3.740203330
H323	3.126381874	3.035848940	-10.120684748
H324	6.735058071	-4.320459800	-1.358975829
H325	3.335711685	-7.057065804	0.443906023
H326	2.301420422	-1.604392527	-7.957388297

	H327	3.766228410	-8.754501744	-1.430995589
	H328	2.734334566	-6.444493181	-7.663302885
	H329	1.999385090	-5.048016776	-5.662767493
	H330	8.372349320	-11.371531789	2.669949719
	H331	5.285761000	-0.124581278	1.057881545
	H332	8.978812317	-4.823883754	-0.666068350
	H333	7.336977974	-2.889072181	-1.329038785
	H334	10.027812779	-5.726531617	1.273348066
	H335	5.662469070	-2.032077620	4.653714944
	H336	3.401136805	-11.333591359	-4.288954762
	H337	3.631381285	-7.791866643	-4.337530642
	H338	5.154139464	-7.865199465	-3.424884305
	H339	2.304077935	-8.379377061	-2.386254225
	H340	0.648752992	-1.959977288	-6.243191567
	H341	-3.660498130	1.730604749	-5.482177704
	H342	-5.930821969	0.530134002	-5.640086229
	H343	2.654661485	-5.478411184	-0.039337887
	H344	9.179680319	-6.357247585	-0.806261041
	H345	8.238982077	-8.360013152	-0.434103234
	H346	4.836160155	-0.515309384	4.586692675
	H347	5.252172340	-6.948360490	-7.609030068
	H348	6.513903953	-5.261322856	-4.153379854
	H349	-0.355283622	-2.836140471	-7.437600393
	H350	2.648857019	4.658928200	-7.596920629
	H351	7.954489511	0.770217387	2.897323557
	H352	7.829742258	1.241785685	1.186950070
	H353	8.555266474	-5.721798143	-7.785985282
	H354	8.143065000	-7.076266023	-6.966178394
	H355	10.826976472	-7.367717287	-3.396094560
	H356	8.792978077	-7.200675077	-4.414174765
	H357	11.688700727	-6.124921569	-5.679445506
	H358	10.556371672	-5.700268266	-2.842780917
	H359	10.614201853	1.032179977	0.912344499
	H360	-3.704613732	12.892942738	4.286698392
	H361	11.667642864	-5.568525589	3.796348312
	H362	10.678626901	-3.228944024	2.708106502
	H363	-5.780975025	11.846768352	2.360021135
	H364	-4.218987878	9.832862202	4.092812699
	H365	-5.946047251	10.195484229	4.242731746
	H366	-7.206418893	6.393659802	-0.839027588
	H367	0.985046798	-3.068995653	-1.594623144
	H368	3.694991991	-1.641481909	-0.632126492
	H369	1.051702262	-0.503695615	0.168736433
	H370	-1.450988091	0.002933069	-0.536700673
	Mo371	3.748428352	-3.943289026	-3.052370605
	N372	-6.083906512	-7.784086772	0.468443717
	N373	-8.049160129	-5.420306186	2.599795577
	N374	-7.432806610	-5.705823016	5.354428010
	N375	-4.780306291	-6.632270148	5.319033177
	N376	-3.425314455	-4.385760612	4.171751941
	N377	-3.925659177	-2.476783728	6.231849364
	N378	-2.826235084	-3.700680959	8.607839034
	N379	-0.219956801	-4.740336245	7.969548345
	N380	1.159352152	-2.933219254	6.349505623
	N381	0.568262501	-0.044853376	6.763155164
	N382	0.237328991	-0.043557049	9.652617435
	N383	7.585503940	-10.432422416	4.463350307
	N384	7.828062509	-8.213720806	2.970701282
	N385	5.801181210	-2.363028592	2.080311435

	N386	5.203620194	-1.323216353	4.091256853
	N387	5.088830470	-0.155969132	2.082103712
	N388	10.262433027	-5.790201368	2.287869084
	N389	-5.848416325	-3.582072512	-2.074254961
	N390	-1.661632782	-5.678312601	-2.904634223
	N391	-6.295482853	-1.122704769	-1.274968048
	N392	-8.419579624	-0.471285256	0.385281516
	N393	-7.293575380	-0.373319538	2.913037836
	N394	-5.380726590	1.670493872	3.089673364
	N395	-3.555347595	2.356935734	-0.667992587
	N396	-2.230699366	0.679373493	-0.330540782
	N397	-6.197958162	4.205160433	3.882754132
	N398	-10.012767633	6.893531253	3.113323477
	N399	-9.880254810	8.692952223	4.405294871
	N400	9.546633586	3.339579555	1.328979770
	N401	5.549869607	8.271779673	-0.904495769
	N402	3.110103393	8.981433060	-3.413671696
	N403	0.574411529	7.782301901	-3.509338763
	N404	-4.038121520	5.893258478	-3.978391284
	N405	-3.947014865	7.919348982	-5.152714238
	N406	-5.168304570	7.758882001	-3.190355130
	N407	0.046083407	6.683835783	-0.926082518
	N408	0.322871558	8.986680935	0.677165442
	N409	-1.436232349	11.212167496	0.572362737
	N410	-2.819923895	9.891294280	-1.878367164
	N411	-3.728196127	11.563439783	2.077274119
	N412	2.919882344	-4.168093933	-10.848794020
	N413	1.511872874	-2.240079314	-8.129281695
	N414	0.261870596	0.420866200	-7.363498532
	N415	2.071544890	2.626092797	-7.777898757
	N416	-2.860174355	-3.089343853	-9.731607387
	N417	-4.401366891	-1.441788705	-7.979986766
	N418	7.820778307	-6.132602687	-7.202613457
	N419	4.073304038	-5.128926251	-5.096974162
	N420	3.368595977	-6.111688814	-6.942652544
	N421	9.380051009	-6.369154974	-4.491984323
	N422	-0.232751708	3.040494659	-0.790007761
	N423	0.551555280	2.352283595	-1.230351974
	O424	-5.803038525	-5.774487843	2.498243885
	O425	-6.537864282	-3.609569108	5.314447974
	O426	-4.535837515	-5.920620179	7.477525601
	O427	-1.620831127	-5.015052996	5.405229008
	O428	-1.946767213	-1.510116466	5.668040706
	O429	-2.259008336	-1.580285848	9.240060411
	O430	0.330295267	-4.517215725	10.187407701
	O431	3.080784212	-4.188539045	6.475512880
	O432	2.842375089	0.052708608	6.532566553
	O433	1.677772204	1.710601801	9.584685203
	O434	2.458867997	-0.218693351	12.462424393
	O435	8.234728763	-9.399443768	1.024658044
	O436	9.558965807	-6.436782926	4.379285924
	O437	12.714849654	-3.180375401	2.584794182
	O438	-5.413274318	-1.425206497	0.820616283
	O439	-3.722626512	-4.875233805	-3.432956017
	O440	-7.904138684	1.750501476	0.283494843
	O441	-7.098705400	2.400455053	-2.232455772
	O442	-9.304905099	0.245289088	3.839222529
	O443	-4.767158242	0.993060769	5.185676223
	O444	-4.029976043	4.847550430	4.211048836

	O445 -6.293456124 6.723770228 6.368094919<br>O446 10.888983235 0.431839962 2.844476627<br>O447 3.754376030 5.278577570 3.415522579<br>O448 2.737569222 9.066297364 -1.168343286<br>O449 -0.423597952 9.851908329 -3.501023078<br>O450 -2.177036082 6.915249285 -1.413453121<br>O451 -0.945228851 7.995421523 2.306688999<br>O452 2.063543086 6.420790619 1.335683214<br>O453 -0.015455826 12.449632559 1.877379681<br>O454 -4.220031654 13.548431326 1.058238964<br>O455 -4.782074470 10.883548833 -1.247884262<br>O456 -5.694082295 12.953644325 4.745896813<br>O457 -6.242309583 6.420274847 -0.683599712<br>O458 0.873544156 -4.272357333 -8.997636187<br>O459 -1.462833369 -0.733803360 -8.337956729<br>O460 0.354697951 3.846175536 -8.643685102<br>O461 3.969931707 4.878685356 -9.874548796<br>O462 -5.961895614 -3.106952603 -8.019221106<br>O463 -1.641430964 -6.670671688 -5.821293759<br>O464 0.015448683 -5.626947469 -6.888246665<br>O465 -5.307186631 1.964276207 -7.621739291<br>O466 9.733652211 -4.256701746 -5.230137906<br>O467 12.824953984 -5.438357698 -4.129039138<br>O468 0.302766340 -6.497485811 -0.668008082<br>O469 1.152277548 -8.365377488 0.188066943<br>O470 5.430269332 -10.308915654 -4.031332669<br>O471 3.173925493 -10.407775141 -4.057006083<br>O472 5.340543268 -5.240735800 -2.099588549<br>O473 5.615858026 -7.019782790 -0.741629376<br>O474 2.816807730 -5.763246022 -2.604260667<br>O475 9.640368023 -5.537251294 -0.483324804<br>O476 7.451479274 -3.778452494 -0.940003484<br>O477 -5.183026747 4.325638684 -1.869904577<br>O478 8.392736625 -9.467044442 -3.556218799<br>O479 8.156798426 -7.779458291 -1.233992942<br>S480 3.042242773 5.574590833 -2.794009545<br>S481 1.571111864 2.399225879 -3.916407919<br>S482 1.711096001 -3.073162560 -3.979693370<br>S483 3.402092524 2.337159861 -0.693458777<br>S484 0.630070502 -0.589742463 -1.128249637<br>S485 3.275596422 -0.259697995 -6.129929271<br>S486 3.449003812 -3.172746875 -0.769230160<br>S487 5.173717042 2.308787019 -3.779128023<br>S488 5.364089502 -2.448821640 -3.893437564<br>S489 6.133208682 -0.282615543 -1.007861678

13 35 (S=1)

**13 35 (S=1)**	bm22b2n2x6x3b135td.car_3
Fe(139) -2.392<br>Fe(140) -1.336<br>Fe(141) -2.583<br>Fe(142) 2.766<br>Fe(143) 2.910<br>Fe(144) 2.279<br>Fe(145) 0.039	C1 -6.936185983 -6.078825798 2.118393310<br>C2 -7.221092610 -7.237414204 1.158126835<br>C3 -7.891454776 -4.232552878 3.421865747<br>C4 -7.228476602 -4.480231893 4.775464836<br>C5 -6.799713979 -6.040649005 6.608400449<br>C6 -5.276691770 -6.180077482 6.504422737<br>C7 -3.367927347 -6.697574501 5.057701398<br>C8 -2.724326055 -5.317269026 4.902203193<br>C9 -2.926595272 -3.039410107 4.060900326<br>C10 -2.878563372 -2.295341178 5.403783312

	C11	-3.952606931	-1.812424870	7.525140905
	C12	-2.933848464	-2.356735576	8.533772809
	C13	-2.003722948	-4.341270940	9.625079347
	C14	-0.519320919	-4.530755726	9.287436705
	C15	1.126649386	-5.078945889	7.546729959
	C16	1.881553493	-4.007922699	6.755920915
	C17	1.717838399	-1.857001867	5.547757024
	C18	1.760511840	-0.525192834	6.316236722
	C19	1.010149310	-1.741496390	4.167571179
	C20	1.482213937	-0.519514110	3.370849225
	C21	1.196261065	-3.026017613	3.352048474
	C22	0.469016603	1.192452162	7.533137010
	C23	0.843116610	0.968670003	9.012326751
	C24	-0.922569379	1.843427108	7.358975270
	C25	-1.076679028	3.068989253	8.267755245
	C26	-1.163992825	2.226872030	5.892969933
	C27	0.482910154	-0.266426488	11.069384802
	C28	1.959951328	-0.435555344	11.361327544
	C29	7.658090741	-10.602103585	3.009041150
	C30	7.968053379	-9.315798507	2.242920205
	C31	8.081135667	-6.830271940	2.512320801
	C32	9.382116692	-6.309113891	3.154494478
	C33	6.872311421	-5.948222362	2.868478022
	C34	7.010679315	-4.487521459	2.428342570
	C35	5.667206294	-3.763307432	2.526138156
	C36	5.357706305	-1.286873064	2.743799467
	C37	11.562599933	-5.271202665	2.728269473
	C38	11.673517262	-3.762688265	2.645870993
	C39	-5.155755023	-3.231093352	-0.817949401
	C40	-5.619155232	-1.847445748	-0.334007312
	C41	-3.618543289	-3.195242661	-0.975783832
	C42	-2.930219264	-4.565959566	-1.152084735
	C43	-2.802344110	-5.049714909	-2.595800163
	C44	-6.577894332	0.279860438	-1.088413112
	C45	-7.680951165	0.564713316	-0.067494320
	C46	-6.899172575	0.988505608	-2.420228626
	C47	-9.314057435	-0.325456994	1.510817288
	C48	-8.628700191	-0.120833159	2.868779840
	C49	-6.535389210	-0.235364426	4.153392372
	C50	-5.470388710	0.860144448	4.186028290
	C51	-4.406552025	2.763053020	3.048974921
	C52	-4.845797950	4.031566864	3.820086081
	C53	-4.119304633	3.225268164	1.612504135
	C54	-3.459752105	2.242419543	0.698552544
	C55	-2.639965853	1.155647751	0.887731927
	C56	-2.849133249	1.466974790	-1.303956361
	C57	-6.767977049	5.471547917	4.373435935
	C58	-6.425815364	5.686818729	5.844473281
	C59	-8.300071979	5.383715783	4.177579433
	C60	-9.035271777	6.690403827	4.124457793
	C61	-8.962769189	7.805835648	4.936892590
	C62	-10.471499054	8.109726122	3.342903143
	C63	9.409169732	2.265078691	2.309100980
	C64	10.409402784	1.162770676	2.011314084
	C65	7.997714108	1.627764257	2.209732023
	C66	6.890795157	2.599584683	2.544193824
	C67	6.495729000	2.825794081	3.876215189
	C68	6.222568742	3.307357035	1.530405843
	C69	5.465005087	3.720981592	4.187218601

	C70	5.199854903	4.210248958	1.825756027
	C71	4.818676152	4.412076254	3.156015570
	C72	4.656339006	7.739411937	-1.946961986
	C73	3.424065459	8.632226264	-2.137558404
	C74	4.223835879	6.307960952	-1.589012362
	C75	1.967581568	9.780864692	-3.740775260
	C76	0.604285544	9.119244900	-3.537093391
	C77	-0.652882339	7.015716609	-3.278236100
	C78	-0.985854804	6.858125266	-1.780081670
	C79	-0.566261215	5.667021180	-4.016472652
	C80	-1.784486813	4.739135779	-3.942940345
	C81	-3.033217938	5.145329863	-4.748163889
	C82	-4.374756861	7.164538882	-4.106995484
	C83	-0.199512605	6.578528893	0.515371100
	C84	-0.317717278	7.938146262	1.235885931
	C85	0.843674425	5.728875647	1.247584665
	C86	0.610283720	10.191150023	1.457744045
	C87	-0.318865957	11.395210409	1.308045839
	C88	-2.326514517	12.328651038	0.218937627
	C89	-3.541972064	12.527613431	1.152262814
	C90	-2.793484015	12.232198067	-1.254580922
	C91	-3.580168432	10.951314589	-1.506482079
	C92	-4.922688691	11.551869564	2.943269871
	C93	-4.780818006	12.568531546	4.071977645
	C94	-5.149150205	10.132026599	3.529650510
	C95	-5.446051648	9.099483727	2.464547250
	C96	-6.756708494	8.924768291	1.992202303
	C97	-4.425551469	8.322513695	1.890040551
	C98	-7.043904428	8.017778885	0.969044532
	C99	-4.689665936	7.427436888	0.848013429
	C100	-6.000355782	7.291782904	0.394069826
	C101	3.066262210	-3.428001160	-9.573525659
	C102	1.716889485	-3.342601505	-8.878277854
	C103	0.344846342	-2.014008309	-7.288124249
	C104	-0.362035029	-0.715998835	-7.698385752
	C105	-0.202991240	1.732747565	-7.695950885
	C106	0.826554708	2.783426355	-8.105190957
	C107	3.085222246	3.660556615	-8.031989343
	C108	3.307752061	3.909280587	-9.509133846
	C109	-3.700128487	-3.673915232	-8.704401198
	C110	-4.773332612	-2.701183059	-8.199487528
	C111	-2.985167860	-4.325604709	-7.498088635
	C112	-2.183236173	-5.567108850	-7.927000195
	C113	-1.262580522	-6.019769241	-6.815625622
	C114	-5.314998948	-0.434296769	-7.456640574
	C115	-5.098742659	0.899501415	-8.146362519
	C116	-5.383374124	-0.357774633	-5.910027705
	C117	-4.022537234	-0.378772384	-5.259709899
	C118	-3.234796384	0.782867431	-5.173673059
	C119	-3.477657519	-1.593932302	-4.813073123
	C120	-1.921024235	0.716015982	-4.696799501
	C121	-2.161779447	-1.662873754	-4.342854816
	C122	-1.374168585	-0.510619383	-4.303694156
	C123	7.679245071	-5.357522569	-5.995700038
	C124	8.997390097	-5.276384144	-5.206857660
	C125	6.522498228	-5.839663561	-5.107053385
	C126	5.151157814	-5.755604673	-5.706377786
	C127	4.693546634	-6.344896328	-6.868966252
	C128	3.001206899	-5.375392048	-5.817134997

	C129	10.614320158	-6.339664154	-3.734040834
	C130	11.800597322	-5.928107738	-4.577926559
	C131	1.301214829	-7.072487294	-0.292237194
	C132	2.706676477	-6.540305440	-0.295080813
	C133	3.397890087	-6.630270864	-1.713615776
	C134	3.338714439	-8.096954193	-2.230606353
	C135	4.158777685	-8.305042214	-3.514298615
	C136	4.353766416	-9.747369109	-3.886799547
	C137	4.906472169	-6.295146608	-1.503376034
	C138	3.278595297	-0.225743844	-2.750618438
	Fe139	3.282645419	-1.535507143	-4.232351435
	Fe140	1.845223040	1.255124190	-1.975536727
	Fe141	4.670817051	1.015756514	-2.061894482
	Fe142	3.348979263	1.001082488	-4.282530647
	Fe143	3.431974340	3.269965686	-2.728934845
	Fe144	4.710015233	-1.496761275	-2.108474441
	Fe145	2.237898162	-2.055928176	-1.770687149
	H146	-5.333758260	-8.010421382	1.153287503
	H147	-5.667678405	-7.105621207	-0.162257140
	H148	-8.944945602	-5.633991881	2.197662201
	H149	-7.966430712	-6.911800018	0.415031406
	H150	-7.711995602	-8.027190847	1.753043159
	H151	-7.279579992	-3.469301233	2.919801608
	H152	-8.030567199	-6.367333839	4.872420864
	H153	-8.892122830	-3.813290746	3.595651452
	H154	-7.221120990	-6.993061902	6.956557868
	H155	-5.432760766	-6.719899214	4.527800431
	H156	-6.997259074	-5.276082236	7.370732005
	H157	-1.907900203	-3.032464716	3.652744481
	H158	-4.279950552	-4.677893347	3.698931358
	H159	-3.208421350	-7.279592242	4.139801796
	H160	-3.742930776	-0.743616692	7.393263719
	H161	-4.739001399	-3.034835916	5.937230361
	H162	-2.852036343	-7.201283207	5.883434790
	H163	-3.578417616	-2.497290828	3.361220774
	H164	-3.451364811	-4.301222093	8.054004479
	H165	-4.959585381	-1.924919238	7.950697990
	H166	0.827800582	-3.909577074	3.892617290
	H167	0.651343751	-2.954901843	2.397973528
	H168	2.257595397	-3.188730101	3.106248414
	H169	-0.064459980	-1.615243904	4.374804166
	H170	-2.424767199	-5.336067362	9.832780872
	H171	-1.115133405	1.355960633	5.223622859
	H172	-0.543178626	-0.578640422	9.228606362
	H173	-0.991132315	2.809981674	9.333054995
	H174	0.915051845	-0.453346460	2.429815935
	H175	2.544548811	-0.595441103	3.097711861
	H176	9.564807257	2.574284877	3.361503041
	H177	-2.160594324	2.678412366	5.781625090
	H178	-2.062572264	3.530486724	8.108792052
	H179	-0.414771385	2.964103946	5.560784079
	H180	-0.307165175	3.823193609	8.040828861
	H181	-1.683018771	1.098456376	7.655952046
	H182	1.238418509	1.880653282	7.154523179
	H183	-0.298818468	-0.536174069	6.515210126
	H184	1.333241209	0.424413293	3.916410207
	H185	0.955441238	4.768991788	0.716099421
	H186	0.441348960	5.530016037	2.255169865
	H187	2.637638769	5.996096784	1.994245914

	H188	3.583782506	5.321332430	4.347022243
	H189	4.675914858	4.731554100	1.024718663
	H190	6.480840139	3.127178344	0.484157299
	H191	6.990506062	2.284369471	4.687024725
	H192	0.158002250	-2.921447991	6.568920910
	H193	1.100676652	-5.980122171	6.915740734
	H194	1.726281556	-5.307377154	8.437060046
	H195	-0.972781390	-4.864662465	7.290886660
	H196	-2.038939607	-3.750813384	10.549120686
	H197	5.159689020	3.871376568	5.226842296
	H198	2.769535273	-2.130084797	5.387871510
	H199	0.090505681	0.547418406	11.701297709
	H200	-0.017852064	-1.201672764	11.366790147
	H201	2.571517786	-0.815655738	10.510730044
	H202	12.384686996	-5.728940497	2.157994577
	H203	-1.532866885	-6.031877667	-3.866204641
	H204	2.015023784	-8.732621711	0.382338498
	H205	-2.880633449	-6.388477282	-8.148596324
	H206	-1.601172899	-5.351315627	-8.834704695
	H207	-3.720836195	-4.624696453	-6.736046159
	H208	-4.760070526	0.811021895	-9.205458779
	H209	-4.283648206	-4.461457471	-9.205901549
	H210	-3.365891481	-2.534622906	-1.821108880
	H211	-3.492854479	-5.341362541	-0.603204803
	H212	-3.391651552	-1.159337365	-8.077188294
	H213	-6.312503380	-0.763388609	-7.812188275
	H214	-2.149211749	-2.428569043	-9.297888914
	H215	-0.890782090	-5.787995246	-2.242057097
	H216	-2.331067643	-3.575667094	-7.026748439
	H217	-2.316043550	-3.786830999	-10.214022680
	H218	-6.649246295	-4.184403202	-1.877164420
	H219	-3.212493697	-2.728091432	-0.066367638
	H220	0.213725858	-5.101768299	-7.742005573
	H221	-1.934522241	-4.532233177	-0.688051633
	H222	-3.483915391	2.420517312	3.538141484
	H223	-2.276520581	0.684680835	1.794119842
	H224	-7.249823211	-0.013959770	4.958907726
	H225	-6.794569275	-0.747248052	2.098861224
	H226	-6.809247840	3.469076973	3.615273964
	H227	-8.347924101	8.048172139	5.796284427
	H228	-6.395783779	4.742860760	6.442061150
	H229	-8.728480067	4.718744976	4.947935748
	H230	-6.359825663	6.310948155	3.789403148
	H231	-11.242697888	8.601224267	2.754893101
	H232	-3.477552359	4.118523209	1.687435969
	H233	-5.063776254	3.559562429	1.151476269
	H234	-10.072876376	9.628111593	4.782861886
	H235	-8.476987964	4.889874799	3.209584475
	H236	-6.004256651	1.518411291	2.301637000
	H237	-6.045563793	-1.183739559	4.417630877
	H238	-9.972176320	0.538708195	1.353283334
	H239	-2.728100750	1.343454193	-2.376219236
	H240	-9.946574445	-1.220770621	1.572511340
	H241	10.392227748	3.896370964	1.550922070
	H242	8.766716481	3.981248263	1.398908829
	H243	0.989556180	6.487049012	-1.290179106
	H244	-1.174635735	6.088852514	0.646480719
	H245	0.973553824	8.811849416	-0.100725978
	H246	-4.125260051	3.171443250	-1.141139638

	H247	-5.917713462	3.680671918	-2.101460268
	H248	-5.577909393	4.986508278	-1.248031251
	H249	-6.051764659	0.867670389	-3.109027464
	H250	-7.792864060	0.534386098	-2.880190349
	H251	-1.906094908	12.289256747	-1.901962560
	H252	-3.442283069	13.093083311	-1.455801197
	H253	-1.902243344	9.962482614	-2.309221602
	H254	-3.271849011	8.965401897	-1.926874169
	H255	-4.241190474	8.869364019	-5.224886746
	H256	-3.535024520	4.243072020	-5.126251726
	H257	-8.072104656	7.882413438	0.623766327
	H258	-3.398148317	8.400846590	2.254941381
	H259	-5.473064140	7.251706858	-2.350774049
	H260	0.300360061	5.105608398	-3.632547748
	H261	1.465936468	7.240866062	-3.502051878
	H262	-2.066990981	4.551865342	-2.894731038
	H263	-8.211989088	-1.396347650	0.004308670
	H264	2.036751569	10.093499209	-4.791390312
	H265	-6.385471931	-1.633618384	-2.171749824
	H266	-0.347400615	5.891186356	-5.073937235
	H267	-3.877329845	6.874516971	0.372202911
	H268	-7.569087473	2.482086344	-1.349572319
	H269	1.967044579	10.686095252	-3.117818887
	H270	-3.570895651	7.474041438	-5.970508314
	H271	-5.556678402	8.672516909	-3.352813116
	H272	-5.185757585	-4.112863651	-2.676730669
	H273	-5.383864995	-3.922061777	0.015977425
	H274	-7.573497922	9.498417451	2.436198867
	H275	-5.688156447	0.761349013	-0.651376175
	H276	-1.476743748	7.619560140	-3.683103720
	H277	-2.742568909	5.724641107	-5.633137527
	H278	-1.444632242	3.772233479	-4.342491907
	H279	-4.475465644	5.354131620	-3.196968070
	H280	0.605992719	9.927008462	2.526601690
	H281	-1.551337967	10.335711702	0.057080789
	H282	-1.731150446	13.247131221	0.328225915
	H283	1.620045580	10.540372617	1.205261520
	H284	-3.093166773	10.786209915	2.114874968
	H285	3.451210379	-2.417146433	-9.772893779
	H286	3.750938218	-3.889995722	-8.832554363
	H287	7.474372437	-4.325554630	-6.315358391
	H288	3.877827950	-4.462609180	-11.133507433
	H289	2.423372958	-5.034668278	-10.639264835
	H290	5.128488454	5.691738942	-1.515166655
	H291	4.983215674	8.432437606	-0.058227868
	H292	3.666627001	8.556613521	-4.177486410
	H293	3.714174907	6.321583266	-0.614308633
	H294	5.924269282	9.156757774	-1.180019324
	H295	4.047702505	3.354971726	-7.592132505
	H296	5.225516177	7.707251272	-2.889138010
	H297	6.706538335	-5.999846714	3.957655611
	H298	8.194278569	-6.872624776	1.422072760
	H299	2.435726065	1.797517656	-7.209874014
	H300	5.137030088	0.730809702	2.563423110
	H301	-0.910901719	1.621500047	-8.526940226
	H302	-1.740365410	-2.619344841	-4.024875152
	H303	-0.768320191	2.169993406	-6.855075351
	H304	5.280615081	-3.778325624	3.553423279
	H305	1.130557191	0.299340739	-6.744878526

	H306	4.922108210	-4.278690181	1.897815747
	H307	5.994723366	-6.403467573	2.381346140
	H308	7.350824067	-4.432736109	1.383095876
	H309	7.756932538	-3.965043195	3.048992500
	H310	-5.975292216	-1.216538851	-5.561833126
	H311	7.632150932	-8.357764120	3.964714145
	H312	6.682664447	-10.948011370	2.633409267
	H313	-0.336968637	-0.559584676	-3.976000613
	H314	8.493204561	-10.568283073	4.889516970
	H315	6.946567492	-11.106366096	4.872306633
	H316	5.849147395	-2.242874980	1.048343561
	H317	-1.293330914	1.609098587	-4.643933410
	H318	-4.081998285	-2.503476786	-4.847127692
	H319	8.308666181	-8.925817666	-2.716710691
	H320	6.711777296	-6.890667332	-4.822298977
	H321	7.231539422	-7.397396694	-1.129632029
	H322	7.478019786	-9.736063599	-3.736236341
	H323	3.021735439	3.060857524	-10.176179312
	H324	6.745975471	-4.357519051	-1.418943801
	H325	3.329814882	-7.065212538	0.444641920
	H326	2.315690497	-1.625261274	-7.929739064
	H327	3.729900300	-8.756210413	-1.440206078
	H328	2.691691285	-6.419098851	-7.630700758
	H329	1.990041995	-5.059140138	-5.593583301
	H330	8.403279636	-11.347830596	2.682765199
	H331	5.229435284	-0.131491110	1.051764120
	H332	8.981469452	-4.817868998	-0.669023549
	H333	7.299282951	-2.907832657	-1.324701348
	H334	10.038454285	-5.711925537	1.270380366
	H335	5.671622878	-2.052135019	4.632173766
	H336	3.410976058	-11.333063417	-4.305362884
	H337	3.657323686	-7.790511797	-4.346974324
	H338	5.160469540	-7.874455063	-3.408346815
	H339	2.288354801	-8.365310955	-2.417697394
	H340	0.650263363	-1.978121774	-6.230164468
	H341	-3.649886614	1.735759151	-5.511784601
	H342	-5.934931920	0.560909658	-5.657825016
	H343	2.670505078	-5.471270076	-0.046054515
	H344	9.184686966	-6.349394451	-0.804524032
	H345	8.243529524	-8.345948936	-0.429583099
	H346	4.862746283	-0.523592844	4.596870493
	H347	5.212011996	-6.917459094	-7.629349936
	H348	6.527628037	-5.266270837	-4.172538778
	H349	-0.352944332	-2.841291198	-7.435562291
	H350	2.781439941	4.603633123	-7.550358210
	H351	7.975319713	0.771725372	2.901197845
	H352	7.854785006	1.238229771	1.189100529
	H353	8.562524754	-5.786724882	-7.796785690
	H354	8.129577970	-7.122877189	-6.957576275
	H355	10.812034403	-7.361248806	-3.372934505
	H356	8.792667895	-7.208120273	-4.413436054
	H357	11.708831003	-6.155239591	-5.667107927
	H358	10.546054020	-5.684313724	-2.850720218
	H359	10.664036327	1.050102650	0.929838346
	H360	-3.741381646	12.929295210	4.257779799
	H361	11.684671285	-5.549286394	3.789818898
	H362	10.704347895	-3.211900860	2.720724953
	H363	-5.805444161	11.844550187	2.352851136
	H364	-4.260230008	9.838053756	4.109857175

	H365	-5.987977434	10.204903768	4.237132260
	H366	-7.205857921	6.392863102	-0.844039504
	H367	1.360117787	-3.057893242	-1.060359725
	H368	2.381723670	-1.480176947	-0.393347257
	H369	0.565102445	-0.383914947	-0.123989542
	H370	-1.589305288	-0.034627126	-0.602744099
	Mo371	3.761750209	-3.930773119	-3.015981187
	N372	-6.068952601	-7.799130505	0.473385897
	N373	-8.031077629	-5.417926957	2.588791315
	N374	-7.443021616	-5.688609163	5.351102846
	N375	-4.797964069	-6.642277248	5.321405995
	N376	-3.421413488	-4.404058712	4.178697704
	N377	-3.926439520	-2.490940838	6.238540187
	N378	-2.827275453	-3.708174497	8.606987190
	N379	-0.220224995	-4.742623389	7.974521979
	N380	1.154870480	-2.929188634	6.359854377
	N381	0.564785391	-0.049539649	6.764710158
	N382	0.219862477	-0.050950065	9.658280535
	N383	7.576992696	-10.415920143	4.462223523
	N384	7.845139697	-8.192530067	2.974998220
	N385	5.739356368	-2.373519770	2.059163960
	N386	5.221201661	-1.325676456	4.086245770
	N387	5.049417421	-0.160495982	2.077327376
	N388	10.275124128	-5.773078921	2.284846300
	N389	-5.839731607	-3.589614853	-2.065501896
	N390	-1.647933118	-5.663356090	-2.916703135
	N391	-6.284764882	-1.126399981	-1.277120071
	N392	-8.405937169	-0.473713677	0.387618907
	N393	-7.287817636	-0.373457502	2.919303484
	N394	-5.381608245	1.680214064	3.091942065
	N395	-3.571388599	2.404445442	-0.676232726
	N396	-2.292107281	0.693092901	-0.366789572
	N397	-6.186446693	4.214451288	3.916222118
	N398	-9.983708270	6.893574617	3.133947816
	N399	-9.879430194	8.697859228	4.421008157
	N400	9.562623696	3.340939561	1.329096733
	N401	5.547642867	8.248306930	-0.894305960
	N402	3.118405364	8.961653816	-3.423364627
	N403	0.577437754	7.767927857	-3.511697199
	N404	-4.037733819	5.882233959	-3.971071720
	N405	-3.896987099	7.920208213	-5.119447908
	N406	-5.163145187	7.751248395	-3.186271437
	N407	0.052213325	6.685672658	-0.919698944
	N408	0.322712755	9.000797202	0.668371778
	N409	-1.457081576	11.211626273	0.575672679
	N410	-2.817718376	9.874931808	-1.849895901
	N411	-3.755197281	11.557639698	2.075997335
	N412	2.945558026	-4.174317327	-10.829468331
	N413	1.523278434	-2.254525800	-8.111833242
	N414	0.289880297	0.411993174	-7.347910108
	N415	2.128260224	2.594501998	-7.781284808
	N416	-2.839689478	-3.060030822	-9.722099905
	N417	-4.380349883	-1.414828268	-7.981315875
	N418	7.818955294	-6.180151844	-7.212297336
	N419	4.073470742	-5.146649926	-5.065249911
	N420	3.338603735	-6.093570608	-6.917796492
	N421	9.378921554	-6.376298250	-4.498255756
	N422	-0.163266893	2.998872092	-0.704585186
	N423	0.613752621	2.329563069	-1.185459583

	O424	-5.786870685	-5.782540253	2.494837030
	O425	-6.536440543	-3.597659404	5.309129871
	O426	-4.546407174	-5.906640994	7.470362124
	O427	-1.628615945	-5.048842876	5.419863161
	O428	-1.930440765	-1.549123344	5.698724076
	O429	-2.283327438	-1.588175464	9.262350992
	O430	0.323421564	-4.528945177	10.194900164
	O431	3.081166243	-4.176642724	6.490947968
	O432	2.834324563	0.072545780	6.493898547
	O433	1.675652073	1.689551606	9.588328350
	O434	2.447724765	-0.236684594	12.463594368
	O435	8.274332986	-9.377024955	1.033382345
	O436	9.566825199	-6.397006631	4.381147273
	O437	12.734820874	-3.172733068	2.520291330
	O438	-5.401345880	-1.421793484	0.818442443
	O439	-3.726735214	-4.895982182	-3.426253910
	O440	-7.849443774	1.739931550	0.329713777
	O441	-7.108976515	2.396352456	-2.222150065
	O442	-9.301739685	0.250617975	3.836742474
	O443	-4.746715861	0.987990769	5.177268396
	O444	-4.014000103	4.855002724	4.210641864
	O445	-6.235306437	6.775507199	6.356272994
	O446	10.888112689	0.424313503	2.860149844
	O447	3.783966193	5.296482650	3.393088242
	O448	2.742257072	9.042070602	-1.179653316
	O449	-0.409612083	9.840548044	-3.443397014
	O450	-2.171346383	6.908715633	-1.405598252
	O451	-0.944065833	8.015829746	2.302558997
	O452	2.083116409	6.437902624	1.317655383
	O453	-0.034051564	12.462958562	1.865869844
	O454	-4.261320502	13.528981184	1.037616001
	O455	-4.802140093	10.874094576	-1.304240092
	O456	-5.724934980	12.951485231	4.743077909
	O457	-6.242396986	6.418355236	-0.683215251
	O458	0.878371055	-4.272371186	-9.007741187
	O459	-1.442390631	-0.727022710	-8.328467491
	O460	0.434843213	3.810729603	-8.687407673
	O461	3.796635938	4.936361536	-9.952516667
	O462	-5.935839785	-3.084047628	-7.983999950
	O463	-1.643399310	-6.663818724	-5.842524230
	O464	0.027225904	-5.641641314	-6.909417885
	O465	-5.312444842	1.990762508	-7.639269523
	O466	9.730588379	-4.271832741	-5.261930489
	O467	12.825839718	-5.452940395	-4.110564913
	O468	0.300484677	-6.462759171	-0.639593179
	O469	1.144973508	-8.370519226	0.127202194
	O470	5.436717576	-10.309429742	-4.033282441
	O471	3.180441358	-10.410087097	-4.065421540
	O472	5.356171892	-5.301260158	-2.196412993
	O473	5.609110649	-7.007638576	-0.748609130
	O474	2.797025213	-5.747243541	-2.586776178
	O475	9.645800697	-5.528169712	-0.485430682
	O476	7.436233185	-3.808246448	-0.969432222
	O477	-5.188612323	4.317614875	-1.863608359
	O478	8.396743884	-9.450567677	-3.552853096
	O479	8.155849665	-7.768749311	-1.231553381
	S480	3.077835039	5.556867528	-2.840431100
	S481	1.612675066	2.430936287	-3.904792321
	S482	1.674824178	-3.031809359	-3.742720710

S483	3.453031897	2.330969517	-0.705989362
S484	0.500549549	-0.591292270	-1.467214840
S485	3.227765307	-0.290545841	-6.099658770
S486	3.851393120	-3.118312681	-0.804126023
S487	5.201955186	2.290019537	-3.817438228
S488	5.330438920	-2.449671209	-4.027987684
S489	6.195199385	-0.255771017	-1.038351911

13 35 (S=1)

TS 13 → 1 47 (S=1)

Fe(139) 2.333
Fe(140) -1.060
Fe(141) 2.804
Fe(142) -2.617
Fe(143) 3.000
Fe(144) -2.263
Fe(145) -0.812

bm22b2n2x6x6hn2hn47tj_1_53131.955

C1	-7.856478855	-5.134861677	-0.988096461
C2	-7.954621775	-6.147909089	-2.133902058
C3	-9.001111534	-3.339499696	0.238818608
C4	-8.817054871	-3.839212182	1.670664420
C5	-9.150004167	-5.646604781	3.281203612
C6	-7.697818310	-5.998574042	3.622458248
C7	-5.490177585	-6.611296937	2.751919636
C8	-4.690417521	-5.330261681	3.008354577
C9	-4.374545673	-2.970509893	2.485358154
C10	-4.675030270	-2.390978382	3.875851219
C11	-6.309901617	-2.013000226	5.630337014
C12	-5.716734696	-2.802622185	6.805493205
C13	-5.405826316	-4.996893887	7.841037358
C14	-3.920869747	-5.374339771	7.928149821
C15	-1.885111093	-5.975948261	6.688149809
C16	-0.798702217	-4.959284316	6.328188973
C17	-0.326264107	-2.690303266	5.448480822
C18	-0.391347889	-1.481546732	6.400718289
C19	-0.543949268	-2.309718222	3.958976756
C20	0.300663572	-1.099115626	3.549670734
C21	-0.258033950	-3.510991808	3.050635642
C22	-1.795631402	0.280498373	7.407050088
C23	-1.930865591	-0.154889669	8.879652989
C24	-2.985907818	1.149422747	6.931225111
C25	-3.294877095	2.266406975	7.933924778
C26	-2.705556189	1.734371085	5.540540655
C27	-3.062577099	-1.537463378	10.522293292
C28	-1.790669400	-1.970425000	11.223931298
C29	5.083641504	-11.909584996	3.520100306
C30	5.746260420	-10.606584974	3.071895330
C31	6.066098010	-8.219294074	3.749555904
C32	7.141312312	-7.982198234	4.826447238
C33	4.915306933	-7.201972376	3.855446397
C34	5.358699896	-5.742164727	3.725724717
C35	4.150951728	-4.828922061	3.508425420
C36	4.080232070	-2.365605379	3.948218998
C37	9.436357278	-7.236158769	5.250618011
C38	9.778417092	-5.770590339	5.403790404
C39	-4.932626911	-2.298154920	-2.790133428
C40	-5.375939411	-0.920630601	-2.267905633
C41	-3.431433615	-2.475754896	-2.475528338
C42	-2.874970850	-3.905825394	-2.637443940
C43	-2.351984591	-4.249828170	-4.029734074
C44	-5.832583228	1.389545121	-2.956870799
C45	-7.177814968	1.726307080	-2.307882481
C46	-5.609953212	2.277758704	-4.198902495
C47	-9.294778448	0.904676368	-1.412086083
C48	-9.059677106	0.844647786	0.104315558

	C49	-7.505018195	0.303921079	1.929822342
	C50	-6.387903608	1.209939104	2.442242149
	C51	-4.807472767	3.031736214	1.979762291
	C52	-5.329582491	4.257331423	2.765177801
	C53	-4.034087834	3.610831041	0.785707228
	C54	-3.266085099	2.644654738	-0.054803378
	C55	-2.716540767	1.404711709	0.166896282
	C56	-2.190877965	2.013228238	-1.905794276
	C57	-7.145637001	5.913385061	2.894631072
	C58	-7.268815469	5.909793473	4.416471335
	C59	-8.538027505	6.082523637	2.237506395
	C60	-9.074454683	7.482480237	2.169424310
	C61	-9.161552709	8.466723183	3.135892346
	C62	-10.024960507	9.183094446	1.225086278
	C63	8.376855098	0.549261194	5.342933865
	C64	9.299760750	-0.643817475	5.176300037
	C65	7.010561868	0.145298737	4.726899014
	C66	5.964670849	1.227969525	4.856063414
	C67	5.196199687	1.362599144	6.028325831
	C68	5.728633235	2.133223282	3.806013514
	C69	4.219716184	2.357242251	6.148201072
	C70	4.763359451	3.134800362	3.914638469
	C71	4.004800035	3.243274827	5.083250475
	C72	5.831971923	7.092310991	0.724257983
	C73	4.837146364	8.186178286	0.318551999
	C74	5.133799217	5.722214048	0.771884094
	C75	4.108478890	9.726187514	-1.447871815
	C76	2.694869607	9.262196085	-1.797379560
	C77	1.200041038	7.353619300	-2.234693459
	C78	0.388714233	7.081558289	-0.950968808
	C79	1.372300791	6.096679045	-3.109223766
	C80	0.103984514	5.356806333	-3.548879541
	C81	-0.778471573	6.037180840	-4.613705565
	C82	-2.033164271	8.152620715	-4.112830953
	C83	0.361914433	6.426454539	1.401011929
	C84	0.171017775	7.693028382	2.265196686
	C85	1.015333585	5.340037222	2.261422247
	C86	1.211977814	9.741172006	3.110391786
	C87	0.521040927	11.083767803	2.871719501
	C88	-0.895546902	12.441200555	1.371766638
	C89	-2.312676705	12.712401069	1.923569618
	C90	-0.881511687	12.581980733	-0.170705574
	C91	-1.682352371	11.470714392	-0.838238750
	C92	-4.286755973	11.758965519	3.041393136
	C93	-4.396524417	12.615592869	4.300646645
	C94	-4.840610503	10.334688838	3.313976860
	C95	-4.900688816	9.485774579	2.063966781
	C96	-6.008391688	9.560888269	1.204778783
	C97	-3.841643777	8.633578513	1.708068936
	C98	-6.055117477	8.823875249	0.018923814
	C99	-3.860106496	7.906706522	0.513202767
	C100	-4.969875686	8.016939884	-0.322564929
	C101	5.536309550	-2.779831533	-8.638890955
	C102	4.058070720	-2.573168683	-8.359231942
	C103	2.416504789	-1.223128170	-7.071863083
	C104	2.016717719	0.197709160	-7.487337453
	C105	2.418640100	2.585646185	-7.092818681
	C106	3.623397013	3.518248470	-6.998243082
	C107	5.809490541	4.049381753	-6.084197854

	C108	6.594489103	4.339710404	-7.345304230
	C109	-1.133765090	-2.129547470	-9.848668542
	C110	-2.198107356	-1.064084669	-9.556965983
	C111	-0.905978199	-2.996992558	-8.588403191
	C112	-0.168188041	-4.304201193	-8.932271205
	C113	0.305357252	-4.993196298	-7.671911491
	C114	-2.697896384	1.161515569	-8.667584755
	C115	-2.128108463	2.514697272	-9.048416229
	C116	-3.258714548	1.076747834	-7.224175383
	C117	-2.180249906	0.808081731	-6.206334634
	C118	-1.363101021	1.841899591	-5.714834591
	C119	-1.893116035	-0.514096364	-5.826108632
	C120	-0.257418020	1.545754386	-4.911079825
	C121	-0.784784016	-0.809694919	-5.028135658
	C122	0.051808780	0.221181367	-4.591915276
	C123	8.540095915	-5.770276187	-4.155158102
	C124	9.546084980	-5.977680377	-3.009899944
	C125	7.116307230	-6.175432738	-3.735748827
	C126	6.021550272	-5.807436706	-4.690131028
	C127	5.891332500	-6.185193923	-6.012456234
	C128	4.077480225	-5.084317877	-5.375310295
	C129	10.506972166	-7.433997134	-1.315580979
	C130	11.930801941	-7.067096301	-1.675410073
	C131	0.559365968	-7.082951063	-0.885938224
	C132	1.944655896	-6.756569278	-0.402548410
	C133	3.022056462	-6.797868268	-1.561586854
	C134	2.953710083	-8.176471346	-2.284622099
	C135	4.099880860	-8.383036539	-3.289387325
	C136	4.222036572	-9.788400228	-3.811110960
	C137	4.417737346	-6.718432984	-0.871026435
	C138	3.960462294	-0.420487661	-1.653062270
	Fe139	4.318977707	-1.506060619	-3.252020938
	Fe140	2.429730788	0.782911548	-0.913427246
	Fe141	5.207482806	0.510386835	-0.426413807
	Fe142	4.558974627	0.984920842	-2.892144917
	Fe143	4.230669837	2.973751976	-1.149603035
	Fe144	4.986531815	-1.961809507	-0.818876350
	Fe145	2.454985226	-1.964931532	-1.367504251
	H146	-6.242055004	-7.176825101	-1.720200056
	H147	-6.067051608	-6.086329883	-2.921427312
	H148	-9.744376909	-4.443252792	-1.436844387
	H149	-8.419427083	-5.663778221	-3.007084203
	H150	-8.665773296	-6.925501538	-1.804929943
	H151	-8.201441301	-2.605943347	0.066890370
	H152	-9.792747625	-5.591874079	1.227581573
	H153	-9.967446695	-2.820832575	0.173296395
	H154	-9.757387855	-6.558999845	3.339182592
	H155	-7.270980914	-6.256418908	1.632330136
	H156	-9.498810270	-4.949850453	4.054393838
	H157	-3.283228020	-3.062274989	2.409581842
	H158	-5.694928804	-4.351591423	1.489073089
	H159	-5.110029843	-7.104515596	1.848205676
	H160	-5.965875736	-0.975876601	5.731762087
	H161	-6.667779813	-2.932574506	3.718338737
	H162	-5.321362313	-7.274176829	3.608315508
	H163	-4.715418647	-2.268680626	1.711368855
	H164	-6.297434346	-4.591951782	5.941890093
	H165	-7.404946902	-2.029651176	5.712289991
	H166	-0.898170875	-4.369768835	3.298455463

	H167	-0.430737821	-3.243098680	1.996349282
	H168	0.793695080	-3.826859454	3.140101304
	H169	-1.605262618	-2.035430793	3.849941699
	H170	-5.978331541	-5.934418894	7.786065061
	H171	-2.525270089	0.950204390	4.791951740
	H172	-3.467296587	-1.496161963	8.433401338
	H173	-3.583887494	1.871276779	8.918882016
	H174	0.068024457	-0.811864320	2.512289831
	H175	1.376354991	-1.325304940	3.580651600
	H176	8.224416145	0.708027625	6.427616229
	H177	-3.564560381	2.332074480	5.202435129
	H178	-4.128635039	2.880366215	7.562017925
	H179	-1.821867086	2.392496432	5.572782706
	H180	-2.423935759	2.925270502	8.074487930
	H181	-3.872909615	0.493466232	6.863165790
	H182	-0.877040642	0.883770119	7.380232030
	H183	-2.389290674	-1.179343866	5.945198749
	H184	0.116494517	-0.221830498	4.187720534
	H185	1.198736405	4.454098911	1.630176682
	H186	0.291267977	5.070598283	3.048418600
	H187	2.476464679	5.245040636	3.567904143
	H188	2.561763184	4.190139245	5.970657432
	H189	4.571845843	3.807488648	3.078909754
	H190	6.280080828	2.027937607	2.868569278
	H191	5.354978003	0.672925702	6.863003708
	H192	-2.235902245	-3.612770844	5.777364208
	H193	-1.815697924	-6.790523401	5.951481805
	H194	-1.626644241	-6.391082013	7.671444844
	H195	-3.759488932	-5.410077271	5.860225670
	H196	-5.655573065	-4.498801911	8.786618701
	H197	3.620420563	2.434807450	7.058716847
	H198	0.686570731	-3.096543662	5.572082145
	H199	-3.545999995	-0.745960808	11.118370895
	H200	-3.736474943	-2.409041199	10.507363341
	H201	-0.992423383	-2.366645285	10.555201487
	H202	10.330429850	-7.788692487	4.923331769
	H203	-0.880814403	-5.303184678	-4.987491196
	H204	0.831880067	-8.873940380	-0.225325249
	H205	-0.858142005	-4.981884582	-9.455166261
	H206	0.685060048	-4.098289765	-9.595424496
	H207	-1.869524343	-3.245560625	-8.117407009
	H208	-1.466021932	2.494323328	-9.945784196
	H209	-1.612912358	-2.768950785	-10.606886583
	H210	-2.854356689	-1.775099402	-3.099622404
	H211	-3.658344763	-4.643935396	-2.393201291
	H212	-0.767146589	0.224193800	-8.777742258
	H213	-3.554666862	1.028048978	-9.358272660
	H214	0.647706750	-1.065287269	-9.776506366
	H215	-0.768521436	-5.330755452	-3.225412352
	H216	-0.340738102	-2.405677532	-7.851958506
	H217	0.635613517	-2.291641171	-10.875809158
	H218	-6.104474586	-2.908202156	-4.378306987
	H219	-3.283341721	-2.173603821	-1.428012121
	H220	2.080802349	-4.194249733	-7.973388545
	H221	-2.073344982	-4.063404089	-1.902150106
	H222	-4.132969941	2.496882214	2.663325665
	H223	-2.720840568	0.767480879	1.044335390
	H224	-8.409129895	0.553087899	2.501893486
	H225	-7.142591640	0.068700983	-0.160357621

	H226	-7.157133664	4.038081693	1.860745582
	H227	-8.835409389	8.506722731	4.169071330
	H228	-7.497275073	4.904380911	4.848716742
	H229	-9.256730778	5.407332110	2.735524861
	H230	-6.484423366	6.741419789	2.596222271
	H231	-10.504161026	9.853516936	0.516195949
	H232	-3.338218328	4.361791476	1.192384005
	H233	-4.741294047	4.158016690	0.141003479
	H234	-9.961047166	10.440865090	2.942289424
	H235	-8.451920881	5.731941725	1.197417820
	H236	-6.200421179	2.125471236	0.599768188
	H237	-7.238843592	-0.732318714	2.185582893
	H238	-9.767693180	1.873629802	-1.619816745
	H239	-1.752594572	2.001156894	-2.900414546
	H240	-10.009872263	0.115658176	-1.680923680
	H241	9.715617585	2.094321382	5.179733440
	H242	8.239391320	2.439209268	4.566940182
	H243	2.046999023	6.339636281	0.045014668
	H244	-0.653567851	6.089924839	1.150022817
	H245	1.909104240	8.510387318	1.538618861
	H246	-3.199282262	3.893185285	-1.829208862
	H247	-4.477497425	4.742354992	-3.198639333
	H248	-4.292705977	5.879711410	-2.095343552
	H249	-4.603103535	2.094701736	-4.599857225
	H250	-6.348634326	2.027875794	-4.978581764
	H251	0.166917801	12.578837544	-0.504347614
	H252	-1.336443311	13.547619466	-0.424729751
	H253	0.043825191	10.342568380	-1.268298401
	H254	-1.478149735	9.530877710	-1.467567956
	H255	-1.388271340	9.927339149	-4.893143357
	H256	-1.235950550	5.270657652	-5.256402655
	H257	-6.931384511	8.881085427	-0.632256954
	H258	-2.980469185	8.510773463	2.370792020
	H259	-3.604937327	8.204614899	-2.764227979
	H260	2.003102330	5.375539741	-2.565583785
	H261	3.279863364	7.281019807	-1.745655018
	H262	-0.511963708	5.086769858	-2.676486506
	H263	-7.877942558	-0.153424276	-2.638189265
	H264	4.555055314	10.158003719	-2.353204582
	H265	-5.510855249	-0.408315201	-4.197043674
	H266	1.943722347	6.398771284	-4.002300671
	H267	-3.003612101	7.293835212	0.224584024
	H268	-6.420768099	3.737402548	-3.183494883
	H269	3.994498892	10.534748076	-0.713111433
	H270	-0.682342623	8.533829551	-5.610480965
	H271	-3.253393538	9.711880960	-3.557118846
	H272	-4.463842618	-2.955019528	-4.682901683
	H273	-5.486322213	-3.032345570	-2.174726658
	H274	-6.857590590	10.196685902	1.467816059
	H275	-5.094666554	1.687184004	-2.194227566
	H276	0.620814557	8.115229429	-2.773809758
	H277	-0.160741151	6.657678911	-5.274982123
	H278	0.448347527	4.405170728	-3.978767706
	H279	-2.593242572	6.291133391	-3.536131256
	H280	0.830275821	9.364598678	4.071486487
	H281	-0.326236489	10.383932285	1.136661338
	H282	-0.268114830	13.240604072	1.793534868
	H283	2.280355763	9.960551998	3.238822825
	H284	-2.392309297	10.814819088	2.672557635

	H285	6.065730016	-1.817340013	-8.579740326
	H286	5.914105874	-3.402738494	-7.801152999
	H287	8.553263337	-4.689058812	-4.354283086
	H288	6.687290557	-3.721571058	-10.033654149
	H289	5.115877400	-4.183346618	-10.051590652
	H290	5.890115240	4.962715108	1.006095954
	H291	5.682366608	7.545157809	2.707628761
	H292	5.713071694	8.315589246	-1.538201491
	H293	4.373336996	5.737124055	1.565311973
	H294	6.984892007	8.223220509	1.990860603
	H295	6.498379199	3.593764731	-5.355295732
	H296	6.643989054	7.050624324	-0.018534754
	H297	4.392380109	-7.359042931	4.813716373
	H298	6.516041771	-8.155595896	2.751736322
	H299	4.740409981	2.223697771	-5.793114299
	H300	4.140633240	-0.328359168	3.970973395
	H301	2.022029043	2.664388332	-8.111512761
	H302	-0.564888082	-1.843586834	-4.753223876
	H303	1.649849211	3.008216857	-6.422635512
	H304	3.458394580	-4.877432442	4.359703047
	H305	3.222859308	0.888071988	-5.995129925
	H306	3.589827535	-5.162487044	2.619560320
	H307	4.205042789	-7.446383319	3.048659684
	H308	6.035319929	-5.623218410	2.864724913
	H309	5.908361620	-5.420007389	4.625473916
	H310	-4.001350484	0.266049463	-7.208178477
	H311	5.035137046	-9.815250222	4.765211737
	H312	4.225625789	-12.058908446	2.846873338
	H313	0.941261817	0.002944791	-4.003367798
	H314	5.341958635	-12.206076306	5.540432298
	H315	3.819729051	-12.520316556	5.024309127
	H316	4.962542108	-3.230935100	2.366042755
	H317	0.396071390	2.336935704	-4.538061635
	H318	-2.534951227	-1.328577166	-6.171183482
	H319	7.710678338	-9.739158160	-1.433396888
	H320	7.093995095	-7.269458298	-3.584438658
	H321	6.384502830	-8.233579359	-0.029797690
	H322	7.143940678	-10.295047979	-2.758230527
	H323	6.443288904	3.609431908	-8.175297472
	H324	6.329055927	-5.105973278	0.056003143
	H325	2.255762195	-7.432797803	0.407078487
	H326	4.520448325	-1.060827654	-7.051081823
	H327	2.995911269	-8.971480051	-1.522445945
	H328	4.240926462	-5.854708328	-7.339647483
	H329	3.091165111	-4.638868425	-5.416813898
	H330	5.800234765	-12.719298366	3.298741703
	H331	4.610415800	-1.050081654	2.457600209
	H332	8.158323047	-6.023730365	1.331601305
	H333	7.026964758	-3.797604535	0.521099267
	H334	8.421504810	-7.264533314	3.349232558
	H335	3.721468902	-3.326490508	5.734458005
	H336	3.277142227	-11.139674237	-4.742264893
	H337	3.955904892	-7.703339083	-4.142397235
	H338	5.065782890	-8.146095741	-2.829590022
	H339	1.989233019	-8.252426482	-2.810151090
	H340	2.375981167	-1.337000703	-5.976777566
	H341	-1.576620170	2.875187665	-5.999194716
	H342	-3.777556161	2.028240448	-7.028770930
	H343	1.946258896	-5.720477593	-0.038194490

	H344	8.225956624	-7.545867477	1.037638084
	H345	6.990347627	-9.402578734	0.790752371
	H346	3.142870485	-1.701807931	5.661028761
	H347	6.557684017	-6.737941524	-6.663682806
	H348	6.890336747	-5.727428739	-2.760061884
	H349	1.713706365	-1.919805732	-7.532546831
	H350	5.458587958	5.000744037	-5.655312170
	H351	6.677634301	-0.768923136	5.241636385
	H352	7.160213872	-0.105438580	3.664212405
	H353	9.886809602	-6.095829665	-5.666726842
	H354	9.099994730	-7.453075349	-5.209444819
	H355	10.497720224	-8.513427808	-1.094040201
	H356	8.915878156	-7.943011228	-2.638213840
	H357	12.170863033	-7.115092973	-2.764682850
	H358	10.207693878	-6.905077974	-0.396617406
	H359	9.830313412	-0.674376923	4.193524797
	H360	-3.438914598	12.777519285	4.851214925
	H361	9.152223892	-7.607753521	6.250502300
	H362	8.932343942	-5.062610820	5.234454012
	H363	-4.902429787	12.245068365	2.267149973
	H364	-4.212339338	9.847409562	4.077333642
	H365	-5.843805273	10.455798877	3.746937977
	H366	-5.819738945	7.441553401	-1.977659413
	H367	1.350532417	-2.907847810	-0.939258826
	H368	2.422008630	-0.644565050	-0.048460991
	H369	0.194150480	-0.822094418	-0.221984549
	H370	-1.434318673	0.236942114	-1.145766886
	Mo371	4.051535176	-4.041646433	-2.273568882
	N372	-6.711687488	-6.782179977	-2.539006436
	N373	-8.974376799	-4.384738301	-0.774137678
	N374	-9.316738786	-5.066398093	1.957740117
	N375	-6.915832498	-6.375716913	2.579701829
	N376	-5.016947202	-4.254350061	2.246574953
	N377	-5.943995258	-2.538406676	4.324794461
	N378	-5.800768351	-4.155694923	6.723021784
	N379	-3.251256240	-5.484408727	6.745625797
	N380	-1.232266238	-3.751453074	5.879541394
	N381	-1.602560961	-0.867296078	6.519014419
	N382	-2.828214839	-1.139094169	9.147275516
	N383	4.607345534	-11.880763610	4.908719080
	N384	5.543261426	-9.570308753	3.907653822
	N385	4.538188545	-3.431836045	3.277461061
	N386	3.521196792	-2.507264579	5.170786611
	N387	4.128100565	-1.153526527	3.374556162
	N388	8.311880965	-7.474545683	4.365188308
	N389	-5.222049058	-2.416395507	-4.223666784
	N390	-1.257026402	-5.033236544	-4.072597411
	N391	-5.639751426	-0.015567231	-3.249888227
	N392	-8.099362629	0.748078886	-2.220660150
	N393	-7.830072001	0.418885135	0.519064332
	N394	-5.861670109	2.117202896	1.560191333
	N395	-2.923519665	2.990116341	-1.355518335
	N396	-2.075150392	1.039097787	-0.998803469
	N397	-6.590873874	4.643506883	2.448654457
	N398	-9.619672650	7.943142824	0.980387970
	N399	-9.763763295	9.539916538	2.514800471
	N400	8.943787715	1.699649616	4.636571057
	N401	6.436366827	7.359994936	2.038154374
	N402	4.996463323	8.705191568	-0.931481839

	N403	2.511646208	7.933991700	-1.965683065
	N404	-1.884482683	6.829375588	-4.062516214
	N405	-1.200453523	8.931712405	-4.834398272
	N406	-2.993145921	8.746541898	-3.377127797
	N407	1.072195725	6.659840595	0.145845832
	N408	1.070898420	8.703909316	2.095902441
	N409	-0.309889214	11.170094533	1.790574224
	N410	-0.971556441	10.340365418	-1.111232982
	N411	-2.911504271	11.684164316	2.574474769
	N412	5.730459363	-3.368555434	-9.966025711
	N413	3.758968443	-1.550793040	-7.535503036
	N414	2.634975993	1.180247369	-6.800010289
	N415	4.712586787	3.119876568	-6.296383594
	N416	0.062744601	-1.549091684	-10.469255270
	N417	-1.756636037	0.111709571	-9.025154650
	N418	8.967246283	-6.453266139	-5.392515245
	N419	4.874448214	-5.109318220	-4.311215551
	N420	4.660584981	-5.719471658	-6.423774140
	N421	9.584077871	-7.203032570	-2.416056583
	N422	0.353426003	2.779495350	-0.178871645
	N423	1.123809183	1.977365483	-0.381835893
	O424	-6.851610437	-5.033214240	-0.260347827
	O425	-8.244996668	-3.133409163	2.518351375
	O426	-7.294764262	-5.964259464	4.796542113
	O427	-3.806246326	-5.281788512	3.877028059
	O428	-3.798621690	-1.813043497	4.539356426
	O429	-5.225143291	-2.218977999	7.786364516
	O430	-3.411567423	-5.607824345	9.032188186
	O431	0.394493362	-5.279634397	6.433884650
	O432	0.618581036	-1.099428924	7.012651597
	O433	-1.254366650	0.372172932	9.778378160
	O434	-1.661192294	-1.957089970	12.438875887
	O435	6.388087282	-10.578358295	2.000275471
	O436	6.920885975	-8.255732385	6.019356778
	O437	10.890963646	-5.369837911	5.709469809
	O438	-5.484373322	-0.657204838	-1.053292261
	O439	-2.920954402	-3.845415583	-5.069032505
	O440	-7.357082783	2.877803777	-1.851949959
	O441	-5.701138143	3.671045634	-3.861586722
	O442	-9.975867983	1.170228225	0.868404241
	O443	-6.006623558	1.120978876	3.612078695
	O444	-4.592753632	4.886955260	3.529480085
	O445	-7.169494619	6.900599934	5.119212899
	O446	9.445539369	-1.524931207	6.010818706
	O447	3.046951025	4.238072475	5.126744819
	O448	3.942695161	8.592375506	1.083242953
	O449	1.792926309	10.113413879	-1.937932286
	O450	-0.842225837	7.269634853	-0.949894774
	O451	-0.751471829	7.737425230	3.091940146
	O452	2.229091750	5.839285387	2.829188948
	O453	0.706369609	12.019582747	3.661002707
	O454	-2.848824703	13.816540702	1.762302152
	O455	-2.907954294	11.551017243	-1.012767745
	O456	-5.453549135	13.076256085	4.697272033
	O457	-4.954557857	7.314379950	-1.542453097
	O458	3.199939152	-3.349516991	-8.851976999
	O459	1.200621191	0.403281128	-8.411796999
	O460	3.554781113	4.642219162	-7.525511733
	O461	7.368282457	5.277891391	-7.453703793

	O462	-3.402623527	-1.279448861	-9.773892712
	O463	-0.422800042	-5.676249201	-6.958574148
	O464	1.588340517	-4.782483098	-7.318592120
	O465	-2.383667051	3.563003006	-8.473913272
	O466	10.345156173	-5.083681636	-2.673752162
	O467	12.788299833	-6.810394375	-0.841875906
	O468	-0.205957469	-6.311890312	-1.446974040
	O469	0.136432879	-8.374176974	-0.693714483
	O470	5.220429870	-10.498675526	-3.716852070
	O471	3.096094352	-10.225162474	-4.435656723
	O472	5.140246281	-5.699051960	-1.200493761
	O473	4.785574973	-7.631684754	-0.094635752
	O474	2.820814138	-5.741638233	-2.426604217
	O475	8.641639743	-6.843562694	1.605585448
	O476	6.929942710	-4.741983867	0.755206411
	O477	-3.798847261	5.228988788	-2.652577762
	O478	7.988108606	-10.184098570	-2.274646846
	O479	7.240328449	-8.736979929	0.099231788
	S480	4.285503508	5.267230806	-0.812584386
	S481	2.791736487	2.373368618	-2.752126906
	S482	2.468632580	-2.757808459	-3.480279125
	S483	3.710959618	1.749270813	0.759207197
	S484	0.576297511	-0.654296764	-1.518754078
	S485	4.956140185	-0.032668827	-4.828590943
	S486	3.458051393	-3.400231915	-0.042963311
	S487	6.216224878	2.025459540	-1.812448967
	S488	6.053243164	-2.787102990	-2.576525289
	S489	6.218800587	-1.076959043	0.786289356
